# Entropic Stabilization of Cas4 Protein SSO0001 Predicted with Popcoen

**DOI:** 10.3390/e20080580

**Published:** 2018-08-07

**Authors:** Martin Goethe, Ignacio Fita, J. Miguel Rubi

**Affiliations:** 1 Department of Condensed Matter Physics, University of Barcelona, Carrer Martí i Franquès 1, 08028 Barcelona, Spain; 2 Department of Inorganic and Organic Chemistry, University of Barcelona, Carrer Martí i Franquès 1, 08028 Barcelona, Spain; 3 Molecular Biology Institute of Barcelona (IBMB-CSIC, Maria de Maeztu Unit of Excellence), Carrer Baldiri Reixac 4-8, 08028 Barcelona, Spain

**Keywords:** configurational entropy, entropy estimation, protein conformations, entropic stabilization, CRISPR/Cas system, oligomerization

## Abstract

*Popcoen* is a method for configurational entropy estimation of proteins based on machine-learning. Entropy is predicted with an artificial neural network which was trained on simulation trajectories of a large set of representative proteins. *Popcoen* is extremely fast compared to other approaches based on the sampling of a multitude of microstates. Consequently, *Popcoen* can be incorporated into a large class of protein software which currently neglects configurational entropy for performance reasons. Here, we apply *Popcoen* to various conformations of the Cas4 protein SSO0001 of *Sulfolobus solfataricus*, a protein that assembles to a decamer of known toroidal shape. We provide numerical evidence that the native state (NAT) of a SSO0001 monomer has a similar structure to the protomers of the oligomer, where NAT of the monomer is stabilized mainly entropically. Due to its large amount of configurational entropy, NAT has lower free energy than alternative conformations of very low enthalpy and solvation free-energy. Hence, SSO0001 serves as an example case where neglecting configurational entropy leads to incorrect conclusion. Our results imply that no refolding of the subunits is required during oligomerization which suggests that configurational entropy is employed by nature to largely enhance the rate of assembly.

## 1. Introduction

Over the past 40 years, computer simulations have been applied very successfully to gather insight of biological systems at the molecular level [1]. The most common computational approaches to study particular macromolecules, such as proteins, are based on molecular dynamics and Monte Carlo simulations [2]. Both techniques have in common that they sample a multitude of microstates of the protein which allows accurate unraveling the molecular mechanisms at play. However, the involved sampling is computationally very expensive which limits these approaches to studies of particular proteins while they are unfeasible for the screening of many, say hundreds or thousands, of proteins. A prominent example is protein design whose goal is to find an amino-acid sequence which folds spontaneously to a target protein structure. For target structures of realistic size, there is an enormously large number of possible sequences. Screening these candidates, or even only a reasonable subset of them, requires software which cannot afford to sample various microstates per candidate [3].

For such screenings, a third class of protein software is employed which is grounded on the fact that the equilibrium state of a physical system is the one of lowest free energy. A cost function $\hat{G}$ is defined which maps a given protein structure onto an approximation of the free energy *G* of the protein folded in this conformation and surrounded by solvent. The native state of the protein is computed by minimizing $\hat{G}$ using elaborated minimization strategies. A fundamental problem of this approach emerges from the missing information about thermal motion. While *G* depends on the spatial fluctuations and correlations of the protein atoms, this information is absent in the protein structure. Hence, by construction, no cost function $\hat{G}$ can represent *G* exactly. Nevertheless, $\hat{G}$ may approximate *G* to sufficient accuracy.

The exact free energy *G* of the protein in solvent can be decomposed to excellent approximation into three important terms, namely the average intramolecular enthalpy $E_{\mathrm{intra}}$, the average solvation free-energy $G_{\mathrm{solv}}$, and the configurational entropy $S_{\mathrm{conf}}$ (multiplied by the negative temperature) [4].

(1) $$G\approx E_{\mathrm{intra}}+G_{\mathrm{solv}}-TS_{\mathrm{conf}}+\mathrm{const}.$$

The constant does not depend on the protein conformation, and hence does not impact the native-state selection. $E_{\mathrm{intra}}$ and $G_{\mathrm{solv}}$ can be expressed reasonably well in terms of the protein structure by neglecting specific fluctuation-induced effects [5]. Existing protein software usually model these two terms within their cost functions $\hat{G}$. In contrast, $S_{\mathrm{conf}}$ is much less accessible from the protein structure since $S_{\mathrm{conf}}$ depends crucially on fluctuations and correlations of the protein atoms. As a consequence, protein software based on cost-function minimization either account for $S_{\mathrm{conf}}$ only rudimentarily [6,7] or neglect it entirely [8,9,10,11,12,13]. This, however, represents a rather crude approximation since $S_{\mathrm{conf}}$ can have strong influence on the native-state selection of proteins [14,15,16,17].

Recently, our group developed a machine-learning approach called *Popcoen* for $S_{\mathrm{conf}}$ estimation solely from the protein structure [18]. $S_{\mathrm{conf}}$ is derived by evaluating an artificial neural network for various features measured from the protein structure (see Section 4.3). The network was trained in a supervised manner using molecular-dynamics simulations of about 1000 representative proteins. Entropy estimation is extremely fast compared to alternative approaches [17,19,20,21,22,23,24,25,26,27] since it does not involve the sampling of microstates. Therefore, *Popcoen* offers a way to incorporate $S_{\mathrm{conf}}$ into protein software without compromising their runtime. This can significantly improve the accuracy of these software packages [18].

In this work, we employ *Popcoen* to study the Cas4 protein SSO0001 of *Sulfolobus solfataricus*. Cas4 is one family of the CRISPR associated (Cas) proteins which are located in close proximity to the CRISPR (clustered regularly interspaced short palindromic repeats) region in the DNA of prokaryotes [28,29]. The CRISPR/Cas system represents an adaptive protection mechanism of prokaryotes against viruses and other foreign genetic material. Immune response is organized in three steps, all performed by specific Cas proteins. After the first invasion of the virus, specific viral DNA segments are captured and inserted into the CRISPR sequence. This allows the cell to “remember” the invader. The CRISPR sequence is transcribed and post-processed to CRISPR–RNA. In a subsequent infection, Cas nucleases can now degrade foreign DNA identified on the basis of CRISPR–RNA [28,29]. Cas4 proteins are nucleases involved in the acquisition of new genetic information to CRISPR [30]. The structure of SSO0001 has been solved [31]. SSO0001 forms a decamer of toroidal structure into which various cofactors (manganese ions and iron/sulfur clusters) are integrated. At the active center (situated in the hole of the torus), double-stranded DNA can be unwound and single-stranded DNA can be cleaved. The precise role of SSO0001 within the CRISPR/Cas system remains unknown. Understanding CRISPR/Cas is of specific importance since it allows for controlled genome editing in a (relatively) simple manner which offers a broad range of biotechnological applications [29].

Here, we provide numerical evidence that the native state of a SSO0001 monomer adopts the same conformation as the protomers of the oligomer. This allows for efficient oligomerization without refolding. The monomer is stabilized mainly by its large amount of configurational entropy while various decoy conformations of very low enthalpy have negligible contributions to the equilibrium state due to their low amount of $S_{\mathrm{conf}}$. Hence, the SSO0001 monomer represents an example for which protein software neglecting $S_{\mathrm{conf}}$ fail to identify the native state.

## 2. Results

**Figure 1:**
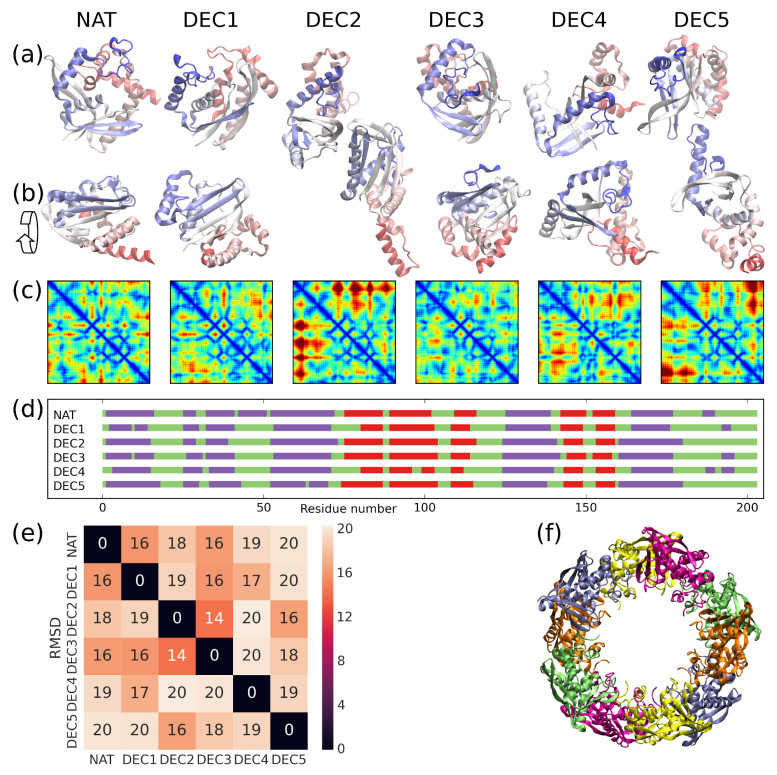
*Structure characterization:* (**a**) Cartoon representation of the six structures NAT, DEC1, DEC2, ..., DEC5. The scaffold is colored from N-terminus to C-terminus in red to white to blue. (**b**) Same as Panel (a) but rotated by 90 degrees along the shown arrow. (**c**) Contact maps of the structures. Colors indicate $C_{\alpha }$ distances in the range from 0 (dark blue) to $50\;\overset{˚}{A}$ (dark red). (**d**) Secondary structure of the structures where helical, extended, and coil regions are colored in violet, red, and green, respectively. (**e**) Root-mean-square deviation (RMSD) in units of $\overset{˚}{A}$ between the conformations. (**f**) Oligomer of ten SSO0001 proteins [31]. Structure obtained from PDB databank (pdb-code 4IC1).

We compare the native state of SSO0001 with five decoy states. The structure of SSO0001 was solved [31] inside a decamer of toroidal shape (pdb-code 4IC1; shown in Figure 1f). All protomers have indistinguishable conformation within the experimental resolution (resolution = $2.35\;\overset{˚}{A}$, mutual root-mean-square deviation (RMSD) in range 0.6–$1.0\;\overset{˚}{A}$). The structure of the entirely resolved protomer (chain D) is referred to as the native state (NAT) of a SSO0001 monomer. This nomenclature is justified by the analysis below. Alternative conformations of the protein were obtained from the data repository of the protein-structure prediction competition CASP10 [32]. The stability of 217 distinct decoy structures was computed in terms of three cost-functions ($E_{\mathrm{intra}}$, $G_{0}$ and $\hat{G}$, defined below). Most decoys were found to represent high-energy states with negligible contribution to the equilibrium state of the protein at ambient conditions. Five conformations were identified having low energy with respect to at least one of the cost functions (see Section 4.1). In the following, we focus our attention to these decoys, referred to as DEC1, DEC2, ..., DEC5.

Figure 1 illustrates the six structures. They are composed of similar secondary structure (see Figure 1d) with one notable deviation for residues 43–51, which form a helix in NAT but do not adopt regular secondary structure in the decoys. Albeit these similarities, all structures are mutually very distinct with RMSDs in the range of 14–$20\;\overset{˚}{A}$ (see Figure 1e) because of their dissimilar secondary-structure arrangement. The different tertiary structures are shown using a cartoon representation of the protein backbone (Figure 1a,b), and can be appreciated by comparing the associated contact maps (Figure 1c).

We computed the average intramolecular enthalpies $E_{\mathrm{intra}}$ of all structures using the prominent protein software tool *FoldX* [6]. The values are reported in Table 1 in units of kcal/mol (where $k_{B}T\approx 0.6$ kcal/mol at T=300 K). The decoys DEC1, DEC2, and DEC3 have significantly lower enthalpy than NAT ($\Delta E_{\mathrm{intra}}\approx$ 6 kcal/mol = $10\;k_{B}T$) while their mutual differences are of the order of $k_{B}T$. Therefore, if $E_{\mathrm{intra}}$ is used as cost function, one predicts the native state to be a mixture of DEC1, DEC2, and DEC3 with similar weights of about 0.65, 0.19, and 0.16, respectively, at room temperature. NAT would be assigned negligible contribution to the native state (having a weight of about $10^{-5}$).

The solvation free-energies $G_{\mathrm{solv}}$ of all structures were also computed with *FoldX* (see Table 1) and the structures were ranked in terms of the cost-function $G_{0}\equiv E_{\mathrm{intra}}+G_{\mathrm{solv}}$ which contains all significant contributions of *G* except $S_{\mathrm{conf}}$. DEC3 has lowest $G_{0}$, followed by NAT, DEC5 and DEC2. The energy differences $\Delta G_{0}$ yield the weights 0.58, 0.30, 0.11, and 0.006, for these states, respectively. Hence, $G_{0}$ predicts that the native state is a mixture of various conformations including NAT. Again, NAT is not identified as the predominant state of SSO0001.

Finally, configurational entropy $S_{\mathrm{conf}}$ was also incorporated into the cost function. For this end, we employed *Popcoen* [18] which is a new method for entropy estimation based on machine-learning. For a given input structure, *Popcoen* outputs the so-called partial entropy $S_{i}$ for each residues *i* ($i=1,\ldots ,N_{\mathrm{res}}$; $N_{\mathrm{res}}$ = number residues) which estimates the contribution of $S_{\mathrm{conf}}$ stemming from residue *i*. From the $S_{i}$s, we obtain $S_{\mathrm{conf}}=\sum_{i=1}^{N_{\mathrm{res}}}S_{i}$ up to an unimportant constant (see Section 4.3). The values of $\left(-T\right)S_{\mathrm{conf}}$ are given in Table 1. NAT and DEC4 have substantially more configurational entropy than all other conformations with an associated free-energy separation $\left(-T\right)\Delta S_{\mathrm{conf}}$ of about 7–8 kcal/mol. The total free-energy $\hat{G}=E_{\mathrm{intra}}+G_{\mathrm{solv}}-TS_{\mathrm{conf}}$ is also listed in Table 1. NAT has lowest $\hat{G}$, separated by more than 7.6 kcal/mol = $12.6\;k_{B}T$ from the second lowest structure (DEC5). It follows that NAT is the predominant state with an associated weight of more than 0.99999. Hence, at ambient conditions, only NAT contributes significantly to the equilibrium state such that it is meaningful to denote NAT as the native state of the protein.

**Figure 2:**
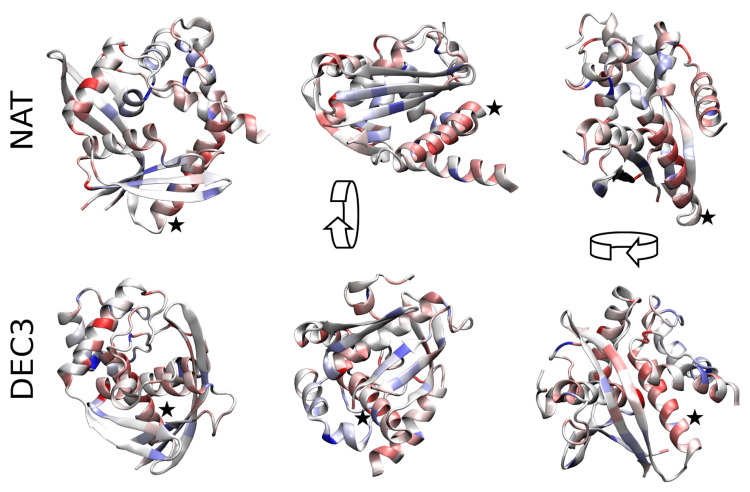
*Configurational entropy differences.* The entropy differences $\Delta S_{i}$ between the native conformation and DEC3 are represented using a color code on the scaffold of the structures. Residues colored in dark red have $1.3\;k_{B}$ more entropy in NAT than in DEC3 while residues in dark blue have $1.4\;k_{B}$ less entropy. Pale colors show intermediate values on a linear scale. Both structures are shown from three different perspectives (obtained via 90-degree rotation along the shown arrows). NAT is significantly more loosely packed than DEC3 which involves a total entropy difference of $14.6\;k_{B}$. The Helix formed by residues 53–72 (indicated by an asterisk) accounts for 44% of that difference. It is widely exposed in NAT while buried in DEC3. (For 3D visualization, see Supplementary Materials.)

To assess why NAT is entropically stabilized with respect to DEC3 (i.e., the decoy with lowest $G_{0}$), backbone representations of both structures are shown in Figure 2. The color encodes the differences $\Delta S_{i}$ between the partial entropies in both conformations where amino acids colored in dark red have $1\;k_{B}$ more entropy in NAT than in DEC3 while ones colored in dark blue have $1\;k_{B}$ less entropy. In general, NAT is significantly more loosely-packed than DEC3 (radius of gyration =17.2 vs. $16.3\;\overset{˚}{A}$; solvent-accessible surface area = 12.5k vs. $11.5k\;{\overset{˚}{A}}^{2}$) which allows for more configurational entropy. In particular, the helix formed by residues 53–72 (indicated with an asterisk) is buried in DEC3 while it is largely exposed in NAT. Summing the $\Delta S_{i}$’s of these residues gives that this helix has $\sum_{i=53}^{72}\Delta S_{i}=6.4\;k_{B}$ more entropy in NAT than in DEC3, which alone accounts for 44% of the total entropy difference between the conformations.

## 3. Discussion

We analyzed the stability of a single SSO0001 monomer in solvent. The conformation NAT was found to have much lower free energy than all alternative conformations of the CASP dataset (with an energy separation of at least 7.5 kcal/mol). NAT is a relatively open structure which is mainly stabilized by its large amount of $S_{\mathrm{conf}}$ with respect to alternative conformations of much lower enthalpy.

In vitro, ten SSO0001 proteins assemble to an oligomer of toroidal structure (Figure 1f) which is the biologically relevant conformation involved in the CRISPR/Cas mechanism [31,33]. The structures of all protomers of the compound are identical to NAT. This leads us to the following hypothesis. After SSO0001 is translated by the ribosome, it spontaneously folds into NAT stabilized by configurational entropy. Then, ten monomers and specific cofactors (ten manganese ions and iron/sulfur clusters) assemble to the oligomer where the precise mechanism of assembly requires further investigation. However, since each subunit has the correct structure already prior to oligomerization, this process does not involve significant refolding. Refolding is usually a very slow process and often requires the assistance of specific helper proteins (chaperones) [34]. Therefore, efficient oligomer formation is rendered possible by the entropic stabilization of NAT for the individual subunits.

Our analysis is based on the assumption that the CASP dataset contains all low energy conformations of SSO0001. A similar assumption is made very generally in computational studies of proteins since no available method can guarantee that a given state has lowest free energy (given the force field) for proteins of realistic size. However, since the dataset is composed of models derived with a variety of methods by the large number of 61 research groups and 64 prediction servers, we believe that the chance of having missed important alternative conformation is low.

Our analysis required the estimation of $S_{\mathrm{conf}}$ for hundreds of conformations which would involve massive computational effort with methods for $S_{\mathrm{conf}}$ estimation based on the sampling of microstates. On the other hand, $S_{\mathrm{conf}}$ is found to have essential impact on the native-state selection of SSO0001 such that an analysis without considering $S_{\mathrm{conf}}$ leads to incorrect conclusions. *Popcoen* allowed us to compute $S_{\mathrm{conf}}$ efficiently on a single CPU during a couple of minutes. This revealed the entropic stabilization of NAT.

## 4. Materials and Methods

### 4.1. Decoy Structures Acquisition

The decoy structures were obtained from the protein structure prediction competition CASP [32]. The participants of CASP10 submitted in total 523 models for the amino-acid sequence of SSO0001 (referred to as target *T0720* within CASP). We downloaded all models and processed them in the following way. Models for incorrect amino-acid sequence were dropped. Models with missing side-chain information were dropped. Unfolded models (submitted by prediction servers without human supervision) were dropped. Using hierarchical clustering, groups of very similar models (with mutual RMSD $<5\;\overset{˚}{A}$) were reduced to a single representative structure chosen randomly. Hydrogens were added using *FoldX*. As described below, the free-energy contributions $E_{\mathrm{intra}}$, $G_{\mathrm{solv}}$, and $S_{\mathrm{conf}}$ were computed for the set M of the remaining structures (composed by NAT and 217 models). The weights

(2) $$w_{X}\left(k\right)=\frac{\exp(-\beta X(k))}{\sum_{i\in M}\exp\left(-\beta X\left(i\right)\right)}$$

were derived for all structures k∈M and for the three cost-functions $X\in \{E_{\mathrm{intra}},G_{0},\hat{G}\}$, where $\beta ={\left(k_{B}T\right)}^{-1}$. The smallest subset U⊂M was identified which guaranteed that $\sum_{k\in U}w_{X}\left(k\right)>1-10^{-7}$ for all three cost-functions $X\in \{E_{\mathrm{intra}},G_{0},\hat{G}\}$. All structures k∉U were dropped since their contribution to the equilibrium state is negligible (for details, see Figures S1 and S2 of Supplementary Materials). In this way, we obtained the decoys DEC1, DEC2, ... DEC5 (shown in Figure 1) from the models denoted as *T0720TS172_3*, *T0720TS195_5*, *T0720TS492_2*, *T0720TS172_2*, and *T0720TS195_4* in the CASP dataset, respectively.

### 4.2. Free-Energy Calculation

Free-energies were computed for 218 structures. $E_{\mathrm{intra}}$ and $G_{\mathrm{solv}}$ were obtained with *FoldX* (FoldX Consortium, Barcelona, Spain, Version 4) by applying the *FoldX* protocols *RepairPDB* and *Stability* to the structures [35]. From the output, we identified $E_{\mathrm{intra}}$ as the sum of the *FoldX* energy terms denoted as *BackHbond*, *SideHbond*, *Energy_VdW*, *Electro*, *Energy_vdwclash*, *energy_torsion*, *helixdipole*, *cis_bond*, *disulfide*, *Energy_Ionisation*. $G_{\mathrm{solv}}$ is the sum of the *FoldX* energy terms *Energy_SolvP*, *Energy_SolvH*.

Configurational entropies $S_{\mathrm{conf}}$ were computed with *Popcoen* (see below). The calculation for all 218 structures required 130 seconds on a single CPU (*Intel i5-6500*, Intel Corporation, Santa Clara, CA, USA). This is very quick compared to sampling approaches for estimating $S_{\mathrm{conf}}$. For example, measuring $S_{\mathrm{conf}}$ from molecular-dynamics simulations is about 60,000 times slower even when computed on much more powerful architecture (a 10 ns trajectory of SSO0001 in explicit water requires about 10 h on a state-of-the-art graphics card (*Nvidia GeForce GTX1060*, Nvidia Corporation, Santa Clara, CA, USA)).

### 4.3. Popcoen

Configurational entropy was estimated using the software tool *Popcoen* recently developed in our group [18]. *Popcoen* is a machine-learning approach based on an artificial neural network which was trained on molecular-dynamics trajectories (obtained from the MoDEL database [36]) of about 1000 representative proteins. Entropy is predicted from the protein structure in two calculation steps. First, various structural features per amino-acid are measured from the input structure (such as residue type, burial level, local density profile, relative and total solvent accessible surface area, average torsion angles, local and total number of hydrogen bonds, properties of the gyration tensor, and $N_{\mathrm{res}}$). Second, the neural network is evaluated for the features giving an estimate for the partial entropies $S_{i}$ ($i=1,...N_{\mathrm{res}}$) and for

(3) $$S_{\mathrm{conf}}+C=\sum_{i=1}^{N_{\mathrm{res}}}S_{i}$$

where *C* is a constant for fixed amino-acid sequence which always cancels out in this work. In [18], *Popcoen*’s prediction accuracy is assessed with a test set of about 100 representative proteins. It is further shown that incorporating *Popcoen* into *FoldX* improves *FoldX*’s accuracy for native-state identification.

*Popcoen* exploits patterns of how spatial fluctuations and correlations typically occur inside proteins. For example, amino acids on the surface of the proteins usually fluctuate stronger than in the bulk (mainly due to the large steric constraints in the bulk); and adjacent amino acids in regular secondary structure are typically stronger correlated than in coil regions [37,38]. During training of the neural network in a supervised-learning fashion, such patterns were automatically extracted from the simulation trajectories.

*Popcoen* relies on an approximation of entropy similar to the maximum information spanning tree approximation [23]. Within the approximation, entropy can be decomposed into the sum of the partial entropies (Equation (3)). The partial entropy $S_{i}$ of a residue *i* is basically defined as the sum of the marginal entropies of all torsion angles belonging to the residue, minus all mutual informations between pairs of these torsions which are adjacent in terms of the covalent structure. The precise definition is given in [18]. It also accounts for mutual information between backbone torsions of adjacent residues, and special conditions at the chain ends.

### 4.4. Structure Characterization

Structure visualizations were generated with *VMD* [39]. Secondary structure and solvent accessible surface area were computed with the *mdtraj* implementation [40] of the DSSP [41] and the Shrake–Rupley algorithm [42]. Structures were aligned with *mdtraj*. Hierarchical clustering was performed with the *sciPy* implementation of the unweighted pair group method with arithmetic mean (UPGMA) [43] with a hard threshold of $5\;\overset{˚}{A}$ as cluster separation criterion.

## Figures and Tables

**Table 1:** Energy values in units of kcal/mol (with 1 kcal/mol ≈$1.67\;k_{B}T$ at T=300 K) of the six conformations NAT, DEC1, DEC2, ..., DEC5. $E_{\mathrm{intra}}$, $G_{\mathrm{solv}}$, and $S_{\mathrm{conf}}$ represent the average intramolecular enthalpy, the average solvation free-energy, and the configurational entropy, respectively. $G_{0}=E_{\mathrm{intra}}+G_{\mathrm{solv}}$; $\hat{G}=G_{0}-TS_{\mathrm{conf}}$. For better comparability, all values of each column are shifted such that the lowest value equals zero. The numbers in brackets are the ranks of the values in each column. *FoldX* [6] was employed for $E_{\mathrm{intra}}$ and $G_{\mathrm{solv}}$; *Popcoen* [18] was employed for $S_{\mathrm{conf}}$.

Conformation	$E_{\mathrm{intra}}$	$G_{\mathrm{solv}}$	$-T\;S_{\mathrm{conf}}$	$G_{0}$	$\hat{G}$
NAT	6.6 [5]	1.3 [2]	0.0 [1]	0.4 [2]	0.0 [1]
DEC1	0.0 [1]	17.0 [6]	7.3 [5]	9.5 [5]	16.4 [6]
DEC2	0.8 [2]	9.5 [4]	6.6 [3]	2.8 [4]	9.0 [4]
DEC3	0.9 [3]	6.6 [3]	8.7 [6]	0.0 [1]	8.3 [3]
DEC4	5.6 [4]	14.0 [5]	0.5 [2]	12.1 [6]	12.2 [5]
DEC5	8.5 [6]	0.0 [1]	7.0 [4]	1.0 [3]	7.6 [2]

## Supplementary Materials

Supplementary materials are available at http://www.mdpi.com/1099-4300/20/8/580/s1.
