# Supplementary material for: Entropic Stabilization of Cas4 Protein SSO0001 Predicted with Popcoen
Source: Entropy (Basel). 2018 Aug 7;20(8):580. doi: 10.3390/e20080580 (PMC7513108; doi:10.3390/e20080580)
Supplement: Supplementary file 1 [file entropy-20-00580-s001.pdf]

Supplementary Material for "Entropic Stabilization of Cas4 Protein SSO0001 predicted with Popcoen"

Martin Goethe,* Ignacio Fita, and J. Miguel Rubi

E-mail: martingoethe@ub.edu

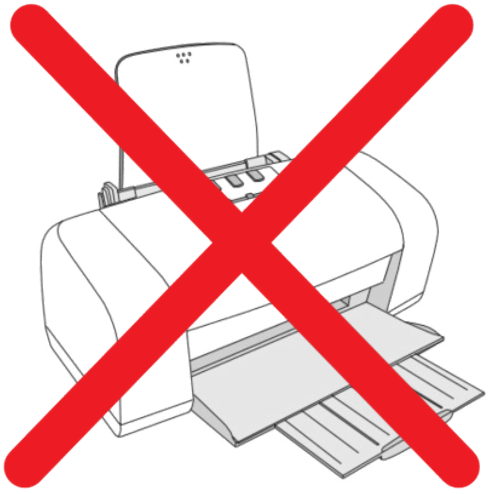

This document contains 90 pages or raw data.

Please consider this when sending it to your printer.

Supplementary Figures

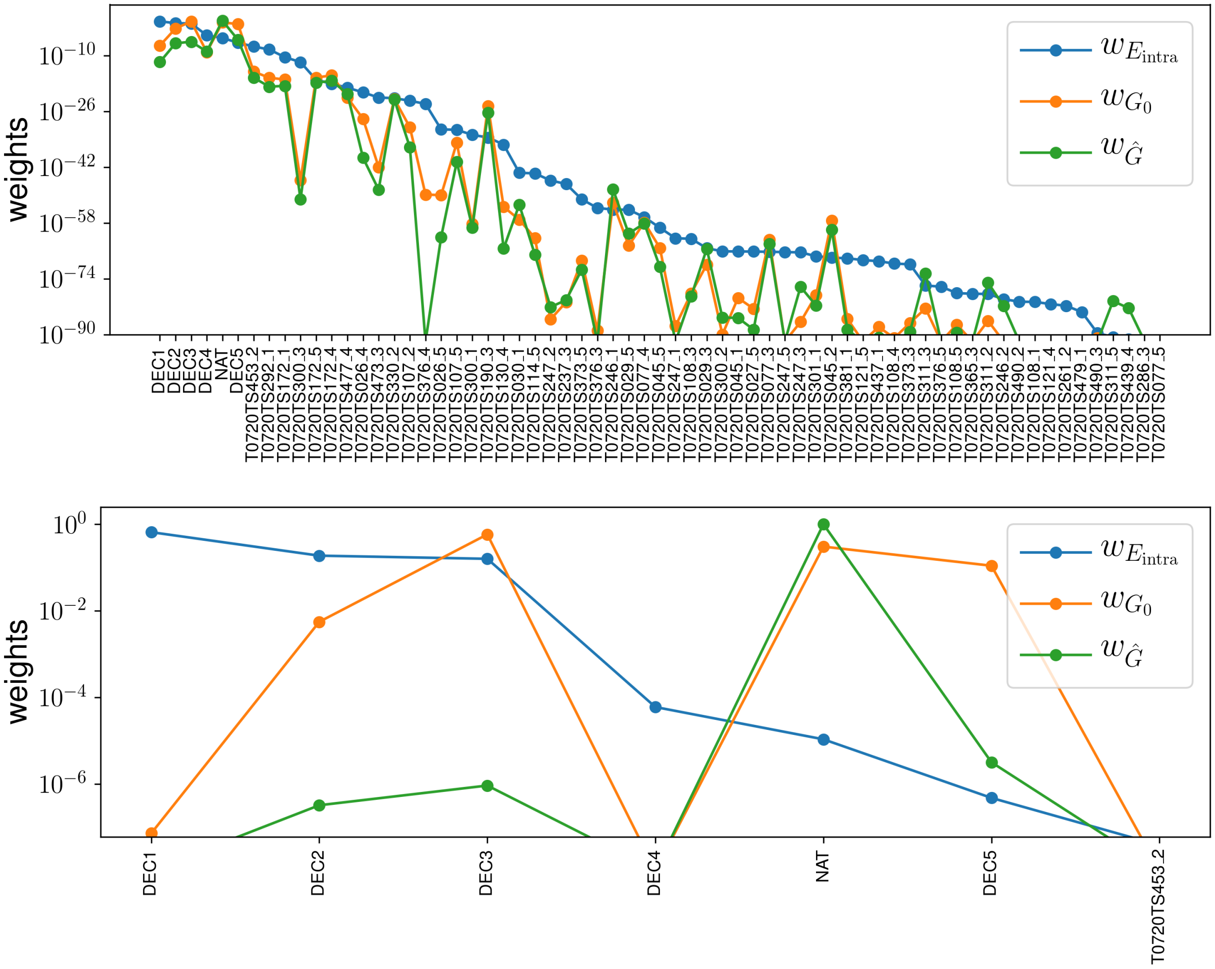

Figure S1: The weights $w_{E_{\mathrm{intra}}}$, w_{G_0}, and $w_{\hat{G}}$ (of Eq. 2) for all structures with weights larger 10^{-90}. The top panel shows the range $[10^{-90}, 1]$ and the bottom panel a zoom to the range $[10^{-7}, 1]$. Most of the CASP-decoys are high-energy states which have only negligible contribution to the equilibrium state of the protein at ambient conditions. In the Result section, we limit our considerations to six states (NAT, DEC1, ... DEC5) because the accumulated weights of these states exceed $1 - 10^{-7}$ for all three cost functions. NAT has a weight $w_{\hat{G}} > 0.99999$. Therefore, it is meaningful to denote NAT as the native state of the protein. The weight $w_{\hat{G}}$ is smaller than w_{G_0} for all decoys with reasonable values w_{G_0}. Therefore, configurational entropy stabilized the native state of the protein against decoy states of low G_0.

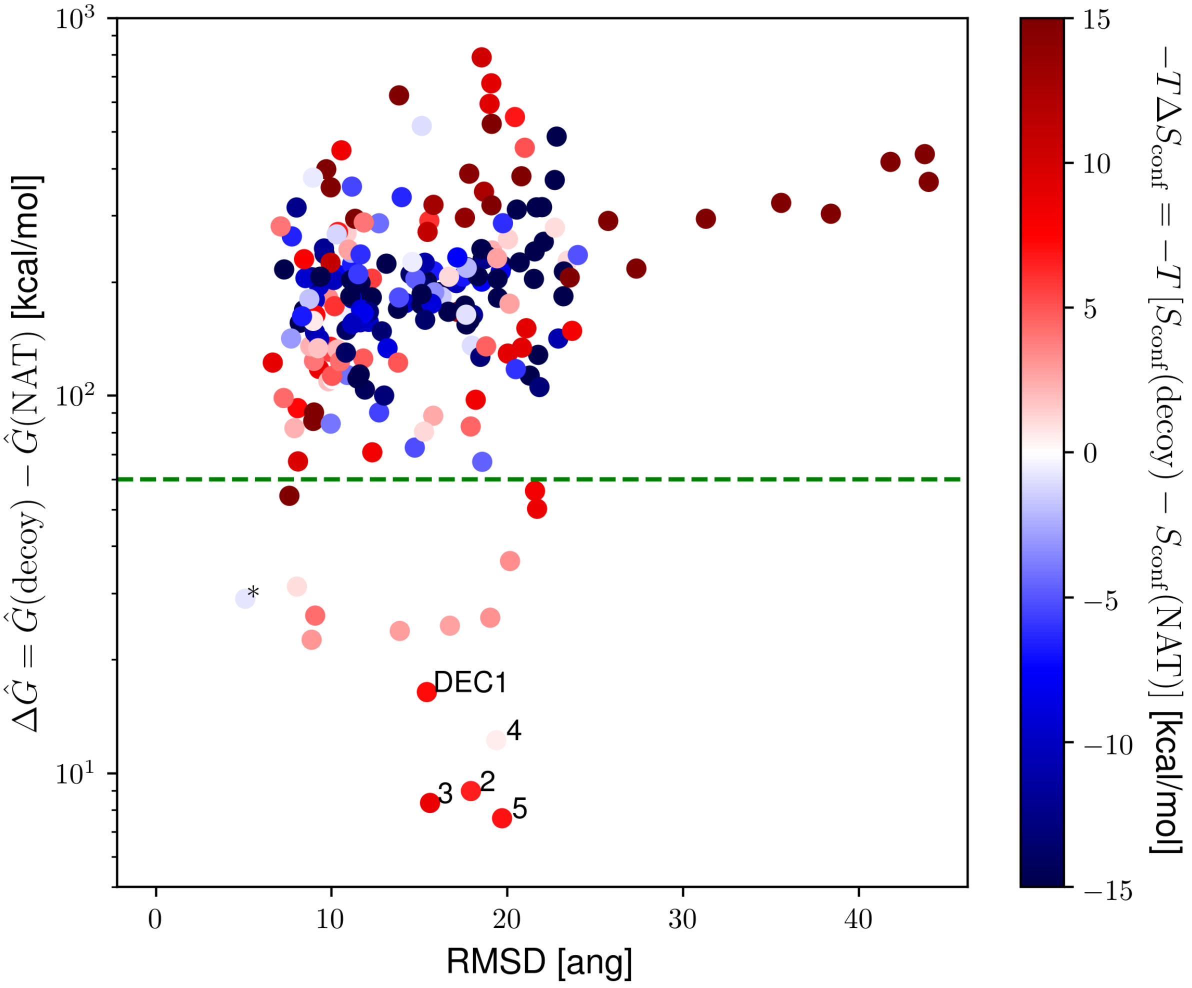

Figure S2: Scatter plot of RMSD vs. $\Delta\hat{G}$ for all 217 decoys where RMSD denotes the root-mean-square deviation between NAT and the decoys, and $\Delta\hat{G}$ is the difference of $\hat{G}$ between the decoys and NAT. DEC1, ..., DEC5 are labeled with DEC1, 2, ... 5. The color code represents $-T\Delta S_{\text{conf}}$. This shows that NAT has more configurational entropy then basically all decoys with $\Delta\hat{G} < 60$ kcal/mol (indicated with the dashed line). (The exception is decoy T0720TS477_4 with $-T\Delta S_{\text{conf}} = -0.78$ kcal/mol labeled with an asterisk.) This shows that configurational entropy stabilizes NAT against other decoy states of low G_0.

3D visualization of ΔS_i

Fig. 2 of the article shows the scaffold of NAT and DEC3. The color represents the partial entropy differences ΔS_i between the conformations. The raw data for such plots are given below. This allows to easily reproduce the images in 3D using VMD.

Ten pdb files are listed below. The file names are of the format `XXX_vs_YYY_dSi_colored.pdb` where either `XXX` or `YYY` equals NAT and the other equals a decoy name. The pdb file represents the structure of `XXX`. The occupancy records of the pdb file are modified to (rescaled values) of dSi = S_i(DEC) − S_i(NAT). Note that dSi = $-\Delta S_i$. (By turning the sign, VMD represents residues with more entropy in NAT than in the decoy with red colors). The pdb files can be extracted from this .pdf document (using e.g. `pdftotext`) and loaded into VMD. The representation of Fig. 2 was generated by chosing `"Occupancy"` as the `"Coloring Method"` and `"NewCartoon"` as `"Drawing Method"`. The used linear scaling between occupancy values and dSi is outlined in the header of each pdb file.

```
REMARK   1 *********************************************************************
REMARK   1 Start File DEC1_vs_NAT_dSi_colored.pdb
REMARK   1 The occupancy record was modified to represent dSi := Si(DEC)-Si(NAT).
REMARK   1 Occ=0.00 means dSi=-1.507170 kB; Occ=99.99 means dSi=1.490229 kB.
MODEL        0
ATOM      1  N   MET A   1       5.474  16.109  -4.355 35.21  0.00           N
ATOM      2  CA  MET A   1       6.038  15.157  -3.406 35.21  0.00           C
ATOM      3  C   MET A   1       7.504  14.877  -3.712 35.21  0.00           C
ATOM      4  O   MET A   1       7.930  14.938  -4.865 35.21  0.00           O
ATOM      5  N   ILE A   2       8.272  14.570  -2.672 31.82  0.00           N
ATOM      6  CA  ILE A   2       9.694  14.291  -2.827 31.82  0.00           C
ATOM      7  C   ILE A   2       9.949  12.797  -2.982 31.82  0.00           C
ATOM      8  O   ILE A   2       9.316  11.977  -2.316 31.82  0.00           O
ATOM      9  N   THR A   3      10.878  12.449  -3.865 27.55  0.00           N
ATOM     10  CA  THR A   3      11.218  11.053  -4.109 27.55  0.00           C
ATOM     11  C   THR A   3      11.486  10.315  -2.804 27.55  0.00           C
ATOM     12  O   THR A   3      10.894   9.269  -2.539 27.55  0.00           O
ATOM     13  N   GLU A   4      12.381  10.867  -1.992 41.92  0.00           N
ATOM     14  CA  GLU A   4      12.747  10.248  -0.722 41.92  0.00           C
ATOM     15  C   GLU A   4      11.565  10.224   0.239 41.92  0.00           C
ATOM     16  O   GLU A   4      11.391   9.271   0.998 41.92  0.00           O
ATOM     17  N   PHE A   5      10.757  11.277   0.201 41.07  0.00           N
ATOM     18  CA  PHE A   5       9.560  11.354   1.031 41.07  0.00           C
ATOM     19  C   PHE A   5       8.653  10.153   0.801 41.07  0.00           C
ATOM     20  O   PHE A   5       8.264   9.466   1.746 41.07  0.00           O
ATOM     21  N   LEU A   6       8.318   9.902  -0.461 17.98  0.00           N
ATOM     22  CA  LEU A   6       7.410   8.817  -0.813 17.98  0.00           C
ATOM     23  C   LEU A   6       8.047   7.459  -0.547 17.98  0.00           C
ATOM     24  O   LEU A   6       7.369   6.512  -0.149 17.98  0.00           O
ATOM     25  N   LEU A   7       9.353   7.370  -0.772 29.60  0.00           N
ATOM     26  CA  LEU A   7      10.096   6.144  -0.500 29.60  0.00           C
ATOM     27  C   LEU A   7      10.037   5.783   0.979 29.60  0.00           C
ATOM     28  O   LEU A   7       9.849   4.620   1.336 29.60  0.00           O
ATOM     29  N   LYS A   8      10.200   6.786   1.835 47.82  0.00           N
ATOM     30  CA  LYS A   8      10.158   6.576   3.277 47.82  0.00           C
ATOM     31  C   LYS A   8       8.737   6.305   3.753 47.82  0.00           C
ATOM     32  O   LYS A   8       8.528   5.698   4.805 47.82  0.00           O
ATOM     33  N   LYS A   9       7.760   6.759   2.975 30.34  0.00           N
ATOM     34  CA  LYS A   9       6.356   6.533   3.295 30.34  0.00           C
ATOM     35  C   LYS A   9       5.938   5.106   2.961 30.34  0.00           C
ATOM     36  O   LYS A   9       5.088   4.525   3.636 30.34  0.00           O
ATOM     37  N   LYS A  10       6.539   4.548   1.915 26.27  0.00           N
ATOM     38  CA  LYS A  10       6.268   3.171   1.521 26.27  0.00           C
ATOM     39  C   LYS A  10       7.299   2.217   2.110 26.27  0.00           C
ATOM     40  O   LYS A  10       7.264   1.014   1.852 26.27  0.00           O
```

```
ATOM     41  N   LEU A  11       8.216   2.760   2.903 25.05  0.00           N
ATOM     42  CA  LEU A  11       9.294   1.968   3.483 25.05  0.00           C
ATOM     43  C   LEU A  11       8.751   0.735   4.193 25.05  0.00           C
ATOM     44  O   LEU A  11       9.351  -0.338   4.140 25.05  0.00           O
ATOM     45  N   GLU A  12       7.612   0.895   4.859 18.67  0.00           N
ATOM     46  CA  GLU A  12       7.005  -0.195   5.612 18.67  0.00           C
ATOM     47  C   GLU A  12       6.632  -1.355   4.697 18.67  0.00           C
ATOM     48  O   GLU A  12       6.660  -2.516   5.107 18.67  0.00           O
ATOM     49  N   GLU A  13       6.283  -1.035   3.455  0.00  0.00           N
ATOM     50  CA  GLU A  13       6.022  -2.054   2.446  0.00  0.00           C
ATOM     51  C   GLU A  13       7.321  -2.638   1.904  0.00  0.00           C
ATOM     52  O   GLU A  13       7.409  -3.836   1.636  0.00  0.00           O
ATOM     53  N   HIS A  14       8.325  -1.783   1.744 18.87  0.00           N
ATOM     54  CA  HIS A  14       9.631  -2.218   1.261 18.87  0.00           C
ATOM     55  C   HIS A  14      10.335  -3.092   2.292 18.87  0.00           C
ATOM     56  O   HIS A  14      11.251  -3.845   1.959 18.87  0.00           O
ATOM     57  N   LEU A  15       9.904  -2.987   3.543 29.94  0.00           N
ATOM     58  CA  LEU A  15      10.425  -3.837   4.608 29.94  0.00           C
ATOM     59  C   LEU A  15       9.356  -4.794   5.120 29.94  0.00           C
ATOM     60  O   LEU A  15       9.386  -5.210   6.278 29.94  0.00           O
ATOM     61  N   SER A  16       8.414  -5.141   4.251 29.27  0.00           N
ATOM     62  CA  SER A  16       7.306  -6.010   4.628 29.27  0.00           C
ATOM     63  C   SER A  16       7.798  -7.409   4.981 29.27  0.00           C
ATOM     64  O   SER A  16       7.141  -8.141   5.720 29.27  0.00           O
ATOM     65  N   HIS A  17       8.959  -7.773   4.447 26.74  0.00           N
ATOM     66  CA  HIS A  17       9.580  -9.053   4.765 26.74  0.00           C
ATOM     67  C   HIS A  17      10.221  -9.026   6.147 26.74  0.00           C
ATOM     68  O   HIS A  17      10.534 -10.071   6.718 26.74  0.00           O
ATOM     69  N   VAL A  18      10.414  -7.825   6.681 28.01  0.00           N
ATOM     70  CA  VAL A  18      10.985  -7.660   8.011 28.01  0.00           C
ATOM     71  C   VAL A  18       9.935  -7.876   9.093 28.01  0.00           C
ATOM     72  O   VAL A  18       8.851  -7.295   9.045 28.01  0.00           O
ATOM     73  N   LYS A  19      10.262  -8.717  10.069 25.89  0.00           N
ATOM     74  CA  LYS A  19       9.372  -8.966  11.196 25.89  0.00           C
ATOM     75  C   LYS A  19       8.921  -7.662  11.840 25.89  0.00           C
ATOM     76  O   LYS A  19       9.720  -6.747  12.038 25.89  0.00           O
ATOM     77  N   GLU A  20       7.634  -7.583  12.165 38.34  0.00           N
ATOM     78  CA  GLU A  20       7.052  -6.356  12.695 38.34  0.00           C
ATOM     79  C   GLU A  20       7.622  -6.023  14.067 38.34  0.00           C
ATOM     80  O   GLU A  20       7.384  -4.940  14.603 38.34  0.00           O
ATOM     81  N   GLU A  21       8.375  -6.959  14.633 52.27  0.00           N
ATOM     82  CA  GLU A  21       9.076  -6.724  15.889 52.27  0.00           C
ATOM     83  C   GLU A  21      10.514  -6.283  15.644 52.27  0.00           C
ATOM     84  O   GLU A  21      11.367  -6.397  16.525 52.27  0.00           O
ATOM     85  N   ASN A  22      10.776  -5.781  14.443 45.69  0.00           N
ATOM     86  CA  ASN A  22      12.078  -5.209  14.119 45.69  0.00           C
ATOM     87  C   ASN A  22      13.144  -6.292  14.015 45.69  0.00           C
ATOM     88  O   ASN A  22      14.261  -6.126  14.505 45.69  0.00           O
ATOM     89  N   THR A  23      12.793  -7.400  13.372 71.65  0.00           N
ATOM     90  CA  THR A  23      13.758  -8.453  13.077 71.65  0.00           C
ATOM     91  C   THR A  23      13.774  -8.786  11.591 71.65  0.00           C
ATOM     92  O   THR A  23      12.815  -9.347  11.061 71.65  0.00           O
ATOM     93  N   ILE A  24      14.867  -8.435  10.923 76.27  0.00           N
ATOM     94  CA  ILE A  24      14.963  -8.586   9.476 76.27  0.00           C
ATOM     95  C   ILE A  24      15.043 -10.056   9.080 76.27  0.00           C
ATOM     96  O   ILE A  24      15.957 -10.771   9.492 76.27  0.00           O
ATOM     97  N   TYR A  25      14.081 -10.500   8.279 61.46  0.00           N
ATOM     98  CA  TYR A  25      14.097 -11.856   7.742 61.46  0.00           C
ATOM     99  C   TYR A  25      14.610 -11.874   6.307 61.46  0.00           C
ATOM    100  O   TYR A  25      13.971 -11.337   5.403 61.46  0.00           O
ATOM    101  N   VAL A  26      15.768 -12.495   6.107 76.75  0.00           N
ATOM    102  CA  VAL A  26      16.377 -12.569   4.784 76.75  0.00           C
ATOM    103  C   VAL A  26      15.764 -13.690   3.955 76.75  0.00           C
ATOM    104  O   VAL A  26      15.330 -13.473   2.824 76.75  0.00           O
ATOM    105  N   THR A  27      15.730 -14.889   4.526 59.89  0.00           N
ATOM    106  CA  THR A  27      15.187 -16.052   3.833 59.89  0.00           C
ATOM    107  C   THR A  27      13.781 -15.777   3.313 59.89  0.00           C
ATOM    108  O   THR A  27      13.426 -16.193   2.211 59.89  0.00           O
ATOM    109  N   ASP A  28      12.987 -15.074   4.113 62.29  0.00           N
ATOM    110  CA  ASP A  28      11.622 -14.732   3.730 62.29  0.00           C
ATOM    111  C   ASP A  28      11.592 -14.007   2.390 62.29  0.00           C
ATOM    112  O   ASP A  28      10.728 -14.265   1.553 62.29  0.00           O
ATOM    113  N   LEU A  29      12.541 -13.097   2.195 78.11  0.00           N
ATOM    114  CA  LEU A  29      12.636 -12.345   0.949 78.11  0.00           C
ATOM    115  C   LEU A  29      13.227 -13.198  -0.165 78.11  0.00           C
ATOM    116  O   LEU A  29      12.760 -13.163  -1.303 78.11  0.00           O
ATOM    117  N   VAL A  30      14.260 -13.966   0.168 60.79  0.00           N
ATOM    118  CA  VAL A  30      14.974 -14.762  -0.823 60.79  0.00           C
ATOM    119  C   VAL A  30      14.049 -15.770  -1.491 60.79  0.00           C
ATOM    120  O   VAL A  30      14.140 -16.009  -2.696 60.79  0.00           O
ATOM    121  N   ARG A  31      13.156 -16.359  -0.702 99.99  0.00           N
ATOM    122  CA  ARG A  31      12.295 -17.431  -1.187 99.99  0.00           C
ATOM    123  C   ARG A  31      10.952 -16.890  -1.659 99.99  0.00           C
ATOM    124  O   ARG A  31      10.035 -17.653  -1.962 99.99  0.00           O
ATOM    125  N   CYS A  32      10.843 -15.567  -1.721 67.03  0.00           N
ATOM    126  CA  CYS A  32       9.624 -14.920  -2.191 67.03  0.00           C
ATOM    127  C   CYS A  32       9.536 -14.947  -3.711 67.03  0.00           C
ATOM    128  O   CYS A  32      10.473 -14.551  -4.404 67.03  0.00           O
ATOM    129  N   PRO A  33       8.405 -15.419  -4.224 50.80  0.00           N
ATOM    130  CA  PRO A  33       8.181 -15.473  -5.664 50.80  0.00           C
ATOM    131  C   PRO A  33       8.104 -14.074  -6.262 50.80  0.00           C
ATOM    132  O   PRO A  33       8.375 -13.880  -7.447 50.80  0.00           O
```

```
ATOM    133  N   ARG A  34       7.735 -13.102  -5.436 19.13  0.00           N
ATOM    134  CA  ARG A  34       7.681 -11.710  -5.864 19.13  0.00           C
ATOM    135  C   ARG A  34       9.077 -11.160  -6.129 19.13  0.00           C
ATOM    136  O   ARG A  34       9.292 -10.418  -7.087 19.13  0.00           O
ATOM    137  N   ARG A  35      10.024 -11.530  -5.274 22.35  0.00           N
ATOM    138  CA  ARG A  35      11.430 -11.223  -5.507 22.35  0.00           C
ATOM    139  C   ARG A  35      11.911 -11.815  -6.826 22.35  0.00           C
ATOM    140  O   ARG A  35      12.628 -11.163  -7.586 22.35  0.00           O
ATOM    141  N   VAL A  36      11.514 -13.054  -7.092 50.49  0.00           N
ATOM    142  CA  VAL A  36      11.834 -13.707  -8.356 50.49  0.00           C
ATOM    143  C   VAL A  36      11.285 -12.918  -9.538 50.49  0.00           C
ATOM    144  O   VAL A  36      11.943 -12.790 -10.571 50.49  0.00           O
ATOM    145  N   ARG A  37      10.075 -12.393  -9.381 50.75  0.00           N
ATOM    146  CA  ARG A  37       9.466 -11.553 -10.406 50.75  0.00           C
ATOM    147  C   ARG A  37      10.294 -10.299 -10.654 50.75  0.00           C
ATOM    148  O   ARG A  37      10.454  -9.865 -11.796 50.75  0.00           O
ATOM    149  N   TYR A  38      10.819  -9.720  -9.579 51.51  0.00           N
ATOM    150  CA  TYR A  38      11.687  -8.553  -9.685 51.51  0.00           C
ATOM    151  C   TYR A  38      12.991  -8.898 -10.392 51.51  0.00           C
ATOM    152  O   TYR A  38      13.480  -8.133 -11.224 51.51  0.00           O
ATOM    153  N   GLU A  39      13.552 -10.055 -10.056 73.67  0.00           N
ATOM    154  CA  GLU A  39      14.832 -10.477 -10.613 73.67  0.00           C
ATOM    155  C   GLU A  39      14.724 -10.723 -12.113 73.67  0.00           C
ATOM    156  O   GLU A  39      15.615 -10.352 -12.878 73.67  0.00           O
ATOM    157  N   SER A  40      13.628 -11.348 -12.528 55.15  0.00           N
ATOM    158  CA  SER A  40      13.440 -11.719 -13.927 55.15  0.00           C
ATOM    159  C   SER A  40      13.018 -10.518 -14.762 55.15  0.00           C
ATOM    160  O   SER A  40      13.599 -10.245 -15.812 55.15  0.00           O
ATOM    161  N   GLU A  41      12.002  -9.802 -14.290 47.58  0.00           N
ATOM    162  CA  GLU A  41      11.468  -8.656 -15.017 47.58  0.00           C
ATOM    163  C   GLU A  41      12.462  -7.503 -15.036 47.58  0.00           C
ATOM    164  O   GLU A  41      12.611  -6.814 -16.046 47.58  0.00           O
ATOM    165  N   TYR A  42      13.143  -7.297 -13.913 46.48  0.00           N
ATOM    166  CA  TYR A  42      14.100  -6.205 -13.786 46.48  0.00           C
ATOM    167  C   TYR A  42      15.492  -6.728 -13.459 46.48  0.00           C
ATOM    168  O   TYR A  42      15.936  -6.666 -12.312 46.48  0.00           O
ATOM    169  N   LYS A  43      16.180  -7.243 -14.474 61.04  0.00           N
ATOM    170  CA  LYS A  43      17.492  -7.848 -14.281 61.04  0.00           C
ATOM    171  C   LYS A  43      18.500  -6.827 -13.769 61.04  0.00           C
ATOM    172  O   LYS A  43      19.471  -7.180 -13.100 61.04  0.00           O
ATOM    173  N   GLU A  44      18.263  -5.558 -14.087 57.96  0.00           N
ATOM    174  CA  GLU A  44      19.156  -4.485 -13.670 57.96  0.00           C
ATOM    175  C   GLU A  44      18.840  -4.021 -12.254 57.96  0.00           C
ATOM    176  O   GLU A  44      19.630  -3.315 -11.630 57.96  0.00           O
ATOM    177  N   LEU A  45      17.677  -4.425 -11.752 52.74  0.00           N
ATOM    178  CA  LEU A  45      17.225  -4.003 -10.431 52.74  0.00           C
ATOM    179  C   LEU A  45      17.990  -4.726  -9.330 52.74  0.00           C
ATOM    180  O   LEU A  45      17.980  -5.955  -9.258 52.74  0.00           O
ATOM    181  N   ALA A  46      18.654  -3.956  -8.475 72.00  0.00           N
ATOM    182  CA  ALA A  46      19.441  -4.523  -7.386 72.00  0.00           C
ATOM    183  C   ALA A  46      18.543  -5.156  -6.330 72.00  0.00           C
ATOM    184  O   ALA A  46      17.481  -4.625  -6.005 72.00  0.00           O
ATOM    185  N   ILE A  47      18.976  -6.294  -5.798 59.42  0.00           N
ATOM    186  CA  ILE A  47      18.234  -6.980  -4.747 59.42  0.00           C
ATOM    187  C   ILE A  47      19.088  -7.165  -3.501 59.42  0.00           C
ATOM    188  O   ILE A  47      20.067  -7.911  -3.511 59.42  0.00           O
ATOM    189  N   SER A  48      18.712  -6.482  -2.425 66.56  0.00           N
ATOM    190  CA  SER A  48      19.439  -6.576  -1.164 66.56  0.00           C
ATOM    191  C   SER A  48      18.808  -7.610  -0.241 66.56  0.00           C
ATOM    192  O   SER A  48      17.709  -8.102  -0.502 66.56  0.00           O
ATOM    193  N   GLN A  49      19.508  -7.937   0.839 62.34  0.00           N
ATOM    194  CA  GLN A  49      18.961  -8.810   1.873 62.34  0.00           C
ATOM    195  C   GLN A  49      18.290  -8.003   2.977 62.34  0.00           C
ATOM    196  O   GLN A  49      17.410  -8.504   3.676 62.34  0.00           O
ATOM    197  N   VAL A  50      18.713  -6.753   3.129 62.33  0.00           N
ATOM    198  CA  VAL A  50      18.189  -5.890   4.182 62.33  0.00           C
ATOM    199  C   VAL A  50      16.787  -5.400   3.846 62.33  0.00           C
ATOM    200  O   VAL A  50      15.921  -5.323   4.716 62.33  0.00           O
ATOM    201  N   TYR A  51      16.569  -5.070   2.577 23.81  0.00           N
ATOM    202  CA  TYR A  51      15.303  -4.493   2.142 23.81  0.00           C
ATOM    203  C   TYR A  51      14.898  -5.024   0.773 23.81  0.00           C
ATOM    204  O   TYR A  51      15.735  -5.510   0.012 23.81  0.00           O
ATOM    205  N   ALA A  52      13.609  -4.928   0.464 43.17  0.00           N
ATOM    206  CA  ALA A  52      13.081  -5.444  -0.793 43.17  0.00           C
ATOM    207  C   ALA A  52      13.537  -4.592  -1.971 43.17  0.00           C
ATOM    208  O   ALA A  52      13.737  -3.385  -1.837 43.17  0.00           O
ATOM    209  N   PRO A  53      13.701  -5.229  -3.126 43.74  0.00           N
ATOM    210  CA  PRO A  53      14.125  -4.529  -4.334 43.74  0.00           C
ATOM    211  C   PRO A  53      13.085  -3.506  -4.771 43.74  0.00           C
ATOM    212  O   PRO A  53      13.374  -2.616  -5.572 43.74  0.00           O
ATOM    213  N   SER A  54      11.873  -3.637  -4.242 38.09  0.00           N
ATOM    214  CA  SER A  54      10.808  -2.681  -4.520 38.09  0.00           C
ATOM    215  C   SER A  54      11.169  -1.291  -4.008 38.09  0.00           C
ATOM    216  O   SER A  54      10.608  -0.290  -4.456 38.09  0.00           O
ATOM    217  N   ALA A  55      12.108  -1.237  -3.071 38.71  0.00           N
ATOM    218  CA  ALA A  55      12.633   0.034  -2.585 38.71  0.00           C
ATOM    219  C   ALA A  55      13.374   0.781  -3.686 38.71  0.00           C
ATOM    220  O   ALA A  55      13.191   1.985  -3.865 38.71  0.00           O
ATOM    221  N   ILE A  56      14.213   0.059  -4.423 47.01  0.00           N
ATOM    222  CA  ILE A  56      15.015   0.660  -5.481 47.01  0.00           C
ATOM    223  C   ILE A  56      14.155   1.024  -6.684 47.01  0.00           C
ATOM    224  O   ILE A  56      14.272   2.119  -7.235 47.01  0.00           O
```

```
ATOM    225  N   LEU A  57      13.290   0.101  -7.088 30.78  0.00           N
ATOM    226  CA  LEU A  57      12.359   0.348  -8.182 30.78  0.00           C
ATOM    227  C   LEU A  57      11.362   1.440  -7.821 30.78  0.00           C
ATOM    228  O   LEU A  57      11.021   2.284  -8.651 30.78  0.00           O
ATOM    229  N   GLY A  58      10.896   1.422  -6.577 41.70  0.00           N
ATOM    230  CA  GLY A  58       9.983   2.446  -6.084 41.70  0.00           C
ATOM    231  C   GLY A  58      10.639   3.820  -6.096 41.70  0.00           C
ATOM    232  O   GLY A  58      10.012   4.815  -6.462 41.70  0.00           O
ATOM    233  N   ASP A  59      11.904   3.870  -5.694 47.60  0.00           N
ATOM    234  CA  ASP A  59      12.679   5.103  -5.756 47.60  0.00           C
ATOM    235  C   ASP A  59      12.720   5.659  -7.173 47.60  0.00           C
ATOM    236  O   ASP A  59      12.530   6.857  -7.387 47.60  0.00           O
ATOM    237  N   ILE A  60      12.967   4.781  -8.140 48.47  0.00           N
ATOM    238  CA  ILE A  60      12.964   5.169  -9.546 48.47  0.00           C
ATOM    239  C   ILE A  60      11.595   5.684  -9.973 48.47  0.00           C
ATOM    240  O   ILE A  60      11.490   6.702 -10.656 48.47  0.00           O
ATOM    241  N   LEU A  61      10.548   4.976  -9.564 35.67  0.00           N
ATOM    242  CA  LEU A  61       9.184   5.356  -9.909 35.67  0.00           C
ATOM    243  C   LEU A  61       8.801   6.681  -9.263 35.67  0.00           C
ATOM    244  O   LEU A  61       8.051   7.470  -9.839 35.67  0.00           O
ATOM    245  N   HIS A  62       9.322   6.922  -8.064 37.96  0.00           N
ATOM    246  CA  HIS A  62       9.016   8.141  -7.326 37.96  0.00           C
ATOM    247  C   HIS A  62       9.746   9.340  -7.919 37.96  0.00           C
ATOM    248  O   HIS A  62       9.257  10.469  -7.858 37.96  0.00           O
ATOM    249  N   LEU A  63      10.919   9.090  -8.491 34.78  0.00           N
ATOM    250  CA  LEU A  63      11.650  10.117  -9.221 34.78  0.00           C
ATOM    251  C   LEU A  63      10.859  10.603 -10.429 34.78  0.00           C
ATOM    252  O   LEU A  63      10.672  11.804 -10.618 34.78  0.00           O
ATOM    253  N   GLY A  64      10.396   9.661 -11.244 36.31  0.00           N
ATOM    254  CA  GLY A  64       9.577   9.989 -12.405 36.31  0.00           C
ATOM    255  C   GLY A  64       8.251  10.611 -11.988 36.31  0.00           C
ATOM    256  O   GLY A  64       7.772  11.556 -12.616 36.31  0.00           O
ATOM    257  N   LEU A  65       7.661  10.076 -10.924 17.94  0.00           N
ATOM    258  CA  LEU A  65       6.412  10.608 -10.393 17.94  0.00           C
ATOM    259  C   LEU A  65       6.561  12.070  -9.994 17.94  0.00           C
ATOM    260  O   LEU A  65       5.716  12.903 -10.321 17.94  0.00           O
ATOM    261  N   GLU A  66       7.642  12.377  -9.284 54.01  0.00           N
ATOM    262  CA  GLU A  66       7.918  13.745  -8.861 54.01  0.00           C
ATOM    263  C   GLU A  66       8.073  14.671 -10.061 54.01  0.00           C
ATOM    264  O   GLU A  66       7.549  15.786 -10.068 54.01  0.00           O
ATOM    265  N   SER A  67       8.794  14.204 -11.075 46.40  0.00           N
ATOM    266  CA  SER A  67       9.001  14.982 -12.290 46.40  0.00           C
ATOM    267  C   SER A  67       7.675  15.334 -12.952 46.40  0.00           C
ATOM    268  O   SER A  67       7.480  16.458 -13.413 46.40  0.00           O
ATOM    269  N   VAL A  68       6.766  14.366 -12.993 51.69  0.00           N
ATOM    270  CA  VAL A  68       5.454  14.573 -13.595 51.69  0.00           C
ATOM    271  C   VAL A  68       4.602  15.514 -12.751 51.69  0.00           C
ATOM    272  O   VAL A  68       3.969  16.431 -13.273 51.69  0.00           O
ATOM    273  N   LEU A  69       4.591  15.278 -11.443 50.04  0.00           N
ATOM    274  CA  LEU A  69       3.755  16.053 -10.533 50.04  0.00           C
ATOM    275  C   LEU A  69       4.206  17.507 -10.474 50.04  0.00           C
ATOM    276  O   LEU A  69       3.386  18.416 -10.348 50.04  0.00           O
ATOM    277  N   LYS A  70       5.514  17.719 -10.566 52.71  0.00           N
ATOM    278  CA  LYS A  70       6.078  19.062 -10.508 52.71  0.00           C
ATOM    279  C   LYS A  70       5.368  20.001 -11.475 52.71  0.00           C
ATOM    280  O   LYS A  70       5.007  21.121 -11.115 52.71  0.00           O
ATOM    281  N   GLY A  71       5.171  19.537 -12.704 49.67  0.00           N
ATOM    282  CA  GLY A  71       4.479  20.323 -13.717 49.67  0.00           C
ATOM    283  C   GLY A  71       2.967  20.200 -13.575 49.67  0.00           C
ATOM    284  O   GLY A  71       2.237  21.179 -13.734 49.67  0.00           O
ATOM    285  N   ASN A  72       2.503  18.992 -13.272 49.24  0.00           N
ATOM    286  CA  ASN A  72       1.073  18.722 -13.183 49.24  0.00           C
ATOM    287  C   ASN A  72       0.749  17.874 -11.960 49.24  0.00           C
ATOM    288  O   ASN A  72       0.626  16.653 -12.054 49.24  0.00           O
ATOM    289  N   PHE A  73       0.609  18.529 -10.812 43.22  0.00           N
ATOM    290  CA  PHE A  73       0.374  17.830  -9.555 43.22  0.00           C
ATOM    291  C   PHE A  73      -0.940  17.060  -9.591 43.22  0.00           C
ATOM    292  O   PHE A  73      -1.070  16.005  -8.970 43.22  0.00           O
ATOM    293  N   ASN A  74      -1.914  17.595 -10.320 60.11  0.00           N
ATOM    294  CA  ASN A  74      -3.255  17.022 -10.346 60.11  0.00           C
ATOM    295  C   ASN A  74      -3.379  15.958 -11.427 60.11  0.00           C
ATOM    296  O   ASN A  74      -4.459  15.410 -11.652 60.11  0.00           O
ATOM    297  N   ALA A  75      -2.268  15.668 -12.096 54.45  0.00           N
ATOM    298  CA  ALA A  75      -2.230  14.607 -13.095 54.45  0.00           C
ATOM    299  C   ALA A  75      -2.141  13.234 -12.441 54.45  0.00           C
ATOM    300  O   ALA A  75      -1.349  13.025 -11.522 54.45  0.00           O
ATOM    301  N   GLU A  76      -2.959  12.303 -12.919 56.74  0.00           N
ATOM    302  CA  GLU A  76      -2.994  10.957 -12.361 56.74  0.00           C
ATOM    303  C   GLU A  76      -1.989  10.046 -13.054 56.74  0.00           C
ATOM    304  O   GLU A  76      -2.204   9.619 -14.188 56.74  0.00           O
ATOM    305  N   THR A  77      -0.890   9.753 -12.366 71.61  0.00           N
ATOM    306  CA  THR A  77       0.103   8.814 -12.872 71.61  0.00           C
ATOM    307  C   THR A  77      -0.322   7.374 -12.622 71.61  0.00           C
ATOM    308  O   THR A  77      -0.623   6.992 -11.491 71.61  0.00           O
ATOM    309  N   GLU A  78      -0.346   6.576 -13.684 74.39  0.00           N
ATOM    310  CA  GLU A  78      -0.862   5.214 -13.608 74.39  0.00           C
ATOM    311  C   GLU A  78       0.262   4.192 -13.736 74.39  0.00           C
ATOM    312  O   GLU A  78       0.636   3.802 -14.842 74.39  0.00           O
ATOM    313  N   VAL A  79       0.795   3.762 -12.598 55.67  0.00           N
ATOM    314  CA  VAL A  79       1.814   2.719 -12.575 55.67  0.00           C
ATOM    315  C   VAL A  79       1.203   1.358 -12.266 55.67  0.00           C
ATOM    316  O   VAL A  79       0.630   1.154 -11.196 55.67  0.00           O
```

```
ATOM    317  N   GLU A  80       1.329   0.431 -13.209 51.09  0.00           N
ATOM    318  CA  GLU A  80       0.790  -0.914 -13.037 51.09  0.00           C
ATOM    319  C   GLU A  80       1.486  -1.644 -11.896 51.09  0.00           C
ATOM    320  O   GLU A  80       2.713  -1.718 -11.849 51.09  0.00           O
ATOM    321  N   THR A  81       0.694  -2.182 -10.974 43.26  0.00           N
ATOM    322  CA  THR A  81       1.222  -3.007  -9.895 43.26  0.00           C
ATOM    323  C   THR A  81       0.981  -4.487 -10.163 43.26  0.00           C
ATOM    324  O   THR A  81      -0.153  -4.910 -10.391 43.26  0.00           O
ATOM    325  N   LEU A  82       2.053  -5.272 -10.136 50.29  0.00           N
ATOM    326  CA  LEU A  82       1.955  -6.711 -10.344 50.29  0.00           C
ATOM    327  C   LEU A  82       2.526  -7.479  -9.160 50.29  0.00           C
ATOM    328  O   LEU A  82       3.699  -7.331  -8.819 50.29  0.00           O
ATOM    329  N   ARG A  83       1.690  -8.301  -8.535 18.69  0.00           N
ATOM    330  CA  ARG A  83       2.072  -9.000  -7.314 18.69  0.00           C
ATOM    331  C   ARG A  83       1.652 -10.464  -7.364 18.69  0.00           C
ATOM    332  O   ARG A  83       0.495 -10.779  -7.642 18.69  0.00           O
ATOM    333  N   GLU A  84       2.599 -11.355  -7.092 14.28  0.00           N
ATOM    334  CA  GLU A  84       2.286 -12.761  -6.860 14.28  0.00           C
ATOM    335  C   GLU A  84       1.899 -13.006  -5.407 14.28  0.00           C
ATOM    336  O   GLU A  84       2.754 -13.022  -4.521 14.28  0.00           O
ATOM    337  N   ILE A  85       0.607 -13.196  -5.169 56.26  0.00           N
ATOM    338  CA  ILE A  85       0.073 -13.226  -3.812 56.26  0.00           C
ATOM    339  C   ILE A  85      -0.412 -14.621  -3.441 56.26  0.00           C
ATOM    340  O   ILE A  85      -1.249 -15.202  -4.133 56.26  0.00           O
ATOM    341  N   ASN A  86       0.117 -15.155  -2.346 40.06  0.00           N
ATOM    342  CA  ASN A  86      -0.292 -16.467  -1.859 40.06  0.00           C
ATOM    343  C   ASN A  86      -1.647 -16.401  -1.167 40.06  0.00           C
ATOM    344  O   ASN A  86      -1.775 -15.830  -0.085 40.06  0.00           O
ATOM    345  N   VAL A  87      -2.657 -16.989  -1.800 50.74  0.00           N
ATOM    346  CA  VAL A  87      -4.005 -17.004  -1.244 50.74  0.00           C
ATOM    347  C   VAL A  87      -4.441 -18.421  -0.894 50.74  0.00           C
ATOM    348  O   VAL A  87      -4.785 -19.210  -1.776 50.74  0.00           O
ATOM    349  N   GLY A  88      -4.426 -18.739   0.395 49.52  0.00           N
ATOM    350  CA  GLY A  88      -4.904 -20.031   0.872 49.52  0.00           C
ATOM    351  C   GLY A  88      -3.945 -21.150   0.486 49.52  0.00           C
ATOM    352  O   GLY A  88      -4.337 -22.314   0.401 49.52  0.00           O
ATOM    353  N   GLY A  89      -2.687 -20.790   0.255 48.06  0.00           N
ATOM    354  CA  GLY A  89      -1.673 -21.761  -0.137 48.06  0.00           C
ATOM    355  C   GLY A  89      -1.499 -21.798  -1.650 48.06  0.00           C
ATOM    356  O   GLY A  89      -0.654 -22.525  -2.170 48.06  0.00           O
ATOM    357  N   LYS A  90      -2.305 -21.008  -2.352 49.69  0.00           N
ATOM    358  CA  LYS A  90      -2.242 -20.947  -3.807 49.69  0.00           C
ATOM    359  C   LYS A  90      -1.870 -19.550  -4.284 49.69  0.00           C
ATOM    360  O   LYS A  90      -2.650 -18.608  -4.146 49.69  0.00           O
ATOM    361  N   VAL A  91      -0.673 -19.420  -4.847 50.28  0.00           N
ATOM    362  CA  VAL A  91      -0.190 -18.135  -5.335 50.28  0.00           C
ATOM    363  C   VAL A  91      -0.874 -17.748  -6.640 50.28  0.00           C
ATOM    364  O   VAL A  91      -0.806 -18.479  -7.628 50.28  0.00           O
ATOM    365  N   TYR A  92      -1.535 -16.595  -6.638 61.33  0.00           N
ATOM    366  CA  TYR A  92      -2.205 -16.093  -7.831 61.33  0.00           C
ATOM    367  C   TYR A  92      -1.548 -14.814  -8.335 61.33  0.00           C
ATOM    368  O   TYR A  92      -1.100 -13.983  -7.546 61.33  0.00           O
ATOM    369  N   LYS A  93      -1.495 -14.662  -9.654 75.56  0.00           N
ATOM    370  CA  LYS A  93      -1.036 -13.421 -10.264 75.56  0.00           C
ATOM    371  C   LYS A  93      -2.099 -12.336 -10.173 75.56  0.00           C
ATOM    372  O   LYS A  93      -3.121 -12.393 -10.858 75.56  0.00           O
ATOM    373  N   ILE A  94      -1.854 -11.344  -9.322 54.38  0.00           N
ATOM    374  CA  ILE A  94      -2.834 -10.295  -9.066 54.38  0.00           C
ATOM    375  C   ILE A  94      -2.268  -8.920  -9.396 54.38  0.00           C
ATOM    376  O   ILE A  94      -1.149  -8.589  -9.005 54.38  0.00           O
ATOM    377  N   LYS A  95      -3.049  -8.123 -10.117 65.40  0.00           N
ATOM    378  CA  LYS A  95      -2.610  -6.796 -10.536 65.40  0.00           C
ATOM    379  C   LYS A  95      -3.477  -5.707  -9.915 65.40  0.00           C
ATOM    380  O   LYS A  95      -4.426  -5.997  -9.187 65.40  0.00           O
ATOM    381  N   GLY A  96      -3.145  -4.456 -10.208 50.04  0.00           N
ATOM    382  CA  GLY A  96      -4.026  -3.337  -9.899 50.04  0.00           C
ATOM    383  C   GLY A  96      -3.231  -2.062  -9.647 50.04  0.00           C
ATOM    384  O   GLY A  96      -2.071  -1.954 -10.043 50.04  0.00           O
ATOM    385  N   ARG A  97      -3.863  -1.098  -8.984 43.71  0.00           N
ATOM    386  CA  ARG A  97      -3.202   0.156  -8.642 43.71  0.00           C
ATOM    387  C   ARG A  97      -4.031   0.963  -7.652 43.71  0.00           C
ATOM    388  O   ARG A  97      -5.201   0.661  -7.415 43.71  0.00           O
ATOM    389  N   ALA A  98      -3.419   1.990  -7.074 45.35  0.00           N
ATOM    390  CA  ALA A  98      -4.127   2.902  -6.183 45.35  0.00           C
ATOM    391  C   ALA A  98      -3.531   4.303  -6.240 45.35  0.00           C
ATOM    392  O   ALA A  98      -2.354   4.475  -6.557 45.35  0.00           O
ATOM    393  N   ASP A  99      -4.350   5.302  -5.932 52.70  0.00           N
ATOM    394  CA  ASP A  99      -3.910   6.692  -5.966 52.70  0.00           C
ATOM    395  C   ASP A  99      -4.496   7.484  -4.803 52.70  0.00           C
ATOM    396  O   ASP A  99      -5.699   7.430  -4.547 52.70  0.00           O
ATOM    397  N   ALA A 100      -3.638   8.219  -4.103 56.40  0.00           N
ATOM    398  CA  ALA A 100      -4.090   9.164  -3.088 56.40  0.00           C
ATOM    399  C   ALA A 100      -4.265  10.559  -3.674 56.40  0.00           C
ATOM    400  O   ALA A 100      -3.508  10.974  -4.551 56.40  0.00           O
ATOM    401  N   ILE A 101      -5.269  11.279  -3.183 52.13  0.00           N
ATOM    402  CA  ILE A 101      -5.554  12.624  -3.669 52.13  0.00           C
ATOM    403  C   ILE A 101      -5.525  13.637  -2.532 52.13  0.00           C
ATOM    404  O   ILE A 101      -6.465  13.727  -1.742 52.13  0.00           O
ATOM    405  N   ILE A 102      -4.440  14.400  -2.453 51.64  0.00           N
ATOM    406  CA  ILE A 102      -4.299  15.429  -1.431 51.64  0.00           C
ATOM    407  C   ILE A 102      -4.842  16.767  -1.917 51.64  0.00           C
ATOM    408  O   ILE A 102      -4.306  17.362  -2.854 51.64  0.00           O
```

ATOM	409	N	ARG	A	103	-5.906	17.237	-1.277	49.29	0.00	N
ATOM	410	CA	ARG	A	103	-6.489	18.532	-1.604	49.29	0.00	C
ATOM	411	C	ARG	A	103	-6.093	19.589	-0.580	49.29	0.00	C
ATOM	412	O	ARG	A	103	-6.312	19.418	0.618	49.29	0.00	O
ATOM	413	N	ASN	A	104	-5.508	20.681	-1.061	45.99	0.00	N
ATOM	414	CA	ASN	A	104	-5.132	21.792	-0.195	45.99	0.00	C
ATOM	415	C	ASN	A	104	-6.217	22.861	-0.169	45.99	0.00	C
ATOM	416	O	ASN	A	104	-7.085	22.901	-1.041	45.99	0.00	O
ATOM	417	N	ASP	A	105	-6.164	23.727	0.838	43.08	0.00	N
ATOM	418	CA	ASP	A	105	-7.141	24.799	0.980	43.08	0.00	C
ATOM	419	C	ASP	A	105	-7.041	25.791	-0.172	43.08	0.00	C
ATOM	420	O	ASP	A	105	-8.000	26.499	-0.479	43.08	0.00	O
ATOM	421	N	ASN	A	106	-5.874	25.838	-0.805	50.08	0.00	N
ATOM	422	CA	ASN	A	106	-5.655	26.724	-1.942	50.08	0.00	C
ATOM	423	C	ASN	A	106	-6.260	26.145	-3.216	50.08	0.00	C
ATOM	424	O	ASN	A	106	-6.341	26.823	-4.240	50.08	0.00	O
ATOM	425	N	GLY	A	107	-6.684	24.887	-3.144	44.36	0.00	N
ATOM	426	CA	GLY	A	107	-7.288	24.217	-4.289	44.36	0.00	C
ATOM	427	C	GLY	A	107	-6.264	23.368	-5.033	44.36	0.00	C
ATOM	428	O	GLY	A	107	-6.555	22.812	-6.091	44.36	0.00	O
ATOM	429	N	LYS	A	108	-5.062	23.274	-4.472	50.81	0.00	N
ATOM	430	CA	LYS	A	108	-3.996	22.484	-5.075	50.81	0.00	C
ATOM	431	C	LYS	A	108	-4.222	20.993	-4.855	50.81	0.00	C
ATOM	432	O	LYS	A	108	-4.259	20.522	-3.717	50.81	0.00	O
ATOM	433	N	SER	A	109	-4.374	20.255	-5.948	55.18	0.00	N
ATOM	434	CA	SER	A	109	-4.595	18.815	-5.877	55.18	0.00	C
ATOM	435	C	SER	A	109	-3.332	18.046	-6.244	55.18	0.00	C
ATOM	436	O	SER	A	109	-2.792	18.205	-7.338	55.18	0.00	O
ATOM	437	N	ILE	A	110	-2.865	17.212	-5.321	54.55	0.00	N
ATOM	438	CA	ILE	A	110	-1.674	16.403	-5.552	54.55	0.00	C
ATOM	439	C	ILE	A	110	-2.012	14.917	-5.564	54.55	0.00	C
ATOM	440	O	ILE	A	110	-2.475	14.368	-4.564	54.55	0.00	O
ATOM	441	N	VAL	A	111	-1.776	14.271	-6.701	56.94	0.00	N
ATOM	442	CA	VAL	A	111	-2.064	12.849	-6.847	56.94	0.00	C
ATOM	443	C	VAL	A	111	-0.816	12.007	-6.620	56.94	0.00	C
ATOM	444	O	VAL	A	111	0.221	12.237	-7.242	56.94	0.00	O
ATOM	445	N	ILE	A	112	-0.922	11.032	-5.725	55.75	0.00	N
ATOM	446	CA	ILE	A	112	0.212	10.183	-5.379	55.75	0.00	C
ATOM	447	C	ILE	A	112	-0.040	8.735	-5.782	55.75	0.00	C
ATOM	448	O	ILE	A	112	-0.946	8.085	-5.262	55.75	0.00	O
ATOM	449	N	GLU	A	113	0.766	8.237	-6.713	47.46	0.00	N
ATOM	450	CA	GLU	A	113	0.744	6.822	-7.069	47.46	0.00	C
ATOM	451	C	GLU	A	113	1.080	5.947	-5.868	47.46	0.00	C
ATOM	452	O	GLU	A	113	2.105	6.141	-5.214	47.46	0.00	O
ATOM	453	N	ILE	A	114	0.211	4.983	-5.584	50.04	0.00	N
ATOM	454	CA	ILE	A	114	0.447	4.035	-4.502	50.04	0.00	C
ATOM	455	C	ILE	A	114	0.756	2.645	-5.044	50.04	0.00	C
ATOM	456	O	ILE	A	114	-0.012	2.092	-5.832	50.04	0.00	O
ATOM	457	N	LYS	A	115	1.883	2.086	-4.619	48.70	0.00	N
ATOM	458	CA	LYS	A	115	2.279	0.747	-5.035	48.70	0.00	C
ATOM	459	C	LYS	A	115	2.822	-0.058	-3.861	48.70	0.00	C
ATOM	460	O	LYS	A	115	3.929	0.193	-3.384	48.70	0.00	O
ATOM	461	N	THR	A	116	2.038	-1.025	-3.398	39.30	0.00	N
ATOM	462	CA	THR	A	116	2.376	-1.777	-2.195	39.30	0.00	C
ATOM	463	C	THR	A	116	3.144	-3.047	-2.537	39.30	0.00	C
ATOM	464	O	THR	A	116	3.309	-3.388	-3.710	39.30	0.00	O
ATOM	465	N	SER	A	117	3.613	-3.745	-1.508	35.66	0.00	N
ATOM	466	CA	SER	A	117	4.461	-4.914	-1.698	35.66	0.00	C
ATOM	467	C	SER	A	117	3.656	-6.203	-1.590	35.66	0.00	C
ATOM	468	O	SER	A	117	4.023	-7.226	-2.167	35.66	0.00	O
ATOM	469	N	ARG	A	118	2.556	-6.146	-0.846	2.77	0.00	N
ATOM	470	CA	ARG	A	118	1.725	-7.322	-0.616	2.77	0.00	C
ATOM	471	C	ARG	A	118	0.339	-6.929	-0.123	2.77	0.00	C
ATOM	472	O	ARG	A	118	0.135	-5.816	0.363	2.77	0.00	O
ATOM	473	N	SER	A	119	-0.612	-7.848	-0.250	43.98	0.00	N
ATOM	474	CA	SER	A	119	-1.941	-7.660	0.320	43.98	0.00	C
ATOM	475	C	SER	A	119	-2.739	-8.957	0.293	43.98	0.00	C
ATOM	476	O	SER	A	119	-2.706	-9.698	-0.689	43.98	0.00	O
ATOM	477	N	ASP	A	120	-3.459	-9.225	1.377	48.09	0.00	N
ATOM	478	CA	ASP	A	120	-4.126	-10.509	1.555	48.09	0.00	C
ATOM	479	C	ASP	A	120	-5.511	-10.503	0.921	48.09	0.00	C
ATOM	480	O	ASP	A	120	-6.459	-9.957	1.483	48.09	0.00	O
ATOM	481	N	LYS	A	121	-5.620	-11.114	-0.255	17.69	0.00	N
ATOM	482	CA	LYS	A	121	-6.899	-11.220	-0.946	17.69	0.00	C
ATOM	483	C	LYS	A	121	-7.938	-11.918	-0.079	17.69	0.00	C
ATOM	484	O	LYS	A	121	-9.135	-11.654	-0.193	17.69	0.00	O
ATOM	485	N	GLY	A	122	-7.474	-12.812	0.788	51.98	0.00	N
ATOM	486	CA	GLY	A	122	-8.368	-13.672	1.553	51.98	0.00	C
ATOM	487	C	GLY	A	122	-9.190	-12.865	2.549	51.98	0.00	C
ATOM	488	O	GLY	A	122	-10.196	-13.345	3.073	51.98	0.00	O
ATOM	489	N	LEU	A	123	-8.757	-11.636	2.808	66.67	0.00	N
ATOM	490	CA	LEU	A	123	-9.459	-10.756	3.735	66.67	0.00	C
ATOM	491	C	LEU	A	123	-10.801	-10.315	3.167	66.67	0.00	C
ATOM	492	O	LEU	A	123	-10.998	-10.305	1.952	66.67	0.00	O
ATOM	493	N	PRO	A	124	-11.722	-9.952	4.053	59.92	0.00	N
ATOM	494	CA	PRO	A	124	-13.007	-9.398	3.639	59.92	0.00	C
ATOM	495	C	PRO	A	124	-12.819	-8.212	2.703	59.92	0.00	C
ATOM	496	O	PRO	A	124	-11.840	-7.474	2.808	59.92	0.00	O
ATOM	497	N	LEU	A	125	-13.764	-8.034	1.785	44.28	0.00	N
ATOM	498	CA	LEU	A	125	-13.659	-6.994	0.769	44.28	0.00	C
ATOM	499	C	LEU	A	125	-13.431	-5.627	1.403	44.28	0.00	C
ATOM	500	O	LEU	A	125	-12.870	-4.727	0.776	44.28	0.00	O

```
ATOM    501  N    ILE A 126     -13.870  -5.477   2.648 45.33  0.00           N
ATOM    502  CA   ILE A 126     -13.682  -4.230   3.381 45.33  0.00           C
ATOM    503  C    ILE A 126     -12.224  -3.793   3.361 45.33  0.00           C
ATOM    504  O    ILE A 126     -11.924  -2.601   3.303 45.33  0.00           O
ATOM    505  N    HIS A 127     -11.319  -4.766   3.410 35.63  0.00           N
ATOM    506  CA   HIS A 127      -9.901  -4.484   3.600 35.63  0.00           C
ATOM    507  C    HIS A 127      -9.333  -3.700   2.423 35.63  0.00           C
ATOM    508  O    HIS A 127      -8.273  -3.083   2.530 35.63  0.00           O
ATOM    509  N    HIS A 128     -10.043  -3.730   1.301 39.03  0.00           N
ATOM    510  CA   HIS A 128      -9.643  -2.971   0.122 39.03  0.00           C
ATOM    511  C    HIS A 128      -9.780  -1.473   0.358 39.03  0.00           C
ATOM    512  O    HIS A 128      -8.834  -0.713   0.149 39.03  0.00           O
ATOM    513  N    LYS A 129     -10.963  -1.053   0.796 61.21  0.00           N
ATOM    514  CA   LYS A 129     -11.218   0.353   1.081 61.21  0.00           C
ATOM    515  C    LYS A 129     -10.592   0.770   2.406 61.21  0.00           C
ATOM    516  O    LYS A 129     -10.298   1.945   2.623 61.21  0.00           O
ATOM    517  N    MET A 130     -10.391  -0.201   3.291 46.12  0.00           N
ATOM    518  CA   MET A 130      -9.710   0.044   4.556 46.12  0.00           C
ATOM    519  C    MET A 130      -8.259   0.450   4.331 46.12  0.00           C
ATOM    520  O    MET A 130      -7.805   1.474   4.842 46.12  0.00           O
ATOM    521  N    GLN A 131      -7.535  -0.357   3.563 56.30  0.00           N
ATOM    522  CA   GLN A 131      -6.170  -0.024   3.174 56.30  0.00           C
ATOM    523  C    GLN A 131      -6.106   1.342   2.502 56.30  0.00           C
ATOM    524  O    GLN A 131      -5.254   2.166   2.831 56.30  0.00           O
ATOM    525  N    LEU A 132      -7.013   1.574   1.559 53.92  0.00           N
ATOM    526  CA   LEU A 132      -7.030   2.821   0.804 53.92  0.00           C
ATOM    527  C    LEU A 132      -7.169   4.023   1.728 53.92  0.00           C
ATOM    528  O    LEU A 132      -6.457   5.016   1.583 53.92  0.00           O
ATOM    529  N    GLN A 133      -8.093   3.928   2.679 50.81  0.00           N
ATOM    530  CA   GLN A 133      -8.322   5.004   3.636 50.81  0.00           C
ATOM    531  C    GLN A 133      -7.134   5.166   4.576 50.81  0.00           C
ATOM    532  O    GLN A 133      -6.812   6.277   4.998 50.81  0.00           O
ATOM    533  N    ILE A 134      -6.486   4.053   4.901 59.98  0.00           N
ATOM    534  CA   ILE A 134      -5.266   4.081   5.699 59.98  0.00           C
ATOM    535  C    ILE A 134      -4.118   4.720   4.927 59.98  0.00           C
ATOM    536  O    ILE A 134      -3.253   5.373   5.510 59.98  0.00           O
ATOM    537  N    TYR A 135      -4.116   4.528   3.612 55.08  0.00           N
ATOM    538  CA   TYR A 135      -3.146   5.183   2.743 55.08  0.00           C
ATOM    539  C    TYR A 135      -3.415   6.679   2.646 55.08  0.00           C
ATOM    540  O    TYR A 135      -2.488   7.480   2.536 55.08  0.00           O
ATOM    541  N    LEU A 136      -4.690   7.049   2.688 49.84  0.00           N
ATOM    542  CA   LEU A 136      -5.077   8.450   2.807 49.84  0.00           C
ATOM    543  C    LEU A 136      -4.537   9.065   4.091 49.84  0.00           C
ATOM    544  O    LEU A 136      -4.006  10.175   4.083 49.84  0.00           O
ATOM    545  N    TRP A 137      -4.675   8.336   5.193 49.66  0.00           N
ATOM    546  CA   TRP A 137      -4.039   8.718   6.449 49.66  0.00           C
ATOM    547  C    TRP A 137      -2.540   8.918   6.268 49.66  0.00           C
ATOM    548  O    TRP A 137      -1.983   9.929   6.696 49.66  0.00           O
ATOM    549  N    LEU A 138      -1.891   7.948   5.633 53.75  0.00           N
ATOM    550  CA   LEU A 138      -0.449   8.000   5.424 53.75  0.00           C
ATOM    551  C    LEU A 138      -0.035   9.307   4.759 53.75  0.00           C
ATOM    552  O    LEU A 138       0.918   9.957   5.187 53.75  0.00           O
ATOM    553  N    PHE A 139      -0.758   9.686   3.711 48.93  0.00           N
ATOM    554  CA   PHE A 139      -0.378  10.832   2.893 48.93  0.00           C
ATOM    555  C    PHE A 139      -1.228  12.052   3.226 48.93  0.00           C
ATOM    556  O    PHE A 139      -1.148  13.078   2.552 48.93  0.00           O
ATOM    557  N    SER A 140      -2.043  11.932   4.269 50.30  0.00           N
ATOM    558  CA   SER A 140      -2.911  13.025   4.691 50.30  0.00           C
ATOM    559  C    SER A 140      -3.799  13.496   3.547 50.30  0.00           C
ATOM    560  O    SER A 140      -4.050  14.692   3.395 50.30  0.00           O
ATOM    561  N    ALA A 141      -4.273  12.550   2.743 48.21  0.00           N
ATOM    562  CA   ALA A 141      -5.101  12.870   1.588 48.21  0.00           C
ATOM    563  C    ALA A 141      -6.568  13.001   1.981 48.21  0.00           C
ATOM    564  O    ALA A 141      -6.973  12.562   3.057 48.21  0.00           O
ATOM    565  N    GLU A 142      -7.358  13.608   1.103 28.93  0.00           N
ATOM    566  CA   GLU A 142      -8.783  13.791   1.353 28.93  0.00           C
ATOM    567  C    GLU A 142      -9.619  12.854   0.491 28.93  0.00           C
ATOM    568  O    GLU A 142     -10.706  12.435   0.888 28.93  0.00           O
ATOM    569  N    LYS A 143      -9.105  12.528  -0.689 52.21  0.00           N
ATOM    570  CA   LYS A 143      -9.728  11.527  -1.547 52.21  0.00           C
ATOM    571  C    LYS A 143      -8.721  10.472  -1.984 52.21  0.00           C
ATOM    572  O    LYS A 143      -7.518  10.620  -1.769 52.21  0.00           O
ATOM    573  N    GLY A 144      -9.219   9.405  -2.601 52.67  0.00           N
ATOM    574  CA   GLY A 144      -8.356   8.370  -3.160 52.67  0.00           C
ATOM    575  C    GLY A 144      -9.162   7.355  -3.959 52.67  0.00           C
ATOM    576  O    GLY A 144     -10.391   7.344  -3.907 52.67  0.00           O
ATOM    577  N    ILE A 145      -8.461   6.502  -4.698 51.81  0.00           N
ATOM    578  CA   ILE A 145      -9.110   5.491  -5.525 51.81  0.00           C
ATOM    579  C    ILE A 145      -8.251   4.239  -5.642 51.81  0.00           C
ATOM    580  O    ILE A 145      -7.023   4.320  -5.682 51.81  0.00           O
ATOM    581  N    LEU A 146      -8.903   3.083  -5.698 54.22  0.00           N
ATOM    582  CA   LEU A 146      -8.198   1.806  -5.697 54.22  0.00           C
ATOM    583  C    LEU A 146      -8.839   0.824  -6.670 54.22  0.00           C
ATOM    584  O    LEU A 146     -10.051   0.612  -6.645 54.22  0.00           O
ATOM    585  N    VAL A 147      -8.017   0.227  -7.527 56.90  0.00           N
ATOM    586  CA   VAL A 147      -8.482  -0.813  -8.437 56.90  0.00           C
ATOM    587  C    VAL A 147      -7.746  -2.124  -8.197 56.90  0.00           C
ATOM    588  O    VAL A 147      -6.521  -2.190  -8.307 56.90  0.00           O
ATOM    589  N    TYR A 148      -8.499  -3.169  -7.868 56.66  0.00           N
ATOM    590  CA   TYR A 148      -7.913  -4.453  -7.504 56.66  0.00           C
ATOM    591  C    TYR A 148      -8.250  -5.523  -8.533 56.66  0.00           C
ATOM    592  O    TYR A 148      -9.397  -5.958  -8.639 56.66  0.00           O
```

```
ATOM    593  N    ILE A 149      -7.243  -5.947  -9.291 48.41  0.00           N
ATOM    594  CA   ILE A 149      -7.464  -6.801 -10.451 48.41  0.00           C
ATOM    595  C    ILE A 149      -7.029  -8.234 -10.171 48.41  0.00           C
ATOM    596  O    ILE A 149      -5.836  -8.527 -10.090 48.41  0.00           O
ATOM    597  N    THR A 150      -8.004  -9.125 -10.024 51.51  0.00           N
ATOM    598  CA   THR A 150      -7.723 -10.535  -9.779 51.51  0.00           C
ATOM    599  C    THR A 150      -8.141 -11.394 -10.965 51.51  0.00           C
ATOM    600  O    THR A 150      -8.932 -10.966 -11.806 51.51  0.00           O
ATOM    601  N    PRO A 151      -7.604 -12.607 -11.028 42.72  0.00           N
ATOM    602  CA   PRO A 151      -8.004 -13.570 -12.046 42.72  0.00           C
ATOM    603  C    PRO A 151      -9.510 -13.806 -12.020 42.72  0.00           C
ATOM    604  O    PRO A 151     -10.111 -14.152 -13.037 42.72  0.00           O
ATOM    605  N    ASP A 152     -10.113 -13.616 -10.852 49.44  0.00           N
ATOM    606  CA   ASP A 152     -11.548 -13.815 -10.690 49.44  0.00           C
ATOM    607  C    ASP A 152     -12.335 -12.644 -11.264 49.44  0.00           C
ATOM    608  O    ASP A 152     -13.229 -12.830 -12.090 49.44  0.00           O
ATOM    609  N    ARG A 153     -11.997 -11.438 -10.822 50.16  0.00           N
ATOM    610  CA   ARG A 153     -12.718 -10.240 -11.237 50.16  0.00           C
ATOM    611  C    ARG A 153     -11.969  -8.978 -10.831 50.16  0.00           C
ATOM    612  O    ARG A 153     -10.963  -9.041 -10.126 50.16  0.00           O
ATOM    613  N    ILE A 154     -12.466  -7.831 -11.281 49.79  0.00           N
ATOM    614  CA   ILE A 154     -11.872  -6.547 -10.927 49.79  0.00           C
ATOM    615  C    ILE A 154     -12.769  -5.769  -9.974 49.79  0.00           C
ATOM    616  O    ILE A 154     -13.904  -5.430 -10.310 49.79  0.00           O
ATOM    617  N    ALA A 155     -12.254  -5.487  -8.782 50.43  0.00           N
ATOM    618  CA   ALA A 155     -13.002  -4.732  -7.784 50.43  0.00           C
ATOM    619  C    ALA A 155     -12.555  -3.276  -7.746 50.43  0.00           C
ATOM    620  O    ALA A 155     -11.360  -2.985  -7.702 50.43  0.00           O
ATOM    621  N    GLU A 156     -13.522  -2.365  -7.764 50.47  0.00           N
ATOM    622  CA   GLU A 156     -13.228  -0.938  -7.799 50.47  0.00           C
ATOM    623  C    GLU A 156     -13.694  -0.248  -6.524 50.47  0.00           C
ATOM    624  O    GLU A 156     -14.822  -0.449  -6.074 50.47  0.00           O
ATOM    625  N    TYR A 157     -12.819   0.568  -5.944 38.52  0.00           N
ATOM    626  CA   TYR A 157     -13.153   1.318  -4.739 38.52  0.00           C
ATOM    627  C    TYR A 157     -12.754   2.781  -4.874 38.52  0.00           C
ATOM    628  O    TYR A 157     -11.762   3.108  -5.525 38.52  0.00           O
ATOM    629  N    GLU A 158     -13.534   3.661  -4.253 47.79  0.00           N
ATOM    630  CA   GLU A 158     -13.259   5.091  -4.295 47.79  0.00           C
ATOM    631  C    GLU A 158     -13.680   5.772  -3.000 47.79  0.00           C
ATOM    632  O    GLU A 158     -14.786   5.553  -2.502 47.79  0.00           O
ATOM    633  N    ILE A 159     -12.794   6.599  -2.456 53.24  0.00           N
ATOM    634  CA   ILE A 159     -13.075   7.318  -1.219 53.24  0.00           C
ATOM    635  C    ILE A 159     -13.140   8.821  -1.458 53.24  0.00           C
ATOM    636  O    ILE A 159     -12.178   9.426  -1.933 53.24  0.00           O
ATOM    637  N    ASN A 160     -14.279   9.420  -1.127 60.63  0.00           N
ATOM    638  CA   ASN A 160     -14.433  10.868  -1.200 60.63  0.00           C
ATOM    639  C    ASN A 160     -14.303  11.506   0.177 60.63  0.00           C
ATOM    640  O    ASN A 160     -13.991  12.691   0.295 60.63  0.00           O
ATOM    641  N    GLU A 161     -14.544  10.715   1.216 50.70  0.00           N
ATOM    642  CA   GLU A 161     -14.460  11.204   2.587 50.70  0.00           C
ATOM    643  C    GLU A 161     -13.012  11.417   3.008 50.70  0.00           C
ATOM    644  O    GLU A 161     -12.178  10.523   2.873 50.70  0.00           O
ATOM    645  N    PRO A 162     -12.720  12.609   3.518 52.96  0.00           N
ATOM    646  CA   PRO A 162     -11.372  12.942   3.963 52.96  0.00           C
ATOM    647  C    PRO A 162     -10.992  12.154   5.209 52.96  0.00           C
ATOM    648  O    PRO A 162     -11.838  11.862   6.053 52.96  0.00           O
ATOM    649  N    LEU A 163      -9.713  11.809   5.317 52.16  0.00           N
ATOM    650  CA   LEU A 163      -9.228  11.005   6.433 52.16  0.00           C
ATOM    651  C    LEU A 163      -8.789  11.885   7.597 52.16  0.00           C
ATOM    652  O    LEU A 163      -7.630  12.288   7.679 52.16  0.00           O
ATOM    653  N    ASP A 164      -9.725  12.179   8.494 57.09  0.00           N
ATOM    654  CA   ASP A 164      -9.415  12.930   9.704 57.09  0.00           C
ATOM    655  C    ASP A 164      -9.518  12.048  10.942 57.09  0.00           C
ATOM    656  O    ASP A 164      -9.729  10.840  10.839 57.09  0.00           O
ATOM    657  N    GLU A 165      -9.369  12.660  12.112 61.08  0.00           N
ATOM    658  CA   GLU A 165      -9.411  11.926  13.371 61.08  0.00           C
ATOM    659  C    GLU A 165     -10.729  11.178  13.528 61.08  0.00           C
ATOM    660  O    GLU A 165     -10.750  10.012  13.924 61.08  0.00           O
ATOM    661  N    ALA A 166     -11.829  11.856  13.218 53.69  0.00           N
ATOM    662  CA   ALA A 166     -13.156  11.271  13.360 53.69  0.00           C
ATOM    663  C    ALA A 166     -13.324  10.058  12.453 53.69  0.00           C
ATOM    664  O    ALA A 166     -13.949   9.069  12.832 53.69  0.00           O
ATOM    665  N    THR A 167     -12.762  10.143  11.252 50.62  0.00           N
ATOM    666  CA   THR A 167     -12.809   9.036  10.305 50.62  0.00           C
ATOM    667  C    THR A 167     -11.999   7.847  10.805 50.62  0.00           C
ATOM    668  O    THR A 167     -12.410   6.696  10.658 50.62  0.00           O
ATOM    669  N    ILE A 168     -10.844   8.133  11.398 56.16  0.00           N
ATOM    670  CA   ILE A 168     -10.014   7.095  11.998 56.16  0.00           C
ATOM    671  C    ILE A 168     -10.759   6.368  13.109 56.16  0.00           C
ATOM    672  O    ILE A 168     -10.712   5.141  13.202 56.16  0.00           O
ATOM    673  N    VAL A 169     -11.447   7.131  13.952 58.37  0.00           N
ATOM    674  CA   VAL A 169     -12.234   6.557  15.036 58.37  0.00           C
ATOM    675  C    VAL A 169     -13.375   5.704  14.498 58.37  0.00           C
ATOM    676  O    VAL A 169     -13.654   4.623  15.018 58.37  0.00           O
ATOM    677  N    ARG A 170     -14.033   6.195  13.453 59.48  0.00           N
ATOM    678  CA   ARG A 170     -15.111   5.455  12.809 59.48  0.00           C
ATOM    679  C    ARG A 170     -14.615   4.122  12.264 59.48  0.00           C
ATOM    680  O    ARG A 170     -15.248   3.085  12.464 59.48  0.00           O
ATOM    681  N    LEU A 171     -13.481   4.156  11.574 49.98  0.00           N
ATOM    682  CA   LEU A 171     -12.902   2.952  10.990 49.98  0.00           C
ATOM    683  C    LEU A 171     -12.490   1.960  12.068 49.98  0.00           C
ATOM    684  O    LEU A 171     -12.612   0.748  11.890 49.98  0.00           O
```

```
ATOM    685  N   ALA A 172     -12.002   2.481  13.189 58.47  0.00           N
ATOM    686  CA  ALA A 172     -11.698   1.653  14.350 58.47  0.00           C
ATOM    687  C   ALA A 172     -12.945   0.949  14.867 58.47  0.00           C
ATOM    688  O   ALA A 172     -12.945  -0.263  15.078 58.47  0.00           O
ATOM    689  N   GLU A 173     -14.011   1.718  15.071 69.53  0.00           N
ATOM    690  CA  GLU A 173     -15.255   1.176  15.603 69.53  0.00           C
ATOM    691  C   GLU A 173     -15.873   0.167  14.643 69.53  0.00           C
ATOM    692  O   GLU A 173     -16.553  -0.769  15.065 69.53  0.00           O
ATOM    693  N   ASP A 174     -15.629   0.361  13.351 57.67  0.00           N
ATOM    694  CA  ASP A 174     -16.150  -0.539  12.330 57.67  0.00           C
ATOM    695  C   ASP A 174     -15.596  -1.948  12.504 57.67  0.00           C
ATOM    696  O   ASP A 174     -16.242  -2.930  12.137 57.67  0.00           O
ATOM    697  N   THR A 175     -14.396  -2.041  13.066 51.60  0.00           N
ATOM    698  CA  THR A 175     -13.741  -3.329  13.267 51.60  0.00           C
ATOM    699  C   THR A 175     -13.899  -3.810  14.703 51.60  0.00           C
ATOM    700  O   THR A 175     -13.870  -5.010  14.974 51.60  0.00           O
ATOM    701  N   ILE A 176     -14.065  -2.864  15.623 44.53  0.00           N
ATOM    702  CA  ILE A 176     -14.351  -3.193  17.015 44.53  0.00           C
ATOM    703  C   ILE A 176     -15.669  -3.944  17.144 44.53  0.00           C
ATOM    704  O   ILE A 176     -15.787  -4.878  17.938 44.53  0.00           O
ATOM    705  N   MET A 177     -16.660  -3.533  16.361 55.85  0.00           N
ATOM    706  CA  MET A 177     -17.988  -4.128  16.428 55.85  0.00           C
ATOM    707  C   MET A 177     -18.147  -5.238  15.398 55.85  0.00           C
ATOM    708  O   MET A 177     -19.247  -5.745  15.179 55.85  0.00           O
ATOM    709  N   LEU A 178     -17.040  -5.614  14.765 59.55  0.00           N
ATOM    710  CA  LEU A 178     -17.061  -6.635  13.725 59.55  0.00           C
ATOM    711  C   LEU A 178     -16.166  -7.812  14.090 59.55  0.00           C
ATOM    712  O   LEU A 178     -14.962  -7.650  14.291 59.55  0.00           O
ATOM    713  N   GLN A 179     -16.760  -8.997  14.174 54.12  0.00           N
ATOM    714  CA  GLN A 179     -16.018 -10.204  14.515 54.12  0.00           C
ATOM    715  C   GLN A 179     -15.066 -10.602  13.394 54.12  0.00           C
ATOM    716  O   GLN A 179     -13.882 -10.846  13.628 54.12  0.00           O
ATOM    717  N   ASN A 180     -15.590 -10.666  12.175 48.75  0.00           N
ATOM    718  CA  ASN A 180     -14.781 -11.001  11.009 48.75  0.00           C
ATOM    719  C   ASN A 180     -14.136  -9.758  10.411 48.75  0.00           C
ATOM    720  O   ASN A 180     -14.413  -9.392   9.268 48.75  0.00           O
ATOM    721  N   SER A 181     -13.277  -9.110  11.190 62.92  0.00           N
ATOM    722  CA  SER A 181     -12.619  -7.885  10.751 62.92  0.00           C
ATOM    723  C   SER A 181     -11.434  -8.190   9.843 62.92  0.00           C
ATOM    724  O   SER A 181     -10.838  -9.264   9.925 62.92  0.00           O
ATOM    725  N   PRO A 182     -11.098  -7.240   8.977 62.16  0.00           N
ATOM    726  CA  PRO A 182      -9.967  -7.395   8.071 62.16  0.00           C
ATOM    727  C   PRO A 182      -8.644  -7.269   8.814 62.16  0.00           C
ATOM    728  O   PRO A 182      -8.618  -6.997  10.014 62.16  0.00           O
ATOM    729  N   ARG A 183      -7.545  -7.472   8.094 55.41  0.00           N
ATOM    730  CA  ARG A 183      -6.214  -7.307   8.666 55.41  0.00           C
ATOM    731  C   ARG A 183      -5.410  -6.265   7.899 55.41  0.00           C
ATOM    732  O   ARG A 183      -5.899  -5.676   6.936 55.41  0.00           O
ATOM    733  N   PHE A 184      -4.174  -6.042   8.333 42.08  0.00           N
ATOM    734  CA  PHE A 184      -3.260  -5.161   7.617 42.08  0.00           C
ATOM    735  C   PHE A 184      -1.809  -5.572   7.842 42.08  0.00           C
ATOM    736  O   PHE A 184      -1.504  -6.328   8.764 42.08  0.00           O
ATOM    737  N   ASN A 185      -0.919  -5.070   6.992 36.30  0.00           N
ATOM    738  CA  ASN A 185       0.497  -5.403   7.082 36.30  0.00           C
ATOM    739  C   ASN A 185       1.075  -4.990   8.431 36.30  0.00           C
ATOM    740  O   ASN A 185       1.114  -3.806   8.764 36.30  0.00           O
ATOM    741  N   TRP A 186       1.523  -5.975   9.203 34.90  0.00           N
ATOM    742  CA  TRP A 186       1.956  -5.736  10.574 34.90  0.00           C
ATOM    743  C   TRP A 186       3.144  -4.783  10.620 34.90  0.00           C
ATOM    744  O   TRP A 186       3.358  -4.091  11.614 34.90  0.00           O
ATOM    745  N   GLU A 187       3.913  -4.753   9.537 46.05  0.00           N
ATOM    746  CA  GLU A 187       5.099  -3.908   9.463 46.05  0.00           C
ATOM    747  C   GLU A 187       4.725  -2.431   9.488 46.05  0.00           C
ATOM    748  O   GLU A 187       5.479  -1.600   9.992 46.05  0.00           O
ATOM    749  N   CYS A 188       3.558  -2.112   8.941 53.49  0.00           N
ATOM    750  CA  CYS A 188       3.076  -0.736   8.910 53.49  0.00           C
ATOM    751  C   CYS A 188       2.514  -0.320  10.263 53.49  0.00           C
ATOM    752  O   CYS A 188       1.668  -1.008  10.834 53.49  0.00           O
ATOM    753  N   LYS A 189       2.990   0.812  10.773 40.03  0.00           N
ATOM    754  CA  LYS A 189       2.582   1.291  12.087 40.03  0.00           C
ATOM    755  C   LYS A 189       1.621   2.467  11.971 40.03  0.00           C
ATOM    756  O   LYS A 189       1.108   2.965  12.973 40.03  0.00           O
ATOM    757  N   TYR A 190       1.379   2.907  10.740 37.23  0.00           N
ATOM    758  CA  TYR A 190       0.476   4.025  10.490 37.23  0.00           C
ATOM    759  C   TYR A 190      -0.953   3.542  10.275 37.23  0.00           C
ATOM    760  O   TYR A 190      -1.852   4.336   9.999 37.23  0.00           O
ATOM    761  N   CYS A 191      -1.156   2.235  10.402 43.81  0.00           N
ATOM    762  CA  CYS A 191      -2.475   1.643  10.217 43.81  0.00           C
ATOM    763  C   CYS A 191      -3.212   1.510  11.543 43.81  0.00           C
ATOM    764  O   CYS A 191      -2.591   1.446  12.605 43.81  0.00           O
ATOM    765  N   ILE A 192      -4.538   1.470  11.476 41.27  0.00           N
ATOM    766  CA  ILE A 192      -5.361   1.335  12.672 41.27  0.00           C
ATOM    767  C   ILE A 192      -5.216  -0.050  13.287 41.27  0.00           C
ATOM    768  O   ILE A 192      -5.561  -1.056  12.666 41.27  0.00           O
ATOM    769  N   PHE A 193      -4.704  -0.097  14.512 42.93  0.00           N
ATOM    770  CA  PHE A 193      -4.322  -1.359  15.135 42.93  0.00           C
ATOM    771  C   PHE A 193      -5.546  -2.123  15.627 42.93  0.00           C
ATOM    772  O   PHE A 193      -5.497  -3.338  15.814 42.93  0.00           O
ATOM    773  N   SER A 194      -6.643  -1.401  15.835 34.28  0.00           N
ATOM    774  CA  SER A 194      -7.890  -2.014  16.275 34.28  0.00           C
ATOM    775  C   SER A 194      -8.550  -2.794  15.145 34.28  0.00           C
ATOM    776  O   SER A 194      -9.547  -3.486  15.354 34.28  0.00           O
```

```
ATOM    777  N   VAL A 195      -7.988  -2.678  13.946 39.35  0.00           N
ATOM    778  CA  VAL A 195      -8.430  -3.481  12.812 39.35  0.00           C
ATOM    779  C   VAL A 195      -8.156  -4.962  13.045 39.35  0.00           C
ATOM    780  O   VAL A 195      -8.973  -5.815  12.701 39.35  0.00           O
ATOM    781  N   ILE A 196      -7.002  -5.259  13.633 37.38  0.00           N
ATOM    782  CA  ILE A 196      -6.609  -6.638  13.894 37.38  0.00           C
ATOM    783  C   ILE A 196      -6.961  -7.051  15.318 37.38  0.00           C
ATOM    784  O   ILE A 196      -7.494  -8.138  15.545 37.38  0.00           O
ATOM    785  N   CYS A 197      -6.661  -6.179  16.273 52.86  0.00           N
ATOM    786  CA  CYS A 197      -6.797  -6.513  17.686 52.86  0.00           C
ATOM    787  C   CYS A 197      -7.147  -5.282  18.511 52.86  0.00           C
ATOM    788  O   CYS A 197      -6.269  -4.629  19.074 52.86  0.00           O
ATOM    789  N   PRO A 198      -8.437  -4.969  18.581 49.63  0.00           N
ATOM    790  CA  PRO A 198      -8.914  -3.855  19.391 49.63  0.00           C
ATOM    791  C   PRO A 198      -8.736  -4.139  20.877 49.63  0.00           C
ATOM    792  O   PRO A 198      -8.658  -3.218  21.689 49.63  0.00           O
ATOM    793  N   ALA A 199      -8.675  -5.419  21.226 49.32  0.00           N
ATOM    794  CA  ALA A 199      -8.504  -5.827  22.615 49.32  0.00           C
ATOM    795  C   ALA A 199      -7.152  -5.382  23.158 49.32  0.00           C
ATOM    796  O   ALA A 199      -7.015  -5.094  24.347 49.32  0.00           O
ATOM    797  N   LYS A 200      -6.157  -5.326  22.280 64.63  0.00           N
ATOM    798  CA  LYS A 200      -4.826  -4.869  22.660 64.63  0.00           C
ATOM    799  C   LYS A 200      -4.666  -3.374  22.413 64.63  0.00           C
ATOM    800  O   LYS A 200      -4.394  -2.609  23.338 64.63  0.00           O
ATOM    801  N   LEU A 201      -4.838  -2.964  21.162 49.70  0.00           N
ATOM    802  CA  LEU A 201      -4.684  -1.563  20.787 49.70  0.00           C
ATOM    803  C   LEU A 201      -5.983  -0.996  20.228 49.70  0.00           C
ATOM    804  O   LEU A 201      -6.253  -1.099  19.032 49.70  0.00           O
ATOM    805  N   THR A 202      -6.785  -0.398  21.102 50.36  0.00           N
ATOM    806  CA  THR A 202      -8.031   0.237  20.688 50.36  0.00           C
ATOM    807  C   THR A 202      -7.765   1.521  19.913 50.36  0.00           C
ATOM    808  O   THR A 202      -8.090   1.613  18.971 50.36  0.00           O
TER     809      THR A 202
ENDMDL
END
REMARK   1 ***********************************************************************
REMARK   1 Start File DEC2_vs_NAT_dSi_colored.pdb
REMARK   1 The occupancy record was modified to represent dSi := Si(DEC)-Si(NAT).
REMARK   1 Occ=0.00 means dSi=-1.461482 kB; Occ=99.99 means dSi=1.491450 kB.
MODEL        0
ATOM      1  N   MET A   1      13.208  10.652   1.184 37.22  0.00           N
ATOM      2  CA  MET A   1      13.950   9.398   1.239 37.22  0.00           C
ATOM      3  C   MET A   1      14.997   9.328   0.135 37.22  0.00           C
ATOM      4  O   MET A   1      16.179   9.584   0.368 37.22  0.00           O
ATOM      5  N   ILE A   2      14.555   8.981  -1.070 27.40  0.00           N
ATOM      6  CA  ILE A   2      15.469   8.753  -2.184 27.40  0.00           C
ATOM      7  C   ILE A   2      16.216  10.028  -2.553 27.40  0.00           C
ATOM      8  O   ILE A   2      17.408   9.993  -2.858 27.40  0.00           O
ATOM      9  N   THR A   3      15.509  11.153  -2.524 41.96  0.00           N
ATOM     10  CA  THR A   3      16.109  12.443  -2.836 41.96  0.00           C
ATOM     11  C   THR A   3      17.319  12.716  -1.951 41.96  0.00           C
ATOM     12  O   THR A   3      18.385  13.092  -2.440 41.96  0.00           O
ATOM     13  N   GLU A   4      17.148  12.526  -0.648 43.40  0.00           N
ATOM     14  CA  GLU A   4      18.230  12.736   0.307 43.40  0.00           C
ATOM     15  C   GLU A   4      19.378  11.765   0.059 43.40  0.00           C
ATOM     16  O   GLU A   4      20.547  12.143   0.120 43.40  0.00           O
ATOM     17  N   PHE A   5      19.036  10.511  -0.221 31.36  0.00           N
ATOM     18  CA  PHE A   5      20.037   9.482  -0.474 31.36  0.00           C
ATOM     19  C   PHE A   5      20.897   9.835  -1.680 31.36  0.00           C
ATOM     20  O   PHE A   5      22.119   9.690  -1.647 31.36  0.00           O
ATOM     21  N   LEU A   6      20.252  10.298  -2.744 28.08  0.00           N
ATOM     22  CA  LEU A   6      20.947  10.600  -3.990 28.08  0.00           C
ATOM     23  C   LEU A   6      21.839  11.826  -3.840 28.08  0.00           C
ATOM     24  O   LEU A   6      22.974  11.842  -4.318 28.08  0.00           O
ATOM     25  N   LEU A   7      21.320  12.852  -3.174 47.44  0.00           N
ATOM     26  CA  LEU A   7      22.075  14.077  -2.943 47.44  0.00           C
ATOM     27  C   LEU A   7      23.280  13.821  -2.048 47.44  0.00           C
ATOM     28  O   LEU A   7      24.363  14.361  -2.277 47.44  0.00           O
ATOM     29  N   LYS A   8      23.087  12.992  -1.027 29.50  0.00           N
ATOM     30  CA  LYS A   8      24.178  12.596  -0.146 29.50  0.00           C
ATOM     31  C   LYS A   8      25.300  11.922  -0.926 29.50  0.00           C
ATOM     32  O   LYS A   8      26.461  12.317  -0.828 29.50  0.00           O
ATOM     33  N   LYS A   9      24.946  10.902  -1.700  0.00  0.00           N
ATOM     34  CA  LYS A   9      25.935  10.087  -2.395  0.00  0.00           C
ATOM     35  C   LYS A   9      26.461  10.797  -3.635  0.00  0.00           C
ATOM     36  O   LYS A   9      27.532  10.466  -4.144  0.00  0.00           O
ATOM     37  N   LYS A  10      25.703  11.776  -4.117 56.51  0.00           N
ATOM     38  CA  LYS A  10      26.183  12.674  -5.159 56.51  0.00           C
ATOM     39  C   LYS A  10      27.364  13.505  -4.672 56.51  0.00           C
ATOM     40  O   LYS A  10      28.363  13.653  -5.374 56.51  0.00           O
ATOM     41  N   LEU A  11      27.241  14.045  -3.464 56.08  0.00           N
ATOM     42  CA  LEU A  11      28.329  14.791  -2.844 56.08  0.00           C
ATOM     43  C   LEU A  11      29.495  13.876  -2.494 56.08  0.00           C
ATOM     44  O   LEU A  11      30.657  14.273  -2.582 56.08  0.00           O
ATOM     45  N   GLU A  12      29.178  12.648  -2.097 40.56  0.00           N
ATOM     46  CA  GLU A  12      30.200  11.647  -1.811 40.56  0.00           C
ATOM     47  C   GLU A  12      30.978  11.279  -3.068 40.56  0.00           C
ATOM     48  O   GLU A  12      32.180  11.021  -3.012 40.56  0.00           O
ATOM     49  N   GLU A  13      30.284  11.255  -4.201 43.09  0.00           N
ATOM     50  CA  GLU A  13      30.931  11.044  -5.490 43.09  0.00           C
ATOM     51  C   GLU A  13      31.823  12.222  -5.859 43.09  0.00           C
ATOM     52  O   GLU A  13      32.930  12.041  -6.365 43.09  0.00           O
```

ATOM	53	N	HIS	A	14	31.334	13.432	-5.601	62.74	0.00	N
ATOM	54	CA	HIS	A	14	32.102	14.641	-5.869	62.74	0.00	C
ATOM	55	C	HIS	A	14	33.423	14.635	-5.112	62.74	0.00	C
ATOM	56	O	HIS	A	14	34.457	15.044	-5.642	62.74	0.00	O
ATOM	57	N	LEU	A	15	33.384	14.169	-3.868	54.44	0.00	N
ATOM	58	CA	LEU	A	15	34.585	14.074	-3.047	54.44	0.00	C
ATOM	59	C	LEU	A	15	35.462	12.910	-3.486	54.44	0.00	C
ATOM	60	O	LEU	A	15	36.677	13.053	-3.626	54.44	0.00	O
ATOM	61	N	SER	A	16	34.840	11.756	-3.702	50.75	0.00	N
ATOM	62	CA	SER	A	16	35.567	10.557	-4.102	50.75	0.00	C
ATOM	63	C	SER	A	16	34.737	9.700	-5.050	50.75	0.00	C
ATOM	64	O	SER	A	16	34.060	8.764	-4.625	50.75	0.00	O
ATOM	65	N	HIS	A	17	34.795	10.026	-6.337	47.33	0.00	N
ATOM	66	CA	HIS	A	17	33.972	9.352	-7.335	47.33	0.00	C
ATOM	67	C	HIS	A	17	34.395	7.899	-7.508	47.33	0.00	C
ATOM	68	O	HIS	A	17	33.632	7.077	-8.013	47.33	0.00	O
ATOM	69	N	VAL	A	18	35.615	7.589	-7.084	46.62	0.00	N
ATOM	70	CA	VAL	A	18	36.121	6.223	-7.138	46.62	0.00	C
ATOM	71	C	VAL	A	18	35.542	5.377	-6.011	46.62	0.00	C
ATOM	72	O	VAL	A	18	35.530	4.148	-6.087	46.62	0.00	O
ATOM	73	N	LYS	A	19	35.061	6.041	-4.966	50.02	0.00	N
ATOM	74	CA	LYS	A	19	34.504	5.351	-3.809	50.02	0.00	C
ATOM	75	C	LYS	A	19	32.991	5.220	-3.920	50.02	0.00	C
ATOM	76	O	LYS	A	19	32.455	4.112	-3.952	50.02	0.00	O
ATOM	77	N	GLU	A	20	32.307	6.357	-3.979	23.75	0.00	N
ATOM	78	CA	GLU	A	20	30.849	6.372	-4.019	23.75	0.00	C
ATOM	79	C	GLU	A	20	30.339	6.969	-5.325	23.75	0.00	C
ATOM	80	O	GLU	A	20	31.031	7.758	-5.969	23.75	0.00	O
ATOM	81	N	GLU	A	21	29.127	6.586	-5.711	36.12	0.00	N
ATOM	82	CA	GLU	A	21	28.493	7.137	-6.903	36.12	0.00	C
ATOM	83	C	GLU	A	21	27.108	7.686	-6.586	36.12	0.00	C
ATOM	84	O	GLU	A	21	26.530	7.376	-5.544	36.12	0.00	O
ATOM	85	N	ASN	A	22	26.579	8.503	-7.491	44.32	0.00	N
ATOM	86	CA	ASN	A	22	25.247	9.072	-7.325	44.32	0.00	C
ATOM	87	C	ASN	A	22	24.169	8.014	-7.520	44.32	0.00	C
ATOM	88	O	ASN	A	22	23.479	7.997	-8.539	44.32	0.00	O
ATOM	89	N	THR	A	23	24.027	7.131	-6.536	60.13	0.00	N
ATOM	90	CA	THR	A	23	23.005	6.094	-6.579	60.13	0.00	C
ATOM	91	C	THR	A	23	22.565	5.694	-5.176	60.13	0.00	C
ATOM	92	O	THR	A	23	22.987	6.296	-4.189	60.13	0.00	O
ATOM	93	N	ILE	A	24	21.716	4.675	-5.094	61.59	0.00	N
ATOM	94	CA	ILE	A	24	21.183	4.223	-3.815	61.59	0.00	C
ATOM	95	C	ILE	A	24	21.969	3.033	-3.281	61.59	0.00	C
ATOM	96	O	ILE	A	24	21.986	1.963	-3.891	61.59	0.00	O
ATOM	97	N	TYR	A	25	22.618	3.223	-2.138	56.15	0.00	N
ATOM	98	CA	TYR	A	25	23.339	2.144	-1.477	56.15	0.00	C
ATOM	99	C	TYR	A	25	22.549	1.596	-0.294	56.15	0.00	C
ATOM	100	O	TYR	A	25	21.750	2.308	0.314	56.15	0.00	O
ATOM	101	N	VAL	A	26	22.778	0.327	0.027	70.44	0.00	N
ATOM	102	CA	VAL	A	26	22.094	-0.316	1.143	70.44	0.00	C
ATOM	103	C	VAL	A	26	22.264	0.484	2.427	70.44	0.00	C
ATOM	104	O	VAL	A	26	21.338	0.588	3.232	70.44	0.00	O
ATOM	105	N	THR	A	27	23.451	1.050	2.614	57.80	0.00	N
ATOM	106	CA	THR	A	27	23.751	1.825	3.812	57.80	0.00	C
ATOM	107	C	THR	A	27	22.677	2.874	4.072	57.80	0.00	C
ATOM	108	O	THR	A	27	22.298	3.118	5.217	57.80	0.00	O
ATOM	109	N	ASP	A	28	22.192	3.494	3.002	52.14	0.00	N
ATOM	110	CA	ASP	A	28	21.183	4.540	3.114	52.14	0.00	C
ATOM	111	C	ASP	A	28	19.777	3.964	3.009	52.14	0.00	C
ATOM	112	O	ASP	A	28	18.846	4.453	3.649	52.14	0.00	O
ATOM	113	N	LEU	A	29	19.629	2.923	2.197	66.81	0.00	N
ATOM	114	CA	LEU	A	29	18.328	2.303	1.975	66.81	0.00	C
ATOM	115	C	LEU	A	29	17.700	1.853	3.287	66.81	0.00	C
ATOM	116	O	LEU	A	29	16.496	1.997	3.492	66.81	0.00	O
ATOM	117	N	VAL	A	30	18.525	1.308	4.175	55.91	0.00	N
ATOM	118	CA	VAL	A	30	18.036	0.731	5.422	55.91	0.00	C
ATOM	119	C	VAL	A	30	17.506	1.810	6.358	55.91	0.00	C
ATOM	120	O	VAL	A	30	16.894	1.509	7.383	55.91	0.00	O
ATOM	121	N	ARG	A	31	17.743	3.066	5.998	86.64	0.00	N
ATOM	122	CA	ARG	A	31	17.255	4.193	6.784	86.64	0.00	C
ATOM	123	C	ARG	A	31	15.736	4.284	6.733	86.64	0.00	C
ATOM	124	O	ARG	A	31	15.114	4.931	7.575	86.64	0.00	O
ATOM	125	N	CYS	A	32	15.142	3.630	5.740	50.59	0.00	N
ATOM	126	CA	CYS	A	32	13.691	3.609	5.595	50.59	0.00	C
ATOM	127	C	CYS	A	32	13.007	3.401	6.941	50.59	0.00	C
ATOM	128	O	CYS	A	32	13.276	2.425	7.640	50.59	0.00	O
ATOM	129	N	PRO	A	33	12.120	4.325	7.296	50.11	0.00	N
ATOM	130	CA	PRO	A	33	11.462	4.295	8.596	50.11	0.00	C
ATOM	131	C	PRO	A	33	10.497	3.121	8.697	50.11	0.00	C
ATOM	132	O	PRO	A	33	10.210	2.632	9.791	50.11	0.00	O
ATOM	133	N	ARG	A	34	9.998	2.671	7.551	34.08	0.00	N
ATOM	134	CA	ARG	A	34	9.094	1.528	7.505	34.08	0.00	C
ATOM	135	C	ARG	A	34	9.788	0.257	7.977	34.08	0.00	C
ATOM	136	O	ARG	A	34	9.242	-0.497	8.783	34.08	0.00	O
ATOM	137	N	ARG	A	35	10.993	0.024	7.469	25.98	0.00	N
ATOM	138	CA	ARG	A	35	11.803	-1.107	7.906	25.98	0.00	C
ATOM	139	C	ARG	A	35	12.069	-1.047	9.405	25.98	0.00	C
ATOM	140	O	ARG	A	35	11.930	-2.047	10.110	25.98	0.00	O
ATOM	141	N	VAL	A	36	12.452	0.131	9.887	52.80	0.00	N
ATOM	142	CA	VAL	A	36	12.781	0.312	11.296	52.80	0.00	C
ATOM	143	C	VAL	A	36	11.563	0.083	12.180	52.80	0.00	C
ATOM	144	O	VAL	A	36	11.652	-0.569	13.221	52.80	0.00	O

ATOM	145	N	ARG	A	37	10.423	0.624	11.760	54.12	0.00	N
ATOM	146	CA	ARG	A	37	9.204	0.551	12.554	54.12	0.00	C
ATOM	147	C	ARG	A	37	8.748	-0.891	12.735	54.12	0.00	C
ATOM	148	O	ARG	A	37	8.289	-1.277	13.810	54.12	0.00	O
ATOM	149	N	TYR	A	38	8.881	-1.685	11.678	45.86	0.00	N
ATOM	150	CA	TYR	A	38	8.579	-3.110	11.747	45.86	0.00	C
ATOM	151	C	TYR	A	38	9.566	-3.839	12.648	45.86	0.00	C
ATOM	152	O	TYR	A	38	9.171	-4.602	13.530	45.86	0.00	O
ATOM	153	N	GLU	A	39	10.854	-3.602	12.421	57.04	0.00	N
ATOM	154	CA	GLU	A	39	11.902	-4.271	13.182	57.04	0.00	C
ATOM	155	C	GLU	A	39	11.712	-4.068	14.679	57.04	0.00	C
ATOM	156	O	GLU	A	39	11.937	-4.983	15.473	57.04	0.00	O
ATOM	157	N	SER	A	40	11.296	-2.865	15.060	46.13	0.00	N
ATOM	158	CA	SER	A	40	11.228	-2.488	16.467	46.13	0.00	C
ATOM	159	C	SER	A	40	10.197	-3.326	17.213	46.13	0.00	C
ATOM	160	O	SER	A	40	10.234	-3.427	18.439	46.13	0.00	O
ATOM	161	N	GLU	A	41	9.277	-3.927	16.465	51.09	0.00	N
ATOM	162	CA	GLU	A	41	8.230	-4.752	17.054	51.09	0.00	C
ATOM	163	C	GLU	A	41	8.410	-6.218	16.684	51.09	0.00	C
ATOM	164	O	GLU	A	41	7.971	-7.110	17.410	51.09	0.00	O
ATOM	165	N	TYR	A	42	9.058	-6.462	15.550	43.68	0.00	N
ATOM	166	CA	TYR	A	42	9.215	-7.817	15.034	43.68	0.00	C
ATOM	167	C	TYR	A	42	10.679	-8.134	14.758	43.68	0.00	C
ATOM	168	O	TYR	A	42	11.112	-8.163	13.605	43.68	0.00	O
ATOM	169	N	LYS	A	43	11.438	-8.372	15.822	60.71	0.00	N
ATOM	170	CA	LYS	A	43	12.859	-8.678	15.696	60.71	0.00	C
ATOM	171	C	LYS	A	43	13.081	-10.157	15.407	60.71	0.00	C
ATOM	172	O	LYS	A	43	14.021	-10.527	14.704	60.71	0.00	O
ATOM	173	N	GLU	A	44	12.209	-10.999	15.952	55.81	0.00	N
ATOM	174	CA	GLU	A	44	12.332	-12.442	15.786	55.81	0.00	C
ATOM	175	C	GLU	A	44	10.962	-13.104	15.696	55.81	0.00	C
ATOM	176	O	GLU	A	44	10.105	-12.896	16.555	55.81	0.00	O
ATOM	177	N	LEU	A	45	10.763	-13.902	14.653	48.24	0.00	N
ATOM	178	CA	LEU	A	45	9.474	-14.537	14.409	48.24	0.00	C
ATOM	179	C	LEU	A	45	9.554	-16.044	14.614	48.24	0.00	C
ATOM	180	O	LEU	A	45	10.588	-16.661	14.356	48.24	0.00	O
ATOM	181	N	ALA	A	46	8.458	-16.632	15.081	62.46	0.00	N
ATOM	182	CA	ALA	A	46	8.364	-18.081	15.215	62.46	0.00	C
ATOM	183	C	ALA	A	46	7.943	-18.730	13.902	62.46	0.00	C
ATOM	184	O	ALA	A	46	7.888	-19.955	13.794	62.46	0.00	O
ATOM	185	N	ILE	A	47	7.647	-17.902	12.907	56.61	0.00	N
ATOM	186	CA	ILE	A	47	7.259	-18.394	11.590	56.61	0.00	C
ATOM	187	C	ILE	A	47	8.364	-18.161	10.569	56.61	0.00	C
ATOM	188	O	ILE	A	47	8.104	-18.073	9.369	56.61	0.00	O
ATOM	189	N	SER	A	48	9.598	-18.061	11.051	57.74	0.00	N
ATOM	190	CA	SER	A	48	10.722	-17.675	10.206	57.74	0.00	C
ATOM	191	C	SER	A	48	11.025	-18.747	9.167	57.74	0.00	C
ATOM	192	O	SER	A	48	11.725	-18.494	8.187	57.74	0.00	O
ATOM	193	N	GLN	A	49	10.493	-19.944	9.388	65.98	0.00	N
ATOM	194	CA	GLN	A	49	10.654	-21.040	8.439	65.98	0.00	C
ATOM	195	C	GLN	A	49	10.079	-20.677	7.076	65.98	0.00	C
ATOM	196	O	GLN	A	49	10.571	-21.133	6.043	65.98	0.00	O
ATOM	197	N	VAL	A	50	9.036	-19.854	7.078	68.48	0.00	N
ATOM	198	CA	VAL	A	50	8.419	-19.395	5.840	68.48	0.00	C
ATOM	199	C	VAL	A	50	8.594	-17.892	5.660	68.48	0.00	C
ATOM	200	O	VAL	A	50	9.024	-17.429	4.604	68.48	0.00	O
ATOM	201	N	TYR	A	51	8.259	-17.135	6.700	34.17	0.00	N
ATOM	202	CA	TYR	A	51	8.376	-15.682	6.658	34.17	0.00	C
ATOM	203	C	TYR	A	51	9.430	-15.186	7.638	34.17	0.00	C
ATOM	204	O	TYR	A	51	9.103	-14.695	8.720	34.17	0.00	O
ATOM	205	N	ALA	A	52	10.695	-15.315	7.256	46.56	0.00	N
ATOM	206	CA	ALA	A	52	11.798	-14.824	8.074	46.56	0.00	C
ATOM	207	C	ALA	A	52	11.755	-13.306	8.202	46.56	0.00	C
ATOM	208	O	ALA	A	52	11.495	-12.598	7.228	46.56	0.00	O
ATOM	209	N	PRO	A	53	12.012	-12.812	9.409	49.85	0.00	N
ATOM	210	CA	PRO	A	53	11.958	-11.380	9.677	49.85	0.00	C
ATOM	211	C	PRO	A	53	13.017	-10.629	8.880	49.85	0.00	C
ATOM	212	O	PRO	A	53	12.828	-9.469	8.517	49.85	0.00	O
ATOM	213	N	SER	A	54	14.133	-11.300	8.610	47.01	0.00	N
ATOM	214	CA	SER	A	54	15.193	-10.724	7.792	47.01	0.00	C
ATOM	215	C	SER	A	54	14.739	-10.544	6.350	47.01	0.00	C
ATOM	216	O	SER	A	54	14.934	-9.483	5.755	47.01	0.00	O
ATOM	217	N	ALA	A	55	14.132	-11.585	5.790	43.60	0.00	N
ATOM	218	CA	ALA	A	55	13.612	-11.529	4.430	43.60	0.00	C
ATOM	219	C	ALA	A	55	12.495	-10.500	4.310	43.60	0.00	C
ATOM	220	O	ALA	A	55	12.371	-9.821	3.291	43.60	0.00	O
ATOM	221	N	ILE	A	56	11.683	-10.390	5.356	26.91	0.00	N
ATOM	222	CA	ILE	A	56	10.598	-9.417	5.385	26.91	0.00	C
ATOM	223	C	ILE	A	56	11.135	-7.993	5.345	26.91	0.00	C
ATOM	224	O	ILE	A	56	10.679	-7.170	4.550	26.91	0.00	O
ATOM	225	N	LEU	A	57	12.103	-7.707	6.207	22.36	0.00	N
ATOM	226	CA	LEU	A	57	12.694	-6.376	6.282	22.36	0.00	C
ATOM	227	C	LEU	A	57	13.305	-5.967	4.947	22.36	0.00	C
ATOM	228	O	LEU	A	57	13.194	-4.815	4.530	22.36	0.00	O
ATOM	229	N	GLY	A	58	13.949	-6.920	4.282	47.90	0.00	N
ATOM	230	CA	GLY	A	58	14.524	-6.678	2.964	47.90	0.00	C
ATOM	231	C	GLY	A	58	13.441	-6.353	1.942	47.90	0.00	C
ATOM	232	O	GLY	A	58	13.588	-5.430	1.141	47.90	0.00	O
ATOM	233	N	ASP	A	59	12.354	-7.115	1.977	47.45	0.00	N
ATOM	234	CA	ASP	A	59	11.253	-6.923	1.041	47.45	0.00	C
ATOM	235	C	ASP	A	59	10.505	-5.628	1.331	47.45	0.00	C
ATOM	236	O	ASP	A	59	9.920	-5.023	0.432	47.45	0.00	O

ATOM	237	N	ILE	A	60	10.529	-5.206	2.590	21.25	0.00	N
ATOM	238	CA	ILE	A	60	9.979	-3.910	2.976	21.25	0.00	C
ATOM	239	C	ILE	A	60	10.769	-2.769	2.350	21.25	0.00	C
ATOM	240	O	ILE	A	60	10.194	-1.779	1.895	21.25	0.00	O
ATOM	241	N	LEU	A	61	12.090	-2.911	2.329	25.12	0.00	N
ATOM	242	CA	LEU	A	61	12.953	-1.958	1.642	25.12	0.00	C
ATOM	243	C	LEU	A	61	12.652	-1.918	0.150	25.12	0.00	C
ATOM	244	O	LEU	A	61	12.658	-0.852	-0.467	25.12	0.00	O
ATOM	245	N	HIS	A	62	12.390	-3.087	-0.427	66.36	0.00	N
ATOM	246	CA	HIS	A	62	12.070	-3.185	-1.846	66.36	0.00	C
ATOM	247	C	HIS	A	62	10.793	-2.426	-2.177	66.36	0.00	C
ATOM	248	O	HIS	A	62	10.763	-1.615	-3.103	66.36	0.00	O
ATOM	249	N	LEU	A	63	9.737	-2.694	-1.416	23.84	0.00	N
ATOM	250	CA	LEU	A	63	8.443	-2.066	-1.651	23.84	0.00	C
ATOM	251	C	LEU	A	63	8.500	-0.567	-1.380	23.84	0.00	C
ATOM	252	O	LEU	A	63	7.981	0.234	-2.157	23.84	0.00	O
ATOM	253	N	GLY	A	64	9.135	-0.196	-0.274	27.85	0.00	N
ATOM	254	CA	GLY	A	64	9.240	1.206	0.114	27.85	0.00	C
ATOM	255	C	GLY	A	64	10.021	2.006	-0.919	27.85	0.00	C
ATOM	256	O	GLY	A	64	9.630	3.116	-1.284	27.85	0.00	O
ATOM	257	N	LEU	A	65	11.125	1.438	-1.390	31.75	0.00	N
ATOM	258	CA	LEU	A	65	11.929	2.069	-2.430	31.75	0.00	C
ATOM	259	C	LEU	A	65	11.109	2.305	-3.692	31.75	0.00	C
ATOM	260	O	LEU	A	65	11.115	3.400	-4.253	31.75	0.00	O
ATOM	261	N	GLU	A	66	10.404	1.268	-4.135	78.61	0.00	N
ATOM	262	CA	GLU	A	66	9.573	1.362	-5.329	78.61	0.00	C
ATOM	263	C	GLU	A	66	8.424	2.341	-5.125	78.61	0.00	C
ATOM	264	O	GLU	A	66	8.013	3.034	-6.055	78.61	0.00	O
ATOM	265	N	SER	A	67	7.907	2.391	-3.902	40.41	0.00	N
ATOM	266	CA	SER	A	67	6.797	3.279	-3.575	40.41	0.00	C
ATOM	267	C	SER	A	67	7.189	4.739	-3.754	40.41	0.00	C
ATOM	268	O	SER	A	67	6.462	5.516	-4.374	40.41	0.00	O
ATOM	269	N	VAL	A	68	8.343	5.109	-3.209	37.35	0.00	N
ATOM	270	CA	VAL	A	68	8.839	6.476	-3.318	37.35	0.00	C
ATOM	271	C	VAL	A	68	9.177	6.825	-4.762	37.35	0.00	C
ATOM	272	O	VAL	A	68	8.937	7.945	-5.211	37.35	0.00	O
ATOM	273	N	LEU	A	69	9.735	5.860	-5.484	35.55	0.00	N
ATOM	274	CA	LEU	A	69	9.988	6.018	-6.912	35.55	0.00	C
ATOM	275	C	LEU	A	69	8.705	6.345	-7.665	35.55	0.00	C
ATOM	276	O	LEU	A	69	8.669	7.268	-8.480	35.55	0.00	O
ATOM	277	N	LYS	A	70	7.651	5.584	-7.388	19.55	0.00	N
ATOM	278	CA	LYS	A	70	6.366	5.786	-8.045	19.55	0.00	C
ATOM	279	C	LYS	A	70	5.783	7.152	-7.707	19.55	0.00	C
ATOM	280	O	LYS	A	70	5.176	7.805	-8.554	19.55	0.00	O
ATOM	281	N	GLY	A	71	5.971	7.578	-6.462	30.56	0.00	N
ATOM	282	CA	GLY	A	71	5.517	8.893	-6.026	30.56	0.00	C
ATOM	283	C	GLY	A	71	6.223	10.002	-6.796	30.56	0.00	C
ATOM	284	O	GLY	A	71	5.604	10.995	-7.182	30.56	0.00	O
ATOM	285	N	ASN	A	72	7.521	9.829	-7.017	33.38	0.00	N
ATOM	286	CA	ASN	A	72	8.312	10.810	-7.751	33.38	0.00	C
ATOM	287	C	ASN	A	72	8.022	10.746	-9.244	33.38	0.00	C
ATOM	288	O	ASN	A	72	8.169	11.737	-9.958	33.38	0.00	O
ATOM	289	N	PHE	A	73	7.610	9.572	-9.712	52.13	0.00	N
ATOM	290	CA	PHE	A	73	7.342	9.364	-11.130	52.13	0.00	C
ATOM	291	C	PHE	A	73	5.870	9.588	-11.450	52.13	0.00	C
ATOM	292	O	PHE	A	73	5.481	9.646	-12.617	52.13	0.00	O
ATOM	293	N	ASN	A	74	5.054	9.712	-10.408	42.19	0.00	N
ATOM	294	CA	ASN	A	74	3.611	9.835	-10.575	42.19	0.00	C
ATOM	295	C	ASN	A	74	3.023	8.587	-11.219	42.19	0.00	C
ATOM	296	O	ASN	A	74	2.076	8.669	-12.003	42.19	0.00	O
ATOM	297	N	ALA	A	75	3.588	7.432	-10.887	49.67	0.00	N
ATOM	298	CA	ALA	A	75	3.165	6.171	-11.484	49.67	0.00	C
ATOM	299	C	ALA	A	75	1.959	5.594	-10.754	49.67	0.00	C
ATOM	300	O	ALA	A	75	1.664	5.978	-9.623	49.67	0.00	O
ATOM	301	N	GLU	A	76	1.265	4.669	-11.408	45.22	0.00	N
ATOM	302	CA	GLU	A	76	0.058	4.076	-10.846	45.22	0.00	C
ATOM	303	C	GLU	A	76	-0.008	2.581	-11.133	45.22	0.00	C
ATOM	304	O	GLU	A	76	0.506	2.112	-12.149	45.22	0.00	O
ATOM	305	N	THR	A	77	-0.644	1.839	-10.233	63.11	0.00	N
ATOM	306	CA	THR	A	77	-0.790	0.398	-10.396	63.11	0.00	C
ATOM	307	C	THR	A	77	-2.251	-0.022	-10.300	63.11	0.00	C
ATOM	308	O	THR	A	77	-2.905	0.200	-9.280	63.11	0.00	O
ATOM	309	N	GLU	A	78	-2.759	-0.629	-11.366	62.28	0.00	N
ATOM	310	CA	GLU	A	78	-4.142	-1.090	-11.400	62.28	0.00	C
ATOM	311	C	GLU	A	78	-4.219	-2.609	-11.297	62.28	0.00	C
ATOM	312	O	GLU	A	78	-3.682	-3.326	-12.141	62.28	0.00	O
ATOM	313	N	VAL	A	79	-4.891	-3.092	-10.258	42.88	0.00	N
ATOM	314	CA	VAL	A	79	-5.038	-4.526	-10.041	42.88	0.00	C
ATOM	315	C	VAL	A	79	-6.505	-4.921	-9.941	42.88	0.00	C
ATOM	316	O	VAL	A	79	-7.300	-4.237	-9.295	42.88	0.00	O
ATOM	317	N	GLU	A	80	-6.860	-6.028	-10.584	42.38	0.00	N
ATOM	318	CA	GLU	A	80	-8.245	-6.484	-10.620	42.38	0.00	C
ATOM	319	C	GLU	A	80	-8.440	-7.723	-9.756	42.38	0.00	C
ATOM	320	O	GLU	A	80	-7.653	-8.667	-9.824	42.38	0.00	O
ATOM	321	N	THR	A	81	-9.491	-7.713	-8.944	41.50	0.00	N
ATOM	322	CA	THR	A	81	-9.765	-8.816	-8.031	41.50	0.00	C
ATOM	323	C	THR	A	81	-11.223	-8.818	-7.590	41.50	0.00	C
ATOM	324	O	THR	A	81	-12.017	-7.989	-8.036	41.50	0.00	O
ATOM	325	N	LEU	A	82	-11.569	-9.753	-6.711	42.94	0.00	N
ATOM	326	CA	LEU	A	82	-12.909	-9.808	-6.141	42.94	0.00	C
ATOM	327	C	LEU	A	82	-13.001	-8.976	-4.869	42.94	0.00	C
ATOM	328	O	LEU	A	82	-12.283	-9.223	-3.900	42.94	0.00	O

```
ATOM    329  N   ARG A  83     -13.889  -7.987  -4.878 22.34  0.00           N
ATOM    330  CA  ARG A  83     -14.107  -7.143  -3.709 22.34  0.00           C
ATOM    331  C   ARG A  83     -15.192  -7.718  -2.808 22.34  0.00           C
ATOM    332  O   ARG A  83     -16.372  -7.715  -3.161 22.34  0.00           O
ATOM    333  N   GLU A  84     -14.787  -8.211  -1.643 13.69  0.00           N
ATOM    334  CA  GLU A  84     -15.731  -8.731  -0.661 13.69  0.00           C
ATOM    335  C   GLU A  84     -15.988  -7.718   0.446 13.69  0.00           C
ATOM    336  O   GLU A  84     -15.325  -7.737   1.484 13.69  0.00           O
ATOM    337  N   ILE A  85     -16.954  -6.835   0.221 55.14  0.00           N
ATOM    338  CA  ILE A  85     -17.197  -5.717   1.126 55.14  0.00           C
ATOM    339  C   ILE A  85     -18.562  -5.834   1.792 55.14  0.00           C
ATOM    340  O   ILE A  85     -19.557  -6.151   1.139 55.14  0.00           O
ATOM    341  N   ASN A  86     -18.603  -5.578   3.095 49.91  0.00           N
ATOM    342  CA  ASN A  86     -19.861  -5.561   3.833 49.91  0.00           C
ATOM    343  C   ASN A  86     -20.424  -4.149   3.930 49.91  0.00           C
ATOM    344  O   ASN A  86     -19.934  -3.328   4.706 49.91  0.00           O
ATOM    345  N   VAL A  87     -21.454  -3.873   3.139 49.44  0.00           N
ATOM    346  CA  VAL A  87     -22.001  -2.524   3.036 49.44  0.00           C
ATOM    347  C   VAL A  87     -23.394  -2.447   3.649 49.44  0.00           C
ATOM    348  O   VAL A  87     -24.344  -3.032   3.131 49.44  0.00           O
ATOM    349  N   GLY A  88     -23.508  -1.722   4.757 48.77  0.00           N
ATOM    350  CA  GLY A  88     -24.801  -1.476   5.382 48.77  0.00           C
ATOM    351  C   GLY A  88     -25.332  -2.730   6.064 48.77  0.00           C
ATOM    352  O   GLY A  88     -26.524  -2.838   6.348 48.77  0.00           O
ATOM    353  N   GLY A  89     -24.437  -3.679   6.325 49.03  0.00           N
ATOM    354  CA  GLY A  89     -24.819  -4.942   6.945 49.03  0.00           C
ATOM    355  C   GLY A  89     -25.056  -6.021   5.896 49.03  0.00           C
ATOM    356  O   GLY A  89     -25.298  -7.181   6.230 49.03  0.00           O
ATOM    357  N   LYS A  90     -24.985  -5.632   4.628 49.16  0.00           N
ATOM    358  CA  LYS A  90     -25.193  -6.565   3.528 49.16  0.00           C
ATOM    359  C   LYS A  90     -23.876  -6.918   2.848 49.16  0.00           C
ATOM    360  O   LYS A  90     -23.041  -6.047   2.602 49.16  0.00           O
ATOM    361  N   VAL A  91     -23.697  -8.200   2.546 52.06  0.00           N
ATOM    362  CA  VAL A  91     -22.476  -8.671   1.903 52.06  0.00           C
ATOM    363  C   VAL A  91     -22.526  -8.449   0.397 52.06  0.00           C
ATOM    364  O   VAL A  91     -23.435  -8.929  -0.281 52.06  0.00           O
ATOM    365  N   TYR A  92     -21.543  -7.722  -0.122 47.93  0.00           N
ATOM    366  CA  TYR A  92     -21.409  -7.529  -1.562 47.93  0.00           C
ATOM    367  C   TYR A  92     -20.090  -8.094  -2.072 47.93  0.00           C
ATOM    368  O   TYR A  92     -19.025  -7.527  -1.828 47.93  0.00           O
ATOM    369  N   LYS A  93     -20.167  -9.215  -2.782 70.62  0.00           N
ATOM    370  CA  LYS A  93     -18.995  -9.797  -3.425 70.62  0.00           C
ATOM    371  C   LYS A  93     -18.956  -9.459  -4.909 70.62  0.00           C
ATOM    372  O   LYS A  93     -19.509 -10.184  -5.736 70.62  0.00           O
ATOM    373  N   ILE A  94     -18.298  -8.354  -5.242 47.02  0.00           N
ATOM    374  CA  ILE A  94     -18.385  -7.785  -6.582 47.02  0.00           C
ATOM    375  C   ILE A  94     -17.017  -7.741  -7.251 47.02  0.00           C
ATOM    376  O   ILE A  94     -16.045  -7.260  -6.669 47.02  0.00           O
ATOM    377  N   LYS A  95     -16.949  -8.247  -8.478 69.21  0.00           N
ATOM    378  CA  LYS A  95     -15.722  -8.185  -9.265 69.21  0.00           C
ATOM    379  C   LYS A  95     -15.409  -6.755  -9.684 69.21  0.00           C
ATOM    380  O   LYS A  95     -16.261  -6.058 -10.237 69.21  0.00           O
ATOM    381  N   GLY A  96     -14.182  -6.321  -9.418 48.31  0.00           N
ATOM    382  CA  GLY A  96     -13.750  -4.975  -9.774 48.31  0.00           C
ATOM    383  C   GLY A  96     -12.235  -4.843  -9.696 48.31  0.00           C
ATOM    384  O   GLY A  96     -11.514  -5.842  -9.691 48.31  0.00           O
ATOM    385  N   ARG A  97     -11.756  -3.606  -9.635 42.47  0.00           N
ATOM    386  CA  ARG A  97     -10.323  -3.336  -9.657 42.47  0.00           C
ATOM    387  C   ARG A  97      -9.993  -2.046  -8.918 42.47  0.00           C
ATOM    388  O   ARG A  97     -10.856  -1.188  -8.730 42.47  0.00           O
ATOM    389  N   ALA A  98      -8.740  -1.914  -8.498 45.19  0.00           N
ATOM    390  CA  ALA A  98      -8.279  -0.702  -7.832 45.19  0.00           C
ATOM    391  C   ALA A  98      -7.037  -0.138  -8.511 45.19  0.00           C
ATOM    392  O   ALA A  98      -6.023  -0.824  -8.641 45.19  0.00           O
ATOM    393  N   ASP A  99      -7.123   1.116  -8.943 66.23  0.00           N
ATOM    394  CA  ASP A  99      -5.987   1.795  -9.554 66.23  0.00           C
ATOM    395  C   ASP A  99      -5.373   2.809  -8.597 66.23  0.00           C
ATOM    396  O   ASP A  99      -5.882   3.920  -8.444 66.23  0.00           O
ATOM    397  N   ALA A 100      -4.276   2.421  -7.956 53.59  0.00           N
ATOM    398  CA  ALA A 100      -3.644   3.258  -6.944 53.59  0.00           C
ATOM    399  C   ALA A 100      -2.569   4.147  -7.557 53.59  0.00           C
ATOM    400  O   ALA A 100      -1.694   3.671  -8.280 53.59  0.00           O
ATOM    401  N   ILE A 101      -2.640   5.442  -7.262 57.33  0.00           N
ATOM    402  CA  ILE A 101      -1.630   6.387  -7.719 57.33  0.00           C
ATOM    403  C   ILE A 101      -1.213   7.332  -6.598 57.33  0.00           C
ATOM    404  O   ILE A 101      -2.058   7.888  -5.897 57.33  0.00           O
ATOM    405  N   ILE A 102       0.094   7.508  -6.436 55.03  0.00           N
ATOM    406  CA  ILE A 102       0.627   8.335  -5.360 55.03  0.00           C
ATOM    407  C   ILE A 102       0.665   9.804  -5.763 55.03  0.00           C
ATOM    408  O   ILE A 102       1.239  10.161  -6.792 55.03  0.00           O
ATOM    409  N   ARG A 103       0.052  10.653  -4.945 27.39  0.00           N
ATOM    410  CA  ARG A 103      -0.066  12.072  -5.261 27.39  0.00           C
ATOM    411  C   ARG A 103       0.943  12.897  -4.474 27.39  0.00           C
ATOM    412  O   ARG A 103       1.551  13.826  -5.008 27.39  0.00           O
ATOM    413  N   ASN A 104       1.118  12.554  -3.202 50.54  0.00           N
ATOM    414  CA  ASN A 104       2.040  13.277  -2.334 50.54  0.00           C
ATOM    415  C   ASN A 104       2.675  12.345  -1.310 50.54  0.00           C
ATOM    416  O   ASN A 104       2.086  12.056  -0.269 50.54  0.00           O
ATOM    417  N   ASP A 105       3.883  11.878  -1.610 48.89  0.00           N
ATOM    418  CA  ASP A 105       4.569  10.920  -0.753 48.89  0.00           C
ATOM    419  C   ASP A 105       5.257  11.619   0.414 48.89  0.00           C
ATOM    420  O   ASP A 105       5.922  10.978   1.229 48.89  0.00           O
```

ATOM	421	N	ASN	A	106	5.090	12.934	0.490	47.26	0.00	N
ATOM	422	CA	ASN	A	106	5.550	13.699	1.643	47.26	0.00	C
ATOM	423	C	ASN	A	106	4.401	13.994	2.600	47.26	0.00	C
ATOM	424	O	ASN	A	106	4.609	14.159	3.803	47.26	0.00	O
ATOM	425	N	GLY	A	107	3.190	14.061	2.060	52.85	0.00	N
ATOM	426	CA	GLY	A	107	1.990	14.185	2.880	52.85	0.00	C
ATOM	427	C	GLY	A	107	1.264	12.851	3.000	52.85	0.00	C
ATOM	428	O	GLY	A	107	0.224	12.756	3.651	52.85	0.00	O
ATOM	429	N	LYS	A	108	1.819	11.822	2.369	29.53	0.00	N
ATOM	430	CA	LYS	A	108	1.293	10.469	2.499	29.53	0.00	C
ATOM	431	C	LYS	A	108	-0.107	10.362	1.907	29.53	0.00	C
ATOM	432	O	LYS	A	108	-0.957	9.638	2.426	29.53	0.00	O
ATOM	433	N	SER	A	109	-0.341	11.090	0.820	43.88	0.00	N
ATOM	434	CA	SER	A	109	-1.633	11.066	0.146	43.88	0.00	C
ATOM	435	C	SER	A	109	-1.586	10.197	-1.105	43.88	0.00	C
ATOM	436	O	SER	A	109	-0.891	10.518	-2.068	43.88	0.00	O
ATOM	437	N	ILE	A	110	-2.331	9.097	-1.083	43.16	0.00	N
ATOM	438	CA	ILE	A	110	-2.419	8.209	-2.235	43.16	0.00	C
ATOM	439	C	ILE	A	110	-3.864	8.036	-2.689	43.16	0.00	C
ATOM	440	O	ILE	A	110	-4.726	7.638	-1.906	43.16	0.00	O
ATOM	441	N	VAL	A	111	-4.121	8.340	-3.957	55.63	0.00	N
ATOM	442	CA	VAL	A	111	-5.468	8.254	-4.507	55.63	0.00	C
ATOM	443	C	VAL	A	111	-5.687	6.928	-5.224	55.63	0.00	C
ATOM	444	O	VAL	A	111	-4.970	6.596	-6.167	55.63	0.00	O
ATOM	445	N	ILE	A	112	-6.682	6.173	-4.772	58.72	0.00	N
ATOM	446	CA	ILE	A	112	-6.992	4.878	-5.363	58.72	0.00	C
ATOM	447	C	ILE	A	112	-8.355	4.895	-6.043	58.72	0.00	C
ATOM	448	O	ILE	A	112	-9.388	5.001	-5.383	58.72	0.00	O
ATOM	449	N	GLU	A	113	-8.352	4.790	-7.368	83.18	0.00	N
ATOM	450	CA	GLU	A	113	-9.587	4.802	-8.141	83.18	0.00	C
ATOM	451	C	GLU	A	113	-10.228	3.421	-8.176	83.18	0.00	C
ATOM	452	O	GLU	A	113	-9.707	2.499	-8.804	83.18	0.00	O
ATOM	453	N	ILE	A	114	-11.362	3.283	-7.497	52.42	0.00	N
ATOM	454	CA	ILE	A	114	-12.054	2.003	-7.411	52.42	0.00	C
ATOM	455	C	ILE	A	114	-13.033	1.828	-8.565	52.42	0.00	C
ATOM	456	O	ILE	A	114	-13.987	2.593	-8.704	52.42	0.00	O
ATOM	457	N	LYS	A	115	-12.791	0.816	-9.391	70.74	0.00	N
ATOM	458	CA	LYS	A	115	-13.617	0.572	-10.568	70.74	0.00	C
ATOM	459	C	LYS	A	115	-14.397	-0.729	-10.433	70.74	0.00	C
ATOM	460	O	LYS	A	115	-13.958	-1.660	-9.757	70.74	0.00	O
ATOM	461	N	THR	A	116	-15.555	-0.789	-11.081	42.92	0.00	N
ATOM	462	CA	THR	A	116	-16.378	-1.993	-11.069	42.92	0.00	C
ATOM	463	C	THR	A	116	-16.338	-2.701	-12.418	42.92	0.00	C
ATOM	464	O	THR	A	116	-16.472	-2.069	-13.465	42.92	0.00	O
ATOM	465	N	SER	A	117	-16.154	-4.016	-12.384	45.57	0.00	N
ATOM	466	CA	SER	A	117	-16.085	-4.811	-13.604	45.57	0.00	C
ATOM	467	C	SER	A	117	-17.324	-5.682	-13.765	45.57	0.00	C
ATOM	468	O	SER	A	117	-17.806	-5.891	-14.878	45.57	0.00	O
ATOM	469	N	ARG	A	118	-17.835	-6.188	-12.648	29.06	0.00	N
ATOM	470	CA	ARG	A	118	-19.020	-7.036	-12.663	29.06	0.00	C
ATOM	471	C	ARG	A	118	-20.179	-6.353	-13.378	29.06	0.00	C
ATOM	472	O	ARG	A	118	-20.672	-5.318	-12.931	29.06	0.00	O
ATOM	473	N	SER	A	119	-20.610	-6.939	-14.489	43.52	0.00	N
ATOM	474	CA	SER	A	119	-21.662	-6.348	-15.308	43.52	0.00	C
ATOM	475	C	SER	A	119	-22.998	-6.354	-14.578	43.52	0.00	C
ATOM	476	O	SER	A	119	-23.907	-5.598	-14.920	43.52	0.00	O
ATOM	477	N	ASP	A	120	-23.112	-7.213	-13.571	45.20	0.00	N
ATOM	478	CA	ASP	A	120	-24.335	-7.313	-12.784	45.20	0.00	C
ATOM	479	C	ASP	A	120	-24.580	-6.040	-11.984	45.20	0.00	C
ATOM	480	O	ASP	A	120	-25.722	-5.703	-11.669	45.20	0.00	O
ATOM	481	N	LYS	A	121	-23.501	-5.334	-11.659	28.78	0.00	N
ATOM	482	CA	LYS	A	121	-23.579	-4.185	-10.766	28.78	0.00	C
ATOM	483	C	LYS	A	121	-23.308	-2.886	-11.514	28.78	0.00	C
ATOM	484	O	LYS	A	121	-22.497	-2.851	-12.440	28.78	0.00	O
ATOM	485	N	GLY	A	122	-23.990	-1.821	-11.109	50.28	0.00	N
ATOM	486	CA	GLY	A	122	-23.842	-0.523	-11.756	50.28	0.00	C
ATOM	487	C	GLY	A	122	-23.209	0.493	-10.815	50.28	0.00	C
ATOM	488	O	GLY	A	122	-22.815	0.159	-9.698	50.28	0.00	O
ATOM	489	N	LEU	A	123	-23.116	1.737	-11.273	74.40	0.00	N
ATOM	490	CA	LEU	A	123	-22.538	2.808	-10.468	74.40	0.00	C
ATOM	491	C	LEU	A	123	-23.474	4.008	-10.396	74.40	0.00	C
ATOM	492	O	LEU	A	123	-23.379	4.931	-11.204	74.40	0.00	O
ATOM	493	N	PRO	A	124	-24.377	3.990	-9.422	58.93	0.00	N
ATOM	494	CA	PRO	A	124	-25.310	5.092	-9.221	58.93	0.00	C
ATOM	495	C	PRO	A	124	-24.574	6.418	-9.082	58.93	0.00	C
ATOM	496	O	PRO	A	124	-23.563	6.508	-8.386	58.93	0.00	O
ATOM	497	N	LEU	A	125	-25.085	7.447	-9.750	43.87	0.00	N
ATOM	498	CA	LEU	A	125	-24.449	8.758	-9.742	43.87	0.00	C
ATOM	499	C	LEU	A	125	-24.468	9.371	-8.347	43.87	0.00	C
ATOM	500	O	LEU	A	125	-23.632	10.213	-8.018	43.87	0.00	O
ATOM	501	N	ILE	A	126	-25.426	8.943	-7.532	58.05	0.00	N
ATOM	502	CA	ILE	A	126	-25.542	9.434	-6.164	58.05	0.00	C
ATOM	503	C	ILE	A	126	-24.407	8.913	-5.293	58.05	0.00	C
ATOM	504	O	ILE	A	126	-24.210	9.376	-4.168	58.05	0.00	O
ATOM	505	N	HIS	A	127	-23.660	7.947	-5.817	43.35	0.00	N
ATOM	506	CA	HIS	A	127	-22.487	7.424	-5.126	43.35	0.00	C
ATOM	507	C	HIS	A	127	-21.213	7.711	-5.910	43.35	0.00	C
ATOM	508	O	HIS	A	127	-20.139	7.871	-5.329	43.35	0.00	O
ATOM	509	N	HIS	A	128	-21.338	7.775	-7.231	52.41	0.00	N
ATOM	510	CA	HIS	A	128	-20.196	8.042	-8.097	52.41	0.00	C
ATOM	511	C	HIS	A	128	-19.692	9.468	-7.919	52.41	0.00	C
ATOM	512	O	HIS	A	128	-18.485	9.711	-7.890	52.41	0.00	O

ATOM	513	N	LYS	A	129	-20.622	10.409	-7.799	88.91	0.00	N
ATOM	514	CA	LYS	A	129	-20.274	11.805	-7.565	88.91	0.00	C
ATOM	515	C	LYS	A	129	-19.535	11.975	-6.244	88.91	0.00	C
ATOM	516	O	LYS	A	129	-18.642	12.812	-6.124	88.91	0.00	O
ATOM	517	N	MET	A	130	-19.913	11.174	-5.253	78.04	0.00	N
ATOM	518	CA	MET	A	130	-19.219	11.161	-3.972	78.04	0.00	C
ATOM	519	C	MET	A	130	-17.787	10.663	-4.125	78.04	0.00	C
ATOM	520	O	MET	A	130	-16.852	11.258	-3.588	78.04	0.00	O
ATOM	521	N	GLN	A	131	-17.622	9.569	-4.861	58.11	0.00	N
ATOM	522	CA	GLN	A	131	-16.297	9.037	-5.155	58.11	0.00	C
ATOM	523	C	GLN	A	131	-15.412	10.091	-5.809	58.11	0.00	C
ATOM	524	O	GLN	A	131	-14.248	10.252	-5.444	58.11	0.00	O
ATOM	525	N	LEU	A	132	-15.972	10.808	-6.778	72.67	0.00	N
ATOM	526	CA	LEU	A	132	-15.223	11.820	-7.512	72.67	0.00	C
ATOM	527	C	LEU	A	132	-14.768	12.945	-6.591	72.67	0.00	C
ATOM	528	O	LEU	A	132	-13.606	13.350	-6.619	72.67	0.00	O
ATOM	529	N	GLN	A	133	-15.690	13.445	-5.775	99.99	0.00	N
ATOM	530	CA	GLN	A	133	-15.390	14.537	-4.859	99.99	0.00	C
ATOM	531	C	GLN	A	133	-14.294	14.147	-3.875	99.99	0.00	C
ATOM	532	O	GLN	A	133	-13.375	14.923	-3.615	99.99	0.00	O
ATOM	533	N	ILE	A	134	-14.398	12.939	-3.331	69.20	0.00	N
ATOM	534	CA	ILE	A	134	-13.425	12.449	-2.362	69.20	0.00	C
ATOM	535	C	ILE	A	134	-12.025	12.407	-2.961	69.20	0.00	C
ATOM	536	O	ILE	A	134	-11.074	12.928	-2.377	69.20	0.00	O
ATOM	537	N	TYR	A	135	-11.904	11.786	-4.129	53.52	0.00	N
ATOM	538	CA	TYR	A	135	-10.608	11.601	-4.769	53.52	0.00	C
ATOM	539	C	TYR	A	135	-9.911	12.937	-4.996	53.52	0.00	C
ATOM	540	O	TYR	A	135	-8.691	13.043	-4.861	53.52	0.00	O
ATOM	541	N	LEU	A	136	-10.691	13.955	-5.340	90.27	0.00	N
ATOM	542	CA	LEU	A	136	-10.149	15.286	-5.590	90.27	0.00	C
ATOM	543	C	LEU	A	136	-9.721	15.957	-4.292	90.27	0.00	C
ATOM	544	O	LEU	A	136	-8.885	16.861	-4.296	90.27	0.00	O
ATOM	545	N	TRP	A	137	-10.297	15.512	-3.181	77.33	0.00	N
ATOM	546	CA	TRP	A	137	-10.027	16.114	-1.882	77.33	0.00	C
ATOM	547	C	TRP	A	137	-8.845	15.441	-1.197	77.33	0.00	C
ATOM	548	O	TRP	A	137	-8.222	16.017	-0.305	77.33	0.00	O
ATOM	549	N	LEU	A	138	-8.540	14.219	-1.619	32.22	0.00	N
ATOM	550	CA	LEU	A	138	-7.532	13.406	-0.950	32.22	0.00	C
ATOM	551	C	LEU	A	138	-6.191	14.125	-0.895	32.22	0.00	C
ATOM	552	O	LEU	A	138	-5.507	14.106	0.128	32.22	0.00	O
ATOM	553	N	PHE	A	139	-5.821	14.762	-2.001	59.66	0.00	N
ATOM	554	CA	PHE	A	139	-4.550	15.470	-2.088	59.66	0.00	C
ATOM	555	C	PHE	A	139	-4.455	16.565	-1.033	59.66	0.00	C
ATOM	556	O	PHE	A	139	-3.522	16.589	-0.231	59.66	0.00	O
ATOM	557	N	SER	A	140	-5.427	17.471	-1.040	50.13	0.00	N
ATOM	558	CA	SER	A	140	-5.436	18.592	-0.108	50.13	0.00	C
ATOM	559	C	SER	A	140	-5.690	18.120	1.318	50.13	0.00	C
ATOM	560	O	SER	A	140	-5.347	18.807	2.280	50.13	0.00	O
ATOM	561	N	ALA	A	141	-6.294	16.943	1.448	49.30	0.00	N
ATOM	562	CA	ALA	A	141	-6.559	16.359	2.758	49.30	0.00	C
ATOM	563	C	ALA	A	141	-5.352	15.585	3.271	49.30	0.00	C
ATOM	564	O	ALA	A	141	-5.324	15.151	4.422	49.30	0.00	O
ATOM	565	N	GLU	A	142	-4.355	15.415	2.408	35.86	0.00	N
ATOM	566	CA	GLU	A	142	-3.127	14.724	2.785	35.86	0.00	C
ATOM	567	C	GLU	A	142	-3.420	13.318	3.291	35.86	0.00	C
ATOM	568	O	GLU	A	142	-2.924	12.911	4.342	35.86	0.00	O
ATOM	569	N	LYS	A	143	-4.226	12.579	2.537	43.24	0.00	N
ATOM	570	CA	LYS	A	143	-4.584	11.214	2.908	43.24	0.00	C
ATOM	571	C	LYS	A	143	-5.110	10.437	1.709	43.24	0.00	C
ATOM	572	O	LYS	A	143	-5.334	11.004	0.639	43.24	0.00	O
ATOM	573	N	GLY	A	144	-5.303	9.136	1.891	49.83	0.00	N
ATOM	574	CA	GLY	A	144	-5.952	8.308	0.882	49.83	0.00	C
ATOM	575	C	GLY	A	144	-7.239	7.692	1.417	49.83	0.00	C
ATOM	576	O	GLY	A	144	-7.380	7.475	2.620	49.83	0.00	O
ATOM	577	N	ILE	A	145	-8.174	7.414	0.515	38.62	0.00	N
ATOM	578	CA	ILE	A	145	-9.412	6.736	0.882	38.62	0.00	C
ATOM	579	C	ILE	A	145	-9.814	5.716	-0.176	38.62	0.00	C
ATOM	580	O	ILE	A	145	-9.766	5.997	-1.373	38.62	0.00	O
ATOM	581	N	LEU	A	146	-10.212	4.530	0.274	51.00	0.00	N
ATOM	582	CA	LEU	A	146	-10.783	3.525	-0.615	51.00	0.00	C
ATOM	583	C	LEU	A	146	-12.301	3.635	-0.668	51.00	0.00	C
ATOM	584	O	LEU	A	146	-12.991	3.311	0.299	51.00	0.00	O
ATOM	585	N	VAL	A	147	-12.817	4.096	-1.803	42.62	0.00	N
ATOM	586	CA	VAL	A	147	-14.248	4.334	-1.953	42.62	0.00	C
ATOM	587	C	VAL	A	147	-14.893	3.274	-2.836	42.62	0.00	C
ATOM	588	O	VAL	A	147	-14.583	3.168	-4.023	42.62	0.00	O
ATOM	589	N	TYR	A	148	-15.793	2.492	-2.250	47.38	0.00	N
ATOM	590	CA	TYR	A	148	-16.444	1.402	-2.968	47.38	0.00	C
ATOM	591	C	TYR	A	148	-17.920	1.697	-3.196	47.38	0.00	C
ATOM	592	O	TYR	A	148	-18.619	2.156	-2.292	47.38	0.00	O
ATOM	593	N	ILE	A	149	-18.391	1.432	-4.410	37.50	0.00	N
ATOM	594	CA	ILE	A	149	-19.755	1.774	-4.794	37.50	0.00	C
ATOM	595	C	ILE	A	149	-20.559	0.527	-5.137	37.50	0.00	C
ATOM	596	O	ILE	A	149	-20.159	-0.268	-5.988	37.50	0.00	O
ATOM	597	N	THR	A	150	-21.697	0.361	-4.471	44.28	0.00	N
ATOM	598	CA	THR	A	150	-22.661	-0.667	-4.844	44.28	0.00	C
ATOM	599	C	THR	A	150	-23.933	-0.049	-5.411	44.28	0.00	C
ATOM	600	O	THR	A	150	-24.183	1.144	-5.243	44.28	0.00	O
ATOM	601	N	PRO	A	151	-24.734	-0.868	-6.084	38.24	0.00	N
ATOM	602	CA	PRO	A	151	-25.966	-0.396	-6.705	38.24	0.00	C
ATOM	603	C	PRO	A	151	-26.876	0.274	-5.684	38.24	0.00	C
ATOM	604	O	PRO	A	151	-27.700	1.118	-6.033	38.24	0.00	O

```
ATOM    605  N   ASP A 152     -26.723  -0.108  -4.421 44.34  0.00           N
ATOM    606  CA  ASP A 152     -27.594   0.382  -3.361 44.34  0.00           C
ATOM    607  C   ASP A 152     -26.924   1.499  -2.569 44.34  0.00           C
ATOM    608  O   ASP A 152     -27.498   2.572  -2.386 44.34  0.00           O
ATOM    609  N   ARG A 153     -25.707   1.240  -2.104 47.43  0.00           N
ATOM    610  CA  ARG A 153     -25.077   2.083  -1.095 47.43  0.00           C
ATOM    611  C   ARG A 153     -23.600   2.295  -1.399 47.43  0.00           C
ATOM    612  O   ARG A 153     -23.081   1.785  -2.391 47.43  0.00           O
ATOM    613  N   ILE A 154     -22.927   3.052  -0.538 36.41  0.00           N
ATOM    614  CA  ILE A 154     -21.524   3.388  -0.748 36.41  0.00           C
ATOM    615  C   ILE A 154     -20.713   3.188   0.526 36.41  0.00           C
ATOM    616  O   ILE A 154     -21.144   3.571   1.614 36.41  0.00           O
ATOM    617  N   ALA A 155     -19.537   2.585   0.385 46.26  0.00           N
ATOM    618  CA  ALA A 155     -18.674   2.312   1.528 46.26  0.00           C
ATOM    619  C   ALA A 155     -17.390   3.127   1.454 46.26  0.00           C
ATOM    620  O   ALA A 155     -16.833   3.331   0.376 46.26  0.00           O
ATOM    621  N   GLU A 156     -16.922   3.592   2.608 38.25  0.00           N
ATOM    622  CA  GLU A 156     -15.736   4.438   2.670 38.25  0.00           C
ATOM    623  C   GLU A 156     -14.720   3.888   3.663 38.25  0.00           C
ATOM    624  O   GLU A 156     -14.979   3.833   4.865 38.25  0.00           O
ATOM    625  N   TYR A 157     -13.562   3.482   3.152 48.68  0.00           N
ATOM    626  CA  TYR A 157     -12.477   3.004   4.000 48.68  0.00           C
ATOM    627  C   TYR A 157     -11.297   3.966   3.978 48.68  0.00           C
ATOM    628  O   TYR A 157     -10.478   3.940   3.059 48.68  0.00           O
ATOM    629  N   GLU A 158     -11.214   4.816   4.997 43.72  0.00           N
ATOM    630  CA  GLU A 158     -10.190   5.853   5.049 43.72  0.00           C
ATOM    631  C   GLU A 158      -8.832   5.268   5.414 43.72  0.00           C
ATOM    632  O   GLU A 158      -8.739   4.351   6.229 43.72  0.00           O
ATOM    633  N   ILE A 159      -7.780   5.804   4.806 48.13  0.00           N
ATOM    634  CA  ILE A 159      -6.418   5.394   5.126 48.13  0.00           C
ATOM    635  C   ILE A 159      -5.567   6.589   5.540 48.13  0.00           C
ATOM    636  O   ILE A 159      -4.984   7.270   4.696 48.13  0.00           O
ATOM    637  N   ASN A 160      -5.499   6.838   6.843 47.40  0.00           N
ATOM    638  CA  ASN A 160      -4.645   7.891   7.379 47.40  0.00           C
ATOM    639  C   ASN A 160      -3.216   7.401   7.567 47.40  0.00           C
ATOM    640  O   ASN A 160      -2.960   6.197   7.584 47.40  0.00           O
ATOM    641  N   GLU A 161      -2.287   8.340   7.710 37.06  0.00           N
ATOM    642  CA  GLU A 161      -0.872   8.007   7.829 37.06  0.00           C
ATOM    643  C   GLU A 161      -0.635   7.006   8.952 37.06  0.00           C
ATOM    644  O   GLU A 161       0.008   5.976   8.752 37.06  0.00           O
ATOM    645  N   PRO A 162      -1.158   7.315  10.133 52.91  0.00           N
ATOM    646  CA  PRO A 162      -1.004   6.443  11.292 52.91  0.00           C
ATOM    647  C   PRO A 162      -1.816   5.164  11.129 52.91  0.00           C
ATOM    648  O   PRO A 162      -1.545   4.159  11.785 52.91  0.00           O
ATOM    649  N   LEU A 163      -2.814   5.211  10.254 61.33  0.00           N
ATOM    650  CA  LEU A 163      -3.684   4.064  10.022 61.33  0.00           C
ATOM    651  C   LEU A 163      -3.203   3.238   8.836 61.33  0.00           C
ATOM    652  O   LEU A 163      -3.698   2.138   8.591 61.33  0.00           O
ATOM    653  N   ASP A 164      -2.234   3.775   8.102 23.30  0.00           N
ATOM    654  CA  ASP A 164      -1.689   3.091   6.936 23.30  0.00           C
ATOM    655  C   ASP A 164      -0.917   1.842   7.341 23.30  0.00           C
ATOM    656  O   ASP A 164      -1.228   0.737   6.894 23.30  0.00           O
ATOM    657  N   GLU A 165       0.090   2.023   8.189 46.45  0.00           N
ATOM    658  CA  GLU A 165       0.911   0.911   8.650 46.45  0.00           C
ATOM    659  C   GLU A 165       0.094  -0.069   9.484 46.45  0.00           C
ATOM    660  O   GLU A 165       0.366  -1.270   9.491 46.45  0.00           O
ATOM    661  N   ALA A 166      -0.908   0.451  10.185 53.02  0.00           N
ATOM    662  CA  ALA A 166      -1.801  -0.384  10.978 53.02  0.00           C
ATOM    663  C   ALA A 166      -2.565  -1.366  10.099 53.02  0.00           C
ATOM    664  O   ALA A 166      -2.593  -2.567  10.372 53.02  0.00           O
ATOM    665  N   THR A 167      -3.182  -0.850   9.042 42.30  0.00           N
ATOM    666  CA  THR A 167      -3.903  -1.687   8.091 42.30  0.00           C
ATOM    667  C   THR A 167      -2.961  -2.645   7.374 42.30  0.00           C
ATOM    668  O   THR A 167      -3.296  -3.808   7.147 42.30  0.00           O
ATOM    669  N   ILE A 168      -1.780  -2.150   7.019 51.74  0.00           N
ATOM    670  CA  ILE A 168      -0.796  -2.953   6.305 51.74  0.00           C
ATOM    671  C   ILE A 168      -0.370  -4.163   7.128 51.74  0.00           C
ATOM    672  O   ILE A 168      -0.366  -5.291   6.637 51.74  0.00           O
ATOM    673  N   VAL A 169      -0.011  -3.919   8.385 43.45  0.00           N
ATOM    674  CA  VAL A 169       0.451  -4.982   9.269 43.45  0.00           C
ATOM    675  C   VAL A 169      -0.661  -5.984   9.554 43.45  0.00           C
ATOM    676  O   VAL A 169      -0.430  -7.193   9.580 43.45  0.00           O
ATOM    677  N   ARG A 170      -1.869  -5.473   9.770 50.30  0.00           N
ATOM    678  CA  ARG A 170      -3.019  -6.322  10.057 50.30  0.00           C
ATOM    679  C   ARG A 170      -3.308  -7.268   8.898 50.30  0.00           C
ATOM    680  O   ARG A 170      -3.557  -8.457   9.102 50.30  0.00           O
ATOM    681  N   LEU A 171      -3.274  -6.734   7.682 43.66  0.00           N
ATOM    682  CA  LEU A 171      -3.561  -7.523   6.490 43.66  0.00           C
ATOM    683  C   LEU A 171      -2.431  -8.500   6.191 43.66  0.00           C
ATOM    684  O   LEU A 171      -2.668  -9.612   5.720 43.66  0.00           O
ATOM    685  N   ALA A 172      -1.202  -8.076   6.465 49.64  0.00           N
ATOM    686  CA  ALA A 172      -0.037  -8.933   6.276 49.64  0.00           C
ATOM    687  C   ALA A 172      -0.113 -10.170   7.162 49.64  0.00           C
ATOM    688  O   ALA A 172       0.137 -11.286   6.710 49.64  0.00           O
ATOM    689  N   GLU A 173      -0.459  -9.963   8.429 48.78  0.00           N
ATOM    690  CA  GLU A 173      -0.573 -11.061   9.381 48.78  0.00           C
ATOM    691  C   GLU A 173      -1.742 -11.974   9.032 48.78  0.00           C
ATOM    692  O   GLU A 173      -1.639 -13.196   9.131 48.78  0.00           O
ATOM    693  N   ASP A 174      -2.853 -11.372   8.623 38.26  0.00           N
ATOM    694  CA  ASP A 174      -4.040 -12.130   8.244 38.26  0.00           C
ATOM    695  C   ASP A 174      -3.777 -12.982   7.009 38.26  0.00           C
ATOM    696  O   ASP A 174      -4.267 -14.108   6.904 38.26  0.00           O
```

```
ATOM    697  N   THR A 175      -3.001 -12.442   6.076 43.17  0.00           N
ATOM    698  CA  THR A 175      -2.617 -13.178   4.877 43.17  0.00           C
ATOM    699  C   THR A 175      -1.702 -14.348   5.219 43.17  0.00           C
ATOM    700  O   THR A 175      -1.824 -15.432   4.648 43.17  0.00           O
ATOM    701  N   ILE A 176      -0.785 -14.123   6.154 18.83  0.00           N
ATOM    702  CA  ILE A 176       0.078 -15.186   6.653 18.83  0.00           C
ATOM    703  C   ILE A 176      -0.736 -16.295   7.307 18.83  0.00           C
ATOM    704  O   ILE A 176      -0.441 -17.478   7.138 18.83  0.00           O
ATOM    705  N   MET A 177      -1.762 -15.905   8.056 33.37  0.00           N
ATOM    706  CA  MET A 177      -2.672 -16.865   8.668 33.37  0.00           C
ATOM    707  C   MET A 177      -3.453 -17.635   7.611 33.37  0.00           C
ATOM    708  O   MET A 177      -3.750 -18.817   7.783 33.37  0.00           O
ATOM    709  N   LEU A 178      -3.785 -16.956   6.518 30.21  0.00           N
ATOM    710  CA  LEU A 178      -4.422 -17.605   5.378 30.21  0.00           C
ATOM    711  C   LEU A 178      -3.493 -18.628   4.735 30.21  0.00           C
ATOM    712  O   LEU A 178      -3.928 -19.705   4.328 30.21  0.00           O
ATOM    713  N   GLN A 179      -2.213 -18.284   4.650 48.05  0.00           N
ATOM    714  CA  GLN A 179      -1.207 -19.202   4.128 48.05  0.00           C
ATOM    715  C   GLN A 179      -1.049 -20.417   5.034 48.05  0.00           C
ATOM    716  O   GLN A 179      -0.894 -21.542   4.558 48.05  0.00           O
ATOM    717  N   ASN A 180      -1.089 -20.184   6.342 39.77  0.00           N
ATOM    718  CA  ASN A 180      -0.967 -21.261   7.316 39.77  0.00           C
ATOM    719  C   ASN A 180      -2.208 -22.145   7.318 39.77  0.00           C
ATOM    720  O   ASN A 180      -2.112 -23.364   7.467 39.77  0.00           O
ATOM    721  N   SER A 181      -3.371 -21.525   7.152 58.29  0.00           N
ATOM    722  CA  SER A 181      -4.634 -22.252   7.157 58.29  0.00           C
ATOM    723  C   SER A 181      -5.577 -21.726   6.083 58.29  0.00           C
ATOM    724  O   SER A 181      -6.097 -20.615   6.189 58.29  0.00           O
ATOM    725  N   PRO A 182      -5.793 -22.529   5.047 63.56  0.00           N
ATOM    726  CA  PRO A 182      -6.665 -22.142   3.945 63.56  0.00           C
ATOM    727  C   PRO A 182      -8.071 -21.826   4.441 63.56  0.00           C
ATOM    728  O   PRO A 182      -8.808 -21.071   3.807 63.56  0.00           O
ATOM    729  N   ARG A 183      -8.437 -22.408   5.578 84.53  0.00           N
ATOM    730  CA  ARG A 183      -9.784 -22.261   6.115 84.53  0.00           C
ATOM    731  C   ARG A 183      -9.944 -20.931   6.841 84.53  0.00           C
ATOM    732  O   ARG A 183     -11.027 -20.605   7.329 84.53  0.00           O
ATOM    733  N   PHE A 184      -8.860 -20.165   6.910 54.72  0.00           N
ATOM    734  CA  PHE A 184      -8.908 -18.820   7.469 54.72  0.00           C
ATOM    735  C   PHE A 184      -9.933 -17.957   6.744 54.72  0.00           C
ATOM    736  O   PHE A 184      -9.987 -17.943   5.513 54.72  0.00           O
ATOM    737  N   ASN A 185     -10.744 -17.240   7.513 64.00  0.00           N
ATOM    738  CA  ASN A 185     -11.822 -16.436   6.948 64.00  0.00           C
ATOM    739  C   ASN A 185     -11.289 -15.143   6.346 64.00  0.00           C
ATOM    740  O   ASN A 185     -11.490 -14.061   6.897 64.00  0.00           O
ATOM    741  N   TRP A 186     -10.607 -15.261   5.212 35.33  0.00           N
ATOM    742  CA  TRP A 186     -10.074 -14.098   4.513 35.33  0.00           C
ATOM    743  C   TRP A 186     -11.179 -13.330   3.799 35.33  0.00           C
ATOM    744  O   TRP A 186     -11.593 -13.699   2.699 35.33  0.00           O
ATOM    745  N   GLU A 187     -11.652 -12.260   4.428 83.66  0.00           N
ATOM    746  CA  GLU A 187     -12.692 -11.424   3.843 83.66  0.00           C
ATOM    747  C   GLU A 187     -12.150 -10.047   3.478 83.66  0.00           C
ATOM    748  O   GLU A 187     -12.880  -9.055   3.507 83.66  0.00           O
ATOM    749  N   CYS A 188     -10.868  -9.993   3.136 55.50  0.00           N
ATOM    750  CA  CYS A 188     -10.201  -8.724   2.866 55.50  0.00           C
ATOM    751  C   CYS A 188      -9.600  -8.706   1.466 55.50  0.00           C
ATOM    752  O   CYS A 188      -8.540  -8.122   1.242 55.50  0.00           O
ATOM    753  N   LYS A 189     -10.284  -9.349   0.525  7.38  0.00           N
ATOM    754  CA  LYS A 189      -9.826  -9.397  -0.858  7.38  0.00           C
ATOM    755  C   LYS A 189      -9.871  -8.018  -1.502  7.38  0.00           C
ATOM    756  O   LYS A 189      -9.092  -7.720  -2.408  7.38  0.00           O
ATOM    757  N   TYR A 190     -10.785  -7.178  -1.028 22.21  0.00           N
ATOM    758  CA  TYR A 190     -11.014  -5.873  -1.634 22.21  0.00           C
ATOM    759  C   TYR A 190      -9.855  -4.925  -1.358 22.21  0.00           C
ATOM    760  O   TYR A 190      -9.667  -3.935  -2.065 22.21  0.00           O
ATOM    761  N   CYS A 191      -9.077  -5.235  -0.326 36.59  0.00           N
ATOM    762  CA  CYS A 191      -8.007  -4.351   0.120 36.59  0.00           C
ATOM    763  C   CYS A 191      -6.916  -4.232  -0.937 36.59  0.00           C
ATOM    764  O   CYS A 191      -6.617  -5.192  -1.646 36.59  0.00           O
ATOM    765  N   ILE A 192      -6.322  -3.047  -1.036 43.02  0.00           N
ATOM    766  CA  ILE A 192      -5.291  -2.788  -2.034 43.02  0.00           C
ATOM    767  C   ILE A 192      -3.972  -3.441  -1.644 43.02  0.00           C
ATOM    768  O   ILE A 192      -3.026  -3.471  -2.432 43.02  0.00           O
ATOM    769  N   PHE A 193      -3.913  -3.963  -0.424 49.91  0.00           N
ATOM    770  CA  PHE A 193      -2.723  -4.654   0.057 49.91  0.00           C
ATOM    771  C   PHE A 193      -2.884  -6.166  -0.045 49.91  0.00           C
ATOM    772  O   PHE A 193      -1.976  -6.922   0.300 49.91  0.00           O
ATOM    773  N   SER A 194      -4.046  -6.600  -0.522 41.94  0.00           N
ATOM    774  CA  SER A 194      -4.344  -8.023  -0.633 41.94  0.00           C
ATOM    775  C   SER A 194      -3.534  -8.671  -1.748 41.94  0.00           C
ATOM    776  O   SER A 194      -3.431  -9.895  -1.825 41.94  0.00           O
ATOM    777  N   VAL A 195      -2.958  -7.841  -2.612 53.52  0.00           N
ATOM    778  CA  VAL A 195      -2.196  -8.333  -3.755 53.52  0.00           C
ATOM    779  C   VAL A 195      -0.698  -8.192  -3.518 53.52  0.00           C
ATOM    780  O   VAL A 195       0.110  -8.493  -4.397 53.52  0.00           O
ATOM    781  N   ILE A 196      -0.333  -7.735  -2.326 37.19  0.00           N
ATOM    782  CA  ILE A 196       1.067  -7.506  -1.988 37.19  0.00           C
ATOM    783  C   ILE A 196       1.562  -8.525  -0.969 37.19  0.00           C
ATOM    784  O   ILE A 196       0.962  -8.698   0.091 37.19  0.00           O
ATOM    785  N   CYS A 197       2.660  -9.197  -1.299 45.29  0.00           N
ATOM    786  CA  CYS A 197       3.252 -10.182  -0.401 45.29  0.00           C
ATOM    787  C   CYS A 197       3.413  -9.620   1.005 45.29  0.00           C
ATOM    788  O   CYS A 197       3.892  -8.500   1.185 45.29  0.00           O
```

```
ATOM    789  N   PRO A 198       3.011 -10.404   1.999 31.76  0.00           N
ATOM    790  CA  PRO A 198       3.098  -9.980   3.392 31.76  0.00           C
ATOM    791  C   PRO A 198       4.512  -9.537   3.746 31.76  0.00           C
ATOM    792  O   PRO A 198       4.704  -8.625   4.550 31.76  0.00           O
ATOM    793  N   ALA A 199       5.499 -10.189   3.142 39.98  0.00           N
ATOM    794  CA  ALA A 199       6.898  -9.850   3.377 39.98  0.00           C
ATOM    795  C   ALA A 199       7.216  -8.447   2.878 39.98  0.00           C
ATOM    796  O   ALA A 199       7.970  -7.708   3.512 39.98  0.00           O
ATOM    797  N   LYS A 200       6.637  -8.083   1.739 26.21  0.00           N
ATOM    798  CA  LYS A 200       6.827  -6.754   1.173 26.21  0.00           C
ATOM    799  C   LYS A 200       6.074  -5.699   1.976 26.21  0.00           C
ATOM    800  O   LYS A 200       6.531  -4.565   2.110 26.21  0.00           O
ATOM    801  N   LEU A 201       4.919  -6.083   2.509 31.76  0.00           N
ATOM    802  CA  LEU A 201       4.111  -5.178   3.318 31.76  0.00           C
ATOM    803  C   LEU A 201       4.853  -4.757   4.580 31.76  0.00           C
ATOM    804  O   LEU A 201       5.399  -5.593   5.299 31.76  0.00           O
ATOM    805  N   THR A 202       4.870  -3.453   4.843 46.74  0.00           N
ATOM    806  CA  THR A 202       5.512  -2.923   6.038 46.74  0.00           C
ATOM    807  C   THR A 202       5.197  -3.777   7.260 46.74  0.00           C
ATOM    808  O   THR A 202       5.799  -4.797   7.449 46.74  0.00           O
TER     809      THR A 202
ENDMDL
END
REMARK   1 ***********************************************************************
REMARK   1 Start File DEC3_vs_NAT_dSi_colored.pdb
REMARK   1 The occupancy record was modified to represent dSi := Si(DEC)-Si(NAT).
REMARK   1 Occ=0.00 means dSi=-1.285567 kB; Occ=99.99 means dSi=1.380460 kB.
MODEL        0
ATOM      1  N   MET A   1      15.717  15.959  -1.793 40.19  0.00           N
ATOM      2  CA  MET A   1      17.073  15.585  -2.177 40.19  0.00           C
ATOM      3  C   MET A   1      17.455  14.228  -1.600 40.19  0.00           C
ATOM      4  O   MET A   1      17.587  13.247  -2.332 40.19  0.00           O
ATOM      5  N   ILE A   2      17.631  14.179  -0.284 42.66  0.00           N
ATOM      6  CA  ILE A   2      18.039  12.952   0.389 42.66  0.00           C
ATOM      7  C   ILE A   2      16.948  11.892   0.311 42.66  0.00           C
ATOM      8  O   ILE A   2      17.218  10.730   0.007 42.66  0.00           O
ATOM      9  N   THR A   3      15.714  12.299   0.588 29.88  0.00           N
ATOM     10  CA  THR A   3      14.570  11.399   0.496 29.88  0.00           C
ATOM     11  C   THR A   3      14.412  10.851  -0.917 29.88  0.00           C
ATOM     12  O   THR A   3      14.157   9.663  -1.107 29.88  0.00           O
ATOM     13  N   GLU A   4      14.567  11.725  -1.906 21.02  0.00           N
ATOM     14  CA  GLU A   4      14.482  11.323  -3.305 21.02  0.00           C
ATOM     15  C   GLU A   4      15.518  10.256  -3.635 21.02  0.00           C
ATOM     16  O   GLU A   4      15.204   9.246  -4.264 21.02  0.00           O
ATOM     17  N   PHE A   5      16.755  10.488  -3.207 47.14  0.00           N
ATOM     18  CA  PHE A   5      17.824   9.515  -3.394 47.14  0.00           C
ATOM     19  C   PHE A   5      17.471   8.178  -2.756 47.14  0.00           C
ATOM     20  O   PHE A   5      17.612   7.125  -3.378 47.14  0.00           O
ATOM     21  N   LEU A   6      17.011   8.226  -1.510 26.94  0.00           N
ATOM     22  CA  LEU A   6      16.722   7.014  -0.753 26.94  0.00           C
ATOM     23  C   LEU A   6      15.610   6.206  -1.410 26.94  0.00           C
ATOM     24  O   LEU A   6      15.634   4.975  -1.398 26.94  0.00           O
ATOM     25  N   LEU A   7      14.637   6.906  -1.983 35.53  0.00           N
ATOM     26  CA  LEU A   7      13.545   6.256  -2.699 35.53  0.00           C
ATOM     27  C   LEU A   7      14.036   5.624  -3.995 35.53  0.00           C
ATOM     28  O   LEU A   7      13.613   4.527  -4.362 35.53  0.00           O
ATOM     29  N   LYS A   8      14.931   6.323  -4.687 28.46  0.00           N
ATOM     30  CA  LYS A   8      15.507   5.815  -5.926 28.46  0.00           C
ATOM     31  C   LYS A   8      16.289   4.529  -5.683 28.46  0.00           C
ATOM     32  O   LYS A   8      16.499   3.737  -6.601 28.46  0.00           O
ATOM     33  N   LYS A   9      16.717   4.329  -4.442 81.62  0.00           N
ATOM     34  CA  LYS A   9      17.499   3.153  -4.082 81.62  0.00           C
ATOM     35  C   LYS A   9      16.639   2.115  -3.372 81.62  0.00           C
ATOM     36  O   LYS A   9      16.899   0.915  -3.458 81.62  0.00           O
ATOM     37  N   LYS A  10      15.614   2.585  -2.670 48.84  0.00           N
ATOM     38  CA  LYS A  10      14.723   1.700  -1.928 48.84  0.00           C
ATOM     39  C   LYS A  10      13.432   1.449  -2.696 48.84  0.00           C
ATOM     40  O   LYS A  10      13.126   0.314  -3.060 48.84  0.00           O
ATOM     41  N   LEU A  11      12.677   2.515  -2.938 40.34  0.00           N
ATOM     42  CA  LEU A  11      11.351   2.395  -3.532 40.34  0.00           C
ATOM     43  C   LEU A  11      11.410   1.677  -4.874 40.34  0.00           C
ATOM     44  O   LEU A  11      10.698   0.697  -5.096 40.34  0.00           O
ATOM     45  N   GLU A  12      12.263   2.169  -5.765 37.85  0.00           N
ATOM     46  CA  GLU A  12      12.368   1.616  -7.111 37.85  0.00           C
ATOM     47  C   GLU A  12      12.706   0.131  -7.071 37.85  0.00           C
ATOM     48  O   GLU A  12      12.113  -0.669  -7.794 37.85  0.00           O
ATOM     49  N   GLU A  13      13.660  -0.231  -6.220 55.83  0.00           N
ATOM     50  CA  GLU A  13      14.115  -1.614  -6.123 55.83  0.00           C
ATOM     51  C   GLU A  13      13.057  -2.498  -5.477 55.83  0.00           C
ATOM     52  O   GLU A  13      12.820  -3.622  -5.917 55.83  0.00           O
ATOM     53  N   HIS A  14      12.423  -1.983  -4.429 31.91  0.00           N
ATOM     54  CA  HIS A  14      11.494  -2.774  -3.631 31.91  0.00           C
ATOM     55  C   HIS A  14      10.225  -3.088  -4.413 31.91  0.00           C
ATOM     56  O   HIS A  14       9.590  -4.119  -4.196 31.91  0.00           O
ATOM     57  N   LEU A  15       9.860  -2.191  -5.324 27.01  0.00           N
ATOM     58  CA  LEU A  15       8.730  -2.421  -6.215 27.01  0.00           C
ATOM     59  C   LEU A  15       9.048  -3.500  -7.242 27.01  0.00           C
ATOM     60  O   LEU A  15       8.199  -4.326  -7.573 27.01  0.00           O
ATOM     61  N   SER A  16      10.279  -3.486  -7.745 43.85  0.00           N
ATOM     62  CA  SER A  16      10.703  -4.444  -8.758 43.85  0.00           C
ATOM     63  C   SER A  16      11.022  -5.798  -8.138 43.85  0.00           C
ATOM     64  O   SER A  16      11.035  -6.820  -8.825 43.85  0.00           O
```

```
ATOM     65  N   HIS A  17      11.279  -5.801  -6.834 38.37  0.00           N
ATOM     66  CA  HIS A  17      11.537  -7.037  -6.105 38.37  0.00           C
ATOM     67  C   HIS A  17      10.237  -7.741  -5.738 38.37  0.00           C
ATOM     68  O   HIS A  17       9.224  -7.094  -5.469 38.37  0.00           O
ATOM     69  N   VAL A  18      10.271  -9.069  -5.728 36.75  0.00           N
ATOM     70  CA  VAL A  18       9.073  -9.864  -5.485 36.75  0.00           C
ATOM     71  C   VAL A  18       8.917 -10.185  -4.005 36.75  0.00           C
ATOM     72  O   VAL A  18       7.844  -9.998  -3.429 36.75  0.00           O
ATOM     73  N   LYS A  19       9.991 -10.671  -3.392 47.79  0.00           N
ATOM     74  CA  LYS A  19       9.924 -11.212  -2.040 47.79  0.00           C
ATOM     75  C   LYS A  19      10.379 -10.184  -1.012 47.79  0.00           C
ATOM     76  O   LYS A  19      10.461 -10.478   0.180 47.79  0.00           O
ATOM     77  N   GLU A  20      10.677  -8.977  -1.481 14.56  0.00           N
ATOM     78  CA  GLU A  20      11.192  -7.924  -0.614 14.56  0.00           C
ATOM     79  C   GLU A  20      10.273  -7.693   0.578 14.56  0.00           C
ATOM     80  O   GLU A  20       9.141  -7.234   0.422 14.56  0.00           O
ATOM     81  N   GLU A  21      10.765  -8.013   1.769  5.07  0.00           N
ATOM     82  CA  GLU A  21      10.006  -7.800   2.995  5.07  0.00           C
ATOM     83  C   GLU A  21      10.454  -6.530   3.707  5.07  0.00           C
ATOM     84  O   GLU A  21       9.890  -6.150   4.733  5.07  0.00           O
ATOM     85  N   ASN A  22      11.473  -5.878   3.157 50.91  0.00           N
ATOM     86  CA  ASN A  22      12.082  -4.722   3.805 50.91  0.00           C
ATOM     87  C   ASN A  22      11.209  -3.483   3.655 50.91  0.00           C
ATOM     88  O   ASN A  22      10.835  -3.105   2.545 50.91  0.00           O
ATOM     89  N   THR A  23      10.886  -2.854   4.780 46.08  0.00           N
ATOM     90  CA  THR A  23      10.092  -1.631   4.774 46.08  0.00           C
ATOM     91  C   THR A  23      10.944  -0.423   4.404 46.08  0.00           C
ATOM     92  O   THR A  23      12.096  -0.313   4.823 46.08  0.00           O
ATOM     93  N   ILE A  24      10.370   0.481   3.617 51.79  0.00           N
ATOM     94  CA  ILE A  24      11.071   1.689   3.201 51.79  0.00           C
ATOM     95  C   ILE A  24      11.001   2.765   4.277 51.79  0.00           C
ATOM     96  O   ILE A  24       9.919   3.238   4.626 51.79  0.00           O
ATOM     97  N   TYR A  25      12.160   3.146   4.802 52.24  0.00           N
ATOM     98  CA  TYR A  25      12.256   4.292   5.699 52.24  0.00           C
ATOM     99  C   TYR A  25      13.173   5.365   5.127 52.24  0.00           C
ATOM    100  O   TYR A  25      14.305   5.082   4.733 52.24  0.00           O
ATOM    101  N   VAL A  26      12.679   6.597   5.084 68.16  0.00           N
ATOM    102  CA  VAL A  26      13.417   7.699   4.478 68.16  0.00           C
ATOM    103  C   VAL A  26      13.369   8.943   5.355 68.16  0.00           C
ATOM    104  O   VAL A  26      12.603   9.007   6.317 68.16  0.00           O
ATOM    105  N   THR A  27      14.191   9.931   5.017 51.67  0.00           N
ATOM    106  CA  THR A  27      14.295  11.146   5.815 51.67  0.00           C
ATOM    107  C   THR A  27      12.926  11.773   6.047 51.67  0.00           C
ATOM    108  O   THR A  27      12.628  12.248   7.142 51.67  0.00           O
ATOM    109  N   ASP A  28      12.098  11.773   5.008 63.07  0.00           N
ATOM    110  CA  ASP A  28      10.740  12.295   5.111 63.07  0.00           C
ATOM    111  C   ASP A  28       9.940  11.547   6.169 63.07  0.00           C
ATOM    112  O   ASP A  28       9.165  12.147   6.914 63.07  0.00           O
ATOM    113  N   LEU A  29      10.130  10.234   6.228 61.09  0.00           N
ATOM    114  CA  LEU A  29       9.417   9.399   7.187 61.09  0.00           C
ATOM    115  C   LEU A  29       9.989   9.559   8.589 61.09  0.00           C
ATOM    116  O   LEU A  29       9.325   9.252   9.581 61.09  0.00           O
ATOM    117  N   VAL A  30      11.223  10.043   8.667 62.86  0.00           N
ATOM    118  CA  VAL A  30      11.830  10.394   9.947 62.86  0.00           C
ATOM    119  C   VAL A  30      11.217  11.665  10.518 62.86  0.00           C
ATOM    120  O   VAL A  30      10.906  11.737  11.707 62.86  0.00           O
ATOM    121  N   ARG A  31      11.043  12.668   9.662 99.99  0.00           N
ATOM    122  CA  ARG A  31      10.462  13.938  10.080 99.99  0.00           C
ATOM    123  C   ARG A  31       8.951  13.831  10.229 99.99  0.00           C
ATOM    124  O   ARG A  31       8.327  14.635  10.920 99.99  0.00           O
ATOM    125  N   CYS A  32       8.367  12.832   9.575 60.45  0.00           N
ATOM    126  CA  CYS A  32       6.926  12.617   9.635 60.45  0.00           C
ATOM    127  C   CYS A  32       6.474  12.301  11.056 60.45  0.00           C
ATOM    128  O   CYS A  32       7.039  11.432  11.719 60.45  0.00           O
ATOM    129  N   PRO A  33       5.452  13.014  11.517 51.71  0.00           N
ATOM    130  CA  PRO A  33       4.979  12.874  12.889 51.71  0.00           C
ATOM    131  C   PRO A  33       4.308  11.524  13.106 51.71  0.00           C
ATOM    132  O   PRO A  33       4.182  11.057  14.237 51.71  0.00           O
ATOM    133  N   ARG A  34       3.879  10.901  12.013 62.60  0.00           N
ATOM    134  CA  ARG A  34       3.235   9.593  12.080 62.60  0.00           C
ATOM    135  C   ARG A  34       4.198   8.531  12.593 62.60  0.00           C
ATOM    136  O   ARG A  34       3.780   7.533  13.181 62.60  0.00           O
ATOM    137  N   ARG A  35       5.489   8.751  12.369 46.60  0.00           N
ATOM    138  CA  ARG A  35       6.523   7.897  12.942 46.60  0.00           C
ATOM    139  C   ARG A  35       6.502   7.955  14.464 46.60  0.00           C
ATOM    140  O   ARG A  35       6.350   6.931  15.131 46.60  0.00           O
ATOM    141  N   VAL A  36       6.658   9.156  15.008 66.80  0.00           N
ATOM    142  CA  VAL A  36       6.602   9.357  16.451 66.80  0.00           C
ATOM    143  C   VAL A  36       5.312   8.799  17.036 66.80  0.00           C
ATOM    144  O   VAL A  36       5.309   8.231  18.129 66.80  0.00           O
ATOM    145  N   ARG A  37       4.216   8.963  16.303 57.00  0.00           N
ATOM    146  CA  ARG A  37       2.920   8.452  16.736 57.00  0.00           C
ATOM    147  C   ARG A  37       2.989   6.960  17.032 57.00  0.00           C
ATOM    148  O   ARG A  37       2.729   6.528  18.155 57.00  0.00           O
ATOM    149  N   TYR A  38       3.339   6.175  16.018 57.26  0.00           N
ATOM    150  CA  TYR A  38       3.290   4.722  16.121 57.26  0.00           C
ATOM    151  C   TYR A  38       4.371   4.200  17.060 57.26  0.00           C
ATOM    152  O   TYR A  38       4.275   3.086  17.574 57.26  0.00           O
ATOM    153  N   GLU A  39       5.399   5.013  17.280 53.99  0.00           N
ATOM    154  CA  GLU A  39       6.439   4.687  18.247 53.99  0.00           C
ATOM    155  C   GLU A  39       5.926   4.818  19.675 53.99  0.00           C
ATOM    156  O   GLU A  39       6.235   3.992  20.534 53.99  0.00           O
```

ATOM	157	N	SER	A	40	5.142	5.861	19.923	43.00	0.00	N
ATOM	158	CA	SER	A	40	4.590	6.107	21.249	43.00	0.00	C
ATOM	159	C	SER	A	40	3.390	5.208	21.522	43.00	0.00	C
ATOM	160	O	SER	A	40	3.126	4.840	22.667	43.00	0.00	O
ATOM	161	N	GLU	A	41	2.666	4.859	20.465	45.51	0.00	N
ATOM	162	CA	GLU	A	41	1.539	3.941	20.578	45.51	0.00	C
ATOM	163	C	GLU	A	41	2.010	2.495	20.648	45.51	0.00	C
ATOM	164	O	GLU	A	41	1.448	1.684	21.387	45.51	0.00	O
ATOM	165	N	TYR	A	42	3.042	2.175	19.876	33.77	0.00	N
ATOM	166	CA	TYR	A	42	3.543	0.809	19.793	33.77	0.00	C
ATOM	167	C	TYR	A	42	5.049	0.759	20.023	33.77	0.00	C
ATOM	168	O	TYR	A	42	5.831	0.739	19.073	33.77	0.00	O
ATOM	169	N	LYS	A	43	5.448	0.740	21.290	72.52	0.00	N
ATOM	170	CA	LYS	A	43	6.860	0.797	21.648	72.52	0.00	C
ATOM	171	C	LYS	A	43	7.605	-0.435	21.154	72.52	0.00	C
ATOM	172	O	LYS	A	43	8.834	-0.442	21.079	72.52	0.00	O
ATOM	173	N	GLU	A	44	6.854	-1.479	20.817	49.66	0.00	N
ATOM	174	CA	GLU	A	44	7.442	-2.712	20.306	49.66	0.00	C
ATOM	175	C	GLU	A	44	7.858	-2.562	18.849	49.66	0.00	C
ATOM	176	O	GLU	A	44	8.681	-3.327	18.346	49.66	0.00	O
ATOM	177	N	LEU	A	45	7.284	-1.571	18.173	32.70	0.00	N
ATOM	178	CA	LEU	A	45	7.631	-1.287	16.787	32.70	0.00	C
ATOM	179	C	LEU	A	45	8.817	-0.335	16.701	32.70	0.00	C
ATOM	180	O	LEU	A	45	8.721	0.829	17.091	32.70	0.00	O
ATOM	181	N	ALA	A	46	9.935	-0.835	16.186	71.21	0.00	N
ATOM	182	CA	ALA	A	46	11.113	-0.005	15.961	71.21	0.00	C
ATOM	183	C	ALA	A	46	11.066	0.659	14.591	71.21	0.00	C
ATOM	184	O	ALA	A	46	10.107	0.484	13.838	71.21	0.00	O
ATOM	185	N	ILE	A	47	12.105	1.423	14.273	54.66	0.00	N
ATOM	186	CA	ILE	A	47	12.244	2.014	12.947	54.66	0.00	C
ATOM	187	C	ILE	A	47	12.897	1.040	11.975	54.66	0.00	C
ATOM	188	O	ILE	A	47	13.064	1.343	10.795	54.66	0.00	O
ATOM	189	N	SER	A	48	13.263	-0.135	12.480	68.54	0.00	N
ATOM	190	CA	SER	A	48	13.922	-1.147	11.663	68.54	0.00	C
ATOM	191	C	SER	A	48	13.028	-1.593	10.512	68.54	0.00	C
ATOM	192	O	SER	A	48	11.802	-1.554	10.615	68.54	0.00	O
ATOM	193	N	GLN	A	49	13.649	-2.016	9.417	57.91	0.00	N
ATOM	194	CA	GLN	A	49	12.917	-2.354	8.203	57.91	0.00	C
ATOM	195	C	GLN	A	49	12.108	-3.632	8.385	57.91	0.00	C
ATOM	196	O	GLN	A	49	11.270	-3.973	7.550	57.91	0.00	O
ATOM	197	N	VAL	A	50	12.367	-4.338	9.481	53.84	0.00	N
ATOM	198	CA	VAL	A	50	11.641	-5.564	9.790	53.84	0.00	C
ATOM	199	C	VAL	A	50	10.234	-5.262	10.286	53.84	0.00	C
ATOM	200	O	VAL	A	50	9.370	-6.138	10.306	53.84	0.00	O
ATOM	201	N	TYR	A	51	10.009	-4.015	10.689	36.40	0.00	N
ATOM	202	CA	TYR	A	51	8.685	-3.572	11.106	36.40	0.00	C
ATOM	203	C	TYR	A	51	8.016	-2.735	10.022	36.40	0.00	C
ATOM	204	O	TYR	A	51	8.683	-2.004	9.290	36.40	0.00	O
ATOM	205	N	ALA	A	52	6.696	-2.847	9.925	43.22	0.00	N
ATOM	206	CA	ALA	A	52	5.947	-2.179	8.867	43.22	0.00	C
ATOM	207	C	ALA	A	52	4.621	-1.639	9.388	43.22	0.00	C
ATOM	208	O	ALA	A	52	3.562	-2.210	9.128	43.22	0.00	O
ATOM	209	N	PRO	A	53	4.686	-0.534	10.124	57.51	0.00	N
ATOM	210	CA	PRO	A	53	3.485	0.166	10.563	57.51	0.00	C
ATOM	211	C	PRO	A	53	2.601	0.537	9.379	57.51	0.00	C
ATOM	212	O	PRO	A	53	3.090	0.990	8.344	57.51	0.00	O
ATOM	213	N	SER	A	54	1.296	0.341	9.537	25.24	0.00	N
ATOM	214	CA	SER	A	54	0.344	0.621	8.469	25.24	0.00	C
ATOM	215	C	SER	A	54	0.390	2.088	8.060	25.24	0.00	C
ATOM	216	O	SER	A	54	0.311	2.415	6.877	25.24	0.00	O
ATOM	217	N	ALA	A	55	0.519	2.968	9.047	34.28	0.00	N
ATOM	218	CA	ALA	A	55	0.611	4.400	8.789	34.28	0.00	C
ATOM	219	C	ALA	A	55	1.834	4.730	7.945	34.28	0.00	C
ATOM	220	O	ALA	A	55	1.754	5.514	6.998	34.28	0.00	O
ATOM	221	N	ILE	A	56	2.967	4.128	8.292	33.53	0.00	N
ATOM	222	CA	ILE	A	56	4.201	4.326	7.541	33.53	0.00	C
ATOM	223	C	ILE	A	56	4.067	3.812	6.114	33.53	0.00	C
ATOM	224	O	ILE	A	56	4.529	4.449	5.168	33.53	0.00	O
ATOM	225	N	LEU	A	57	3.430	2.655	5.965	18.72	0.00	N
ATOM	226	CA	LEU	A	57	3.215	2.062	4.651	18.72	0.00	C
ATOM	227	C	LEU	A	57	2.392	2.983	3.759	18.72	0.00	C
ATOM	228	O	LEU	A	57	2.704	3.164	2.582	18.72	0.00	O
ATOM	229	N	GLY	A	58	1.340	3.563	4.326	37.26	0.00	N
ATOM	230	CA	GLY	A	58	0.519	4.532	3.609	37.26	0.00	C
ATOM	231	C	GLY	A	58	1.338	5.748	3.197	37.26	0.00	C
ATOM	232	O	GLY	A	58	1.166	6.281	2.101	37.26	0.00	O
ATOM	233	N	ASP	A	59	2.229	6.183	4.082	27.71	0.00	N
ATOM	234	CA	ASP	A	59	3.118	7.301	3.787	27.71	0.00	C
ATOM	235	C	ASP	A	59	4.093	6.950	2.670	27.71	0.00	C
ATOM	236	O	ASP	A	59	4.476	7.808	1.876	27.71	0.00	O
ATOM	237	N	ILE	A	60	4.491	5.683	2.615	13.66	0.00	N
ATOM	238	CA	ILE	A	60	5.377	5.204	1.562	13.66	0.00	C
ATOM	239	C	ILE	A	60	4.706	5.288	0.197	13.66	0.00	C
ATOM	240	O	ILE	A	60	5.339	5.649	-0.796	13.66	0.00	O
ATOM	241	N	LEU	A	61	3.422	4.953	0.153	31.03	0.00	N
ATOM	242	CA	LEU	A	61	2.624	5.129	-1.056	31.03	0.00	C
ATOM	243	C	LEU	A	61	2.564	6.594	-1.467	31.03	0.00	C
ATOM	244	O	LEU	A	61	2.735	6.927	-2.640	31.03	0.00	O
ATOM	245	N	HIS	A	62	2.319	7.466	-0.495	54.69	0.00	N
ATOM	246	CA	HIS	A	62	2.326	8.905	-0.737	54.69	0.00	C
ATOM	247	C	HIS	A	62	3.643	9.352	-1.358	54.69	0.00	C
ATOM	248	O	HIS	A	62	3.659	10.143	-2.301	54.69	0.00	O

ATOM	249	N	LEU	A	63	4.747	8.840	-0.824	12.94	0.00	N
ATOM	250	CA	LEU	A	63	6.068	9.130	-1.371	12.94	0.00	C
ATOM	251	C	LEU	A	63	6.174	8.684	-2.823	12.94	0.00	C
ATOM	252	O	LEU	A	63	6.720	9.398	-3.664	12.94	0.00	O
ATOM	253	N	GLY	A	64	5.648	7.498	-3.112	20.96	0.00	N
ATOM	254	CA	GLY	A	64	5.639	6.975	-4.473	20.96	0.00	C
ATOM	255	C	GLY	A	64	4.808	7.857	-5.397	20.96	0.00	C
ATOM	256	O	GLY	A	64	5.144	8.035	-6.568	20.96	0.00	O
ATOM	257	N	LEU	A	65	3.723	8.406	-4.863	35.09	0.00	N
ATOM	258	CA	LEU	A	65	2.849	9.280	-5.636	35.09	0.00	C
ATOM	259	C	LEU	A	65	3.509	10.628	-5.898	35.09	0.00	C
ATOM	260	O	LEU	A	65	3.293	11.242	-6.943	35.09	0.00	O
ATOM	261	N	GLU	A	66	4.315	11.082	-4.945	79.25	0.00	N
ATOM	262	CA	GLU	A	66	5.106	12.294	-5.123	79.25	0.00	C
ATOM	263	C	GLU	A	66	6.216	12.081	-6.143	79.25	0.00	C
ATOM	264	O	GLU	A	66	6.584	13.001	-6.875	79.25	0.00	O
ATOM	265	N	SER	A	67	6.746	10.865	-6.188	33.56	0.00	N
ATOM	266	CA	SER	A	67	7.708	10.485	-7.217	33.56	0.00	C
ATOM	267	C	SER	A	67	7.071	10.507	-8.601	33.56	0.00	C
ATOM	268	O	SER	A	67	7.690	10.938	-9.573	33.56	0.00	O
ATOM	269	N	VAL	A	68	5.829	10.040	-8.683	22.61	0.00	N
ATOM	270	CA	VAL	A	68	5.062	10.116	-9.921	22.61	0.00	C
ATOM	271	C	VAL	A	68	4.839	11.562	-10.341	22.61	0.00	C
ATOM	272	O	VAL	A	68	4.997	11.911	-11.511	22.61	0.00	O
ATOM	273	N	LEU	A	69	4.467	12.402	-9.381	39.64	0.00	N
ATOM	274	CA	LEU	A	69	4.276	13.824	-9.638	39.64	0.00	C
ATOM	275	C	LEU	A	69	5.544	14.460	-10.194	39.64	0.00	C
ATOM	276	O	LEU	A	69	5.513	15.133	-11.224	39.64	0.00	O
ATOM	277	N	LYS	A	70	6.659	14.242	-9.505	56.21	0.00	N
ATOM	278	CA	LYS	A	70	7.921	14.876	-9.867	56.21	0.00	C
ATOM	279	C	LYS	A	70	8.454	14.329	-11.185	56.21	0.00	C
ATOM	280	O	LYS	A	70	9.086	15.049	-11.958	56.21	0.00	O
ATOM	281	N	GLY	A	71	8.194	13.050	-11.437	41.93	0.00	N
ATOM	282	CA	GLY	A	71	8.617	12.413	-12.678	41.93	0.00	C
ATOM	283	C	GLY	A	71	7.932	13.045	-13.883	41.93	0.00	C
ATOM	284	O	GLY	A	71	8.581	13.378	-14.875	41.93	0.00	O
ATOM	285	N	ASN	A	72	6.616	13.206	-13.792	45.44	0.00	N
ATOM	286	CA	ASN	A	72	5.841	13.803	-14.873	45.44	0.00	C
ATOM	287	C	ASN	A	72	4.498	14.316	-14.370	45.44	0.00	C
ATOM	288	O	ASN	A	72	3.549	13.548	-14.213	45.44	0.00	O
ATOM	289	N	PHE	A	73	4.424	15.618	-14.116	59.09	0.00	N
ATOM	290	CA	PHE	A	73	3.200	16.235	-13.619	59.09	0.00	C
ATOM	291	C	PHE	A	73	2.076	16.129	-14.642	59.09	0.00	C
ATOM	292	O	PHE	A	73	2.302	16.261	-15.844	59.09	0.00	O
ATOM	293	N	ASN	A	74	0.861	15.890	-14.156	50.33	0.00	N
ATOM	294	CA	ASN	A	74	-0.310	15.825	-15.021	50.33	0.00	C
ATOM	295	C	ASN	A	74	-0.133	14.773	-16.109	50.33	0.00	C
ATOM	296	O	ASN	A	74	-0.159	15.088	-17.299	50.33	0.00	O
ATOM	297	N	ALA	A	75	0.047	13.524	-15.695	51.14	0.00	N
ATOM	298	CA	ALA	A	75	0.305	12.436	-16.630	51.14	0.00	C
ATOM	299	C	ALA	A	75	-0.112	11.093	-16.043	51.14	0.00	C
ATOM	300	O	ALA	A	75	-0.453	11.000	-14.864	51.14	0.00	O
ATOM	301	N	GLU	A	76	-0.084	10.057	-16.872	46.53	0.00	N
ATOM	302	CA	GLU	A	76	-0.354	8.699	-16.412	46.53	0.00	C
ATOM	303	C	GLU	A	76	0.925	7.876	-16.344	46.53	0.00	C
ATOM	304	O	GLU	A	76	1.557	7.608	-17.366	46.53	0.00	O
ATOM	305	N	THR	A	77	1.302	7.476	-15.135	53.48	0.00	N
ATOM	306	CA	THR	A	77	2.521	6.703	-14.928	53.48	0.00	C
ATOM	307	C	THR	A	77	2.202	5.256	-14.577	53.48	0.00	C
ATOM	308	O	THR	A	77	1.539	4.980	-13.578	53.48	0.00	O
ATOM	309	N	GLU	A	78	2.679	4.332	-15.406	67.11	0.00	N
ATOM	310	CA	GLU	A	78	2.480	2.909	-15.163	67.11	0.00	C
ATOM	311	C	GLU	A	78	3.499	2.371	-14.167	67.11	0.00	C
ATOM	312	O	GLU	A	78	4.707	2.483	-14.379	67.11	0.00	O
ATOM	313	N	VAL	A	79	3.005	1.787	-13.081	53.82	0.00	N
ATOM	314	CA	VAL	A	79	3.872	1.281	-12.023	53.82	0.00	C
ATOM	315	C	VAL	A	79	3.622	-0.199	-11.767	53.82	0.00	C
ATOM	316	O	VAL	A	79	2.483	-0.621	-11.566	53.82	0.00	O
ATOM	317	N	GLU	A	80	4.693	-0.985	-11.777	38.92	0.00	N
ATOM	318	CA	GLU	A	80	4.592	-2.422	-11.550	38.92	0.00	C
ATOM	319	C	GLU	A	80	4.761	-2.760	-10.074	38.92	0.00	C
ATOM	320	O	GLU	A	80	5.670	-2.258	-9.413	38.92	0.00	O
ATOM	321	N	THR	A	81	3.881	-3.613	-9.563	38.45	0.00	N
ATOM	322	CA	THR	A	81	3.927	-4.014	-8.162	38.45	0.00	C
ATOM	323	C	THR	A	81	4.027	-5.528	-8.025	38.45	0.00	C
ATOM	324	O	THR	A	81	3.098	-6.256	-8.374	38.45	0.00	O
ATOM	325	N	LEU	A	82	5.161	-5.997	-7.515	48.62	0.00	N
ATOM	326	CA	LEU	A	82	5.367	-7.422	-7.287	48.62	0.00	C
ATOM	327	C	LEU	A	82	5.455	-7.735	-5.799	48.62	0.00	C
ATOM	328	O	LEU	A	82	6.493	-7.527	-5.171	48.62	0.00	O
ATOM	329	N	ARG	A	83	4.358	-8.235	-5.240	25.29	0.00	N
ATOM	330	CA	ARG	A	83	4.287	-8.516	-3.810	25.29	0.00	C
ATOM	331	C	ARG	A	83	3.930	-9.974	-3.553	25.29	0.00	C
ATOM	332	O	ARG	A	83	2.807	-10.404	-3.817	25.29	0.00	O
ATOM	333	N	GLU	A	84	4.892	-10.732	-3.037	20.97	0.00	N
ATOM	334	CA	GLU	A	84	4.627	-12.081	-2.551	20.97	0.00	C
ATOM	335	C	GLU	A	84	4.144	-12.062	-1.107	20.97	0.00	C
ATOM	336	O	GLU	A	84	4.927	-11.842	-0.183	20.97	0.00	O
ATOM	337	N	ILE	A	85	2.849	-12.293	-0.919	55.92	0.00	N
ATOM	338	CA	ILE	A	85	2.241	-12.213	0.403	55.92	0.00	C
ATOM	339	C	ILE	A	85	1.608	-13.540	0.800	55.92	0.00	C
ATOM	340	O	ILE	A	85	0.741	-14.060	0.099	55.92	0.00	O

```
ATOM    341  N   ASN A  86       2.048 -14.086   1.929 48.27  0.00           N
ATOM    342  CA  ASN A  86       1.504 -15.340   2.437 48.27  0.00           C
ATOM    343  C   ASN A  86       0.259 -15.097   3.282 48.27  0.00           C
ATOM    344  O   ASN A  86       0.339 -14.543   4.377 48.27  0.00           O
ATOM    345  N   VAL A  87      -0.890 -15.518   2.764 38.64  0.00           N
ATOM    346  CA  VAL A  87      -2.157 -15.335   3.463 38.64  0.00           C
ATOM    347  C   VAL A  87      -2.795 -16.675   3.806 38.64  0.00           C
ATOM    348  O   VAL A  87      -3.062 -17.490   2.924 38.64  0.00           O
ATOM    349  N   GLY A  88      -3.038 -16.895   5.094 47.04  0.00           N
ATOM    350  CA  GLY A  88      -3.772 -18.072   5.544 47.04  0.00           C
ATOM    351  C   GLY A  88      -2.921 -19.330   5.425 47.04  0.00           C
ATOM    352  O   GLY A  88      -3.439 -20.447   5.467 47.04  0.00           O
ATOM    353  N   GLY A  89      -1.614 -19.143   5.276 51.00  0.00           N
ATOM    354  CA  GLY A  89      -0.693 -20.262   5.128 51.00  0.00           C
ATOM    355  C   GLY A  89      -0.430 -20.570   3.660 51.00  0.00           C
ATOM    356  O   GLY A  89       0.303 -21.503   3.332 51.00  0.00           O
ATOM    357  N   LYS A  90      -1.034 -19.780   2.778 53.00  0.00           N
ATOM    358  CA  LYS A  90      -0.860 -19.962   1.342 53.00  0.00           C
ATOM    359  C   LYS A  90      -0.190 -18.749   0.710 53.00  0.00           C
ATOM    360  O   LYS A  90      -0.652 -17.619   0.869 53.00  0.00           O
ATOM    361  N   VAL A  91       0.900 -18.991  -0.009 47.74  0.00           N
ATOM    362  CA  VAL A  91       1.647 -17.916  -0.653 47.74  0.00           C
ATOM    363  C   VAL A  91       0.910 -17.392  -1.880 47.74  0.00           C
ATOM    364  O   VAL A  91       0.607 -18.149  -2.803 47.74  0.00           O
ATOM    365  N   TYR A  92       0.626 -16.094  -1.884 54.10  0.00           N
ATOM    366  CA  TYR A  92       0.053 -15.439  -3.053 54.10  0.00           C
ATOM    367  C   TYR A  92       1.056 -14.491  -3.699 54.10  0.00           C
ATOM    368  O   TYR A  92       1.328 -13.410  -3.177 54.10  0.00           O
ATOM    369  N   LYS A  93       1.603 -14.903  -4.838 52.22  0.00           N
ATOM    370  CA  LYS A  93       2.582 -14.093  -5.554 52.22  0.00           C
ATOM    371  C   LYS A  93       1.901 -13.024  -6.399 52.22  0.00           C
ATOM    372  O   LYS A  93       1.759 -13.176  -7.612 52.22  0.00           O
ATOM    373  N   ILE A  94       1.482 -11.943  -5.750 51.93  0.00           N
ATOM    374  CA  ILE A  94       0.730 -10.890  -6.422 51.93  0.00           C
ATOM    375  C   ILE A  94       1.610 -10.122  -7.399 51.93  0.00           C
ATOM    376  O   ILE A  94       2.356  -9.225  -7.006 51.93  0.00           O
ATOM    377  N   LYS A  95       1.519 -10.480  -8.676 70.68  0.00           N
ATOM    378  CA  LYS A  95       2.338  -9.854  -9.708 70.68  0.00           C
ATOM    379  C   LYS A  95       1.472  -9.172 -10.760 70.68  0.00           C
ATOM    380  O   LYS A  95       0.869  -9.834 -11.605 70.68  0.00           O
ATOM    381  N   GLY A  96       1.414  -7.846 -10.703 49.96  0.00           N
ATOM    382  CA  GLY A  96       0.594  -7.074 -11.630 49.96  0.00           C
ATOM    383  C   GLY A  96       1.035  -5.618 -11.674 49.96  0.00           C
ATOM    384  O   GLY A  96       2.092  -5.263 -11.151 49.96  0.00           O
ATOM    385  N   ARG A  97       0.219  -4.776 -12.300 30.60  0.00           N
ATOM    386  CA  ARG A  97       0.569  -3.374 -12.498 30.60  0.00           C
ATOM    387  C   ARG A  97      -0.585  -2.459 -12.116 30.60  0.00           C
ATOM    388  O   ARG A  97      -1.714  -2.913 -11.928 30.60  0.00           O
ATOM    389  N   ALA A  98      -0.297  -1.167 -12.001 43.89  0.00           N
ATOM    390  CA  ALA A  98      -1.333  -0.167 -11.780 43.89  0.00           C
ATOM    391  C   ALA A  98      -0.963   1.163 -12.424 43.89  0.00           C
ATOM    392  O   ALA A  98       0.200   1.566 -12.413 43.89  0.00           O
ATOM    393  N   ASP A  99      -1.958   1.841 -12.984 66.23  0.00           N
ATOM    394  CA  ASP A  99      -1.735   3.117 -13.653 66.23  0.00           C
ATOM    395  C   ASP A  99      -2.050   4.286 -12.729 66.23  0.00           C
ATOM    396  O   ASP A  99      -3.201   4.494 -12.344 66.23  0.00           O
ATOM    397  N   ALA A 100      -1.020   5.048 -12.377 61.99  0.00           N
ATOM    398  CA  ALA A 100      -1.193   6.233 -11.545 61.99  0.00           C
ATOM    399  C   ALA A 100      -1.529   7.455 -12.390 61.99  0.00           C
ATOM    400  O   ALA A 100      -0.658   8.030 -13.041 61.99  0.00           O
ATOM    401  N   ILE A 101      -2.799   7.846 -12.374 70.75  0.00           N
ATOM    402  CA  ILE A 101      -3.265   8.965 -13.186 70.75  0.00           C
ATOM    403  C   ILE A 101      -3.400  10.233 -12.351 70.75  0.00           C
ATOM    404  O   ILE A 101      -4.229  10.304 -11.444 70.75  0.00           O
ATOM    405  N   ILE A 102      -2.580  11.231 -12.663 38.77  0.00           N
ATOM    406  CA  ILE A 102      -2.625  12.506 -11.959 38.77  0.00           C
ATOM    407  C   ILE A 102      -3.844  13.321 -12.371 38.77  0.00           C
ATOM    408  O   ILE A 102      -4.045  13.602 -13.552 38.77  0.00           O
ATOM    409  N   ARG A 103      -4.655  13.700 -11.388 47.14  0.00           N
ATOM    410  CA  ARG A 103      -5.842  14.507 -11.644 47.14  0.00           C
ATOM    411  C   ARG A 103      -5.649  15.939 -11.159 47.14  0.00           C
ATOM    412  O   ARG A 103      -6.201  16.878 -11.732 47.14  0.00           O
ATOM    413  N   ASN A 104      -4.860  16.098 -10.101 50.55  0.00           N
ATOM    414  CA  ASN A 104      -4.531  17.421  -9.584 50.55  0.00           C
ATOM    415  C   ASN A 104      -3.099  17.469  -9.066 50.55  0.00           C
ATOM    416  O   ASN A 104      -2.836  17.137  -7.910 50.55  0.00           O
ATOM    417  N   ASP A 105      -2.177  17.884  -9.928 44.17  0.00           N
ATOM    418  CA  ASP A 105      -0.765  17.947  -9.567 44.17  0.00           C
ATOM    419  C   ASP A 105      -0.558  18.763  -8.298 44.17  0.00           C
ATOM    420  O   ASP A 105       0.086  18.306  -7.352 44.17  0.00           O
ATOM    421  N   ASN A 106      -1.105  19.973  -8.281 47.42  0.00           N
ATOM    422  CA  ASN A 106      -0.899  20.893  -7.169 47.42  0.00           C
ATOM    423  C   ASN A 106      -1.519  20.355  -5.887 47.42  0.00           C
ATOM    424  O   ASN A 106      -0.984  20.556  -4.796 47.42  0.00           O
ATOM    425  N   GLY A 107      -2.649  19.670  -6.024 50.23  0.00           N
ATOM    426  CA  GLY A 107      -3.347  19.104  -4.875 50.23  0.00           C
ATOM    427  C   GLY A 107      -2.827  17.710  -4.548 50.23  0.00           C
ATOM    428  O   GLY A 107      -3.282  17.076  -3.596 50.23  0.00           O
ATOM    429  N   LYS A 108      -1.873  17.238  -5.342 19.82  0.00           N
ATOM    430  CA  LYS A 108      -1.304  15.910  -5.150 19.82  0.00           C
ATOM    431  C   LYS A 108      -2.385  14.837  -5.182 19.82  0.00           C
ATOM    432  O   LYS A 108      -2.338  13.871  -4.421 19.82  0.00           O
```

ATOM	433	N	SER	A	109	-3.361	15.014	-6.068	46.23	0.00	N
ATOM	434	CA	SER	A	109	-4.454	14.059	-6.205	46.23	0.00	C
ATOM	435	C	SER	A	109	-4.203	13.097	-7.360	46.23	0.00	C
ATOM	436	O	SER	A	109	-4.251	13.487	-8.526	46.23	0.00	O
ATOM	437	N	ILE	A	110	-3.935	11.838	-7.027	49.84	0.00	N
ATOM	438	CA	ILE	A	110	-3.684	10.817	-8.036	49.84	0.00	C
ATOM	439	C	ILE	A	110	-4.602	9.616	-7.845	49.84	0.00	C
ATOM	440	O	ILE	A	110	-4.763	9.118	-6.732	49.84	0.00	O
ATOM	441	N	VAL	A	111	-5.202	9.157	-8.938	62.91	0.00	N
ATOM	442	CA	VAL	A	111	-6.023	7.953	-8.913	62.91	0.00	C
ATOM	443	C	VAL	A	111	-5.250	6.748	-9.433	62.91	0.00	C
ATOM	444	O	VAL	A	111	-4.755	6.755	-10.560	62.91	0.00	O
ATOM	445	N	ILE	A	112	-5.150	5.713	-8.605	58.29	0.00	N
ATOM	446	CA	ILE	A	112	-4.419	4.506	-8.973	58.29	0.00	C
ATOM	447	C	ILE	A	112	-5.357	3.446	-9.538	58.29	0.00	C
ATOM	448	O	ILE	A	112	-6.109	2.811	-8.799	58.29	0.00	O
ATOM	449	N	GLU	A	113	-5.306	3.259	-10.853	83.80	0.00	N
ATOM	450	CA	GLU	A	113	-6.138	2.263	-11.517	83.80	0.00	C
ATOM	451	C	GLU	A	113	-5.455	0.901	-11.539	83.80	0.00	C
ATOM	452	O	GLU	A	113	-4.482	0.696	-12.265	83.80	0.00	O
ATOM	453	N	ILE	A	114	-5.971	-0.027	-10.741	47.53	0.00	N
ATOM	454	CA	ILE	A	114	-5.400	-1.366	-10.653	47.53	0.00	C
ATOM	455	C	ILE	A	114	-5.764	-2.201	-11.874	47.53	0.00	C
ATOM	456	O	ILE	A	114	-6.933	-2.290	-12.251	47.53	0.00	O
ATOM	457	N	LYS	A	115	-4.758	-2.812	-12.488	61.71	0.00	N
ATOM	458	CA	LYS	A	115	-4.971	-3.651	-13.661	61.71	0.00	C
ATOM	459	C	LYS	A	115	-5.818	-4.871	-13.321	61.71	0.00	C
ATOM	460	O	LYS	A	115	-5.614	-5.512	-12.291	61.71	0.00	O
ATOM	461	N	THR	A	116	-6.771	-5.184	-14.191	35.95	0.00	N
ATOM	462	CA	THR	A	116	-7.696	-6.286	-13.951	35.95	0.00	C
ATOM	463	C	THR	A	116	-7.192	-7.576	-14.586	35.95	0.00	C
ATOM	464	O	THR	A	116	-6.188	-7.578	-15.299	35.95	0.00	O
ATOM	465	N	SER	A	117	-7.895	-8.673	-14.323	31.53	0.00	N
ATOM	466	CA	SER	A	117	-7.587	-9.950	-14.956	31.53	0.00	C
ATOM	467	C	SER	A	117	-7.885	-9.910	-16.449	31.53	0.00	C
ATOM	468	O	SER	A	117	-7.430	-10.768	-17.206	31.53	0.00	O
ATOM	469	N	ARG	A	118	-8.653	-8.910	-16.868	55.43	0.00	N
ATOM	470	CA	ARG	A	118	-8.954	-8.715	-18.281	55.43	0.00	C
ATOM	471	C	ARG	A	118	-7.875	-7.881	-18.964	55.43	0.00	C
ATOM	472	O	ARG	A	118	-7.416	-8.217	-20.055	55.43	0.00	O
ATOM	473	N	SER	A	119	-7.476	-6.794	-18.314	29.24	0.00	N
ATOM	474	CA	SER	A	119	-6.436	-5.922	-18.846	29.24	0.00	C
ATOM	475	C	SER	A	119	-5.054	-6.535	-18.657	29.24	0.00	C
ATOM	476	O	SER	A	119	-4.086	-6.115	-19.292	29.24	0.00	O
ATOM	477	N	ASP	A	120	-4.968	-7.529	-17.781	36.70	0.00	N
ATOM	478	CA	ASP	A	120	-3.713	-8.231	-17.539	36.70	0.00	C
ATOM	479	C	ASP	A	120	-3.953	-9.707	-17.251	36.70	0.00	C
ATOM	480	O	ASP	A	120	-4.281	-10.085	-16.126	36.70	0.00	O
ATOM	481	N	LYS	A	121	-3.787	-10.539	-18.274	43.60	0.00	N
ATOM	482	CA	LYS	A	121	-4.044	-11.968	-18.149	43.60	0.00	C
ATOM	483	C	LYS	A	121	-3.019	-12.635	-17.241	43.60	0.00	C
ATOM	484	O	LYS	A	121	-3.226	-13.754	-16.771	43.60	0.00	O
ATOM	485	N	GLY	A	122	-1.913	-11.941	-16.996	49.91	0.00	N
ATOM	486	CA	GLY	A	122	-0.859	-12.459	-16.132	49.91	0.00	C
ATOM	487	C	GLY	A	122	-1.167	-12.189	-14.665	49.91	0.00	C
ATOM	488	O	GLY	A	122	-0.437	-12.628	-13.776	49.91	0.00	O
ATOM	489	N	LEU	A	123	-2.252	-11.463	-14.417	46.62	0.00	N
ATOM	490	CA	LEU	A	123	-2.676	-11.158	-13.056	46.62	0.00	C
ATOM	491	C	LEU	A	123	-3.993	-11.845	-12.722	46.62	0.00	C
ATOM	492	O	LEU	A	123	-5.048	-11.467	-13.233	46.62	0.00	O
ATOM	493	N	PRO	A	124	-3.927	-12.856	-11.862	73.97	0.00	N
ATOM	494	CA	PRO	A	124	-5.121	-13.572	-11.427	73.97	0.00	C
ATOM	495	C	PRO	A	124	-6.149	-12.618	-10.833	73.97	0.00	C
ATOM	496	O	PRO	A	124	-5.795	-11.589	-10.255	73.97	0.00	O
ATOM	497	N	LEU	A	125	-7.423	-12.964	-10.978	23.98	0.00	N
ATOM	498	CA	LEU	A	125	-8.505	-12.146	-10.443	23.98	0.00	C
ATOM	499	C	LEU	A	125	-8.359	-11.955	-8.939	23.98	0.00	C
ATOM	500	O	LEU	A	125	-8.610	-10.869	-8.414	23.98	0.00	O
ATOM	501	N	ILE	A	126	-7.953	-13.015	-8.250	38.08	0.00	N
ATOM	502	CA	ILE	A	126	-7.707	-12.947	-6.814	38.08	0.00	C
ATOM	503	C	ILE	A	126	-6.616	-11.936	-6.488	38.08	0.00	C
ATOM	504	O	ILE	A	126	-6.794	-11.070	-5.632	38.08	0.00	O
ATOM	505	N	HIS	A	127	-5.486	-12.052	-7.177	29.29	0.00	N
ATOM	506	CA	HIS	A	127	-4.350	-11.168	-6.938	29.29	0.00	C
ATOM	507	C	HIS	A	127	-4.694	-9.724	-7.278	29.29	0.00	C
ATOM	508	O	HIS	A	127	-4.193	-8.792	-6.650	29.29	0.00	O
ATOM	509	N	HIS	A	128	-5.554	-9.544	-8.276	39.69	0.00	N
ATOM	510	CA	HIS	A	128	-6.082	-8.226	-8.603	39.69	0.00	C
ATOM	511	C	HIS	A	128	-6.847	-7.631	-7.428	39.69	0.00	C
ATOM	512	O	HIS	A	128	-6.621	-6.482	-7.046	39.69	0.00	O
ATOM	513	N	LYS	A	129	-7.751	-8.419	-6.857	27.03	0.00	N
ATOM	514	CA	LYS	A	129	-8.598	-7.949	-5.766	27.03	0.00	C
ATOM	515	C	LYS	A	129	-7.821	-7.876	-4.458	27.03	0.00	C
ATOM	516	O	LYS	A	129	-8.223	-7.182	-3.525	27.03	0.00	O
ATOM	517	N	MET	A	130	-6.707	-8.598	-4.395	40.21	0.00	N
ATOM	518	CA	MET	A	130	-5.795	-8.502	-3.263	40.21	0.00	C
ATOM	519	C	MET	A	130	-4.957	-7.232	-3.336	40.21	0.00	C
ATOM	520	O	MET	A	130	-4.637	-6.629	-2.311	40.21	0.00	O
ATOM	521	N	GLN	A	131	-4.602	-6.832	-4.552	46.26	0.00	N
ATOM	522	CA	GLN	A	131	-3.996	-5.527	-4.782	46.26	0.00	C
ATOM	523	C	GLN	A	131	-4.937	-4.401	-4.373	46.26	0.00	C
ATOM	524	O	GLN	A	131	-4.509	-3.392	-3.813	46.26	0.00	O

```
ATOM    525  N   LEU A 132      -6.224  -4.580  -4.658 48.70  0.00           N
ATOM    526  CA  LEU A 132      -7.241  -3.626  -4.236 48.70  0.00           C
ATOM    527  C   LEU A 132      -7.300  -3.518  -2.717 48.70  0.00           C
ATOM    528  O   LEU A 132      -7.461  -2.428  -2.168 48.70  0.00           O
ATOM    529  N   GLN A 133      -7.166  -4.656  -2.044 49.66  0.00           N
ATOM    530  CA  GLN A 133      -7.084  -4.678  -0.588 49.66  0.00           C
ATOM    531  C   GLN A 133      -5.851  -3.932  -0.094 49.66  0.00           C
ATOM    532  O   GLN A 133      -5.914  -3.189   0.884 49.66  0.00           O
ATOM    533  N   ILE A 134      -4.730  -4.137  -0.777 54.32  0.00           N
ATOM    534  CA  ILE A 134      -3.472  -3.510  -0.389 54.32  0.00           C
ATOM    535  C   ILE A 134      -3.555  -1.993  -0.501 54.32  0.00           C
ATOM    536  O   ILE A 134      -3.204  -1.272   0.433 54.32  0.00           O
ATOM    537  N   TYR A 135      -4.024  -1.513  -1.648 55.17  0.00           N
ATOM    538  CA  TYR A 135      -3.996  -0.088  -1.952 55.17  0.00           C
ATOM    539  C   TYR A 135      -5.095   0.656  -1.203 55.17  0.00           C
ATOM    540  O   TYR A 135      -4.933   1.822  -0.843 55.17  0.00           O
ATOM    541  N   LEU A 136      -6.212  -0.025  -0.973 51.61  0.00           N
ATOM    542  CA  LEU A 136      -7.276   0.510  -0.132 51.61  0.00           C
ATOM    543  C   LEU A 136      -6.812   0.668   1.310 51.61  0.00           C
ATOM    544  O   LEU A 136      -7.070   1.689   1.948 51.61  0.00           O
ATOM    545  N   TRP A 137      -6.126  -0.350   1.820 40.90  0.00           N
ATOM    546  CA  TRP A 137      -5.485  -0.263   3.127 40.90  0.00           C
ATOM    547  C   TRP A 137      -4.538   0.927   3.198 40.90  0.00           C
ATOM    548  O   TRP A 137      -4.556   1.691   4.165 40.90  0.00           O
ATOM    549  N   LEU A 138      -3.709   1.081   2.171 23.53  0.00           N
ATOM    550  CA  LEU A 138      -2.773   2.196   2.101 23.53  0.00           C
ATOM    551  C   LEU A 138      -3.509   3.528   2.030 23.53  0.00           C
ATOM    552  O   LEU A 138      -3.069   4.523   2.606 23.53  0.00           O
ATOM    553  N   PHE A 139      -4.632   3.541   1.320 31.22  0.00           N
ATOM    554  CA  PHE A 139      -5.432   4.751   1.173 31.22  0.00           C
ATOM    555  C   PHE A 139      -5.988   5.210   2.515 31.22  0.00           C
ATOM    556  O   PHE A 139      -5.977   6.401   2.828 31.22  0.00           O
ATOM    557  N   SER A 140      -6.475   4.259   3.304 30.40  0.00           N
ATOM    558  CA  SER A 140      -7.013   4.561   4.625 30.40  0.00           C
ATOM    559  C   SER A 140      -5.910   4.979   5.588 30.40  0.00           C
ATOM    560  O   SER A 140      -6.141   5.757   6.513 30.40  0.00           O
ATOM    561  N   ALA A 141      -4.709   4.457   5.365 38.58  0.00           N
ATOM    562  CA  ALA A 141      -3.557   4.810   6.185 38.58  0.00           C
ATOM    563  C   ALA A 141      -2.949   6.134   5.741 38.58  0.00           C
ATOM    564  O   ALA A 141      -2.288   6.817   6.523 38.58  0.00           O
ATOM    565  N   GLU A 142      -3.177   6.491   4.482  0.00  0.00           N
ATOM    566  CA  GLU A 142      -2.580   7.690   3.906  0.00  0.00           C
ATOM    567  C   GLU A 142      -3.406   8.927   4.233  0.00  0.00           C
ATOM    568  O   GLU A 142      -2.943   9.827   4.935  0.00  0.00           O
ATOM    569  N   LYS A 143      -4.630   8.969   3.719 66.08  0.00           N
ATOM    570  CA  LYS A 143      -5.468  10.157   3.829 66.08  0.00           C
ATOM    571  C   LYS A 143      -6.839   9.814   4.394 66.08  0.00           C
ATOM    572  O   LYS A 143      -7.461  10.627   5.077 66.08  0.00           O
ATOM    573  N   GLY A 144      -7.307   8.604   4.107 60.83  0.00           N
ATOM    574  CA  GLY A 144      -8.645   8.184   4.507 60.83  0.00           C
ATOM    575  C   GLY A 144      -9.616   8.251   3.335 60.83  0.00           C
ATOM    576  O   GLY A 144     -10.832   8.203   3.521 60.83  0.00           O
ATOM    577  N   ILE A 145      -9.072   8.362   2.128 72.37  0.00           N
ATOM    578  CA  ILE A 145      -9.889   8.417   0.922 72.37  0.00           C
ATOM    579  C   ILE A 145      -9.722   7.157   0.084 72.37  0.00           C
ATOM    580  O   ILE A 145      -8.659   6.917  -0.490 72.37  0.00           O
ATOM    581  N   LEU A 146     -10.778   6.353   0.016 57.82  0.00           N
ATOM    582  CA  LEU A 146     -10.707   5.048  -0.631 57.82  0.00           C
ATOM    583  C   LEU A 146     -11.049   5.147  -2.111 57.82  0.00           C
ATOM    584  O   LEU A 146     -11.937   5.905  -2.504 57.82  0.00           O
ATOM    585  N   VAL A 147     -10.340   4.377  -2.930 49.59  0.00           N
ATOM    586  CA  VAL A 147     -10.628   4.307  -4.357 49.59  0.00           C
ATOM    587  C   VAL A 147     -10.805   2.864  -4.813 49.59  0.00           C
ATOM    588  O   VAL A 147      -9.866   2.070  -4.772 49.59  0.00           O
ATOM    589  N   TYR A 148     -12.016   2.531  -5.248 53.71  0.00           N
ATOM    590  CA  TYR A 148     -12.314   1.188  -5.730 53.71  0.00           C
ATOM    591  C   TYR A 148     -12.243   1.119  -7.251 53.71  0.00           C
ATOM    592  O   TYR A 148     -12.922   1.874  -7.947 53.71  0.00           O
ATOM    593  N   ILE A 149     -11.418   0.210  -7.758 38.41  0.00           N
ATOM    594  CA  ILE A 149     -11.216   0.078  -9.196 38.41  0.00           C
ATOM    595  C   ILE A 149     -11.879  -1.184  -9.732 38.41  0.00           C
ATOM    596  O   ILE A 149     -11.446  -2.297  -9.438 38.41  0.00           O
ATOM    597  N   THR A 150     -12.934  -1.003 -10.520 57.09  0.00           N
ATOM    598  CA  THR A 150     -13.520  -2.101 -11.280 57.09  0.00           C
ATOM    599  C   THR A 150     -13.205  -1.971 -12.765 57.09  0.00           C
ATOM    600  O   THR A 150     -12.769  -0.918 -13.227 57.09  0.00           O
ATOM    601  N   PRO A 151     -13.430  -3.050 -13.508 48.53  0.00           N
ATOM    602  CA  PRO A 151     -13.038  -3.109 -14.911 48.53  0.00           C
ATOM    603  C   PRO A 151     -13.660  -1.969 -15.704 48.53  0.00           C
ATOM    604  O   PRO A 151     -13.090  -1.502 -16.691 48.53  0.00           O
ATOM    605  N   ASP A 152     -14.834  -1.522 -15.269 47.48  0.00           N
ATOM    606  CA  ASP A 152     -15.635  -0.585 -16.048 47.48  0.00           C
ATOM    607  C   ASP A 152     -16.265   0.476 -15.154 47.48  0.00           C
ATOM    608  O   ASP A 152     -17.316   1.028 -15.476 47.48  0.00           O
ATOM    609  N   ARG A 153     -15.615   0.756 -14.030 75.55  0.00           N
ATOM    610  CA  ARG A 153     -16.069   1.806 -13.125 75.55  0.00           C
ATOM    611  C   ARG A 153     -15.009   2.127 -12.079 75.55  0.00           C
ATOM    612  O   ARG A 153     -14.325   1.234 -11.578 75.55  0.00           O
ATOM    613  N   ILE A 154     -14.878   3.408 -11.751 47.28  0.00           N
ATOM    614  CA  ILE A 154     -14.048   3.831 -10.630 47.28  0.00           C
ATOM    615  C   ILE A 154     -14.870   4.577  -9.586 47.28  0.00           C
ATOM    616  O   ILE A 154     -15.444   5.628  -9.870 47.28  0.00           O
```

ATOM	617	N	ALA	A	155	-14.922	4.027	-8.378	49.85	0.00	N
ATOM	618	CA	ALA	A	155	-15.759	4.579	-7.320	49.85	0.00	C
ATOM	619	C	ALA	A	155	-14.914	5.091	-6.159	49.85	0.00	C
ATOM	620	O	ALA	A	155	-13.872	4.519	-5.839	49.85	0.00	O
ATOM	621	N	GLU	A	156	-15.371	6.170	-5.533	57.54	0.00	N
ATOM	622	CA	GLU	A	156	-14.655	6.763	-4.410	57.54	0.00	C
ATOM	623	C	GLU	A	156	-15.462	6.656	-3.123	57.54	0.00	C
ATOM	624	O	GLU	A	156	-16.678	6.849	-3.123	57.54	0.00	O
ATOM	625	N	TYR	A	157	-14.778	6.347	-2.026	56.62	0.00	N
ATOM	626	CA	TYR	A	157	-15.425	6.247	-0.723	56.62	0.00	C
ATOM	627	C	TYR	A	157	-14.594	6.926	0.358	56.62	0.00	C
ATOM	628	O	TYR	A	157	-13.409	7.200	0.164	56.62	0.00	O
ATOM	629	N	GLU	A	158	-15.222	7.196	1.498	49.97	0.00	N
ATOM	630	CA	GLU	A	158	-14.514	7.739	2.651	49.97	0.00	C
ATOM	631	C	GLU	A	158	-14.480	6.737	3.798	49.97	0.00	C
ATOM	632	O	GLU	A	158	-15.430	5.980	4.003	49.97	0.00	O
ATOM	633	N	ILE	A	159	-13.381	6.735	4.544	52.35	0.00	N
ATOM	634	CA	ILE	A	159	-13.235	5.849	5.693	52.35	0.00	C
ATOM	635	C	ILE	A	159	-12.746	6.611	6.918	52.35	0.00	C
ATOM	636	O	ILE	A	159	-11.827	7.425	6.828	52.35	0.00	O
ATOM	637	N	ASN	A	160	-13.365	6.343	8.061	52.16	0.00	N
ATOM	638	CA	ASN	A	160	-13.024	7.033	9.299	52.16	0.00	C
ATOM	639	C	ASN	A	160	-11.744	6.474	9.907	52.16	0.00	C
ATOM	640	O	ASN	A	160	-11.449	5.286	9.771	52.16	0.00	O
ATOM	641	N	GLU	A	161	-10.987	7.336	10.577	24.33	0.00	N
ATOM	642	CA	GLU	A	161	-9.660	6.974	11.061	24.33	0.00	C
ATOM	643	C	GLU	A	161	-9.720	5.763	11.982	24.33	0.00	C
ATOM	644	O	GLU	A	161	-8.898	4.852	11.881	24.33	0.00	O
ATOM	645	N	PRO	A	162	-10.698	5.759	12.882	48.62	0.00	N
ATOM	646	CA	PRO	A	162	-10.862	4.663	13.830	48.62	0.00	C
ATOM	647	C	PRO	A	162	-10.874	3.316	13.119	48.62	0.00	C
ATOM	648	O	PRO	A	162	-10.347	2.328	13.631	48.62	0.00	O
ATOM	649	N	LEU	A	163	-11.479	3.283	11.937	50.64	0.00	N
ATOM	650	CA	LEU	A	163	-11.574	2.054	11.158	50.64	0.00	C
ATOM	651	C	LEU	A	163	-10.206	1.618	10.648	50.64	0.00	C
ATOM	652	O	LEU	A	163	-9.923	0.425	10.545	50.64	0.00	O
ATOM	653	N	ASP	A	164	-9.361	2.593	10.329	13.03	0.00	N
ATOM	654	CA	ASP	A	164	-7.975	2.319	9.973	13.03	0.00	C
ATOM	655	C	ASP	A	164	-7.171	1.875	11.189	13.03	0.00	C
ATOM	656	O	ASP	A	164	-6.316	0.995	11.092	13.03	0.00	O
ATOM	657	N	GLU	A	165	-7.450	2.491	12.332	52.58	0.00	N
ATOM	658	CA	GLU	A	165	-6.737	2.177	13.565	52.58	0.00	C
ATOM	659	C	GLU	A	165	-6.906	0.711	13.941	52.58	0.00	C
ATOM	660	O	GLU	A	165	-6.002	0.099	14.510	52.58	0.00	O
ATOM	661	N	ALA	A	166	-8.067	0.152	13.618	52.45	0.00	N
ATOM	662	CA	ALA	A	166	-8.308	-1.274	13.801	52.45	0.00	C
ATOM	663	C	ALA	A	166	-7.261	-2.108	13.073	52.45	0.00	C
ATOM	664	O	ALA	A	166	-6.753	-3.093	13.610	52.45	0.00	O
ATOM	665	N	THR	A	167	-6.943	-1.708	11.847	22.42	0.00	N
ATOM	666	CA	THR	A	167	-6.001	-2.452	11.018	22.42	0.00	C
ATOM	667	C	THR	A	167	-4.561	-2.108	11.374	22.42	0.00	C
ATOM	668	O	THR	A	167	-3.650	-2.906	11.156	22.42	0.00	O
ATOM	669	N	ILE	A	168	-4.362	-0.915	11.926	46.72	0.00	N
ATOM	670	CA	ILE	A	168	-3.056	-0.515	12.434	46.72	0.00	C
ATOM	671	C	ILE	A	168	-2.629	-1.390	13.605	46.72	0.00	C
ATOM	672	O	ILE	A	168	-1.479	-1.824	13.681	46.72	0.00	O
ATOM	673	N	VAL	A	169	-3.560	-1.644	14.518	48.67	0.00	N
ATOM	674	CA	VAL	A	169	-3.302	-2.522	15.653	48.67	0.00	C
ATOM	675	C	VAL	A	169	-3.029	-3.949	15.196	48.67	0.00	C
ATOM	676	O	VAL	A	169	-2.095	-4.595	15.670	48.67	0.00	O
ATOM	677	N	ARG	A	170	-3.851	-4.435	14.270	4.85	0.00	N
ATOM	678	CA	ARG	A	170	-3.708	-5.793	13.757	4.85	0.00	C
ATOM	679	C	ARG	A	170	-2.342	-5.998	13.117	4.85	0.00	C
ATOM	680	O	ARG	A	170	-1.647	-6.970	13.413	4.85	0.00	O
ATOM	681	N	LEU	A	171	-1.961	-5.078	12.238	35.06	0.00	N
ATOM	682	CA	LEU	A	171	-0.685	-5.169	11.537	35.06	0.00	C
ATOM	683	C	LEU	A	171	0.484	-5.004	12.498	35.06	0.00	C
ATOM	684	O	LEU	A	171	1.510	-5.672	12.366	35.06	0.00	O
ATOM	685	N	ALA	A	172	0.324	-4.109	13.468	35.22	0.00	N
ATOM	686	CA	ALA	A	172	1.321	-3.930	14.518	35.22	0.00	C
ATOM	687	C	ALA	A	172	1.656	-5.255	15.190	35.22	0.00	C
ATOM	688	O	ALA	A	172	2.826	-5.600	15.354	35.22	0.00	O
ATOM	689	N	GLU	A	173	0.623	-5.995	15.577	57.67	0.00	N
ATOM	690	CA	GLU	A	173	0.805	-7.298	16.205	57.67	0.00	C
ATOM	691	C	GLU	A	173	1.409	-8.300	15.228	57.67	0.00	C
ATOM	692	O	GLU	A	173	2.216	-9.146	15.613	57.67	0.00	O
ATOM	693	N	ASP	A	174	1.013	-8.199	13.965	41.81	0.00	N
ATOM	694	CA	ASP	A	174	1.501	-9.107	12.933	41.81	0.00	C
ATOM	695	C	ASP	A	174	2.994	-8.921	12.697	41.81	0.00	C
ATOM	696	O	ASP	A	174	3.723	-9.886	12.469	41.81	0.00	O
ATOM	697	N	THR	A	175	3.445	-7.671	12.754	37.60	0.00	N
ATOM	698	CA	THR	A	175	4.844	-7.349	12.496	37.60	0.00	C
ATOM	699	C	THR	A	175	5.701	-7.588	13.732	37.60	0.00	C
ATOM	700	O	THR	A	175	6.905	-7.828	13.629	37.60	0.00	O
ATOM	701	N	ILE	A	176	5.075	-7.521	14.902	46.10	0.00	N
ATOM	702	CA	ILE	A	176	5.733	-7.901	16.146	46.10	0.00	C
ATOM	703	C	ILE	A	176	5.959	-9.406	16.212	46.10	0.00	C
ATOM	704	O	ILE	A	176	7.042	-9.865	16.574	46.10	0.00	O
ATOM	705	N	MET	A	177	4.930	-10.170	15.860	53.53	0.00	N
ATOM	706	CA	MET	A	177	5.016	-11.625	15.875	53.53	0.00	C
ATOM	707	C	MET	A	177	5.862	-12.140	14.718	53.53	0.00	C
ATOM	708	O	MET	A	177	6.651	-13.072	14.878	53.53	0.00	O

ATOM	709	N	LEU	A	178	5.695	-11.527	13.551	54.52	0.00	N
ATOM	710	CA	LEU	A	178	6.406	-11.954	12.351	54.52	0.00	C
ATOM	711	C	LEU	A	178	7.177	-10.798	11.728	54.52	0.00	C
ATOM	712	O	LEU	A	178	6.830	-10.318	10.649	54.52	0.00	O
ATOM	713	N	GLN	A	179	8.226	-10.355	12.413	9.65	0.00	N
ATOM	714	CA	GLN	A	179	9.062	-9.268	11.916	9.65	0.00	C
ATOM	715	C	GLN	A	179	9.892	-9.715	10.719	9.65	0.00	C
ATOM	716	O	GLN	A	179	10.498	-8.895	10.031	9.65	0.00	O
ATOM	717	N	ASN	A	180	9.914	-11.021	10.476	36.56	0.00	N
ATOM	718	CA	ASN	A	180	10.691	-11.582	9.376	36.56	0.00	C
ATOM	719	C	ASN	A	180	9.958	-11.429	8.051	36.56	0.00	C
ATOM	720	O	ASN	A	180	10.571	-11.466	6.983	36.56	0.00	O
ATOM	721	N	SER	A	181	8.642	-11.257	8.123	54.59	0.00	N
ATOM	722	CA	SER	A	181	7.818	-11.128	6.927	54.59	0.00	C
ATOM	723	C	SER	A	181	6.642	-10.191	7.166	54.59	0.00	C
ATOM	724	O	SER	A	181	5.487	-10.617	7.165	54.59	0.00	O
ATOM	725	N	PRO	A	182	6.942	-8.914	7.373	58.07	0.00	N
ATOM	726	CA	PRO	A	182	5.913	-7.881	7.411	58.07	0.00	C
ATOM	727	C	PRO	A	182	5.234	-7.730	6.055	58.07	0.00	C
ATOM	728	O	PRO	A	182	5.900	-7.619	5.025	58.07	0.00	O
ATOM	729	N	ARG	A	183	3.905	-7.728	6.061	45.05	0.00	N
ATOM	730	CA	ARG	A	183	3.133	-7.788	4.826	45.05	0.00	C
ATOM	731	C	ARG	A	183	1.671	-7.439	5.073	45.05	0.00	C
ATOM	732	O	ARG	A	183	1.268	-7.175	6.206	45.05	0.00	O
ATOM	733	N	PHE	A	184	0.879	-7.441	4.006	40.86	0.00	N
ATOM	734	CA	PHE	A	184	-0.495	-6.955	4.070	40.86	0.00	C
ATOM	735	C	PHE	A	184	-1.470	-8.096	4.329	40.86	0.00	C
ATOM	736	O	PHE	A	184	-2.602	-8.078	3.847	40.86	0.00	O
ATOM	737	N	ASN	A	185	-1.024	-9.088	5.092	41.19	0.00	N
ATOM	738	CA	ASN	A	185	-1.880	-10.204	5.476	41.19	0.00	C
ATOM	739	C	ASN	A	185	-2.866	-9.793	6.562	41.19	0.00	C
ATOM	740	O	ASN	A	185	-2.470	-9.429	7.670	41.19	0.00	O
ATOM	741	N	TRP	A	186	-4.153	-9.853	6.238	25.29	0.00	N
ATOM	742	CA	TRP	A	186	-5.201	-9.543	7.204	25.29	0.00	C
ATOM	743	C	TRP	A	186	-6.470	-10.334	6.912	25.29	0.00	C
ATOM	744	O	TRP	A	186	-6.625	-10.901	5.831	25.29	0.00	O
ATOM	745	N	GLU	A	187	-7.376	-10.369	7.884	93.17	0.00	N
ATOM	746	CA	GLU	A	187	-8.559	-11.217	7.798	93.17	0.00	C
ATOM	747	C	GLU	A	187	-9.701	-10.498	7.092	93.17	0.00	C
ATOM	748	O	GLU	A	187	-9.628	-9.296	6.836	93.17	0.00	O
ATOM	749	N	CYS	A	188	-10.758	-11.241	6.780	43.08	0.00	N
ATOM	750	CA	CYS	A	188	-11.922	-10.674	6.109	43.08	0.00	C
ATOM	751	C	CYS	A	188	-12.572	-9.587	6.957	43.08	0.00	C
ATOM	752	O	CYS	A	188	-13.252	-8.703	6.436	43.08	0.00	O
ATOM	753	N	LYS	A	189	-12.356	-9.658	8.266	45.12	0.00	N
ATOM	754	CA	LYS	A	189	-12.901	-8.666	9.186	45.12	0.00	C
ATOM	755	C	LYS	A	189	-12.206	-7.321	9.021	45.12	0.00	C
ATOM	756	O	LYS	A	189	-12.710	-6.291	9.470	45.12	0.00	O
ATOM	757	N	TYR	A	190	-11.045	-7.335	8.374	37.47	0.00	N
ATOM	758	CA	TYR	A	190	-10.256	-6.124	8.190	37.47	0.00	C
ATOM	759	C	TYR	A	190	-10.228	-5.702	6.727	37.47	0.00	C
ATOM	760	O	TYR	A	190	-10.025	-4.529	6.412	37.47	0.00	O
ATOM	761	N	CYS	A	191	-10.432	-6.665	5.835	32.14	0.00	N
ATOM	762	CA	CYS	A	191	-10.428	-6.396	4.402	32.14	0.00	C
ATOM	763	C	CYS	A	191	-11.374	-5.253	4.054	32.14	0.00	C
ATOM	764	O	CYS	A	191	-12.595	-5.407	4.106	32.14	0.00	O
ATOM	765	N	ILE	A	192	-10.804	-4.107	3.700	21.11	0.00	N
ATOM	766	CA	ILE	A	192	-11.596	-2.938	3.334	21.11	0.00	C
ATOM	767	C	ILE	A	192	-12.373	-3.181	2.047	21.11	0.00	C
ATOM	768	O	ILE	A	192	-13.530	-2.778	1.922	21.11	0.00	O
ATOM	769	N	PHE	A	193	-11.731	-3.840	1.089	33.12	0.00	N
ATOM	770	CA	PHE	A	193	-12.351	-4.114	-0.202	33.12	0.00	C
ATOM	771	C	PHE	A	193	-13.673	-4.851	-0.033	33.12	0.00	C
ATOM	772	O	PHE	A	193	-14.658	-4.540	-0.704	33.12	0.00	O
ATOM	773	N	SER	A	194	-13.689	-5.829	0.866	47.00	0.00	N
ATOM	774	CA	SER	A	194	-14.839	-6.712	1.017	47.00	0.00	C
ATOM	775	C	SER	A	194	-16.043	-5.958	1.566	47.00	0.00	C
ATOM	776	O	SER	A	194	-17.175	-6.436	1.485	47.00	0.00	O
ATOM	777	N	VAL	A	195	-15.793	-4.780	2.126	41.00	0.00	N
ATOM	778	CA	VAL	A	195	-16.861	-3.944	2.664	41.00	0.00	C
ATOM	779	C	VAL	A	195	-17.840	-3.534	1.572	41.00	0.00	C
ATOM	780	O	VAL	A	195	-19.045	-3.440	1.809	41.00	0.00	O
ATOM	781	N	ILE	A	196	-17.317	-3.288	0.376	42.60	0.00	N
ATOM	782	CA	ILE	A	196	-18.149	-2.922	-0.764	42.60	0.00	C
ATOM	783	C	ILE	A	196	-18.400	-4.119	-1.671	42.60	0.00	C
ATOM	784	O	ILE	A	196	-19.522	-4.343	-2.123	42.60	0.00	O
ATOM	785	N	CYS	A	197	-17.347	-4.886	-1.934	52.08	0.00	N
ATOM	786	CA	CYS	A	197	-17.459	-6.082	-2.761	52.08	0.00	C
ATOM	787	C	CYS	A	197	-16.514	-7.175	-2.279	52.08	0.00	C
ATOM	788	O	CYS	A	197	-15.293	-7.036	-2.365	52.08	0.00	O
ATOM	789	N	PRO	A	198	-17.085	-8.262	-1.772	46.10	0.00	N
ATOM	790	CA	PRO	A	198	-16.295	-9.396	-1.307	46.10	0.00	C
ATOM	791	C	PRO	A	198	-15.353	-9.896	-2.395	46.10	0.00	C
ATOM	792	O	PRO	A	198	-15.726	-9.968	-3.566	46.10	0.00	O
ATOM	793	N	ALA	A	199	-14.132	-10.239	-2.001	29.17	0.00	N
ATOM	794	CA	ALA	A	199	-13.109	-10.655	-2.953	29.17	0.00	C
ATOM	795	C	ALA	A	199	-13.340	-12.087	-3.420	29.17	0.00	C
ATOM	796	O	ALA	A	199	-14.042	-12.857	-2.766	29.17	0.00	O
ATOM	797	N	LYS	A	200	-12.745	-12.437	-4.556	51.72	0.00	N
ATOM	798	CA	LYS	A	200	-12.793	-13.805	-5.056	51.72	0.00	C
ATOM	799	C	LYS	A	200	-12.228	-14.785	-4.035	51.72	0.00	C
ATOM	800	O	LYS	A	200	-12.727	-15.902	-3.891	51.72	0.00	O

```
ATOM    801  N   LEU A 201     -11.187 -14.360  -3.329 46.35  0.00           N
ATOM    802  CA  LEU A 201     -10.613 -15.161  -2.255 46.35  0.00           C
ATOM    803  C   LEU A 201     -11.557 -15.242  -1.063 46.35  0.00           C
ATOM    804  O   LEU A 201     -11.734 -16.307  -0.470 46.35  0.00           O
ATOM    805  N   THR A 202     -12.162 -14.111  -0.715 44.58  0.00           N
ATOM    806  CA  THR A 202     -13.093 -14.053   0.406 44.58  0.00           C
ATOM    807  C   THR A 202     -14.063 -15.227   0.377 44.58  0.00           C
ATOM    808  O   THR A 202     -13.717 -16.302   0.783 44.58  0.00           O
TER     809      THR A 202
ENDMDL
END
REMARK   1 ***********************************************************************
REMARK   1 Start File DEC4_vs_NAT_dSi_colored.pdb
REMARK   1 The occupancy record was modified to represent dSi := Si(DEC)-Si(NAT).
REMARK   1 Occ=0.00 means dSi=-1.484741 kB; Occ=99.99 means dSi=1.546813 kB.
MODEL        0
ATOM      1  N   MET A   1      22.940  -3.371 -15.900 48.49  0.00           N
ATOM      2  CA  MET A   1      21.679  -3.206 -16.614 48.49  0.00           C
ATOM      3  C   MET A   1      20.708  -2.338 -15.824 48.49  0.00           C
ATOM      4  O   MET A   1      19.875  -1.639 -16.400 48.49  0.00           O
ATOM      5  N   ILE A   2      20.820  -2.387 -14.502 39.73  0.00           N
ATOM      6  CA  ILE A   2      19.952  -1.606 -13.629 39.73  0.00           C
ATOM      7  C   ILE A   2      20.651  -0.344 -13.141 39.73  0.00           C
ATOM      8  O   ILE A   2      21.639  -0.413 -12.409 39.73  0.00           O
ATOM      9  N   THR A   3      20.132   0.810 -13.548 54.25  0.00           N
ATOM     10  CA  THR A   3      20.740   2.088 -13.201 54.25  0.00           C
ATOM     11  C   THR A   3      20.262   2.574 -11.840 54.25  0.00           C
ATOM     12  O   THR A   3      19.353   1.993 -11.247 54.25  0.00           O
ATOM     13  N   GLU A   4      20.879   3.643 -11.348 44.58  0.00           N
ATOM     14  CA  GLU A   4      20.456   4.265 -10.099 44.58  0.00           C
ATOM     15  C   GLU A   4      18.987   4.663 -10.151 44.58  0.00           C
ATOM     16  O   GLU A   4      18.257   4.506  -9.172 44.58  0.00           O
ATOM     17  N   PHE A   5      18.558   5.178 -11.298 52.03  0.00           N
ATOM     18  CA  PHE A   5      17.154   5.505 -11.514 52.03  0.00           C
ATOM     19  C   PHE A   5      16.266   4.286 -11.301 52.03  0.00           C
ATOM     20  O   PHE A   5      15.275   4.348 -10.573 52.03  0.00           O
ATOM     21  N   LEU A   6      16.626   3.178 -11.941 45.33  0.00           N
ATOM     22  CA  LEU A   6      15.823   1.962 -11.878 45.33  0.00           C
ATOM     23  C   LEU A   6      15.843   1.361 -10.478 45.33  0.00           C
ATOM     24  O   LEU A   6      14.870   0.746 -10.043 45.33  0.00           O
ATOM     25  N   LEU A   7      16.957   1.543  -9.777 34.02  0.00           N
ATOM     26  CA  LEU A   7      17.069   1.107  -8.391 34.02  0.00           C
ATOM     27  C   LEU A   7      16.083   1.849  -7.497 34.02  0.00           C
ATOM     28  O   LEU A   7      15.365   1.237  -6.708 34.02  0.00           O
ATOM     29  N   LYS A   8      16.055   3.171  -7.627 38.58  0.00           N
ATOM     30  CA  LYS A   8      15.133   3.996  -6.857 38.58  0.00           C
ATOM     31  C   LYS A   8      13.688   3.729  -7.256 38.58  0.00           C
ATOM     32  O   LYS A   8      12.786   3.760  -6.419 38.58  0.00           O
ATOM     33  N   LYS A   9      13.473   3.466  -8.541 37.09  0.00           N
ATOM     34  CA  LYS A   9      12.150   3.114  -9.041 37.09  0.00           C
ATOM     35  C   LYS A   9      11.619   1.860  -8.359 37.09  0.00           C
ATOM     36  O   LYS A   9      10.482   1.831  -7.887 37.09  0.00           O
ATOM     37  N   LYS A  10      12.450   0.824  -8.308 45.03  0.00           N
ATOM     38  CA  LYS A  10      12.044  -0.456  -7.739 45.03  0.00           C
ATOM     39  C   LYS A  10      11.944  -0.377  -6.221 45.03  0.00           C
ATOM     40  O   LYS A  10      11.134  -1.071  -5.605 45.03  0.00           O
ATOM     41  N   LEU A  11      12.774   0.471  -5.622 27.27  0.00           N
ATOM     42  CA  LEU A  11      12.707   0.722  -4.187 27.27  0.00           C
ATOM     43  C   LEU A  11      11.367   1.331  -3.796 27.27  0.00           C
ATOM     44  O   LEU A  11      10.769   0.946  -2.790 27.27  0.00           O
ATOM     45  N   GLU A  12      10.899   2.282  -4.597 25.13  0.00           N
ATOM     46  CA  GLU A  12       9.606   2.915  -4.361 25.13  0.00           C
ATOM     47  C   GLU A  12       8.463   1.935  -4.590 25.13  0.00           C
ATOM     48  O   GLU A  12       7.483   1.925  -3.846 25.13  0.00           O
ATOM     49  N   GLU A  13       8.594   1.111  -5.625 28.47  0.00           N
ATOM     50  CA  GLU A  13       7.569   0.129  -5.958 28.47  0.00           C
ATOM     51  C   GLU A  13       7.478  -0.954  -4.890 28.47  0.00           C
ATOM     52  O   GLU A  13       6.392  -1.445  -4.582 28.47  0.00           O
ATOM     53  N   HIS A  14       8.624  -1.323  -4.330 20.56  0.00           N
ATOM     54  CA  HIS A  14       8.679  -2.368  -3.314 20.56  0.00           C
ATOM     55  C   HIS A  14       8.471  -1.793  -1.919 20.56  0.00           C
ATOM     56  O   HIS A  14       8.124  -2.515  -0.984 20.56  0.00           O
ATOM     57  N   LEU A  15       8.684  -0.489  -1.785  7.47  0.00           N
ATOM     58  CA  LEU A  15       8.359   0.218  -0.552  7.47  0.00           C
ATOM     59  C   LEU A  15       6.881   0.080  -0.211  7.47  0.00           C
ATOM     60  O   LEU A  15       6.514  -0.084   0.952  7.47  0.00           O
ATOM     61  N   SER A  16       6.034   0.147  -1.234 18.29  0.00           N
ATOM     62  CA  SER A  16       4.600  -0.041  -1.055 18.29  0.00           C
ATOM     63  C   SER A  16       4.278  -1.475  -0.654 18.29  0.00           C
ATOM     64  O   SER A  16       3.175  -1.769  -0.195 18.29  0.00           O
ATOM     65  N   HIS A  17       5.249  -2.366  -0.832 18.83  0.00           N
ATOM     66  CA  HIS A  17       5.069  -3.772  -0.492 18.83  0.00           C
ATOM     67  C   HIS A  17       5.478  -4.047   0.950 18.83  0.00           C
ATOM     68  O   HIS A  17       5.283  -5.148   1.461 18.83  0.00           O
ATOM     69  N   VAL A  18       6.045  -3.036   1.601 21.61  0.00           N
ATOM     70  CA  VAL A  18       6.578  -3.198   2.949 21.61  0.00           C
ATOM     71  C   VAL A  18       5.774  -2.388   3.959 21.61  0.00           C
ATOM     72  O   VAL A  18       5.456  -1.223   3.723 21.61  0.00           O
ATOM     73  N   LYS A  19       5.450  -3.012   5.085 41.33  0.00           N
ATOM     74  CA  LYS A  19       4.620  -2.378   6.102 41.33  0.00           C
ATOM     75  C   LYS A  19       5.293  -1.132   6.663 41.33  0.00           C
ATOM     76  O   LYS A  19       4.645  -0.108   6.880 41.33  0.00           O
```

```
ATOM     77  N   GLU A  20       6.599  -1.224   6.895 49.34  0.00           N
ATOM     78  CA  GLU A  20       7.361  -0.106   7.438 49.34  0.00           C
ATOM     79  C   GLU A  20       7.122   1.166   6.635 49.34  0.00           C
ATOM     80  O   GLU A  20       7.280   1.180   5.415 49.34  0.00           O
ATOM     81  N   GLU A  21       6.739   2.233   7.327 48.16  0.00           N
ATOM     82  CA  GLU A  21       6.395   3.489   6.672 48.16  0.00           C
ATOM     83  C   GLU A  21       7.636   4.178   6.116 48.16  0.00           C
ATOM     84  O   GLU A  21       7.574   4.855   5.090 48.16  0.00           O
ATOM     85  N   ASN A  22       8.760   4.002   6.801 60.36  0.00           N
ATOM     86  CA  ASN A  22      10.012   4.627   6.392 60.36  0.00           C
ATOM     87  C   ASN A  22      10.488   4.083   5.051 60.36  0.00           C
ATOM     88  O   ASN A  22      10.222   2.931   4.708 60.36  0.00           O
ATOM     89  N   THR A  23      11.192   4.919   4.295 74.63  0.00           N
ATOM     90  CA  THR A  23      11.654   4.544   2.964 74.63  0.00           C
ATOM     91  C   THR A  23      12.794   3.537   3.040 74.63  0.00           C
ATOM     92  O   THR A  23      13.450   3.404   4.074 74.63  0.00           O
ATOM     93  N   ILE A  24      13.027   2.829   1.940 61.70  0.00           N
ATOM     94  CA  ILE A  24      14.033   1.774   1.905 61.70  0.00           C
ATOM     95  C   ILE A  24      15.075   2.043   0.827 61.70  0.00           C
ATOM     96  O   ILE A  24      14.772   2.632  -0.210 61.70  0.00           O
ATOM     97  N   TYR A  25      16.305   1.607   1.079 57.19  0.00           N
ATOM     98  CA  TYR A  25      17.397   1.805   0.133 57.19  0.00           C
ATOM     99  C   TYR A  25      18.289   0.573   0.055 57.19  0.00           C
ATOM    100  O   TYR A  25      18.571  -0.065   1.068 57.19  0.00           O
ATOM    101  N   VAL A  26      18.729   0.243  -1.154 64.40  0.00           N
ATOM    102  CA  VAL A  26      19.552  -0.940  -1.374 64.40  0.00           C
ATOM    103  C   VAL A  26      20.871  -0.842  -0.620 64.40  0.00           C
ATOM    104  O   VAL A  26      21.181  -1.687   0.221 64.40  0.00           O
ATOM    105  N   THR A  27      21.645   0.194  -0.922 61.12  0.00           N
ATOM    106  CA  THR A  27      22.972   0.355  -0.342 61.12  0.00           C
ATOM    107  C   THR A  27      22.906   0.402   1.179 61.12  0.00           C
ATOM    108  O   THR A  27      23.690  -0.256   1.864 61.12  0.00           O
ATOM    109  N   ASP A  28      21.966   1.181   1.703 60.47  0.00           N
ATOM    110  CA  ASP A  28      21.878   1.417   3.139 60.47  0.00           C
ATOM    111  C   ASP A  28      21.527   0.137   3.887 60.47  0.00           C
ATOM    112  O   ASP A  28      22.187  -0.224   4.861 60.47  0.00           O
ATOM    113  N   LEU A  29      20.486  -0.545   3.425 76.20  0.00           N
ATOM    114  CA  LEU A  29      19.945  -1.696   4.138 76.20  0.00           C
ATOM    115  C   LEU A  29      20.842  -2.917   3.973 76.20  0.00           C
ATOM    116  O   LEU A  29      20.889  -3.786   4.843 76.20  0.00           O
ATOM    117  N   VAL A  30      21.552  -2.976   2.852 58.21  0.00           N
ATOM    118  CA  VAL A  30      22.523  -4.037   2.615 58.21  0.00           C
ATOM    119  C   VAL A  30      23.752  -3.868   3.498 58.21  0.00           C
ATOM    120  O   VAL A  30      24.287  -4.842   4.028 58.21  0.00           O
ATOM    121  N   ARG A  31      24.195  -2.625   3.655 99.99  0.00           N
ATOM    122  CA  ARG A  31      25.314  -2.316   4.538 99.99  0.00           C
ATOM    123  C   ARG A  31      24.928  -2.495   6.000 99.99  0.00           C
ATOM    124  O   ARG A  31      25.746  -2.912   6.821 99.99  0.00           O
ATOM    125  N   CYS A  32      23.679  -2.176   6.321 78.31  0.00           N
ATOM    126  CA  CYS A  32      23.184  -2.297   7.686 78.31  0.00           C
ATOM    127  C   CYS A  32      23.117  -3.755   8.122 78.31  0.00           C
ATOM    128  O   CYS A  32      22.743  -4.629   7.341 78.31  0.00           O
ATOM    129  N   PRO A  33      23.482  -4.010   9.374 51.38  0.00           N
ATOM    130  CA  PRO A  33      23.412  -5.355   9.933 51.38  0.00           C
ATOM    131  C   PRO A  33      22.013  -5.939   9.792 51.38  0.00           C
ATOM    132  O   PRO A  33      21.835  -7.158   9.799 51.38  0.00           O
ATOM    133  N   ARG A  34      21.022  -5.064   9.662 62.75  0.00           N
ATOM    134  CA  ARG A  34      19.647  -5.491   9.435 62.75  0.00           C
ATOM    135  C   ARG A  34      19.584  -6.641   8.439 62.75  0.00           C
ATOM    136  O   ARG A  34      18.842  -7.604   8.632 62.75  0.00           O
ATOM    137  N   ARG A  35      20.370  -6.536   7.372 35.09  0.00           N
ATOM    138  CA  ARG A  35      20.418  -7.576   6.350 35.09  0.00           C
ATOM    139  C   ARG A  35      20.877  -8.904   6.937 35.09  0.00           C
ATOM    140  O   ARG A  35      20.329  -9.958   6.617 35.09  0.00           O
ATOM    141  N   VAL A  36      21.888  -8.846   7.798 55.22  0.00           N
ATOM    142  CA  VAL A  36      22.444 -10.048   8.408 55.22  0.00           C
ATOM    143  C   VAL A  36      21.400 -10.772   9.248 55.22  0.00           C
ATOM    144  O   VAL A  36      21.215 -11.982   9.119 55.22  0.00           O
ATOM    145  N   ARG A  37      20.721 -10.024  10.112 66.63  0.00           N
ATOM    146  CA  ARG A  37      19.624 -10.571  10.901 66.63  0.00           C
ATOM    147  C   ARG A  37      18.525 -11.128  10.006 66.63  0.00           C
ATOM    148  O   ARG A  37      18.002 -12.215  10.252 66.63  0.00           O
ATOM    149  N   TYR A  38      18.179 -10.376   8.966 51.44  0.00           N
ATOM    150  CA  TYR A  38      17.141 -10.795   8.031 51.44  0.00           C
ATOM    151  C   TYR A  38      17.469 -12.147   7.412 51.44  0.00           C
ATOM    152  O   TYR A  38      16.620 -13.037   7.356 51.44  0.00           O
ATOM    153  N   GLU A  39      18.705 -12.296   6.949 76.94  0.00           N
ATOM    154  CA  GLU A  39      19.132 -13.520   6.282 76.94  0.00           C
ATOM    155  C   GLU A  39      19.150 -14.697   7.250 76.94  0.00           C
ATOM    156  O   GLU A  39      18.839 -15.827   6.873 76.94  0.00           O
ATOM    157  N   SER A  40      19.515 -14.425   8.498 51.76  0.00           N
ATOM    158  CA  SER A  40      19.515 -15.447   9.536 51.76  0.00           C
ATOM    159  C   SER A  40      18.100 -15.923   9.840 51.76  0.00           C
ATOM    160  O   SER A  40      17.865 -17.114  10.045 51.76  0.00           O
ATOM    161  N   GLU A  41      17.158 -14.985   9.866 49.17  0.00           N
ATOM    162  CA  GLU A  41      15.761 -15.310  10.123 49.17  0.00           C
ATOM    163  C   GLU A  41      15.095 -15.897   8.885 49.17  0.00           C
ATOM    164  O   GLU A  41      14.186 -16.720   8.989 49.17  0.00           O
ATOM    165  N   TYR A  42      15.553 -15.469   7.714 54.89  0.00           N
ATOM    166  CA  TYR A  42      14.967 -15.910   6.454 54.89  0.00           C
ATOM    167  C   TYR A  42      16.023 -16.508   5.534 54.89  0.00           C
ATOM    168  O   TYR A  42      16.308 -15.967   4.465 54.89  0.00           O
```

ATOM	169	N	LYS	A	43	16.600	-17.628	5.955	62.62	0.00	N
ATOM	170	CA	LYS	A	43	17.667	-18.271	5.197	62.62	0.00	C
ATOM	171	C	LYS	A	43	17.150	-18.819	3.873	62.62	0.00	C
ATOM	172	O	LYS	A	43	17.924	-19.064	2.948	62.62	0.00	O
ATOM	173	N	GLU	A	44	15.837	-19.008	3.789	58.52	0.00	N
ATOM	174	CA	GLU	A	44	15.217	-19.551	2.587	58.52	0.00	C
ATOM	175	C	GLU	A	44	15.049	-18.475	1.521	58.52	0.00	C
ATOM	176	O	GLU	A	44	14.828	-18.778	0.348	58.52	0.00	O
ATOM	177	N	LEU	A	45	15.155	-17.217	1.936	58.40	0.00	N
ATOM	178	CA	LEU	A	45	14.954	-16.092	1.028	58.40	0.00	C
ATOM	179	C	LEU	A	45	16.286	-15.524	0.556	58.40	0.00	C
ATOM	180	O	LEU	A	45	17.221	-15.372	1.341	58.40	0.00	O
ATOM	181	N	ALA	A	46	16.365	-15.209	-0.733	72.70	0.00	N
ATOM	182	CA	ALA	A	46	17.584	-14.658	-1.314	72.70	0.00	C
ATOM	183	C	ALA	A	46	17.434	-13.169	-1.596	72.70	0.00	C
ATOM	184	O	ALA	A	46	16.357	-12.701	-1.963	72.70	0.00	O
ATOM	185	N	ILE	A	47	18.524	-12.427	-1.423	57.95	0.00	N
ATOM	186	CA	ILE	A	47	18.524	-10.994	-1.688	57.95	0.00	C
ATOM	187	C	ILE	A	47	19.415	-10.654	-2.877	57.95	0.00	C
ATOM	188	O	ILE	A	47	20.595	-11.004	-2.900	57.95	0.00	O
ATOM	189	N	SER	A	48	18.842	-9.972	-3.862	66.06	0.00	N
ATOM	190	CA	SER	A	48	19.577	-9.602	-5.067	66.06	0.00	C
ATOM	191	C	SER	A	48	20.474	-8.397	-4.817	66.06	0.00	C
ATOM	192	O	SER	A	48	20.471	-7.824	-3.727	66.06	0.00	O
ATOM	193	N	GLN	A	49	21.239	-8.016	-5.833	66.28	0.00	N
ATOM	194	CA	GLN	A	49	22.124	-6.860	-5.735	66.28	0.00	C
ATOM	195	C	GLN	A	49	21.419	-5.587	-6.184	66.28	0.00	C
ATOM	196	O	GLN	A	49	22.009	-4.507	-6.187	66.28	0.00	O
ATOM	197	N	VAL	A	50	20.151	-5.720	-6.562	60.14	0.00	N
ATOM	198	CA	VAL	A	50	19.370	-4.584	-7.034	60.14	0.00	C
ATOM	199	C	VAL	A	50	18.157	-4.342	-6.145	60.14	0.00	C
ATOM	200	O	VAL	A	50	17.531	-3.284	-6.206	60.14	0.00	O
ATOM	201	N	TYR	A	51	17.829	-5.329	-5.318	32.22	0.00	N
ATOM	202	CA	TYR	A	51	16.739	-5.196	-4.359	32.22	0.00	C
ATOM	203	C	TYR	A	51	17.267	-5.124	-2.932	32.22	0.00	C
ATOM	204	O	TYR	A	51	18.234	-5.800	-2.582	32.22	0.00	O
ATOM	205	N	ALA	A	52	16.627	-4.298	-2.111	41.28	0.00	N
ATOM	206	CA	ALA	A	52	16.946	-4.232	-0.689	41.28	0.00	C
ATOM	207	C	ALA	A	52	16.343	-5.410	0.065	41.28	0.00	C
ATOM	208	O	ALA	A	52	15.367	-6.012	-0.382	41.28	0.00	O
ATOM	209	N	PRO	A	53	16.932	-5.736	1.211	48.93	0.00	N
ATOM	210	CA	PRO	A	53	16.404	-6.788	2.071	48.93	0.00	C
ATOM	211	C	PRO	A	53	14.920	-6.582	2.346	48.93	0.00	C
ATOM	212	O	PRO	A	53	14.144	-7.538	2.367	48.93	0.00	O
ATOM	213	N	SER	A	54	14.530	-5.329	2.558	36.30	0.00	N
ATOM	214	CA	SER	A	54	13.134	-4.993	2.804	36.30	0.00	C
ATOM	215	C	SER	A	54	12.296	-5.162	1.543	36.30	0.00	C
ATOM	216	O	SER	A	54	11.159	-5.632	1.599	36.30	0.00	O
ATOM	217	N	ALA	A	55	12.864	-4.774	0.406	32.49	0.00	N
ATOM	218	CA	ALA	A	55	12.202	-4.953	-0.881	32.49	0.00	C
ATOM	219	C	ALA	A	55	11.919	-6.425	-1.155	32.49	0.00	C
ATOM	220	O	ALA	A	55	10.871	-6.775	-1.698	32.49	0.00	O
ATOM	221	N	ILE	A	56	12.860	-7.283	-0.777	36.07	0.00	N
ATOM	222	CA	ILE	A	56	12.678	-8.725	-0.902	36.07	0.00	C
ATOM	223	C	ILE	A	56	11.552	-9.217	-0.002	36.07	0.00	C
ATOM	224	O	ILE	A	56	10.703	-10.002	-0.427	36.07	0.00	O
ATOM	225	N	LEU	A	57	11.549	-8.752	1.242	30.30	0.00	N
ATOM	226	CA	LEU	A	57	10.497	-9.104	2.189	30.30	0.00	C
ATOM	227	C	LEU	A	57	9.118	-8.790	1.623	30.30	0.00	C
ATOM	228	O	LEU	A	57	8.232	-9.643	1.611	30.30	0.00	O
ATOM	229	N	GLY	A	58	8.943	-7.559	1.154	40.09	0.00	N
ATOM	230	CA	GLY	A	58	7.654	-7.110	0.641	40.09	0.00	C
ATOM	231	C	GLY	A	58	7.201	-7.966	-0.535	40.09	0.00	C
ATOM	232	O	GLY	A	58	6.036	-8.356	-0.619	40.09	0.00	O
ATOM	233	N	ASP	A	59	8.128	-8.256	-1.442	43.66	0.00	N
ATOM	234	CA	ASP	A	59	7.826	-9.068	-2.613	43.66	0.00	C
ATOM	235	C	ASP	A	59	7.396	-10.474	-2.212	43.66	0.00	C
ATOM	236	O	ASP	A	59	6.446	-11.026	-2.770	43.66	0.00	O
ATOM	237	N	ILE	A	60	8.100	-11.049	-1.245	47.37	0.00	N
ATOM	238	CA	ILE	A	60	7.814	-12.405	-0.789	47.37	0.00	C
ATOM	239	C	ILE	A	60	6.494	-12.464	-0.031	47.37	0.00	C
ATOM	240	O	ILE	A	60	5.779	-13.464	-0.086	47.37	0.00	O
ATOM	241	N	LEU	A	61	6.177	-11.386	0.679	37.07	0.00	N
ATOM	242	CA	LEU	A	61	4.911	-11.286	1.395	37.07	0.00	C
ATOM	243	C	LEU	A	61	3.731	-11.293	0.431	37.07	0.00	C
ATOM	244	O	LEU	A	61	2.736	-11.982	0.660	37.07	0.00	O
ATOM	245	N	HIS	A	62	3.848	-10.523	-0.645	40.48	0.00	N
ATOM	246	CA	HIS	A	62	2.818	-10.489	-1.677	40.48	0.00	C
ATOM	247	C	HIS	A	62	2.781	-11.794	-2.462	40.48	0.00	C
ATOM	248	O	HIS	A	62	1.729	-12.206	-2.950	40.48	0.00	O
ATOM	249	N	LEU	A	63	3.935	-12.441	-2.580	37.63	0.00	N
ATOM	250	CA	LEU	A	63	4.016	-13.758	-3.201	37.63	0.00	C
ATOM	251	C	LEU	A	63	3.261	-14.799	-2.385	37.63	0.00	C
ATOM	252	O	LEU	A	63	2.559	-15.646	-2.938	37.63	0.00	O
ATOM	253	N	GLY	A	64	3.409	-14.732	-1.066	32.57	0.00	N
ATOM	254	CA	GLY	A	64	2.692	-15.629	-0.168	32.57	0.00	C
ATOM	255	C	GLY	A	64	1.185	-15.430	-0.277	32.57	0.00	C
ATOM	256	O	GLY	A	64	0.430	-16.395	-0.401	32.57	0.00	O
ATOM	257	N	LEU	A	65	0.754	-14.175	-0.229	17.30	0.00	N
ATOM	258	CA	LEU	A	65	-0.661	-13.846	-0.346	17.30	0.00	C
ATOM	259	C	LEU	A	65	-1.201	-14.214	-1.722	17.30	0.00	C
ATOM	260	O	LEU	A	65	-2.347	-14.640	-1.857	17.30	0.00	O

ATOM	261	N	GLU	A	66	-0.367	-14.044	-2.743	52.15	0.00	N
ATOM	262	CA	GLU	A	66	-0.709	-14.480	-4.092	52.15	0.00	C
ATOM	263	C	GLU	A	66	-1.008	-15.973	-4.130	52.15	0.00	C
ATOM	264	O	GLU	A	66	-2.009	-16.402	-4.703	52.15	0.00	O
ATOM	265	N	SER	A	67	-0.132	-16.763	-3.516	48.13	0.00	N
ATOM	266	CA	SER	A	67	-0.279	-18.213	-3.513	48.13	0.00	C
ATOM	267	C	SER	A	67	-1.530	-18.638	-2.755	48.13	0.00	C
ATOM	268	O	SER	A	67	-2.250	-19.541	-3.182	48.13	0.00	O
ATOM	269	N	VAL	A	68	-1.784	-17.983	-1.627	43.23	0.00	N
ATOM	270	CA	VAL	A	68	-2.938	-18.305	-0.797	43.23	0.00	C
ATOM	271	C	VAL	A	68	-4.242	-18.010	-1.527	43.23	0.00	C
ATOM	272	O	VAL	A	68	-5.166	-18.823	-1.522	43.23	0.00	O
ATOM	273	N	LEU	A	69	-4.311	-16.841	-2.155	36.64	0.00	N
ATOM	274	CA	LEU	A	69	-5.508	-16.428	-2.877	36.64	0.00	C
ATOM	275	C	LEU	A	69	-5.759	-17.320	-4.086	36.64	0.00	C
ATOM	276	O	LEU	A	69	-6.878	-17.781	-4.309	36.64	0.00	O
ATOM	277	N	LYS	A	70	-4.708	-17.560	-4.865	44.43	0.00	N
ATOM	278	CA	LYS	A	70	-4.815	-18.385	-6.062	44.43	0.00	C
ATOM	279	C	LYS	A	70	-5.133	-19.833	-5.707	44.43	0.00	C
ATOM	280	O	LYS	A	70	-5.802	-20.535	-6.464	44.43	0.00	O
ATOM	281	N	GLY	A	71	-4.650	-20.273	-4.550	47.19	0.00	N
ATOM	282	CA	GLY	A	71	-4.967	-21.602	-4.043	47.19	0.00	C
ATOM	283	C	GLY	A	71	-6.442	-21.716	-3.682	47.19	0.00	C
ATOM	284	O	GLY	A	71	-7.105	-22.689	-4.042	47.19	0.00	O
ATOM	285	N	ASN	A	72	-6.952	-20.718	-2.970	43.26	0.00	N
ATOM	286	CA	ASN	A	72	-8.352	-20.701	-2.566	43.26	0.00	C
ATOM	287	C	ASN	A	72	-8.819	-19.285	-2.255	43.26	0.00	C
ATOM	288	O	ASN	A	72	-8.639	-18.792	-1.141	43.26	0.00	O
ATOM	289	N	PHE	A	73	-9.419	-18.634	-3.246	39.90	0.00	N
ATOM	290	CA	PHE	A	73	-9.900	-17.267	-3.084	39.90	0.00	C
ATOM	291	C	PHE	A	73	-10.956	-17.180	-1.991	39.90	0.00	C
ATOM	292	O	PHE	A	73	-11.075	-16.162	-1.309	39.90	0.00	O
ATOM	293	N	ASN	A	74	-11.721	-18.254	-1.827	60.33	0.00	N
ATOM	294	CA	ASN	A	74	-12.811	-18.278	-0.860	60.33	0.00	C
ATOM	295	C	ASN	A	74	-12.282	-18.284	0.568	60.33	0.00	C
ATOM	296	O	ASN	A	74	-12.955	-17.829	1.492	60.33	0.00	O
ATOM	297	N	ALA	A	75	-11.070	-18.802	0.743	52.99	0.00	N
ATOM	298	CA	ALA	A	75	-10.442	-18.856	2.057	52.99	0.00	C
ATOM	299	C	ALA	A	75	-10.089	-17.460	2.555	52.99	0.00	C
ATOM	300	O	ALA	A	75	-9.472	-16.671	1.839	52.99	0.00	O
ATOM	301	N	GLU	A	76	-10.485	-17.160	3.788	61.81	0.00	N
ATOM	302	CA	GLU	A	76	-10.231	-15.851	4.377	61.81	0.00	C
ATOM	303	C	GLU	A	76	-8.749	-15.505	4.332	61.81	0.00	C
ATOM	304	O	GLU	A	76	-7.919	-16.212	4.904	61.81	0.00	O
ATOM	305	N	THR	A	77	-8.421	-14.412	3.651	66.18	0.00	N
ATOM	306	CA	THR	A	77	-7.031	-14.016	3.459	66.18	0.00	C
ATOM	307	C	THR	A	77	-6.825	-12.546	3.797	66.18	0.00	C
ATOM	308	O	THR	A	77	-7.405	-11.666	3.162	66.18	0.00	O
ATOM	309	N	GLU	A	78	-5.995	-12.286	4.802	71.90	0.00	N
ATOM	310	CA	GLU	A	78	-5.709	-10.920	5.226	71.90	0.00	C
ATOM	311	C	GLU	A	78	-4.668	-10.271	4.324	71.90	0.00	C
ATOM	312	O	GLU	A	78	-3.494	-10.641	4.346	71.90	0.00	O
ATOM	313	N	VAL	A	79	-5.105	-9.299	3.529	45.89	0.00	N
ATOM	314	CA	VAL	A	79	-4.213	-8.602	2.610	45.89	0.00	C
ATOM	315	C	VAL	A	79	-4.144	-7.114	2.929	45.89	0.00	C
ATOM	316	O	VAL	A	79	-5.166	-6.427	2.949	45.89	0.00	O
ATOM	317	N	GLU	A	80	-2.936	-6.623	3.180	26.43	0.00	N
ATOM	318	CA	GLU	A	80	-2.721	-5.200	3.412	26.43	0.00	C
ATOM	319	C	GLU	A	80	-1.443	-4.719	2.735	26.43	0.00	C
ATOM	320	O	GLU	A	80	-0.424	-5.410	2.747	26.43	0.00	O
ATOM	321	N	THR	A	81	-1.504	-3.531	2.144	34.54	0.00	N
ATOM	322	CA	THR	A	81	-0.337	-2.929	1.512	34.54	0.00	C
ATOM	323	C	THR	A	81	-0.143	-1.488	1.967	34.54	0.00	C
ATOM	324	O	THR	A	81	-1.014	-0.911	2.618	34.54	0.00	O
ATOM	325	N	LEU	A	82	1.003	-0.912	1.622	26.00	0.00	N
ATOM	326	CA	LEU	A	82	1.323	0.454	2.017	26.00	0.00	C
ATOM	327	C	LEU	A	82	1.090	1.428	0.869	26.00	0.00	C
ATOM	328	O	LEU	A	82	1.534	1.196	-0.255	26.00	0.00	O
ATOM	329	N	ARG	A	83	0.391	2.520	1.160	0.00	0.00	N
ATOM	330	CA	ARG	A	83	0.134	3.553	0.164	0.00	0.00	C
ATOM	331	C	ARG	A	83	0.347	4.945	0.745	0.00	0.00	C
ATOM	332	O	ARG	A	83	0.076	5.185	1.922	0.00	0.00	O
ATOM	333	N	GLU	A	84	0.835	5.860	-0.086	16.62	0.00	N
ATOM	334	CA	GLU	A	84	1.049	7.240	0.334	16.62	0.00	C
ATOM	335	C	GLU	A	84	-0.142	8.116	-0.029	16.62	0.00	C
ATOM	336	O	GLU	A	84	-0.407	8.364	-1.206	16.62	0.00	O
ATOM	337	N	ILE	A	85	-0.858	8.585	0.987	49.03	0.00	N
ATOM	338	CA	ILE	A	85	-2.069	9.370	0.776	49.03	0.00	C
ATOM	339	C	ILE	A	85	-2.007	10.693	1.527	49.03	0.00	C
ATOM	340	O	ILE	A	85	-1.666	10.731	2.709	49.03	0.00	O
ATOM	341	N	ASN	A	86	-2.337	11.778	0.833	23.99	0.00	N
ATOM	342	CA	ASN	A	86	-2.324	13.105	1.436	23.99	0.00	C
ATOM	343	C	ASN	A	86	-3.583	13.347	2.259	23.99	0.00	C
ATOM	344	O	ASN	A	86	-4.681	13.458	1.714	23.99	0.00	O
ATOM	345	N	VAL	A	87	-3.416	13.430	3.575	34.12	0.00	N
ATOM	346	CA	VAL	A	87	-4.531	13.709	4.472	34.12	0.00	C
ATOM	347	C	VAL	A	87	-4.343	15.041	5.188	34.12	0.00	C
ATOM	348	O	VAL	A	87	-3.511	15.160	6.088	34.12	0.00	O
ATOM	349	N	GLY	A	88	-5.119	16.040	4.782	45.20	0.00	N
ATOM	350	CA	GLY	A	88	-5.113	17.335	5.452	45.20	0.00	C
ATOM	351	C	GLY	A	88	-3.864	18.132	5.098	45.20	0.00	C
ATOM	352	O	GLY	A	88	-3.455	19.024	5.841	45.20	0.00	O

```
ATOM    353  N   GLY A  89      -3.263  17.805   3.959 44.67  0.00           N
ATOM    354  CA  GLY A  89      -2.093  18.530   3.475 44.67  0.00           C
ATOM    355  C   GLY A  89      -0.806  17.806   3.845 44.67  0.00           C
ATOM    356  O   GLY A  89       0.289  18.247   3.494 44.67  0.00           O
ATOM    357  N   LYS A  90      -0.941  16.691   4.555 48.32  0.00           N
ATOM    358  CA  LYS A  90       0.211  15.897   4.963 48.32  0.00           C
ATOM    359  C   LYS A  90       0.109  14.469   4.442 48.32  0.00           C
ATOM    360  O   LYS A  90      -0.873  13.773   4.698 48.32  0.00           O
ATOM    361  N   VAL A  91       1.130  14.039   3.708 38.80  0.00           N
ATOM    362  CA  VAL A  91       1.150  12.698   3.134 38.80  0.00           C
ATOM    363  C   VAL A  91       1.535  11.658   4.177 38.80  0.00           C
ATOM    364  O   VAL A  91       2.628  11.706   4.742 38.80  0.00           O
ATOM    365  N   TYR A  92       0.632  10.717   4.428 52.81  0.00           N
ATOM    366  CA  TYR A  92       0.885   9.648   5.388 52.81  0.00           C
ATOM    367  C   TYR A  92       1.035   8.304   4.687 52.81  0.00           C
ATOM    368  O   TYR A  92       0.397   8.049   3.666 52.81  0.00           O
ATOM    369  N   LYS A  93       1.885   7.445   5.241 52.93  0.00           N
ATOM    370  CA  LYS A  93       2.034   6.084   4.742 52.93  0.00           C
ATOM    371  C   LYS A  93       1.070   5.133   5.439 52.93  0.00           C
ATOM    372  O   LYS A  93       1.323   4.689   6.560 52.93  0.00           O
ATOM    373  N   ILE A  94      -0.035   4.824   4.771 51.17  0.00           N
ATOM    374  CA  ILE A  94      -1.116   4.063   5.387 51.17  0.00           C
ATOM    375  C   ILE A  94      -1.045   2.592   4.998 51.17  0.00           C
ATOM    376  O   ILE A  94      -1.013   2.254   3.814 51.17  0.00           O
ATOM    377  N   LYS A  95      -1.020   1.720   6.000 49.55  0.00           N
ATOM    378  CA  LYS A  95      -1.006   0.281   5.764 49.55  0.00           C
ATOM    379  C   LYS A  95      -2.388  -0.325   5.965 49.55  0.00           C
ATOM    380  O   LYS A  95      -2.947  -0.269   7.061 49.55  0.00           O
ATOM    381  N   GLY A  96      -2.935  -0.905   4.903 51.60  0.00           N
ATOM    382  CA  GLY A  96      -4.266  -1.499   4.954 51.60  0.00           C
ATOM    383  C   GLY A  96      -4.818  -1.737   3.554 51.60  0.00           C
ATOM    384  O   GLY A  96      -4.064  -1.980   2.612 51.60  0.00           O
ATOM    385  N   ARG A  97      -6.138  -1.665   3.425 35.35  0.00           N
ATOM    386  CA  ARG A  97      -6.790  -1.817   2.129 35.35  0.00           C
ATOM    387  C   ARG A  97      -7.449  -0.515   1.688 35.35  0.00           C
ATOM    388  O   ARG A  97      -7.552   0.434   2.466 35.35  0.00           O
ATOM    389  N   ALA A  98      -7.893  -0.477   0.437 48.81  0.00           N
ATOM    390  CA  ALA A  98      -8.488   0.728  -0.128 48.81  0.00           C
ATOM    391  C   ALA A  98      -9.726   1.149   0.651 48.81  0.00           C
ATOM    392  O   ALA A  98     -10.544   0.312   1.033 48.81  0.00           O
ATOM    393  N   ASP A  99      -9.859   2.450   0.883 46.60  0.00           N
ATOM    394  CA  ASP A  99     -10.974   2.980   1.659 46.60  0.00           C
ATOM    395  C   ASP A  99     -11.962   3.722   0.770 46.60  0.00           C
ATOM    396  O   ASP A  99     -13.164   3.740   1.038 46.60  0.00           O
ATOM    397  N   ALA A 100     -11.448   4.337  -0.292 51.50  0.00           N
ATOM    398  CA  ALA A 100     -12.283   5.090  -1.219 51.50  0.00           C
ATOM    399  C   ALA A 100     -11.772   4.963  -2.648 51.50  0.00           C
ATOM    400  O   ALA A 100     -10.564   4.892  -2.881 51.50  0.00           O
ATOM    401  N   ILE A 101     -12.696   4.937  -3.603 67.57  0.00           N
ATOM    402  CA  ILE A 101     -12.338   4.891  -5.016 67.57  0.00           C
ATOM    403  C   ILE A 101     -12.902   6.091  -5.766 67.57  0.00           C
ATOM    404  O   ILE A 101     -14.116   6.276  -5.835 67.57  0.00           O
ATOM    405  N   ILE A 102     -12.011   6.904  -6.325 54.39  0.00           N
ATOM    406  CA  ILE A 102     -12.416   8.116  -7.027 54.39  0.00           C
ATOM    407  C   ILE A 102     -11.694   8.248  -8.360 54.39  0.00           C
ATOM    408  O   ILE A 102     -10.702   7.563  -8.611 54.39  0.00           O
ATOM    409  N   ARG A 103     -12.198   9.131  -9.215 48.81  0.00           N
ATOM    410  CA  ARG A 103     -11.593   9.366 -10.522 48.81  0.00           C
ATOM    411  C   ARG A 103     -11.341  10.849 -10.754 48.81  0.00           C
ATOM    412  O   ARG A 103     -12.113  11.697 -10.307 48.81  0.00           O
ATOM    413  N   ASN A 104     -10.255  11.157 -11.456 49.62  0.00           N
ATOM    414  CA  ASN A 104      -9.877  12.541 -11.713 49.62  0.00           C
ATOM    415  C   ASN A 104     -10.598  13.091 -12.938 49.62  0.00           C
ATOM    416  O   ASN A 104     -11.453  12.423 -13.519 49.62  0.00           O
ATOM    417  N   ASP A 105     -10.249  14.313 -13.325 50.98  0.00           N
ATOM    418  CA  ASP A 105     -10.918  14.988 -14.431 50.98  0.00           C
ATOM    419  C   ASP A 105     -10.667  14.266 -15.748 50.98  0.00           C
ATOM    420  O   ASP A 105     -11.487  14.325 -16.664 50.98  0.00           O
ATOM    421  N   ASN A 106      -9.530  13.585 -15.838 41.30  0.00           N
ATOM    422  CA  ASN A 106      -9.168  12.854 -17.045 41.30  0.00           C
ATOM    423  C   ASN A 106      -9.917  11.531 -17.134 41.30  0.00           C
ATOM    424  O   ASN A 106      -9.914  10.874 -18.175 41.30  0.00           O
ATOM    425  N   GLY A 107     -10.558  11.146 -16.037 55.42  0.00           N
ATOM    426  CA  GLY A 107     -11.321   9.903 -15.991 55.42  0.00           C
ATOM    427  C   GLY A 107     -10.455   8.741 -15.523 55.42  0.00           C
ATOM    428  O   GLY A 107     -10.858   7.582 -15.603 55.42  0.00           O
ATOM    429  N   LYS A 108      -9.260   9.059 -15.036 47.85  0.00           N
ATOM    430  CA  LYS A 108      -8.331   8.042 -14.559 47.85  0.00           C
ATOM    431  C   LYS A 108      -8.643   7.642 -13.123 47.85  0.00           C
ATOM    432  O   LYS A 108      -9.087   8.465 -12.322 47.85  0.00           O
ATOM    433  N   SER A 109      -8.407   6.374 -12.801 50.40  0.00           N
ATOM    434  CA  SER A 109      -8.781   5.830 -11.502 50.40  0.00           C
ATOM    435  C   SER A 109      -7.831   6.306 -10.411 50.40  0.00           C
ATOM    436  O   SER A 109      -6.650   6.545 -10.664 50.40  0.00           O
ATOM    437  N   ILE A 110      -8.353   6.442  -9.196 39.96  0.00           N
ATOM    438  CA  ILE A 110      -7.536   6.817  -8.049 39.96  0.00           C
ATOM    439  C   ILE A 110      -7.901   5.996  -6.820 39.96  0.00           C
ATOM    440  O   ILE A 110      -9.064   5.950  -6.416 39.96  0.00           O
ATOM    441  N   VAL A 111      -6.903   5.348  -6.230 55.49  0.00           N
ATOM    442  CA  VAL A 111      -7.106   4.583  -5.005 55.49  0.00           C
ATOM    443  C   VAL A 111      -6.747   5.406  -3.775 55.49  0.00           C
ATOM    444  O   VAL A 111      -5.591   5.784  -3.584 55.49  0.00           O
```

```
ATOM    445  N   ILE A 112      -7.746   5.684  -2.943 50.38  0.00           N
ATOM    446  CA  ILE A 112      -7.534   6.451  -1.722 50.38  0.00           C
ATOM    447  C   ILE A 112      -7.616   5.559  -0.490 50.38  0.00           C
ATOM    448  O   ILE A 112      -8.530   4.745  -0.359 50.38  0.00           O
ATOM    449  N   GLU A 113      -6.654   5.717   0.414 55.15  0.00           N
ATOM    450  CA  GLU A 113      -6.598   4.905   1.623 55.15  0.00           C
ATOM    451  C   GLU A 113      -6.339   5.766   2.854 55.15  0.00           C
ATOM    452  O   GLU A 113      -5.226   6.247   3.063 55.15  0.00           O
ATOM    453  N   ILE A 114      -7.374   5.956   3.665 49.82  0.00           N
ATOM    454  CA  ILE A 114      -7.273   6.796   4.852 49.82  0.00           C
ATOM    455  C   ILE A 114      -7.759   6.058   6.092 49.82  0.00           C
ATOM    456  O   ILE A 114      -8.959   5.849   6.272 49.82  0.00           O
ATOM    457  N   LYS A 115      -6.820   5.664   6.946 55.94  0.00           N
ATOM    458  CA  LYS A 115      -7.152   4.951   8.175 55.94  0.00           C
ATOM    459  C   LYS A 115      -6.577   5.661   9.395 55.94  0.00           C
ATOM    460  O   LYS A 115      -5.588   6.385   9.294 55.94  0.00           O
ATOM    461  N   THR A 116      -7.205   5.447  10.546 47.50  0.00           N
ATOM    462  CA  THR A 116      -6.766   6.076  11.786 47.50  0.00           C
ATOM    463  C   THR A 116      -5.408   5.542  12.224 47.50  0.00           C
ATOM    464  O   THR A 116      -5.008   4.444  11.837 47.50  0.00           O
ATOM    465  N   SER A 117      -4.703   6.325  13.034 38.09  0.00           N
ATOM    466  CA  SER A 117      -3.351   5.976  13.451 38.09  0.00           C
ATOM    467  C   SER A 117      -3.336   4.667  14.231 38.09  0.00           C
ATOM    468  O   SER A 117      -3.975   4.548  15.277 38.09  0.00           O
ATOM    469  N   ARG A 118      -2.604   3.685  13.716 50.72  0.00           N
ATOM    470  CA  ARG A 118      -2.512   2.380  14.358 50.72  0.00           C
ATOM    471  C   ARG A 118      -1.557   2.416  15.545 50.72  0.00           C
ATOM    472  O   ARG A 118      -0.610   3.203  15.568 50.72  0.00           O
ATOM    473  N   SER A 119      -1.808   1.558  16.527 49.06  0.00           N
ATOM    474  CA  SER A 119      -1.040   1.564  17.765 49.06  0.00           C
ATOM    475  C   SER A 119       0.425   1.233  17.506 49.06  0.00           C
ATOM    476  O   SER A 119       0.739   0.318  16.746 49.06  0.00           O
ATOM    477  N   ASP A 120       1.317   1.983  18.143 54.49  0.00           N
ATOM    478  CA  ASP A 120       2.749   1.729  18.034 54.49  0.00           C
ATOM    479  C   ASP A 120       3.481   2.147  19.303 54.49  0.00           C
ATOM    480  O   ASP A 120       3.459   3.317  19.687 54.49  0.00           O
ATOM    481  N   LYS A 121       4.128   1.185  19.950 48.74  0.00           N
ATOM    482  CA  LYS A 121       4.779   1.428  21.233 48.74  0.00           C
ATOM    483  C   LYS A 121       5.914   2.434  21.092 48.74  0.00           C
ATOM    484  O   LYS A 121       6.314   3.074  22.065 48.74  0.00           O
ATOM    485  N   GLY A 122       6.431   2.570  19.876 52.58  0.00           N
ATOM    486  CA  GLY A 122       7.548   3.470  19.614 52.58  0.00           C
ATOM    487  C   GLY A 122       7.077   4.914  19.493 52.58  0.00           C
ATOM    488  O   GLY A 122       7.883   5.844  19.515 52.58  0.00           O
ATOM    489  N   LEU A 123       5.767   5.094  19.363 73.63  0.00           N
ATOM    490  CA  LEU A 123       5.190   6.422  19.193 73.63  0.00           C
ATOM    491  C   LEU A 123       4.311   6.796  20.381 73.63  0.00           C
ATOM    492  O   LEU A 123       3.643   5.942  20.963 73.63  0.00           O
ATOM    493  N   PRO A 124       4.317   8.077  20.734 60.33  0.00           N
ATOM    494  CA  PRO A 124       3.503   8.570  21.838 60.33  0.00           C
ATOM    495  C   PRO A 124       2.026   8.284  21.604 60.33  0.00           C
ATOM    496  O   PRO A 124       1.556   8.288  20.466 60.33  0.00           O
ATOM    497  N   LEU A 125       1.297   8.036  22.687 46.18  0.00           N
ATOM    498  CA  LEU A 125      -0.146   7.843  22.612 46.18  0.00           C
ATOM    499  C   LEU A 125      -0.844   9.099  22.107 46.18  0.00           C
ATOM    500  O   LEU A 125      -1.748   9.027  21.275 46.18  0.00           O
ATOM    501  N   ILE A 126      -0.420  10.252  22.615 54.49  0.00           N
ATOM    502  CA  ILE A 126      -0.942  11.532  22.154 54.49  0.00           C
ATOM    503  C   ILE A 126      -0.661  11.740  20.672 54.49  0.00           C
ATOM    504  O   ILE A 126      -1.506  12.249  19.935 54.49  0.00           O
ATOM    505  N   HIS A 127       0.532  11.345  20.241 57.34  0.00           N
ATOM    506  CA  HIS A 127       0.884  11.375  18.826 57.34  0.00           C
ATOM    507  C   HIS A 127      -0.125  10.598  17.990 57.34  0.00           C
ATOM    508  O   HIS A 127      -0.563  11.062  16.939 57.34  0.00           O
ATOM    509  N   HIS A 128      -0.490   9.411  18.465 49.74  0.00           N
ATOM    510  CA  HIS A 128      -1.498   8.596  17.799 49.74  0.00           C
ATOM    511  C   HIS A 128      -2.850   9.299  17.780 49.74  0.00           C
ATOM    512  O   HIS A 128      -3.566   9.261  16.779 49.74  0.00           O
ATOM    513  N   LYS A 129      -3.195   9.938  18.893 85.68  0.00           N
ATOM    514  CA  LYS A 129      -4.480  10.613  19.022 85.68  0.00           C
ATOM    515  C   LYS A 129      -4.596  11.770  18.039 85.68  0.00           C
ATOM    516  O   LYS A 129      -5.650  11.987  17.441 85.68  0.00           O
ATOM    517  N   MET A 130      -3.506  12.513  17.877 84.86  0.00           N
ATOM    518  CA  MET A 130      -3.494  13.673  16.994 84.86  0.00           C
ATOM    519  C   MET A 130      -3.703  13.262  15.542 84.86  0.00           C
ATOM    520  O   MET A 130      -4.511  13.857  14.828 84.86  0.00           O
ATOM    521  N   GLN A 131      -2.970  12.242  15.109 72.35  0.00           N
ATOM    522  CA  GLN A 131      -3.103  11.724  13.754 72.35  0.00           C
ATOM    523  C   GLN A 131      -4.484  11.120  13.528 72.35  0.00           C
ATOM    524  O   GLN A 131      -5.106  11.339  12.489 72.35  0.00           O
ATOM    525  N   LEU A 132      -4.957  10.358  14.508 55.73  0.00           N
ATOM    526  CA  LEU A 132      -6.292   9.773  14.446 55.73  0.00           C
ATOM    527  C   LEU A 132      -7.343  10.831  14.141 55.73  0.00           C
ATOM    528  O   LEU A 132      -8.191  10.644  13.268 55.73  0.00           O
ATOM    529  N   GLN A 133      -7.284  11.944  14.865 90.16  0.00           N
ATOM    530  CA  GLN A 133      -8.214  13.046  14.653 90.16  0.00           C
ATOM    531  C   GLN A 133      -8.078  13.619  13.248 90.16  0.00           C
ATOM    532  O   GLN A 133      -9.075  13.899  12.583 90.16  0.00           O
ATOM    533  N   ILE A 134      -6.839  13.792  12.803 63.70  0.00           N
ATOM    534  CA  ILE A 134      -6.570  14.325  11.472 63.70  0.00           C
ATOM    535  C   ILE A 134      -7.221  13.467  10.394 63.70  0.00           C
ATOM    536  O   ILE A 134      -7.805  13.988   9.443 63.70  0.00           O
```

```
ATOM    537  N   TYR A 135      -7.119  12.153  10.548 52.62  0.00           N
ATOM    538  CA  TYR A 135      -7.697  11.221   9.588 52.62  0.00           C
ATOM    539  C   TYR A 135      -9.216  11.329   9.561 52.62  0.00           C
ATOM    540  O   TYR A 135      -9.830  11.323   8.493 52.62  0.00           O
ATOM    541  N   LEU A 136      -9.818  11.429  10.741 79.77  0.00           N
ATOM    542  CA  LEU A 136     -11.271  11.487  10.858 79.77  0.00           C
ATOM    543  C   LEU A 136     -11.803  12.851  10.438 79.77  0.00           C
ATOM    544  O   LEU A 136     -12.953  12.976  10.017 79.77  0.00           O
ATOM    545  N   TRP A 137     -10.961  13.871  10.557 62.11  0.00           N
ATOM    546  CA  TRP A 137     -11.339  15.226  10.170 62.11  0.00           C
ATOM    547  C   TRP A 137     -11.617  15.314   8.676 62.11  0.00           C
ATOM    548  O   TRP A 137     -12.650  15.834   8.256 62.11  0.00           O
ATOM    549  N   LEU A 138     -10.687  14.804   7.875 52.60  0.00           N
ATOM    550  CA  LEU A 138     -10.844  14.799   6.424 52.60  0.00           C
ATOM    551  C   LEU A 138     -11.970  13.868   5.995 52.60  0.00           C
ATOM    552  O   LEU A 138     -12.740  14.187   5.088 52.60  0.00           O
ATOM    553  N   PHE A 139     -12.064  12.718   6.653 56.87  0.00           N
ATOM    554  CA  PHE A 139     -13.124  11.758   6.370 56.87  0.00           C
ATOM    555  C   PHE A 139     -14.500  12.384   6.566 56.87  0.00           C
ATOM    556  O   PHE A 139     -15.374  12.268   5.707 56.87  0.00           O
ATOM    557  N   SER A 140     -14.686  13.048   7.701 58.25  0.00           N
ATOM    558  CA  SER A 140     -15.982  13.617   8.054 58.25  0.00           C
ATOM    559  C   SER A 140     -16.238  14.914   7.298 58.25  0.00           C
ATOM    560  O   SER A 140     -17.385  15.320   7.112 58.25  0.00           O
ATOM    561  N   ALA A 141     -15.162  15.563   6.866 50.28  0.00           N
ATOM    562  CA  ALA A 141     -15.270  16.801   6.103 50.28  0.00           C
ATOM    563  C   ALA A 141     -16.076  16.594   4.827 50.28  0.00           C
ATOM    564  O   ALA A 141     -15.862  15.627   4.096 50.28  0.00           O
ATOM    565  N   GLU A 142     -17.004  17.507   4.565 51.95  0.00           N
ATOM    566  CA  GLU A 142     -17.817  17.450   3.356 51.95  0.00           C
ATOM    567  C   GLU A 142     -16.973  17.697   2.112 51.95  0.00           C
ATOM    568  O   GLU A 142     -17.030  16.933   1.149 51.95  0.00           O
ATOM    569  N   LYS A 143     -16.189  18.770   2.140 77.71  0.00           N
ATOM    570  CA  LYS A 143     -15.351  19.135   1.004 77.71  0.00           C
ATOM    571  C   LYS A 143     -13.873  18.961   1.332 77.71  0.00           C
ATOM    572  O   LYS A 143     -13.460  19.117   2.481 77.71  0.00           O
ATOM    573  N   GLY A 144     -13.081  18.636   0.316 59.28  0.00           N
ATOM    574  CA  GLY A 144     -11.638  18.507   0.481 59.28  0.00           C
ATOM    575  C   GLY A 144     -10.987  17.958  -0.783 59.28  0.00           C
ATOM    576  O   GLY A 144     -11.667  17.668  -1.768 59.28  0.00           O
ATOM    577  N   ILE A 145      -9.666  17.818  -0.748 67.60  0.00           N
ATOM    578  CA  ILE A 145      -8.921  17.294  -1.887 67.60  0.00           C
ATOM    579  C   ILE A 145      -8.173  16.020  -1.516 67.60  0.00           C
ATOM    580  O   ILE A 145      -7.423  15.991  -0.539 67.60  0.00           O
ATOM    581  N   LEU A 146      -8.381  14.968  -2.301 54.97  0.00           N
ATOM    582  CA  LEU A 146      -7.711  13.694  -2.067 54.97  0.00           C
ATOM    583  C   LEU A 146      -6.575  13.478  -3.058 54.97  0.00           C
ATOM    584  O   LEU A 146      -6.752  13.644  -4.265 54.97  0.00           O
ATOM    585  N   VAL A 147      -5.409  13.105  -2.543 46.30  0.00           N
ATOM    586  CA  VAL A 147      -4.211  12.987  -3.365 46.30  0.00           C
ATOM    587  C   VAL A 147      -3.556  11.623  -3.191 46.30  0.00           C
ATOM    588  O   VAL A 147      -3.260  11.204  -2.072 46.30  0.00           O
ATOM    589  N   TYR A 148      -3.333  10.933  -4.304 50.57  0.00           N
ATOM    590  CA  TYR A 148      -2.767   9.590  -4.273 50.57  0.00           C
ATOM    591  C   TYR A 148      -1.394   9.554  -4.930 50.57  0.00           C
ATOM    592  O   TYR A 148      -1.268   9.755  -6.138 50.57  0.00           O
ATOM    593  N   ILE A 149      -0.366   9.297  -4.129 43.47  0.00           N
ATOM    594  CA  ILE A 149       1.010   9.343  -4.609 43.47  0.00           C
ATOM    595  C   ILE A 149       1.490   7.962  -5.039 43.47  0.00           C
ATOM    596  O   ILE A 149       1.446   7.010  -4.261 43.47  0.00           O
ATOM    597  N   THR A 150       1.946   7.861  -6.282 49.22  0.00           N
ATOM    598  CA  THR A 150       2.562   6.635  -6.775 49.22  0.00           C
ATOM    599  C   THR A 150       4.019   6.863  -7.154 49.22  0.00           C
ATOM    600  O   THR A 150       4.459   8.002  -7.307 49.22  0.00           O
ATOM    601  N   PRO A 151       4.763   5.772  -7.306 41.72  0.00           N
ATOM    602  CA  PRO A 151       6.179   5.853  -7.645 41.72  0.00           C
ATOM    603  C   PRO A 151       6.395   6.667  -8.914 41.72  0.00           C
ATOM    604  O   PRO A 151       7.421   7.329  -9.071 41.72  0.00           O
ATOM    605  N   ASP A 152       5.423   6.616  -9.817 44.73  0.00           N
ATOM    606  CA  ASP A 152       5.532   7.300 -11.101 44.73  0.00           C
ATOM    607  C   ASP A 152       4.993   8.721 -11.015 44.73  0.00           C
ATOM    608  O   ASP A 152       5.706   9.685 -11.297 44.73  0.00           O
ATOM    609  N   ARG A 153       3.728   8.846 -10.626 52.13  0.00           N
ATOM    610  CA  ARG A 153       3.024  10.120 -10.706 52.13  0.00           C
ATOM    611  C   ARG A 153       2.146  10.343  -9.481 52.13  0.00           C
ATOM    612  O   ARG A 153       1.921   9.425  -8.692 52.13  0.00           O
ATOM    613  N   ILE A 154       1.652  11.566  -9.327 42.27  0.00           N
ATOM    614  CA  ILE A 154       0.750  11.897  -8.230 42.27  0.00           C
ATOM    615  C   ILE A 154      -0.618  12.317  -8.750 42.27  0.00           C
ATOM    616  O   ILE A 154      -0.743  13.313  -9.462 42.27  0.00           O
ATOM    617  N   ALA A 155      -1.643  11.554  -8.388 45.33  0.00           N
ATOM    618  CA  ALA A 155      -3.006  11.847  -8.816 45.33  0.00           C
ATOM    619  C   ALA A 155      -3.734  12.702  -7.788 45.33  0.00           C
ATOM    620  O   ALA A 155      -3.501  12.577  -6.585 45.33  0.00           O
ATOM    621  N   GLU A 156      -4.619  13.570  -8.266 43.25  0.00           N
ATOM    622  CA  GLU A 156      -5.316  14.511  -7.398 43.25  0.00           C
ATOM    623  C   GLU A 156      -6.741  14.752  -7.877 43.25  0.00           C
ATOM    624  O   GLU A 156      -6.981  14.948  -9.069 43.25  0.00           O
ATOM    625  N   TYR A 157      -7.685  14.734  -6.943 49.46  0.00           N
ATOM    626  CA  TYR A 157      -9.081  15.016  -7.259 49.46  0.00           C
ATOM    627  C   TYR A 157      -9.804  15.622  -6.064 49.46  0.00           C
ATOM    628  O   TYR A 157      -9.726  15.104  -4.949 49.46  0.00           O
```

ATOM	629	N	GLU	A	158	-10.510	16.723	-6.300	47.41	0.00	N
ATOM	630	CA	GLU	A	158	-11.288	17.375	-5.254	47.41	0.00	C
ATOM	631	C	GLU	A	158	-12.675	16.758	-5.134	47.41	0.00	C
ATOM	632	O	GLU	A	158	-13.337	16.493	-6.136	47.41	0.00	O
ATOM	633	N	ILE	A	159	-13.110	16.531	-3.899	51.83	0.00	N
ATOM	634	CA	ILE	A	159	-14.441	15.993	-3.642	51.83	0.00	C
ATOM	635	C	ILE	A	159	-15.297	16.987	-2.868	51.83	0.00	C
ATOM	636	O	ILE	A	159	-14.781	17.797	-2.097	51.83	0.00	O
ATOM	637	N	ASN	A	160	-16.607	16.922	-3.077	61.93	0.00	N
ATOM	638	CA	ASN	A	160	-17.536	17.825	-2.408	61.93	0.00	C
ATOM	639	C	ASN	A	160	-18.543	17.053	-1.563	61.93	0.00	C
ATOM	640	O	ASN	A	160	-19.567	17.597	-1.151	61.93	0.00	O
ATOM	641	N	GLU	A	161	-18.244	15.783	-1.310	47.82	0.00	N
ATOM	642	CA	GLU	A	161	-19.142	14.924	-0.548	47.82	0.00	C
ATOM	643	C	GLU	A	161	-18.371	13.825	0.173	47.82	0.00	C
ATOM	644	O	GLU	A	161	-17.489	13.191	-0.405	47.82	0.00	O
ATOM	645	N	PRO	A	162	-18.712	13.603	1.438	55.75	0.00	N
ATOM	646	CA	PRO	A	162	-18.103	12.533	2.219	55.75	0.00	C
ATOM	647	C	PRO	A	162	-18.227	11.192	1.507	55.75	0.00	C
ATOM	648	O	PRO	A	162	-19.284	10.858	0.972	55.75	0.00	O
ATOM	649	N	LEU	A	163	-17.140	10.427	1.504	62.98	0.00	N
ATOM	650	CA	LEU	A	163	-17.111	9.141	0.818	62.98	0.00	C
ATOM	651	C	LEU	A	163	-17.369	7.995	1.788	62.98	0.00	C
ATOM	652	O	LEU	A	163	-16.910	8.018	2.930	62.98	0.00	O
ATOM	653	N	ASP	A	164	-18.107	6.991	1.326	51.91	0.00	N
ATOM	654	CA	ASP	A	164	-18.512	5.880	2.180	51.91	0.00	C
ATOM	655	C	ASP	A	164	-17.432	4.809	2.240	51.91	0.00	C
ATOM	656	O	ASP	A	164	-17.272	4.022	1.306	51.91	0.00	O
ATOM	657	N	GLU	A	165	-16.692	4.782	3.343	46.92	0.00	N
ATOM	658	CA	GLU	A	165	-15.608	3.822	3.516	46.92	0.00	C
ATOM	659	C	GLU	A	165	-16.141	2.394	3.565	46.92	0.00	C
ATOM	660	O	GLU	A	165	-15.557	1.484	2.978	46.92	0.00	O
ATOM	661	N	ALA	A	166	-17.251	2.207	4.270	53.66	0.00	N
ATOM	662	CA	ALA	A	166	-17.793	0.874	4.504	53.66	0.00	C
ATOM	663	C	ALA	A	166	-18.098	0.165	3.191	53.66	0.00	C
ATOM	664	O	ALA	A	166	-17.767	-1.008	3.017	53.66	0.00	O
ATOM	665	N	THR	A	167	-18.730	0.883	2.269	56.37	0.00	N
ATOM	666	CA	THR	A	167	-19.137	0.305	0.993	56.37	0.00	C
ATOM	667	C	THR	A	167	-17.929	-0.154	0.187	56.37	0.00	C
ATOM	668	O	THR	A	167	-17.895	-1.277	-0.314	56.37	0.00	O
ATOM	669	N	ILE	A	168	-16.937	0.723	0.065	67.38	0.00	N
ATOM	670	CA	ILE	A	168	-15.758	0.437	-0.742	67.38	0.00	C
ATOM	671	C	ILE	A	168	-14.958	-0.721	-0.158	67.38	0.00	C
ATOM	672	O	ILE	A	168	-14.499	-1.601	-0.887	67.38	0.00	O
ATOM	673	N	VAL	A	169	-14.794	-0.715	1.160	49.35	0.00	N
ATOM	674	CA	VAL	A	169	-14.045	-1.762	1.844	49.35	0.00	C
ATOM	675	C	VAL	A	169	-14.743	-3.110	1.718	49.35	0.00	C
ATOM	676	O	VAL	A	169	-14.100	-4.134	1.488	49.35	0.00	O
ATOM	677	N	ARG	A	170	-16.063	-3.103	1.872	56.01	0.00	N
ATOM	678	CA	ARG	A	170	-16.852	-4.325	1.766	56.01	0.00	C
ATOM	679	C	ARG	A	170	-16.792	-4.899	0.358	56.01	0.00	C
ATOM	680	O	ARG	A	170	-16.685	-6.112	0.175	56.01	0.00	O
ATOM	681	N	LEU	A	171	-16.861	-4.022	-0.637	73.24	0.00	N
ATOM	682	CA	LEU	A	171	-16.757	-4.434	-2.032	73.24	0.00	C
ATOM	683	C	LEU	A	171	-15.373	-4.992	-2.338	73.24	0.00	C
ATOM	684	O	LEU	A	171	-15.235	-5.961	-3.085	73.24	0.00	O
ATOM	685	N	ALA	A	172	-14.350	-4.372	-1.760	49.39	0.00	N
ATOM	686	CA	ALA	A	172	-12.977	-4.828	-1.941	49.39	0.00	C
ATOM	687	C	ALA	A	172	-12.782	-6.228	-1.374	49.39	0.00	C
ATOM	688	O	ALA	A	172	-12.154	-7.080	-2.003	49.39	0.00	O
ATOM	689	N	GLU	A	173	-13.324	-6.460	-0.184	55.60	0.00	N
ATOM	690	CA	GLU	A	173	-13.204	-7.756	0.473	55.60	0.00	C
ATOM	691	C	GLU	A	173	-14.016	-8.820	-0.256	55.60	0.00	C
ATOM	692	O	GLU	A	173	-13.630	-9.988	-0.298	55.60	0.00	O
ATOM	693	N	ASP	A	174	-15.142	-8.408	-0.828	44.03	0.00	N
ATOM	694	CA	ASP	A	174	-15.931	-9.285	-1.685	44.03	0.00	C
ATOM	695	C	ASP	A	174	-15.164	-9.662	-2.944	44.03	0.00	C
ATOM	696	O	ASP	A	174	-15.253	-10.794	-3.423	44.03	0.00	O
ATOM	697	N	THR	A	175	-14.408	-8.708	-3.479	49.85	0.00	N
ATOM	698	CA	THR	A	175	-13.555	-8.963	-4.633	49.85	0.00	C
ATOM	699	C	THR	A	175	-12.449	-9.955	-4.293	49.85	0.00	C
ATOM	700	O	THR	A	175	-12.105	-10.817	-5.101	49.85	0.00	O
ATOM	701	N	ILE	A	176	-11.896	-9.827	-3.091	17.70	0.00	N
ATOM	702	CA	ILE	A	176	-10.885	-10.761	-2.610	17.70	0.00	C
ATOM	703	C	ILE	A	176	-11.434	-12.181	-2.544	17.70	0.00	C
ATOM	704	O	ILE	A	176	-10.776	-13.130	-2.971	17.70	0.00	O
ATOM	705	N	MET	A	177	-12.640	-12.319	-2.008	27.24	0.00	N
ATOM	706	CA	MET	A	177	-13.287	-13.621	-1.902	27.24	0.00	C
ATOM	707	C	MET	A	177	-13.656	-14.166	-3.276	27.24	0.00	C
ATOM	708	O	MET	A	177	-13.596	-15.372	-3.514	27.24	0.00	O
ATOM	709	N	LEU	A	178	-14.039	-13.270	-4.180	45.20	0.00	N
ATOM	710	CA	LEU	A	178	-14.426	-13.659	-5.530	45.20	0.00	C
ATOM	711	C	LEU	A	178	-13.247	-14.252	-6.292	45.20	0.00	C
ATOM	712	O	LEU	A	178	-12.191	-13.630	-6.401	45.20	0.00	O
ATOM	713	N	GLN	A	179	-13.435	-15.458	-6.818	46.50	0.00	N
ATOM	714	CA	GLN	A	179	-12.409	-16.111	-7.622	46.50	0.00	C
ATOM	715	C	GLN	A	179	-12.236	-15.414	-8.965	46.50	0.00	C
ATOM	716	O	GLN	A	179	-11.153	-14.926	-9.287	46.50	0.00	O
ATOM	717	N	ASN	A	180	-13.309	-15.370	-9.747	46.72	0.00	N
ATOM	718	CA	ASN	A	180	-13.291	-14.692	-11.037	46.72	0.00	C
ATOM	719	C	ASN	A	180	-13.454	-13.186	-10.871	46.72	0.00	C
ATOM	720	O	ASN	A	180	-14.491	-12.622	-11.221	46.72	0.00	O

```
ATOM    721  N   SER A 181     -12.424 -12.541 -10.334 59.21  0.00           N
ATOM    722  CA  SER A 181     -12.480 -11.112 -10.049 59.21  0.00           C
ATOM    723  C   SER A 181     -12.416 -10.292 -11.331 59.21  0.00           C
ATOM    724  O   SER A 181     -11.943 -10.770 -12.362 59.21  0.00           O
ATOM    725  N   PRO A 182     -12.896  -9.054 -11.261 65.34  0.00           N
ATOM    726  CA  PRO A 182     -12.893  -8.165 -12.416 65.34  0.00           C
ATOM    727  C   PRO A 182     -11.484  -7.981 -12.967 65.34  0.00           C
ATOM    728  O   PRO A 182     -10.511  -7.956 -12.213 65.34  0.00           O
ATOM    729  N   ARG A 183     -11.381  -7.853 -14.285 81.95  0.00           N
ATOM    730  CA  ARG A 183     -10.105  -7.571 -14.930 81.95  0.00           C
ATOM    731  C   ARG A 183      -9.699  -6.117 -14.734 81.95  0.00           C
ATOM    732  O   ARG A 183     -10.513  -5.208 -14.892 81.95  0.00           O
ATOM    733  N   PHE A 184      -8.432  -5.902 -14.390 48.95  0.00           N
ATOM    734  CA  PHE A 184      -7.909  -4.556 -14.193 48.95  0.00           C
ATOM    735  C   PHE A 184      -7.386  -3.971 -15.498 48.95  0.00           C
ATOM    736  O   PHE A 184      -7.085  -4.704 -16.441 48.95  0.00           O
ATOM    737  N   ASN A 185      -7.280  -2.648 -15.548 52.94  0.00           N
ATOM    738  CA  ASN A 185      -6.819  -1.961 -16.747 52.94  0.00           C
ATOM    739  C   ASN A 185      -5.337  -1.619 -16.651 52.94  0.00           C
ATOM    740  O   ASN A 185      -4.638  -2.094 -15.756 52.94  0.00           O
ATOM    741  N   TRP A 186      -4.864  -0.791 -17.576 49.34  0.00           N
ATOM    742  CA  TRP A 186      -3.440  -0.501 -17.689 49.34  0.00           C
ATOM    743  C   TRP A 186      -2.960   0.365 -16.531 49.34  0.00           C
ATOM    744  O   TRP A 186      -1.761   0.456 -16.266 49.34  0.00           O
ATOM    745  N   GLU A 187      -3.903   1.001 -15.844 69.55  0.00           N
ATOM    746  CA  GLU A 187      -3.586   1.800 -14.667 69.55  0.00           C
ATOM    747  C   GLU A 187      -3.431   0.923 -13.432 69.55  0.00           C
ATOM    748  O   GLU A 187      -4.158   1.081 -12.451 69.55  0.00           O
ATOM    749  N   CYS A 188      -2.478  -0.002 -13.485 43.75  0.00           N
ATOM    750  CA  CYS A 188      -2.372  -1.052 -12.478 43.75  0.00           C
ATOM    751  C   CYS A 188      -2.109  -0.466 -11.097 43.75  0.00           C
ATOM    752  O   CYS A 188      -2.667  -0.925 -10.101 43.75  0.00           O
ATOM    753  N   LYS A 189      -1.254   0.550 -11.045 20.45  0.00           N
ATOM    754  CA  LYS A 189      -0.882   1.174  -9.780 20.45  0.00           C
ATOM    755  C   LYS A 189      -2.008   2.051  -9.247 20.45  0.00           C
ATOM    756  O   LYS A 189      -1.958   2.517  -8.109 20.45  0.00           O
ATOM    757  N   TYR A 190      -3.022   2.272 -10.076 14.03  0.00           N
ATOM    758  CA  TYR A 190      -4.193   3.038  -9.666 14.03  0.00           C
ATOM    759  C   TYR A 190      -5.434   2.156  -9.607 14.03  0.00           C
ATOM    760  O   TYR A 190      -6.556   2.653  -9.496 14.03  0.00           O
ATOM    761  N   CYS A 191      -5.228   0.847  -9.683 35.90  0.00           N
ATOM    762  CA  CYS A 191      -6.283  -0.114  -9.386 35.90  0.00           C
ATOM    763  C   CYS A 191      -6.172  -0.629  -7.957 35.90  0.00           C
ATOM    764  O   CYS A 191      -5.089  -0.638  -7.371 35.90  0.00           O
ATOM    765  N   ILE A 192      -7.299  -1.060  -7.399 22.39  0.00           N
ATOM    766  CA  ILE A 192      -7.340  -1.528  -6.019 22.39  0.00           C
ATOM    767  C   ILE A 192      -6.762  -2.932  -5.895 22.39  0.00           C
ATOM    768  O   ILE A 192      -6.442  -3.387  -4.798 22.39  0.00           O
ATOM    769  N   PHE A 193      -6.630  -3.613  -7.028 23.80  0.00           N
ATOM    770  CA  PHE A 193      -6.141  -4.986  -7.043 23.80  0.00           C
ATOM    771  C   PHE A 193      -4.703  -5.064  -6.544 23.80  0.00           C
ATOM    772  O   PHE A 193      -4.303  -6.051  -5.926 23.80  0.00           O
ATOM    773  N   SER A 194      -3.931  -4.017  -6.813 36.08  0.00           N
ATOM    774  CA  SER A 194      -2.567  -3.920  -6.307 36.08  0.00           C
ATOM    775  C   SER A 194      -2.554  -3.744  -4.793 36.08  0.00           C
ATOM    776  O   SER A 194      -1.510  -3.871  -4.153 36.08  0.00           O
ATOM    777  N   VAL A 195      -3.720  -3.452  -4.226 33.25  0.00           N
ATOM    778  CA  VAL A 195      -3.840  -3.233  -2.789 33.25  0.00           C
ATOM    779  C   VAL A 195      -4.418  -4.457  -2.091 33.25  0.00           C
ATOM    780  O   VAL A 195      -3.967  -4.838  -1.011 33.25  0.00           O
ATOM    781  N   ILE A 196      -5.418  -5.069  -2.714 21.76  0.00           N
ATOM    782  CA  ILE A 196      -6.301  -5.997  -2.018 21.76  0.00           C
ATOM    783  C   ILE A 196      -6.081  -7.428  -2.492 21.76  0.00           C
ATOM    784  O   ILE A 196      -6.479  -8.382  -1.823 21.76  0.00           O
ATOM    785  N   CYS A 197      -5.444  -7.570  -3.650 34.17  0.00           N
ATOM    786  CA  CYS A 197      -5.334  -8.866  -4.310 34.17  0.00           C
ATOM    787  C   CYS A 197      -4.040  -8.971  -5.105 34.17  0.00           C
ATOM    788  O   CYS A 197      -4.016  -8.710  -6.308 34.17  0.00           O
ATOM    789  N   PRO A 198      -2.964  -9.356  -4.427 39.58  0.00           N
ATOM    790  CA  PRO A 198      -1.691  -9.611  -5.090 39.58  0.00           C
ATOM    791  C   PRO A 198      -1.805 -10.768  -6.074 39.58  0.00           C
ATOM    792  O   PRO A 198      -0.934 -10.961  -6.922 39.58  0.00           O
ATOM    793  N   ALA A 199      -2.883 -11.535  -5.955 39.90  0.00           N
ATOM    794  CA  ALA A 199      -3.164 -12.613  -6.896 39.90  0.00           C
ATOM    795  C   ALA A 199      -3.387 -12.072  -8.303 39.90  0.00           C
ATOM    796  O   ALA A 199      -3.230 -12.794  -9.287 39.90  0.00           O
ATOM    797  N   LYS A 200      -3.753 -10.799  -8.390 31.81  0.00           N
ATOM    798  CA  LYS A 200      -3.956 -10.146  -9.679 31.81  0.00           C
ATOM    799  C   LYS A 200      -2.850  -9.139  -9.966 31.81  0.00           C
ATOM    800  O   LYS A 200      -2.251  -9.152 -11.042 31.81  0.00           O
ATOM    801  N   LEU A 201      -2.583  -8.268  -8.999 38.75  0.00           N
ATOM    802  CA  LEU A 201      -1.463  -7.340  -9.093 38.75  0.00           C
ATOM    803  C   LEU A 201      -0.515  -7.499  -7.911 38.75  0.00           C
ATOM    804  O   LEU A 201      -0.675  -6.843  -6.881 38.75  0.00           O
ATOM    805  N   THR A 202       0.473  -8.373  -8.066 45.33  0.00           N
ATOM    806  CA  THR A 202       1.411  -8.668  -6.989 45.33  0.00           C
ATOM    807  C   THR A 202       2.192  -7.425  -6.584 45.33  0.00           C
ATOM    808  O   THR A 202       2.133  -7.081  -5.647 45.33  0.00           O
TER     809      THR A 202
ENDMDL
END
REMARK   1 ***********************************************************************
```

```
REMARK   1 Start File DEC5_vs_NAT_dSi_colored.pdb
REMARK   1 The occupancy record was modified to represent dSi := Si(DEC)-Si(NAT).
REMARK   1 Occ=0.00 means dSi=-1.555227 kB; Occ=99.99 means dSi=1.594793 kB.
MODEL        0
ATOM      1  N   MET A   1      20.279  17.173 -18.693 45.76  0.00           N
ATOM      2  CA  MET A   1      19.053  16.399 -18.542 45.76  0.00           C
ATOM      3  C   MET A   1      19.026  15.664 -17.208 45.76  0.00           C
ATOM      4  O   MET A   1      19.795  14.728 -16.988 45.76  0.00           O
ATOM      5  N   ILE A   2      18.137  16.093 -16.319 46.21  0.00           N
ATOM      6  CA  ILE A   2      18.045  15.512 -14.985 46.21  0.00           C
ATOM      7  C   ILE A   2      17.569  14.066 -15.046 46.21  0.00           C
ATOM      8  O   ILE A   2      18.151  13.184 -14.414 46.21  0.00           O
ATOM      9  N   THR A   3      16.508  13.829 -15.810 45.10  0.00           N
ATOM     10  CA  THR A   3      15.931  12.495 -15.928 45.10  0.00           C
ATOM     11  C   THR A   3      16.921  11.521 -16.552 45.10  0.00           C
ATOM     12  O   THR A   3      17.057  10.384 -16.100 45.10  0.00           O
ATOM     13  N   GLU A   4      17.614  11.972 -17.592 48.31  0.00           N
ATOM     14  CA  GLU A   4      18.623  11.156 -18.256 48.31  0.00           C
ATOM     15  C   GLU A   4      19.739  10.771 -17.293 48.31  0.00           C
ATOM     16  O   GLU A   4      20.094   9.598 -17.176 48.31  0.00           O
ATOM     17  N   PHE A   5      20.289  11.766 -16.604 48.37  0.00           N
ATOM     18  CA  PHE A   5      21.387  11.537 -15.672 48.37  0.00           C
ATOM     19  C   PHE A   5      20.934  10.700 -14.483 48.37  0.00           C
ATOM     20  O   PHE A   5      21.720   9.950 -13.905 48.37  0.00           O
ATOM     21  N   LEU A   6      19.662  10.834 -14.122 36.80  0.00           N
ATOM     22  CA  LEU A   6      19.076  10.018 -13.065 36.80  0.00           C
ATOM     23  C   LEU A   6      19.100   8.540 -13.433 36.80  0.00           C
ATOM     24  O   LEU A   6      19.575   7.706 -12.661 36.80  0.00           O
ATOM     25  N   LEU A   7      18.586   8.222 -14.616 54.72  0.00           N
ATOM     26  CA  LEU A   7      18.351   6.835 -15.001 54.72  0.00           C
ATOM     27  C   LEU A   7      19.604   6.209 -15.601 54.72  0.00           C
ATOM     28  O   LEU A   7      19.700   4.989 -15.730 54.72  0.00           O
ATOM     29  N   LYS A   8      20.564   7.053 -15.966 51.73  0.00           N
ATOM     30  CA  LYS A   8      21.818   6.585 -16.543 51.73  0.00           C
ATOM     31  C   LYS A   8      22.928   6.555 -15.500 51.73  0.00           C
ATOM     32  O   LYS A   8      23.607   5.543 -15.332 51.73  0.00           O
ATOM     33  N   LYS A   9      23.107   7.672 -14.803 52.97  0.00           N
ATOM     34  CA  LYS A   9      24.252   7.846 -13.917 52.97  0.00           C
ATOM     35  C   LYS A   9      23.899   7.474 -12.482 52.97  0.00           C
ATOM     36  O   LYS A   9      24.508   6.581 -11.895 52.97  0.00           O
ATOM     37  N   LYS A  10      22.911   8.165 -11.923 52.55  0.00           N
ATOM     38  CA  LYS A  10      22.539   7.975 -10.526 52.55  0.00           C
ATOM     39  C   LYS A  10      22.049   6.555 -10.274 52.55  0.00           C
ATOM     40  O   LYS A  10      22.421   5.927  -9.283 52.55  0.00           O
ATOM     41  N   LEU A  11      21.213   6.054 -11.177 38.88  0.00           N
ATOM     42  CA  LEU A  11      20.689   4.698 -11.066 38.88  0.00           C
ATOM     43  C   LEU A  11      21.810   3.690 -10.846 38.88  0.00           C
ATOM     44  O   LEU A  11      21.823   2.970  -9.848 38.88  0.00           O
ATOM     45  N   GLU A  12      22.749   3.644 -11.784 45.02  0.00           N
ATOM     46  CA  GLU A  12      23.853   2.693 -11.717 45.02  0.00           C
ATOM     47  C   GLU A  12      24.729   2.952 -10.499 45.02  0.00           C
ATOM     48  O   GLU A  12      25.220   2.018  -9.866 45.02  0.00           O
ATOM     49  N   GLU A  13      24.924   4.226 -10.177 49.97  0.00           N
ATOM     50  CA  GLU A  13      25.705   4.609  -9.006 49.97  0.00           C
ATOM     51  C   GLU A  13      25.135   3.991  -7.736 49.97  0.00           C
ATOM     52  O   GLU A  13      25.878   3.535  -6.869 49.97  0.00           O
ATOM     53  N   HIS A  14      23.809   3.980  -7.633 31.53  0.00           N
ATOM     54  CA  HIS A  14      23.139   3.523  -6.422 31.53  0.00           C
ATOM     55  C   HIS A  14      22.904   2.018  -6.458 31.53  0.00           C
ATOM     56  O   HIS A  14      22.830   1.367  -5.416 31.53  0.00           O
ATOM     57  N   LEU A  15      22.788   1.470  -7.662 13.88  0.00           N
ATOM     58  CA  LEU A  15      22.660   0.029  -7.840 13.88  0.00           C
ATOM     59  C   LEU A  15      23.854  -0.707  -7.243 13.88  0.00           C
ATOM     60  O   LEU A  15      23.712  -1.809  -6.713 13.88  0.00           O
ATOM     61  N   SER A  16      25.027  -0.091  -7.332 30.48  0.00           N
ATOM     62  CA  SER A  16      26.241  -0.671  -6.771 30.48  0.00           C
ATOM     63  C   SER A  16      26.212  -0.642  -5.248 30.48  0.00           C
ATOM     64  O   SER A  16      27.006  -1.315  -4.590 30.48  0.00           O
ATOM     65  N   HIS A  17      25.294   0.141  -4.694 47.88  0.00           N
ATOM     66  CA  HIS A  17      25.177   0.279  -3.247 47.88  0.00           C
ATOM     67  C   HIS A  17      23.848  -0.273  -2.746 47.88  0.00           C
ATOM     68  O   HIS A  17      23.628  -0.388  -1.542 47.88  0.00           O
ATOM     69  N   VAL A  18      22.967  -0.615  -3.680 27.03  0.00           N
ATOM     70  CA  VAL A  18      21.659  -1.161  -3.336 27.03  0.00           C
ATOM     71  C   VAL A  18      21.486  -2.568  -3.892 27.03  0.00           C
ATOM     72  O   VAL A  18      21.189  -3.507  -3.154 27.03  0.00           O
ATOM     73  N   LYS A  19      21.673  -2.708  -5.201 25.84  0.00           N
ATOM     74  CA  LYS A  19      21.480  -3.989  -5.870 25.84  0.00           C
ATOM     75  C   LYS A  19      22.601  -4.963  -5.531 25.84  0.00           C
ATOM     76  O   LYS A  19      22.350  -6.105  -5.147 25.84  0.00           O
ATOM     77  N   GLU A  20      23.840  -4.504  -5.675 52.41  0.00           N
ATOM     78  CA  GLU A  20      25.003  -5.321  -5.351 52.41  0.00           C
ATOM     79  C   GLU A  20      25.103  -5.566  -3.850 52.41  0.00           C
ATOM     80  O   GLU A  20      25.664  -6.571  -3.413 52.41  0.00           O
ATOM     81  N   GLU A  21      24.555  -4.644  -3.067 47.67  0.00           N
ATOM     82  CA  GLU A  21      24.571  -4.764  -1.615 47.67  0.00           C
ATOM     83  C   GLU A  21      23.285  -5.397  -1.099 47.67  0.00           C
ATOM     84  O   GLU A  21      22.457  -5.866  -1.879 47.67  0.00           O
ATOM     85  N   ASN A  22      23.123  -5.406   0.220 55.84  0.00           N
ATOM     86  CA  ASN A  22      21.929  -5.965   0.841 55.84  0.00           C
ATOM     87  C   ASN A  22      20.706  -5.100   0.560 55.84  0.00           C
ATOM     88  O   ASN A  22      20.315  -4.274   1.385 55.84  0.00           O
```

ATOM	89	N	THR	A	23	20.106	-5.296	-0.609	52.17	0.00	N
ATOM	90	CA	THR	A	23	18.926	-4.534	-1.001	52.17	0.00	C
ATOM	91	C	THR	A	23	17.928	-4.436	0.144	52.17	0.00	C
ATOM	92	O	THR	A	23	17.601	-5.437	0.784	52.17	0.00	O
ATOM	93	N	ILE	A	24	17.444	-3.225	0.399	52.05	0.00	N
ATOM	94	CA	ILE	A	24	16.486	-2.993	1.473	52.05	0.00	C
ATOM	95	C	ILE	A	24	15.077	-3.383	1.047	52.05	0.00	C
ATOM	96	O	ILE	A	24	14.474	-2.733	0.191	52.05	0.00	O
ATOM	97	N	TYR	A	25	14.555	-4.446	1.649	49.96	0.00	N
ATOM	98	CA	TYR	A	25	13.183	-4.872	1.398	49.96	0.00	C
ATOM	99	C	TYR	A	25	12.277	-4.531	2.574	49.96	0.00	C
ATOM	100	O	TYR	A	25	12.745	-4.092	3.624	49.96	0.00	O
ATOM	101	N	VAL	A	26	10.977	-4.736	2.391	44.54	0.00	N
ATOM	102	CA	VAL	A	26	10.004	-4.469	3.443	44.54	0.00	C
ATOM	103	C	VAL	A	26	10.394	-5.163	4.742	44.54	0.00	C
ATOM	104	O	VAL	A	26	10.461	-4.533	5.798	44.54	0.00	O
ATOM	105	N	THR	A	27	10.651	-6.464	4.658	50.31	0.00	N
ATOM	106	CA	THR	A	27	11.001	-7.253	5.833	50.31	0.00	C
ATOM	107	C	THR	A	27	12.260	-6.713	6.503	50.31	0.00	C
ATOM	108	O	THR	A	27	12.347	-6.660	7.729	50.31	0.00	O
ATOM	109	N	ASP	A	28	13.232	-6.314	5.690	54.19	0.00	N
ATOM	110	CA	ASP	A	28	14.472	-5.744	6.202	54.19	0.00	C
ATOM	111	C	ASP	A	28	14.197	-4.556	7.115	54.19	0.00	C
ATOM	112	O	ASP	A	28	14.839	-4.398	8.153	54.19	0.00	O
ATOM	113	N	LEU	A	29	13.239	-3.723	6.721	58.99	0.00	N
ATOM	114	CA	LEU	A	29	12.855	-2.566	7.521	58.99	0.00	C
ATOM	115	C	LEU	A	29	12.069	-2.987	8.756	58.99	0.00	C
ATOM	116	O	LEU	A	29	12.303	-2.483	9.855	58.99	0.00	O
ATOM	117	N	VAL	A	30	11.135	-3.914	8.570	54.59	0.00	N
ATOM	118	CA	VAL	A	30	10.297	-4.387	9.665	54.59	0.00	C
ATOM	119	C	VAL	A	30	11.142	-4.871	10.837	54.59	0.00	C
ATOM	120	O	VAL	A	30	10.808	-4.629	11.997	54.59	0.00	O
ATOM	121	N	ARG	A	31	12.237	-5.556	10.527	99.99	0.00	N
ATOM	122	CA	ARG	A	31	13.064	-6.184	11.551	99.99	0.00	C
ATOM	123	C	ARG	A	31	13.808	-5.141	12.374	99.99	0.00	C
ATOM	124	O	ARG	A	31	14.328	-5.441	13.450	99.99	0.00	O
ATOM	125	N	CYS	A	32	13.856	-3.916	11.864	79.02	0.00	N
ATOM	126	CA	CYS	A	32	14.565	-2.833	12.536	79.02	0.00	C
ATOM	127	C	CYS	A	32	13.592	-1.801	13.093	79.02	0.00	C
ATOM	128	O	CYS	A	32	12.429	-1.752	12.695	79.02	0.00	O
ATOM	129	N	PRO	A	33	14.077	-0.978	14.017	65.88	0.00	N
ATOM	130	CA	PRO	A	33	13.279	0.112	14.565	65.88	0.00	C
ATOM	131	C	PRO	A	33	13.074	1.216	13.537	65.88	0.00	C
ATOM	132	O	PRO	A	33	13.844	1.343	12.585	65.88	0.00	O
ATOM	133	N	ARG	A	34	12.030	2.015	13.734	59.12	0.00	N
ATOM	134	CA	ARG	A	34	11.645	3.027	12.757	59.12	0.00	C
ATOM	135	C	ARG	A	34	12.767	4.035	12.536	59.12	0.00	C
ATOM	136	O	ARG	A	34	12.922	4.574	11.441	59.12	0.00	O
ATOM	137	N	ARG	A	35	13.546	4.284	13.584	93.15	0.00	N
ATOM	138	CA	ARG	A	35	14.731	5.125	13.473	93.15	0.00	C
ATOM	139	C	ARG	A	35	15.690	4.590	12.418	93.15	0.00	C
ATOM	140	O	ARG	A	35	16.103	5.317	11.514	93.15	0.00	O
ATOM	141	N	VAL	A	36	16.041	3.314	12.537	55.58	0.00	N
ATOM	142	CA	VAL	A	36	16.929	2.671	11.575	55.58	0.00	C
ATOM	143	C	VAL	A	36	16.278	2.579	10.201	55.58	0.00	C
ATOM	144	O	VAL	A	36	16.957	2.649	9.176	55.58	0.00	O
ATOM	145	N	ARG	A	37	14.959	2.421	10.186	20.52	0.00	N
ATOM	146	CA	ARG	A	37	14.208	2.368	8.937	20.52	0.00	C
ATOM	147	C	ARG	A	37	14.417	3.633	8.113	20.52	0.00	C
ATOM	148	O	ARG	A	37	14.613	3.570	6.900	20.52	0.00	O
ATOM	149	N	TYR	A	38	14.374	4.781	8.781	54.45	0.00	N
ATOM	150	CA	TYR	A	38	14.611	6.060	8.122	54.45	0.00	C
ATOM	151	C	TYR	A	38	16.074	6.212	7.725	54.45	0.00	C
ATOM	152	O	TYR	A	38	16.385	6.673	6.627	54.45	0.00	O
ATOM	153	N	GLU	A	39	16.969	5.821	8.626	73.98	0.00	N
ATOM	154	CA	GLU	A	39	18.402	5.959	8.391	73.98	0.00	C
ATOM	155	C	GLU	A	39	18.842	5.154	7.175	73.98	0.00	C
ATOM	156	O	GLU	A	39	19.711	5.582	6.416	73.98	0.00	O
ATOM	157	N	SER	A	40	18.235	3.985	6.996	37.54	0.00	N
ATOM	158	CA	SER	A	40	18.606	3.089	5.906	37.54	0.00	C
ATOM	159	C	SER	A	40	18.329	3.727	4.550	37.54	0.00	C
ATOM	160	O	SER	A	40	18.923	3.346	3.542	37.54	0.00	O
ATOM	161	N	GLU	A	41	17.425	4.702	4.534	2.00	0.00	N
ATOM	162	CA	GLU	A	41	17.068	5.394	3.302	2.00	0.00	C
ATOM	163	C	GLU	A	41	18.167	6.357	2.873	2.00	0.00	C
ATOM	164	O	GLU	A	41	18.262	6.724	1.701	2.00	0.00	O
ATOM	165	N	TYR	A	42	18.994	6.766	3.828	52.16	0.00	N
ATOM	166	CA	TYR	A	42	20.038	7.751	3.568	52.16	0.00	C
ATOM	167	C	TYR	A	42	21.424	7.157	3.786	52.16	0.00	C
ATOM	168	O	TYR	A	42	22.435	7.790	3.483	52.16	0.00	O
ATOM	169	N	LYS	A	43	21.463	5.938	4.312	61.43	0.00	N
ATOM	170	CA	LYS	A	43	22.722	5.235	4.523	61.43	0.00	C
ATOM	171	C	LYS	A	43	22.929	4.149	3.475	61.43	0.00	C
ATOM	172	O	LYS	A	43	24.025	3.995	2.935	61.43	0.00	O
ATOM	173	N	GLU	A	44	21.871	3.398	3.192	40.87	0.00	N
ATOM	174	CA	GLU	A	44	21.942	2.305	2.229	40.87	0.00	C
ATOM	175	C	GLU	A	44	21.383	2.726	0.875	40.87	0.00	C
ATOM	176	O	GLU	A	44	22.007	2.502	-0.161	40.87	0.00	O
ATOM	177	N	LEU	A	45	20.203	3.337	0.893	34.19	0.00	N
ATOM	178	CA	LEU	A	45	19.525	3.727	-0.337	34.19	0.00	C
ATOM	179	C	LEU	A	45	20.014	5.084	-0.828	34.19	0.00	C
ATOM	180	O	LEU	A	45	20.023	5.355	-2.029	34.19	0.00	O

```
ATOM    181  N   ALA A  46      20.419   5.935   0.108 56.47  0.00           N
ATOM    182  CA  ALA A  46      20.983   7.237  -0.232 56.47  0.00           C
ATOM    183  C   ALA A  46      20.043   8.025  -1.135 56.47  0.00           C
ATOM    184  O   ALA A  46      20.452   8.532  -2.180 56.47  0.00           O
ATOM    185  N   ILE A  47      18.783   8.126  -0.726 44.14  0.00           N
ATOM    186  CA  ILE A  47      17.759   8.757  -1.549 44.14  0.00           C
ATOM    187  C   ILE A  47      17.930  10.269  -1.577 44.14  0.00           C
ATOM    188  O   ILE A  47      17.333  10.956  -2.407 44.14  0.00           O
ATOM    189  N   SER A  48      18.748  10.785  -0.666 56.13  0.00           N
ATOM    190  CA  SER A  48      19.074  12.206  -0.646 56.13  0.00           C
ATOM    191  C   SER A  48      19.890  12.602  -1.870 56.13  0.00           C
ATOM    192  O   SER A  48      19.992  13.781  -2.205 56.13  0.00           O
ATOM    193  N   GLN A  49      20.467  11.607  -2.536 63.24  0.00           N
ATOM    194  CA  GLN A  49      21.241  11.846  -3.749 63.24  0.00           C
ATOM    195  C   GLN A  49      20.479  11.388  -4.986 63.24  0.00           C
ATOM    196  O   GLN A  49      21.056  11.246  -6.065 63.24  0.00           O
ATOM    197  N   VAL A  50      19.180  11.159  -4.825 56.27  0.00           N
ATOM    198  CA  VAL A  50      18.340  10.703  -5.925 56.27  0.00           C
ATOM    199  C   VAL A  50      17.291  11.747  -6.287 56.27  0.00           C
ATOM    200  O   VAL A  50      16.582  12.256  -5.418 56.27  0.00           O
ATOM    201  N   TYR A  51      17.197  12.062  -7.573 45.04  0.00           N
ATOM    202  CA  TYR A  51      16.254  13.069  -8.049 45.04  0.00           C
ATOM    203  C   TYR A  51      14.815  12.599  -7.879 45.04  0.00           C
ATOM    204  O   TYR A  51      13.941  13.375  -7.493 45.04  0.00           O
ATOM    205  N   ALA A  52      14.576  11.325  -8.169 44.86  0.00           N
ATOM    206  CA  ALA A  52      13.257  10.731  -7.980 44.86  0.00           C
ATOM    207  C   ALA A  52      13.361   9.347  -7.352 44.86  0.00           C
ATOM    208  O   ALA A  52      13.360   8.335  -8.051 44.86  0.00           O
ATOM    209  N   PRO A  53      13.451   9.311  -6.027 43.70  0.00           N
ATOM    210  CA  PRO A  53      13.607   8.054  -5.303 43.70  0.00           C
ATOM    211  C   PRO A  53      12.506   7.067  -5.670 43.70  0.00           C
ATOM    212  O   PRO A  53      12.726   5.856  -5.695 43.70  0.00           O
ATOM    213  N   SER A  54      11.318   7.593  -5.956 42.78  0.00           N
ATOM    214  CA  SER A  54      10.196   6.763  -6.380 42.78  0.00           C
ATOM    215  C   SER A  54      10.522   6.009  -7.663 42.78  0.00           C
ATOM    216  O   SER A  54      10.183   4.835  -7.807 42.78  0.00           O
ATOM    217  N   ALA A  55      11.184   6.691  -8.592 45.72  0.00           N
ATOM    218  CA  ALA A  55      11.513   6.103  -9.885 45.72  0.00           C
ATOM    219  C   ALA A  55      12.622   5.067  -9.754 45.72  0.00           C
ATOM    220  O   ALA A  55      12.538   3.977 -10.319 45.72  0.00           O
ATOM    221  N   ILE A  56      13.662   5.415  -9.004 37.37  0.00           N
ATOM    222  CA  ILE A  56      14.825   4.548  -8.857 37.37  0.00           C
ATOM    223  C   ILE A  56      14.493   3.317  -8.023 37.37  0.00           C
ATOM    224  O   ILE A  56      14.739   2.186  -8.441 37.37  0.00           O
ATOM    225  N   LEU A  57      13.932   3.545  -6.840 21.14  0.00           N
ATOM    226  CA  LEU A  57      13.512   2.454  -5.968 21.14  0.00           C
ATOM    227  C   LEU A  57      12.328   1.699  -6.561 21.14  0.00           C
ATOM    228  O   LEU A  57      12.154   0.506  -6.315 21.14  0.00           O
ATOM    229  N   GLY A  58      11.517   2.403  -7.343 40.76  0.00           N
ATOM    230  CA  GLY A  58      10.409   1.779  -8.056 40.76  0.00           C
ATOM    231  C   GLY A  58      10.902   0.670  -8.976 40.76  0.00           C
ATOM    232  O   GLY A  58      10.363  -0.437  -8.974 40.76  0.00           O
ATOM    233  N   ASP A  59      11.929   0.972  -9.762 51.13  0.00           N
ATOM    234  CA  ASP A  59      12.511  -0.005 -10.674 51.13  0.00           C
ATOM    235  C   ASP A  59      13.190  -1.134  -9.909 51.13  0.00           C
ATOM    236  O   ASP A  59      13.005  -2.310 -10.225 51.13  0.00           O
ATOM    237  N   ILE A  60      13.980  -0.770  -8.905 29.56  0.00           N
ATOM    238  CA  ILE A  60      14.739  -1.747  -8.134 29.56  0.00           C
ATOM    239  C   ILE A  60      13.814  -2.718  -7.412 29.56  0.00           C
ATOM    240  O   ILE A  60      14.056  -3.925  -7.396 29.56  0.00           O
ATOM    241  N   LEU A  61      12.753  -2.185  -6.816 26.41  0.00           N
ATOM    242  CA  LEU A  61      11.835  -2.990  -6.020 26.41  0.00           C
ATOM    243  C   LEU A  61      10.872  -3.768  -6.908 26.41  0.00           C
ATOM    244  O   LEU A  61      10.434  -4.863  -6.556 26.41  0.00           O
ATOM    245  N   HIS A  62      10.545  -3.195  -8.061 53.85  0.00           N
ATOM    246  CA  HIS A  62       9.725  -3.881  -9.052 53.85  0.00           C
ATOM    247  C   HIS A  62      10.354  -5.205  -9.466 53.85  0.00           C
ATOM    248  O   HIS A  62       9.667  -6.220  -9.585 53.85  0.00           O
ATOM    249  N   LEU A  63      11.665  -5.188  -9.687 53.09  0.00           N
ATOM    250  CA  LEU A  63      12.387  -6.385 -10.099 53.09  0.00           C
ATOM    251  C   LEU A  63      12.630  -7.316  -8.917 53.09  0.00           C
ATOM    252  O   LEU A  63      12.680  -8.537  -9.077 53.09  0.00           O
ATOM    253  N   GLY A  64      12.780  -6.734  -7.732 47.77  0.00           N
ATOM    254  CA  GLY A  64      13.088  -7.505  -6.534 47.77  0.00           C
ATOM    255  C   GLY A  64      11.864  -7.642  -5.638 47.77  0.00           C
ATOM    256  O   GLY A  64      11.247  -8.705  -5.572 47.77  0.00           O
ATOM    257  N   LEU A  65      11.518  -6.560  -4.949 26.44  0.00           N
ATOM    258  CA  LEU A  65      10.537  -6.620  -3.871 26.44  0.00           C
ATOM    259  C   LEU A  65       9.259  -7.311  -4.327 26.44  0.00           C
ATOM    260  O   LEU A  65       8.707  -8.149  -3.613 26.44  0.00           O
ATOM    261  N   GLU A  66       8.792  -6.956  -5.519 54.44  0.00           N
ATOM    262  CA  GLU A  66       7.540  -7.490  -6.042 54.44  0.00           C
ATOM    263  C   GLU A  66       7.419  -8.983  -5.762 54.44  0.00           C
ATOM    264  O   GLU A  66       6.398  -9.447  -5.255 54.44  0.00           O
ATOM    265  N   SER A  67       8.465  -9.730  -6.096 47.72  0.00           N
ATOM    266  CA  SER A  67       8.449 -11.180  -5.950 47.72  0.00           C
ATOM    267  C   SER A  67       8.604 -11.589  -4.491 47.72  0.00           C
ATOM    268  O   SER A  67       8.163 -12.665  -4.087 47.72  0.00           O
ATOM    269  N   VAL A  68       9.234 -10.724  -3.703 48.52  0.00           N
ATOM    270  CA  VAL A  68       9.393 -10.964  -2.274 48.52  0.00           C
ATOM    271  C   VAL A  68       8.054 -10.889  -1.550 48.52  0.00           C
ATOM    272  O   VAL A  68       7.738 -11.740  -0.720 48.52  0.00           O
```

```
ATOM    273  N   LEU A  69       7.271  -9.866  -1.872 40.41  0.00           N
ATOM    274  CA  LEU A  69       5.980  -9.656  -1.225 40.41  0.00           C
ATOM    275  C   LEU A  69       4.946 -10.658  -1.723 40.41  0.00           C
ATOM    276  O   LEU A  69       4.231 -11.271  -0.931 40.41  0.00           O
ATOM    277  N   LYS A  70       4.871 -10.819  -3.040 41.93  0.00           N
ATOM    278  CA  LYS A  70       3.845 -11.654  -3.652 41.93  0.00           C
ATOM    279  C   LYS A  70       4.027 -13.118  -3.272 41.93  0.00           C
ATOM    280  O   LYS A  70       3.078 -13.901  -3.308 41.93  0.00           O
ATOM    281  N   GLY A  71       5.252 -13.481  -2.907 46.57  0.00           N
ATOM    282  CA  GLY A  71       5.552 -14.843  -2.481 46.57  0.00           C
ATOM    283  C   GLY A  71       5.089 -15.087  -1.051 46.57  0.00           C
ATOM    284  O   GLY A  71       5.055 -16.226  -0.584 46.57  0.00           O
ATOM    285  N   ASN A  72       4.732 -14.010  -0.358 46.68  0.00           N
ATOM    286  CA  ASN A  72       4.356 -14.095   1.049 46.68  0.00           C
ATOM    287  C   ASN A  72       2.928 -13.609   1.267 46.68  0.00           C
ATOM    288  O   ASN A  72       2.463 -13.510   2.402 46.68  0.00           O
ATOM    289  N   PHE A  73       2.237 -13.309   0.172 43.70  0.00           N
ATOM    290  CA  PHE A  73       0.848 -12.873   0.239 43.70  0.00           C
ATOM    291  C   PHE A  73      -0.068 -14.014   0.662 43.70  0.00           C
ATOM    292  O   PHE A  73       0.091 -15.149   0.213 43.70  0.00           O
ATOM    293  N   ASN A  74      -1.028 -13.707   1.528 53.08  0.00           N
ATOM    294  CA  ASN A  74      -1.947 -14.714   2.043 53.08  0.00           C
ATOM    295  C   ASN A  74      -3.373 -14.448   1.577 53.08  0.00           C
ATOM    296  O   ASN A  74      -4.208 -15.353   1.556 53.08  0.00           O
ATOM    297  N   ALA A  75      -3.647 -13.203   1.205 38.69  0.00           N
ATOM    298  CA  ALA A  75      -4.928 -12.844   0.611 38.69  0.00           C
ATOM    299  C   ALA A  75      -4.804 -11.600  -0.259 38.69  0.00           C
ATOM    300  O   ALA A  75      -4.085 -10.661   0.084 38.69  0.00           O
ATOM    301  N   GLU A  76      -5.508 -11.600  -1.385 45.22  0.00           N
ATOM    302  CA  GLU A  76      -5.538 -10.440  -2.269 45.22  0.00           C
ATOM    303  C   GLU A  76      -6.868 -10.345  -3.008 45.22  0.00           C
ATOM    304  O   GLU A  76      -7.166 -11.164  -3.876 45.22  0.00           O
ATOM    305  N   THR A  77      -7.662  -9.340  -2.657 56.43  0.00           N
ATOM    306  CA  THR A  77      -9.011  -9.206  -3.194 56.43  0.00           C
ATOM    307  C   THR A  77      -9.336  -7.751  -3.510 56.43  0.00           C
ATOM    308  O   THR A  77      -9.064  -6.857  -2.710 56.43  0.00           O
ATOM    309  N   GLU A  78      -9.920  -7.522  -4.681 57.47  0.00           N
ATOM    310  CA  GLU A  78     -10.337  -6.183  -5.080 57.47  0.00           C
ATOM    311  C   GLU A  78     -11.740  -5.870  -4.577 57.47  0.00           C
ATOM    312  O   GLU A  78     -12.623  -6.728  -4.594 57.47  0.00           O
ATOM    313  N   VAL A  79     -11.942  -4.635  -4.131 35.16  0.00           N
ATOM    314  CA  VAL A  79     -13.246  -4.198  -3.647 35.16  0.00           C
ATOM    315  C   VAL A  79     -13.470  -2.718  -3.927 35.16  0.00           C
ATOM    316  O   VAL A  79     -12.557  -1.904  -3.785 35.16  0.00           O
ATOM    317  N   GLU A  80     -14.689  -2.375  -4.328 42.23  0.00           N
ATOM    318  CA  GLU A  80     -15.090  -0.978  -4.456 42.23  0.00           C
ATOM    319  C   GLU A  80     -15.319  -0.343  -3.091 42.23  0.00           C
ATOM    320  O   GLU A  80     -16.112  -0.837  -2.291 42.23  0.00           O
ATOM    321  N   THR A  81     -14.618   0.756  -2.831 32.21  0.00           N
ATOM    322  CA  THR A  81     -14.666   1.403  -1.525 32.21  0.00           C
ATOM    323  C   THR A  81     -15.248   2.807  -1.626 32.21  0.00           C
ATOM    324  O   THR A  81     -15.247   3.414  -2.698 32.21  0.00           O
ATOM    325  N   LEU A  82     -15.746   3.318  -0.506 30.57  0.00           N
ATOM    326  CA  LEU A  82     -16.377   4.633  -0.477 30.57  0.00           C
ATOM    327  C   LEU A  82     -15.918   5.437   0.732 30.57  0.00           C
ATOM    328  O   LEU A  82     -16.029   4.983   1.871 30.57  0.00           O
ATOM    329  N   ARG A  83     -15.403   6.635   0.479 43.98  0.00           N
ATOM    330  CA  ARG A  83     -14.987   7.533   1.551 43.98  0.00           C
ATOM    331  C   ARG A  83     -15.441   8.962   1.279 43.98  0.00           C
ATOM    332  O   ARG A  83     -15.627   9.355   0.128 43.98  0.00           O
ATOM    333  N   GLU A  84     -15.616   9.734   2.345 48.79  0.00           N
ATOM    334  CA  GLU A  84     -16.001  11.135   2.222 48.79  0.00           C
ATOM    335  C   GLU A  84     -14.786  12.049   2.305 48.79  0.00           C
ATOM    336  O   GLU A  84     -14.024  12.000   3.272 48.79  0.00           O
ATOM    337  N   ILE A  85     -14.608  12.883   1.286 54.30  0.00           N
ATOM    338  CA  ILE A  85     -13.444  13.758   1.206 54.30  0.00           C
ATOM    339  C   ILE A  85     -13.855  15.197   0.921 54.30  0.00           C
ATOM    340  O   ILE A  85     -14.708  15.452   0.071 54.30  0.00           O
ATOM    341  N   ASN A  86     -13.243  16.134   1.637 49.78  0.00           N
ATOM    342  CA  ASN A  86     -13.487  17.554   1.408 49.78  0.00           C
ATOM    343  C   ASN A  86     -12.820  18.028   0.124 49.78  0.00           C
ATOM    344  O   ASN A  86     -11.610  18.251   0.087 49.78  0.00           O
ATOM    345  N   VAL A  87     -13.616  18.180  -0.929 52.65  0.00           N
ATOM    346  CA  VAL A  87     -13.102  18.618  -2.221 52.65  0.00           C
ATOM    347  C   VAL A  87     -13.692  19.964  -2.622 52.65  0.00           C
ATOM    348  O   VAL A  87     -14.906  20.102  -2.765 52.65  0.00           O
ATOM    349  N   GLY A  88     -12.825  20.955  -2.802 48.80  0.00           N
ATOM    350  CA  GLY A  88     -13.253  22.277  -3.241 48.80  0.00           C
ATOM    351  C   GLY A  88     -13.935  23.038  -2.111 48.80  0.00           C
ATOM    352  O   GLY A  88     -14.637  24.022  -2.348 48.80  0.00           O
ATOM    353  N   GLY A  89     -13.724  22.578  -0.883 48.31  0.00           N
ATOM    354  CA  GLY A  89     -14.297  23.230   0.288 48.31  0.00           C
ATOM    355  C   GLY A  89     -15.596  22.556   0.715 48.31  0.00           C
ATOM    356  O   GLY A  89     -16.224  22.961   1.692 48.31  0.00           O
ATOM    357  N   LYS A  90     -15.991  21.526  -0.025 50.78  0.00           N
ATOM    358  CA  LYS A  90     -17.207  20.783   0.286 50.78  0.00           C
ATOM    359  C   LYS A  90     -16.946  19.282   0.301 50.78  0.00           C
ATOM    360  O   LYS A  90     -16.261  18.752  -0.574 50.78  0.00           O
ATOM    361  N   VAL A  91     -17.496  18.601   1.301 52.58  0.00           N
ATOM    362  CA  VAL A  91     -17.302  17.164   1.447 52.58  0.00           C
ATOM    363  C   VAL A  91     -18.195  16.387   0.487 52.58  0.00           C
ATOM    364  O   VAL A  91     -19.415  16.549   0.492 52.58  0.00           O
```

ATOM	365	N	TYR	A	92	-17.579	15.544	-0.334	65.64	0.00	N
ATOM	366	CA	TYR	A	92	-18.319	14.720	-1.282	65.64	0.00	C
ATOM	367	C	TYR	A	92	-18.048	13.239	-1.054	65.64	0.00	C
ATOM	368	O	TYR	A	92	-16.952	12.854	-0.645	65.64	0.00	O
ATOM	369	N	LYS	A	93	-19.052	12.410	-1.319	72.23	0.00	N
ATOM	370	CA	LYS	A	93	-18.878	10.963	-1.298	72.23	0.00	C
ATOM	371	C	LYS	A	93	-18.201	10.470	-2.570	72.23	0.00	C
ATOM	372	O	LYS	A	93	-18.718	10.656	-3.672	72.23	0.00	O
ATOM	373	N	ILE	A	94	-17.041	9.841	-2.411	56.66	0.00	N
ATOM	374	CA	ILE	A	94	-16.238	9.417	-3.552	56.66	0.00	C
ATOM	375	C	ILE	A	94	-15.970	7.918	-3.510	56.66	0.00	C
ATOM	376	O	ILE	A	94	-15.439	7.400	-2.527	56.66	0.00	O
ATOM	377	N	LYS	A	95	-16.341	7.226	-4.582	73.79	0.00	N
ATOM	378	CA	LYS	A	95	-16.077	5.797	-4.699	73.79	0.00	C
ATOM	379	C	LYS	A	95	-14.855	5.531	-5.567	73.79	0.00	C
ATOM	380	O	LYS	A	95	-14.468	6.367	-6.384	73.79	0.00	O
ATOM	381	N	GLY	A	96	-14.250	4.362	-5.388	50.94	0.00	N
ATOM	382	CA	GLY	A	96	-13.123	3.947	-6.213	50.94	0.00	C
ATOM	383	C	GLY	A	96	-12.812	2.469	-6.020	50.94	0.00	C
ATOM	384	O	GLY	A	96	-13.457	1.789	-5.221	50.94	0.00	O
ATOM	385	N	ARG	A	97	-11.822	1.975	-6.756	36.06	0.00	N
ATOM	386	CA	ARG	A	97	-11.459	0.564	-6.706	36.06	0.00	C
ATOM	387	C	ARG	A	97	-10.170	0.354	-5.923	36.06	0.00	C
ATOM	388	O	ARG	A	97	-9.115	0.865	-6.297	36.06	0.00	O
ATOM	389	N	ALA	A	98	-10.263	-0.402	-4.834	44.10	0.00	N
ATOM	390	CA	ALA	A	98	-9.107	-0.670	-3.987	44.10	0.00	C
ATOM	391	C	ALA	A	98	-8.847	-2.165	-3.864	44.10	0.00	C
ATOM	392	O	ALA	A	98	-9.741	-2.933	-3.507	44.10	0.00	O
ATOM	393	N	ASP	A	99	-7.618	-2.574	-4.163	48.55	0.00	N
ATOM	394	CA	ASP	A	99	-7.213	-3.966	-4.003	48.55	0.00	C
ATOM	395	C	ASP	A	99	-6.610	-4.209	-2.626	48.55	0.00	C
ATOM	396	O	ASP	A	99	-5.477	-3.811	-2.355	48.55	0.00	O
ATOM	397	N	ALA	A	100	-7.373	-4.864	-1.759	55.53	0.00	N
ATOM	398	CA	ALA	A	100	-6.911	-5.173	-0.410	55.53	0.00	C
ATOM	399	C	ALA	A	100	-5.886	-6.300	-0.424	55.53	0.00	C
ATOM	400	O	ALA	A	100	-6.172	-7.407	-0.880	55.53	0.00	O
ATOM	401	N	ILE	A	101	-4.691	-6.012	0.078	45.41	0.00	N
ATOM	402	CA	ILE	A	101	-3.597	-6.975	0.054	45.41	0.00	C
ATOM	403	C	ILE	A	101	-3.146	-7.332	1.465	45.41	0.00	C
ATOM	404	O	ILE	A	101	-2.630	-6.486	2.195	45.41	0.00	O
ATOM	405	N	ILE	A	102	-3.344	-8.590	1.843	45.98	0.00	N
ATOM	406	CA	ILE	A	102	-2.907	-9.076	3.146	45.98	0.00	C
ATOM	407	C	ILE	A	102	-1.684	-9.977	3.016	45.98	0.00	C
ATOM	408	O	ILE	A	102	-1.742	-11.032	2.385	45.98	0.00	O
ATOM	409	N	ARG	A	103	-0.578	-9.554	3.617	0.00	0.00	N
ATOM	410	CA	ARG	A	103	0.664	-10.316	3.562	0.00	0.00	C
ATOM	411	C	ARG	A	103	0.882	-11.107	4.845	0.00	0.00	C
ATOM	412	O	ARG	A	103	1.496	-12.174	4.832	0.00	0.00	O
ATOM	413	N	ASN	A	104	0.376	-10.577	5.954	49.80	0.00	N
ATOM	414	CA	ASN	A	104	0.451	-11.268	7.236	49.80	0.00	C
ATOM	415	C	ASN	A	104	-0.619	-10.765	8.196	49.80	0.00	C
ATOM	416	O	ASN	A	104	-0.427	-9.761	8.883	49.80	0.00	O
ATOM	417	N	ASP	A	105	-1.746	-11.467	8.240	49.74	0.00	N
ATOM	418	CA	ASP	A	105	-2.863	-11.070	9.089	49.74	0.00	C
ATOM	419	C	ASP	A	105	-2.420	-10.887	10.534	49.74	0.00	C
ATOM	420	O	ASP	A	105	-2.654	-9.840	11.138	49.74	0.00	O
ATOM	421	N	ASN	A	106	-1.776	-11.912	11.085	45.80	0.00	N
ATOM	422	CA	ASN	A	106	-1.372	-11.899	12.485	45.80	0.00	C
ATOM	423	C	ASN	A	106	-0.329	-10.821	12.748	45.80	0.00	C
ATOM	424	O	ASN	A	106	-0.302	-10.218	13.821	45.80	0.00	O
ATOM	425	N	GLY	A	107	0.531	-10.584	11.764	40.33	0.00	N
ATOM	426	CA	GLY	A	107	1.592	-9.593	11.894	40.33	0.00	C
ATOM	427	C	GLY	A	107	1.103	-8.208	11.491	40.33	0.00	C
ATOM	428	O	GLY	A	107	1.876	-7.250	11.462	40.33	0.00	O
ATOM	429	N	LYS	A	108	-0.184	-8.108	11.178	28.24	0.00	N
ATOM	430	CA	LYS	A	108	-0.790	-6.828	10.828	28.24	0.00	C
ATOM	431	C	LYS	A	108	-0.114	-6.213	9.610	28.24	0.00	C
ATOM	432	O	LYS	A	108	0.002	-4.992	9.503	28.24	0.00	O
ATOM	433	N	SER	A	109	0.333	-7.066	8.693	50.50	0.00	N
ATOM	434	CA	SER	A	109	0.935	-6.606	7.449	50.50	0.00	C
ATOM	435	C	SER	A	109	-0.095	-6.550	6.326	50.50	0.00	C
ATOM	436	O	SER	A	109	-0.343	-7.547	5.649	50.50	0.00	O
ATOM	437	N	ILE	A	110	-0.690	-5.379	6.134	46.11	0.00	N
ATOM	438	CA	ILE	A	110	-1.730	-5.202	5.127	46.11	0.00	C
ATOM	439	C	ILE	A	110	-1.574	-3.873	4.400	46.11	0.00	C
ATOM	440	O	ILE	A	110	-1.365	-2.834	5.026	46.11	0.00	O
ATOM	441	N	VAL	A	111	-1.676	-3.913	3.076	52.77	0.00	N
ATOM	442	CA	VAL	A	111	-1.586	-2.705	2.264	52.77	0.00	C
ATOM	443	C	VAL	A	111	-2.720	-2.640	1.249	52.77	0.00	C
ATOM	444	O	VAL	A	111	-3.483	-3.592	1.094	52.77	0.00	O
ATOM	445	N	ILE	A	112	-2.824	-1.509	0.559	49.99	0.00	N
ATOM	446	CA	ILE	A	112	-3.885	-1.304	-0.420	49.99	0.00	C
ATOM	447	C	ILE	A	112	-3.319	-0.838	-1.756	49.99	0.00	C
ATOM	448	O	ILE	A	112	-2.634	0.183	-1.828	49.99	0.00	O
ATOM	449	N	GLU	A	113	-3.609	-1.591	-2.811	56.99	0.00	N
ATOM	450	CA	GLU	A	113	-3.240	-1.190	-4.163	56.99	0.00	C
ATOM	451	C	GLU	A	113	-4.434	-0.611	-4.912	56.99	0.00	C
ATOM	452	O	GLU	A	113	-5.368	-1.332	-5.262	56.99	0.00	O
ATOM	453	N	ILE	A	114	-4.398	0.695	-5.153	52.33	0.00	N
ATOM	454	CA	ILE	A	114	-5.512	1.387	-5.789	52.33	0.00	C
ATOM	455	C	ILE	A	114	-5.478	1.212	-7.302	52.33	0.00	C
ATOM	456	O	ILE	A	114	-4.453	1.447	-7.941	52.33	0.00	O

```
ATOM    457  N   LYS A 115      -6.606   0.799  -7.870 54.13  0.00           N
ATOM    458  CA  LYS A 115      -6.678   0.488  -9.292 54.13  0.00           C
ATOM    459  C   LYS A 115      -7.791   1.274  -9.972 54.13  0.00           C
ATOM    460  O   LYS A 115      -8.637   1.873  -9.308 54.13  0.00           O
ATOM    461  N   THR A 116      -7.785   1.269 -11.301 43.55  0.00           N
ATOM    462  CA  THR A 116      -8.842   1.909 -12.074 43.55  0.00           C
ATOM    463  C   THR A 116     -10.181   1.218 -11.856 43.55  0.00           C
ATOM    464  O   THR A 116     -10.266  -0.010 -11.870 43.55  0.00           O
ATOM    465  N   SER A 117     -11.226   2.013 -11.653 40.11  0.00           N
ATOM    466  CA  SER A 117     -12.526   1.484 -11.257 40.11  0.00           C
ATOM    467  C   SER A 117     -13.064   0.509 -12.295 40.11  0.00           C
ATOM    468  O   SER A 117     -13.720  -0.476 -11.955 40.11  0.00           O
ATOM    469  N   ARG A 118     -12.783   0.788 -13.563 45.62  0.00           N
ATOM    470  CA  ARG A 118     -13.249  -0.057 -14.656 45.62  0.00           C
ATOM    471  C   ARG A 118     -12.644  -1.453 -14.569 45.62  0.00           C
ATOM    472  O   ARG A 118     -11.434  -1.605 -14.401 45.62  0.00           O
ATOM    473  N   SER A 119     -13.493  -2.468 -14.686 45.19  0.00           N
ATOM    474  CA  SER A 119     -13.060  -3.851 -14.523 45.19  0.00           C
ATOM    475  C   SER A 119     -12.137  -4.276 -15.658 45.19  0.00           C
ATOM    476  O   SER A 119     -11.366  -5.226 -15.520 45.19  0.00           O
ATOM    477  N   ASP A 120     -12.220  -3.567 -16.779 53.65  0.00           N
ATOM    478  CA  ASP A 120     -11.357  -3.839 -17.922 53.65  0.00           C
ATOM    479  C   ASP A 120     -10.000  -3.166 -17.761 53.65  0.00           C
ATOM    480  O   ASP A 120      -9.066  -3.444 -18.514 53.65  0.00           O
ATOM    481  N   LYS A 121      -9.896  -2.283 -16.775 42.30  0.00           N
ATOM    482  CA  LYS A 121      -8.668  -1.534 -16.543 42.30  0.00           C
ATOM    483  C   LYS A 121      -8.080  -1.850 -15.173 42.30  0.00           C
ATOM    484  O   LYS A 121      -8.634  -2.650 -14.420 42.30  0.00           O
ATOM    485  N   GLY A 122      -6.955  -1.218 -14.858 49.97  0.00           N
ATOM    486  CA  GLY A 122      -6.294  -1.427 -13.574 49.97  0.00           C
ATOM    487  C   GLY A 122      -5.207  -0.385 -13.340 49.97  0.00           C
ATOM    488  O   GLY A 122      -5.158   0.640 -14.021 49.97  0.00           O
ATOM    489  N   LEU A 123      -4.336  -0.653 -12.374 66.42  0.00           N
ATOM    490  CA  LEU A 123      -3.232   0.248 -12.064 66.42  0.00           C
ATOM    491  C   LEU A 123      -2.035  -0.017 -12.966 66.42  0.00           C
ATOM    492  O   LEU A 123      -1.493  -1.122 -12.987 66.42  0.00           O
ATOM    493  N   PRO A 124      -1.625   1.004 -13.712 52.81  0.00           N
ATOM    494  CA  PRO A 124      -0.461   0.897 -14.584 52.81  0.00           C
ATOM    495  C   PRO A 124       0.771   0.456 -13.805 52.81  0.00           C
ATOM    496  O   PRO A 124       0.934   0.800 -12.633 52.81  0.00           O
ATOM    497  N   LEU A 125       1.638  -0.307 -14.461 37.58  0.00           N
ATOM    498  CA  LEU A 125       2.822  -0.856 -13.810 37.58  0.00           C
ATOM    499  C   LEU A 125       3.741   0.253 -13.315 37.58  0.00           C
ATOM    500  O   LEU A 125       4.473   0.077 -12.340 37.58  0.00           O
ATOM    501  N   ILE A 126       3.700   1.396 -13.990 55.60  0.00           N
ATOM    502  CA  ILE A 126       4.514   2.543 -13.606 55.60  0.00           C
ATOM    503  C   ILE A 126       4.041   3.136 -12.284 55.60  0.00           C
ATOM    504  O   ILE A 126       4.781   3.861 -11.619 55.60  0.00           O
ATOM    505  N   HIS A 127       2.805   2.823 -11.910 54.70  0.00           N
ATOM    506  CA  HIS A 127       2.281   3.204 -10.603 54.70  0.00           C
ATOM    507  C   HIS A 127       2.455   2.078  -9.592 54.70  0.00           C
ATOM    508  O   HIS A 127       2.622   2.324  -8.397 54.70  0.00           O
ATOM    509  N   HIS A 128       2.415   0.842 -10.077 42.39  0.00           N
ATOM    510  CA  HIS A 128       2.726  -0.317  -9.249 42.39  0.00           C
ATOM    511  C   HIS A 128       4.125  -0.210  -8.657 42.39  0.00           C
ATOM    512  O   HIS A 128       4.354  -0.591  -7.509 42.39  0.00           O
ATOM    513  N   LYS A 129       5.059   0.308  -9.447 38.83  0.00           N
ATOM    514  CA  LYS A 129       6.413   0.559  -8.971 38.83  0.00           C
ATOM    515  C   LYS A 129       6.408   1.455  -7.739 38.83  0.00           C
ATOM    516  O   LYS A 129       7.098   1.180  -6.756 38.83  0.00           O
ATOM    517  N   MET A 130       5.626   2.527  -7.797 53.12  0.00           N
ATOM    518  CA  MET A 130       5.529   3.466  -6.686 53.12  0.00           C
ATOM    519  C   MET A 130       4.824   2.836  -5.492 53.12  0.00           C
ATOM    520  O   MET A 130       5.153   3.124  -4.341 53.12  0.00           O
ATOM    521  N   GLN A 131       3.851   1.976  -5.772 55.83  0.00           N
ATOM    522  CA  GLN A 131       3.175   1.217  -4.728 55.83  0.00           C
ATOM    523  C   GLN A 131       4.166   0.395  -3.914 55.83  0.00           C
ATOM    524  O   GLN A 131       4.127   0.398  -2.683 55.83  0.00           O
ATOM    525  N   LEU A 132       5.055  -0.308  -4.607 51.50  0.00           N
ATOM    526  CA  LEU A 132       6.068  -1.124  -3.950 51.50  0.00           C
ATOM    527  C   LEU A 132       7.001  -0.269  -3.103 51.50  0.00           C
ATOM    528  O   LEU A 132       7.452  -0.691  -2.039 51.50  0.00           O
ATOM    529  N   GLN A 133       7.287   0.938  -3.581 48.48  0.00           N
ATOM    530  CA  GLN A 133       8.145   1.866  -2.856 48.48  0.00           C
ATOM    531  C   GLN A 133       7.520   2.273  -1.529 48.48  0.00           C
ATOM    532  O   GLN A 133       8.187   2.281  -0.494 48.48  0.00           O
ATOM    533  N   ILE A 134       6.235   2.609  -1.564 74.19  0.00           N
ATOM    534  CA  ILE A 134       5.535   3.090  -0.379 74.19  0.00           C
ATOM    535  C   ILE A 134       5.147   1.938   0.538 74.19  0.00           C
ATOM    536  O   ILE A 134       4.868   2.139   1.719 74.19  0.00           O
ATOM    537  N   TYR A 135       5.132   0.730  -0.014 50.36  0.00           N
ATOM    538  CA  TYR A 135       4.877  -0.470   0.776 50.36  0.00           C
ATOM    539  C   TYR A 135       5.951  -0.670   1.836 50.36  0.00           C
ATOM    540  O   TYR A 135       5.722  -1.332   2.848 50.36  0.00           O
ATOM    541  N   LEU A 136       7.125  -0.096   1.597 55.33  0.00           N
ATOM    542  CA  LEU A 136       8.222  -0.166   2.556 55.33  0.00           C
ATOM    543  C   LEU A 136       7.791   0.346   3.924 55.33  0.00           C
ATOM    544  O   LEU A 136       8.285  -0.114   4.954 55.33  0.00           O
ATOM    545  N   TRP A 137       6.866   1.300   3.929 58.33  0.00           N
ATOM    546  CA  TRP A 137       6.444   1.951   5.163 58.33  0.00           C
ATOM    547  C   TRP A 137       5.014   1.571   5.526 58.33  0.00           C
ATOM    548  O   TRP A 137       4.650   1.533   6.701 58.33  0.00           O
```

ATOM	549	N	LEU	A	138	4.206	1.289	4.508	32.14	0.00	N
ATOM	550	CA	LEU	A	138	2.816	0.903	4.719	32.14	0.00	C
ATOM	551	C	LEU	A	138	2.719	-0.444	5.422	32.14	0.00	C
ATOM	552	O	LEU	A	138	1.850	-0.653	6.268	32.14	0.00	O
ATOM	553	N	PHE	A	139	3.616	-1.357	5.066	45.69	0.00	N
ATOM	554	CA	PHE	A	139	3.709	-2.646	5.742	45.69	0.00	C
ATOM	555	C	PHE	A	139	4.290	-2.493	7.143	45.69	0.00	C
ATOM	556	O	PHE	A	139	3.740	-3.013	8.114	45.69	0.00	O
ATOM	557	N	SER	A	140	5.404	-1.775	7.240	48.76	0.00	N
ATOM	558	CA	SER	A	140	6.189	-1.743	8.468	48.76	0.00	C
ATOM	559	C	SER	A	140	5.547	-0.836	9.509	48.76	0.00	C
ATOM	560	O	SER	A	140	5.817	-0.958	10.704	48.76	0.00	O
ATOM	561	N	ALA	A	141	4.694	0.074	9.049	46.70	0.00	N
ATOM	562	CA	ALA	A	141	3.954	0.954	9.946	46.70	0.00	C
ATOM	563	C	ALA	A	141	2.557	0.415	10.217	46.70	0.00	C
ATOM	564	O	ALA	A	141	1.811	0.972	11.023	46.70	0.00	O
ATOM	565	N	GLU	A	142	2.206	-0.672	9.538	24.72	0.00	N
ATOM	566	CA	GLU	A	142	0.882	-1.271	9.681	24.72	0.00	C
ATOM	567	C	GLU	A	142	-0.214	-0.273	9.329	24.72	0.00	C
ATOM	568	O	GLU	A	142	-1.168	-0.093	10.085	24.72	0.00	O
ATOM	569	N	LYS	A	143	-0.071	0.374	8.178	36.43	0.00	N
ATOM	570	CA	LYS	A	143	-1.005	1.414	7.762	36.43	0.00	C
ATOM	571	C	LYS	A	143	-1.541	1.146	6.362	36.43	0.00	C
ATOM	572	O	LYS	A	143	-1.013	0.303	5.636	36.43	0.00	O
ATOM	573	N	GLY	A	144	-2.590	1.870	5.988	50.78	0.00	N
ATOM	574	CA	GLY	A	144	-3.191	1.721	4.667	50.78	0.00	C
ATOM	575	C	GLY	A	144	-3.476	3.079	4.036	50.78	0.00	C
ATOM	576	O	GLY	A	144	-3.227	4.120	4.643	50.78	0.00	O
ATOM	577	N	ILE	A	145	-3.997	3.059	2.814	48.61	0.00	N
ATOM	578	CA	ILE	A	145	-4.347	4.288	2.111	48.61	0.00	C
ATOM	579	C	ILE	A	145	-5.389	4.026	1.031	48.61	0.00	C
ATOM	580	O	ILE	A	145	-5.278	3.067	0.268	48.61	0.00	O
ATOM	581	N	LEU	A	146	-6.400	4.886	0.971	52.71	0.00	N
ATOM	582	CA	LEU	A	146	-7.386	4.837	-0.102	52.71	0.00	C
ATOM	583	C	LEU	A	146	-7.304	6.078	-0.981	52.71	0.00	C
ATOM	584	O	LEU	A	146	-7.851	7.127	-0.642	52.71	0.00	O
ATOM	585	N	VAL	A	147	-6.619	5.951	-2.113	58.59	0.00	N
ATOM	586	CA	VAL	A	147	-6.448	7.068	-3.034	58.59	0.00	C
ATOM	587	C	VAL	A	147	-7.651	7.212	-3.955	58.59	0.00	C
ATOM	588	O	VAL	A	147	-8.104	6.239	-4.558	58.59	0.00	O
ATOM	589	N	TYR	A	148	-8.167	8.433	-4.060	50.20	0.00	N
ATOM	590	CA	TYR	A	148	-9.299	8.713	-4.936	50.20	0.00	C
ATOM	591	C	TYR	A	148	-8.964	9.812	-5.936	50.20	0.00	C
ATOM	592	O	TYR	A	148	-8.220	10.742	-5.623	50.20	0.00	O
ATOM	593	N	ILE	A	149	-9.515	9.699	-7.138	59.40	0.00	N
ATOM	594	CA	ILE	A	149	-9.255	10.669	-8.195	59.40	0.00	C
ATOM	595	C	ILE	A	149	-10.551	11.264	-8.730	59.40	0.00	C
ATOM	596	O	ILE	A	149	-11.401	10.548	-9.260	59.40	0.00	O
ATOM	597	N	THR	A	150	-10.697	12.576	-8.589	50.91	0.00	N
ATOM	598	CA	THR	A	150	-11.848	13.283	-9.139	50.91	0.00	C
ATOM	599	C	THR	A	150	-11.435	14.196	-10.286	50.91	0.00	C
ATOM	600	O	THR	A	150	-10.267	14.560	-10.414	50.91	0.00	O
ATOM	601	N	PRO	A	151	-12.403	14.565	-11.119	38.79	0.00	N
ATOM	602	CA	PRO	A	151	-12.150	15.467	-12.236	38.79	0.00	C
ATOM	603	C	PRO	A	151	-11.749	16.853	-11.745	38.79	0.00	C
ATOM	604	O	PRO	A	151	-11.261	17.677	-12.518	38.79	0.00	O
ATOM	605	N	ASP	A	152	-11.957	17.102	-10.458	46.49	0.00	N
ATOM	606	CA	ASP	A	152	-11.584	18.375	-9.853	46.49	0.00	C
ATOM	607	C	ASP	A	152	-10.167	18.325	-9.295	46.49	0.00	C
ATOM	608	O	ASP	A	152	-9.387	19.261	-9.472	46.49	0.00	O
ATOM	609	N	ARG	A	153	-9.841	17.229	-8.620	48.05	0.00	N
ATOM	610	CA	ARG	A	153	-8.526	17.068	-8.010	48.05	0.00	C
ATOM	611	C	ARG	A	153	-8.323	15.644	-7.509	48.05	0.00	C
ATOM	612	O	ARG	A	153	-9.284	14.897	-7.324	48.05	0.00	O
ATOM	613	N	ILE	A	154	-7.065	15.272	-7.291	44.50	0.00	N
ATOM	614	CA	ILE	A	154	-6.740	13.998	-6.664	44.50	0.00	C
ATOM	615	C	ILE	A	154	-6.587	14.148	-5.156	44.50	0.00	C
ATOM	616	O	ILE	A	154	-5.919	15.064	-4.679	44.50	0.00	O
ATOM	617	N	ALA	A	155	-7.212	13.244	-4.409	44.74	0.00	N
ATOM	618	CA	ALA	A	155	-7.213	13.320	-2.954	44.74	0.00	C
ATOM	619	C	ALA	A	155	-7.017	11.944	-2.330	44.74	0.00	C
ATOM	620	O	ALA	A	155	-7.554	10.949	-2.816	44.74	0.00	O
ATOM	621	N	GLU	A	156	-6.245	11.894	-1.249	44.85	0.00	N
ATOM	622	CA	GLU	A	156	-5.882	10.628	-0.625	44.85	0.00	C
ATOM	623	C	GLU	A	156	-6.514	10.493	0.755	44.85	0.00	C
ATOM	624	O	GLU	A	156	-6.402	11.391	1.590	44.85	0.00	O
ATOM	625	N	TYR	A	157	-7.179	9.366	0.988	45.56	0.00	N
ATOM	626	CA	TYR	A	157	-7.688	9.038	2.314	45.56	0.00	C
ATOM	627	C	TYR	A	157	-6.734	8.111	3.056	45.56	0.00	C
ATOM	628	O	TYR	A	157	-6.838	6.888	2.958	45.56	0.00	O
ATOM	629	N	GLU	A	158	-5.804	8.701	3.800	42.77	0.00	N
ATOM	630	CA	GLU	A	158	-4.818	7.931	4.548	42.77	0.00	C
ATOM	631	C	GLU	A	158	-5.462	7.202	5.720	42.77	0.00	C
ATOM	632	O	GLU	A	158	-6.300	7.762	6.427	42.77	0.00	O
ATOM	633	N	ILE	A	159	-5.067	5.950	5.921	48.46	0.00	N
ATOM	634	CA	ILE	A	159	-5.604	5.142	7.010	48.46	0.00	C
ATOM	635	C	ILE	A	159	-4.551	4.892	8.082	48.46	0.00	C
ATOM	636	O	ILE	A	159	-3.571	4.185	7.850	48.46	0.00	O
ATOM	637	N	ASN	A	160	-4.761	5.477	9.257	42.29	0.00	N
ATOM	638	CA	ASN	A	160	-3.779	5.407	10.332	42.29	0.00	C
ATOM	639	C	ASN	A	160	-3.594	3.976	10.818	42.29	0.00	C
ATOM	640	O	ASN	A	160	-4.358	3.082	10.452	42.29	0.00	O

```
ATOM    641  N   GLU A 161      -2.575   3.763  11.644 29.62  0.00           N
ATOM    642  CA  GLU A 161      -2.253   2.429  12.137 29.62  0.00           C
ATOM    643  C   GLU A 161      -3.358   1.894  13.041 29.62  0.00           C
ATOM    644  O   GLU A 161      -3.853   0.785  12.840 29.62  0.00           O
ATOM    645  N   PRO A 162      -3.739   2.688  14.035 47.60  0.00           N
ATOM    646  CA  PRO A 162      -4.800   2.304  14.958 47.60  0.00           C
ATOM    647  C   PRO A 162      -6.158   2.303  14.268 47.60  0.00           C
ATOM    648  O   PRO A 162      -7.060   1.560  14.652 47.60  0.00           O
ATOM    649  N   LEU A 163      -6.297   3.141  13.245 43.37  0.00           N
ATOM    650  CA  LEU A 163      -7.508   3.166  12.435 43.37  0.00           C
ATOM    651  C   LEU A 163      -7.657   1.883  11.626 43.37  0.00           C
ATOM    652  O   LEU A 163      -8.745   1.317  11.537 43.37  0.00           O
ATOM    653  N   ASP A 164      -6.555   1.430  11.038 15.75  0.00           N
ATOM    654  CA  ASP A 164      -6.541   0.170  10.305 15.75  0.00           C
ATOM    655  C   ASP A 164      -6.905  -0.999  11.212 15.75  0.00           C
ATOM    656  O   ASP A 164      -7.704  -1.859  10.841 15.75  0.00           O
ATOM    657  N   GLU A 165      -6.314  -1.024  12.402 44.28  0.00           N
ATOM    658  CA  GLU A 165      -6.625  -2.051  13.389 44.28  0.00           C
ATOM    659  C   GLU A 165      -8.101  -2.027  13.764 44.28  0.00           C
ATOM    660  O   GLU A 165      -8.738  -3.074  13.885 44.28  0.00           O
ATOM    661  N   ALA A 166      -8.641  -0.826  13.946 56.50  0.00           N
ATOM    662  CA  ALA A 166     -10.042  -0.664  14.320 56.50  0.00           C
ATOM    663  C   ALA A 166     -10.966  -1.240  13.256 56.50  0.00           C
ATOM    664  O   ALA A 166     -11.924  -1.947  13.569 56.50  0.00           O
ATOM    665  N   THR A 167     -10.675  -0.933  11.997 42.80  0.00           N
ATOM    666  CA  THR A 167     -11.529  -1.344  10.890 42.80  0.00           C
ATOM    667  C   THR A 167     -11.428  -2.843  10.642 42.80  0.00           C
ATOM    668  O   THR A 167     -12.402  -3.486  10.251 42.80  0.00           O
ATOM    669  N   ILE A 168     -10.242  -3.397  10.872 43.48  0.00           N
ATOM    670  CA  ILE A 168     -10.030  -4.834  10.752 43.48  0.00           C
ATOM    671  C   ILE A 168     -10.884  -5.602  11.753 43.48  0.00           C
ATOM    672  O   ILE A 168     -11.614  -6.523  11.384 43.48  0.00           O
ATOM    673  N   VAL A 169     -10.789  -5.219  13.021 59.04  0.00           N
ATOM    674  CA  VAL A 169     -11.501  -5.913  14.087 59.04  0.00           C
ATOM    675  C   VAL A 169     -13.005  -5.697  13.977 59.04  0.00           C
ATOM    676  O   VAL A 169     -13.788  -6.638  14.105 59.04  0.00           O
ATOM    677  N   ARG A 170     -13.403  -4.452  13.740 42.54  0.00           N
ATOM    678  CA  ARG A 170     -14.815  -4.093  13.701 42.54  0.00           C
ATOM    679  C   ARG A 170     -15.531  -4.793  12.552 42.54  0.00           C
ATOM    680  O   ARG A 170     -16.626  -5.327  12.724 42.54  0.00           O
ATOM    681  N   LEU A 171     -14.904  -4.787  11.381 50.99  0.00           N
ATOM    682  CA  LEU A 171     -15.507  -5.367  10.186 50.99  0.00           C
ATOM    683  C   LEU A 171     -15.463  -6.888  10.230 50.99  0.00           C
ATOM    684  O   LEU A 171     -16.337  -7.560   9.681 50.99  0.00           O
ATOM    685  N   ALA A 172     -14.441  -7.428  10.886 48.07  0.00           N
ATOM    686  CA  ALA A 172     -14.363  -8.861  11.136 48.07  0.00           C
ATOM    687  C   ALA A 172     -15.526  -9.335  11.999 48.07  0.00           C
ATOM    688  O   ALA A 172     -16.155 -10.351  11.705 48.07  0.00           O
ATOM    689  N   GLU A 173     -15.806  -8.593  13.064 69.67  0.00           N
ATOM    690  CA  GLU A 173     -16.909  -8.923  13.959 69.67  0.00           C
ATOM    691  C   GLU A 173     -18.254  -8.722  13.274 69.67  0.00           C
ATOM    692  O   GLU A 173     -19.193  -9.488  13.492 69.67  0.00           O
ATOM    693  N   ASP A 174     -18.341  -7.688  12.444 40.91  0.00           N
ATOM    694  CA  ASP A 174     -19.549  -7.427  11.670 40.91  0.00           C
ATOM    695  C   ASP A 174     -19.832  -8.558  10.690 40.91  0.00           C
ATOM    696  O   ASP A 174     -20.981  -8.955  10.500 40.91  0.00           O
ATOM    697  N   THR A 175     -18.776  -9.076  10.071 47.42  0.00           N
ATOM    698  CA  THR A 175     -18.902 -10.206   9.159 47.42  0.00           C
ATOM    699  C   THR A 175     -19.352 -11.461   9.896 47.42  0.00           C
ATOM    700  O   THR A 175     -20.185 -12.219   9.399 47.42  0.00           O
ATOM    701  N   ILE A 176     -18.797 -11.674  11.085 32.81  0.00           N
ATOM    702  CA  ILE A 176     -19.205 -12.788  11.932 32.81  0.00           C
ATOM    703  C   ILE A 176     -20.692 -12.716  12.258 32.81  0.00           C
ATOM    704  O   ILE A 176     -21.408 -13.713  12.155 32.81  0.00           O
ATOM    705  N   MET A 177     -21.150 -11.533  12.649 38.99  0.00           N
ATOM    706  CA  MET A 177     -22.556 -11.325  12.975 38.99  0.00           C
ATOM    707  C   MET A 177     -23.436 -11.475  11.741 38.99  0.00           C
ATOM    708  O   MET A 177     -24.568 -11.952  11.827 38.99  0.00           O
ATOM    709  N   LEU A 178     -22.909 -11.065  10.591 39.02  0.00           N
ATOM    710  CA  LEU A 178     -23.625 -11.202   9.328 39.02  0.00           C
ATOM    711  C   LEU A 178     -23.839 -12.668   8.973 39.02  0.00           C
ATOM    712  O   LEU A 178     -24.902 -13.048   8.481 39.02  0.00           O
ATOM    713  N   GLN A 179     -22.823 -13.487   9.223 51.25  0.00           N
ATOM    714  CA  GLN A 179     -22.923 -14.923   8.994 51.25  0.00           C
ATOM    715  C   GLN A 179     -23.858 -15.579  10.001 51.25  0.00           C
ATOM    716  O   GLN A 179     -24.593 -16.508   9.668 51.25  0.00           O
ATOM    717  N   ASN A 180     -23.825 -15.089  11.236 45.90  0.00           N
ATOM    718  CA  ASN A 180     -24.684 -15.614  12.292 45.90  0.00           C
ATOM    719  C   ASN A 180     -26.139 -15.229  12.060 45.90  0.00           C
ATOM    720  O   ASN A 180     -27.052 -15.984  12.393 45.90  0.00           O
ATOM    721  N   SER A 181     -26.349 -14.049  11.486 62.18  0.00           N
ATOM    722  CA  SER A 181     -27.695 -13.547  11.236 62.18  0.00           C
ATOM    723  C   SER A 181     -27.768 -12.803   9.909 62.18  0.00           C
ATOM    724  O   SER A 181     -27.204 -11.718   9.763 62.18  0.00           O
ATOM    725  N   PRO A 182     -28.465 -13.392   8.943 60.53  0.00           N
ATOM    726  CA  PRO A 182     -28.622 -12.781   7.629 60.53  0.00           C
ATOM    727  C   PRO A 182     -29.268 -11.405   7.736 60.53  0.00           C
ATOM    728  O   PRO A 182     -29.086 -10.553   6.866 60.53  0.00           O
ATOM    729  N   ARG A 183     -30.025 -11.194   8.808 84.37  0.00           N
ATOM    730  CA  ARG A 183     -30.759  -9.948   8.995 84.37  0.00           C
ATOM    731  C   ARG A 183     -29.881  -8.883   9.638 84.37  0.00           C
ATOM    732  O   ARG A 183     -30.347  -7.786   9.948 84.37  0.00           O
```

```
ATOM    733  N   PHE A 184     -28.610  -9.212   9.838 52.25  0.00           N
ATOM    734  CA  PHE A 184     -27.642  -8.252  10.354 52.25  0.00           C
ATOM    735  C   PHE A 184     -27.592  -6.999   9.488 52.25  0.00           C
ATOM    736  O   PHE A 184     -27.570  -7.083   8.260 52.25  0.00           O
ATOM    737  N   ASN A 185     -27.576  -5.840  10.134 63.62  0.00           N
ATOM    738  CA  ASN A 185     -27.545  -4.566   9.424 63.62  0.00           C
ATOM    739  C   ASN A 185     -26.163  -4.289   8.849 63.62  0.00           C
ATOM    740  O   ASN A 185     -25.415  -3.464   9.375 63.62  0.00           O
ATOM    741  N   TRP A 186     -25.827  -4.984   7.767 44.36  0.00           N
ATOM    742  CA  TRP A 186     -24.536  -4.809   7.114 44.36  0.00           C
ATOM    743  C   TRP A 186     -24.491  -3.508   6.322 44.36  0.00           C
ATOM    744  O   TRP A 186     -24.978  -3.441   5.193 44.36  0.00           O
ATOM    745  N   GLU A 187     -23.903  -2.477   6.919 82.86  0.00           N
ATOM    746  CA  GLU A 187     -23.773  -1.183   6.262 82.86  0.00           C
ATOM    747  C   GLU A 187     -22.320  -0.886   5.913 82.86  0.00           C
ATOM    748  O   GLU A 187     -21.895   0.270   5.912 82.86  0.00           O
ATOM    749  N   CYS A 188     -21.560  -1.936   5.619 55.08  0.00           N
ATOM    750  CA  CYS A 188     -20.135  -1.798   5.348 55.08  0.00           C
ATOM    751  C   CYS A 188     -19.776  -2.376   3.985 55.08  0.00           C
ATOM    752  O   CYS A 188     -18.698  -2.944   3.805 55.08  0.00           O
ATOM    753  N   LYS A 189     -20.684  -2.226   3.026 38.40  0.00           N
ATOM    754  CA  LYS A 189     -20.477  -2.760   1.684 38.40  0.00           C
ATOM    755  C   LYS A 189     -19.211  -2.193   1.056 38.40  0.00           C
ATOM    756  O   LYS A 189     -18.490  -2.896   0.349 38.40  0.00           O
ATOM    757  N   TYR A 190     -18.947  -0.918   1.316 25.73  0.00           N
ATOM    758  CA  TYR A 190     -17.894  -0.194   0.614 25.73  0.00           C
ATOM    759  C   TYR A 190     -16.692   0.046   1.519 25.73  0.00           C
ATOM    760  O   TYR A 190     -16.037   1.085   1.437 25.73  0.00           O
ATOM    761  N   CYS A 191     -16.407  -0.922   2.384 41.49  0.00           N
ATOM    762  CA  CYS A 191     -15.184  -0.906   3.177 41.49  0.00           C
ATOM    763  C   CYS A 191     -14.118  -1.807   2.566 41.49  0.00           C
ATOM    764  O   CYS A 191     -14.431  -2.746   1.834 41.49  0.00           O
ATOM    765  N   ILE A 192     -12.859  -1.515   2.868 38.99  0.00           N
ATOM    766  CA  ILE A 192     -11.739  -2.209   2.244 38.99  0.00           C
ATOM    767  C   ILE A 192     -11.698  -3.674   2.659 38.99  0.00           C
ATOM    768  O   ILE A 192     -11.155  -4.517   1.944 38.99  0.00           O
ATOM    769  N   PHE A 193     -12.277  -3.973   3.817 38.05  0.00           N
ATOM    770  CA  PHE A 193     -12.291  -5.334   4.339 38.05  0.00           C
ATOM    771  C   PHE A 193     -13.676  -5.955   4.216 38.05  0.00           C
ATOM    772  O   PHE A 193     -13.962  -6.984   4.830 38.05  0.00           O
ATOM    773  N   SER A 194     -14.533  -5.327   3.418 44.71  0.00           N
ATOM    774  CA  SER A 194     -15.924  -5.748   3.305 44.71  0.00           C
ATOM    775  C   SER A 194     -16.038  -7.087   2.587 44.71  0.00           C
ATOM    776  O   SER A 194     -17.053  -7.775   2.692 44.71  0.00           O
ATOM    777  N   VAL A 195     -14.989  -7.452   1.857 51.78  0.00           N
ATOM    778  CA  VAL A 195     -14.989  -8.685   1.080 51.78  0.00           C
ATOM    779  C   VAL A 195     -14.024  -9.706   1.670 51.78  0.00           C
ATOM    780  O   VAL A 195     -13.720 -10.722   1.044 51.78  0.00           O
ATOM    781  N   ILE A 196     -13.543  -9.431   2.878 33.86  0.00           N
ATOM    782  CA  ILE A 196     -12.543 -10.277   3.515 33.86  0.00           C
ATOM    783  C   ILE A 196     -13.060 -10.846   4.831 33.86  0.00           C
ATOM    784  O   ILE A 196     -13.620 -10.121   5.653 33.86  0.00           O
ATOM    785  N   CYS A 197     -12.867 -12.146   5.024 51.61  0.00           N
ATOM    786  CA  CYS A 197     -13.447 -12.844   6.165 51.61  0.00           C
ATOM    787  C   CYS A 197     -12.610 -12.638   7.421 51.61  0.00           C
ATOM    788  O   CYS A 197     -11.436 -12.276   7.344 51.61  0.00           O
ATOM    789  N   PRO A 198     -13.221 -12.871   8.578 31.41  0.00           N
ATOM    790  CA  PRO A 198     -12.525 -12.739   9.853 31.41  0.00           C
ATOM    791  C   PRO A 198     -11.358 -13.715   9.946 31.41  0.00           C
ATOM    792  O   PRO A 198     -10.437 -13.521  10.739 31.41  0.00           O
ATOM    793  N   ALA A 199     -11.405 -14.765   9.132 44.28  0.00           N
ATOM    794  CA  ALA A 199     -10.309 -15.722   9.055 44.28  0.00           C
ATOM    795  C   ALA A 199      -9.023 -15.052   8.588 44.28  0.00           C
ATOM    796  O   ALA A 199      -7.925 -15.474   8.950 44.28  0.00           O
ATOM    797  N   LYS A 200      -9.166 -14.006   7.783 51.19  0.00           N
ATOM    798  CA  LYS A 200      -8.017 -13.260   7.283 51.19  0.00           C
ATOM    799  C   LYS A 200      -7.829 -11.958   8.052 51.19  0.00           C
ATOM    800  O   LYS A 200      -6.725 -11.419   8.117 51.19  0.00           O
ATOM    801  N   LEU A 201      -8.914 -11.458   8.634 36.97  0.00           N
ATOM    802  CA  LEU A 201      -8.859 -10.251   9.448 36.97  0.00           C
ATOM    803  C   LEU A 201      -8.462 -10.573  10.884 36.97  0.00           C
ATOM    804  O   LEU A 201      -9.279 -10.480  11.800 36.97  0.00           O
ATOM    805  N   THR A 202      -7.203 -10.952  11.074 47.06  0.00           N
ATOM    806  CA  THR A 202      -6.689 -11.267  12.401 47.06  0.00           C
ATOM    807  C   THR A 202      -6.152 -10.021  13.094 47.06  0.00           C
ATOM    808  O   THR A 202      -6.910  -9.255  13.622 47.06  0.00           O
TER     809      THR A 202
ENDMDL
END
REMARK   1 ***********************************************************************
REMARK   1 Start File NAT_vs_DEC1_dSi_colored.pdb
REMARK   1 The occupancy record was modified to represent dSi := Si(DEC)-Si(NAT).
REMARK   1 Occ=0.00 means dSi=-1.507170 kB; Occ=99.99 means dSi=1.490229 kB.
MODEL        0
ATOM      1  N   MSE A   1      23.268  -4.104 -18.521 35.21  0.00           N
ATOM      2  CA  MSE A   1      23.439  -2.722 -18.016 35.21  0.00           C
ATOM      3  C   MSE A   1      22.152  -2.161 -17.383 35.21  0.00           C
ATOM      4  O   MSE A   1      22.147  -1.650 -16.252 35.21  0.00           O
ATOM      5  N   ILE A   2      21.045  -2.254 -18.100 31.82  0.00           N
ATOM      6  CA  ILE A   2      19.816  -1.632 -17.608 31.82  0.00           C
ATOM      7  C   ILE A   2      19.269  -2.405 -16.412 31.82  0.00           C
ATOM      8  O   ILE A   2      18.935  -1.835 -15.391 31.82  0.00           O
```

```
ATOM      9  N   THR A   3      19.186  -3.703 -16.563 27.55  0.00           N
ATOM     10  CA  THR A   3      18.718  -4.606 -15.498 27.55  0.00           C
ATOM     11  C   THR A   3      19.478  -4.398 -14.212 27.55  0.00           C
ATOM     12  O   THR A   3      18.884  -4.188 -13.150 27.55  0.00           O
ATOM     13  N   GLU A   4      20.800  -4.447 -14.318 41.92  0.00           N
ATOM     14  CA  GLU A   4      21.687  -4.148 -13.195 41.92  0.00           C
ATOM     15  C   GLU A   4      21.342  -2.832 -12.484 41.92  0.00           C
ATOM     16  O   GLU A   4      21.232  -2.791 -11.242 41.92  0.00           O
ATOM     17  N   PHE A   5      21.133  -1.765 -13.247 41.07  0.00           N
ATOM     18  CA  PHE A   5      20.826  -0.486 -12.602 41.07  0.00           C
ATOM     19  C   PHE A   5      19.486  -0.520 -11.886 41.07  0.00           C
ATOM     20  O   PHE A   5      19.415  -0.122 -10.736 41.07  0.00           O
ATOM     21  N   LEU A   6      18.429  -1.039 -12.529 17.98  0.00           N
ATOM     22  CA  LEU A   6      17.100  -1.072 -11.873 17.98  0.00           C
ATOM     23  C   LEU A   6      17.119  -1.921 -10.586 17.98  0.00           C
ATOM     24  O   LEU A   6      16.551  -1.545  -9.531 17.98  0.00           O
ATOM     25  N   LEU A   7      17.767  -3.071 -10.672 29.60  0.00           N
ATOM     26  CA  LEU A   7      17.843  -3.929  -9.486 29.60  0.00           C
ATOM     27  C   LEU A   7      18.603  -3.241  -8.341 29.60  0.00           C
ATOM     28  O   LEU A   7      18.190  -3.292  -7.184 29.60  0.00           O
ATOM     29  N   LYS A   8      19.691  -2.566  -8.693 47.82  0.00           N
ATOM     30  CA  LYS A   8      20.463  -1.856  -7.680 47.82  0.00           C
ATOM     31  C   LYS A   8      19.628  -0.762  -7.044 47.82  0.00           C
ATOM     32  O   LYS A   8      19.527  -0.688  -5.819 47.82  0.00           O
ATOM     33  N   LYS A   9      18.947   0.036  -7.860 30.34  0.00           N
ATOM     34  CA  LYS A   9      18.144   1.113  -7.292 30.34  0.00           C
ATOM     35  C   LYS A   9      17.009   0.545  -6.471 30.34  0.00           C
ATOM     36  O   LYS A   9      16.708   1.086  -5.402 30.34  0.00           O
ATOM     37  N   LYS A  10      16.379  -0.547  -6.912 26.27  0.00           N
ATOM     38  CA  LYS A  10      15.262  -1.072  -6.116 26.27  0.00           C
ATOM     39  C   LYS A  10      15.660  -1.681  -4.726 26.27  0.00           C
ATOM     40  O   LYS A  10      14.931  -1.547  -3.695 26.27  0.00           O
ATOM     41  N   LEU A  11      16.798  -2.366  -4.699 25.05  0.00           N
ATOM     42  CA  LEU A  11      17.328  -2.879  -3.411 25.05  0.00           C
ATOM     43  C   LEU A  11      17.613  -1.686  -2.439 25.05  0.00           C
ATOM     44  O   LEU A  11      17.179  -1.698  -1.270 25.05  0.00           O
ATOM     45  N   GLU A  12      18.299  -0.655  -2.945 18.67  0.00           N
ATOM     46  CA  GLU A  12      18.475   0.605  -2.181 18.67  0.00           C
ATOM     47  C   GLU A  12      17.179   1.213  -1.616 18.67  0.00           C
ATOM     48  O   GLU A  12      17.048   1.390  -0.426 18.67  0.00           O
ATOM     49  N   GLU A  13      16.225   1.536  -2.461  0.00  0.00           N
ATOM     50  CA  GLU A  13      14.901   1.950  -1.974  0.00  0.00           C
ATOM     51  C   GLU A  13      14.358   1.037  -0.868  0.00  0.00           C
ATOM     52  O   GLU A  13      13.966   1.505   0.190  0.00  0.00           O
ATOM     53  N   HIS A  14      14.335  -0.267  -1.101 18.87  0.00           N
ATOM     54  CA  HIS A  14      13.794  -1.199  -0.106 18.87  0.00           C
ATOM     55  C   HIS A  14      14.560  -1.126   1.236 18.87  0.00           C
ATOM     56  O   HIS A  14      13.970  -1.122   2.322 18.87  0.00           O
ATOM     57  N   LEU A  15      15.884  -1.079   1.145 29.94  0.00           N
ATOM     58  CA  LEU A  15      16.750  -1.011   2.322 29.94  0.00           C
ATOM     59  C   LEU A  15      16.724   0.377   2.988 29.94  0.00           C
ATOM     60  O   LEU A  15      17.114   0.492   4.140 29.94  0.00           O
ATOM     61  N   SER A  16      16.283   1.421   2.277 29.27  0.00           N
ATOM     62  CA  SER A  16      16.215   2.761   2.870 29.27  0.00           C
ATOM     63  C   SER A  16      15.126   2.866   3.937 29.27  0.00           C
ATOM     64  O   SER A  16      15.184   3.766   4.746 29.27  0.00           O
ATOM     65  N   HIS A  17      14.126   1.987   3.905 26.74  0.00           N
ATOM     66  CA  HIS A  17      12.931   2.175   4.710 26.74  0.00           C
ATOM     67  C   HIS A  17      12.911   1.290   5.925 26.74  0.00           C
ATOM     68  O   HIS A  17      12.211   0.285   5.939 26.74  0.00           O
ATOM     69  N   VAL A  18      13.667   1.662   6.952 28.01  0.00           N
ATOM     70  CA  VAL A  18      13.649   0.930   8.226 28.01  0.00           C
ATOM     71  C   VAL A  18      12.455   1.416   9.073 28.01  0.00           C
ATOM     72  O   VAL A  18      12.231   2.619   9.198 28.01  0.00           O
ATOM     73  N   LYS A  19      11.711   0.480   9.654 25.89  0.00           N
ATOM     74  CA  LYS A  19      10.394   0.738  10.251 25.89  0.00           C
ATOM     75  C   LYS A  19      10.466   0.797  11.779 25.89  0.00           C
ATOM     76  O   LYS A  19      11.024  -0.102  12.394 25.89  0.00           O
ATOM     77  N   GLU A  20       9.892   1.832  12.391 38.34  0.00           N
ATOM     78  CA  GLU A  20      10.003   2.007  13.848 38.34  0.00           C
ATOM     79  C   GLU A  20       8.986   1.178  14.597 38.34  0.00           C
ATOM     80  O   GLU A  20       7.952   0.808  14.049 38.34  0.00           O
ATOM     81  N   GLU A  21       9.300   0.899  15.861 52.27  0.00           N
ATOM     82  CA  GLU A  21       8.634  -0.168  16.607 52.27  0.00           C
ATOM     83  C   GLU A  21       7.153   0.124  16.844 52.27  0.00           C
ATOM     84  O   GLU A  21       6.369  -0.811  17.040 52.27  0.00           O
ATOM     85  N   ASN A  22       6.774   1.399  16.808 45.69  0.00           N
ATOM     86  CA  ASN A  22       5.371   1.790  16.970 45.69  0.00           C
ATOM     87  C   ASN A  22       4.716   2.391  15.715 45.69  0.00           C
ATOM     88  O   ASN A  22       3.648   3.000  15.824 45.69  0.00           O
ATOM     89  N   THR A  23       5.339   2.269  14.540 71.65  0.00           N
ATOM     90  CA  THR A  23       4.636   2.674  13.297 71.65  0.00           C
ATOM     91  C   THR A  23       3.937   1.492  12.597 71.65  0.00           C
ATOM     92  O   THR A  23       4.343   0.315  12.707 71.65  0.00           O
ATOM     93  N   ILE A  24       2.863   1.841  11.907 76.27  0.00           N
ATOM     94  CA  ILE A  24       2.133   0.908  11.089 76.27  0.00           C
ATOM     95  C   ILE A  24       1.893   1.588   9.781 76.27  0.00           C
ATOM     96  O   ILE A  24       1.270   2.646   9.760 76.27  0.00           O
ATOM     97  N   TYR A  25       2.393   1.015   8.691 61.46  0.00           N
ATOM     98  CA  TYR A  25       2.072   1.558   7.393 61.46  0.00           C
ATOM     99  C   TYR A  25       0.609   1.246   7.051 61.46  0.00           C
ATOM    100  O   TYR A  25       0.029   0.273   7.510 61.46  0.00           O
```

```
ATOM    101  N   VAL A  26       0.019   2.088   6.230 76.75  0.00           N
ATOM    102  CA  VAL A  26      -1.326   1.856   5.716 76.75  0.00           C
ATOM    103  C   VAL A  26      -1.394   0.504   4.989 76.75  0.00           C
ATOM    104  O   VAL A  26      -2.320  -0.244   5.138 76.75  0.00           O
ATOM    105  N   THR A  27      -0.413   0.264   4.153 59.89  0.00           N
ATOM    106  CA  THR A  27      -0.225  -0.940   3.432 59.89  0.00           C
ATOM    107  C   THR A  27      -0.214  -2.155   4.325 59.89  0.00           C
ATOM    108  O   THR A  27      -0.816  -3.189   4.005 59.89  0.00           O
ATOM    109  N   ASP A  28       0.421  -2.066   5.476 62.29  0.00           N
ATOM    110  CA  ASP A  28       0.217  -3.125   6.447 62.29  0.00           C
ATOM    111  C   ASP A  28      -1.221  -3.366   6.899 62.29  0.00           C
ATOM    112  O   ASP A  28      -1.560  -4.494   7.172 62.29  0.00           O
ATOM    113  N   LEU A  29      -2.082  -2.355   6.958 78.11  0.00           N
ATOM    114  CA  LEU A  29      -3.418  -2.565   7.521 78.11  0.00           C
ATOM    115  C   LEU A  29      -4.304  -3.447   6.637 78.11  0.00           C
ATOM    116  O   LEU A  29      -5.335  -3.962   7.065 78.11  0.00           O
ATOM    117  N   VAL A  30      -3.930  -3.597   5.378 60.79  0.00           N
ATOM    118  CA  VAL A  30      -4.776  -4.325   4.449 60.79  0.00           C
ATOM    119  C   VAL A  30      -4.099  -5.619   4.014 60.79  0.00           C
ATOM    120  O   VAL A  30      -4.413  -6.174   2.971 60.79  0.00           O
ATOM    121  N   ARG A  31      -3.154  -6.069   4.828 99.99  0.00           N
ATOM    122  CA  ARG A  31      -2.426  -7.287   4.613 99.99  0.00           C
ATOM    123  C   ARG A  31      -2.433  -8.174   5.834 99.99  0.00           C
ATOM    124  O   ARG A  31      -2.927  -7.806   6.907 99.99  0.00           O
ATOM    125  N   CYS A  32      -1.955  -9.395   5.616 67.03  0.00           N
ATOM    126  CA  CYS A  32      -1.910 -10.426   6.637 67.03  0.00           C
ATOM    127  C   CYS A  32      -1.015  -9.951   7.770 67.03  0.00           C
ATOM    128  O   CYS A  32       0.134  -9.567   7.534 67.03  0.00           O
ATOM    129  N   PRO A  33      -1.508 -10.024   8.999 50.80  0.00           N
ATOM    130  CA  PRO A  33      -0.664  -9.610  10.083 50.80  0.00           C
ATOM    131  C   PRO A  33       0.611 -10.429  10.223 50.80  0.00           C
ATOM    132  O   PRO A  33       1.694  -9.852  10.411 50.80  0.00           O
ATOM    133  N   ARG A  34       0.512 -11.757  10.119 19.13  0.00           N
ATOM    134  CA  ARG A  34       1.689 -12.574  10.256 19.13  0.00           C
ATOM    135  C   ARG A  34       2.720 -12.198   9.218 19.13  0.00           C
ATOM    136  O   ARG A  34       3.911 -12.267   9.453 19.13  0.00           O
ATOM    137  N   ARG A  35       2.276 -11.837   8.033 22.35  0.00           N
ATOM    138  CA  ARG A  35       3.238 -11.530   6.965 22.35  0.00           C
ATOM    139  C   ARG A  35       3.971 -10.221   7.251 22.35  0.00           C
ATOM    140  O   ARG A  35       5.151 -10.042   6.901 22.35  0.00           O
ATOM    141  N   VAL A  36       3.228  -9.297   7.848 50.49  0.00           N
ATOM    142  CA  VAL A  36       3.791  -7.981   8.175 50.49  0.00           C
ATOM    143  C   VAL A  36       4.864  -8.200   9.230 50.49  0.00           C
ATOM    144  O   VAL A  36       5.972  -7.734   9.085 50.49  0.00           O
ATOM    145  N   ARG A  37       4.561  -8.972  10.259 50.75  0.00           N
ATOM    146  CA  ARG A  37       5.600  -9.259  11.227 50.75  0.00           C
ATOM    147  C   ARG A  37       6.749 -10.045  10.630 50.75  0.00           C
ATOM    148  O   ARG A  37       7.879  -9.890  11.061 50.75  0.00           O
ATOM    149  N   TYR A  38       6.481 -10.904   9.649 51.51  0.00           N
ATOM    150  CA  TYR A  38       7.570 -11.660   9.036 51.51  0.00           C
ATOM    151  C   TYR A  38       8.499 -10.724   8.363 51.51  0.00           C
ATOM    152  O   TYR A  38       9.689 -10.942   8.377 51.51  0.00           O
ATOM    153  N   GLU A  39       7.963  -9.678   7.760 73.67  0.00           N
ATOM    154  CA  GLU A  39       8.816  -8.667   7.126 73.67  0.00           C
ATOM    155  C   GLU A  39       9.769  -7.904   8.077 73.67  0.00           C
ATOM    156  O   GLU A  39      10.773  -7.394   7.597 73.67  0.00           O
ATOM    157  N   SER A  40       9.464  -7.844   9.380 55.15  0.00           N
ATOM    158  CA  SER A  40      10.398  -7.338  10.412 55.15  0.00           C
ATOM    159  C   SER A  40      11.345  -8.393  10.958 55.15  0.00           C
ATOM    160  O   SER A  40      12.540  -8.130  11.114 55.15  0.00           O
ATOM    161  N   GLU A  41      10.808  -9.578  11.263 47.58  0.00           N
ATOM    162  CA  GLU A  41      11.604 -10.637  11.865 47.58  0.00           C
ATOM    163  C   GLU A  41      12.456 -11.415  10.889 47.58  0.00           C
ATOM    164  O   GLU A  41      13.321 -12.135  11.370 47.58  0.00           O
ATOM    165  N   TYR A  42      12.238 -11.336   9.566 46.48  0.00           N
ATOM    166  CA  TYR A  42      13.052 -12.135   8.586 46.48  0.00           C
ATOM    167  C   TYR A  42      13.369 -11.290   7.378 46.48  0.00           C
ATOM    168  O   TYR A  42      13.024 -11.599   6.196 46.48  0.00           O
ATOM    169  N   LYS A  43      14.059 -10.210   7.691 61.04  0.00           N
ATOM    170  CA  LYS A  43      14.286  -9.169   6.756 61.04  0.00           C
ATOM    171  C   LYS A  43      15.175  -9.664   5.651 61.04  0.00           C
ATOM    172  O   LYS A  43      15.118  -9.127   4.534 61.04  0.00           O
ATOM    173  N   GLU A  44      16.024 -10.635   5.936 57.96  0.00           N
ATOM    174  CA  GLU A  44      16.906 -11.087   4.887 57.96  0.00           C
ATOM    175  C   GLU A  44      16.077 -11.799   3.791 57.96  0.00           C
ATOM    176  O   GLU A  44      16.309 -11.615   2.572 57.96  0.00           O
ATOM    177  N   LEU A  45      15.070 -12.551   4.234 52.74  0.00           N
ATOM    178  CA  LEU A  45      14.177 -13.244   3.311 52.74  0.00           C
ATOM    179  C   LEU A  45      13.268 -12.185   2.642 52.74  0.00           C
ATOM    180  O   LEU A  45      13.095 -12.177   1.406 52.74  0.00           O
ATOM    181  N   ALA A  46      12.710 -11.278   3.448 72.00  0.00           N
ATOM    182  CA  ALA A  46      11.893 -10.178   2.897 72.00  0.00           C
ATOM    183  C   ALA A  46      12.519  -9.534   1.649 72.00  0.00           C
ATOM    184  O   ALA A  46      11.825  -9.262   0.709 72.00  0.00           O
ATOM    185  N   ILE A  47      13.830  -9.341   1.634 59.42  0.00           N
ATOM    186  CA  ILE A  47      14.526  -8.691   0.538 59.42  0.00           C
ATOM    187  C   ILE A  47      14.299  -9.332  -0.838 59.42  0.00           C
ATOM    188  O   ILE A  47      14.451  -8.669  -1.895 59.42  0.00           O
ATOM    189  N   SER A  48      13.959 -10.620  -0.874 66.56  0.00           N
ATOM    190  CA  SER A  48      13.715 -11.277  -2.169 66.56  0.00           C
ATOM    191  C   SER A  48      12.509 -10.666  -2.882 66.56  0.00           C
ATOM    192  O   SER A  48      12.401 -10.723  -4.108 66.56  0.00           O
```

ATOM	193	N	GLN	A	49	11.610	-10.068	-2.123	62.34	0.00	N
ATOM	194	CA	GLN	A	49	10.459	-9.434	-2.743	62.34	0.00	C
ATOM	195	C	GLN	A	49	10.847	-8.226	-3.623	62.34	0.00	C
ATOM	196	O	GLN	A	49	10.116	-7.889	-4.482	62.34	0.00	O
ATOM	197	N	VAL	A	50	12.031	-7.643	-3.424	62.33	0.00	N
ATOM	198	CA	VAL	A	50	12.659	-6.700	-4.358	62.33	0.00	C
ATOM	199	C	VAL	A	50	12.750	-7.176	-5.802	62.33	0.00	C
ATOM	200	O	VAL	A	50	12.805	-6.373	-6.754	62.33	0.00	O
ATOM	201	N	TYR	A	51	12.865	-8.468	-5.991	23.81	0.00	N
ATOM	202	CA	TYR	A	51	13.231	-8.953	-7.306	23.81	0.00	C
ATOM	203	C	TYR	A	51	12.000	-9.515	-8.013	23.81	0.00	C
ATOM	204	O	TYR	A	51	12.116	-10.179	-9.031	23.81	0.00	O
ATOM	205	N	ALA	A	52	10.823	-9.258	-7.445	43.17	0.00	N
ATOM	206	CA	ALA	A	52	9.580	-9.586	-8.075	43.17	0.00	C
ATOM	207	C	ALA	A	52	9.375	-8.694	-9.336	43.17	0.00	C
ATOM	208	O	ALA	A	52	9.152	-7.478	-9.216	43.17	0.00	O
ATOM	209	N	PRO	A	53	9.401	-9.282	-10.541	43.74	0.00	N
ATOM	210	CA	PRO	A	53	9.338	-8.519	-11.804	43.74	0.00	C
ATOM	211	C	PRO	A	53	8.051	-7.715	-11.969	43.74	0.00	C
ATOM	212	O	PRO	A	53	8.107	-6.662	-12.589	43.74	0.00	O
ATOM	213	N	SER	A	54	6.912	-8.185	-11.428	38.09	0.00	N
ATOM	214	CA	SER	A	54	5.662	-7.382	-11.372	38.09	0.00	C
ATOM	215	C	SER	A	54	5.807	-6.143	-10.551	38.09	0.00	C
ATOM	216	O	SER	A	54	5.159	-5.116	-10.806	38.09	0.00	O
ATOM	217	N	ALA	A	55	6.666	-6.210	-9.534	38.71	0.00	N
ATOM	218	CA	ALA	A	55	6.871	-5.023	-8.707	38.71	0.00	C
ATOM	219	C	ALA	A	55	7.756	-4.055	-9.490	38.71	0.00	C
ATOM	220	O	ALA	A	55	7.598	-2.860	-9.403	38.71	0.00	O
ATOM	221	N	ILE	A	56	8.662	-4.543	-10.314	47.01	0.00	N
ATOM	222	CA	ILE	A	56	9.562	-3.610	-11.005	47.01	0.00	C
ATOM	223	C	ILE	A	56	8.724	-2.964	-12.115	47.01	0.00	C
ATOM	224	O	ILE	A	56	8.790	-1.755	-12.348	47.01	0.00	O
ATOM	225	N	LEU	A	57	7.912	-3.805	-12.760	30.78	0.00	N
ATOM	226	CA	LEU	A	57	6.935	-3.366	-13.747	30.78	0.00	C
ATOM	227	C	LEU	A	57	6.033	-2.336	-13.087	30.78	0.00	C
ATOM	228	O	LEU	A	57	5.874	-1.207	-13.590	30.78	0.00	O
ATOM	229	N	GLY	A	58	5.483	-2.701	-11.946	41.70	0.00	N
ATOM	230	CA	GLY	A	58	4.634	-1.806	-11.182	41.70	0.00	C
ATOM	231	C	GLY	A	58	5.258	-0.473	-10.875	41.70	0.00	C
ATOM	232	O	GLY	A	58	4.607	0.560	-11.036	41.70	0.00	O
ATOM	233	N	ASP	A	59	6.514	-0.439	-10.443	47.60	0.00	N
ATOM	234	CA	ASP	A	59	7.150	0.844	-10.105	47.60	0.00	C
ATOM	235	C	ASP	A	59	7.486	1.775	-11.275	47.60	0.00	C
ATOM	236	O	ASP	A	59	7.461	2.943	-11.121	47.60	0.00	O
ATOM	237	N	ILE	A	60	7.874	1.212	-12.405	48.47	0.00	N
ATOM	238	CA	ILE	A	60	8.072	1.923	-13.651	48.47	0.00	C
ATOM	239	C	ILE	A	60	6.774	2.586	-14.133	48.47	0.00	C
ATOM	240	O	ILE	A	60	6.798	3.761	-14.559	48.47	0.00	O
ATOM	241	N	LEU	A	61	5.641	1.870	-14.058	35.67	0.00	N
ATOM	242	CA	LEU	A	61	4.334	2.454	-14.392	35.67	0.00	C
ATOM	243	C	LEU	A	61	4.024	3.653	-13.537	35.67	0.00	C
ATOM	244	O	LEU	A	61	3.668	4.678	-14.050	35.67	0.00	O
ATOM	245	N	HIS	A	62	4.172	3.508	-12.232	37.96	0.00	N
ATOM	246	CA	HIS	A	62	3.968	4.616	-11.306	37.96	0.00	C
ATOM	247	C	HIS	A	62	4.884	5.714	-11.684	37.96	0.00	C
ATOM	248	O	HIS	A	62	4.443	6.854	-11.841	37.96	0.00	O
ATOM	249	N	LEU	A	63	6.158	5.423	-11.888	34.78	0.00	N
ATOM	250	CA	LEU	A	63	7.034	6.526	-12.325	34.78	0.00	C
ATOM	251	C	LEU	A	63	6.405	7.217	-13.537	34.78	0.00	C
ATOM	252	O	LEU	A	63	6.167	8.394	-13.497	34.78	0.00	O
ATOM	253	N	GLY	A	64	6.109	6.499	-14.603	36.31	0.00	N
ATOM	254	CA	GLY	A	64	5.719	7.158	-15.811	36.31	0.00	C
ATOM	255	C	GLY	A	64	4.400	7.890	-15.740	36.31	0.00	C
ATOM	256	O	GLY	A	64	4.174	8.755	-16.509	36.31	0.00	O
ATOM	257	N	LEU	A	65	3.499	7.454	-14.886	17.94	0.00	N
ATOM	258	CA	LEU	A	65	2.129	7.928	-14.922	17.94	0.00	C
ATOM	259	C	LEU	A	65	2.136	9.075	-13.968	17.94	0.00	C
ATOM	260	O	LEU	A	65	1.699	10.153	-14.266	17.94	0.00	O
ATOM	261	N	GLU	A	66	2.672	8.845	-12.789	54.01	0.00	N
ATOM	262	CA	GLU	A	66	3.061	9.970	-11.943	54.01	0.00	C
ATOM	263	C	GLU	A	66	3.702	11.154	-12.688	54.01	0.00	C
ATOM	264	O	GLU	A	66	3.297	12.272	-12.436	54.01	0.00	O
ATOM	265	N	SER	A	67	4.665	10.981	-13.581	46.40	0.00	N
ATOM	266	CA	SER	A	67	5.183	12.221	-14.250	46.40	0.00	C
ATOM	267	C	SER	A	67	4.100	13.012	-15.067	46.40	0.00	C
ATOM	268	O	SER	A	67	4.182	14.248	-15.203	46.40	0.00	O
ATOM	269	N	VAL	A	68	3.091	12.293	-15.600	51.69	0.00	N
ATOM	270	CA	VAL	A	68	1.939	12.934	-16.238	51.69	0.00	C
ATOM	271	C	VAL	A	68	1.158	13.773	-15.231	51.69	0.00	C
ATOM	272	O	VAL	A	68	0.645	14.865	-15.520	51.69	0.00	O
ATOM	273	N	LEU	A	69	1.040	13.216	-14.047	50.04	0.00	N
ATOM	274	CA	LEU	A	69	0.261	13.818	-13.004	50.04	0.00	C
ATOM	275	C	LEU	A	69	0.848	15.118	-12.430	50.04	0.00	C
ATOM	276	O	LEU	A	69	0.134	15.992	-11.952	50.04	0.00	O
ATOM	277	N	LYS	A	70	2.165	15.215	-12.445	52.71	0.00	N
ATOM	278	CA	LYS	A	70	2.860	16.356	-11.911	52.71	0.00	C
ATOM	279	C	LYS	A	70	2.708	17.450	-12.934	52.71	0.00	C
ATOM	280	O	LYS	A	70	2.497	18.596	-12.562	52.71	0.00	O
ATOM	281	N	GLY	A	71	2.769	17.105	-14.229	49.67	0.00	N
ATOM	282	CA	GLY	A	71	2.550	18.111	-15.305	49.67	0.00	C
ATOM	283	C	GLY	A	71	1.130	18.685	-15.376	49.67	0.00	C
ATOM	284	O	GLY	A	71	0.821	19.815	-14.923	49.67	0.00	O

```
ATOM    285  N   ASN A  72       0.217  17.876 -15.876 49.24  0.00           N
ATOM    286  CA  ASN A  72      -1.095  18.370 -16.206 49.24  0.00           C
ATOM    287  C   ASN A  72      -2.040  18.612 -15.074 49.24  0.00           C
ATOM    288  O   ASN A  72      -2.921  19.420 -15.211 49.24  0.00           O
ATOM    289  N   PHE A  73      -1.880  17.933 -13.966 43.22  0.00           N
ATOM    290  CA  PHE A  73      -2.766  18.118 -12.847 43.22  0.00           C
ATOM    291  C   PHE A  73      -2.091  18.771 -11.639 43.22  0.00           C
ATOM    292  O   PHE A  73      -2.674  18.861 -10.593 43.22  0.00           O
ATOM    293  N   ASN A  74      -0.838  19.166 -11.735 60.11  0.00           N
ATOM    294  CA  ASN A  74      -0.201  19.781 -10.582 60.11  0.00           C
ATOM    295  C   ASN A  74      -0.355  18.949  -9.279 60.11  0.00           C
ATOM    296  O   ASN A  74      -0.590  19.474  -8.165 60.11  0.00           O
ATOM    297  N   ALA A  75      -0.160  17.641  -9.414 54.45  0.00           N
ATOM    298  CA  ALA A  75      -0.115  16.792  -8.245 54.45  0.00           C
ATOM    299  C   ALA A  75       1.282  16.777  -7.622 54.45  0.00           C
ATOM    300  O   ALA A  75       2.270  16.992  -8.289 54.45  0.00           O
ATOM    301  N   GLU A  76       1.336  16.530  -6.332 56.74  0.00           N
ATOM    302  CA  GLU A  76       2.559  16.069  -5.695 56.74  0.00           C
ATOM    303  C   GLU A  76       2.544  14.546  -5.834 56.74  0.00           C
ATOM    304  O   GLU A  76       1.466  13.957  -5.865 56.74  0.00           O
ATOM    305  N   THR A  77       3.710  13.915  -5.909 71.61  0.00           N
ATOM    306  CA  THR A  77       3.794  12.484  -6.087 71.61  0.00           C
ATOM    307  C   THR A  77       4.639  11.898  -4.978 71.61  0.00           C
ATOM    308  O   THR A  77       5.510  12.566  -4.500 71.61  0.00           O
ATOM    309  N   GLU A  78       4.321  10.666  -4.562 74.39  0.00           N
ATOM    310  CA  GLU A  78       4.988   9.920  -3.517 74.39  0.00           C
ATOM    311  C   GLU A  78       5.020  10.703  -2.232 74.39  0.00           C
ATOM    312  O   GLU A  78       6.063  10.936  -1.623 74.39  0.00           O
ATOM    313  N   VAL A  79       3.826  11.074  -1.816 55.67  0.00           N
ATOM    314  CA  VAL A  79       3.635  12.010  -0.767 55.67  0.00           C
ATOM    315  C   VAL A  79       3.474  11.303   0.544 55.67  0.00           C
ATOM    316  O   VAL A  79       2.510  10.573   0.774 55.67  0.00           O
ATOM    317  N   GLU A  80       4.416  11.580   1.421 51.09  0.00           N
ATOM    318  CA  GLU A  80       4.530  10.857   2.668 51.09  0.00           C
ATOM    319  C   GLU A  80       3.901  11.655   3.780 51.09  0.00           C
ATOM    320  O   GLU A  80       4.090  12.840   3.853 51.09  0.00           O
ATOM    321  N   THR A  81       3.177  11.027   4.670 43.26  0.00           N
ATOM    322  CA  THR A  81       2.510  11.744   5.761 43.26  0.00           C
ATOM    323  C   THR A  81       2.133  10.811   6.884 43.26  0.00           C
ATOM    324  O   THR A  81       2.164   9.597   6.754 43.26  0.00           O
ATOM    325  N   LEU A  82       1.667  11.373   7.976 50.29  0.00           N
ATOM    326  CA  LEU A  82       1.644  10.601   9.202 50.29  0.00           C
ATOM    327  C   LEU A  82       0.492  11.060  10.035 50.29  0.00           C
ATOM    328  O   LEU A  82       0.098  12.203   9.950 50.29  0.00           O
ATOM    329  N   ARG A  83      -0.124  10.141  10.761 18.69  0.00           N
ATOM    330  CA  ARG A  83      -1.326  10.449  11.518 18.69  0.00           C
ATOM    331  C   ARG A  83      -1.368   9.467  12.659 18.69  0.00           C
ATOM    332  O   ARG A  83      -0.926   8.327  12.512 18.69  0.00           O
ATOM    333  N   GLU A  84      -1.873   9.890  13.809 14.28  0.00           N
ATOM    334  CA  GLU A  84      -1.727   9.022  14.978 14.28  0.00           C
ATOM    335  C   GLU A  84      -3.042   8.425  15.425 14.28  0.00           C
ATOM    336  O   GLU A  84      -4.110   8.996  15.183 14.28  0.00           O
ATOM    337  N   ILE A  85      -2.918   7.275  16.095 56.26  0.00           N
ATOM    338  CA  ILE A  85      -4.029   6.451  16.449 56.26  0.00           C
ATOM    339  C   ILE A  85      -3.781   5.841  17.813 56.26  0.00           C
ATOM    340  O   ILE A  85      -2.643   5.507  18.123 56.26  0.00           O
ATOM    341  N   ASN A  86      -4.840   5.701  18.619 40.06  0.00           N
ATOM    342  CA  ASN A  86      -4.761   5.021  19.937 40.06  0.00           C
ATOM    343  C   ASN A  86      -5.250   3.566  19.895 40.06  0.00           C
ATOM    344  O   ASN A  86      -6.399   3.293  19.511 40.06  0.00           O
ATOM    345  N   VAL A  87      -4.383   2.645  20.322 50.74  0.00           N
ATOM    346  CA  VAL A  87      -4.703   1.227  20.308 50.74  0.00           C
ATOM    347  C   VAL A  87      -4.355   0.547  21.630 50.74  0.00           C
ATOM    348  O   VAL A  87      -3.230   0.067  21.819 50.74  0.00           O
ATOM    349  N   GLY A  88      -5.321   0.484  22.542 49.52  0.00           N
ATOM    350  CA  GLY A  88      -5.066  -0.115  23.865 49.52  0.00           C
ATOM    351  C   GLY A  88      -4.192   0.803  24.708 49.52  0.00           C
ATOM    352  O   GLY A  88      -3.216   0.373  25.351 49.52  0.00           O
ATOM    353  N   GLY A  89      -4.538   2.089  24.663 48.06  0.00           N
ATOM    354  CA  GLY A  89      -3.780   3.124  25.348 48.06  0.00           C
ATOM    355  C   GLY A  89      -2.339   3.328  24.890 48.06  0.00           C
ATOM    356  O   GLY A  89      -1.668   4.231  25.406 48.06  0.00           O
ATOM    357  N   LYS A  90      -1.846   2.502  23.956 49.69  0.00           N
ATOM    358  CA  LYS A  90      -0.547   2.733  23.318 49.69  0.00           C
ATOM    359  C   LYS A  90      -0.830   3.651  22.138 49.69  0.00           C
ATOM    360  O   LYS A  90      -1.881   3.541  21.497 49.69  0.00           O
ATOM    361  N   VAL A  91       0.069   4.592  21.872 50.28  0.00           N
ATOM    362  CA  VAL A  91      -0.106   5.503  20.744 50.28  0.00           C
ATOM    363  C   VAL A  91       0.675   4.963  19.561 50.28  0.00           C
ATOM    364  O   VAL A  91       1.817   4.523  19.727 50.28  0.00           O
ATOM    365  N   TYR A  92       0.052   5.005  18.377 61.33  0.00           N
ATOM    366  CA  TYR A  92       0.676   4.480  17.190 61.33  0.00           C
ATOM    367  C   TYR A  92       0.645   5.490  16.087 61.33  0.00           C
ATOM    368  O   TYR A  92      -0.329   6.191  15.893 61.33  0.00           O
ATOM    369  N   LYS A  93       1.747   5.547  15.371 75.56  0.00           N
ATOM    370  CA  LYS A  93       1.859   6.412  14.217 75.56  0.00           C
ATOM    371  C   LYS A  93       1.524   5.603  12.955 75.56  0.00           C
ATOM    372  O   LYS A  93       2.270   4.714  12.581 75.56  0.00           O
ATOM    373  N   ILE A  94       0.406   5.927  12.320 54.38  0.00           N
ATOM    374  CA  ILE A  94       0.084   5.372  11.038 54.38  0.00           C
ATOM    375  C   ILE A  94       0.743   6.184   9.929 54.38  0.00           C
ATOM    376  O   ILE A  94       0.556   7.385   9.807 54.38  0.00           O
```

ATOM	377	N	LYS	A	95	1.497	5.507	9.097	65.40	0.00	N
ATOM	378	CA	LYS	A	95	2.305	6.166	8.106	65.40	0.00	C
ATOM	379	C	LYS	A	95	1.950	5.702	6.699	65.40	0.00	C
ATOM	380	O	LYS	A	95	1.760	4.545	6.470	65.40	0.00	O
ATOM	381	N	GLY	A	96	1.853	6.613	5.743	50.04	0.00	N
ATOM	382	CA	GLY	A	96	1.572	6.242	4.383	50.04	0.00	C
ATOM	383	C	GLY	A	96	2.142	7.179	3.378	50.04	0.00	C
ATOM	384	O	GLY	A	96	2.537	8.280	3.703	50.04	0.00	O
ATOM	385	N	ARG	A	97	2.107	6.740	2.138	43.71	0.00	N
ATOM	386	CA	ARG	A	97	2.693	7.428	1.007	43.71	0.00	C
ATOM	387	C	ARG	A	97	1.769	7.206	-0.178	43.71	0.00	C
ATOM	388	O	ARG	A	97	1.672	6.084	-0.699	43.71	0.00	O
ATOM	389	N	ALA	A	98	1.104	8.281	-0.588	45.35	0.00	N
ATOM	390	CA	ALA	A	98	0.117	8.243	-1.592	45.35	0.00	C
ATOM	391	C	ALA	A	98	0.862	8.383	-2.906	45.35	0.00	C
ATOM	392	O	ALA	A	98	1.855	9.097	-3.010	45.35	0.00	O
ATOM	393	N	ASP	A	99	0.424	7.673	-3.912	52.70	0.00	N
ATOM	394	CA	ASP	A	99	1.076	7.754	-5.196	52.70	0.00	C
ATOM	395	C	ASP	A	99	0.974	9.188	-5.720	52.70	0.00	C
ATOM	396	O	ASP	A	99	1.922	9.707	-6.350	52.70	0.00	O
ATOM	397	N	ALA	A	100	-0.166	9.846	-5.513	56.40	0.00	N
ATOM	398	CA	ALA	A	100	-0.251	11.269	-5.953	56.40	0.00	C
ATOM	399	C	ALA	A	100	-1.290	11.992	-5.184	56.40	0.00	C
ATOM	400	O	ALA	A	100	-2.232	11.366	-4.731	56.40	0.00	O
ATOM	401	N	ILE	A	101	-1.104	13.303	-4.998	52.13	0.00	N
ATOM	402	CA	ILE	A	101	-2.137	14.137	-4.384	52.13	0.00	C
ATOM	403	C	ILE	A	101	-2.390	15.420	-5.140	52.13	0.00	C
ATOM	404	O	ILE	A	101	-1.467	16.143	-5.558	52.13	0.00	O
ATOM	405	N	ILE	A	102	-3.672	15.695	-5.329	51.64	0.00	N
ATOM	406	CA	ILE	A	102	-4.083	17.004	-5.796	51.64	0.00	C
ATOM	407	C	ILE	A	102	-4.628	17.765	-4.581	51.64	0.00	C
ATOM	408	O	ILE	A	102	-5.695	17.431	-4.073	51.64	0.00	O
ATOM	409	N	ARG	A	103	-3.845	18.751	-4.103	49.29	0.00	N
ATOM	410	CA	ARG	A	103	-4.150	19.504	-2.879	49.29	0.00	C
ATOM	411	C	ARG	A	103	-5.423	20.391	-2.949	49.29	0.00	C
ATOM	412	O	ARG	A	103	-6.199	20.482	-1.960	49.29	0.00	O
ATOM	413	N	ASN	A	104	-5.626	21.051	-4.095	45.99	0.00	N
ATOM	414	CA	ASN	A	104	-6.745	21.998	-4.259	45.99	0.00	C
ATOM	415	C	ASN	A	104	-7.230	21.994	-5.679	45.99	0.00	C
ATOM	416	O	ASN	A	104	-6.693	22.692	-6.538	45.99	0.00	O
ATOM	417	N	ASP	A	105	-8.253	21.174	-5.879	43.08	0.00	N
ATOM	418	CA	ASP	A	105	-9.039	21.134	-7.084	43.08	0.00	C
ATOM	419	C	ASP	A	105	-10.471	21.605	-6.714	43.08	0.00	C
ATOM	420	O	ASP	A	105	-11.262	20.843	-6.158	43.08	0.00	O
ATOM	421	N	ASN	A	106	-10.768	22.878	-6.986	50.08	0.00	N
ATOM	422	CA	ASN	A	106	-12.040	23.506	-6.575	50.08	0.00	C
ATOM	423	C	ASN	A	106	-12.422	23.321	-5.104	50.08	0.00	C
ATOM	424	O	ASN	A	106	-13.561	23.020	-4.774	50.08	0.00	O
ATOM	425	N	GLY	A	107	-11.470	23.524	-4.215	44.36	0.00	N
ATOM	426	CA	GLY	A	107	-11.717	23.337	-2.789	44.36	0.00	C
ATOM	427	C	GLY	A	107	-11.737	21.883	-2.335	44.36	0.00	C
ATOM	428	O	GLY	A	107	-12.404	21.544	-1.352	44.36	0.00	O
ATOM	429	N	LYS	A	108	-11.008	21.019	-3.043	50.81	0.00	N
ATOM	430	CA	LYS	A	108	-10.915	19.615	-2.668	50.81	0.00	C
ATOM	431	C	LYS	A	108	-9.483	19.049	-2.884	50.81	0.00	C
ATOM	432	O	LYS	A	108	-8.779	19.403	-3.839	50.81	0.00	O
ATOM	433	N	SER	A	109	-9.039	18.236	-1.933	55.18	0.00	N
ATOM	434	CA	SER	A	109	-7.862	17.387	-2.118	55.18	0.00	C
ATOM	435	C	SER	A	109	-8.308	16.067	-2.739	55.18	0.00	C
ATOM	436	O	SER	A	109	-9.315	15.517	-2.353	55.18	0.00	O
ATOM	437	N	ILE	A	110	-7.524	15.551	-3.650	54.55	0.00	N
ATOM	438	CA	ILE	A	110	-7.790	14.265	-4.209	54.55	0.00	C
ATOM	439	C	ILE	A	110	-6.588	13.343	-3.999	54.55	0.00	C
ATOM	440	O	ILE	A	110	-5.512	13.629	-4.510	54.55	0.00	O
ATOM	441	N	VAL	A	111	-6.752	12.243	-3.266	56.94	0.00	N
ATOM	442	CA	VAL	A	111	-5.679	11.220	-3.198	56.94	0.00	C
ATOM	443	C	VAL	A	111	-5.836	10.276	-4.388	56.94	0.00	C
ATOM	444	O	VAL	A	111	-6.932	9.764	-4.636	56.94	0.00	O
ATOM	445	N	ILE	A	112	-4.775	10.018	-5.128	55.75	0.00	N
ATOM	446	CA	ILE	A	112	-4.866	9.126	-6.282	55.75	0.00	C
ATOM	447	C	ILE	A	112	-4.010	7.894	-6.056	55.75	0.00	C
ATOM	448	O	ILE	A	112	-2.842	8.019	-5.770	55.75	0.00	O
ATOM	449	N	GLU	A	113	-4.547	6.709	-6.243	47.46	0.00	N
ATOM	450	CA	GLU	A	113	-3.780	5.455	-6.060	47.46	0.00	C
ATOM	451	C	GLU	A	113	-3.823	4.710	-7.388	47.46	0.00	C
ATOM	452	O	GLU	A	113	-4.882	4.569	-8.052	47.46	0.00	O
ATOM	453	N	ILE	A	114	-2.657	4.354	-7.861	50.04	0.00	N
ATOM	454	CA	ILE	A	114	-2.531	3.758	-9.150	50.04	0.00	C
ATOM	455	C	ILE	A	114	-2.210	2.295	-8.960	50.04	0.00	C
ATOM	456	O	ILE	A	114	-1.423	1.954	-8.099	50.04	0.00	O
ATOM	457	N	LYS	A	115	-2.787	1.433	-9.786	48.70	0.00	N
ATOM	458	CA	LYS	A	115	-2.621	0.003	-9.616	48.70	0.00	C
ATOM	459	C	LYS	A	115	-2.438	-0.643	-10.947	48.70	0.00	C
ATOM	460	O	LYS	A	115	-2.964	-0.176	-11.951	48.70	0.00	O
ATOM	461	N	THR	A	116	-1.684	-1.729	-10.958	39.30	0.00	N
ATOM	462	CA	THR	A	116	-1.468	-2.466	-12.194	39.30	0.00	C
ATOM	463	C	THR	A	116	-1.602	-3.921	-11.890	39.30	0.00	C
ATOM	464	O	THR	A	116	-1.369	-4.337	-10.804	39.30	0.00	O
ATOM	465	N	SER	A	117	-2.085	-4.706	-12.802	35.66	0.00	N
ATOM	466	CA	SER	A	117	-2.109	-6.154	-12.601	35.66	0.00	C
ATOM	467	C	SER	A	117	-2.180	-6.692	-13.946	35.66	0.00	C
ATOM	468	O	SER	A	117	-2.324	-5.911	-14.909	35.66	0.00	O

ATOM	469	N	ARG	A	118	-2.032	-8.006	-14.033	2.77	0.00	N
ATOM	470	CA	ARG	A	118	-2.068	-8.640	-15.363	2.77	0.00	C
ATOM	471	C	ARG	A	118	-3.471	-8.928	-15.862	2.77	0.00	C
ATOM	472	O	ARG	A	118	-3.648	-9.162	-17.069	2.77	0.00	O
ATOM	473	N	SER	A	119	-4.457	-8.893	-14.965	43.98	0.00	N
ATOM	474	CA	SER	A	119	-5.872	-9.062	-15.362	43.98	0.00	C
ATOM	475	C	SER	A	119	-6.800	-7.967	-14.770	43.98	0.00	C
ATOM	476	O	SER	A	119	-6.533	-7.439	-13.690	43.98	0.00	O
ATOM	477	N	ASP	A	120	-7.938	-7.766	-15.424	48.09	0.00	N
ATOM	478	CA	ASP	A	120	-8.922	-6.789	-15.023	48.09	0.00	C
ATOM	479	C	ASP	A	120	-10.062	-7.464	-14.339	48.09	0.00	C
ATOM	480	O	ASP	A	120	-11.180	-6.930	-14.286	48.09	0.00	O
ATOM	481	N	LYS	A	121	-9.805	-8.622	-13.800	17.69	0.00	N
ATOM	482	CA	LYS	A	121	-10.861	-9.343	-13.147	17.69	0.00	C
ATOM	483	C	LYS	A	121	-11.305	-8.719	-11.850	17.69	0.00	C
ATOM	484	O	LYS	A	121	-10.493	-8.415	-10.994	17.69	0.00	O
ATOM	485	N	GLY	A	122	-12.595	-8.552	-11.661	51.98	0.00	N
ATOM	486	CA	GLY	A	122	-13.099	-8.150	-10.341	51.98	0.00	C
ATOM	487	C	GLY	A	122	-12.816	-6.695	-9.897	51.98	0.00	C
ATOM	488	O	GLY	A	122	-13.062	-6.363	-8.702	51.98	0.00	O
ATOM	489	N	LEU	A	123	-12.369	-5.819	-10.826	66.67	0.00	N
ATOM	490	CA	LEU	A	123	-12.047	-4.418	-10.440	66.67	0.00	C
ATOM	491	C	LEU	A	123	-13.261	-3.672	-9.972	66.67	0.00	C
ATOM	492	O	LEU	A	123	-14.252	-3.694	-10.654	66.67	0.00	O
ATOM	493	N	PRO	A	124	-13.153	-2.951	-8.838	59.92	0.00	N
ATOM	494	CA	PRO	A	124	-11.936	-2.786	-8.048	59.92	0.00	C
ATOM	495	C	PRO	A	124	-11.854	-3.776	-6.934	59.92	0.00	C
ATOM	496	O	PRO	A	124	-12.861	-4.122	-6.314	59.92	0.00	O
ATOM	497	N	LEU	A	125	-10.645	-4.184	-6.634	44.28	0.00	N
ATOM	498	CA	LEU	A	125	-10.394	-5.171	-5.542	44.28	0.00	C
ATOM	499	C	LEU	A	125	-10.497	-4.530	-4.149	44.28	0.00	C
ATOM	500	O	LEU	A	125	-10.112	-3.350	-3.908	44.28	0.00	O
ATOM	501	N	ILE	A	126	-11.048	-5.276	-3.221	45.33	0.00	N
ATOM	502	CA	ILE	A	126	-11.528	-4.647	-1.996	45.33	0.00	C
ATOM	503	C	ILE	A	126	-10.339	-4.126	-1.167	45.33	0.00	C
ATOM	504	O	ILE	A	126	-10.462	-3.123	-0.513	45.33	0.00	O
ATOM	505	N	HIS	A	127	-9.195	-4.779	-1.251	35.63	0.00	N
ATOM	506	CA	HIS	A	127	-8.055	-4.383	-0.479	35.63	0.00	C
ATOM	507	C	HIS	A	127	-7.440	-3.163	-1.103	35.63	0.00	C
ATOM	508	O	HIS	A	127	-6.834	-2.382	-0.415	35.63	0.00	O
ATOM	509	N	HIS	A	128	-7.592	-2.980	-2.400	39.03	0.00	N
ATOM	510	CA	HIS	A	128	-7.176	-1.718	-3.018	39.03	0.00	C
ATOM	511	C	HIS	A	128	-8.083	-0.558	-2.609	39.03	0.00	C
ATOM	512	O	HIS	A	128	-7.606	0.554	-2.368	39.03	0.00	O
ATOM	513	N	LYS	A	129	-9.365	-0.803	-2.526	61.21	0.00	N
ATOM	514	CA	LYS	A	129	-10.266	0.264	-2.198	61.21	0.00	C
ATOM	515	C	LYS	A	129	-10.028	0.593	-0.745	61.21	0.00	C
ATOM	516	O	LYS	A	129	-9.930	1.747	-0.408	61.21	0.00	O
ATOM	517	N	MSE	A	130	-9.911	-0.410	0.128	46.12	0.00	N
ATOM	518	CA	MSE	A	130	-9.661	-0.122	1.564	46.12	0.00	C
ATOM	519	C	MSE	A	130	-8.432	0.772	1.739	46.12	0.00	C
ATOM	520	O	MSE	A	130	-8.445	1.649	2.550	46.12	0.00	O
ATOM	521	N	GLN	A	131	-7.394	0.520	0.958	56.30	0.00	N
ATOM	522	CA	GLN	A	131	-6.133	1.211	1.097	56.30	0.00	C
ATOM	523	C	GLN	A	131	-6.400	2.668	0.780	56.30	0.00	C
ATOM	524	O	GLN	A	131	-5.918	3.542	1.460	56.30	0.00	O
ATOM	525	N	LEU	A	132	-7.195	2.936	-0.242	53.92	0.00	N
ATOM	526	CA	LEU	A	132	-7.436	4.290	-0.682	53.92	0.00	C
ATOM	527	C	LEU	A	132	-8.295	4.984	0.347	53.92	0.00	C
ATOM	528	O	LEU	A	132	-8.142	6.173	0.629	53.92	0.00	O
ATOM	529	N	GLN	A	133	-9.173	4.244	0.963	50.81	0.00	N
ATOM	530	CA	GLN	A	133	-10.034	4.835	1.951	50.81	0.00	C
ATOM	531	C	GLN	A	133	-9.306	5.181	3.263	50.81	0.00	C
ATOM	532	O	GLN	A	133	-9.739	6.050	4.021	50.81	0.00	O
ATOM	533	N	ILE	A	134	-8.240	4.450	3.561	59.98	0.00	N
ATOM	534	CA	ILE	A	134	-7.448	4.750	4.709	59.98	0.00	C
ATOM	535	C	ILE	A	134	-6.635	6.008	4.375	59.98	0.00	C
ATOM	536	O	ILE	A	134	-6.604	6.904	5.182	59.98	0.00	O
ATOM	537	N	TYR	A	135	-5.993	6.089	3.200	55.08	0.00	N
ATOM	538	CA	TYR	A	135	-5.308	7.299	2.832	55.08	0.00	C
ATOM	539	C	TYR	A	135	-6.263	8.486	3.003	55.08	0.00	C
ATOM	540	O	TYR	A	135	-5.876	9.536	3.592	55.08	0.00	O
ATOM	541	N	LEU	A	136	-7.510	8.345	2.563	49.84	0.00	N
ATOM	542	CA	LEU	A	136	-8.431	9.472	2.663	49.84	0.00	C
ATOM	543	C	LEU	A	136	-8.471	9.988	4.123	49.84	0.00	C
ATOM	544	O	LEU	A	136	-8.423	11.193	4.364	49.84	0.00	O
ATOM	545	N	TRP	A	137	-8.562	9.085	5.079	49.66	0.00	N
ATOM	546	CA	TRP	A	137	-8.471	9.465	6.480	49.66	0.00	C
ATOM	547	C	TRP	A	137	-7.090	10.092	6.781	49.66	0.00	C
ATOM	548	O	TRP	A	137	-6.988	11.182	7.363	49.66	0.00	O
ATOM	549	N	LEU	A	138	-6.039	9.457	6.283	53.75	0.00	N
ATOM	550	CA	LEU	A	138	-4.665	9.881	6.616	53.75	0.00	C
ATOM	551	C	LEU	A	138	-4.353	11.332	6.246	53.75	0.00	C
ATOM	552	O	LEU	A	138	-3.806	12.080	7.039	53.75	0.00	O
ATOM	553	N	PHE	A	139	-4.725	11.691	5.030	48.93	0.00	N
ATOM	554	CA	PHE	A	139	-4.602	13.020	4.511	48.93	0.00	C
ATOM	555	C	PHE	A	139	-5.790	13.890	4.834	48.93	0.00	C
ATOM	556	O	PHE	A	139	-5.772	15.074	4.508	48.93	0.00	O
ATOM	557	N	SER	A	140	-6.827	13.321	5.474	50.30	0.00	N
ATOM	558	CA	SER	A	140	-8.140	13.999	5.608	50.30	0.00	C
ATOM	559	C	SER	A	140	-8.590	14.636	4.304	50.30	0.00	C
ATOM	560	O	SER	A	140	-8.961	15.800	4.273	50.30	0.00	O

ATOM	561	N	ALA	A	141	-8.519	13.878	3.227	48.21	0.00	N
ATOM	562	CA	ALA	A	141	-8.945	14.332	1.917	48.21	0.00	C
ATOM	563	C	ALA	A	141	-10.405	13.939	1.667	48.21	0.00	C
ATOM	564	O	ALA	A	141	-10.983	13.084	2.348	48.21	0.00	O
ATOM	565	N	GLU	A	142	-11.035	14.621	0.722	28.93	0.00	N
ATOM	566	CA	GLU	A	142	-12.471	14.427	0.489	28.93	0.00	C
ATOM	567	C	GLU	A	142	-12.738	13.477	-0.702	28.93	0.00	C
ATOM	568	O	GLU	A	142	-13.747	12.791	-0.707	28.93	0.00	O
ATOM	569	N	LYS	A	143	-11.805	13.444	-1.660	52.21	0.00	N
ATOM	570	CA	LYS	A	143	-11.916	12.652	-2.862	52.21	0.00	C
ATOM	571	C	LYS	A	143	-10.727	11.686	-3.055	52.21	0.00	C
ATOM	572	O	LYS	A	143	-9.539	11.991	-2.732	52.21	0.00	O
ATOM	573	N	GLY	A	144	-11.077	10.526	-3.602	52.67	0.00	N
ATOM	574	CA	GLY	A	144	-10.120	9.474	-3.925	52.67	0.00	C
ATOM	575	C	GLY	A	144	-10.352	8.924	-5.300	52.67	0.00	C
ATOM	576	O	GLY	A	144	-11.486	8.740	-5.697	52.67	0.00	O
ATOM	577	N	ILE	A	145	-9.286	8.679	-6.041	51.81	0.00	N
ATOM	578	CA	ILE	A	145	-9.380	8.016	-7.325	51.81	0.00	C
ATOM	579	C	ILE	A	145	-8.437	6.791	-7.270	51.81	0.00	C
ATOM	580	O	ILE	A	145	-7.274	6.920	-6.924	51.81	0.00	O
ATOM	581	N	LEU	A	146	-8.955	5.621	-7.649	54.22	0.00	N
ATOM	582	CA	LEU	A	146	-8.233	4.396	-7.797	54.22	0.00	C
ATOM	583	C	LEU	A	146	-8.251	4.098	-9.279	54.22	0.00	C
ATOM	584	O	LEU	A	146	-9.304	3.843	-9.816	54.22	0.00	O
ATOM	585	N	VAL	A	147	-7.117	4.065	-9.937	56.90	0.00	N
ATOM	586	CA	VAL	A	147	-7.059	3.872	-11.356	56.90	0.00	C
ATOM	587	C	VAL	A	147	-6.211	2.623	-11.646	56.90	0.00	C
ATOM	588	O	VAL	A	147	-5.067	2.470	-11.090	56.90	0.00	O
ATOM	589	N	TYR	A	148	-6.742	1.739	-12.509	56.66	0.00	N
ATOM	590	CA	TYR	A	148	-6.090	0.482	-12.844	56.66	0.00	C
ATOM	591	C	TYR	A	148	-5.595	0.575	-14.257	56.66	0.00	C
ATOM	592	O	TYR	A	148	-6.345	0.903	-15.117	56.66	0.00	O
ATOM	593	N	ILE	A	149	-4.338	0.293	-14.483	48.41	0.00	N
ATOM	594	CA	ILE	A	149	-3.766	0.206	-15.807	48.41	0.00	C
ATOM	595	C	ILE	A	149	-3.430	-1.275	-15.990	48.41	0.00	C
ATOM	596	O	ILE	A	149	-2.545	-1.776	-15.346	48.41	0.00	O
ATOM	597	N	THR	A	150	-4.180	-2.003	-16.800	51.51	0.00	N
ATOM	598	CA	THR	A	150	-3.964	-3.413	-16.968	51.51	0.00	C
ATOM	599	C	THR	A	150	-3.936	-3.690	-18.433	51.51	0.00	C
ATOM	600	O	THR	A	150	-4.311	-2.846	-19.222	51.51	0.00	O
ATOM	601	N	PRO	A	151	-3.486	-4.859	-18.836	42.72	0.00	N
ATOM	602	CA	PRO	A	151	-3.299	-5.051	-20.300	42.72	0.00	C
ATOM	603	C	PRO	A	151	-4.601	-5.273	-21.077	42.72	0.00	C
ATOM	604	O	PRO	A	151	-4.598	-5.259	-22.317	42.72	0.00	O
ATOM	605	N	ASP	A	152	-5.658	-5.505	-20.333	49.44	0.00	N
ATOM	606	CA	ASP	A	152	-6.988	-5.753	-20.794	49.44	0.00	C
ATOM	607	C	ASP	A	152	-7.910	-4.564	-20.707	49.44	0.00	C
ATOM	608	O	ASP	A	152	-8.921	-4.561	-21.326	49.44	0.00	O
ATOM	609	N	ARG	A	153	-7.550	-3.582	-19.909	50.16	0.00	N
ATOM	610	CA	ARG	A	153	-8.418	-2.528	-19.517	50.16	0.00	C
ATOM	611	C	ARG	A	153	-7.808	-1.437	-18.658	50.16	0.00	C
ATOM	612	O	ARG	A	153	-7.169	-1.665	-17.715	50.16	0.00	O
ATOM	613	N	ILE	A	154	-8.107	-0.221	-18.973	49.79	0.00	N
ATOM	614	CA	ILE	A	154	-7.873	0.874	-18.098	49.79	0.00	C
ATOM	615	C	ILE	A	154	-9.177	1.197	-17.432	49.79	0.00	C
ATOM	616	O	ILE	A	154	-10.135	1.182	-18.066	49.79	0.00	O
ATOM	617	N	ALA	A	155	-9.204	1.463	-16.150	50.43	0.00	N
ATOM	618	CA	ALA	A	155	-10.490	1.563	-15.403	50.43	0.00	C
ATOM	619	C	ALA	A	155	-10.307	2.411	-14.178	50.43	0.00	C
ATOM	620	O	ALA	A	155	-9.405	2.165	-13.464	50.43	0.00	O
ATOM	621	N	GLU	A	156	-11.159	3.389	-13.945	50.47	0.00	N
ATOM	622	CA	GLU	A	156	-10.959	4.392	-12.924	50.47	0.00	C
ATOM	623	C	GLU	A	156	-12.193	4.455	-12.023	50.47	0.00	C
ATOM	624	O	GLU	A	156	-13.299	4.455	-12.446	50.47	0.00	O
ATOM	625	N	TYR	A	157	-11.976	4.532	-10.746	38.52	0.00	N
ATOM	626	CA	TYR	A	157	-13.044	4.493	-9.764	38.52	0.00	C
ATOM	627	C	TYR	A	157	-12.934	5.658	-8.804	38.52	0.00	C
ATOM	628	O	TYR	A	157	-11.853	6.011	-8.265	38.52	0.00	O
ATOM	629	N	GLU	A	158	-14.055	6.253	-8.542	47.79	0.00	N
ATOM	630	CA	GLU	A	158	-14.108	7.361	-7.646	47.79	0.00	C
ATOM	631	C	GLU	A	158	-14.532	6.786	-6.302	47.79	0.00	C
ATOM	632	O	GLU	A	158	-15.602	6.184	-6.178	47.79	0.00	O
ATOM	633	N	ILE	A	159	-13.651	6.902	-5.311	53.24	0.00	N
ATOM	634	CA	ILE	A	159	-13.878	6.419	-3.939	53.24	0.00	C
ATOM	635	C	ILE	A	159	-13.755	7.649	-3.027	53.24	0.00	C
ATOM	636	O	ILE	A	159	-12.671	8.212	-2.918	53.24	0.00	O
ATOM	637	N	ASN	A	160	-14.849	8.038	-2.394	60.63	0.00	N
ATOM	638	CA	ASN	A	160	-14.894	9.274	-1.638	60.63	0.00	C
ATOM	639	C	ASN	A	160	-15.165	9.116	-0.146	60.63	0.00	C
ATOM	640	O	ASN	A	160	-14.949	10.056	0.594	60.63	0.00	O
ATOM	641	N	GLU	A	161	-15.536	7.926	0.306	50.70	0.00	N
ATOM	642	CA	GLU	A	161	-15.720	7.704	1.724	50.70	0.00	C
ATOM	643	C	GLU	A	161	-14.467	7.151	2.407	50.70	0.00	C
ATOM	644	O	GLU	A	161	-13.997	6.052	2.116	50.70	0.00	O
ATOM	645	N	PRO	A	162	-13.950	7.900	3.365	52.96	0.00	N
ATOM	646	CA	PRO	A	162	-12.848	7.426	4.187	52.96	0.00	C
ATOM	647	C	PRO	A	162	-13.323	6.439	5.227	52.96	0.00	C
ATOM	648	O	PRO	A	162	-14.483	6.537	5.645	52.96	0.00	O
ATOM	649	N	LEU	A	163	-12.444	5.517	5.661	52.16	0.00	N
ATOM	650	CA	LEU	A	163	-12.805	4.572	6.724	52.16	0.00	C
ATOM	651	C	LEU	A	163	-12.888	5.346	8.019	52.16	0.00	C
ATOM	652	O	LEU	A	163	-12.040	6.223	8.266	52.16	0.00	O

```
ATOM    653  N   ASP A 164     -13.876   5.013   8.858 57.09  0.00           N
ATOM    654  CA  ASP A 164     -13.934   5.590  10.207 57.09  0.00           C
ATOM    655  C   ASP A 164     -12.616   5.280  10.928 57.09  0.00           C
ATOM    656  O   ASP A 164     -11.940   4.300  10.622 57.09  0.00           O
ATOM    657  N   GLU A 165     -12.301   6.090  11.926 61.08  0.00           N
ATOM    658  CA  GLU A 165     -11.127   5.880  12.758 61.08  0.00           C
ATOM    659  C   GLU A 165     -11.240   4.594  13.565 61.08  0.00           C
ATOM    660  O   GLU A 165     -10.265   3.923  13.761 61.08  0.00           O
ATOM    661  N   ALA A 166     -12.438   4.252  14.016 53.69  0.00           N
ATOM    662  CA  ALA A 166     -12.662   3.006  14.753 53.69  0.00           C
ATOM    663  C   ALA A 166     -12.336   1.750  13.921 53.69  0.00           C
ATOM    664  O   ALA A 166     -11.761   0.808  14.415 53.69  0.00           O
ATOM    665  N   THR A 167     -12.694   1.751  12.648 50.62  0.00           N
ATOM    666  CA  THR A 167     -12.382   0.638  11.768 50.62  0.00           C
ATOM    667  C   THR A 167     -10.872   0.497  11.623 50.62  0.00           C
ATOM    668  O   THR A 167     -10.346  -0.601  11.637 50.62  0.00           O
ATOM    669  N   ILE A 168     -10.171   1.619  11.528 56.16  0.00           N
ATOM    670  CA  ILE A 168      -8.732   1.633  11.358 56.16  0.00           C
ATOM    671  C   ILE A 168      -8.078   1.128  12.626 56.16  0.00           C
ATOM    672  O   ILE A 168      -7.058   0.457  12.570 56.16  0.00           O
ATOM    673  N   VAL A 169      -8.665   1.441  13.778 58.37  0.00           N
ATOM    674  CA  VAL A 169      -8.132   0.957  15.026 58.37  0.00           C
ATOM    675  C   VAL A 169      -8.314  -0.550  15.039 58.37  0.00           C
ATOM    676  O   VAL A 169      -7.394  -1.257  15.440 58.37  0.00           O
ATOM    677  N   ARG A 170      -9.472  -1.045  14.590 59.48  0.00           N
ATOM    678  CA  ARG A 170      -9.700  -2.518  14.528 59.48  0.00           C
ATOM    679  C   ARG A 170      -8.661  -3.202  13.604 59.48  0.00           C
ATOM    680  O   ARG A 170      -8.069  -4.207  13.996 59.48  0.00           O
ATOM    681  N   LEU A 171      -8.396  -2.610  12.430 49.98  0.00           N
ATOM    682  CA  LEU A 171      -7.379  -3.127  11.527 49.98  0.00           C
ATOM    683  C   LEU A 171      -6.020  -3.139  12.210 49.98  0.00           C
ATOM    684  O   LEU A 171      -5.257  -4.108  12.109 49.98  0.00           O
ATOM    685  N   ALA A 172      -5.723  -2.049  12.909 58.47  0.00           N
ATOM    686  CA  ALA A 172      -4.457  -1.898  13.604 58.47  0.00           C
ATOM    687  C   ALA A 172      -4.301  -2.947  14.696 58.47  0.00           C
ATOM    688  O   ALA A 172      -3.240  -3.541  14.801 58.47  0.00           O
ATOM    689  N   GLU A 173      -5.352  -3.187  15.479 69.53  0.00           N
ATOM    690  CA  GLU A 173      -5.292  -4.177  16.568 69.53  0.00           C
ATOM    691  C   GLU A 173      -4.915  -5.559  16.053 69.53  0.00           C
ATOM    692  O   GLU A 173      -4.131  -6.255  16.707 69.53  0.00           O
ATOM    693  N   ASP A 174      -5.506  -5.961  14.919 57.67  0.00           N
ATOM    694  CA  ASP A 174      -5.208  -7.255  14.320 57.67  0.00           C
ATOM    695  C   ASP A 174      -3.815  -7.301  13.840 57.67  0.00           C
ATOM    696  O   ASP A 174      -3.169  -8.343  13.873 57.67  0.00           O
ATOM    697  N   THR A 175      -3.325  -6.165  13.384 51.60  0.00           N
ATOM    698  CA  THR A 175      -1.966  -6.171  12.887 51.60  0.00           C
ATOM    699  C   THR A 175      -1.026  -6.460  14.019 51.60  0.00           C
ATOM    700  O   THR A 175      -0.104  -7.267  13.894 51.60  0.00           O
ATOM    701  N   ILE A 176      -1.312  -5.827  15.139 44.53  0.00           N
ATOM    702  CA  ILE A 176      -0.456  -5.907  16.306 44.53  0.00           C
ATOM    703  C   ILE A 176      -0.618  -7.264  17.013 44.53  0.00           C
ATOM    704  O   ILE A 176       0.348  -7.982  17.191 44.53  0.00           O
ATOM    705  N   MSE A 177      -1.846  -7.622  17.359 55.85  0.00           N
ATOM    706  CA  MSE A 177      -2.110  -8.862  18.066 55.85  0.00           C
ATOM    707  C   MSE A 177      -2.036 -10.148  17.184 55.85  0.00           C
ATOM    708  O   MSE A 177      -2.230 -11.232  17.705 55.85  0.00           O
ATOM    709  N   LEU A 178      -1.747 -10.045  15.885 59.55  0.00           N
ATOM    710  CA  LEU A 178      -1.765 -11.195  14.931 59.55  0.00           C
ATOM    711  C   LEU A 178      -3.082 -11.987  14.963 59.55  0.00           C
ATOM    712  O   LEU A 178      -3.059 -13.216  14.908 59.55  0.00           O
ATOM    713  N   GLN A 179      -4.195 -11.295  14.914 54.12  0.00           N
ATOM    714  CA  GLN A 179      -5.488 -11.880  15.107 54.12  0.00           C
ATOM    715  C   GLN A 179      -6.016 -12.818  14.022 54.12  0.00           C
ATOM    716  O   GLN A 179      -6.283 -13.971  14.291 54.12  0.00           O
ATOM    717  N   ASN A 180      -6.209 -12.312  12.822 48.75  0.00           N
ATOM    718  CA  ASN A 180      -6.832 -13.100  11.723 48.75  0.00           C
ATOM    719  C   ASN A 180      -5.958 -13.170  10.495 48.75  0.00           C
ATOM    720  O   ASN A 180      -5.982 -12.302   9.632 48.75  0.00           O
ATOM    721  N   SER A 181      -5.162 -14.213  10.431 62.92  0.00           N
ATOM    722  CA  SER A 181      -4.294 -14.421   9.316 62.92  0.00           C
ATOM    723  C   SER A 181      -4.685 -15.651   8.495 62.92  0.00           C
ATOM    724  O   SER A 181      -4.793 -16.745   9.008 62.92  0.00           O
ATOM    725  N   PRO A 182      -4.770 -15.497   7.199 62.16  0.00           N
ATOM    726  CA  PRO A 182      -4.417 -14.305   6.461 62.16  0.00           C
ATOM    727  C   PRO A 182      -5.557 -13.337   6.500 62.16  0.00           C
ATOM    728  O   PRO A 182      -6.623 -13.675   7.004 62.16  0.00           O
ATOM    729  N   ARG A 183      -5.357 -12.135   5.998 55.41  0.00           N
ATOM    730  CA  ARG A 183      -6.436 -11.191   6.007 55.41  0.00           C
ATOM    731  C   ARG A 183      -7.372 -11.579   4.893 55.41  0.00           C
ATOM    732  O   ARG A 183      -8.603 -11.628   5.107 55.41  0.00           O
ATOM    733  N   PHE A 184      -6.785 -11.851   3.718 42.08  0.00           N
ATOM    734  CA  PHE A 184      -7.550 -12.410   2.590 42.08  0.00           C
ATOM    735  C   PHE A 184      -7.017 -13.778   2.185 42.08  0.00           C
ATOM    736  O   PHE A 184      -5.814 -14.039   2.233 42.08  0.00           O
ATOM    737  N   ASN A 185      -7.926 -14.630   1.714 36.30  0.00           N
ATOM    738  CA  ASN A 185      -7.604 -16.007   1.378 36.30  0.00           C
ATOM    739  C   ASN A 185      -6.563 -16.229   0.291 36.30  0.00           C
ATOM    740  O   ASN A 185      -5.857 -17.232   0.316 36.30  0.00           O
ATOM    741  N   TRP A 186      -6.507 -15.349  -0.690 34.90  0.00           N
ATOM    742  CA  TRP A 186      -5.602 -15.511  -1.821 34.90  0.00           C
ATOM    743  C   TRP A 186      -4.124 -15.190  -1.442 34.90  0.00           C
ATOM    744  O   TRP A 186      -3.168 -15.441  -2.207 34.90  0.00           O
```

```
ATOM    745  N   GLU A 187      -3.932 -14.615  -0.259 46.05  0.00           N
ATOM    746  CA  GLU A 187      -2.575 -14.179   0.176 46.05  0.00           C
ATOM    747  C   GLU A 187      -1.511 -15.229   0.225 46.05  0.00           C
ATOM    748  O   GLU A 187      -0.428 -15.028  -0.334 46.05  0.00           O
ATOM    749  N   CYS A 188      -1.801 -16.352   0.884 53.49  0.00           N
ATOM    750  CA  CYS A 188      -0.760 -17.344   1.180 53.49  0.00           C
ATOM    751  C   CYS A 188      -0.146 -17.865  -0.127 53.49  0.00           C
ATOM    752  O   CYS A 188       1.067 -18.120  -0.218 53.49  0.00           O
ATOM    753  N   LYS A 189      -0.970 -17.963  -1.166 40.03  0.00           N
ATOM    754  CA  LYS A 189      -0.511 -18.522  -2.447 40.03  0.00           C
ATOM    755  C   LYS A 189       0.696 -17.731  -3.018 40.03  0.00           C
ATOM    756  O   LYS A 189       1.569 -18.272  -3.724 40.03  0.00           O
ATOM    757  N   TYR A 190       0.747 -16.439  -2.708 37.23  0.00           N
ATOM    758  CA  TYR A 190       1.763 -15.611  -3.285 37.23  0.00           C
ATOM    759  C   TYR A 190       2.819 -15.235  -2.248 37.23  0.00           C
ATOM    760  O   TYR A 190       3.823 -14.654  -2.584 37.23  0.00           O
ATOM    761  N   CYS A 191       2.587 -15.625  -0.997 43.81  0.00           N
ATOM    762  CA  CYS A 191       3.353 -15.193   0.160 43.81  0.00           C
ATOM    763  C   CYS A 191       4.692 -15.934   0.269 43.81  0.00           C
ATOM    764  O   CYS A 191       4.732 -17.173   0.348 43.81  0.00           O
ATOM    765  N   ILE A 192       5.784 -15.171   0.315 41.27  0.00           N
ATOM    766  CA  ILE A 192       7.106 -15.776   0.279 41.27  0.00           C
ATOM    767  C   ILE A 192       7.409 -16.403   1.615 41.27  0.00           C
ATOM    768  O   ILE A 192       8.394 -17.068   1.711 41.27  0.00           O
ATOM    769  N   PHE A 193       6.578 -16.191   2.632 42.93  0.00           N
ATOM    770  CA  PHE A 193       6.786 -16.794   3.943 42.93  0.00           C
ATOM    771  C   PHE A 193       5.984 -18.074   4.210 42.93  0.00           C
ATOM    772  O   PHE A 193       5.990 -18.637   5.317 42.93  0.00           O
ATOM    773  N   SER A 194       5.262 -18.513   3.198 34.28  0.00           N
ATOM    774  CA  SER A 194       4.413 -19.711   3.328 34.28  0.00           C
ATOM    775  C   SER A 194       5.249 -20.909   3.657 34.28  0.00           C
ATOM    776  O   SER A 194       4.748 -21.746   4.298 34.28  0.00           O
ATOM    777  N   VAL A 195       6.503 -20.952   3.199 39.35  0.00           N
ATOM    778  CA  VAL A 195       7.410 -21.989   3.516 39.35  0.00           C
ATOM    779  C   VAL A 195       7.733 -22.174   4.979 39.35  0.00           C
ATOM    780  O   VAL A 195       8.309 -23.201   5.323 39.35  0.00           O
ATOM    781  N   ILE A 196       7.419 -21.212   5.849 37.38  0.00           N
ATOM    782  CA  ILE A 196       7.718 -21.354   7.295 37.38  0.00           C
ATOM    783  C   ILE A 196       6.564 -20.975   8.148 37.38  0.00           C
ATOM    784  O   ILE A 196       6.683 -20.972   9.331 37.38  0.00           O
ATOM    785  N   CYS A 197       5.424 -20.671   7.561 52.86  0.00           N
ATOM    786  CA  CYS A 197       4.335 -20.157   8.329 52.86  0.00           C
ATOM    787  C   CYS A 197       3.331 -21.219   8.615 52.86  0.00           C
ATOM    788  O   CYS A 197       2.836 -21.849   7.697 52.86  0.00           O
ATOM    789  N   PRO A 198       2.960 -21.375   9.870 49.63  0.00           N
ATOM    790  CA  PRO A 198       1.970 -22.338  10.280 49.63  0.00           C
ATOM    791  C   PRO A 198       0.542 -22.046   9.971 49.63  0.00           C
ATOM    792  O   PRO A 198      -0.290 -22.938  10.156 49.63  0.00           O
ATOM    793  N   ALA A 199       0.227 -20.829   9.562 49.32  0.00           N
ATOM    794  CA  ALA A 199      -1.167 -20.431   9.353 49.32  0.00           C
ATOM    795  C   ALA A 199      -1.570 -20.467   7.877 49.32  0.00           C
ATOM    796  O   ALA A 199      -2.731 -20.267   7.563 49.32  0.00           O
ATOM    797  N   LYS A 200      -0.591 -20.655   6.991 64.63  0.00           N
ATOM    798  CA  LYS A 200      -0.778 -20.823   5.548 64.63  0.00           C
ATOM    799  C   LYS A 200      -1.946 -21.672   5.236 64.63  0.00           C
ATOM    800  O   LYS A 200      -2.051 -22.735   5.769 64.63  0.00           O
ATOM    801  N   LEU A 201      -2.799 -21.218   4.328 49.70  0.00           N
ATOM    802  CA  LEU A 201      -3.935 -22.003   3.876 49.70  0.00           C
ATOM    803  C   LEU A 201      -3.511 -23.046   2.859 49.70  0.00           C
ATOM    804  O   LEU A 201      -2.649 -22.775   2.009 49.70  0.00           O
ATOM    805  N   THR A 202      -4.127 -24.225   2.953 50.36  0.00           N
ATOM    806  CA  THR A 202      -3.796 -25.369   2.082 50.36  0.00           C
ATOM    807  C   THR A 202      -5.038 -25.920   1.370 50.36  0.00           C
ATOM    808  O   THR A 202      -6.101 -25.964   1.999 50.36  0.00           O
TER     809      THR A 202
ENDMDL
END
REMARK   1 ***********************************************************************
REMARK   1 Start File NAT_vs_DEC2_dSi_colored.pdb
REMARK   1 The occupancy record was modified to represent dSi := Si(DEC)-Si(NAT).
REMARK   1 Occ=0.00 means dSi=-1.461482 kB; Occ=99.99 means dSi=1.491450 kB.
MODEL        0
ATOM      1  N   MSE A   1      23.268  -4.104 -18.521 37.22  0.00           N
ATOM      2  CA  MSE A   1      23.439  -2.722 -18.016 37.22  0.00           C
ATOM      3  C   MSE A   1      22.152  -2.161 -17.383 37.22  0.00           C
ATOM      4  O   MSE A   1      22.147  -1.650 -16.252 37.22  0.00           O
ATOM      5  N   ILE A   2      21.045  -2.254 -18.100 27.40  0.00           N
ATOM      6  CA  ILE A   2      19.816  -1.632 -17.608 27.40  0.00           C
ATOM      7  C   ILE A   2      19.269  -2.405 -16.412 27.40  0.00           C
ATOM      8  O   ILE A   2      18.935  -1.835 -15.391 27.40  0.00           O
ATOM      9  N   THR A   3      19.186  -3.703 -16.563 41.96  0.00           N
ATOM     10  CA  THR A   3      18.718  -4.606 -15.498 41.96  0.00           C
ATOM     11  C   THR A   3      19.478  -4.398 -14.212 41.96  0.00           C
ATOM     12  O   THR A   3      18.884  -4.188 -13.150 41.96  0.00           O
ATOM     13  N   GLU A   4      20.800  -4.447 -14.318 43.40  0.00           N
ATOM     14  CA  GLU A   4      21.687  -4.148 -13.195 43.40  0.00           C
ATOM     15  C   GLU A   4      21.342  -2.832 -12.484 43.40  0.00           C
ATOM     16  O   GLU A   4      21.232  -2.791 -11.242 43.40  0.00           O
ATOM     17  N   PHE A   5      21.133  -1.765 -13.247 31.36  0.00           N
ATOM     18  CA  PHE A   5      20.826  -0.486 -12.602 31.36  0.00           C
ATOM     19  C   PHE A   5      19.486  -0.520 -11.886 31.36  0.00           C
ATOM     20  O   PHE A   5      19.415  -0.122 -10.736 31.36  0.00           O
```

ATOM	21	N	LEU	A	6	18.429	-1.039	-12.529	28.08	0.00	N
ATOM	22	CA	LEU	A	6	17.100	-1.072	-11.873	28.08	0.00	C
ATOM	23	C	LEU	A	6	17.119	-1.921	-10.586	28.08	0.00	C
ATOM	24	O	LEU	A	6	16.551	-1.545	-9.531	28.08	0.00	O
ATOM	25	N	LEU	A	7	17.767	-3.071	-10.672	47.44	0.00	N
ATOM	26	CA	LEU	A	7	17.843	-3.929	-9.486	47.44	0.00	C
ATOM	27	C	LEU	A	7	18.603	-3.241	-8.341	47.44	0.00	C
ATOM	28	O	LEU	A	7	18.190	-3.292	-7.184	47.44	0.00	O
ATOM	29	N	LYS	A	8	19.691	-2.566	-8.693	29.50	0.00	N
ATOM	30	CA	LYS	A	8	20.463	-1.856	-7.680	29.50	0.00	C
ATOM	31	C	LYS	A	8	19.628	-0.762	-7.044	29.50	0.00	C
ATOM	32	O	LYS	A	8	19.527	-0.688	-5.819	29.50	0.00	O
ATOM	33	N	LYS	A	9	18.947	0.036	-7.860	0.00	0.00	N
ATOM	34	CA	LYS	A	9	18.144	1.113	-7.292	0.00	0.00	C
ATOM	35	C	LYS	A	9	17.009	0.545	-6.471	0.00	0.00	C
ATOM	36	O	LYS	A	9	16.708	1.086	-5.402	0.00	0.00	O
ATOM	37	N	LYS	A	10	16.379	-0.547	-6.912	56.51	0.00	N
ATOM	38	CA	LYS	A	10	15.262	-1.072	-6.116	56.51	0.00	C
ATOM	39	C	LYS	A	10	15.660	-1.681	-4.726	56.51	0.00	C
ATOM	40	O	LYS	A	10	14.931	-1.547	-3.695	56.51	0.00	O
ATOM	41	N	LEU	A	11	16.798	-2.366	-4.699	56.08	0.00	N
ATOM	42	CA	LEU	A	11	17.328	-2.879	-3.411	56.08	0.00	C
ATOM	43	C	LEU	A	11	17.613	-1.686	-2.439	56.08	0.00	C
ATOM	44	O	LEU	A	11	17.179	-1.698	-1.270	56.08	0.00	O
ATOM	45	N	GLU	A	12	18.299	-0.655	-2.945	40.56	0.00	N
ATOM	46	CA	GLU	A	12	18.475	0.605	-2.181	40.56	0.00	C
ATOM	47	C	GLU	A	12	17.179	1.213	-1.616	40.56	0.00	C
ATOM	48	O	GLU	A	12	17.048	1.390	-0.426	40.56	0.00	O
ATOM	49	N	GLU	A	13	16.225	1.536	-2.461	43.09	0.00	N
ATOM	50	CA	GLU	A	13	14.901	1.950	-1.974	43.09	0.00	C
ATOM	51	C	GLU	A	13	14.358	1.037	-0.868	43.09	0.00	C
ATOM	52	O	GLU	A	13	13.966	1.505	0.190	43.09	0.00	O
ATOM	53	N	HIS	A	14	14.335	-0.267	-1.101	62.74	0.00	N
ATOM	54	CA	HIS	A	14	13.794	-1.199	-0.106	62.74	0.00	C
ATOM	55	C	HIS	A	14	14.560	-1.126	1.236	62.74	0.00	C
ATOM	56	O	HIS	A	14	13.970	-1.122	2.322	62.74	0.00	O
ATOM	57	N	LEU	A	15	15.884	-1.079	1.145	54.44	0.00	N
ATOM	58	CA	LEU	A	15	16.750	-1.011	2.322	54.44	0.00	C
ATOM	59	C	LEU	A	15	16.724	0.377	2.988	54.44	0.00	C
ATOM	60	O	LEU	A	15	17.114	0.492	4.140	54.44	0.00	O
ATOM	61	N	SER	A	16	16.283	1.421	2.277	50.75	0.00	N
ATOM	62	CA	SER	A	16	16.215	2.761	2.870	50.75	0.00	C
ATOM	63	C	SER	A	16	15.126	2.866	3.937	50.75	0.00	C
ATOM	64	O	SER	A	16	15.184	3.766	4.746	50.75	0.00	O
ATOM	65	N	HIS	A	17	14.126	1.987	3.905	47.33	0.00	N
ATOM	66	CA	HIS	A	17	12.931	2.175	4.710	47.33	0.00	C
ATOM	67	C	HIS	A	17	12.911	1.290	5.925	47.33	0.00	C
ATOM	68	O	HIS	A	17	12.211	0.285	5.939	47.33	0.00	O
ATOM	69	N	VAL	A	18	13.667	1.662	6.952	46.62	0.00	N
ATOM	70	CA	VAL	A	18	13.649	0.930	8.226	46.62	0.00	C
ATOM	71	C	VAL	A	18	12.455	1.416	9.073	46.62	0.00	C
ATOM	72	O	VAL	A	18	12.231	2.619	9.198	46.62	0.00	O
ATOM	73	N	LYS	A	19	11.711	0.480	9.654	50.02	0.00	N
ATOM	74	CA	LYS	A	19	10.394	0.738	10.251	50.02	0.00	C
ATOM	75	C	LYS	A	19	10.466	0.797	11.779	50.02	0.00	C
ATOM	76	O	LYS	A	19	11.024	-0.102	12.394	50.02	0.00	O
ATOM	77	N	GLU	A	20	9.892	1.832	12.391	23.75	0.00	N
ATOM	78	CA	GLU	A	20	10.003	2.007	13.848	23.75	0.00	C
ATOM	79	C	GLU	A	20	8.986	1.178	14.597	23.75	0.00	C
ATOM	80	O	GLU	A	20	7.952	0.808	14.049	23.75	0.00	O
ATOM	81	N	GLU	A	21	9.300	0.899	15.861	36.12	0.00	N
ATOM	82	CA	GLU	A	21	8.634	-0.168	16.607	36.12	0.00	C
ATOM	83	C	GLU	A	21	7.153	0.124	16.844	36.12	0.00	C
ATOM	84	O	GLU	A	21	6.369	-0.811	17.040	36.12	0.00	O
ATOM	85	N	ASN	A	22	6.774	1.399	16.808	44.32	0.00	N
ATOM	86	CA	ASN	A	22	5.371	1.790	16.970	44.32	0.00	C
ATOM	87	C	ASN	A	22	4.716	2.391	15.715	44.32	0.00	C
ATOM	88	O	ASN	A	22	3.648	3.000	15.824	44.32	0.00	O
ATOM	89	N	THR	A	23	5.339	2.269	14.540	60.13	0.00	N
ATOM	90	CA	THR	A	23	4.636	2.674	13.297	60.13	0.00	C
ATOM	91	C	THR	A	23	3.937	1.492	12.597	60.13	0.00	C
ATOM	92	O	THR	A	23	4.343	0.315	12.707	60.13	0.00	O
ATOM	93	N	ILE	A	24	2.863	1.841	11.907	61.59	0.00	N
ATOM	94	CA	ILE	A	24	2.133	0.908	11.089	61.59	0.00	C
ATOM	95	C	ILE	A	24	1.893	1.588	9.781	61.59	0.00	C
ATOM	96	O	ILE	A	24	1.270	2.646	9.760	61.59	0.00	O
ATOM	97	N	TYR	A	25	2.393	1.015	8.691	56.15	0.00	N
ATOM	98	CA	TYR	A	25	2.072	1.558	7.393	56.15	0.00	C
ATOM	99	C	TYR	A	25	0.609	1.246	7.051	56.15	0.00	C
ATOM	100	O	TYR	A	25	0.029	0.273	7.510	56.15	0.00	O
ATOM	101	N	VAL	A	26	0.019	2.088	6.230	70.44	0.00	N
ATOM	102	CA	VAL	A	26	-1.326	1.856	5.716	70.44	0.00	C
ATOM	103	C	VAL	A	26	-1.394	0.504	4.989	70.44	0.00	C
ATOM	104	O	VAL	A	26	-2.320	-0.244	5.138	70.44	0.00	O
ATOM	105	N	THR	A	27	-0.413	0.264	4.153	57.80	0.00	N
ATOM	106	CA	THR	A	27	-0.225	-0.940	3.432	57.80	0.00	C
ATOM	107	C	THR	A	27	-0.214	-2.155	4.325	57.80	0.00	C
ATOM	108	O	THR	A	27	-0.816	-3.189	4.005	57.80	0.00	O
ATOM	109	N	ASP	A	28	0.421	-2.066	5.476	52.14	0.00	N
ATOM	110	CA	ASP	A	28	0.217	-3.125	6.447	52.14	0.00	C
ATOM	111	C	ASP	A	28	-1.221	-3.366	6.899	52.14	0.00	C
ATOM	112	O	ASP	A	28	-1.560	-4.494	7.172	52.14	0.00	O

```
ATOM    113  N   LEU A  29      -2.082  -2.355   6.958 66.81  0.00           N
ATOM    114  CA  LEU A  29      -3.418  -2.565   7.521 66.81  0.00           C
ATOM    115  C   LEU A  29      -4.304  -3.447   6.637 66.81  0.00           C
ATOM    116  O   LEU A  29      -5.335  -3.962   7.065 66.81  0.00           O
ATOM    117  N   VAL A  30      -3.930  -3.597   5.378 55.91  0.00           N
ATOM    118  CA  VAL A  30      -4.776  -4.325   4.449 55.91  0.00           C
ATOM    119  C   VAL A  30      -4.099  -5.619   4.014 55.91  0.00           C
ATOM    120  O   VAL A  30      -4.413  -6.174   2.971 55.91  0.00           O
ATOM    121  N   ARG A  31      -3.154  -6.069   4.828 86.64  0.00           N
ATOM    122  CA  ARG A  31      -2.426  -7.287   4.613 86.64  0.00           C
ATOM    123  C   ARG A  31      -2.433  -8.174   5.834 86.64  0.00           C
ATOM    124  O   ARG A  31      -2.927  -7.806   6.907 86.64  0.00           O
ATOM    125  N   CYS A  32      -1.955  -9.395   5.616 50.59  0.00           N
ATOM    126  CA  CYS A  32      -1.910 -10.426   6.637 50.59  0.00           C
ATOM    127  C   CYS A  32      -1.015  -9.951   7.770 50.59  0.00           C
ATOM    128  O   CYS A  32       0.134  -9.567   7.534 50.59  0.00           O
ATOM    129  N   PRO A  33      -1.508 -10.024   8.999 50.11  0.00           N
ATOM    130  CA  PRO A  33      -0.664  -9.610  10.083 50.11  0.00           C
ATOM    131  C   PRO A  33       0.611 -10.429  10.223 50.11  0.00           C
ATOM    132  O   PRO A  33       1.694  -9.852  10.411 50.11  0.00           O
ATOM    133  N   ARG A  34       0.512 -11.757  10.119 34.08  0.00           N
ATOM    134  CA  ARG A  34       1.689 -12.574  10.256 34.08  0.00           C
ATOM    135  C   ARG A  34       2.720 -12.198   9.218 34.08  0.00           C
ATOM    136  O   ARG A  34       3.911 -12.267   9.453 34.08  0.00           O
ATOM    137  N   ARG A  35       2.276 -11.837   8.033 25.98  0.00           N
ATOM    138  CA  ARG A  35       3.238 -11.530   6.965 25.98  0.00           C
ATOM    139  C   ARG A  35       3.971 -10.221   7.251 25.98  0.00           C
ATOM    140  O   ARG A  35       5.151 -10.042   6.901 25.98  0.00           O
ATOM    141  N   VAL A  36       3.228  -9.297   7.848 52.80  0.00           N
ATOM    142  CA  VAL A  36       3.791  -7.981   8.175 52.80  0.00           C
ATOM    143  C   VAL A  36       4.864  -8.200   9.230 52.80  0.00           C
ATOM    144  O   VAL A  36       5.972  -7.734   9.085 52.80  0.00           O
ATOM    145  N   ARG A  37       4.561  -8.972  10.259 54.12  0.00           N
ATOM    146  CA  ARG A  37       5.600  -9.259  11.227 54.12  0.00           C
ATOM    147  C   ARG A  37       6.749 -10.045  10.630 54.12  0.00           C
ATOM    148  O   ARG A  37       7.879  -9.890  11.061 54.12  0.00           O
ATOM    149  N   TYR A  38       6.481 -10.904   9.649 45.86  0.00           N
ATOM    150  CA  TYR A  38       7.570 -11.660   9.036 45.86  0.00           C
ATOM    151  C   TYR A  38       8.499 -10.724   8.363 45.86  0.00           C
ATOM    152  O   TYR A  38       9.689 -10.942   8.377 45.86  0.00           O
ATOM    153  N   GLU A  39       7.963  -9.678   7.760 57.04  0.00           N
ATOM    154  CA  GLU A  39       8.816  -8.667   7.126 57.04  0.00           C
ATOM    155  C   GLU A  39       9.769  -7.904   8.077 57.04  0.00           C
ATOM    156  O   GLU A  39      10.773  -7.394   7.597 57.04  0.00           O
ATOM    157  N   SER A  40       9.464  -7.844   9.380 46.13  0.00           N
ATOM    158  CA  SER A  40      10.398  -7.338  10.412 46.13  0.00           C
ATOM    159  C   SER A  40      11.345  -8.393  10.958 46.13  0.00           C
ATOM    160  O   SER A  40      12.540  -8.130  11.114 46.13  0.00           O
ATOM    161  N   GLU A  41      10.808  -9.578  11.263 51.09  0.00           N
ATOM    162  CA  GLU A  41      11.604 -10.637  11.865 51.09  0.00           C
ATOM    163  C   GLU A  41      12.456 -11.415  10.889 51.09  0.00           C
ATOM    164  O   GLU A  41      13.321 -12.135  11.370 51.09  0.00           O
ATOM    165  N   TYR A  42      12.238 -11.336   9.566 43.68  0.00           N
ATOM    166  CA  TYR A  42      13.052 -12.135   8.586 43.68  0.00           C
ATOM    167  C   TYR A  42      13.369 -11.290   7.378 43.68  0.00           C
ATOM    168  O   TYR A  42      13.024 -11.599   6.196 43.68  0.00           O
ATOM    169  N   LYS A  43      14.059 -10.210   7.691 60.71  0.00           N
ATOM    170  CA  LYS A  43      14.286  -9.169   6.756 60.71  0.00           C
ATOM    171  C   LYS A  43      15.175  -9.664   5.651 60.71  0.00           C
ATOM    172  O   LYS A  43      15.118  -9.127   4.534 60.71  0.00           O
ATOM    173  N   GLU A  44      16.024 -10.635   5.936 55.81  0.00           N
ATOM    174  CA  GLU A  44      16.906 -11.087   4.887 55.81  0.00           C
ATOM    175  C   GLU A  44      16.077 -11.799   3.791 55.81  0.00           C
ATOM    176  O   GLU A  44      16.309 -11.615   2.572 55.81  0.00           O
ATOM    177  N   LEU A  45      15.070 -12.551   4.234 48.24  0.00           N
ATOM    178  CA  LEU A  45      14.177 -13.244   3.311 48.24  0.00           C
ATOM    179  C   LEU A  45      13.268 -12.185   2.642 48.24  0.00           C
ATOM    180  O   LEU A  45      13.095 -12.177   1.406 48.24  0.00           O
ATOM    181  N   ALA A  46      12.710 -11.278   3.448 62.46  0.00           N
ATOM    182  CA  ALA A  46      11.893 -10.178   2.897 62.46  0.00           C
ATOM    183  C   ALA A  46      12.519  -9.534   1.649 62.46  0.00           C
ATOM    184  O   ALA A  46      11.825  -9.262   0.709 62.46  0.00           O
ATOM    185  N   ILE A  47      13.830  -9.341   1.634 56.61  0.00           N
ATOM    186  CA  ILE A  47      14.526  -8.691   0.538 56.61  0.00           C
ATOM    187  C   ILE A  47      14.299  -9.332  -0.838 56.61  0.00           C
ATOM    188  O   ILE A  47      14.451  -8.669  -1.895 56.61  0.00           O
ATOM    189  N   SER A  48      13.959 -10.620  -0.874 57.74  0.00           N
ATOM    190  CA  SER A  48      13.715 -11.277  -2.169 57.74  0.00           C
ATOM    191  C   SER A  48      12.509 -10.666  -2.882 57.74  0.00           C
ATOM    192  O   SER A  48      12.401 -10.723  -4.108 57.74  0.00           O
ATOM    193  N   GLN A  49      11.610 -10.068  -2.123 65.98  0.00           N
ATOM    194  CA  GLN A  49      10.459  -9.434  -2.743 65.98  0.00           C
ATOM    195  C   GLN A  49      10.847  -8.226  -3.623 65.98  0.00           C
ATOM    196  O   GLN A  49      10.116  -7.889  -4.482 65.98  0.00           O
ATOM    197  N   VAL A  50      12.031  -7.643  -3.424 68.48  0.00           N
ATOM    198  CA  VAL A  50      12.659  -6.700  -4.358 68.48  0.00           C
ATOM    199  C   VAL A  50      12.750  -7.176  -5.802 68.48  0.00           C
ATOM    200  O   VAL A  50      12.805  -6.373  -6.754 68.48  0.00           O
ATOM    201  N   TYR A  51      12.865  -8.468  -5.991 34.17  0.00           N
ATOM    202  CA  TYR A  51      13.231  -8.953  -7.306 34.17  0.00           C
ATOM    203  C   TYR A  51      12.000  -9.515  -8.013 34.17  0.00           C
ATOM    204  O   TYR A  51      12.116 -10.179  -9.031 34.17  0.00           O
```

ATOM	205	N	ALA	A	52	10.823	-9.258	-7.445	46.56	0.00	N
ATOM	206	CA	ALA	A	52	9.580	-9.586	-8.075	46.56	0.00	C
ATOM	207	C	ALA	A	52	9.375	-8.694	-9.336	46.56	0.00	C
ATOM	208	O	ALA	A	52	9.152	-7.478	-9.216	46.56	0.00	O
ATOM	209	N	PRO	A	53	9.401	-9.282	-10.541	49.85	0.00	N
ATOM	210	CA	PRO	A	53	9.338	-8.519	-11.804	49.85	0.00	C
ATOM	211	C	PRO	A	53	8.051	-7.715	-11.969	49.85	0.00	C
ATOM	212	O	PRO	A	53	8.107	-6.662	-12.589	49.85	0.00	O
ATOM	213	N	SER	A	54	6.912	-8.185	-11.428	47.01	0.00	N
ATOM	214	CA	SER	A	54	5.662	-7.382	-11.372	47.01	0.00	C
ATOM	215	C	SER	A	54	5.807	-6.143	-10.551	47.01	0.00	C
ATOM	216	O	SER	A	54	5.159	-5.116	-10.806	47.01	0.00	O
ATOM	217	N	ALA	A	55	6.666	-6.210	-9.534	43.60	0.00	N
ATOM	218	CA	ALA	A	55	6.871	-5.023	-8.707	43.60	0.00	C
ATOM	219	C	ALA	A	55	7.756	-4.055	-9.490	43.60	0.00	C
ATOM	220	O	ALA	A	55	7.598	-2.860	-9.403	43.60	0.00	O
ATOM	221	N	ILE	A	56	8.662	-4.543	-10.314	26.91	0.00	N
ATOM	222	CA	ILE	A	56	9.562	-3.610	-11.005	26.91	0.00	C
ATOM	223	C	ILE	A	56	8.724	-2.964	-12.115	26.91	0.00	C
ATOM	224	O	ILE	A	56	8.790	-1.755	-12.348	26.91	0.00	O
ATOM	225	N	LEU	A	57	7.912	-3.805	-12.760	22.36	0.00	N
ATOM	226	CA	LEU	A	57	6.935	-3.366	-13.747	22.36	0.00	C
ATOM	227	C	LEU	A	57	6.033	-2.336	-13.087	22.36	0.00	C
ATOM	228	O	LEU	A	57	5.874	-1.207	-13.590	22.36	0.00	O
ATOM	229	N	GLY	A	58	5.483	-2.701	-11.946	47.90	0.00	N
ATOM	230	CA	GLY	A	58	4.634	-1.806	-11.182	47.90	0.00	C
ATOM	231	C	GLY	A	58	5.258	-0.473	-10.875	47.90	0.00	C
ATOM	232	O	GLY	A	58	4.607	0.560	-11.036	47.90	0.00	O
ATOM	233	N	ASP	A	59	6.514	-0.439	-10.443	47.45	0.00	N
ATOM	234	CA	ASP	A	59	7.150	0.844	-10.105	47.45	0.00	C
ATOM	235	C	ASP	A	59	7.486	1.775	-11.275	47.45	0.00	C
ATOM	236	O	ASP	A	59	7.461	2.943	-11.121	47.45	0.00	O
ATOM	237	N	ILE	A	60	7.874	1.212	-12.405	21.25	0.00	N
ATOM	238	CA	ILE	A	60	8.072	1.923	-13.651	21.25	0.00	C
ATOM	239	C	ILE	A	60	6.774	2.586	-14.133	21.25	0.00	C
ATOM	240	O	ILE	A	60	6.798	3.761	-14.559	21.25	0.00	O
ATOM	241	N	LEU	A	61	5.641	1.870	-14.058	25.12	0.00	N
ATOM	242	CA	LEU	A	61	4.334	2.454	-14.392	25.12	0.00	C
ATOM	243	C	LEU	A	61	4.024	3.653	-13.537	25.12	0.00	C
ATOM	244	O	LEU	A	61	3.668	4.678	-14.050	25.12	0.00	O
ATOM	245	N	HIS	A	62	4.172	3.508	-12.232	66.36	0.00	N
ATOM	246	CA	HIS	A	62	3.968	4.616	-11.306	66.36	0.00	C
ATOM	247	C	HIS	A	62	4.884	5.714	-11.684	66.36	0.00	C
ATOM	248	O	HIS	A	62	4.443	6.854	-11.841	66.36	0.00	O
ATOM	249	N	LEU	A	63	6.158	5.423	-11.888	23.84	0.00	N
ATOM	250	CA	LEU	A	63	7.034	6.526	-12.325	23.84	0.00	C
ATOM	251	C	LEU	A	63	6.405	7.217	-13.537	23.84	0.00	C
ATOM	252	O	LEU	A	63	6.167	8.394	-13.497	23.84	0.00	O
ATOM	253	N	GLY	A	64	6.109	6.499	-14.603	27.85	0.00	N
ATOM	254	CA	GLY	A	64	5.719	7.158	-15.811	27.85	0.00	C
ATOM	255	C	GLY	A	64	4.400	7.890	-15.740	27.85	0.00	C
ATOM	256	O	GLY	A	64	4.174	8.755	-16.509	27.85	0.00	O
ATOM	257	N	LEU	A	65	3.499	7.454	-14.886	31.75	0.00	N
ATOM	258	CA	LEU	A	65	2.129	7.928	-14.922	31.75	0.00	C
ATOM	259	C	LEU	A	65	2.136	9.075	-13.968	31.75	0.00	C
ATOM	260	O	LEU	A	65	1.699	10.153	-14.266	31.75	0.00	O
ATOM	261	N	GLU	A	66	2.672	8.845	-12.789	78.61	0.00	N
ATOM	262	CA	GLU	A	66	3.061	9.970	-11.943	78.61	0.00	C
ATOM	263	C	GLU	A	66	3.702	11.154	-12.688	78.61	0.00	C
ATOM	264	O	GLU	A	66	3.297	12.272	-12.436	78.61	0.00	O
ATOM	265	N	SER	A	67	4.665	10.981	-13.581	40.41	0.00	N
ATOM	266	CA	SER	A	67	5.183	12.221	-14.250	40.41	0.00	C
ATOM	267	C	SER	A	67	4.100	13.012	-15.067	40.41	0.00	C
ATOM	268	O	SER	A	67	4.182	14.248	-15.203	40.41	0.00	O
ATOM	269	N	VAL	A	68	3.091	12.293	-15.600	37.35	0.00	N
ATOM	270	CA	VAL	A	68	1.939	12.934	-16.238	37.35	0.00	C
ATOM	271	C	VAL	A	68	1.158	13.773	-15.231	37.35	0.00	C
ATOM	272	O	VAL	A	68	0.645	14.865	-15.520	37.35	0.00	O
ATOM	273	N	LEU	A	69	1.040	13.216	-14.047	35.55	0.00	N
ATOM	274	CA	LEU	A	69	0.261	13.818	-13.004	35.55	0.00	C
ATOM	275	C	LEU	A	69	0.848	15.118	-12.430	35.55	0.00	C
ATOM	276	O	LEU	A	69	0.134	15.992	-11.952	35.55	0.00	O
ATOM	277	N	LYS	A	70	2.165	15.215	-12.445	19.55	0.00	N
ATOM	278	CA	LYS	A	70	2.860	16.356	-11.911	19.55	0.00	C
ATOM	279	C	LYS	A	70	2.708	17.450	-12.934	19.55	0.00	C
ATOM	280	O	LYS	A	70	2.497	18.596	-12.562	19.55	0.00	O
ATOM	281	N	GLY	A	71	2.769	17.105	-14.229	30.56	0.00	N
ATOM	282	CA	GLY	A	71	2.550	18.111	-15.305	30.56	0.00	C
ATOM	283	C	GLY	A	71	1.130	18.685	-15.376	30.56	0.00	C
ATOM	284	O	GLY	A	71	0.821	19.815	-14.923	30.56	0.00	O
ATOM	285	N	ASN	A	72	0.217	17.876	-15.876	33.38	0.00	N
ATOM	286	CA	ASN	A	72	-1.095	18.370	-16.206	33.38	0.00	C
ATOM	287	C	ASN	A	72	-2.040	18.612	-15.074	33.38	0.00	C
ATOM	288	O	ASN	A	72	-2.921	19.420	-15.211	33.38	0.00	O
ATOM	289	N	PHE	A	73	-1.880	17.933	-13.966	52.13	0.00	N
ATOM	290	CA	PHE	A	73	-2.766	18.118	-12.847	52.13	0.00	C
ATOM	291	C	PHE	A	73	-2.091	18.771	-11.639	52.13	0.00	C
ATOM	292	O	PHE	A	73	-2.674	18.861	-10.593	52.13	0.00	O
ATOM	293	N	ASN	A	74	-0.838	19.166	-11.735	42.19	0.00	N
ATOM	294	CA	ASN	A	74	-0.201	19.781	-10.582	42.19	0.00	C
ATOM	295	C	ASN	A	74	-0.355	18.949	-9.279	42.19	0.00	C
ATOM	296	O	ASN	A	74	-0.590	19.474	-8.165	42.19	0.00	O

ATOM	297	N	ALA	A	75	-0.160	17.641	-9.414	49.67	0.00	N
ATOM	298	CA	ALA	A	75	-0.115	16.792	-8.245	49.67	0.00	C
ATOM	299	C	ALA	A	75	1.282	16.777	-7.622	49.67	0.00	C
ATOM	300	O	ALA	A	75	2.270	16.992	-8.289	49.67	0.00	O
ATOM	301	N	GLU	A	76	1.336	16.530	-6.332	45.22	0.00	N
ATOM	302	CA	GLU	A	76	2.559	16.069	-5.695	45.22	0.00	C
ATOM	303	C	GLU	A	76	2.544	14.546	-5.834	45.22	0.00	C
ATOM	304	O	GLU	A	76	1.466	13.957	-5.865	45.22	0.00	O
ATOM	305	N	THR	A	77	3.710	13.915	-5.909	63.11	0.00	N
ATOM	306	CA	THR	A	77	3.794	12.484	-6.087	63.11	0.00	C
ATOM	307	C	THR	A	77	4.639	11.898	-4.978	63.11	0.00	C
ATOM	308	O	THR	A	77	5.510	12.566	-4.500	63.11	0.00	O
ATOM	309	N	GLU	A	78	4.321	10.666	-4.562	62.28	0.00	N
ATOM	310	CA	GLU	A	78	4.988	9.920	-3.517	62.28	0.00	C
ATOM	311	C	GLU	A	78	5.020	10.703	-2.232	62.28	0.00	C
ATOM	312	O	GLU	A	78	6.063	10.936	-1.623	62.28	0.00	O
ATOM	313	N	VAL	A	79	3.826	11.074	-1.816	42.88	0.00	N
ATOM	314	CA	VAL	A	79	3.635	12.010	-0.767	42.88	0.00	C
ATOM	315	C	VAL	A	79	3.474	11.303	0.544	42.88	0.00	C
ATOM	316	O	VAL	A	79	2.510	10.573	0.774	42.88	0.00	O
ATOM	317	N	GLU	A	80	4.416	11.580	1.421	42.38	0.00	N
ATOM	318	CA	GLU	A	80	4.530	10.857	2.668	42.38	0.00	C
ATOM	319	C	GLU	A	80	3.901	11.655	3.780	42.38	0.00	C
ATOM	320	O	GLU	A	80	4.090	12.840	3.853	42.38	0.00	O
ATOM	321	N	THR	A	81	3.177	11.027	4.670	41.50	0.00	N
ATOM	322	CA	THR	A	81	2.510	11.744	5.761	41.50	0.00	C
ATOM	323	C	THR	A	81	2.133	10.811	6.884	41.50	0.00	C
ATOM	324	O	THR	A	81	2.164	9.597	6.754	41.50	0.00	O
ATOM	325	N	LEU	A	82	1.667	11.373	7.976	42.94	0.00	N
ATOM	326	CA	LEU	A	82	1.644	10.601	9.202	42.94	0.00	C
ATOM	327	C	LEU	A	82	0.492	11.060	10.035	42.94	0.00	C
ATOM	328	O	LEU	A	82	0.098	12.203	9.950	42.94	0.00	O
ATOM	329	N	ARG	A	83	-0.124	10.141	10.761	22.34	0.00	N
ATOM	330	CA	ARG	A	83	-1.326	10.449	11.518	22.34	0.00	C
ATOM	331	C	ARG	A	83	-1.368	9.467	12.659	22.34	0.00	C
ATOM	332	O	ARG	A	83	-0.926	8.327	12.512	22.34	0.00	O
ATOM	333	N	GLU	A	84	-1.873	9.890	13.809	13.69	0.00	N
ATOM	334	CA	GLU	A	84	-1.727	9.022	14.978	13.69	0.00	C
ATOM	335	C	GLU	A	84	-3.042	8.425	15.425	13.69	0.00	C
ATOM	336	O	GLU	A	84	-4.110	8.996	15.183	13.69	0.00	O
ATOM	337	N	ILE	A	85	-2.918	7.275	16.095	55.14	0.00	N
ATOM	338	CA	ILE	A	85	-4.029	6.451	16.449	55.14	0.00	C
ATOM	339	C	ILE	A	85	-3.781	5.841	17.813	55.14	0.00	C
ATOM	340	O	ILE	A	85	-2.643	5.507	18.123	55.14	0.00	O
ATOM	341	N	ASN	A	86	-4.840	5.701	18.619	49.91	0.00	N
ATOM	342	CA	ASN	A	86	-4.761	5.021	19.937	49.91	0.00	C
ATOM	343	C	ASN	A	86	-5.250	3.566	19.895	49.91	0.00	C
ATOM	344	O	ASN	A	86	-6.399	3.293	19.511	49.91	0.00	O
ATOM	345	N	VAL	A	87	-4.383	2.645	20.322	49.44	0.00	N
ATOM	346	CA	VAL	A	87	-4.703	1.227	20.308	49.44	0.00	C
ATOM	347	C	VAL	A	87	-4.355	0.547	21.630	49.44	0.00	C
ATOM	348	O	VAL	A	87	-3.230	0.067	21.819	49.44	0.00	O
ATOM	349	N	GLY	A	88	-5.321	0.484	22.542	48.77	0.00	N
ATOM	350	CA	GLY	A	88	-5.066	-0.115	23.865	48.77	0.00	C
ATOM	351	C	GLY	A	88	-4.192	0.803	24.708	48.77	0.00	C
ATOM	352	O	GLY	A	88	-3.216	0.373	25.351	48.77	0.00	O
ATOM	353	N	GLY	A	89	-4.538	2.089	24.663	49.03	0.00	N
ATOM	354	CA	GLY	A	89	-3.780	3.124	25.348	49.03	0.00	C
ATOM	355	C	GLY	A	89	-2.339	3.328	24.890	49.03	0.00	C
ATOM	356	O	GLY	A	89	-1.668	4.231	25.406	49.03	0.00	O
ATOM	357	N	LYS	A	90	-1.846	2.502	23.956	49.16	0.00	N
ATOM	358	CA	LYS	A	90	-0.547	2.733	23.318	49.16	0.00	C
ATOM	359	C	LYS	A	90	-0.830	3.651	22.138	49.16	0.00	C
ATOM	360	O	LYS	A	90	-1.881	3.541	21.497	49.16	0.00	O
ATOM	361	N	VAL	A	91	0.069	4.592	21.872	52.06	0.00	N
ATOM	362	CA	VAL	A	91	-0.106	5.503	20.744	52.06	0.00	C
ATOM	363	C	VAL	A	91	0.675	4.963	19.561	52.06	0.00	C
ATOM	364	O	VAL	A	91	1.817	4.523	19.727	52.06	0.00	O
ATOM	365	N	TYR	A	92	0.052	5.005	18.377	47.93	0.00	N
ATOM	366	CA	TYR	A	92	0.676	4.480	17.190	47.93	0.00	C
ATOM	367	C	TYR	A	92	0.645	5.490	16.087	47.93	0.00	C
ATOM	368	O	TYR	A	92	-0.329	6.191	15.893	47.93	0.00	O
ATOM	369	N	LYS	A	93	1.747	5.547	15.371	70.62	0.00	N
ATOM	370	CA	LYS	A	93	1.859	6.412	14.217	70.62	0.00	C
ATOM	371	C	LYS	A	93	1.524	5.603	12.955	70.62	0.00	C
ATOM	372	O	LYS	A	93	2.270	4.714	12.581	70.62	0.00	O
ATOM	373	N	ILE	A	94	0.406	5.927	12.320	47.02	0.00	N
ATOM	374	CA	ILE	A	94	0.084	5.372	11.038	47.02	0.00	C
ATOM	375	C	ILE	A	94	0.743	6.184	9.929	47.02	0.00	C
ATOM	376	O	ILE	A	94	0.556	7.385	9.807	47.02	0.00	O
ATOM	377	N	LYS	A	95	1.497	5.507	9.097	69.21	0.00	N
ATOM	378	CA	LYS	A	95	2.305	6.166	8.106	69.21	0.00	C
ATOM	379	C	LYS	A	95	1.950	5.702	6.699	69.21	0.00	C
ATOM	380	O	LYS	A	95	1.760	4.545	6.470	69.21	0.00	O
ATOM	381	N	GLY	A	96	1.853	6.613	5.743	48.31	0.00	N
ATOM	382	CA	GLY	A	96	1.572	6.242	4.383	48.31	0.00	C
ATOM	383	C	GLY	A	96	2.142	7.179	3.378	48.31	0.00	C
ATOM	384	O	GLY	A	96	2.537	8.280	3.703	48.31	0.00	O
ATOM	385	N	ARG	A	97	2.107	6.740	2.138	42.47	0.00	N
ATOM	386	CA	ARG	A	97	2.693	7.428	1.007	42.47	0.00	C
ATOM	387	C	ARG	A	97	1.769	7.206	-0.178	42.47	0.00	C
ATOM	388	O	ARG	A	97	1.672	6.084	-0.699	42.47	0.00	O

ATOM	389	N	ALA	A	98	1.104	8.281	-0.588	45.19	0.00	N
ATOM	390	CA	ALA	A	98	0.117	8.243	-1.592	45.19	0.00	C
ATOM	391	C	ALA	A	98	0.862	8.383	-2.906	45.19	0.00	C
ATOM	392	O	ALA	A	98	1.855	9.097	-3.010	45.19	0.00	O
ATOM	393	N	ASP	A	99	0.424	7.673	-3.912	66.23	0.00	N
ATOM	394	CA	ASP	A	99	1.076	7.754	-5.196	66.23	0.00	C
ATOM	395	C	ASP	A	99	0.974	9.188	-5.720	66.23	0.00	C
ATOM	396	O	ASP	A	99	1.922	9.707	-6.350	66.23	0.00	O
ATOM	397	N	ALA	A	100	-0.166	9.846	-5.513	53.59	0.00	N
ATOM	398	CA	ALA	A	100	-0.251	11.269	-5.953	53.59	0.00	C
ATOM	399	C	ALA	A	100	-1.290	11.992	-5.184	53.59	0.00	C
ATOM	400	O	ALA	A	100	-2.232	11.366	-4.731	53.59	0.00	O
ATOM	401	N	ILE	A	101	-1.104	13.303	-4.998	57.33	0.00	N
ATOM	402	CA	ILE	A	101	-2.137	14.137	-4.384	57.33	0.00	C
ATOM	403	C	ILE	A	101	-2.390	15.420	-5.140	57.33	0.00	C
ATOM	404	O	ILE	A	101	-1.467	16.143	-5.558	57.33	0.00	O
ATOM	405	N	ILE	A	102	-3.672	15.695	-5.329	55.03	0.00	N
ATOM	406	CA	ILE	A	102	-4.083	17.004	-5.796	55.03	0.00	C
ATOM	407	C	ILE	A	102	-4.628	17.765	-4.581	55.03	0.00	C
ATOM	408	O	ILE	A	102	-5.695	17.431	-4.073	55.03	0.00	O
ATOM	409	N	ARG	A	103	-3.845	18.751	-4.103	27.39	0.00	N
ATOM	410	CA	ARG	A	103	-4.150	19.504	-2.879	27.39	0.00	C
ATOM	411	C	ARG	A	103	-5.423	20.391	-2.949	27.39	0.00	C
ATOM	412	O	ARG	A	103	-6.199	20.482	-1.960	27.39	0.00	O
ATOM	413	N	ASN	A	104	-5.626	21.051	-4.095	50.54	0.00	N
ATOM	414	CA	ASN	A	104	-6.745	21.998	-4.259	50.54	0.00	C
ATOM	415	C	ASN	A	104	-7.230	21.994	-5.679	50.54	0.00	C
ATOM	416	O	ASN	A	104	-6.693	22.692	-6.538	50.54	0.00	O
ATOM	417	N	ASP	A	105	-8.253	21.174	-5.879	48.89	0.00	N
ATOM	418	CA	ASP	A	105	-9.039	21.134	-7.084	48.89	0.00	C
ATOM	419	C	ASP	A	105	-10.471	21.605	-6.714	48.89	0.00	C
ATOM	420	O	ASP	A	105	-11.262	20.843	-6.158	48.89	0.00	O
ATOM	421	N	ASN	A	106	-10.768	22.878	-6.986	47.26	0.00	N
ATOM	422	CA	ASN	A	106	-12.040	23.506	-6.575	47.26	0.00	C
ATOM	423	C	ASN	A	106	-12.422	23.321	-5.104	47.26	0.00	C
ATOM	424	O	ASN	A	106	-13.561	23.020	-4.774	47.26	0.00	O
ATOM	425	N	GLY	A	107	-11.470	23.524	-4.215	52.85	0.00	N
ATOM	426	CA	GLY	A	107	-11.717	23.337	-2.789	52.85	0.00	C
ATOM	427	C	GLY	A	107	-11.737	21.883	-2.335	52.85	0.00	C
ATOM	428	O	GLY	A	107	-12.404	21.544	-1.352	52.85	0.00	O
ATOM	429	N	LYS	A	108	-11.008	21.019	-3.043	29.53	0.00	N
ATOM	430	CA	LYS	A	108	-10.915	19.615	-2.668	29.53	0.00	C
ATOM	431	C	LYS	A	108	-9.483	19.049	-2.884	29.53	0.00	C
ATOM	432	O	LYS	A	108	-8.779	19.403	-3.839	29.53	0.00	O
ATOM	433	N	SER	A	109	-9.039	18.236	-1.933	43.88	0.00	N
ATOM	434	CA	SER	A	109	-7.862	17.387	-2.118	43.88	0.00	C
ATOM	435	C	SER	A	109	-8.308	16.067	-2.739	43.88	0.00	C
ATOM	436	O	SER	A	109	-9.315	15.517	-2.353	43.88	0.00	O
ATOM	437	N	ILE	A	110	-7.524	15.551	-3.650	43.16	0.00	N
ATOM	438	CA	ILE	A	110	-7.790	14.265	-4.209	43.16	0.00	C
ATOM	439	C	ILE	A	110	-6.588	13.343	-3.999	43.16	0.00	C
ATOM	440	O	ILE	A	110	-5.512	13.629	-4.510	43.16	0.00	O
ATOM	441	N	VAL	A	111	-6.752	12.243	-3.266	55.63	0.00	N
ATOM	442	CA	VAL	A	111	-5.679	11.220	-3.198	55.63	0.00	C
ATOM	443	C	VAL	A	111	-5.836	10.276	-4.388	55.63	0.00	C
ATOM	444	O	VAL	A	111	-6.932	9.764	-4.636	55.63	0.00	O
ATOM	445	N	ILE	A	112	-4.775	10.018	-5.128	58.72	0.00	N
ATOM	446	CA	ILE	A	112	-4.866	9.126	-6.282	58.72	0.00	C
ATOM	447	C	ILE	A	112	-4.010	7.894	-6.056	58.72	0.00	C
ATOM	448	O	ILE	A	112	-2.842	8.019	-5.770	58.72	0.00	O
ATOM	449	N	GLU	A	113	-4.547	6.709	-6.243	83.18	0.00	N
ATOM	450	CA	GLU	A	113	-3.780	5.455	-6.060	83.18	0.00	C
ATOM	451	C	GLU	A	113	-3.823	4.710	-7.388	83.18	0.00	C
ATOM	452	O	GLU	A	113	-4.882	4.569	-8.052	83.18	0.00	O
ATOM	453	N	ILE	A	114	-2.657	4.354	-7.861	52.42	0.00	N
ATOM	454	CA	ILE	A	114	-2.531	3.758	-9.150	52.42	0.00	C
ATOM	455	C	ILE	A	114	-2.210	2.295	-8.960	52.42	0.00	C
ATOM	456	O	ILE	A	114	-1.423	1.954	-8.099	52.42	0.00	O
ATOM	457	N	LYS	A	115	-2.787	1.433	-9.786	70.74	0.00	N
ATOM	458	CA	LYS	A	115	-2.621	0.003	-9.616	70.74	0.00	C
ATOM	459	C	LYS	A	115	-2.438	-0.643	-10.947	70.74	0.00	C
ATOM	460	O	LYS	A	115	-2.964	-0.176	-11.951	70.74	0.00	O
ATOM	461	N	THR	A	116	-1.684	-1.729	-10.958	42.92	0.00	N
ATOM	462	CA	THR	A	116	-1.468	-2.466	-12.194	42.92	0.00	C
ATOM	463	C	THR	A	116	-1.602	-3.921	-11.890	42.92	0.00	C
ATOM	464	O	THR	A	116	-1.369	-4.337	-10.804	42.92	0.00	O
ATOM	465	N	SER	A	117	-2.085	-4.706	-12.802	45.57	0.00	N
ATOM	466	CA	SER	A	117	-2.109	-6.154	-12.601	45.57	0.00	C
ATOM	467	C	SER	A	117	-2.180	-6.692	-13.946	45.57	0.00	C
ATOM	468	O	SER	A	117	-2.324	-5.911	-14.909	45.57	0.00	O
ATOM	469	N	ARG	A	118	-2.032	-8.006	-14.033	29.06	0.00	N
ATOM	470	CA	ARG	A	118	-2.068	-8.640	-15.363	29.06	0.00	C
ATOM	471	C	ARG	A	118	-3.471	-8.928	-15.862	29.06	0.00	C
ATOM	472	O	ARG	A	118	-3.648	-9.162	-17.069	29.06	0.00	O
ATOM	473	N	SER	A	119	-4.457	-8.893	-14.965	43.52	0.00	N
ATOM	474	CA	SER	A	119	-5.872	-9.062	-15.362	43.52	0.00	C
ATOM	475	C	SER	A	119	-6.800	-7.967	-14.770	43.52	0.00	C
ATOM	476	O	SER	A	119	-6.533	-7.439	-13.690	43.52	0.00	O
ATOM	477	N	ASP	A	120	-7.938	-7.766	-15.424	45.20	0.00	N
ATOM	478	CA	ASP	A	120	-8.922	-6.789	-15.023	45.20	0.00	C
ATOM	479	C	ASP	A	120	-10.062	-7.464	-14.339	45.20	0.00	C
ATOM	480	O	ASP	A	120	-11.180	-6.930	-14.286	45.20	0.00	O

```
ATOM    481  N   LYS A 121      -9.805  -8.622 -13.800 28.78  0.00           N
ATOM    482  CA  LYS A 121     -10.861  -9.343 -13.147 28.78  0.00           C
ATOM    483  C   LYS A 121     -11.305  -8.719 -11.850 28.78  0.00           C
ATOM    484  O   LYS A 121     -10.493  -8.415 -10.994 28.78  0.00           O
ATOM    485  N   GLY A 122     -12.595  -8.552 -11.661 50.28  0.00           N
ATOM    486  CA  GLY A 122     -13.099  -8.150 -10.341 50.28  0.00           C
ATOM    487  C   GLY A 122     -12.816  -6.695  -9.897 50.28  0.00           C
ATOM    488  O   GLY A 122     -13.062  -6.363  -8.702 50.28  0.00           O
ATOM    489  N   LEU A 123     -12.369  -5.819 -10.826 74.40  0.00           N
ATOM    490  CA  LEU A 123     -12.047  -4.418 -10.440 74.40  0.00           C
ATOM    491  C   LEU A 123     -13.261  -3.672  -9.972 74.40  0.00           C
ATOM    492  O   LEU A 123     -14.252  -3.694 -10.654 74.40  0.00           O
ATOM    493  N   PRO A 124     -13.153  -2.951  -8.838 58.93  0.00           N
ATOM    494  CA  PRO A 124     -11.936  -2.786  -8.048 58.93  0.00           C
ATOM    495  C   PRO A 124     -11.854  -3.776  -6.934 58.93  0.00           C
ATOM    496  O   PRO A 124     -12.861  -4.122  -6.314 58.93  0.00           O
ATOM    497  N   LEU A 125     -10.645  -4.184  -6.634 43.87  0.00           N
ATOM    498  CA  LEU A 125     -10.394  -5.171  -5.542 43.87  0.00           C
ATOM    499  C   LEU A 125     -10.497  -4.530  -4.149 43.87  0.00           C
ATOM    500  O   LEU A 125     -10.112  -3.350  -3.908 43.87  0.00           O
ATOM    501  N   ILE A 126     -11.048  -5.276  -3.221 58.05  0.00           N
ATOM    502  CA  ILE A 126     -11.528  -4.647  -1.996 58.05  0.00           C
ATOM    503  C   ILE A 126     -10.339  -4.126  -1.167 58.05  0.00           C
ATOM    504  O   ILE A 126     -10.462  -3.123  -0.513 58.05  0.00           O
ATOM    505  N   HIS A 127      -9.195  -4.779  -1.251 43.35  0.00           N
ATOM    506  CA  HIS A 127      -8.055  -4.383  -0.479 43.35  0.00           C
ATOM    507  C   HIS A 127      -7.440  -3.163  -1.103 43.35  0.00           C
ATOM    508  O   HIS A 127      -6.834  -2.382  -0.415 43.35  0.00           O
ATOM    509  N   HIS A 128      -7.592  -2.980  -2.400 52.41  0.00           N
ATOM    510  CA  HIS A 128      -7.176  -1.718  -3.018 52.41  0.00           C
ATOM    511  C   HIS A 128      -8.083  -0.558  -2.609 52.41  0.00           C
ATOM    512  O   HIS A 128      -7.606   0.554  -2.368 52.41  0.00           O
ATOM    513  N   LYS A 129      -9.365  -0.803  -2.526 88.91  0.00           N
ATOM    514  CA  LYS A 129     -10.266   0.264  -2.198 88.91  0.00           C
ATOM    515  C   LYS A 129     -10.028   0.593  -0.745 88.91  0.00           C
ATOM    516  O   LYS A 129      -9.930   1.747  -0.408 88.91  0.00           O
ATOM    517  N   MSE A 130      -9.911  -0.410   0.128 78.04  0.00           N
ATOM    518  CA  MSE A 130      -9.661  -0.122   1.564 78.04  0.00           C
ATOM    519  C   MSE A 130      -8.432   0.772   1.739 78.04  0.00           C
ATOM    520  O   MSE A 130      -8.445   1.649   2.550 78.04  0.00           O
ATOM    521  N   GLN A 131      -7.394   0.520   0.958 58.11  0.00           N
ATOM    522  CA  GLN A 131      -6.133   1.211   1.097 58.11  0.00           C
ATOM    523  C   GLN A 131      -6.400   2.668   0.780 58.11  0.00           C
ATOM    524  O   GLN A 131      -5.918   3.542   1.460 58.11  0.00           O
ATOM    525  N   LEU A 132      -7.195   2.936  -0.242 72.67  0.00           N
ATOM    526  CA  LEU A 132      -7.436   4.290  -0.682 72.67  0.00           C
ATOM    527  C   LEU A 132      -8.295   4.984   0.347 72.67  0.00           C
ATOM    528  O   LEU A 132      -8.142   6.173   0.629 72.67  0.00           O
ATOM    529  N   GLN A 133      -9.173   4.244   0.963 99.99  0.00           N
ATOM    530  CA  GLN A 133     -10.034   4.835   1.951 99.99  0.00           C
ATOM    531  C   GLN A 133      -9.306   5.181   3.263 99.99  0.00           C
ATOM    532  O   GLN A 133      -9.739   6.050   4.021 99.99  0.00           O
ATOM    533  N   ILE A 134      -8.240   4.450   3.561 69.20  0.00           N
ATOM    534  CA  ILE A 134      -7.448   4.750   4.709 69.20  0.00           C
ATOM    535  C   ILE A 134      -6.635   6.008   4.375 69.20  0.00           C
ATOM    536  O   ILE A 134      -6.604   6.904   5.182 69.20  0.00           O
ATOM    537  N   TYR A 135      -5.993   6.089   3.200 53.52  0.00           N
ATOM    538  CA  TYR A 135      -5.308   7.299   2.832 53.52  0.00           C
ATOM    539  C   TYR A 135      -6.263   8.486   3.003 53.52  0.00           C
ATOM    540  O   TYR A 135      -5.876   9.536   3.592 53.52  0.00           O
ATOM    541  N   LEU A 136      -7.510   8.345   2.563 90.27  0.00           N
ATOM    542  CA  LEU A 136      -8.431   9.472   2.663 90.27  0.00           C
ATOM    543  C   LEU A 136      -8.471   9.988   4.123 90.27  0.00           C
ATOM    544  O   LEU A 136      -8.423  11.193   4.364 90.27  0.00           O
ATOM    545  N   TRP A 137      -8.562   9.085   5.079 77.33  0.00           N
ATOM    546  CA  TRP A 137      -8.471   9.465   6.480 77.33  0.00           C
ATOM    547  C   TRP A 137      -7.090  10.092   6.781 77.33  0.00           C
ATOM    548  O   TRP A 137      -6.988  11.182   7.363 77.33  0.00           O
ATOM    549  N   LEU A 138      -6.039   9.457   6.283 32.22  0.00           N
ATOM    550  CA  LEU A 138      -4.665   9.881   6.616 32.22  0.00           C
ATOM    551  C   LEU A 138      -4.353  11.332   6.246 32.22  0.00           C
ATOM    552  O   LEU A 138      -3.806  12.080   7.039 32.22  0.00           O
ATOM    553  N   PHE A 139      -4.725  11.691   5.030 59.66  0.00           N
ATOM    554  CA  PHE A 139      -4.602  13.020   4.511 59.66  0.00           C
ATOM    555  C   PHE A 139      -5.790  13.890   4.834 59.66  0.00           C
ATOM    556  O   PHE A 139      -5.772  15.074   4.508 59.66  0.00           O
ATOM    557  N   SER A 140      -6.827  13.321   5.474 50.13  0.00           N
ATOM    558  CA  SER A 140      -8.140  13.999   5.608 50.13  0.00           C
ATOM    559  C   SER A 140      -8.590  14.636   4.304 50.13  0.00           C
ATOM    560  O   SER A 140      -8.961  15.800   4.273 50.13  0.00           O
ATOM    561  N   ALA A 141      -8.519  13.878   3.227 49.30  0.00           N
ATOM    562  CA  ALA A 141      -8.945  14.332   1.917 49.30  0.00           C
ATOM    563  C   ALA A 141     -10.405  13.939   1.667 49.30  0.00           C
ATOM    564  O   ALA A 141     -10.983  13.084   2.348 49.30  0.00           O
ATOM    565  N   GLU A 142     -11.035  14.621   0.722 35.86  0.00           N
ATOM    566  CA  GLU A 142     -12.471  14.427   0.489 35.86  0.00           C
ATOM    567  C   GLU A 142     -12.738  13.477  -0.702 35.86  0.00           C
ATOM    568  O   GLU A 142     -13.747  12.791  -0.707 35.86  0.00           O
ATOM    569  N   LYS A 143     -11.805  13.444  -1.660 43.24  0.00           N
ATOM    570  CA  LYS A 143     -11.916  12.652  -2.862 43.24  0.00           C
ATOM    571  C   LYS A 143     -10.727  11.686  -3.055 43.24  0.00           C
ATOM    572  O   LYS A 143      -9.539  11.991  -2.732 43.24  0.00           O
```

ATOM	573	N	GLY	A	144	-11.077	10.526	-3.602	49.83	0.00	N
ATOM	574	CA	GLY	A	144	-10.120	9.474	-3.925	49.83	0.00	C
ATOM	575	C	GLY	A	144	-10.352	8.924	-5.300	49.83	0.00	C
ATOM	576	O	GLY	A	144	-11.486	8.740	-5.697	49.83	0.00	O
ATOM	577	N	ILE	A	145	-9.286	8.679	-6.041	38.62	0.00	N
ATOM	578	CA	ILE	A	145	-9.380	8.016	-7.325	38.62	0.00	C
ATOM	579	C	ILE	A	145	-8.437	6.791	-7.270	38.62	0.00	C
ATOM	580	O	ILE	A	145	-7.274	6.920	-6.924	38.62	0.00	O
ATOM	581	N	LEU	A	146	-8.955	5.621	-7.649	51.00	0.00	N
ATOM	582	CA	LEU	A	146	-8.233	4.396	-7.797	51.00	0.00	C
ATOM	583	C	LEU	A	146	-8.251	4.098	-9.279	51.00	0.00	C
ATOM	584	O	LEU	A	146	-9.304	3.843	-9.816	51.00	0.00	O
ATOM	585	N	VAL	A	147	-7.117	4.065	-9.937	42.62	0.00	N
ATOM	586	CA	VAL	A	147	-7.059	3.872	-11.356	42.62	0.00	C
ATOM	587	C	VAL	A	147	-6.211	2.623	-11.646	42.62	0.00	C
ATOM	588	O	VAL	A	147	-5.067	2.470	-11.090	42.62	0.00	O
ATOM	589	N	TYR	A	148	-6.742	1.739	-12.509	47.38	0.00	N
ATOM	590	CA	TYR	A	148	-6.090	0.482	-12.844	47.38	0.00	C
ATOM	591	C	TYR	A	148	-5.595	0.575	-14.257	47.38	0.00	C
ATOM	592	O	TYR	A	148	-6.345	0.903	-15.117	47.38	0.00	O
ATOM	593	N	ILE	A	149	-4.338	0.293	-14.483	37.50	0.00	N
ATOM	594	CA	ILE	A	149	-3.766	0.206	-15.807	37.50	0.00	C
ATOM	595	C	ILE	A	149	-3.430	-1.275	-15.990	37.50	0.00	C
ATOM	596	O	ILE	A	149	-2.545	-1.776	-15.346	37.50	0.00	O
ATOM	597	N	THR	A	150	-4.180	-2.003	-16.800	44.28	0.00	N
ATOM	598	CA	THR	A	150	-3.964	-3.413	-16.968	44.28	0.00	C
ATOM	599	C	THR	A	150	-3.936	-3.690	-18.433	44.28	0.00	C
ATOM	600	O	THR	A	150	-4.311	-2.846	-19.222	44.28	0.00	O
ATOM	601	N	PRO	A	151	-3.486	-4.859	-18.836	38.24	0.00	N
ATOM	602	CA	PRO	A	151	-3.299	-5.051	-20.300	38.24	0.00	C
ATOM	603	C	PRO	A	151	-4.601	-5.273	-21.077	38.24	0.00	C
ATOM	604	O	PRO	A	151	-4.598	-5.259	-22.317	38.24	0.00	O
ATOM	605	N	ASP	A	152	-5.658	-5.505	-20.333	44.34	0.00	N
ATOM	606	CA	ASP	A	152	-6.988	-5.753	-20.794	44.34	0.00	C
ATOM	607	C	ASP	A	152	-7.910	-4.564	-20.707	44.34	0.00	C
ATOM	608	O	ASP	A	152	-8.921	-4.561	-21.326	44.34	0.00	O
ATOM	609	N	ARG	A	153	-7.550	-3.582	-19.909	47.43	0.00	N
ATOM	610	CA	ARG	A	153	-8.418	-2.528	-19.517	47.43	0.00	C
ATOM	611	C	ARG	A	153	-7.808	-1.437	-18.658	47.43	0.00	C
ATOM	612	O	ARG	A	153	-7.169	-1.665	-17.715	47.43	0.00	O
ATOM	613	N	ILE	A	154	-8.107	-0.221	-18.973	36.41	0.00	N
ATOM	614	CA	ILE	A	154	-7.873	0.874	-18.098	36.41	0.00	C
ATOM	615	C	ILE	A	154	-9.177	1.197	-17.432	36.41	0.00	C
ATOM	616	O	ILE	A	154	-10.135	1.182	-18.066	36.41	0.00	O
ATOM	617	N	ALA	A	155	-9.204	1.463	-16.150	46.26	0.00	N
ATOM	618	CA	ALA	A	155	-10.490	1.563	-15.403	46.26	0.00	C
ATOM	619	C	ALA	A	155	-10.307	2.411	-14.178	46.26	0.00	C
ATOM	620	O	ALA	A	155	-9.405	2.165	-13.464	46.26	0.00	O
ATOM	621	N	GLU	A	156	-11.159	3.389	-13.945	38.25	0.00	N
ATOM	622	CA	GLU	A	156	-10.959	4.392	-12.924	38.25	0.00	C
ATOM	623	C	GLU	A	156	-12.193	4.455	-12.023	38.25	0.00	C
ATOM	624	O	GLU	A	156	-13.299	4.455	-12.446	38.25	0.00	O
ATOM	625	N	TYR	A	157	-11.976	4.532	-10.746	48.68	0.00	N
ATOM	626	CA	TYR	A	157	-13.044	4.493	-9.764	48.68	0.00	C
ATOM	627	C	TYR	A	157	-12.934	5.658	-8.804	48.68	0.00	C
ATOM	628	O	TYR	A	157	-11.853	6.011	-8.265	48.68	0.00	O
ATOM	629	N	GLU	A	158	-14.055	6.253	-8.542	43.72	0.00	N
ATOM	630	CA	GLU	A	158	-14.108	7.361	-7.646	43.72	0.00	C
ATOM	631	C	GLU	A	158	-14.532	6.786	-6.302	43.72	0.00	C
ATOM	632	O	GLU	A	158	-15.602	6.184	-6.178	43.72	0.00	O
ATOM	633	N	ILE	A	159	-13.651	6.902	-5.311	48.13	0.00	N
ATOM	634	CA	ILE	A	159	-13.878	6.419	-3.939	48.13	0.00	C
ATOM	635	C	ILE	A	159	-13.755	7.649	-3.027	48.13	0.00	C
ATOM	636	O	ILE	A	159	-12.671	8.212	-2.918	48.13	0.00	O
ATOM	637	N	ASN	A	160	-14.849	8.038	-2.394	47.40	0.00	N
ATOM	638	CA	ASN	A	160	-14.894	9.274	-1.638	47.40	0.00	C
ATOM	639	C	ASN	A	160	-15.165	9.116	-0.146	47.40	0.00	C
ATOM	640	O	ASN	A	160	-14.949	10.056	0.594	47.40	0.00	O
ATOM	641	N	GLU	A	161	-15.536	7.926	0.306	37.06	0.00	N
ATOM	642	CA	GLU	A	161	-15.720	7.704	1.724	37.06	0.00	C
ATOM	643	C	GLU	A	161	-14.467	7.151	2.407	37.06	0.00	C
ATOM	644	O	GLU	A	161	-13.997	6.052	2.116	37.06	0.00	O
ATOM	645	N	PRO	A	162	-13.950	7.900	3.365	52.91	0.00	N
ATOM	646	CA	PRO	A	162	-12.848	7.426	4.187	52.91	0.00	C
ATOM	647	C	PRO	A	162	-13.323	6.439	5.227	52.91	0.00	C
ATOM	648	O	PRO	A	162	-14.483	6.537	5.645	52.91	0.00	O
ATOM	649	N	LEU	A	163	-12.444	5.517	5.661	61.33	0.00	N
ATOM	650	CA	LEU	A	163	-12.805	4.572	6.724	61.33	0.00	C
ATOM	651	C	LEU	A	163	-12.888	5.346	8.019	61.33	0.00	C
ATOM	652	O	LEU	A	163	-12.040	6.223	8.266	61.33	0.00	O
ATOM	653	N	ASP	A	164	-13.876	5.013	8.858	23.30	0.00	N
ATOM	654	CA	ASP	A	164	-13.934	5.590	10.207	23.30	0.00	C
ATOM	655	C	ASP	A	164	-12.616	5.280	10.928	23.30	0.00	C
ATOM	656	O	ASP	A	164	-11.940	4.300	10.622	23.30	0.00	O
ATOM	657	N	GLU	A	165	-12.301	6.090	11.926	46.45	0.00	N
ATOM	658	CA	GLU	A	165	-11.127	5.880	12.758	46.45	0.00	C
ATOM	659	C	GLU	A	165	-11.240	4.594	13.565	46.45	0.00	C
ATOM	660	O	GLU	A	165	-10.265	3.923	13.761	46.45	0.00	O
ATOM	661	N	ALA	A	166	-12.438	4.252	14.016	53.02	0.00	N
ATOM	662	CA	ALA	A	166	-12.662	3.006	14.753	53.02	0.00	C
ATOM	663	C	ALA	A	166	-12.336	1.750	13.921	53.02	0.00	C
ATOM	664	O	ALA	A	166	-11.761	0.808	14.415	53.02	0.00	O

```
ATOM    665  N   THR A 167     -12.694   1.751  12.648 42.30  0.00           N
ATOM    666  CA  THR A 167     -12.382   0.638  11.768 42.30  0.00           C
ATOM    667  C   THR A 167     -10.872   0.497  11.623 42.30  0.00           C
ATOM    668  O   THR A 167     -10.346  -0.601  11.637 42.30  0.00           O
ATOM    669  N   ILE A 168     -10.171   1.619  11.528 51.74  0.00           N
ATOM    670  CA  ILE A 168      -8.732   1.633  11.358 51.74  0.00           C
ATOM    671  C   ILE A 168      -8.078   1.128  12.626 51.74  0.00           C
ATOM    672  O   ILE A 168      -7.058   0.457  12.570 51.74  0.00           O
ATOM    673  N   VAL A 169      -8.665   1.441  13.778 43.45  0.00           N
ATOM    674  CA  VAL A 169      -8.132   0.957  15.026 43.45  0.00           C
ATOM    675  C   VAL A 169      -8.314  -0.550  15.039 43.45  0.00           C
ATOM    676  O   VAL A 169      -7.394  -1.257  15.440 43.45  0.00           O
ATOM    677  N   ARG A 170      -9.472  -1.045  14.590 50.30  0.00           N
ATOM    678  CA  ARG A 170      -9.700  -2.518  14.528 50.30  0.00           C
ATOM    679  C   ARG A 170      -8.661  -3.202  13.604 50.30  0.00           C
ATOM    680  O   ARG A 170      -8.069  -4.207  13.996 50.30  0.00           O
ATOM    681  N   LEU A 171      -8.396  -2.610  12.430 43.66  0.00           N
ATOM    682  CA  LEU A 171      -7.379  -3.127  11.527 43.66  0.00           C
ATOM    683  C   LEU A 171      -6.020  -3.139  12.210 43.66  0.00           C
ATOM    684  O   LEU A 171      -5.257  -4.108  12.109 43.66  0.00           O
ATOM    685  N   ALA A 172      -5.723  -2.049  12.909 49.64  0.00           N
ATOM    686  CA  ALA A 172      -4.457  -1.898  13.604 49.64  0.00           C
ATOM    687  C   ALA A 172      -4.301  -2.947  14.696 49.64  0.00           C
ATOM    688  O   ALA A 172      -3.240  -3.541  14.801 49.64  0.00           O
ATOM    689  N   GLU A 173      -5.352  -3.187  15.479 48.78  0.00           N
ATOM    690  CA  GLU A 173      -5.292  -4.177  16.568 48.78  0.00           C
ATOM    691  C   GLU A 173      -4.915  -5.559  16.053 48.78  0.00           C
ATOM    692  O   GLU A 173      -4.131  -6.255  16.707 48.78  0.00           O
ATOM    693  N   ASP A 174      -5.506  -5.961  14.919 38.26  0.00           N
ATOM    694  CA  ASP A 174      -5.208  -7.255  14.320 38.26  0.00           C
ATOM    695  C   ASP A 174      -3.815  -7.301  13.840 38.26  0.00           C
ATOM    696  O   ASP A 174      -3.169  -8.343  13.873 38.26  0.00           O
ATOM    697  N   THR A 175      -3.325  -6.165  13.384 43.17  0.00           N
ATOM    698  CA  THR A 175      -1.966  -6.171  12.887 43.17  0.00           C
ATOM    699  C   THR A 175      -1.026  -6.460  14.019 43.17  0.00           C
ATOM    700  O   THR A 175      -0.104  -7.267  13.894 43.17  0.00           O
ATOM    701  N   ILE A 176      -1.312  -5.827  15.139 18.83  0.00           N
ATOM    702  CA  ILE A 176      -0.456  -5.907  16.306 18.83  0.00           C
ATOM    703  C   ILE A 176      -0.618  -7.264  17.013 18.83  0.00           C
ATOM    704  O   ILE A 176       0.348  -7.982  17.191 18.83  0.00           O
ATOM    705  N   MSE A 177      -1.846  -7.622  17.359 33.37  0.00           N
ATOM    706  CA  MSE A 177      -2.110  -8.862  18.066 33.37  0.00           C
ATOM    707  C   MSE A 177      -2.036 -10.148  17.184 33.37  0.00           C
ATOM    708  O   MSE A 177      -2.230 -11.232  17.705 33.37  0.00           O
ATOM    709  N   LEU A 178      -1.747 -10.045  15.885 30.21  0.00           N
ATOM    710  CA  LEU A 178      -1.765 -11.195  14.931 30.21  0.00           C
ATOM    711  C   LEU A 178      -3.082 -11.987  14.963 30.21  0.00           C
ATOM    712  O   LEU A 178      -3.059 -13.216  14.908 30.21  0.00           O
ATOM    713  N   GLN A 179      -4.195 -11.295  14.914 48.05  0.00           N
ATOM    714  CA  GLN A 179      -5.488 -11.880  15.107 48.05  0.00           C
ATOM    715  C   GLN A 179      -6.016 -12.818  14.022 48.05  0.00           C
ATOM    716  O   GLN A 179      -6.283 -13.971  14.291 48.05  0.00           O
ATOM    717  N   ASN A 180      -6.209 -12.312  12.822 39.77  0.00           N
ATOM    718  CA  ASN A 180      -6.832 -13.100  11.723 39.77  0.00           C
ATOM    719  C   ASN A 180      -5.958 -13.170  10.495 39.77  0.00           C
ATOM    720  O   ASN A 180      -5.982 -12.302   9.632 39.77  0.00           O
ATOM    721  N   SER A 181      -5.162 -14.213  10.431 58.29  0.00           N
ATOM    722  CA  SER A 181      -4.294 -14.421   9.316 58.29  0.00           C
ATOM    723  C   SER A 181      -4.685 -15.651   8.495 58.29  0.00           C
ATOM    724  O   SER A 181      -4.793 -16.745   9.008 58.29  0.00           O
ATOM    725  N   PRO A 182      -4.770 -15.497   7.199 63.56  0.00           N
ATOM    726  CA  PRO A 182      -4.417 -14.305   6.461 63.56  0.00           C
ATOM    727  C   PRO A 182      -5.557 -13.337   6.500 63.56  0.00           C
ATOM    728  O   PRO A 182      -6.623 -13.675   7.004 63.56  0.00           O
ATOM    729  N   ARG A 183      -5.357 -12.135   5.998 84.53  0.00           N
ATOM    730  CA  ARG A 183      -6.436 -11.191   6.007 84.53  0.00           C
ATOM    731  C   ARG A 183      -7.372 -11.579   4.893 84.53  0.00           C
ATOM    732  O   ARG A 183      -8.603 -11.628   5.107 84.53  0.00           O
ATOM    733  N   PHE A 184      -6.785 -11.851   3.718 54.72  0.00           N
ATOM    734  CA  PHE A 184      -7.550 -12.410   2.590 54.72  0.00           C
ATOM    735  C   PHE A 184      -7.017 -13.778   2.185 54.72  0.00           C
ATOM    736  O   PHE A 184      -5.814 -14.039   2.233 54.72  0.00           O
ATOM    737  N   ASN A 185      -7.926 -14.630   1.714 64.00  0.00           N
ATOM    738  CA  ASN A 185      -7.604 -16.007   1.378 64.00  0.00           C
ATOM    739  C   ASN A 185      -6.563 -16.229   0.291 64.00  0.00           C
ATOM    740  O   ASN A 185      -5.857 -17.232   0.316 64.00  0.00           O
ATOM    741  N   TRP A 186      -6.507 -15.349  -0.690 35.33  0.00           N
ATOM    742  CA  TRP A 186      -5.602 -15.511  -1.821 35.33  0.00           C
ATOM    743  C   TRP A 186      -4.124 -15.190  -1.442 35.33  0.00           C
ATOM    744  O   TRP A 186      -3.168 -15.441  -2.207 35.33  0.00           O
ATOM    745  N   GLU A 187      -3.932 -14.615  -0.259 83.66  0.00           N
ATOM    746  CA  GLU A 187      -2.575 -14.179   0.176 83.66  0.00           C
ATOM    747  C   GLU A 187      -1.511 -15.229   0.225 83.66  0.00           C
ATOM    748  O   GLU A 187      -0.428 -15.028  -0.334 83.66  0.00           O
ATOM    749  N   CYS A 188      -1.801 -16.352   0.884 55.50  0.00           N
ATOM    750  CA  CYS A 188      -0.760 -17.344   1.180 55.50  0.00           C
ATOM    751  C   CYS A 188      -0.146 -17.865  -0.127 55.50  0.00           C
ATOM    752  O   CYS A 188       1.067 -18.120  -0.218 55.50  0.00           O
ATOM    753  N   LYS A 189      -0.970 -17.963  -1.166  7.38  0.00           N
ATOM    754  CA  LYS A 189      -0.511 -18.522  -2.447  7.38  0.00           C
ATOM    755  C   LYS A 189       0.696 -17.731  -3.018  7.38  0.00           C
ATOM    756  O   LYS A 189       1.569 -18.272  -3.724  7.38  0.00           O
```

```
ATOM    757  N   TYR A 190       0.747 -16.439  -2.708 22.21  0.00           N
ATOM    758  CA  TYR A 190       1.763 -15.611  -3.285 22.21  0.00           C
ATOM    759  C   TYR A 190       2.819 -15.235  -2.248 22.21  0.00           C
ATOM    760  O   TYR A 190       3.823 -14.654  -2.584 22.21  0.00           O
ATOM    761  N   CYS A 191       2.587 -15.625  -0.997 36.59  0.00           N
ATOM    762  CA  CYS A 191       3.353 -15.193   0.160 36.59  0.00           C
ATOM    763  C   CYS A 191       4.692 -15.934   0.269 36.59  0.00           C
ATOM    764  O   CYS A 191       4.732 -17.173   0.348 36.59  0.00           O
ATOM    765  N   ILE A 192       5.784 -15.171   0.315 43.02  0.00           N
ATOM    766  CA  ILE A 192       7.106 -15.776   0.279 43.02  0.00           C
ATOM    767  C   ILE A 192       7.409 -16.403   1.615 43.02  0.00           C
ATOM    768  O   ILE A 192       8.394 -17.068   1.711 43.02  0.00           O
ATOM    769  N   PHE A 193       6.578 -16.191   2.632 49.91  0.00           N
ATOM    770  CA  PHE A 193       6.786 -16.794   3.943 49.91  0.00           C
ATOM    771  C   PHE A 193       5.984 -18.074   4.210 49.91  0.00           C
ATOM    772  O   PHE A 193       5.990 -18.637   5.317 49.91  0.00           O
ATOM    773  N   SER A 194       5.262 -18.513   3.198 41.94  0.00           N
ATOM    774  CA  SER A 194       4.413 -19.711   3.328 41.94  0.00           C
ATOM    775  C   SER A 194       5.249 -20.909   3.657 41.94  0.00           C
ATOM    776  O   SER A 194       4.748 -21.746   4.298 41.94  0.00           O
ATOM    777  N   VAL A 195       6.503 -20.952   3.199 53.52  0.00           N
ATOM    778  CA  VAL A 195       7.410 -21.989   3.516 53.52  0.00           C
ATOM    779  C   VAL A 195       7.733 -22.174   4.979 53.52  0.00           C
ATOM    780  O   VAL A 195       8.309 -23.201   5.323 53.52  0.00           O
ATOM    781  N   ILE A 196       7.419 -21.212   5.849 37.19  0.00           N
ATOM    782  CA  ILE A 196       7.718 -21.354   7.295 37.19  0.00           C
ATOM    783  C   ILE A 196       6.564 -20.975   8.148 37.19  0.00           C
ATOM    784  O   ILE A 196       6.683 -20.972   9.331 37.19  0.00           O
ATOM    785  N   CYS A 197       5.424 -20.671   7.561 45.29  0.00           N
ATOM    786  CA  CYS A 197       4.335 -20.157   8.329 45.29  0.00           C
ATOM    787  C   CYS A 197       3.331 -21.219   8.615 45.29  0.00           C
ATOM    788  O   CYS A 197       2.836 -21.849   7.697 45.29  0.00           O
ATOM    789  N   PRO A 198       2.960 -21.375   9.870 31.76  0.00           N
ATOM    790  CA  PRO A 198       1.970 -22.338  10.280 31.76  0.00           C
ATOM    791  C   PRO A 198       0.542 -22.046   9.971 31.76  0.00           C
ATOM    792  O   PRO A 198      -0.290 -22.938  10.156 31.76  0.00           O
ATOM    793  N   ALA A 199       0.227 -20.829   9.562 39.98  0.00           N
ATOM    794  CA  ALA A 199      -1.167 -20.431   9.353 39.98  0.00           C
ATOM    795  C   ALA A 199      -1.570 -20.467   7.877 39.98  0.00           C
ATOM    796  O   ALA A 199      -2.731 -20.267   7.563 39.98  0.00           O
ATOM    797  N   LYS A 200      -0.591 -20.655   6.991 26.21  0.00           N
ATOM    798  CA  LYS A 200      -0.778 -20.823   5.548 26.21  0.00           C
ATOM    799  C   LYS A 200      -1.946 -21.672   5.236 26.21  0.00           C
ATOM    800  O   LYS A 200      -2.051 -22.735   5.769 26.21  0.00           O
ATOM    801  N   LEU A 201      -2.799 -21.218   4.328 31.76  0.00           N
ATOM    802  CA  LEU A 201      -3.935 -22.003   3.876 31.76  0.00           C
ATOM    803  C   LEU A 201      -3.511 -23.046   2.859 31.76  0.00           C
ATOM    804  O   LEU A 201      -2.649 -22.775   2.009 31.76  0.00           O
ATOM    805  N   THR A 202      -4.127 -24.225   2.953 46.74  0.00           N
ATOM    806  CA  THR A 202      -3.796 -25.369   2.082 46.74  0.00           C
ATOM    807  C   THR A 202      -5.038 -25.920   1.370 46.74  0.00           C
ATOM    808  O   THR A 202      -6.101 -25.964   1.999 46.74  0.00           O
TER     809      THR A 202
ENDMDL
END
REMARK   1 **********************************************************************
REMARK   1 Start File NAT_vs_DEC3_dSi_colored.pdb
REMARK   1 The occupancy record was modified to represent dSi := Si(DEC)-Si(NAT).
REMARK   1 Occ=0.00 means dSi=-1.285567 kB; Occ=99.99 means dSi=1.380460 kB.
MODEL        0
ATOM      1  N   MSE A   1      23.268  -4.104 -18.521 40.19  0.00           N
ATOM      2  CA  MSE A   1      23.439  -2.722 -18.016 40.19  0.00           C
ATOM      3  C   MSE A   1      22.152  -2.161 -17.383 40.19  0.00           C
ATOM      4  O   MSE A   1      22.147  -1.650 -16.252 40.19  0.00           O
ATOM      5  N   ILE A   2      21.045  -2.254 -18.100 42.66  0.00           N
ATOM      6  CA  ILE A   2      19.816  -1.632 -17.608 42.66  0.00           C
ATOM      7  C   ILE A   2      19.269  -2.405 -16.412 42.66  0.00           C
ATOM      8  O   ILE A   2      18.935  -1.835 -15.391 42.66  0.00           O
ATOM      9  N   THR A   3      19.186  -3.703 -16.563 29.88  0.00           N
ATOM     10  CA  THR A   3      18.718  -4.606 -15.498 29.88  0.00           C
ATOM     11  C   THR A   3      19.478  -4.398 -14.212 29.88  0.00           C
ATOM     12  O   THR A   3      18.884  -4.188 -13.150 29.88  0.00           O
ATOM     13  N   GLU A   4      20.800  -4.447 -14.318 21.02  0.00           N
ATOM     14  CA  GLU A   4      21.687  -4.148 -13.195 21.02  0.00           C
ATOM     15  C   GLU A   4      21.342  -2.832 -12.484 21.02  0.00           C
ATOM     16  O   GLU A   4      21.232  -2.791 -11.242 21.02  0.00           O
ATOM     17  N   PHE A   5      21.133  -1.765 -13.247 47.14  0.00           N
ATOM     18  CA  PHE A   5      20.826  -0.486 -12.602 47.14  0.00           C
ATOM     19  C   PHE A   5      19.486  -0.520 -11.886 47.14  0.00           C
ATOM     20  O   PHE A   5      19.415  -0.122 -10.736 47.14  0.00           O
ATOM     21  N   LEU A   6      18.429  -1.039 -12.529 26.94  0.00           N
ATOM     22  CA  LEU A   6      17.100  -1.072 -11.873 26.94  0.00           C
ATOM     23  C   LEU A   6      17.119  -1.921 -10.586 26.94  0.00           C
ATOM     24  O   LEU A   6      16.551  -1.545  -9.531 26.94  0.00           O
ATOM     25  N   LEU A   7      17.767  -3.071 -10.672 35.53  0.00           N
ATOM     26  CA  LEU A   7      17.843  -3.929  -9.486 35.53  0.00           C
ATOM     27  C   LEU A   7      18.603  -3.241  -8.341 35.53  0.00           C
ATOM     28  O   LEU A   7      18.190  -3.292  -7.184 35.53  0.00           O
ATOM     29  N   LYS A   8      19.691  -2.566  -8.693 28.46  0.00           N
ATOM     30  CA  LYS A   8      20.463  -1.856  -7.680 28.46  0.00           C
ATOM     31  C   LYS A   8      19.628  -0.762  -7.044 28.46  0.00           C
ATOM     32  O   LYS A   8      19.527  -0.688  -5.819 28.46  0.00           O
```

ATOM	33	N	LYS	A	9	18.947	0.036	-7.860	81.62	0.00	N
ATOM	34	CA	LYS	A	9	18.144	1.113	-7.292	81.62	0.00	C
ATOM	35	C	LYS	A	9	17.009	0.545	-6.471	81.62	0.00	C
ATOM	36	O	LYS	A	9	16.708	1.086	-5.402	81.62	0.00	O
ATOM	37	N	LYS	A	10	16.379	-0.547	-6.912	48.84	0.00	N
ATOM	38	CA	LYS	A	10	15.262	-1.072	-6.116	48.84	0.00	C
ATOM	39	C	LYS	A	10	15.660	-1.681	-4.726	48.84	0.00	C
ATOM	40	O	LYS	A	10	14.931	-1.547	-3.695	48.84	0.00	O
ATOM	41	N	LEU	A	11	16.798	-2.366	-4.699	40.34	0.00	N
ATOM	42	CA	LEU	A	11	17.328	-2.879	-3.411	40.34	0.00	C
ATOM	43	C	LEU	A	11	17.613	-1.686	-2.439	40.34	0.00	C
ATOM	44	O	LEU	A	11	17.179	-1.698	-1.270	40.34	0.00	O
ATOM	45	N	GLU	A	12	18.299	-0.655	-2.945	37.85	0.00	N
ATOM	46	CA	GLU	A	12	18.475	0.605	-2.181	37.85	0.00	C
ATOM	47	C	GLU	A	12	17.179	1.213	-1.616	37.85	0.00	C
ATOM	48	O	GLU	A	12	17.048	1.390	-0.426	37.85	0.00	O
ATOM	49	N	GLU	A	13	16.225	1.536	-2.461	55.83	0.00	N
ATOM	50	CA	GLU	A	13	14.901	1.950	-1.974	55.83	0.00	C
ATOM	51	C	GLU	A	13	14.358	1.037	-0.868	55.83	0.00	C
ATOM	52	O	GLU	A	13	13.966	1.505	0.190	55.83	0.00	O
ATOM	53	N	HIS	A	14	14.335	-0.267	-1.101	31.91	0.00	N
ATOM	54	CA	HIS	A	14	13.794	-1.199	-0.106	31.91	0.00	C
ATOM	55	C	HIS	A	14	14.560	-1.126	1.236	31.91	0.00	C
ATOM	56	O	HIS	A	14	13.970	-1.122	2.322	31.91	0.00	O
ATOM	57	N	LEU	A	15	15.884	-1.079	1.145	27.01	0.00	N
ATOM	58	CA	LEU	A	15	16.750	-1.011	2.322	27.01	0.00	C
ATOM	59	C	LEU	A	15	16.724	0.377	2.988	27.01	0.00	C
ATOM	60	O	LEU	A	15	17.114	0.492	4.140	27.01	0.00	O
ATOM	61	N	SER	A	16	16.283	1.421	2.277	43.85	0.00	N
ATOM	62	CA	SER	A	16	16.215	2.761	2.870	43.85	0.00	C
ATOM	63	C	SER	A	16	15.126	2.866	3.937	43.85	0.00	C
ATOM	64	O	SER	A	16	15.184	3.766	4.746	43.85	0.00	O
ATOM	65	N	HIS	A	17	14.126	1.987	3.905	38.37	0.00	N
ATOM	66	CA	HIS	A	17	12.931	2.175	4.710	38.37	0.00	C
ATOM	67	C	HIS	A	17	12.911	1.290	5.925	38.37	0.00	C
ATOM	68	O	HIS	A	17	12.211	0.285	5.939	38.37	0.00	O
ATOM	69	N	VAL	A	18	13.667	1.662	6.952	36.75	0.00	N
ATOM	70	CA	VAL	A	18	13.649	0.930	8.226	36.75	0.00	C
ATOM	71	C	VAL	A	18	12.455	1.416	9.073	36.75	0.00	C
ATOM	72	O	VAL	A	18	12.231	2.619	9.198	36.75	0.00	O
ATOM	73	N	LYS	A	19	11.711	0.480	9.654	47.79	0.00	N
ATOM	74	CA	LYS	A	19	10.394	0.738	10.251	47.79	0.00	C
ATOM	75	C	LYS	A	19	10.466	0.797	11.779	47.79	0.00	C
ATOM	76	O	LYS	A	19	11.024	-0.102	12.394	47.79	0.00	O
ATOM	77	N	GLU	A	20	9.892	1.832	12.391	14.56	0.00	N
ATOM	78	CA	GLU	A	20	10.003	2.007	13.848	14.56	0.00	C
ATOM	79	C	GLU	A	20	8.986	1.178	14.597	14.56	0.00	C
ATOM	80	O	GLU	A	20	7.952	0.808	14.049	14.56	0.00	O
ATOM	81	N	GLU	A	21	9.300	0.899	15.861	5.07	0.00	N
ATOM	82	CA	GLU	A	21	8.634	-0.168	16.607	5.07	0.00	C
ATOM	83	C	GLU	A	21	7.153	0.124	16.844	5.07	0.00	C
ATOM	84	O	GLU	A	21	6.369	-0.811	17.040	5.07	0.00	O
ATOM	85	N	ASN	A	22	6.774	1.399	16.808	50.91	0.00	N
ATOM	86	CA	ASN	A	22	5.371	1.790	16.970	50.91	0.00	C
ATOM	87	C	ASN	A	22	4.716	2.391	15.715	50.91	0.00	C
ATOM	88	O	ASN	A	22	3.648	3.000	15.824	50.91	0.00	O
ATOM	89	N	THR	A	23	5.339	2.269	14.540	46.08	0.00	N
ATOM	90	CA	THR	A	23	4.636	2.674	13.297	46.08	0.00	C
ATOM	91	C	THR	A	23	3.937	1.492	12.597	46.08	0.00	C
ATOM	92	O	THR	A	23	4.343	0.315	12.707	46.08	0.00	O
ATOM	93	N	ILE	A	24	2.863	1.841	11.907	51.79	0.00	N
ATOM	94	CA	ILE	A	24	2.133	0.908	11.089	51.79	0.00	C
ATOM	95	C	ILE	A	24	1.893	1.588	9.781	51.79	0.00	C
ATOM	96	O	ILE	A	24	1.270	2.646	9.760	51.79	0.00	O
ATOM	97	N	TYR	A	25	2.393	1.015	8.691	52.24	0.00	N
ATOM	98	CA	TYR	A	25	2.072	1.558	7.393	52.24	0.00	C
ATOM	99	C	TYR	A	25	0.609	1.246	7.051	52.24	0.00	C
ATOM	100	O	TYR	A	25	0.029	0.273	7.510	52.24	0.00	O
ATOM	101	N	VAL	A	26	0.019	2.088	6.230	68.16	0.00	N
ATOM	102	CA	VAL	A	26	-1.326	1.856	5.716	68.16	0.00	C
ATOM	103	C	VAL	A	26	-1.394	0.504	4.989	68.16	0.00	C
ATOM	104	O	VAL	A	26	-2.320	-0.244	5.138	68.16	0.00	O
ATOM	105	N	THR	A	27	-0.413	0.264	4.153	51.67	0.00	N
ATOM	106	CA	THR	A	27	-0.225	-0.940	3.432	51.67	0.00	C
ATOM	107	C	THR	A	27	-0.214	-2.155	4.325	51.67	0.00	C
ATOM	108	O	THR	A	27	-0.816	-3.189	4.005	51.67	0.00	O
ATOM	109	N	ASP	A	28	0.421	-2.066	5.476	63.07	0.00	N
ATOM	110	CA	ASP	A	28	0.217	-3.125	6.447	63.07	0.00	C
ATOM	111	C	ASP	A	28	-1.221	-3.366	6.899	63.07	0.00	C
ATOM	112	O	ASP	A	28	-1.560	-4.494	7.172	63.07	0.00	O
ATOM	113	N	LEU	A	29	-2.082	-2.355	6.958	61.09	0.00	N
ATOM	114	CA	LEU	A	29	-3.418	-2.565	7.521	61.09	0.00	C
ATOM	115	C	LEU	A	29	-4.304	-3.447	6.637	61.09	0.00	C
ATOM	116	O	LEU	A	29	-5.335	-3.962	7.065	61.09	0.00	O
ATOM	117	N	VAL	A	30	-3.930	-3.597	5.378	62.86	0.00	N
ATOM	118	CA	VAL	A	30	-4.776	-4.325	4.449	62.86	0.00	C
ATOM	119	C	VAL	A	30	-4.099	-5.619	4.014	62.86	0.00	C
ATOM	120	O	VAL	A	30	-4.413	-6.174	2.971	62.86	0.00	O
ATOM	121	N	ARG	A	31	-3.154	-6.069	4.828	99.99	0.00	N
ATOM	122	CA	ARG	A	31	-2.426	-7.287	4.613	99.99	0.00	C
ATOM	123	C	ARG	A	31	-2.433	-8.174	5.834	99.99	0.00	C
ATOM	124	O	ARG	A	31	-2.927	-7.806	6.907	99.99	0.00	O

```
ATOM    125  N   CYS A  32      -1.955  -9.395   5.616 60.45  0.00           N
ATOM    126  CA  CYS A  32      -1.910 -10.426   6.637 60.45  0.00           C
ATOM    127  C   CYS A  32      -1.015  -9.951   7.770 60.45  0.00           C
ATOM    128  O   CYS A  32       0.134  -9.567   7.534 60.45  0.00           O
ATOM    129  N   PRO A  33      -1.508 -10.024   8.999 51.71  0.00           N
ATOM    130  CA  PRO A  33      -0.664  -9.610  10.083 51.71  0.00           C
ATOM    131  C   PRO A  33       0.611 -10.429  10.223 51.71  0.00           C
ATOM    132  O   PRO A  33       1.694  -9.852  10.411 51.71  0.00           O
ATOM    133  N   ARG A  34       0.512 -11.757  10.119 62.60  0.00           N
ATOM    134  CA  ARG A  34       1.689 -12.574  10.256 62.60  0.00           C
ATOM    135  C   ARG A  34       2.720 -12.198   9.218 62.60  0.00           C
ATOM    136  O   ARG A  34       3.911 -12.267   9.453 62.60  0.00           O
ATOM    137  N   ARG A  35       2.276 -11.837   8.033 46.60  0.00           N
ATOM    138  CA  ARG A  35       3.238 -11.530   6.965 46.60  0.00           C
ATOM    139  C   ARG A  35       3.971 -10.221   7.251 46.60  0.00           C
ATOM    140  O   ARG A  35       5.151 -10.042   6.901 46.60  0.00           O
ATOM    141  N   VAL A  36       3.228  -9.297   7.848 66.80  0.00           N
ATOM    142  CA  VAL A  36       3.791  -7.981   8.175 66.80  0.00           C
ATOM    143  C   VAL A  36       4.864  -8.200   9.230 66.80  0.00           C
ATOM    144  O   VAL A  36       5.972  -7.734   9.085 66.80  0.00           O
ATOM    145  N   ARG A  37       4.561  -8.972  10.259 57.00  0.00           N
ATOM    146  CA  ARG A  37       5.600  -9.259  11.227 57.00  0.00           C
ATOM    147  C   ARG A  37       6.749 -10.045  10.630 57.00  0.00           C
ATOM    148  O   ARG A  37       7.879  -9.890  11.061 57.00  0.00           O
ATOM    149  N   TYR A  38       6.481 -10.904   9.649 57.26  0.00           N
ATOM    150  CA  TYR A  38       7.570 -11.660   9.036 57.26  0.00           C
ATOM    151  C   TYR A  38       8.499 -10.724   8.363 57.26  0.00           C
ATOM    152  O   TYR A  38       9.689 -10.942   8.377 57.26  0.00           O
ATOM    153  N   GLU A  39       7.963  -9.678   7.760 53.99  0.00           N
ATOM    154  CA  GLU A  39       8.816  -8.667   7.126 53.99  0.00           C
ATOM    155  C   GLU A  39       9.769  -7.904   8.077 53.99  0.00           C
ATOM    156  O   GLU A  39      10.773  -7.394   7.597 53.99  0.00           O
ATOM    157  N   SER A  40       9.464  -7.844   9.380 43.00  0.00           N
ATOM    158  CA  SER A  40      10.398  -7.338  10.412 43.00  0.00           C
ATOM    159  C   SER A  40      11.345  -8.393  10.958 43.00  0.00           C
ATOM    160  O   SER A  40      12.540  -8.130  11.114 43.00  0.00           O
ATOM    161  N   GLU A  41      10.808  -9.578  11.263 45.51  0.00           N
ATOM    162  CA  GLU A  41      11.604 -10.637  11.865 45.51  0.00           C
ATOM    163  C   GLU A  41      12.456 -11.415  10.889 45.51  0.00           C
ATOM    164  O   GLU A  41      13.321 -12.135  11.370 45.51  0.00           O
ATOM    165  N   TYR A  42      12.238 -11.336   9.566 33.77  0.00           N
ATOM    166  CA  TYR A  42      13.052 -12.135   8.586 33.77  0.00           C
ATOM    167  C   TYR A  42      13.369 -11.290   7.378 33.77  0.00           C
ATOM    168  O   TYR A  42      13.024 -11.599   6.196 33.77  0.00           O
ATOM    169  N   LYS A  43      14.059 -10.210   7.691 72.52  0.00           N
ATOM    170  CA  LYS A  43      14.286  -9.169   6.756 72.52  0.00           C
ATOM    171  C   LYS A  43      15.175  -9.664   5.651 72.52  0.00           C
ATOM    172  O   LYS A  43      15.118  -9.127   4.534 72.52  0.00           O
ATOM    173  N   GLU A  44      16.024 -10.635   5.936 49.66  0.00           N
ATOM    174  CA  GLU A  44      16.906 -11.087   4.887 49.66  0.00           C
ATOM    175  C   GLU A  44      16.077 -11.799   3.791 49.66  0.00           C
ATOM    176  O   GLU A  44      16.309 -11.615   2.572 49.66  0.00           O
ATOM    177  N   LEU A  45      15.070 -12.551   4.234 32.70  0.00           N
ATOM    178  CA  LEU A  45      14.177 -13.244   3.311 32.70  0.00           C
ATOM    179  C   LEU A  45      13.268 -12.185   2.642 32.70  0.00           C
ATOM    180  O   LEU A  45      13.095 -12.177   1.406 32.70  0.00           O
ATOM    181  N   ALA A  46      12.710 -11.278   3.448 71.21  0.00           N
ATOM    182  CA  ALA A  46      11.893 -10.178   2.897 71.21  0.00           C
ATOM    183  C   ALA A  46      12.519  -9.534   1.649 71.21  0.00           C
ATOM    184  O   ALA A  46      11.825  -9.262   0.709 71.21  0.00           O
ATOM    185  N   ILE A  47      13.830  -9.341   1.634 54.66  0.00           N
ATOM    186  CA  ILE A  47      14.526  -8.691   0.538 54.66  0.00           C
ATOM    187  C   ILE A  47      14.299  -9.332  -0.838 54.66  0.00           C
ATOM    188  O   ILE A  47      14.451  -8.669  -1.895 54.66  0.00           O
ATOM    189  N   SER A  48      13.959 -10.620  -0.874 68.54  0.00           N
ATOM    190  CA  SER A  48      13.715 -11.277  -2.169 68.54  0.00           C
ATOM    191  C   SER A  48      12.509 -10.666  -2.882 68.54  0.00           C
ATOM    192  O   SER A  48      12.401 -10.723  -4.108 68.54  0.00           O
ATOM    193  N   GLN A  49      11.610 -10.068  -2.123 57.91  0.00           N
ATOM    194  CA  GLN A  49      10.459  -9.434  -2.743 57.91  0.00           C
ATOM    195  C   GLN A  49      10.847  -8.226  -3.623 57.91  0.00           C
ATOM    196  O   GLN A  49      10.116  -7.889  -4.482 57.91  0.00           O
ATOM    197  N   VAL A  50      12.031  -7.643  -3.424 53.84  0.00           N
ATOM    198  CA  VAL A  50      12.659  -6.700  -4.358 53.84  0.00           C
ATOM    199  C   VAL A  50      12.750  -7.176  -5.802 53.84  0.00           C
ATOM    200  O   VAL A  50      12.805  -6.373  -6.754 53.84  0.00           O
ATOM    201  N   TYR A  51      12.865  -8.468  -5.991 36.40  0.00           N
ATOM    202  CA  TYR A  51      13.231  -8.953  -7.306 36.40  0.00           C
ATOM    203  C   TYR A  51      12.000  -9.515  -8.013 36.40  0.00           C
ATOM    204  O   TYR A  51      12.116 -10.179  -9.031 36.40  0.00           O
ATOM    205  N   ALA A  52      10.823  -9.258  -7.445 43.22  0.00           N
ATOM    206  CA  ALA A  52       9.580  -9.586  -8.075 43.22  0.00           C
ATOM    207  C   ALA A  52       9.375  -8.694  -9.336 43.22  0.00           C
ATOM    208  O   ALA A  52       9.152  -7.478  -9.216 43.22  0.00           O
ATOM    209  N   PRO A  53       9.401  -9.282 -10.541 57.51  0.00           N
ATOM    210  CA  PRO A  53       9.338  -8.519 -11.804 57.51  0.00           C
ATOM    211  C   PRO A  53       8.051  -7.715 -11.969 57.51  0.00           C
ATOM    212  O   PRO A  53       8.107  -6.662 -12.589 57.51  0.00           O
ATOM    213  N   SER A  54       6.912  -8.185 -11.428 25.24  0.00           N
ATOM    214  CA  SER A  54       5.662  -7.382 -11.372 25.24  0.00           C
ATOM    215  C   SER A  54       5.807  -6.143 -10.551 25.24  0.00           C
ATOM    216  O   SER A  54       5.159  -5.116 -10.806 25.24  0.00           O
```

ATOM	217	N	ALA	A	55	6.666	-6.210	-9.534	34.28	0.00	N
ATOM	218	CA	ALA	A	55	6.871	-5.023	-8.707	34.28	0.00	C
ATOM	219	C	ALA	A	55	7.756	-4.055	-9.490	34.28	0.00	C
ATOM	220	O	ALA	A	55	7.598	-2.860	-9.403	34.28	0.00	O
ATOM	221	N	ILE	A	56	8.662	-4.543	-10.314	33.53	0.00	N
ATOM	222	CA	ILE	A	56	9.562	-3.610	-11.005	33.53	0.00	C
ATOM	223	C	ILE	A	56	8.724	-2.964	-12.115	33.53	0.00	C
ATOM	224	O	ILE	A	56	8.790	-1.755	-12.348	33.53	0.00	O
ATOM	225	N	LEU	A	57	7.912	-3.805	-12.760	18.72	0.00	N
ATOM	226	CA	LEU	A	57	6.935	-3.366	-13.747	18.72	0.00	C
ATOM	227	C	LEU	A	57	6.033	-2.336	-13.087	18.72	0.00	C
ATOM	228	O	LEU	A	57	5.874	-1.207	-13.590	18.72	0.00	O
ATOM	229	N	GLY	A	58	5.483	-2.701	-11.946	37.26	0.00	N
ATOM	230	CA	GLY	A	58	4.634	-1.806	-11.182	37.26	0.00	C
ATOM	231	C	GLY	A	58	5.258	-0.473	-10.875	37.26	0.00	C
ATOM	232	O	GLY	A	58	4.607	0.560	-11.036	37.26	0.00	O
ATOM	233	N	ASP	A	59	6.514	-0.439	-10.443	27.71	0.00	N
ATOM	234	CA	ASP	A	59	7.150	0.844	-10.105	27.71	0.00	C
ATOM	235	C	ASP	A	59	7.486	1.775	-11.275	27.71	0.00	C
ATOM	236	O	ASP	A	59	7.461	2.943	-11.121	27.71	0.00	O
ATOM	237	N	ILE	A	60	7.874	1.212	-12.405	13.66	0.00	N
ATOM	238	CA	ILE	A	60	8.072	1.923	-13.651	13.66	0.00	C
ATOM	239	C	ILE	A	60	6.774	2.586	-14.133	13.66	0.00	C
ATOM	240	O	ILE	A	60	6.798	3.761	-14.559	13.66	0.00	O
ATOM	241	N	LEU	A	61	5.641	1.870	-14.058	31.03	0.00	N
ATOM	242	CA	LEU	A	61	4.334	2.454	-14.392	31.03	0.00	C
ATOM	243	C	LEU	A	61	4.024	3.653	-13.537	31.03	0.00	C
ATOM	244	O	LEU	A	61	3.668	4.678	-14.050	31.03	0.00	O
ATOM	245	N	HIS	A	62	4.172	3.508	-12.232	54.69	0.00	N
ATOM	246	CA	HIS	A	62	3.968	4.616	-11.306	54.69	0.00	C
ATOM	247	C	HIS	A	62	4.884	5.714	-11.684	54.69	0.00	C
ATOM	248	O	HIS	A	62	4.443	6.854	-11.841	54.69	0.00	O
ATOM	249	N	LEU	A	63	6.158	5.423	-11.888	12.94	0.00	N
ATOM	250	CA	LEU	A	63	7.034	6.526	-12.325	12.94	0.00	C
ATOM	251	C	LEU	A	63	6.405	7.217	-13.537	12.94	0.00	C
ATOM	252	O	LEU	A	63	6.167	8.394	-13.497	12.94	0.00	O
ATOM	253	N	GLY	A	64	6.109	6.499	-14.603	20.96	0.00	N
ATOM	254	CA	GLY	A	64	5.719	7.158	-15.811	20.96	0.00	C
ATOM	255	C	GLY	A	64	4.400	7.890	-15.740	20.96	0.00	C
ATOM	256	O	GLY	A	64	4.174	8.755	-16.509	20.96	0.00	O
ATOM	257	N	LEU	A	65	3.499	7.454	-14.886	35.09	0.00	N
ATOM	258	CA	LEU	A	65	2.129	7.928	-14.922	35.09	0.00	C
ATOM	259	C	LEU	A	65	2.136	9.075	-13.968	35.09	0.00	C
ATOM	260	O	LEU	A	65	1.699	10.153	-14.266	35.09	0.00	O
ATOM	261	N	GLU	A	66	2.672	8.845	-12.789	79.25	0.00	N
ATOM	262	CA	GLU	A	66	3.061	9.970	-11.943	79.25	0.00	C
ATOM	263	C	GLU	A	66	3.702	11.154	-12.688	79.25	0.00	C
ATOM	264	O	GLU	A	66	3.297	12.272	-12.436	79.25	0.00	O
ATOM	265	N	SER	A	67	4.665	10.981	-13.581	33.56	0.00	N
ATOM	266	CA	SER	A	67	5.183	12.221	-14.250	33.56	0.00	C
ATOM	267	C	SER	A	67	4.100	13.012	-15.067	33.56	0.00	C
ATOM	268	O	SER	A	67	4.182	14.248	-15.203	33.56	0.00	O
ATOM	269	N	VAL	A	68	3.091	12.293	-15.600	22.61	0.00	N
ATOM	270	CA	VAL	A	68	1.939	12.934	-16.238	22.61	0.00	C
ATOM	271	C	VAL	A	68	1.158	13.773	-15.231	22.61	0.00	C
ATOM	272	O	VAL	A	68	0.645	14.865	-15.520	22.61	0.00	O
ATOM	273	N	LEU	A	69	1.040	13.216	-14.047	39.64	0.00	N
ATOM	274	CA	LEU	A	69	0.261	13.818	-13.004	39.64	0.00	C
ATOM	275	C	LEU	A	69	0.848	15.118	-12.430	39.64	0.00	C
ATOM	276	O	LEU	A	69	0.134	15.992	-11.952	39.64	0.00	O
ATOM	277	N	LYS	A	70	2.165	15.215	-12.445	56.21	0.00	N
ATOM	278	CA	LYS	A	70	2.860	16.356	-11.911	56.21	0.00	C
ATOM	279	C	LYS	A	70	2.708	17.450	-12.934	56.21	0.00	C
ATOM	280	O	LYS	A	70	2.497	18.596	-12.562	56.21	0.00	O
ATOM	281	N	GLY	A	71	2.769	17.105	-14.229	41.93	0.00	N
ATOM	282	CA	GLY	A	71	2.550	18.111	-15.305	41.93	0.00	C
ATOM	283	C	GLY	A	71	1.130	18.685	-15.376	41.93	0.00	C
ATOM	284	O	GLY	A	71	0.821	19.815	-14.923	41.93	0.00	O
ATOM	285	N	ASN	A	72	0.217	17.876	-15.876	45.44	0.00	N
ATOM	286	CA	ASN	A	72	-1.095	18.370	-16.206	45.44	0.00	C
ATOM	287	C	ASN	A	72	-2.040	18.612	-15.074	45.44	0.00	C
ATOM	288	O	ASN	A	72	-2.921	19.420	-15.211	45.44	0.00	O
ATOM	289	N	PHE	A	73	-1.880	17.933	-13.966	59.09	0.00	N
ATOM	290	CA	PHE	A	73	-2.766	18.118	-12.847	59.09	0.00	C
ATOM	291	C	PHE	A	73	-2.091	18.771	-11.639	59.09	0.00	C
ATOM	292	O	PHE	A	73	-2.674	18.861	-10.593	59.09	0.00	O
ATOM	293	N	ASN	A	74	-0.838	19.166	-11.735	50.33	0.00	N
ATOM	294	CA	ASN	A	74	-0.201	19.781	-10.582	50.33	0.00	C
ATOM	295	C	ASN	A	74	-0.355	18.949	-9.279	50.33	0.00	C
ATOM	296	O	ASN	A	74	-0.590	19.474	-8.165	50.33	0.00	O
ATOM	297	N	ALA	A	75	-0.160	17.641	-9.414	51.14	0.00	N
ATOM	298	CA	ALA	A	75	-0.115	16.792	-8.245	51.14	0.00	C
ATOM	299	C	ALA	A	75	1.282	16.777	-7.622	51.14	0.00	C
ATOM	300	O	ALA	A	75	2.270	16.992	-8.289	51.14	0.00	O
ATOM	301	N	GLU	A	76	1.336	16.530	-6.332	46.53	0.00	N
ATOM	302	CA	GLU	A	76	2.559	16.069	-5.695	46.53	0.00	C
ATOM	303	C	GLU	A	76	2.544	14.546	-5.834	46.53	0.00	C
ATOM	304	O	GLU	A	76	1.466	13.957	-5.865	46.53	0.00	O
ATOM	305	N	THR	A	77	3.710	13.915	-5.909	53.48	0.00	N
ATOM	306	CA	THR	A	77	3.794	12.484	-6.087	53.48	0.00	C
ATOM	307	C	THR	A	77	4.639	11.898	-4.978	53.48	0.00	C
ATOM	308	O	THR	A	77	5.510	12.566	-4.500	53.48	0.00	O

ATOM	309	N	GLU	A	78	4.321	10.666	-4.562	67.11	0.00	N
ATOM	310	CA	GLU	A	78	4.988	9.920	-3.517	67.11	0.00	C
ATOM	311	C	GLU	A	78	5.020	10.703	-2.232	67.11	0.00	C
ATOM	312	O	GLU	A	78	6.063	10.936	-1.623	67.11	0.00	O
ATOM	313	N	VAL	A	79	3.826	11.074	-1.816	53.82	0.00	N
ATOM	314	CA	VAL	A	79	3.635	12.010	-0.767	53.82	0.00	C
ATOM	315	C	VAL	A	79	3.474	11.303	0.544	53.82	0.00	C
ATOM	316	O	VAL	A	79	2.510	10.573	0.774	53.82	0.00	O
ATOM	317	N	GLU	A	80	4.416	11.580	1.421	38.92	0.00	N
ATOM	318	CA	GLU	A	80	4.530	10.857	2.668	38.92	0.00	C
ATOM	319	C	GLU	A	80	3.901	11.655	3.780	38.92	0.00	C
ATOM	320	O	GLU	A	80	4.090	12.840	3.853	38.92	0.00	O
ATOM	321	N	THR	A	81	3.177	11.027	4.670	38.45	0.00	N
ATOM	322	CA	THR	A	81	2.510	11.744	5.761	38.45	0.00	C
ATOM	323	C	THR	A	81	2.133	10.811	6.884	38.45	0.00	C
ATOM	324	O	THR	A	81	2.164	9.597	6.754	38.45	0.00	O
ATOM	325	N	LEU	A	82	1.667	11.373	7.976	48.62	0.00	N
ATOM	326	CA	LEU	A	82	1.644	10.601	9.202	48.62	0.00	C
ATOM	327	C	LEU	A	82	0.492	11.060	10.035	48.62	0.00	C
ATOM	328	O	LEU	A	82	0.098	12.203	9.950	48.62	0.00	O
ATOM	329	N	ARG	A	83	-0.124	10.141	10.761	25.29	0.00	N
ATOM	330	CA	ARG	A	83	-1.326	10.449	11.518	25.29	0.00	C
ATOM	331	C	ARG	A	83	-1.368	9.467	12.659	25.29	0.00	C
ATOM	332	O	ARG	A	83	-0.926	8.327	12.512	25.29	0.00	O
ATOM	333	N	GLU	A	84	-1.873	9.890	13.809	20.97	0.00	N
ATOM	334	CA	GLU	A	84	-1.727	9.022	14.978	20.97	0.00	C
ATOM	335	C	GLU	A	84	-3.042	8.425	15.425	20.97	0.00	C
ATOM	336	O	GLU	A	84	-4.110	8.996	15.183	20.97	0.00	O
ATOM	337	N	ILE	A	85	-2.918	7.275	16.095	55.92	0.00	N
ATOM	338	CA	ILE	A	85	-4.029	6.451	16.449	55.92	0.00	C
ATOM	339	C	ILE	A	85	-3.781	5.841	17.813	55.92	0.00	C
ATOM	340	O	ILE	A	85	-2.643	5.507	18.123	55.92	0.00	O
ATOM	341	N	ASN	A	86	-4.840	5.701	18.619	48.27	0.00	N
ATOM	342	CA	ASN	A	86	-4.761	5.021	19.937	48.27	0.00	C
ATOM	343	C	ASN	A	86	-5.250	3.566	19.895	48.27	0.00	C
ATOM	344	O	ASN	A	86	-6.399	3.293	19.511	48.27	0.00	O
ATOM	345	N	VAL	A	87	-4.383	2.645	20.322	38.64	0.00	N
ATOM	346	CA	VAL	A	87	-4.703	1.227	20.308	38.64	0.00	C
ATOM	347	C	VAL	A	87	-4.355	0.547	21.630	38.64	0.00	C
ATOM	348	O	VAL	A	87	-3.230	0.067	21.819	38.64	0.00	O
ATOM	349	N	GLY	A	88	-5.321	0.484	22.542	47.04	0.00	N
ATOM	350	CA	GLY	A	88	-5.066	-0.115	23.865	47.04	0.00	C
ATOM	351	C	GLY	A	88	-4.192	0.803	24.708	47.04	0.00	C
ATOM	352	O	GLY	A	88	-3.216	0.373	25.351	47.04	0.00	O
ATOM	353	N	GLY	A	89	-4.538	2.089	24.663	51.00	0.00	N
ATOM	354	CA	GLY	A	89	-3.780	3.124	25.348	51.00	0.00	C
ATOM	355	C	GLY	A	89	-2.339	3.328	24.890	51.00	0.00	C
ATOM	356	O	GLY	A	89	-1.668	4.231	25.406	51.00	0.00	O
ATOM	357	N	LYS	A	90	-1.846	2.502	23.956	53.00	0.00	N
ATOM	358	CA	LYS	A	90	-0.547	2.733	23.318	53.00	0.00	C
ATOM	359	C	LYS	A	90	-0.830	3.651	22.138	53.00	0.00	C
ATOM	360	O	LYS	A	90	-1.881	3.541	21.497	53.00	0.00	O
ATOM	361	N	VAL	A	91	0.069	4.592	21.872	47.74	0.00	N
ATOM	362	CA	VAL	A	91	-0.106	5.503	20.744	47.74	0.00	C
ATOM	363	C	VAL	A	91	0.675	4.963	19.561	47.74	0.00	C
ATOM	364	O	VAL	A	91	1.817	4.523	19.727	47.74	0.00	O
ATOM	365	N	TYR	A	92	0.052	5.005	18.377	54.10	0.00	N
ATOM	366	CA	TYR	A	92	0.676	4.480	17.190	54.10	0.00	C
ATOM	367	C	TYR	A	92	0.645	5.490	16.087	54.10	0.00	C
ATOM	368	O	TYR	A	92	-0.329	6.191	15.893	54.10	0.00	O
ATOM	369	N	LYS	A	93	1.747	5.547	15.371	52.22	0.00	N
ATOM	370	CA	LYS	A	93	1.859	6.412	14.217	52.22	0.00	C
ATOM	371	C	LYS	A	93	1.524	5.603	12.955	52.22	0.00	C
ATOM	372	O	LYS	A	93	2.270	4.714	12.581	52.22	0.00	O
ATOM	373	N	ILE	A	94	0.406	5.927	12.320	51.93	0.00	N
ATOM	374	CA	ILE	A	94	0.084	5.372	11.038	51.93	0.00	C
ATOM	375	C	ILE	A	94	0.743	6.184	9.929	51.93	0.00	C
ATOM	376	O	ILE	A	94	0.556	7.385	9.807	51.93	0.00	O
ATOM	377	N	LYS	A	95	1.497	5.507	9.097	70.68	0.00	N
ATOM	378	CA	LYS	A	95	2.305	6.166	8.106	70.68	0.00	C
ATOM	379	C	LYS	A	95	1.950	5.702	6.699	70.68	0.00	C
ATOM	380	O	LYS	A	95	1.760	4.545	6.470	70.68	0.00	O
ATOM	381	N	GLY	A	96	1.853	6.613	5.743	49.96	0.00	N
ATOM	382	CA	GLY	A	96	1.572	6.242	4.383	49.96	0.00	C
ATOM	383	C	GLY	A	96	2.142	7.179	3.378	49.96	0.00	C
ATOM	384	O	GLY	A	96	2.537	8.280	3.703	49.96	0.00	O
ATOM	385	N	ARG	A	97	2.107	6.740	2.138	30.60	0.00	N
ATOM	386	CA	ARG	A	97	2.693	7.428	1.007	30.60	0.00	C
ATOM	387	C	ARG	A	97	1.769	7.206	-0.178	30.60	0.00	C
ATOM	388	O	ARG	A	97	1.672	6.084	-0.699	30.60	0.00	O
ATOM	389	N	ALA	A	98	1.104	8.281	-0.588	43.89	0.00	N
ATOM	390	CA	ALA	A	98	0.117	8.243	-1.592	43.89	0.00	C
ATOM	391	C	ALA	A	98	0.862	8.383	-2.906	43.89	0.00	C
ATOM	392	O	ALA	A	98	1.855	9.097	-3.010	43.89	0.00	O
ATOM	393	N	ASP	A	99	0.424	7.673	-3.912	66.23	0.00	N
ATOM	394	CA	ASP	A	99	1.076	7.754	-5.196	66.23	0.00	C
ATOM	395	C	ASP	A	99	0.974	9.188	-5.720	66.23	0.00	C
ATOM	396	O	ASP	A	99	1.922	9.707	-6.350	66.23	0.00	O
ATOM	397	N	ALA	A	100	-0.166	9.846	-5.513	61.99	0.00	N
ATOM	398	CA	ALA	A	100	-0.251	11.269	-5.953	61.99	0.00	C
ATOM	399	C	ALA	A	100	-1.290	11.992	-5.184	61.99	0.00	C
ATOM	400	O	ALA	A	100	-2.232	11.366	-4.731	61.99	0.00	O

ATOM	401	N	ILE	A	101	-1.104	13.303	-4.998	70.75	0.00	N
ATOM	402	CA	ILE	A	101	-2.137	14.137	-4.384	70.75	0.00	C
ATOM	403	C	ILE	A	101	-2.390	15.420	-5.140	70.75	0.00	C
ATOM	404	O	ILE	A	101	-1.467	16.143	-5.558	70.75	0.00	O
ATOM	405	N	ILE	A	102	-3.672	15.695	-5.329	38.77	0.00	N
ATOM	406	CA	ILE	A	102	-4.083	17.004	-5.796	38.77	0.00	C
ATOM	407	C	ILE	A	102	-4.628	17.765	-4.581	38.77	0.00	C
ATOM	408	O	ILE	A	102	-5.695	17.431	-4.073	38.77	0.00	O
ATOM	409	N	ARG	A	103	-3.845	18.751	-4.103	47.14	0.00	N
ATOM	410	CA	ARG	A	103	-4.150	19.504	-2.879	47.14	0.00	C
ATOM	411	C	ARG	A	103	-5.423	20.391	-2.949	47.14	0.00	C
ATOM	412	O	ARG	A	103	-6.199	20.482	-1.960	47.14	0.00	O
ATOM	413	N	ASN	A	104	-5.626	21.051	-4.095	50.55	0.00	N
ATOM	414	CA	ASN	A	104	-6.745	21.998	-4.259	50.55	0.00	C
ATOM	415	C	ASN	A	104	-7.230	21.994	-5.679	50.55	0.00	C
ATOM	416	O	ASN	A	104	-6.693	22.692	-6.538	50.55	0.00	O
ATOM	417	N	ASP	A	105	-8.253	21.174	-5.879	44.17	0.00	N
ATOM	418	CA	ASP	A	105	-9.039	21.134	-7.084	44.17	0.00	C
ATOM	419	C	ASP	A	105	-10.471	21.605	-6.714	44.17	0.00	C
ATOM	420	O	ASP	A	105	-11.262	20.843	-6.158	44.17	0.00	O
ATOM	421	N	ASN	A	106	-10.768	22.878	-6.986	47.42	0.00	N
ATOM	422	CA	ASN	A	106	-12.040	23.506	-6.575	47.42	0.00	C
ATOM	423	C	ASN	A	106	-12.422	23.321	-5.104	47.42	0.00	C
ATOM	424	O	ASN	A	106	-13.561	23.020	-4.774	47.42	0.00	O
ATOM	425	N	GLY	A	107	-11.470	23.524	-4.215	50.23	0.00	N
ATOM	426	CA	GLY	A	107	-11.717	23.337	-2.789	50.23	0.00	C
ATOM	427	C	GLY	A	107	-11.737	21.883	-2.335	50.23	0.00	C
ATOM	428	O	GLY	A	107	-12.404	21.544	-1.352	50.23	0.00	O
ATOM	429	N	LYS	A	108	-11.008	21.019	-3.043	19.82	0.00	N
ATOM	430	CA	LYS	A	108	-10.915	19.615	-2.668	19.82	0.00	C
ATOM	431	C	LYS	A	108	-9.483	19.049	-2.884	19.82	0.00	C
ATOM	432	O	LYS	A	108	-8.779	19.403	-3.839	19.82	0.00	O
ATOM	433	N	SER	A	109	-9.039	18.236	-1.933	46.23	0.00	N
ATOM	434	CA	SER	A	109	-7.862	17.387	-2.118	46.23	0.00	C
ATOM	435	C	SER	A	109	-8.308	16.067	-2.739	46.23	0.00	C
ATOM	436	O	SER	A	109	-9.315	15.517	-2.353	46.23	0.00	O
ATOM	437	N	ILE	A	110	-7.524	15.551	-3.650	49.84	0.00	N
ATOM	438	CA	ILE	A	110	-7.790	14.265	-4.209	49.84	0.00	C
ATOM	439	C	ILE	A	110	-6.588	13.343	-3.999	49.84	0.00	C
ATOM	440	O	ILE	A	110	-5.512	13.629	-4.510	49.84	0.00	O
ATOM	441	N	VAL	A	111	-6.752	12.243	-3.266	62.91	0.00	N
ATOM	442	CA	VAL	A	111	-5.679	11.220	-3.198	62.91	0.00	C
ATOM	443	C	VAL	A	111	-5.836	10.276	-4.388	62.91	0.00	C
ATOM	444	O	VAL	A	111	-6.932	9.764	-4.636	62.91	0.00	O
ATOM	445	N	ILE	A	112	-4.775	10.018	-5.128	58.29	0.00	N
ATOM	446	CA	ILE	A	112	-4.866	9.126	-6.282	58.29	0.00	C
ATOM	447	C	ILE	A	112	-4.010	7.894	-6.056	58.29	0.00	C
ATOM	448	O	ILE	A	112	-2.842	8.019	-5.770	58.29	0.00	O
ATOM	449	N	GLU	A	113	-4.547	6.709	-6.243	83.80	0.00	N
ATOM	450	CA	GLU	A	113	-3.780	5.455	-6.060	83.80	0.00	C
ATOM	451	C	GLU	A	113	-3.823	4.710	-7.388	83.80	0.00	C
ATOM	452	O	GLU	A	113	-4.882	4.569	-8.052	83.80	0.00	O
ATOM	453	N	ILE	A	114	-2.657	4.354	-7.861	47.53	0.00	N
ATOM	454	CA	ILE	A	114	-2.531	3.758	-9.150	47.53	0.00	C
ATOM	455	C	ILE	A	114	-2.210	2.295	-8.960	47.53	0.00	C
ATOM	456	O	ILE	A	114	-1.423	1.954	-8.099	47.53	0.00	O
ATOM	457	N	LYS	A	115	-2.787	1.433	-9.786	61.71	0.00	N
ATOM	458	CA	LYS	A	115	-2.621	0.003	-9.616	61.71	0.00	C
ATOM	459	C	LYS	A	115	-2.438	-0.643	-10.947	61.71	0.00	C
ATOM	460	O	LYS	A	115	-2.964	-0.176	-11.951	61.71	0.00	O
ATOM	461	N	THR	A	116	-1.684	-1.729	-10.958	35.95	0.00	N
ATOM	462	CA	THR	A	116	-1.468	-2.466	-12.194	35.95	0.00	C
ATOM	463	C	THR	A	116	-1.602	-3.921	-11.890	35.95	0.00	C
ATOM	464	O	THR	A	116	-1.369	-4.337	-10.804	35.95	0.00	O
ATOM	465	N	SER	A	117	-2.085	-4.706	-12.802	31.53	0.00	N
ATOM	466	CA	SER	A	117	-2.109	-6.154	-12.601	31.53	0.00	C
ATOM	467	C	SER	A	117	-2.180	-6.692	-13.946	31.53	0.00	C
ATOM	468	O	SER	A	117	-2.324	-5.911	-14.909	31.53	0.00	O
ATOM	469	N	ARG	A	118	-2.032	-8.006	-14.033	55.43	0.00	N
ATOM	470	CA	ARG	A	118	-2.068	-8.640	-15.363	55.43	0.00	C
ATOM	471	C	ARG	A	118	-3.471	-8.928	-15.862	55.43	0.00	C
ATOM	472	O	ARG	A	118	-3.648	-9.162	-17.069	55.43	0.00	O
ATOM	473	N	SER	A	119	-4.457	-8.893	-14.965	29.24	0.00	N
ATOM	474	CA	SER	A	119	-5.872	-9.062	-15.362	29.24	0.00	C
ATOM	475	C	SER	A	119	-6.800	-7.967	-14.770	29.24	0.00	C
ATOM	476	O	SER	A	119	-6.533	-7.439	-13.690	29.24	0.00	O
ATOM	477	N	ASP	A	120	-7.938	-7.766	-15.424	36.70	0.00	N
ATOM	478	CA	ASP	A	120	-8.922	-6.789	-15.023	36.70	0.00	C
ATOM	479	C	ASP	A	120	-10.062	-7.464	-14.339	36.70	0.00	C
ATOM	480	O	ASP	A	120	-11.180	-6.930	-14.286	36.70	0.00	O
ATOM	481	N	LYS	A	121	-9.805	-8.622	-13.800	43.60	0.00	N
ATOM	482	CA	LYS	A	121	-10.861	-9.343	-13.147	43.60	0.00	C
ATOM	483	C	LYS	A	121	-11.305	-8.719	-11.850	43.60	0.00	C
ATOM	484	O	LYS	A	121	-10.493	-8.415	-10.994	43.60	0.00	O
ATOM	485	N	GLY	A	122	-12.595	-8.552	-11.661	49.91	0.00	N
ATOM	486	CA	GLY	A	122	-13.099	-8.150	-10.341	49.91	0.00	C
ATOM	487	C	GLY	A	122	-12.816	-6.695	-9.897	49.91	0.00	C
ATOM	488	O	GLY	A	122	-13.062	-6.363	-8.702	49.91	0.00	O
ATOM	489	N	LEU	A	123	-12.369	-5.819	-10.826	46.62	0.00	N
ATOM	490	CA	LEU	A	123	-12.047	-4.418	-10.440	46.62	0.00	C
ATOM	491	C	LEU	A	123	-13.261	-3.672	-9.972	46.62	0.00	C
ATOM	492	O	LEU	A	123	-14.252	-3.694	-10.654	46.62	0.00	O

ATOM	493	N	PRO	A	124	-13.153	-2.951	-8.838	73.97	0.00	N
ATOM	494	CA	PRO	A	124	-11.936	-2.786	-8.048	73.97	0.00	C
ATOM	495	C	PRO	A	124	-11.854	-3.776	-6.934	73.97	0.00	C
ATOM	496	O	PRO	A	124	-12.861	-4.122	-6.314	73.97	0.00	O
ATOM	497	N	LEU	A	125	-10.645	-4.184	-6.634	23.98	0.00	N
ATOM	498	CA	LEU	A	125	-10.394	-5.171	-5.542	23.98	0.00	C
ATOM	499	C	LEU	A	125	-10.497	-4.530	-4.149	23.98	0.00	C
ATOM	500	O	LEU	A	125	-10.112	-3.350	-3.908	23.98	0.00	O
ATOM	501	N	ILE	A	126	-11.048	-5.276	-3.221	38.08	0.00	N
ATOM	502	CA	ILE	A	126	-11.528	-4.647	-1.996	38.08	0.00	C
ATOM	503	C	ILE	A	126	-10.339	-4.126	-1.167	38.08	0.00	C
ATOM	504	O	ILE	A	126	-10.462	-3.123	-0.513	38.08	0.00	O
ATOM	505	N	HIS	A	127	-9.195	-4.779	-1.251	29.29	0.00	N
ATOM	506	CA	HIS	A	127	-8.055	-4.383	-0.479	29.29	0.00	C
ATOM	507	C	HIS	A	127	-7.440	-3.163	-1.103	29.29	0.00	C
ATOM	508	O	HIS	A	127	-6.834	-2.382	-0.415	29.29	0.00	O
ATOM	509	N	HIS	A	128	-7.592	-2.980	-2.400	39.69	0.00	N
ATOM	510	CA	HIS	A	128	-7.176	-1.718	-3.018	39.69	0.00	C
ATOM	511	C	HIS	A	128	-8.083	-0.558	-2.609	39.69	0.00	C
ATOM	512	O	HIS	A	128	-7.606	0.554	-2.368	39.69	0.00	O
ATOM	513	N	LYS	A	129	-9.365	-0.803	-2.526	27.03	0.00	N
ATOM	514	CA	LYS	A	129	-10.266	0.264	-2.198	27.03	0.00	C
ATOM	515	C	LYS	A	129	-10.028	0.593	-0.745	27.03	0.00	C
ATOM	516	O	LYS	A	129	-9.930	1.747	-0.408	27.03	0.00	O
ATOM	517	N	MSE	A	130	-9.911	-0.410	0.128	40.21	0.00	N
ATOM	518	CA	MSE	A	130	-9.661	-0.122	1.564	40.21	0.00	C
ATOM	519	C	MSE	A	130	-8.432	0.772	1.739	40.21	0.00	C
ATOM	520	O	MSE	A	130	-8.445	1.649	2.550	40.21	0.00	O
ATOM	521	N	GLN	A	131	-7.394	0.520	0.958	46.26	0.00	N
ATOM	522	CA	GLN	A	131	-6.133	1.211	1.097	46.26	0.00	C
ATOM	523	C	GLN	A	131	-6.400	2.668	0.780	46.26	0.00	C
ATOM	524	O	GLN	A	131	-5.918	3.542	1.460	46.26	0.00	O
ATOM	525	N	LEU	A	132	-7.195	2.936	-0.242	48.70	0.00	N
ATOM	526	CA	LEU	A	132	-7.436	4.290	-0.682	48.70	0.00	C
ATOM	527	C	LEU	A	132	-8.295	4.984	0.347	48.70	0.00	C
ATOM	528	O	LEU	A	132	-8.142	6.173	0.629	48.70	0.00	O
ATOM	529	N	GLN	A	133	-9.173	4.244	0.963	49.66	0.00	N
ATOM	530	CA	GLN	A	133	-10.034	4.835	1.951	49.66	0.00	C
ATOM	531	C	GLN	A	133	-9.306	5.181	3.263	49.66	0.00	C
ATOM	532	O	GLN	A	133	-9.739	6.050	4.021	49.66	0.00	O
ATOM	533	N	ILE	A	134	-8.240	4.450	3.561	54.32	0.00	N
ATOM	534	CA	ILE	A	134	-7.448	4.750	4.709	54.32	0.00	C
ATOM	535	C	ILE	A	134	-6.635	6.008	4.375	54.32	0.00	C
ATOM	536	O	ILE	A	134	-6.604	6.904	5.182	54.32	0.00	O
ATOM	537	N	TYR	A	135	-5.993	6.089	3.200	55.17	0.00	N
ATOM	538	CA	TYR	A	135	-5.308	7.299	2.832	55.17	0.00	C
ATOM	539	C	TYR	A	135	-6.263	8.486	3.003	55.17	0.00	C
ATOM	540	O	TYR	A	135	-5.876	9.536	3.592	55.17	0.00	O
ATOM	541	N	LEU	A	136	-7.510	8.345	2.563	51.61	0.00	N
ATOM	542	CA	LEU	A	136	-8.431	9.472	2.663	51.61	0.00	C
ATOM	543	C	LEU	A	136	-8.471	9.988	4.123	51.61	0.00	C
ATOM	544	O	LEU	A	136	-8.423	11.193	4.364	51.61	0.00	O
ATOM	545	N	TRP	A	137	-8.562	9.085	5.079	40.90	0.00	N
ATOM	546	CA	TRP	A	137	-8.471	9.465	6.480	40.90	0.00	C
ATOM	547	C	TRP	A	137	-7.090	10.092	6.781	40.90	0.00	C
ATOM	548	O	TRP	A	137	-6.988	11.182	7.363	40.90	0.00	O
ATOM	549	N	LEU	A	138	-6.039	9.457	6.283	23.53	0.00	N
ATOM	550	CA	LEU	A	138	-4.665	9.881	6.616	23.53	0.00	C
ATOM	551	C	LEU	A	138	-4.353	11.332	6.246	23.53	0.00	C
ATOM	552	O	LEU	A	138	-3.806	12.080	7.039	23.53	0.00	O
ATOM	553	N	PHE	A	139	-4.725	11.691	5.030	31.22	0.00	N
ATOM	554	CA	PHE	A	139	-4.602	13.020	4.511	31.22	0.00	C
ATOM	555	C	PHE	A	139	-5.790	13.890	4.834	31.22	0.00	C
ATOM	556	O	PHE	A	139	-5.772	15.074	4.508	31.22	0.00	O
ATOM	557	N	SER	A	140	-6.827	13.321	5.474	30.40	0.00	N
ATOM	558	CA	SER	A	140	-8.140	13.999	5.608	30.40	0.00	C
ATOM	559	C	SER	A	140	-8.590	14.636	4.304	30.40	0.00	C
ATOM	560	O	SER	A	140	-8.961	15.800	4.273	30.40	0.00	O
ATOM	561	N	ALA	A	141	-8.519	13.878	3.227	38.58	0.00	N
ATOM	562	CA	ALA	A	141	-8.945	14.332	1.917	38.58	0.00	C
ATOM	563	C	ALA	A	141	-10.405	13.939	1.667	38.58	0.00	C
ATOM	564	O	ALA	A	141	-10.983	13.084	2.348	38.58	0.00	O
ATOM	565	N	GLU	A	142	-11.035	14.621	0.722	0.00	0.00	N
ATOM	566	CA	GLU	A	142	-12.471	14.427	0.489	0.00	0.00	C
ATOM	567	C	GLU	A	142	-12.738	13.477	-0.702	0.00	0.00	C
ATOM	568	O	GLU	A	142	-13.747	12.791	-0.707	0.00	0.00	O
ATOM	569	N	LYS	A	143	-11.805	13.444	-1.660	66.08	0.00	N
ATOM	570	CA	LYS	A	143	-11.916	12.652	-2.862	66.08	0.00	C
ATOM	571	C	LYS	A	143	-10.727	11.686	-3.055	66.08	0.00	C
ATOM	572	O	LYS	A	143	-9.539	11.991	-2.732	66.08	0.00	O
ATOM	573	N	GLY	A	144	-11.077	10.526	-3.602	60.83	0.00	N
ATOM	574	CA	GLY	A	144	-10.120	9.474	-3.925	60.83	0.00	C
ATOM	575	C	GLY	A	144	-10.352	8.924	-5.300	60.83	0.00	C
ATOM	576	O	GLY	A	144	-11.486	8.740	-5.697	60.83	0.00	O
ATOM	577	N	ILE	A	145	-9.286	8.679	-6.041	72.37	0.00	N
ATOM	578	CA	ILE	A	145	-9.380	8.016	-7.325	72.37	0.00	C
ATOM	579	C	ILE	A	145	-8.437	6.791	-7.270	72.37	0.00	C
ATOM	580	O	ILE	A	145	-7.274	6.920	-6.924	72.37	0.00	O
ATOM	581	N	LEU	A	146	-8.955	5.621	-7.649	57.82	0.00	N
ATOM	582	CA	LEU	A	146	-8.233	4.396	-7.797	57.82	0.00	C
ATOM	583	C	LEU	A	146	-8.251	4.098	-9.279	57.82	0.00	C
ATOM	584	O	LEU	A	146	-9.304	3.843	-9.816	57.82	0.00	O

```
ATOM    585  N   VAL A 147      -7.117   4.065  -9.937 49.59  0.00           N
ATOM    586  CA  VAL A 147      -7.059   3.872 -11.356 49.59  0.00           C
ATOM    587  C   VAL A 147      -6.211   2.623 -11.646 49.59  0.00           C
ATOM    588  O   VAL A 147      -5.067   2.470 -11.090 49.59  0.00           O
ATOM    589  N   TYR A 148      -6.742   1.739 -12.509 53.71  0.00           N
ATOM    590  CA  TYR A 148      -6.090   0.482 -12.844 53.71  0.00           C
ATOM    591  C   TYR A 148      -5.595   0.575 -14.257 53.71  0.00           C
ATOM    592  O   TYR A 148      -6.345   0.903 -15.117 53.71  0.00           O
ATOM    593  N   ILE A 149      -4.338   0.293 -14.483 38.41  0.00           N
ATOM    594  CA  ILE A 149      -3.766   0.206 -15.807 38.41  0.00           C
ATOM    595  C   ILE A 149      -3.430  -1.275 -15.990 38.41  0.00           C
ATOM    596  O   ILE A 149      -2.545  -1.776 -15.346 38.41  0.00           O
ATOM    597  N   THR A 150      -4.180  -2.003 -16.800 57.09  0.00           N
ATOM    598  CA  THR A 150      -3.964  -3.413 -16.968 57.09  0.00           C
ATOM    599  C   THR A 150      -3.936  -3.690 -18.433 57.09  0.00           C
ATOM    600  O   THR A 150      -4.311  -2.846 -19.222 57.09  0.00           O
ATOM    601  N   PRO A 151      -3.486  -4.859 -18.836 48.53  0.00           N
ATOM    602  CA  PRO A 151      -3.299  -5.051 -20.300 48.53  0.00           C
ATOM    603  C   PRO A 151      -4.601  -5.273 -21.077 48.53  0.00           C
ATOM    604  O   PRO A 151      -4.598  -5.259 -22.317 48.53  0.00           O
ATOM    605  N   ASP A 152      -5.658  -5.505 -20.333 47.48  0.00           N
ATOM    606  CA  ASP A 152      -6.988  -5.753 -20.794 47.48  0.00           C
ATOM    607  C   ASP A 152      -7.910  -4.564 -20.707 47.48  0.00           C
ATOM    608  O   ASP A 152      -8.921  -4.561 -21.326 47.48  0.00           O
ATOM    609  N   ARG A 153      -7.550  -3.582 -19.909 75.55  0.00           N
ATOM    610  CA  ARG A 153      -8.418  -2.528 -19.517 75.55  0.00           C
ATOM    611  C   ARG A 153      -7.808  -1.437 -18.658 75.55  0.00           C
ATOM    612  O   ARG A 153      -7.169  -1.665 -17.715 75.55  0.00           O
ATOM    613  N   ILE A 154      -8.107  -0.221 -18.973 47.28  0.00           N
ATOM    614  CA  ILE A 154      -7.873   0.874 -18.098 47.28  0.00           C
ATOM    615  C   ILE A 154      -9.177   1.197 -17.432 47.28  0.00           C
ATOM    616  O   ILE A 154     -10.135   1.182 -18.066 47.28  0.00           O
ATOM    617  N   ALA A 155      -9.204   1.463 -16.150 49.85  0.00           N
ATOM    618  CA  ALA A 155     -10.490   1.563 -15.403 49.85  0.00           C
ATOM    619  C   ALA A 155     -10.307   2.411 -14.178 49.85  0.00           C
ATOM    620  O   ALA A 155      -9.405   2.165 -13.464 49.85  0.00           O
ATOM    621  N   GLU A 156     -11.159   3.389 -13.945 57.54  0.00           N
ATOM    622  CA  GLU A 156     -10.959   4.392 -12.924 57.54  0.00           C
ATOM    623  C   GLU A 156     -12.193   4.455 -12.023 57.54  0.00           C
ATOM    624  O   GLU A 156     -13.299   4.455 -12.446 57.54  0.00           O
ATOM    625  N   TYR A 157     -11.976   4.532 -10.746 56.62  0.00           N
ATOM    626  CA  TYR A 157     -13.044   4.493  -9.764 56.62  0.00           C
ATOM    627  C   TYR A 157     -12.934   5.658  -8.804 56.62  0.00           C
ATOM    628  O   TYR A 157     -11.853   6.011  -8.265 56.62  0.00           O
ATOM    629  N   GLU A 158     -14.055   6.253  -8.542 49.97  0.00           N
ATOM    630  CA  GLU A 158     -14.108   7.361  -7.646 49.97  0.00           C
ATOM    631  C   GLU A 158     -14.532   6.786  -6.302 49.97  0.00           C
ATOM    632  O   GLU A 158     -15.602   6.184  -6.178 49.97  0.00           O
ATOM    633  N   ILE A 159     -13.651   6.902  -5.311 52.35  0.00           N
ATOM    634  CA  ILE A 159     -13.878   6.419  -3.939 52.35  0.00           C
ATOM    635  C   ILE A 159     -13.755   7.649  -3.027 52.35  0.00           C
ATOM    636  O   ILE A 159     -12.671   8.212  -2.918 52.35  0.00           O
ATOM    637  N   ASN A 160     -14.849   8.038  -2.394 52.16  0.00           N
ATOM    638  CA  ASN A 160     -14.894   9.274  -1.638 52.16  0.00           C
ATOM    639  C   ASN A 160     -15.165   9.116  -0.146 52.16  0.00           C
ATOM    640  O   ASN A 160     -14.949  10.056   0.594 52.16  0.00           O
ATOM    641  N   GLU A 161     -15.536   7.926   0.306 24.33  0.00           N
ATOM    642  CA  GLU A 161     -15.720   7.704   1.724 24.33  0.00           C
ATOM    643  C   GLU A 161     -14.467   7.151   2.407 24.33  0.00           C
ATOM    644  O   GLU A 161     -13.997   6.052   2.116 24.33  0.00           O
ATOM    645  N   PRO A 162     -13.950   7.900   3.365 48.62  0.00           N
ATOM    646  CA  PRO A 162     -12.848   7.426   4.187 48.62  0.00           C
ATOM    647  C   PRO A 162     -13.323   6.439   5.227 48.62  0.00           C
ATOM    648  O   PRO A 162     -14.483   6.537   5.645 48.62  0.00           O
ATOM    649  N   LEU A 163     -12.444   5.517   5.661 50.64  0.00           N
ATOM    650  CA  LEU A 163     -12.805   4.572   6.724 50.64  0.00           C
ATOM    651  C   LEU A 163     -12.888   5.346   8.019 50.64  0.00           C
ATOM    652  O   LEU A 163     -12.040   6.223   8.266 50.64  0.00           O
ATOM    653  N   ASP A 164     -13.876   5.013   8.858 13.03  0.00           N
ATOM    654  CA  ASP A 164     -13.934   5.590  10.207 13.03  0.00           C
ATOM    655  C   ASP A 164     -12.616   5.280  10.928 13.03  0.00           C
ATOM    656  O   ASP A 164     -11.940   4.300  10.622 13.03  0.00           O
ATOM    657  N   GLU A 165     -12.301   6.090  11.926 52.58  0.00           N
ATOM    658  CA  GLU A 165     -11.127   5.880  12.758 52.58  0.00           C
ATOM    659  C   GLU A 165     -11.240   4.594  13.565 52.58  0.00           C
ATOM    660  O   GLU A 165     -10.265   3.923  13.761 52.58  0.00           O
ATOM    661  N   ALA A 166     -12.438   4.252  14.016 52.45  0.00           N
ATOM    662  CA  ALA A 166     -12.662   3.006  14.753 52.45  0.00           C
ATOM    663  C   ALA A 166     -12.336   1.750  13.921 52.45  0.00           C
ATOM    664  O   ALA A 166     -11.761   0.808  14.415 52.45  0.00           O
ATOM    665  N   THR A 167     -12.694   1.751  12.648 22.42  0.00           N
ATOM    666  CA  THR A 167     -12.382   0.638  11.768 22.42  0.00           C
ATOM    667  C   THR A 167     -10.872   0.497  11.623 22.42  0.00           C
ATOM    668  O   THR A 167     -10.346  -0.601  11.637 22.42  0.00           O
ATOM    669  N   ILE A 168     -10.171   1.619  11.528 46.72  0.00           N
ATOM    670  CA  ILE A 168      -8.732   1.633  11.358 46.72  0.00           C
ATOM    671  C   ILE A 168      -8.078   1.128  12.626 46.72  0.00           C
ATOM    672  O   ILE A 168      -7.058   0.457  12.570 46.72  0.00           O
ATOM    673  N   VAL A 169      -8.665   1.441  13.778 48.67  0.00           N
ATOM    674  CA  VAL A 169      -8.132   0.957  15.026 48.67  0.00           C
ATOM    675  C   VAL A 169      -8.314  -0.550  15.039 48.67  0.00           C
ATOM    676  O   VAL A 169      -7.394  -1.257  15.440 48.67  0.00           O
```

ATOM	677	N	ARG	A	170	-9.472	-1.045	14.590	4.85	0.00	N
ATOM	678	CA	ARG	A	170	-9.700	-2.518	14.528	4.85	0.00	C
ATOM	679	C	ARG	A	170	-8.661	-3.202	13.604	4.85	0.00	C
ATOM	680	O	ARG	A	170	-8.069	-4.207	13.996	4.85	0.00	O
ATOM	681	N	LEU	A	171	-8.396	-2.610	12.430	35.06	0.00	N
ATOM	682	CA	LEU	A	171	-7.379	-3.127	11.527	35.06	0.00	C
ATOM	683	C	LEU	A	171	-6.020	-3.139	12.210	35.06	0.00	C
ATOM	684	O	LEU	A	171	-5.257	-4.108	12.109	35.06	0.00	O
ATOM	685	N	ALA	A	172	-5.723	-2.049	12.909	35.22	0.00	N
ATOM	686	CA	ALA	A	172	-4.457	-1.898	13.604	35.22	0.00	C
ATOM	687	C	ALA	A	172	-4.301	-2.947	14.696	35.22	0.00	C
ATOM	688	O	ALA	A	172	-3.240	-3.541	14.801	35.22	0.00	O
ATOM	689	N	GLU	A	173	-5.352	-3.187	15.479	57.67	0.00	N
ATOM	690	CA	GLU	A	173	-5.292	-4.177	16.568	57.67	0.00	C
ATOM	691	C	GLU	A	173	-4.915	-5.559	16.053	57.67	0.00	C
ATOM	692	O	GLU	A	173	-4.131	-6.255	16.707	57.67	0.00	O
ATOM	693	N	ASP	A	174	-5.506	-5.961	14.919	41.81	0.00	N
ATOM	694	CA	ASP	A	174	-5.208	-7.255	14.320	41.81	0.00	C
ATOM	695	C	ASP	A	174	-3.815	-7.301	13.840	41.81	0.00	C
ATOM	696	O	ASP	A	174	-3.169	-8.343	13.873	41.81	0.00	O
ATOM	697	N	THR	A	175	-3.325	-6.165	13.384	37.60	0.00	N
ATOM	698	CA	THR	A	175	-1.966	-6.171	12.887	37.60	0.00	C
ATOM	699	C	THR	A	175	-1.026	-6.460	14.019	37.60	0.00	C
ATOM	700	O	THR	A	175	-0.104	-7.267	13.894	37.60	0.00	O
ATOM	701	N	ILE	A	176	-1.312	-5.827	15.139	46.10	0.00	N
ATOM	702	CA	ILE	A	176	-0.456	-5.907	16.306	46.10	0.00	C
ATOM	703	C	ILE	A	176	-0.618	-7.264	17.013	46.10	0.00	C
ATOM	704	O	ILE	A	176	0.348	-7.982	17.191	46.10	0.00	O
ATOM	705	N	MSE	A	177	-1.846	-7.622	17.359	53.53	0.00	N
ATOM	706	CA	MSE	A	177	-2.110	-8.862	18.066	53.53	0.00	C
ATOM	707	C	MSE	A	177	-2.036	-10.148	17.184	53.53	0.00	C
ATOM	708	O	MSE	A	177	-2.230	-11.232	17.705	53.53	0.00	O
ATOM	709	N	LEU	A	178	-1.747	-10.045	15.885	54.52	0.00	N
ATOM	710	CA	LEU	A	178	-1.765	-11.195	14.931	54.52	0.00	C
ATOM	711	C	LEU	A	178	-3.082	-11.987	14.963	54.52	0.00	C
ATOM	712	O	LEU	A	178	-3.059	-13.216	14.908	54.52	0.00	O
ATOM	713	N	GLN	A	179	-4.195	-11.295	14.914	9.65	0.00	N
ATOM	714	CA	GLN	A	179	-5.488	-11.880	15.107	9.65	0.00	C
ATOM	715	C	GLN	A	179	-6.016	-12.818	14.022	9.65	0.00	C
ATOM	716	O	GLN	A	179	-6.283	-13.971	14.291	9.65	0.00	O
ATOM	717	N	ASN	A	180	-6.209	-12.312	12.822	36.56	0.00	N
ATOM	718	CA	ASN	A	180	-6.832	-13.100	11.723	36.56	0.00	C
ATOM	719	C	ASN	A	180	-5.958	-13.170	10.495	36.56	0.00	C
ATOM	720	O	ASN	A	180	-5.982	-12.302	9.632	36.56	0.00	O
ATOM	721	N	SER	A	181	-5.162	-14.213	10.431	54.59	0.00	N
ATOM	722	CA	SER	A	181	-4.294	-14.421	9.316	54.59	0.00	C
ATOM	723	C	SER	A	181	-4.685	-15.651	8.495	54.59	0.00	C
ATOM	724	O	SER	A	181	-4.793	-16.745	9.008	54.59	0.00	O
ATOM	725	N	PRO	A	182	-4.770	-15.497	7.199	58.07	0.00	N
ATOM	726	CA	PRO	A	182	-4.417	-14.305	6.461	58.07	0.00	C
ATOM	727	C	PRO	A	182	-5.557	-13.337	6.500	58.07	0.00	C
ATOM	728	O	PRO	A	182	-6.623	-13.675	7.004	58.07	0.00	O
ATOM	729	N	ARG	A	183	-5.357	-12.135	5.998	45.05	0.00	N
ATOM	730	CA	ARG	A	183	-6.436	-11.191	6.007	45.05	0.00	C
ATOM	731	C	ARG	A	183	-7.372	-11.579	4.893	45.05	0.00	C
ATOM	732	O	ARG	A	183	-8.603	-11.628	5.107	45.05	0.00	O
ATOM	733	N	PHE	A	184	-6.785	-11.851	3.718	40.86	0.00	N
ATOM	734	CA	PHE	A	184	-7.550	-12.410	2.590	40.86	0.00	C
ATOM	735	C	PHE	A	184	-7.017	-13.778	2.185	40.86	0.00	C
ATOM	736	O	PHE	A	184	-5.814	-14.039	2.233	40.86	0.00	O
ATOM	737	N	ASN	A	185	-7.926	-14.630	1.714	41.19	0.00	N
ATOM	738	CA	ASN	A	185	-7.604	-16.007	1.378	41.19	0.00	C
ATOM	739	C	ASN	A	185	-6.563	-16.229	0.291	41.19	0.00	C
ATOM	740	O	ASN	A	185	-5.857	-17.232	0.316	41.19	0.00	O
ATOM	741	N	TRP	A	186	-6.507	-15.349	-0.690	25.29	0.00	N
ATOM	742	CA	TRP	A	186	-5.602	-15.511	-1.821	25.29	0.00	C
ATOM	743	C	TRP	A	186	-4.124	-15.190	-1.442	25.29	0.00	C
ATOM	744	O	TRP	A	186	-3.168	-15.441	-2.207	25.29	0.00	O
ATOM	745	N	GLU	A	187	-3.932	-14.615	-0.259	93.17	0.00	N
ATOM	746	CA	GLU	A	187	-2.575	-14.179	0.176	93.17	0.00	C
ATOM	747	C	GLU	A	187	-1.511	-15.229	0.225	93.17	0.00	C
ATOM	748	O	GLU	A	187	-0.428	-15.028	-0.334	93.17	0.00	O
ATOM	749	N	CYS	A	188	-1.801	-16.352	0.884	43.08	0.00	N
ATOM	750	CA	CYS	A	188	-0.760	-17.344	1.180	43.08	0.00	C
ATOM	751	C	CYS	A	188	-0.146	-17.865	-0.127	43.08	0.00	C
ATOM	752	O	CYS	A	188	1.067	-18.120	-0.218	43.08	0.00	O
ATOM	753	N	LYS	A	189	-0.970	-17.963	-1.166	45.12	0.00	N
ATOM	754	CA	LYS	A	189	-0.511	-18.522	-2.447	45.12	0.00	C
ATOM	755	C	LYS	A	189	0.696	-17.731	-3.018	45.12	0.00	C
ATOM	756	O	LYS	A	189	1.569	-18.272	-3.724	45.12	0.00	O
ATOM	757	N	TYR	A	190	0.747	-16.439	-2.708	37.47	0.00	N
ATOM	758	CA	TYR	A	190	1.763	-15.611	-3.285	37.47	0.00	C
ATOM	759	C	TYR	A	190	2.819	-15.235	-2.248	37.47	0.00	C
ATOM	760	O	TYR	A	190	3.823	-14.654	-2.584	37.47	0.00	O
ATOM	761	N	CYS	A	191	2.587	-15.625	-0.997	32.14	0.00	N
ATOM	762	CA	CYS	A	191	3.353	-15.193	0.160	32.14	0.00	C
ATOM	763	C	CYS	A	191	4.692	-15.934	0.269	32.14	0.00	C
ATOM	764	O	CYS	A	191	4.732	-17.173	0.348	32.14	0.00	O
ATOM	765	N	ILE	A	192	5.784	-15.171	0.315	21.11	0.00	N
ATOM	766	CA	ILE	A	192	7.106	-15.776	0.279	21.11	0.00	C
ATOM	767	C	ILE	A	192	7.409	-16.403	1.615	21.11	0.00	C
ATOM	768	O	ILE	A	192	8.394	-17.068	1.711	21.11	0.00	O

```
ATOM    769  N   PHE A 193       6.578 -16.191   2.632 33.12  0.00           N
ATOM    770  CA  PHE A 193       6.786 -16.794   3.943 33.12  0.00           C
ATOM    771  C   PHE A 193       5.984 -18.074   4.210 33.12  0.00           C
ATOM    772  O   PHE A 193       5.990 -18.637   5.317 33.12  0.00           O
ATOM    773  N   SER A 194       5.262 -18.513   3.198 47.00  0.00           N
ATOM    774  CA  SER A 194       4.413 -19.711   3.328 47.00  0.00           C
ATOM    775  C   SER A 194       5.249 -20.909   3.657 47.00  0.00           C
ATOM    776  O   SER A 194       4.748 -21.746   4.298 47.00  0.00           O
ATOM    777  N   VAL A 195       6.503 -20.952   3.199 41.00  0.00           N
ATOM    778  CA  VAL A 195       7.410 -21.989   3.516 41.00  0.00           C
ATOM    779  C   VAL A 195       7.733 -22.174   4.979 41.00  0.00           C
ATOM    780  O   VAL A 195       8.309 -23.201   5.323 41.00  0.00           O
ATOM    781  N   ILE A 196       7.419 -21.212   5.849 42.60  0.00           N
ATOM    782  CA  ILE A 196       7.718 -21.354   7.295 42.60  0.00           C
ATOM    783  C   ILE A 196       6.564 -20.975   8.148 42.60  0.00           C
ATOM    784  O   ILE A 196       6.683 -20.972   9.331 42.60  0.00           O
ATOM    785  N   CYS A 197       5.424 -20.671   7.561 52.08  0.00           N
ATOM    786  CA  CYS A 197       4.335 -20.157   8.329 52.08  0.00           C
ATOM    787  C   CYS A 197       3.331 -21.219   8.615 52.08  0.00           C
ATOM    788  O   CYS A 197       2.836 -21.849   7.697 52.08  0.00           O
ATOM    789  N   PRO A 198       2.960 -21.375   9.870 46.10  0.00           N
ATOM    790  CA  PRO A 198       1.970 -22.338  10.280 46.10  0.00           C
ATOM    791  C   PRO A 198       0.542 -22.046   9.971 46.10  0.00           C
ATOM    792  O   PRO A 198      -0.290 -22.938  10.156 46.10  0.00           O
ATOM    793  N   ALA A 199       0.227 -20.829   9.562 29.17  0.00           N
ATOM    794  CA  ALA A 199      -1.167 -20.431   9.353 29.17  0.00           C
ATOM    795  C   ALA A 199      -1.570 -20.467   7.877 29.17  0.00           C
ATOM    796  O   ALA A 199      -2.731 -20.267   7.563 29.17  0.00           O
ATOM    797  N   LYS A 200      -0.591 -20.655   6.991 51.72  0.00           N
ATOM    798  CA  LYS A 200      -0.778 -20.823   5.548 51.72  0.00           C
ATOM    799  C   LYS A 200      -1.946 -21.672   5.236 51.72  0.00           C
ATOM    800  O   LYS A 200      -2.051 -22.735   5.769 51.72  0.00           O
ATOM    801  N   LEU A 201      -2.799 -21.218   4.328 46.35  0.00           N
ATOM    802  CA  LEU A 201      -3.935 -22.003   3.876 46.35  0.00           C
ATOM    803  C   LEU A 201      -3.511 -23.046   2.859 46.35  0.00           C
ATOM    804  O   LEU A 201      -2.649 -22.775   2.009 46.35  0.00           O
ATOM    805  N   THR A 202      -4.127 -24.225   2.953 44.58  0.00           N
ATOM    806  CA  THR A 202      -3.796 -25.369   2.082 44.58  0.00           C
ATOM    807  C   THR A 202      -5.038 -25.920   1.370 44.58  0.00           C
ATOM    808  O   THR A 202      -6.101 -25.964   1.999 44.58  0.00           O
TER     809      THR A 202
ENDMDL
END
REMARK   1 ***********************************************************************
REMARK   1 Start File NAT_vs_DEC4_dSi_colored.pdb
REMARK   1 The occupancy record was modified to represent dSi := Si(DEC)-Si(NAT).
REMARK   1 Occ=0.00 means dSi=-1.484741 kB; Occ=99.99 means dSi=1.546813 kB.
MODEL        0
ATOM      1  N   MSE A   1      23.268  -4.104 -18.521 48.49  0.00           N
ATOM      2  CA  MSE A   1      23.439  -2.722 -18.016 48.49  0.00           C
ATOM      3  C   MSE A   1      22.152  -2.161 -17.383 48.49  0.00           C
ATOM      4  O   MSE A   1      22.147  -1.650 -16.252 48.49  0.00           O
ATOM      5  N   ILE A   2      21.045  -2.254 -18.100 39.73  0.00           N
ATOM      6  CA  ILE A   2      19.816  -1.632 -17.608 39.73  0.00           C
ATOM      7  C   ILE A   2      19.269  -2.405 -16.412 39.73  0.00           C
ATOM      8  O   ILE A   2      18.935  -1.835 -15.391 39.73  0.00           O
ATOM      9  N   THR A   3      19.186  -3.703 -16.563 54.25  0.00           N
ATOM     10  CA  THR A   3      18.718  -4.606 -15.498 54.25  0.00           C
ATOM     11  C   THR A   3      19.478  -4.398 -14.212 54.25  0.00           C
ATOM     12  O   THR A   3      18.884  -4.188 -13.150 54.25  0.00           O
ATOM     13  N   GLU A   4      20.800  -4.447 -14.318 44.58  0.00           N
ATOM     14  CA  GLU A   4      21.687  -4.148 -13.195 44.58  0.00           C
ATOM     15  C   GLU A   4      21.342  -2.832 -12.484 44.58  0.00           C
ATOM     16  O   GLU A   4      21.232  -2.791 -11.242 44.58  0.00           O
ATOM     17  N   PHE A   5      21.133  -1.765 -13.247 52.03  0.00           N
ATOM     18  CA  PHE A   5      20.826  -0.486 -12.602 52.03  0.00           C
ATOM     19  C   PHE A   5      19.486  -0.520 -11.886 52.03  0.00           C
ATOM     20  O   PHE A   5      19.415  -0.122 -10.736 52.03  0.00           O
ATOM     21  N   LEU A   6      18.429  -1.039 -12.529 45.33  0.00           N
ATOM     22  CA  LEU A   6      17.100  -1.072 -11.873 45.33  0.00           C
ATOM     23  C   LEU A   6      17.119  -1.921 -10.586 45.33  0.00           C
ATOM     24  O   LEU A   6      16.551  -1.545  -9.531 45.33  0.00           O
ATOM     25  N   LEU A   7      17.767  -3.071 -10.672 34.02  0.00           N
ATOM     26  CA  LEU A   7      17.843  -3.929  -9.486 34.02  0.00           C
ATOM     27  C   LEU A   7      18.603  -3.241  -8.341 34.02  0.00           C
ATOM     28  O   LEU A   7      18.190  -3.292  -7.184 34.02  0.00           O
ATOM     29  N   LYS A   8      19.691  -2.566  -8.693 38.58  0.00           N
ATOM     30  CA  LYS A   8      20.463  -1.856  -7.680 38.58  0.00           C
ATOM     31  C   LYS A   8      19.628  -0.762  -7.044 38.58  0.00           C
ATOM     32  O   LYS A   8      19.527  -0.688  -5.819 38.58  0.00           O
ATOM     33  N   LYS A   9      18.947   0.036  -7.860 37.09  0.00           N
ATOM     34  CA  LYS A   9      18.144   1.113  -7.292 37.09  0.00           C
ATOM     35  C   LYS A   9      17.009   0.545  -6.471 37.09  0.00           C
ATOM     36  O   LYS A   9      16.708   1.086  -5.402 37.09  0.00           O
ATOM     37  N   LYS A  10      16.379  -0.547  -6.912 45.03  0.00           N
ATOM     38  CA  LYS A  10      15.262  -1.072  -6.116 45.03  0.00           C
ATOM     39  C   LYS A  10      15.660  -1.681  -4.726 45.03  0.00           C
ATOM     40  O   LYS A  10      14.931  -1.547  -3.695 45.03  0.00           O
ATOM     41  N   LEU A  11      16.798  -2.366  -4.699 27.27  0.00           N
ATOM     42  CA  LEU A  11      17.328  -2.879  -3.411 27.27  0.00           C
ATOM     43  C   LEU A  11      17.613  -1.686  -2.439 27.27  0.00           C
ATOM     44  O   LEU A  11      17.179  -1.698  -1.270 27.27  0.00           O
```

ATOM	45	N	GLU	A	12	18.299	-0.655	-2.945	25.13	0.00	N
ATOM	46	CA	GLU	A	12	18.475	0.605	-2.181	25.13	0.00	C
ATOM	47	C	GLU	A	12	17.179	1.213	-1.616	25.13	0.00	C
ATOM	48	O	GLU	A	12	17.048	1.390	-0.426	25.13	0.00	O
ATOM	49	N	GLU	A	13	16.225	1.536	-2.461	28.47	0.00	N
ATOM	50	CA	GLU	A	13	14.901	1.950	-1.974	28.47	0.00	C
ATOM	51	C	GLU	A	13	14.358	1.037	-0.868	28.47	0.00	C
ATOM	52	O	GLU	A	13	13.966	1.505	0.190	28.47	0.00	O
ATOM	53	N	HIS	A	14	14.335	-0.267	-1.101	20.56	0.00	N
ATOM	54	CA	HIS	A	14	13.794	-1.199	-0.106	20.56	0.00	C
ATOM	55	C	HIS	A	14	14.560	-1.126	1.236	20.56	0.00	C
ATOM	56	O	HIS	A	14	13.970	-1.122	2.322	20.56	0.00	O
ATOM	57	N	LEU	A	15	15.884	-1.079	1.145	7.47	0.00	N
ATOM	58	CA	LEU	A	15	16.750	-1.011	2.322	7.47	0.00	C
ATOM	59	C	LEU	A	15	16.724	0.377	2.988	7.47	0.00	C
ATOM	60	O	LEU	A	15	17.114	0.492	4.140	7.47	0.00	O
ATOM	61	N	SER	A	16	16.283	1.421	2.277	18.29	0.00	N
ATOM	62	CA	SER	A	16	16.215	2.761	2.870	18.29	0.00	C
ATOM	63	C	SER	A	16	15.126	2.866	3.937	18.29	0.00	C
ATOM	64	O	SER	A	16	15.184	3.766	4.746	18.29	0.00	O
ATOM	65	N	HIS	A	17	14.126	1.987	3.905	18.83	0.00	N
ATOM	66	CA	HIS	A	17	12.931	2.175	4.710	18.83	0.00	C
ATOM	67	C	HIS	A	17	12.911	1.290	5.925	18.83	0.00	C
ATOM	68	O	HIS	A	17	12.211	0.285	5.939	18.83	0.00	O
ATOM	69	N	VAL	A	18	13.667	1.662	6.952	21.61	0.00	N
ATOM	70	CA	VAL	A	18	13.649	0.930	8.226	21.61	0.00	C
ATOM	71	C	VAL	A	18	12.455	1.416	9.073	21.61	0.00	C
ATOM	72	O	VAL	A	18	12.231	2.619	9.198	21.61	0.00	O
ATOM	73	N	LYS	A	19	11.711	0.480	9.654	41.33	0.00	N
ATOM	74	CA	LYS	A	19	10.394	0.738	10.251	41.33	0.00	C
ATOM	75	C	LYS	A	19	10.466	0.797	11.779	41.33	0.00	C
ATOM	76	O	LYS	A	19	11.024	-0.102	12.394	41.33	0.00	O
ATOM	77	N	GLU	A	20	9.892	1.832	12.391	49.34	0.00	N
ATOM	78	CA	GLU	A	20	10.003	2.007	13.848	49.34	0.00	C
ATOM	79	C	GLU	A	20	8.986	1.178	14.597	49.34	0.00	C
ATOM	80	O	GLU	A	20	7.952	0.808	14.049	49.34	0.00	O
ATOM	81	N	GLU	A	21	9.300	0.899	15.861	48.16	0.00	N
ATOM	82	CA	GLU	A	21	8.634	-0.168	16.607	48.16	0.00	C
ATOM	83	C	GLU	A	21	7.153	0.124	16.844	48.16	0.00	C
ATOM	84	O	GLU	A	21	6.369	-0.811	17.040	48.16	0.00	O
ATOM	85	N	ASN	A	22	6.774	1.399	16.808	60.36	0.00	N
ATOM	86	CA	ASN	A	22	5.371	1.790	16.970	60.36	0.00	C
ATOM	87	C	ASN	A	22	4.716	2.391	15.715	60.36	0.00	C
ATOM	88	O	ASN	A	22	3.648	3.000	15.824	60.36	0.00	O
ATOM	89	N	THR	A	23	5.339	2.269	14.540	74.63	0.00	N
ATOM	90	CA	THR	A	23	4.636	2.674	13.297	74.63	0.00	C
ATOM	91	C	THR	A	23	3.937	1.492	12.597	74.63	0.00	C
ATOM	92	O	THR	A	23	4.343	0.315	12.707	74.63	0.00	O
ATOM	93	N	ILE	A	24	2.863	1.841	11.907	61.70	0.00	N
ATOM	94	CA	ILE	A	24	2.133	0.908	11.089	61.70	0.00	C
ATOM	95	C	ILE	A	24	1.893	1.588	9.781	61.70	0.00	C
ATOM	96	O	ILE	A	24	1.270	2.646	9.760	61.70	0.00	O
ATOM	97	N	TYR	A	25	2.393	1.015	8.691	57.19	0.00	N
ATOM	98	CA	TYR	A	25	2.072	1.558	7.393	57.19	0.00	C
ATOM	99	C	TYR	A	25	0.609	1.246	7.051	57.19	0.00	C
ATOM	100	O	TYR	A	25	0.029	0.273	7.510	57.19	0.00	O
ATOM	101	N	VAL	A	26	0.019	2.088	6.230	64.40	0.00	N
ATOM	102	CA	VAL	A	26	-1.326	1.856	5.716	64.40	0.00	C
ATOM	103	C	VAL	A	26	-1.394	0.504	4.989	64.40	0.00	C
ATOM	104	O	VAL	A	26	-2.320	-0.244	5.138	64.40	0.00	O
ATOM	105	N	THR	A	27	-0.413	0.264	4.153	61.12	0.00	N
ATOM	106	CA	THR	A	27	-0.225	-0.940	3.432	61.12	0.00	C
ATOM	107	C	THR	A	27	-0.214	-2.155	4.325	61.12	0.00	C
ATOM	108	O	THR	A	27	-0.816	-3.189	4.005	61.12	0.00	O
ATOM	109	N	ASP	A	28	0.421	-2.066	5.476	60.47	0.00	N
ATOM	110	CA	ASP	A	28	0.217	-3.125	6.447	60.47	0.00	C
ATOM	111	C	ASP	A	28	-1.221	-3.366	6.899	60.47	0.00	C
ATOM	112	O	ASP	A	28	-1.560	-4.494	7.172	60.47	0.00	O
ATOM	113	N	LEU	A	29	-2.082	-2.355	6.958	76.20	0.00	N
ATOM	114	CA	LEU	A	29	-3.418	-2.565	7.521	76.20	0.00	C
ATOM	115	C	LEU	A	29	-4.304	-3.447	6.637	76.20	0.00	C
ATOM	116	O	LEU	A	29	-5.335	-3.962	7.065	76.20	0.00	O
ATOM	117	N	VAL	A	30	-3.930	-3.597	5.378	58.21	0.00	N
ATOM	118	CA	VAL	A	30	-4.776	-4.325	4.449	58.21	0.00	C
ATOM	119	C	VAL	A	30	-4.099	-5.619	4.014	58.21	0.00	C
ATOM	120	O	VAL	A	30	-4.413	-6.174	2.971	58.21	0.00	O
ATOM	121	N	ARG	A	31	-3.154	-6.069	4.828	99.99	0.00	N
ATOM	122	CA	ARG	A	31	-2.426	-7.287	4.613	99.99	0.00	C
ATOM	123	C	ARG	A	31	-2.433	-8.174	5.834	99.99	0.00	C
ATOM	124	O	ARG	A	31	-2.927	-7.806	6.907	99.99	0.00	O
ATOM	125	N	CYS	A	32	-1.955	-9.395	5.616	78.31	0.00	N
ATOM	126	CA	CYS	A	32	-1.910	-10.426	6.637	78.31	0.00	C
ATOM	127	C	CYS	A	32	-1.015	-9.951	7.770	78.31	0.00	C
ATOM	128	O	CYS	A	32	0.134	-9.567	7.534	78.31	0.00	O
ATOM	129	N	PRO	A	33	-1.508	-10.024	8.999	51.38	0.00	N
ATOM	130	CA	PRO	A	33	-0.664	-9.610	10.083	51.38	0.00	C
ATOM	131	C	PRO	A	33	0.611	-10.429	10.223	51.38	0.00	C
ATOM	132	O	PRO	A	33	1.694	-9.852	10.411	51.38	0.00	O
ATOM	133	N	ARG	A	34	0.512	-11.757	10.119	62.75	0.00	N
ATOM	134	CA	ARG	A	34	1.689	-12.574	10.256	62.75	0.00	C
ATOM	135	C	ARG	A	34	2.720	-12.198	9.218	62.75	0.00	C
ATOM	136	O	ARG	A	34	3.911	-12.267	9.453	62.75	0.00	O

```
ATOM    137  N   ARG A  35       2.276 -11.837   8.033 35.09  0.00           N
ATOM    138  CA  ARG A  35       3.238 -11.530   6.965 35.09  0.00           C
ATOM    139  C   ARG A  35       3.971 -10.221   7.251 35.09  0.00           C
ATOM    140  O   ARG A  35       5.151 -10.042   6.901 35.09  0.00           O
ATOM    141  N   VAL A  36       3.228  -9.297   7.848 55.22  0.00           N
ATOM    142  CA  VAL A  36       3.791  -7.981   8.175 55.22  0.00           C
ATOM    143  C   VAL A  36       4.864  -8.200   9.230 55.22  0.00           C
ATOM    144  O   VAL A  36       5.972  -7.734   9.085 55.22  0.00           O
ATOM    145  N   ARG A  37       4.561  -8.972  10.259 66.63  0.00           N
ATOM    146  CA  ARG A  37       5.600  -9.259  11.227 66.63  0.00           C
ATOM    147  C   ARG A  37       6.749 -10.045  10.630 66.63  0.00           C
ATOM    148  O   ARG A  37       7.879  -9.890  11.061 66.63  0.00           O
ATOM    149  N   TYR A  38       6.481 -10.904   9.649 51.44  0.00           N
ATOM    150  CA  TYR A  38       7.570 -11.660   9.036 51.44  0.00           C
ATOM    151  C   TYR A  38       8.499 -10.724   8.363 51.44  0.00           C
ATOM    152  O   TYR A  38       9.689 -10.942   8.377 51.44  0.00           O
ATOM    153  N   GLU A  39       7.963  -9.678   7.760 76.94  0.00           N
ATOM    154  CA  GLU A  39       8.816  -8.667   7.126 76.94  0.00           C
ATOM    155  C   GLU A  39       9.769  -7.904   8.077 76.94  0.00           C
ATOM    156  O   GLU A  39      10.773  -7.394   7.597 76.94  0.00           O
ATOM    157  N   SER A  40       9.464  -7.844   9.380 51.76  0.00           N
ATOM    158  CA  SER A  40      10.398  -7.338  10.412 51.76  0.00           C
ATOM    159  C   SER A  40      11.345  -8.393  10.958 51.76  0.00           C
ATOM    160  O   SER A  40      12.540  -8.130  11.114 51.76  0.00           O
ATOM    161  N   GLU A  41      10.808  -9.578  11.263 49.17  0.00           N
ATOM    162  CA  GLU A  41      11.604 -10.637  11.865 49.17  0.00           C
ATOM    163  C   GLU A  41      12.456 -11.415  10.889 49.17  0.00           C
ATOM    164  O   GLU A  41      13.321 -12.135  11.370 49.17  0.00           O
ATOM    165  N   TYR A  42      12.238 -11.336   9.566 54.89  0.00           N
ATOM    166  CA  TYR A  42      13.052 -12.135   8.586 54.89  0.00           C
ATOM    167  C   TYR A  42      13.369 -11.290   7.378 54.89  0.00           C
ATOM    168  O   TYR A  42      13.024 -11.599   6.196 54.89  0.00           O
ATOM    169  N   LYS A  43      14.059 -10.210   7.691 62.62  0.00           N
ATOM    170  CA  LYS A  43      14.286  -9.169   6.756 62.62  0.00           C
ATOM    171  C   LYS A  43      15.175  -9.664   5.651 62.62  0.00           C
ATOM    172  O   LYS A  43      15.118  -9.127   4.534 62.62  0.00           O
ATOM    173  N   GLU A  44      16.024 -10.635   5.936 58.52  0.00           N
ATOM    174  CA  GLU A  44      16.906 -11.087   4.887 58.52  0.00           C
ATOM    175  C   GLU A  44      16.077 -11.799   3.791 58.52  0.00           C
ATOM    176  O   GLU A  44      16.309 -11.615   2.572 58.52  0.00           O
ATOM    177  N   LEU A  45      15.070 -12.551   4.234 58.40  0.00           N
ATOM    178  CA  LEU A  45      14.177 -13.244   3.311 58.40  0.00           C
ATOM    179  C   LEU A  45      13.268 -12.185   2.642 58.40  0.00           C
ATOM    180  O   LEU A  45      13.095 -12.177   1.406 58.40  0.00           O
ATOM    181  N   ALA A  46      12.710 -11.278   3.448 72.70  0.00           N
ATOM    182  CA  ALA A  46      11.893 -10.178   2.897 72.70  0.00           C
ATOM    183  C   ALA A  46      12.519  -9.534   1.649 72.70  0.00           C
ATOM    184  O   ALA A  46      11.825  -9.262   0.709 72.70  0.00           O
ATOM    185  N   ILE A  47      13.830  -9.341   1.634 57.95  0.00           N
ATOM    186  CA  ILE A  47      14.526  -8.691   0.538 57.95  0.00           C
ATOM    187  C   ILE A  47      14.299  -9.332  -0.838 57.95  0.00           C
ATOM    188  O   ILE A  47      14.451  -8.669  -1.895 57.95  0.00           O
ATOM    189  N   SER A  48      13.959 -10.620  -0.874 66.06  0.00           N
ATOM    190  CA  SER A  48      13.715 -11.277  -2.169 66.06  0.00           C
ATOM    191  C   SER A  48      12.509 -10.666  -2.882 66.06  0.00           C
ATOM    192  O   SER A  48      12.401 -10.723  -4.108 66.06  0.00           O
ATOM    193  N   GLN A  49      11.610 -10.068  -2.123 66.28  0.00           N
ATOM    194  CA  GLN A  49      10.459  -9.434  -2.743 66.28  0.00           C
ATOM    195  C   GLN A  49      10.847  -8.226  -3.623 66.28  0.00           C
ATOM    196  O   GLN A  49      10.116  -7.889  -4.482 66.28  0.00           O
ATOM    197  N   VAL A  50      12.031  -7.643  -3.424 60.14  0.00           N
ATOM    198  CA  VAL A  50      12.659  -6.700  -4.358 60.14  0.00           C
ATOM    199  C   VAL A  50      12.750  -7.176  -5.802 60.14  0.00           C
ATOM    200  O   VAL A  50      12.805  -6.373  -6.754 60.14  0.00           O
ATOM    201  N   TYR A  51      12.865  -8.468  -5.991 32.22  0.00           N
ATOM    202  CA  TYR A  51      13.231  -8.953  -7.306 32.22  0.00           C
ATOM    203  C   TYR A  51      12.000  -9.515  -8.013 32.22  0.00           C
ATOM    204  O   TYR A  51      12.116 -10.179  -9.031 32.22  0.00           O
ATOM    205  N   ALA A  52      10.823  -9.258  -7.445 41.28  0.00           N
ATOM    206  CA  ALA A  52       9.580  -9.586  -8.075 41.28  0.00           C
ATOM    207  C   ALA A  52       9.375  -8.694  -9.336 41.28  0.00           C
ATOM    208  O   ALA A  52       9.152  -7.478  -9.216 41.28  0.00           O
ATOM    209  N   PRO A  53       9.401  -9.282 -10.541 48.93  0.00           N
ATOM    210  CA  PRO A  53       9.338  -8.519 -11.804 48.93  0.00           C
ATOM    211  C   PRO A  53       8.051  -7.715 -11.969 48.93  0.00           C
ATOM    212  O   PRO A  53       8.107  -6.662 -12.589 48.93  0.00           O
ATOM    213  N   SER A  54       6.912  -8.185 -11.428 36.30  0.00           N
ATOM    214  CA  SER A  54       5.662  -7.382 -11.372 36.30  0.00           C
ATOM    215  C   SER A  54       5.807  -6.143 -10.551 36.30  0.00           C
ATOM    216  O   SER A  54       5.159  -5.116 -10.806 36.30  0.00           O
ATOM    217  N   ALA A  55       6.666  -6.210  -9.534 32.49  0.00           N
ATOM    218  CA  ALA A  55       6.871  -5.023  -8.707 32.49  0.00           C
ATOM    219  C   ALA A  55       7.756  -4.055  -9.490 32.49  0.00           C
ATOM    220  O   ALA A  55       7.598  -2.860  -9.403 32.49  0.00           O
ATOM    221  N   ILE A  56       8.662  -4.543 -10.314 36.07  0.00           N
ATOM    222  CA  ILE A  56       9.562  -3.610 -11.005 36.07  0.00           C
ATOM    223  C   ILE A  56       8.724  -2.964 -12.115 36.07  0.00           C
ATOM    224  O   ILE A  56       8.790  -1.755 -12.348 36.07  0.00           O
ATOM    225  N   LEU A  57       7.912  -3.805 -12.760 30.30  0.00           N
ATOM    226  CA  LEU A  57       6.935  -3.366 -13.747 30.30  0.00           C
ATOM    227  C   LEU A  57       6.033  -2.336 -13.087 30.30  0.00           C
ATOM    228  O   LEU A  57       5.874  -1.207 -13.590 30.30  0.00           O
```

ATOM	229	N	GLY	A	58	5.483	-2.701	-11.946	40.09	0.00	N
ATOM	230	CA	GLY	A	58	4.634	-1.806	-11.182	40.09	0.00	C
ATOM	231	C	GLY	A	58	5.258	-0.473	-10.875	40.09	0.00	C
ATOM	232	O	GLY	A	58	4.607	0.560	-11.036	40.09	0.00	O
ATOM	233	N	ASP	A	59	6.514	-0.439	-10.443	43.66	0.00	N
ATOM	234	CA	ASP	A	59	7.150	0.844	-10.105	43.66	0.00	C
ATOM	235	C	ASP	A	59	7.486	1.775	-11.275	43.66	0.00	C
ATOM	236	O	ASP	A	59	7.461	2.943	-11.121	43.66	0.00	O
ATOM	237	N	ILE	A	60	7.874	1.212	-12.405	47.37	0.00	N
ATOM	238	CA	ILE	A	60	8.072	1.923	-13.651	47.37	0.00	C
ATOM	239	C	ILE	A	60	6.774	2.586	-14.133	47.37	0.00	C
ATOM	240	O	ILE	A	60	6.798	3.761	-14.559	47.37	0.00	O
ATOM	241	N	LEU	A	61	5.641	1.870	-14.058	37.07	0.00	N
ATOM	242	CA	LEU	A	61	4.334	2.454	-14.392	37.07	0.00	C
ATOM	243	C	LEU	A	61	4.024	3.653	-13.537	37.07	0.00	C
ATOM	244	O	LEU	A	61	3.668	4.678	-14.050	37.07	0.00	O
ATOM	245	N	HIS	A	62	4.172	3.508	-12.232	40.48	0.00	N
ATOM	246	CA	HIS	A	62	3.968	4.616	-11.306	40.48	0.00	C
ATOM	247	C	HIS	A	62	4.884	5.714	-11.684	40.48	0.00	C
ATOM	248	O	HIS	A	62	4.443	6.854	-11.841	40.48	0.00	O
ATOM	249	N	LEU	A	63	6.158	5.423	-11.888	37.63	0.00	N
ATOM	250	CA	LEU	A	63	7.034	6.526	-12.325	37.63	0.00	C
ATOM	251	C	LEU	A	63	6.405	7.217	-13.537	37.63	0.00	C
ATOM	252	O	LEU	A	63	6.167	8.394	-13.497	37.63	0.00	O
ATOM	253	N	GLY	A	64	6.109	6.499	-14.603	32.57	0.00	N
ATOM	254	CA	GLY	A	64	5.719	7.158	-15.811	32.57	0.00	C
ATOM	255	C	GLY	A	64	4.400	7.890	-15.740	32.57	0.00	C
ATOM	256	O	GLY	A	64	4.174	8.755	-16.509	32.57	0.00	O
ATOM	257	N	LEU	A	65	3.499	7.454	-14.886	17.30	0.00	N
ATOM	258	CA	LEU	A	65	2.129	7.928	-14.922	17.30	0.00	C
ATOM	259	C	LEU	A	65	2.136	9.075	-13.968	17.30	0.00	C
ATOM	260	O	LEU	A	65	1.699	10.153	-14.266	17.30	0.00	O
ATOM	261	N	GLU	A	66	2.672	8.845	-12.789	52.15	0.00	N
ATOM	262	CA	GLU	A	66	3.061	9.970	-11.943	52.15	0.00	C
ATOM	263	C	GLU	A	66	3.702	11.154	-12.688	52.15	0.00	C
ATOM	264	O	GLU	A	66	3.297	12.272	-12.436	52.15	0.00	O
ATOM	265	N	SER	A	67	4.665	10.981	-13.581	48.13	0.00	N
ATOM	266	CA	SER	A	67	5.183	12.221	-14.250	48.13	0.00	C
ATOM	267	C	SER	A	67	4.100	13.012	-15.067	48.13	0.00	C
ATOM	268	O	SER	A	67	4.182	14.248	-15.203	48.13	0.00	O
ATOM	269	N	VAL	A	68	3.091	12.293	-15.600	43.23	0.00	N
ATOM	270	CA	VAL	A	68	1.939	12.934	-16.238	43.23	0.00	C
ATOM	271	C	VAL	A	68	1.158	13.773	-15.231	43.23	0.00	C
ATOM	272	O	VAL	A	68	0.645	14.865	-15.520	43.23	0.00	O
ATOM	273	N	LEU	A	69	1.040	13.216	-14.047	36.64	0.00	N
ATOM	274	CA	LEU	A	69	0.261	13.818	-13.004	36.64	0.00	C
ATOM	275	C	LEU	A	69	0.848	15.118	-12.430	36.64	0.00	C
ATOM	276	O	LEU	A	69	0.134	15.992	-11.952	36.64	0.00	O
ATOM	277	N	LYS	A	70	2.165	15.215	-12.445	44.43	0.00	N
ATOM	278	CA	LYS	A	70	2.860	16.356	-11.911	44.43	0.00	C
ATOM	279	C	LYS	A	70	2.708	17.450	-12.934	44.43	0.00	C
ATOM	280	O	LYS	A	70	2.497	18.596	-12.562	44.43	0.00	O
ATOM	281	N	GLY	A	71	2.769	17.105	-14.229	47.19	0.00	N
ATOM	282	CA	GLY	A	71	2.550	18.111	-15.305	47.19	0.00	C
ATOM	283	C	GLY	A	71	1.130	18.685	-15.376	47.19	0.00	C
ATOM	284	O	GLY	A	71	0.821	19.815	-14.923	47.19	0.00	O
ATOM	285	N	ASN	A	72	0.217	17.876	-15.876	43.26	0.00	N
ATOM	286	CA	ASN	A	72	-1.095	18.370	-16.206	43.26	0.00	C
ATOM	287	C	ASN	A	72	-2.040	18.612	-15.074	43.26	0.00	C
ATOM	288	O	ASN	A	72	-2.921	19.420	-15.211	43.26	0.00	O
ATOM	289	N	PHE	A	73	-1.880	17.933	-13.966	39.90	0.00	N
ATOM	290	CA	PHE	A	73	-2.766	18.118	-12.847	39.90	0.00	C
ATOM	291	C	PHE	A	73	-2.091	18.771	-11.639	39.90	0.00	C
ATOM	292	O	PHE	A	73	-2.674	18.861	-10.593	39.90	0.00	O
ATOM	293	N	ASN	A	74	-0.838	19.166	-11.735	60.33	0.00	N
ATOM	294	CA	ASN	A	74	-0.201	19.781	-10.582	60.33	0.00	C
ATOM	295	C	ASN	A	74	-0.355	18.949	-9.279	60.33	0.00	C
ATOM	296	O	ASN	A	74	-0.590	19.474	-8.165	60.33	0.00	O
ATOM	297	N	ALA	A	75	-0.160	17.641	-9.414	52.99	0.00	N
ATOM	298	CA	ALA	A	75	-0.115	16.792	-8.245	52.99	0.00	C
ATOM	299	C	ALA	A	75	1.282	16.777	-7.622	52.99	0.00	C
ATOM	300	O	ALA	A	75	2.270	16.992	-8.289	52.99	0.00	O
ATOM	301	N	GLU	A	76	1.336	16.530	-6.332	61.81	0.00	N
ATOM	302	CA	GLU	A	76	2.559	16.069	-5.695	61.81	0.00	C
ATOM	303	C	GLU	A	76	2.544	14.546	-5.834	61.81	0.00	C
ATOM	304	O	GLU	A	76	1.466	13.957	-5.865	61.81	0.00	O
ATOM	305	N	THR	A	77	3.710	13.915	-5.909	66.18	0.00	N
ATOM	306	CA	THR	A	77	3.794	12.484	-6.087	66.18	0.00	C
ATOM	307	C	THR	A	77	4.639	11.898	-4.978	66.18	0.00	C
ATOM	308	O	THR	A	77	5.510	12.566	-4.500	66.18	0.00	O
ATOM	309	N	GLU	A	78	4.321	10.666	-4.562	71.90	0.00	N
ATOM	310	CA	GLU	A	78	4.988	9.920	-3.517	71.90	0.00	C
ATOM	311	C	GLU	A	78	5.020	10.703	-2.232	71.90	0.00	C
ATOM	312	O	GLU	A	78	6.063	10.936	-1.623	71.90	0.00	O
ATOM	313	N	VAL	A	79	3.826	11.074	-1.816	45.89	0.00	N
ATOM	314	CA	VAL	A	79	3.635	12.010	-0.767	45.89	0.00	C
ATOM	315	C	VAL	A	79	3.474	11.303	0.544	45.89	0.00	C
ATOM	316	O	VAL	A	79	2.510	10.573	0.774	45.89	0.00	O
ATOM	317	N	GLU	A	80	4.416	11.580	1.421	26.43	0.00	N
ATOM	318	CA	GLU	A	80	4.530	10.857	2.668	26.43	0.00	C
ATOM	319	C	GLU	A	80	3.901	11.655	3.780	26.43	0.00	C
ATOM	320	O	GLU	A	80	4.090	12.840	3.853	26.43	0.00	O

```
ATOM    321  N    THR A  81       3.177  11.027   4.670 34.54  0.00           N
ATOM    322  CA   THR A  81       2.510  11.744   5.761 34.54  0.00           C
ATOM    323  C    THR A  81       2.133  10.811   6.884 34.54  0.00           C
ATOM    324  O    THR A  81       2.164   9.597   6.754 34.54  0.00           O
ATOM    325  N    LEU A  82       1.667  11.373   7.976 26.00  0.00           N
ATOM    326  CA   LEU A  82       1.644  10.601   9.202 26.00  0.00           C
ATOM    327  C    LEU A  82       0.492  11.060  10.035 26.00  0.00           C
ATOM    328  O    LEU A  82       0.098  12.203   9.950 26.00  0.00           O
ATOM    329  N    ARG A  83      -0.124  10.141  10.761  0.00  0.00           N
ATOM    330  CA   ARG A  83      -1.326  10.449  11.518  0.00  0.00           C
ATOM    331  C    ARG A  83      -1.368   9.467  12.659  0.00  0.00           C
ATOM    332  O    ARG A  83      -0.926   8.327  12.512  0.00  0.00           O
ATOM    333  N    GLU A  84      -1.873   9.890  13.809 16.62  0.00           N
ATOM    334  CA   GLU A  84      -1.727   9.022  14.978 16.62  0.00           C
ATOM    335  C    GLU A  84      -3.042   8.425  15.425 16.62  0.00           C
ATOM    336  O    GLU A  84      -4.110   8.996  15.183 16.62  0.00           O
ATOM    337  N    ILE A  85      -2.918   7.275  16.095 49.03  0.00           N
ATOM    338  CA   ILE A  85      -4.029   6.451  16.449 49.03  0.00           C
ATOM    339  C    ILE A  85      -3.781   5.841  17.813 49.03  0.00           C
ATOM    340  O    ILE A  85      -2.643   5.507  18.123 49.03  0.00           O
ATOM    341  N    ASN A  86      -4.840   5.701  18.619 23.99  0.00           N
ATOM    342  CA   ASN A  86      -4.761   5.021  19.937 23.99  0.00           C
ATOM    343  C    ASN A  86      -5.250   3.566  19.895 23.99  0.00           C
ATOM    344  O    ASN A  86      -6.399   3.293  19.511 23.99  0.00           O
ATOM    345  N    VAL A  87      -4.383   2.645  20.322 34.12  0.00           N
ATOM    346  CA   VAL A  87      -4.703   1.227  20.308 34.12  0.00           C
ATOM    347  C    VAL A  87      -4.355   0.547  21.630 34.12  0.00           C
ATOM    348  O    VAL A  87      -3.230   0.067  21.819 34.12  0.00           O
ATOM    349  N    GLY A  88      -5.321   0.484  22.542 45.20  0.00           N
ATOM    350  CA   GLY A  88      -5.066  -0.115  23.865 45.20  0.00           C
ATOM    351  C    GLY A  88      -4.192   0.803  24.708 45.20  0.00           C
ATOM    352  O    GLY A  88      -3.216   0.373  25.351 45.20  0.00           O
ATOM    353  N    GLY A  89      -4.538   2.089  24.663 44.67  0.00           N
ATOM    354  CA   GLY A  89      -3.780   3.124  25.348 44.67  0.00           C
ATOM    355  C    GLY A  89      -2.339   3.328  24.890 44.67  0.00           C
ATOM    356  O    GLY A  89      -1.668   4.231  25.406 44.67  0.00           O
ATOM    357  N    LYS A  90      -1.846   2.502  23.956 48.32  0.00           N
ATOM    358  CA   LYS A  90      -0.547   2.733  23.318 48.32  0.00           C
ATOM    359  C    LYS A  90      -0.830   3.651  22.138 48.32  0.00           C
ATOM    360  O    LYS A  90      -1.881   3.541  21.497 48.32  0.00           O
ATOM    361  N    VAL A  91       0.069   4.592  21.872 38.80  0.00           N
ATOM    362  CA   VAL A  91      -0.106   5.503  20.744 38.80  0.00           C
ATOM    363  C    VAL A  91       0.675   4.963  19.561 38.80  0.00           C
ATOM    364  O    VAL A  91       1.817   4.523  19.727 38.80  0.00           O
ATOM    365  N    TYR A  92       0.052   5.005  18.377 52.81  0.00           N
ATOM    366  CA   TYR A  92       0.676   4.480  17.190 52.81  0.00           C
ATOM    367  C    TYR A  92       0.645   5.490  16.087 52.81  0.00           C
ATOM    368  O    TYR A  92      -0.329   6.191  15.893 52.81  0.00           O
ATOM    369  N    LYS A  93       1.747   5.547  15.371 52.93  0.00           N
ATOM    370  CA   LYS A  93       1.859   6.412  14.217 52.93  0.00           C
ATOM    371  C    LYS A  93       1.524   5.603  12.955 52.93  0.00           C
ATOM    372  O    LYS A  93       2.270   4.714  12.581 52.93  0.00           O
ATOM    373  N    ILE A  94       0.406   5.927  12.320 51.17  0.00           N
ATOM    374  CA   ILE A  94       0.084   5.372  11.038 51.17  0.00           C
ATOM    375  C    ILE A  94       0.743   6.184   9.929 51.17  0.00           C
ATOM    376  O    ILE A  94       0.556   7.385   9.807 51.17  0.00           O
ATOM    377  N    LYS A  95       1.497   5.507   9.097 49.55  0.00           N
ATOM    378  CA   LYS A  95       2.305   6.166   8.106 49.55  0.00           C
ATOM    379  C    LYS A  95       1.950   5.702   6.699 49.55  0.00           C
ATOM    380  O    LYS A  95       1.760   4.545   6.470 49.55  0.00           O
ATOM    381  N    GLY A  96       1.853   6.613   5.743 51.60  0.00           N
ATOM    382  CA   GLY A  96       1.572   6.242   4.383 51.60  0.00           C
ATOM    383  C    GLY A  96       2.142   7.179   3.378 51.60  0.00           C
ATOM    384  O    GLY A  96       2.537   8.280   3.703 51.60  0.00           O
ATOM    385  N    ARG A  97       2.107   6.740   2.138 35.35  0.00           N
ATOM    386  CA   ARG A  97       2.693   7.428   1.007 35.35  0.00           C
ATOM    387  C    ARG A  97       1.769   7.206  -0.178 35.35  0.00           C
ATOM    388  O    ARG A  97       1.672   6.084  -0.699 35.35  0.00           O
ATOM    389  N    ALA A  98       1.104   8.281  -0.588 48.81  0.00           N
ATOM    390  CA   ALA A  98       0.117   8.243  -1.592 48.81  0.00           C
ATOM    391  C    ALA A  98       0.862   8.383  -2.906 48.81  0.00           C
ATOM    392  O    ALA A  98       1.855   9.097  -3.010 48.81  0.00           O
ATOM    393  N    ASP A  99       0.424   7.673  -3.912 46.60  0.00           N
ATOM    394  CA   ASP A  99       1.076   7.754  -5.196 46.60  0.00           C
ATOM    395  C    ASP A  99       0.974   9.188  -5.720 46.60  0.00           C
ATOM    396  O    ASP A  99       1.922   9.707  -6.350 46.60  0.00           O
ATOM    397  N    ALA A 100      -0.166   9.846  -5.513 51.50  0.00           N
ATOM    398  CA   ALA A 100      -0.251  11.269  -5.953 51.50  0.00           C
ATOM    399  C    ALA A 100      -1.290  11.992  -5.184 51.50  0.00           C
ATOM    400  O    ALA A 100      -2.232  11.366  -4.731 51.50  0.00           O
ATOM    401  N    ILE A 101      -1.104  13.303  -4.998 67.57  0.00           N
ATOM    402  CA   ILE A 101      -2.137  14.137  -4.384 67.57  0.00           C
ATOM    403  C    ILE A 101      -2.390  15.420  -5.140 67.57  0.00           C
ATOM    404  O    ILE A 101      -1.467  16.143  -5.558 67.57  0.00           O
ATOM    405  N    ILE A 102      -3.672  15.695  -5.329 54.39  0.00           N
ATOM    406  CA   ILE A 102      -4.083  17.004  -5.796 54.39  0.00           C
ATOM    407  C    ILE A 102      -4.628  17.765  -4.581 54.39  0.00           C
ATOM    408  O    ILE A 102      -5.695  17.431  -4.073 54.39  0.00           O
ATOM    409  N    ARG A 103      -3.845  18.751  -4.103 48.81  0.00           N
ATOM    410  CA   ARG A 103      -4.150  19.504  -2.879 48.81  0.00           C
ATOM    411  C    ARG A 103      -5.423  20.391  -2.949 48.81  0.00           C
ATOM    412  O    ARG A 103      -6.199  20.482  -1.960 48.81  0.00           O
```

```
ATOM    413  N   ASN A 104      -5.626  21.051  -4.095 49.62  0.00           N
ATOM    414  CA  ASN A 104      -6.745  21.998  -4.259 49.62  0.00           C
ATOM    415  C   ASN A 104      -7.230  21.994  -5.679 49.62  0.00           C
ATOM    416  O   ASN A 104      -6.693  22.692  -6.538 49.62  0.00           O
ATOM    417  N   ASP A 105      -8.253  21.174  -5.879 50.98  0.00           N
ATOM    418  CA  ASP A 105      -9.039  21.134  -7.084 50.98  0.00           C
ATOM    419  C   ASP A 105     -10.471  21.605  -6.714 50.98  0.00           C
ATOM    420  O   ASP A 105     -11.262  20.843  -6.158 50.98  0.00           O
ATOM    421  N   ASN A 106     -10.768  22.878  -6.986 41.30  0.00           N
ATOM    422  CA  ASN A 106     -12.040  23.506  -6.575 41.30  0.00           C
ATOM    423  C   ASN A 106     -12.422  23.321  -5.104 41.30  0.00           C
ATOM    424  O   ASN A 106     -13.561  23.020  -4.774 41.30  0.00           O
ATOM    425  N   GLY A 107     -11.470  23.524  -4.215 55.42  0.00           N
ATOM    426  CA  GLY A 107     -11.717  23.337  -2.789 55.42  0.00           C
ATOM    427  C   GLY A 107     -11.737  21.883  -2.335 55.42  0.00           C
ATOM    428  O   GLY A 107     -12.404  21.544  -1.352 55.42  0.00           O
ATOM    429  N   LYS A 108     -11.008  21.019  -3.043 47.85  0.00           N
ATOM    430  CA  LYS A 108     -10.915  19.615  -2.668 47.85  0.00           C
ATOM    431  C   LYS A 108      -9.483  19.049  -2.884 47.85  0.00           C
ATOM    432  O   LYS A 108      -8.779  19.403  -3.839 47.85  0.00           O
ATOM    433  N   SER A 109      -9.039  18.236  -1.933 50.40  0.00           N
ATOM    434  CA  SER A 109      -7.862  17.387  -2.118 50.40  0.00           C
ATOM    435  C   SER A 109      -8.308  16.067  -2.739 50.40  0.00           C
ATOM    436  O   SER A 109      -9.315  15.517  -2.353 50.40  0.00           O
ATOM    437  N   ILE A 110      -7.524  15.551  -3.650 39.96  0.00           N
ATOM    438  CA  ILE A 110      -7.790  14.265  -4.209 39.96  0.00           C
ATOM    439  C   ILE A 110      -6.588  13.343  -3.999 39.96  0.00           C
ATOM    440  O   ILE A 110      -5.512  13.629  -4.510 39.96  0.00           O
ATOM    441  N   VAL A 111      -6.752  12.243  -3.266 55.49  0.00           N
ATOM    442  CA  VAL A 111      -5.679  11.220  -3.198 55.49  0.00           C
ATOM    443  C   VAL A 111      -5.836  10.276  -4.388 55.49  0.00           C
ATOM    444  O   VAL A 111      -6.932   9.764  -4.636 55.49  0.00           O
ATOM    445  N   ILE A 112      -4.775  10.018  -5.128 50.38  0.00           N
ATOM    446  CA  ILE A 112      -4.866   9.126  -6.282 50.38  0.00           C
ATOM    447  C   ILE A 112      -4.010   7.894  -6.056 50.38  0.00           C
ATOM    448  O   ILE A 112      -2.842   8.019  -5.770 50.38  0.00           O
ATOM    449  N   GLU A 113      -4.547   6.709  -6.243 55.15  0.00           N
ATOM    450  CA  GLU A 113      -3.780   5.455  -6.060 55.15  0.00           C
ATOM    451  C   GLU A 113      -3.823   4.710  -7.388 55.15  0.00           C
ATOM    452  O   GLU A 113      -4.882   4.569  -8.052 55.15  0.00           O
ATOM    453  N   ILE A 114      -2.657   4.354  -7.861 49.82  0.00           N
ATOM    454  CA  ILE A 114      -2.531   3.758  -9.150 49.82  0.00           C
ATOM    455  C   ILE A 114      -2.210   2.295  -8.960 49.82  0.00           C
ATOM    456  O   ILE A 114      -1.423   1.954  -8.099 49.82  0.00           O
ATOM    457  N   LYS A 115      -2.787   1.433  -9.786 55.94  0.00           N
ATOM    458  CA  LYS A 115      -2.621   0.003  -9.616 55.94  0.00           C
ATOM    459  C   LYS A 115      -2.438  -0.643 -10.947 55.94  0.00           C
ATOM    460  O   LYS A 115      -2.964  -0.176 -11.951 55.94  0.00           O
ATOM    461  N   THR A 116      -1.684  -1.729 -10.958 47.50  0.00           N
ATOM    462  CA  THR A 116      -1.468  -2.466 -12.194 47.50  0.00           C
ATOM    463  C   THR A 116      -1.602  -3.921 -11.890 47.50  0.00           C
ATOM    464  O   THR A 116      -1.369  -4.337 -10.804 47.50  0.00           O
ATOM    465  N   SER A 117      -2.085  -4.706 -12.802 38.09  0.00           N
ATOM    466  CA  SER A 117      -2.109  -6.154 -12.601 38.09  0.00           C
ATOM    467  C   SER A 117      -2.180  -6.692 -13.946 38.09  0.00           C
ATOM    468  O   SER A 117      -2.324  -5.911 -14.909 38.09  0.00           O
ATOM    469  N   ARG A 118      -2.032  -8.006 -14.033 50.72  0.00           N
ATOM    470  CA  ARG A 118      -2.068  -8.640 -15.363 50.72  0.00           C
ATOM    471  C   ARG A 118      -3.471  -8.928 -15.862 50.72  0.00           C
ATOM    472  O   ARG A 118      -3.648  -9.162 -17.069 50.72  0.00           O
ATOM    473  N   SER A 119      -4.457  -8.893 -14.965 49.06  0.00           N
ATOM    474  CA  SER A 119      -5.872  -9.062 -15.362 49.06  0.00           C
ATOM    475  C   SER A 119      -6.800  -7.967 -14.770 49.06  0.00           C
ATOM    476  O   SER A 119      -6.533  -7.439 -13.690 49.06  0.00           O
ATOM    477  N   ASP A 120      -7.938  -7.766 -15.424 54.49  0.00           N
ATOM    478  CA  ASP A 120      -8.922  -6.789 -15.023 54.49  0.00           C
ATOM    479  C   ASP A 120     -10.062  -7.464 -14.339 54.49  0.00           C
ATOM    480  O   ASP A 120     -11.180  -6.930 -14.286 54.49  0.00           O
ATOM    481  N   LYS A 121      -9.805  -8.622 -13.800 48.74  0.00           N
ATOM    482  CA  LYS A 121     -10.861  -9.343 -13.147 48.74  0.00           C
ATOM    483  C   LYS A 121     -11.305  -8.719 -11.850 48.74  0.00           C
ATOM    484  O   LYS A 121     -10.493  -8.415 -10.994 48.74  0.00           O
ATOM    485  N   GLY A 122     -12.595  -8.552 -11.661 52.58  0.00           N
ATOM    486  CA  GLY A 122     -13.099  -8.150 -10.341 52.58  0.00           C
ATOM    487  C   GLY A 122     -12.816  -6.695  -9.897 52.58  0.00           C
ATOM    488  O   GLY A 122     -13.062  -6.363  -8.702 52.58  0.00           O
ATOM    489  N   LEU A 123     -12.369  -5.819 -10.826 73.63  0.00           N
ATOM    490  CA  LEU A 123     -12.047  -4.418 -10.440 73.63  0.00           C
ATOM    491  C   LEU A 123     -13.261  -3.672  -9.972 73.63  0.00           C
ATOM    492  O   LEU A 123     -14.252  -3.694 -10.654 73.63  0.00           O
ATOM    493  N   PRO A 124     -13.153  -2.951  -8.838 60.33  0.00           N
ATOM    494  CA  PRO A 124     -11.936  -2.786  -8.048 60.33  0.00           C
ATOM    495  C   PRO A 124     -11.854  -3.776  -6.934 60.33  0.00           C
ATOM    496  O   PRO A 124     -12.861  -4.122  -6.314 60.33  0.00           O
ATOM    497  N   LEU A 125     -10.645  -4.184  -6.634 46.18  0.00           N
ATOM    498  CA  LEU A 125     -10.394  -5.171  -5.542 46.18  0.00           C
ATOM    499  C   LEU A 125     -10.497  -4.530  -4.149 46.18  0.00           C
ATOM    500  O   LEU A 125     -10.112  -3.350  -3.908 46.18  0.00           O
ATOM    501  N   ILE A 126     -11.048  -5.276  -3.221 54.49  0.00           N
ATOM    502  CA  ILE A 126     -11.528  -4.647  -1.996 54.49  0.00           C
ATOM    503  C   ILE A 126     -10.339  -4.126  -1.167 54.49  0.00           C
ATOM    504  O   ILE A 126     -10.462  -3.123  -0.513 54.49  0.00           O
```

```
ATOM    505  N   HIS A 127      -9.195  -4.779  -1.251 57.34  0.00           N
ATOM    506  CA  HIS A 127      -8.055  -4.383  -0.479 57.34  0.00           C
ATOM    507  C   HIS A 127      -7.440  -3.163  -1.103 57.34  0.00           C
ATOM    508  O   HIS A 127      -6.834  -2.382  -0.415 57.34  0.00           O
ATOM    509  N   HIS A 128      -7.592  -2.980  -2.400 49.74  0.00           N
ATOM    510  CA  HIS A 128      -7.176  -1.718  -3.018 49.74  0.00           C
ATOM    511  C   HIS A 128      -8.083  -0.558  -2.609 49.74  0.00           C
ATOM    512  O   HIS A 128      -7.606   0.554  -2.368 49.74  0.00           O
ATOM    513  N   LYS A 129      -9.365  -0.803  -2.526 85.68  0.00           N
ATOM    514  CA  LYS A 129     -10.266   0.264  -2.198 85.68  0.00           C
ATOM    515  C   LYS A 129     -10.028   0.593  -0.745 85.68  0.00           C
ATOM    516  O   LYS A 129      -9.930   1.747  -0.408 85.68  0.00           O
ATOM    517  N   MSE A 130      -9.911  -0.410   0.128 84.86  0.00           N
ATOM    518  CA  MSE A 130      -9.661  -0.122   1.564 84.86  0.00           C
ATOM    519  C   MSE A 130      -8.432   0.772   1.739 84.86  0.00           C
ATOM    520  O   MSE A 130      -8.445   1.649   2.550 84.86  0.00           O
ATOM    521  N   GLN A 131      -7.394   0.520   0.958 72.35  0.00           N
ATOM    522  CA  GLN A 131      -6.133   1.211   1.097 72.35  0.00           C
ATOM    523  C   GLN A 131      -6.400   2.668   0.780 72.35  0.00           C
ATOM    524  O   GLN A 131      -5.918   3.542   1.460 72.35  0.00           O
ATOM    525  N   LEU A 132      -7.195   2.936  -0.242 55.73  0.00           N
ATOM    526  CA  LEU A 132      -7.436   4.290  -0.682 55.73  0.00           C
ATOM    527  C   LEU A 132      -8.295   4.984   0.347 55.73  0.00           C
ATOM    528  O   LEU A 132      -8.142   6.173   0.629 55.73  0.00           O
ATOM    529  N   GLN A 133      -9.173   4.244   0.963 90.16  0.00           N
ATOM    530  CA  GLN A 133     -10.034   4.835   1.951 90.16  0.00           C
ATOM    531  C   GLN A 133      -9.306   5.181   3.263 90.16  0.00           C
ATOM    532  O   GLN A 133      -9.739   6.050   4.021 90.16  0.00           O
ATOM    533  N   ILE A 134      -8.240   4.450   3.561 63.70  0.00           N
ATOM    534  CA  ILE A 134      -7.448   4.750   4.709 63.70  0.00           C
ATOM    535  C   ILE A 134      -6.635   6.008   4.375 63.70  0.00           C
ATOM    536  O   ILE A 134      -6.604   6.904   5.182 63.70  0.00           O
ATOM    537  N   TYR A 135      -5.993   6.089   3.200 52.62  0.00           N
ATOM    538  CA  TYR A 135      -5.308   7.299   2.832 52.62  0.00           C
ATOM    539  C   TYR A 135      -6.263   8.486   3.003 52.62  0.00           C
ATOM    540  O   TYR A 135      -5.876   9.536   3.592 52.62  0.00           O
ATOM    541  N   LEU A 136      -7.510   8.345   2.563 79.77  0.00           N
ATOM    542  CA  LEU A 136      -8.431   9.472   2.663 79.77  0.00           C
ATOM    543  C   LEU A 136      -8.471   9.988   4.123 79.77  0.00           C
ATOM    544  O   LEU A 136      -8.423  11.193   4.364 79.77  0.00           O
ATOM    545  N   TRP A 137      -8.562   9.085   5.079 62.11  0.00           N
ATOM    546  CA  TRP A 137      -8.471   9.465   6.480 62.11  0.00           C
ATOM    547  C   TRP A 137      -7.090  10.092   6.781 62.11  0.00           C
ATOM    548  O   TRP A 137      -6.988  11.182   7.363 62.11  0.00           O
ATOM    549  N   LEU A 138      -6.039   9.457   6.283 52.60  0.00           N
ATOM    550  CA  LEU A 138      -4.665   9.881   6.616 52.60  0.00           C
ATOM    551  C   LEU A 138      -4.353  11.332   6.246 52.60  0.00           C
ATOM    552  O   LEU A 138      -3.806  12.080   7.039 52.60  0.00           O
ATOM    553  N   PHE A 139      -4.725  11.691   5.030 56.87  0.00           N
ATOM    554  CA  PHE A 139      -4.602  13.020   4.511 56.87  0.00           C
ATOM    555  C   PHE A 139      -5.790  13.890   4.834 56.87  0.00           C
ATOM    556  O   PHE A 139      -5.772  15.074   4.508 56.87  0.00           O
ATOM    557  N   SER A 140      -6.827  13.321   5.474 58.25  0.00           N
ATOM    558  CA  SER A 140      -8.140  13.999   5.608 58.25  0.00           C
ATOM    559  C   SER A 140      -8.590  14.636   4.304 58.25  0.00           C
ATOM    560  O   SER A 140      -8.961  15.800   4.273 58.25  0.00           O
ATOM    561  N   ALA A 141      -8.519  13.878   3.227 50.28  0.00           N
ATOM    562  CA  ALA A 141      -8.945  14.332   1.917 50.28  0.00           C
ATOM    563  C   ALA A 141     -10.405  13.939   1.667 50.28  0.00           C
ATOM    564  O   ALA A 141     -10.983  13.084   2.348 50.28  0.00           O
ATOM    565  N   GLU A 142     -11.035  14.621   0.722 51.95  0.00           N
ATOM    566  CA  GLU A 142     -12.471  14.427   0.489 51.95  0.00           C
ATOM    567  C   GLU A 142     -12.738  13.477  -0.702 51.95  0.00           C
ATOM    568  O   GLU A 142     -13.747  12.791  -0.707 51.95  0.00           O
ATOM    569  N   LYS A 143     -11.805  13.444  -1.660 77.71  0.00           N
ATOM    570  CA  LYS A 143     -11.916  12.652  -2.862 77.71  0.00           C
ATOM    571  C   LYS A 143     -10.727  11.686  -3.055 77.71  0.00           C
ATOM    572  O   LYS A 143      -9.539  11.991  -2.732 77.71  0.00           O
ATOM    573  N   GLY A 144     -11.077  10.526  -3.602 59.28  0.00           N
ATOM    574  CA  GLY A 144     -10.120   9.474  -3.925 59.28  0.00           C
ATOM    575  C   GLY A 144     -10.352   8.924  -5.300 59.28  0.00           C
ATOM    576  O   GLY A 144     -11.486   8.740  -5.697 59.28  0.00           O
ATOM    577  N   ILE A 145      -9.286   8.679  -6.041 67.60  0.00           N
ATOM    578  CA  ILE A 145      -9.380   8.016  -7.325 67.60  0.00           C
ATOM    579  C   ILE A 145      -8.437   6.791  -7.270 67.60  0.00           C
ATOM    580  O   ILE A 145      -7.274   6.920  -6.924 67.60  0.00           O
ATOM    581  N   LEU A 146      -8.955   5.621  -7.649 54.97  0.00           N
ATOM    582  CA  LEU A 146      -8.233   4.396  -7.797 54.97  0.00           C
ATOM    583  C   LEU A 146      -8.251   4.098  -9.279 54.97  0.00           C
ATOM    584  O   LEU A 146      -9.304   3.843  -9.816 54.97  0.00           O
ATOM    585  N   VAL A 147      -7.117   4.065  -9.937 46.30  0.00           N
ATOM    586  CA  VAL A 147      -7.059   3.872 -11.356 46.30  0.00           C
ATOM    587  C   VAL A 147      -6.211   2.623 -11.646 46.30  0.00           C
ATOM    588  O   VAL A 147      -5.067   2.470 -11.090 46.30  0.00           O
ATOM    589  N   TYR A 148      -6.742   1.739 -12.509 50.57  0.00           N
ATOM    590  CA  TYR A 148      -6.090   0.482 -12.844 50.57  0.00           C
ATOM    591  C   TYR A 148      -5.595   0.575 -14.257 50.57  0.00           C
ATOM    592  O   TYR A 148      -6.345   0.903 -15.117 50.57  0.00           O
ATOM    593  N   ILE A 149      -4.338   0.293 -14.483 43.47  0.00           N
ATOM    594  CA  ILE A 149      -3.766   0.206 -15.807 43.47  0.00           C
ATOM    595  C   ILE A 149      -3.430  -1.275 -15.990 43.47  0.00           C
ATOM    596  O   ILE A 149      -2.545  -1.776 -15.346 43.47  0.00           O
```

ATOM	597	N	THR	A	150	-4.180	-2.003	-16.800	49.22	0.00	N
ATOM	598	CA	THR	A	150	-3.964	-3.413	-16.968	49.22	0.00	C
ATOM	599	C	THR	A	150	-3.936	-3.690	-18.433	49.22	0.00	C
ATOM	600	O	THR	A	150	-4.311	-2.846	-19.222	49.22	0.00	O
ATOM	601	N	PRO	A	151	-3.486	-4.859	-18.836	41.72	0.00	N
ATOM	602	CA	PRO	A	151	-3.299	-5.051	-20.300	41.72	0.00	C
ATOM	603	C	PRO	A	151	-4.601	-5.273	-21.077	41.72	0.00	C
ATOM	604	O	PRO	A	151	-4.598	-5.259	-22.317	41.72	0.00	O
ATOM	605	N	ASP	A	152	-5.658	-5.505	-20.333	44.73	0.00	N
ATOM	606	CA	ASP	A	152	-6.988	-5.753	-20.794	44.73	0.00	C
ATOM	607	C	ASP	A	152	-7.910	-4.564	-20.707	44.73	0.00	C
ATOM	608	O	ASP	A	152	-8.921	-4.561	-21.326	44.73	0.00	O
ATOM	609	N	ARG	A	153	-7.550	-3.582	-19.909	52.13	0.00	N
ATOM	610	CA	ARG	A	153	-8.418	-2.528	-19.517	52.13	0.00	C
ATOM	611	C	ARG	A	153	-7.808	-1.437	-18.658	52.13	0.00	C
ATOM	612	O	ARG	A	153	-7.169	-1.665	-17.715	52.13	0.00	O
ATOM	613	N	ILE	A	154	-8.107	-0.221	-18.973	42.27	0.00	N
ATOM	614	CA	ILE	A	154	-7.873	0.874	-18.098	42.27	0.00	C
ATOM	615	C	ILE	A	154	-9.177	1.197	-17.432	42.27	0.00	C
ATOM	616	O	ILE	A	154	-10.135	1.182	-18.066	42.27	0.00	O
ATOM	617	N	ALA	A	155	-9.204	1.463	-16.150	45.33	0.00	N
ATOM	618	CA	ALA	A	155	-10.490	1.563	-15.403	45.33	0.00	C
ATOM	619	C	ALA	A	155	-10.307	2.411	-14.178	45.33	0.00	C
ATOM	620	O	ALA	A	155	-9.405	2.165	-13.464	45.33	0.00	O
ATOM	621	N	GLU	A	156	-11.159	3.389	-13.945	43.25	0.00	N
ATOM	622	CA	GLU	A	156	-10.959	4.392	-12.924	43.25	0.00	C
ATOM	623	C	GLU	A	156	-12.193	4.455	-12.023	43.25	0.00	C
ATOM	624	O	GLU	A	156	-13.299	4.455	-12.446	43.25	0.00	O
ATOM	625	N	TYR	A	157	-11.976	4.532	-10.746	49.46	0.00	N
ATOM	626	CA	TYR	A	157	-13.044	4.493	-9.764	49.46	0.00	C
ATOM	627	C	TYR	A	157	-12.934	5.658	-8.804	49.46	0.00	C
ATOM	628	O	TYR	A	157	-11.853	6.011	-8.265	49.46	0.00	O
ATOM	629	N	GLU	A	158	-14.055	6.253	-8.542	47.41	0.00	N
ATOM	630	CA	GLU	A	158	-14.108	7.361	-7.646	47.41	0.00	C
ATOM	631	C	GLU	A	158	-14.532	6.786	-6.302	47.41	0.00	C
ATOM	632	O	GLU	A	158	-15.602	6.184	-6.178	47.41	0.00	O
ATOM	633	N	ILE	A	159	-13.651	6.902	-5.311	51.83	0.00	N
ATOM	634	CA	ILE	A	159	-13.878	6.419	-3.939	51.83	0.00	C
ATOM	635	C	ILE	A	159	-13.755	7.649	-3.027	51.83	0.00	C
ATOM	636	O	ILE	A	159	-12.671	8.212	-2.918	51.83	0.00	O
ATOM	637	N	ASN	A	160	-14.849	8.038	-2.394	61.93	0.00	N
ATOM	638	CA	ASN	A	160	-14.894	9.274	-1.638	61.93	0.00	C
ATOM	639	C	ASN	A	160	-15.165	9.116	-0.146	61.93	0.00	C
ATOM	640	O	ASN	A	160	-14.949	10.056	0.594	61.93	0.00	O
ATOM	641	N	GLU	A	161	-15.536	7.926	0.306	47.82	0.00	N
ATOM	642	CA	GLU	A	161	-15.720	7.704	1.724	47.82	0.00	C
ATOM	643	C	GLU	A	161	-14.467	7.151	2.407	47.82	0.00	C
ATOM	644	O	GLU	A	161	-13.997	6.052	2.116	47.82	0.00	O
ATOM	645	N	PRO	A	162	-13.950	7.900	3.365	55.75	0.00	N
ATOM	646	CA	PRO	A	162	-12.848	7.426	4.187	55.75	0.00	C
ATOM	647	C	PRO	A	162	-13.323	6.439	5.227	55.75	0.00	C
ATOM	648	O	PRO	A	162	-14.483	6.537	5.645	55.75	0.00	O
ATOM	649	N	LEU	A	163	-12.444	5.517	5.661	62.98	0.00	N
ATOM	650	CA	LEU	A	163	-12.805	4.572	6.724	62.98	0.00	C
ATOM	651	C	LEU	A	163	-12.888	5.346	8.019	62.98	0.00	C
ATOM	652	O	LEU	A	163	-12.040	6.223	8.266	62.98	0.00	O
ATOM	653	N	ASP	A	164	-13.876	5.013	8.858	51.91	0.00	N
ATOM	654	CA	ASP	A	164	-13.934	5.590	10.207	51.91	0.00	C
ATOM	655	C	ASP	A	164	-12.616	5.280	10.928	51.91	0.00	C
ATOM	656	O	ASP	A	164	-11.940	4.300	10.622	51.91	0.00	O
ATOM	657	N	GLU	A	165	-12.301	6.090	11.926	46.92	0.00	N
ATOM	658	CA	GLU	A	165	-11.127	5.880	12.758	46.92	0.00	C
ATOM	659	C	GLU	A	165	-11.240	4.594	13.565	46.92	0.00	C
ATOM	660	O	GLU	A	165	-10.265	3.923	13.761	46.92	0.00	O
ATOM	661	N	ALA	A	166	-12.438	4.252	14.016	53.66	0.00	N
ATOM	662	CA	ALA	A	166	-12.662	3.006	14.753	53.66	0.00	C
ATOM	663	C	ALA	A	166	-12.336	1.750	13.921	53.66	0.00	C
ATOM	664	O	ALA	A	166	-11.761	0.808	14.415	53.66	0.00	O
ATOM	665	N	THR	A	167	-12.694	1.751	12.648	56.37	0.00	N
ATOM	666	CA	THR	A	167	-12.382	0.638	11.768	56.37	0.00	C
ATOM	667	C	THR	A	167	-10.872	0.497	11.623	56.37	0.00	C
ATOM	668	O	THR	A	167	-10.346	-0.601	11.637	56.37	0.00	O
ATOM	669	N	ILE	A	168	-10.171	1.619	11.528	67.38	0.00	N
ATOM	670	CA	ILE	A	168	-8.732	1.633	11.358	67.38	0.00	C
ATOM	671	C	ILE	A	168	-8.078	1.128	12.626	67.38	0.00	C
ATOM	672	O	ILE	A	168	-7.058	0.457	12.570	67.38	0.00	O
ATOM	673	N	VAL	A	169	-8.665	1.441	13.778	49.35	0.00	N
ATOM	674	CA	VAL	A	169	-8.132	0.957	15.026	49.35	0.00	C
ATOM	675	C	VAL	A	169	-8.314	-0.550	15.039	49.35	0.00	C
ATOM	676	O	VAL	A	169	-7.394	-1.257	15.440	49.35	0.00	O
ATOM	677	N	ARG	A	170	-9.472	-1.045	14.590	56.01	0.00	N
ATOM	678	CA	ARG	A	170	-9.700	-2.518	14.528	56.01	0.00	C
ATOM	679	C	ARG	A	170	-8.661	-3.202	13.604	56.01	0.00	C
ATOM	680	O	ARG	A	170	-8.069	-4.207	13.996	56.01	0.00	O
ATOM	681	N	LEU	A	171	-8.396	-2.610	12.430	73.24	0.00	N
ATOM	682	CA	LEU	A	171	-7.379	-3.127	11.527	73.24	0.00	C
ATOM	683	C	LEU	A	171	-6.020	-3.139	12.210	73.24	0.00	C
ATOM	684	O	LEU	A	171	-5.257	-4.108	12.109	73.24	0.00	O
ATOM	685	N	ALA	A	172	-5.723	-2.049	12.909	49.39	0.00	N
ATOM	686	CA	ALA	A	172	-4.457	-1.898	13.604	49.39	0.00	C
ATOM	687	C	ALA	A	172	-4.301	-2.947	14.696	49.39	0.00	C
ATOM	688	O	ALA	A	172	-3.240	-3.541	14.801	49.39	0.00	O

ATOM	689	N	GLU	A	173	-5.352	-3.187	15.479	55.60	0.00	N
ATOM	690	CA	GLU	A	173	-5.292	-4.177	16.568	55.60	0.00	C
ATOM	691	C	GLU	A	173	-4.915	-5.559	16.053	55.60	0.00	C
ATOM	692	O	GLU	A	173	-4.131	-6.255	16.707	55.60	0.00	O
ATOM	693	N	ASP	A	174	-5.506	-5.961	14.919	44.03	0.00	N
ATOM	694	CA	ASP	A	174	-5.208	-7.255	14.320	44.03	0.00	C
ATOM	695	C	ASP	A	174	-3.815	-7.301	13.840	44.03	0.00	C
ATOM	696	O	ASP	A	174	-3.169	-8.343	13.873	44.03	0.00	O
ATOM	697	N	THR	A	175	-3.325	-6.165	13.384	49.85	0.00	N
ATOM	698	CA	THR	A	175	-1.966	-6.171	12.887	49.85	0.00	C
ATOM	699	C	THR	A	175	-1.026	-6.460	14.019	49.85	0.00	C
ATOM	700	O	THR	A	175	-0.104	-7.267	13.894	49.85	0.00	O
ATOM	701	N	ILE	A	176	-1.312	-5.827	15.139	17.70	0.00	N
ATOM	702	CA	ILE	A	176	-0.456	-5.907	16.306	17.70	0.00	C
ATOM	703	C	ILE	A	176	-0.618	-7.264	17.013	17.70	0.00	C
ATOM	704	O	ILE	A	176	0.348	-7.982	17.191	17.70	0.00	O
ATOM	705	N	MSE	A	177	-1.846	-7.622	17.359	27.24	0.00	N
ATOM	706	CA	MSE	A	177	-2.110	-8.862	18.066	27.24	0.00	C
ATOM	707	C	MSE	A	177	-2.036	-10.148	17.184	27.24	0.00	C
ATOM	708	O	MSE	A	177	-2.230	-11.232	17.705	27.24	0.00	O
ATOM	709	N	LEU	A	178	-1.747	-10.045	15.885	45.20	0.00	N
ATOM	710	CA	LEU	A	178	-1.765	-11.195	14.931	45.20	0.00	C
ATOM	711	C	LEU	A	178	-3.082	-11.987	14.963	45.20	0.00	C
ATOM	712	O	LEU	A	178	-3.059	-13.216	14.908	45.20	0.00	O
ATOM	713	N	GLN	A	179	-4.195	-11.295	14.914	46.50	0.00	N
ATOM	714	CA	GLN	A	179	-5.488	-11.880	15.107	46.50	0.00	C
ATOM	715	C	GLN	A	179	-6.016	-12.818	14.022	46.50	0.00	C
ATOM	716	O	GLN	A	179	-6.283	-13.971	14.291	46.50	0.00	O
ATOM	717	N	ASN	A	180	-6.209	-12.312	12.822	46.72	0.00	N
ATOM	718	CA	ASN	A	180	-6.832	-13.100	11.723	46.72	0.00	C
ATOM	719	C	ASN	A	180	-5.958	-13.170	10.495	46.72	0.00	C
ATOM	720	O	ASN	A	180	-5.982	-12.302	9.632	46.72	0.00	O
ATOM	721	N	SER	A	181	-5.162	-14.213	10.431	59.21	0.00	N
ATOM	722	CA	SER	A	181	-4.294	-14.421	9.316	59.21	0.00	C
ATOM	723	C	SER	A	181	-4.685	-15.651	8.495	59.21	0.00	C
ATOM	724	O	SER	A	181	-4.793	-16.745	9.008	59.21	0.00	O
ATOM	725	N	PRO	A	182	-4.770	-15.497	7.199	65.34	0.00	N
ATOM	726	CA	PRO	A	182	-4.417	-14.305	6.461	65.34	0.00	C
ATOM	727	C	PRO	A	182	-5.557	-13.337	6.500	65.34	0.00	C
ATOM	728	O	PRO	A	182	-6.623	-13.675	7.004	65.34	0.00	O
ATOM	729	N	ARG	A	183	-5.357	-12.135	5.998	81.95	0.00	N
ATOM	730	CA	ARG	A	183	-6.436	-11.191	6.007	81.95	0.00	C
ATOM	731	C	ARG	A	183	-7.372	-11.579	4.893	81.95	0.00	C
ATOM	732	O	ARG	A	183	-8.603	-11.628	5.107	81.95	0.00	O
ATOM	733	N	PHE	A	184	-6.785	-11.851	3.718	48.95	0.00	N
ATOM	734	CA	PHE	A	184	-7.550	-12.410	2.590	48.95	0.00	C
ATOM	735	C	PHE	A	184	-7.017	-13.778	2.185	48.95	0.00	C
ATOM	736	O	PHE	A	184	-5.814	-14.039	2.233	48.95	0.00	O
ATOM	737	N	ASN	A	185	-7.926	-14.630	1.714	52.94	0.00	N
ATOM	738	CA	ASN	A	185	-7.604	-16.007	1.378	52.94	0.00	C
ATOM	739	C	ASN	A	185	-6.563	-16.229	0.291	52.94	0.00	C
ATOM	740	O	ASN	A	185	-5.857	-17.232	0.316	52.94	0.00	O
ATOM	741	N	TRP	A	186	-6.507	-15.349	-0.690	49.34	0.00	N
ATOM	742	CA	TRP	A	186	-5.602	-15.511	-1.821	49.34	0.00	C
ATOM	743	C	TRP	A	186	-4.124	-15.190	-1.442	49.34	0.00	C
ATOM	744	O	TRP	A	186	-3.168	-15.441	-2.207	49.34	0.00	O
ATOM	745	N	GLU	A	187	-3.932	-14.615	-0.259	69.55	0.00	N
ATOM	746	CA	GLU	A	187	-2.575	-14.179	0.176	69.55	0.00	C
ATOM	747	C	GLU	A	187	-1.511	-15.229	0.225	69.55	0.00	C
ATOM	748	O	GLU	A	187	-0.428	-15.028	-0.334	69.55	0.00	O
ATOM	749	N	CYS	A	188	-1.801	-16.352	0.884	43.75	0.00	N
ATOM	750	CA	CYS	A	188	-0.760	-17.344	1.180	43.75	0.00	C
ATOM	751	C	CYS	A	188	-0.146	-17.865	-0.127	43.75	0.00	C
ATOM	752	O	CYS	A	188	1.067	-18.120	-0.218	43.75	0.00	O
ATOM	753	N	LYS	A	189	-0.970	-17.963	-1.166	20.45	0.00	N
ATOM	754	CA	LYS	A	189	-0.511	-18.522	-2.447	20.45	0.00	C
ATOM	755	C	LYS	A	189	0.696	-17.731	-3.018	20.45	0.00	C
ATOM	756	O	LYS	A	189	1.569	-18.272	-3.724	20.45	0.00	O
ATOM	757	N	TYR	A	190	0.747	-16.439	-2.708	14.03	0.00	N
ATOM	758	CA	TYR	A	190	1.763	-15.611	-3.285	14.03	0.00	C
ATOM	759	C	TYR	A	190	2.819	-15.235	-2.248	14.03	0.00	C
ATOM	760	O	TYR	A	190	3.823	-14.654	-2.584	14.03	0.00	O
ATOM	761	N	CYS	A	191	2.587	-15.625	-0.997	35.90	0.00	N
ATOM	762	CA	CYS	A	191	3.353	-15.193	0.160	35.90	0.00	C
ATOM	763	C	CYS	A	191	4.692	-15.934	0.269	35.90	0.00	C
ATOM	764	O	CYS	A	191	4.732	-17.173	0.348	35.90	0.00	O
ATOM	765	N	ILE	A	192	5.784	-15.171	0.315	22.39	0.00	N
ATOM	766	CA	ILE	A	192	7.106	-15.776	0.279	22.39	0.00	C
ATOM	767	C	ILE	A	192	7.409	-16.403	1.615	22.39	0.00	C
ATOM	768	O	ILE	A	192	8.394	-17.068	1.711	22.39	0.00	O
ATOM	769	N	PHE	A	193	6.578	-16.191	2.632	23.80	0.00	N
ATOM	770	CA	PHE	A	193	6.786	-16.794	3.943	23.80	0.00	C
ATOM	771	C	PHE	A	193	5.984	-18.074	4.210	23.80	0.00	C
ATOM	772	O	PHE	A	193	5.990	-18.637	5.317	23.80	0.00	O
ATOM	773	N	SER	A	194	5.262	-18.513	3.198	36.08	0.00	N
ATOM	774	CA	SER	A	194	4.413	-19.711	3.328	36.08	0.00	C
ATOM	775	C	SER	A	194	5.249	-20.909	3.657	36.08	0.00	C
ATOM	776	O	SER	A	194	4.748	-21.746	4.298	36.08	0.00	O
ATOM	777	N	VAL	A	195	6.503	-20.952	3.199	33.25	0.00	N
ATOM	778	CA	VAL	A	195	7.410	-21.989	3.516	33.25	0.00	C
ATOM	779	C	VAL	A	195	7.733	-22.174	4.979	33.25	0.00	C
ATOM	780	O	VAL	A	195	8.309	-23.201	5.323	33.25	0.00	O

```
ATOM    781  N   ILE A 196       7.419 -21.212   5.849 21.76  0.00           N
ATOM    782  CA  ILE A 196       7.718 -21.354   7.295 21.76  0.00           C
ATOM    783  C   ILE A 196       6.564 -20.975   8.148 21.76  0.00           C
ATOM    784  O   ILE A 196       6.683 -20.972   9.331 21.76  0.00           O
ATOM    785  N   CYS A 197       5.424 -20.671   7.561 34.17  0.00           N
ATOM    786  CA  CYS A 197       4.335 -20.157   8.329 34.17  0.00           C
ATOM    787  C   CYS A 197       3.331 -21.219   8.615 34.17  0.00           C
ATOM    788  O   CYS A 197       2.836 -21.849   7.697 34.17  0.00           O
ATOM    789  N   PRO A 198       2.960 -21.375   9.870 39.58  0.00           N
ATOM    790  CA  PRO A 198       1.970 -22.338  10.280 39.58  0.00           C
ATOM    791  C   PRO A 198       0.542 -22.046   9.971 39.58  0.00           C
ATOM    792  O   PRO A 198      -0.290 -22.938  10.156 39.58  0.00           O
ATOM    793  N   ALA A 199       0.227 -20.829   9.562 39.90  0.00           N
ATOM    794  CA  ALA A 199      -1.167 -20.431   9.353 39.90  0.00           C
ATOM    795  C   ALA A 199      -1.570 -20.467   7.877 39.90  0.00           C
ATOM    796  O   ALA A 199      -2.731 -20.267   7.563 39.90  0.00           O
ATOM    797  N   LYS A 200      -0.591 -20.655   6.991 31.81  0.00           N
ATOM    798  CA  LYS A 200      -0.778 -20.823   5.548 31.81  0.00           C
ATOM    799  C   LYS A 200      -1.946 -21.672   5.236 31.81  0.00           C
ATOM    800  O   LYS A 200      -2.051 -22.735   5.769 31.81  0.00           O
ATOM    801  N   LEU A 201      -2.799 -21.218   4.328 38.75  0.00           N
ATOM    802  CA  LEU A 201      -3.935 -22.003   3.876 38.75  0.00           C
ATOM    803  C   LEU A 201      -3.511 -23.046   2.859 38.75  0.00           C
ATOM    804  O   LEU A 201      -2.649 -22.775   2.009 38.75  0.00           O
ATOM    805  N   THR A 202      -4.127 -24.225   2.953 45.33  0.00           N
ATOM    806  CA  THR A 202      -3.796 -25.369   2.082 45.33  0.00           C
ATOM    807  C   THR A 202      -5.038 -25.920   1.370 45.33  0.00           C
ATOM    808  O   THR A 202      -6.101 -25.964   1.999 45.33  0.00           O
TER     809      THR A 202
ENDMDL
END
REMARK   1 **********************************************************************
REMARK   1 Start File NAT_vs_DEC5_dSi_colored.pdb
REMARK   1 The occupancy record was modified to represent dSi := Si(DEC)-Si(NAT).
REMARK   1 Occ=0.00 means dSi=-1.555227 kB; Occ=99.99 means dSi=1.594793 kB.
MODEL        0
ATOM      1  N   MSE A   1      23.268  -4.104 -18.521 45.76  0.00           N
ATOM      2  CA  MSE A   1      23.439  -2.722 -18.016 45.76  0.00           C
ATOM      3  C   MSE A   1      22.152  -2.161 -17.383 45.76  0.00           C
ATOM      4  O   MSE A   1      22.147  -1.650 -16.252 45.76  0.00           O
ATOM      5  N   ILE A   2      21.045  -2.254 -18.100 46.21  0.00           N
ATOM      6  CA  ILE A   2      19.816  -1.632 -17.608 46.21  0.00           C
ATOM      7  C   ILE A   2      19.269  -2.405 -16.412 46.21  0.00           C
ATOM      8  O   ILE A   2      18.935  -1.835 -15.391 46.21  0.00           O
ATOM      9  N   THR A   3      19.186  -3.703 -16.563 45.10  0.00           N
ATOM     10  CA  THR A   3      18.718  -4.606 -15.498 45.10  0.00           C
ATOM     11  C   THR A   3      19.478  -4.398 -14.212 45.10  0.00           C
ATOM     12  O   THR A   3      18.884  -4.188 -13.150 45.10  0.00           O
ATOM     13  N   GLU A   4      20.800  -4.447 -14.318 48.31  0.00           N
ATOM     14  CA  GLU A   4      21.687  -4.148 -13.195 48.31  0.00           C
ATOM     15  C   GLU A   4      21.342  -2.832 -12.484 48.31  0.00           C
ATOM     16  O   GLU A   4      21.232  -2.791 -11.242 48.31  0.00           O
ATOM     17  N   PHE A   5      21.133  -1.765 -13.247 48.37  0.00           N
ATOM     18  CA  PHE A   5      20.826  -0.486 -12.602 48.37  0.00           C
ATOM     19  C   PHE A   5      19.486  -0.520 -11.886 48.37  0.00           C
ATOM     20  O   PHE A   5      19.415  -0.122 -10.736 48.37  0.00           O
ATOM     21  N   LEU A   6      18.429  -1.039 -12.529 36.80  0.00           N
ATOM     22  CA  LEU A   6      17.100  -1.072 -11.873 36.80  0.00           C
ATOM     23  C   LEU A   6      17.119  -1.921 -10.586 36.80  0.00           C
ATOM     24  O   LEU A   6      16.551  -1.545  -9.531 36.80  0.00           O
ATOM     25  N   LEU A   7      17.767  -3.071 -10.672 54.72  0.00           N
ATOM     26  CA  LEU A   7      17.843  -3.929  -9.486 54.72  0.00           C
ATOM     27  C   LEU A   7      18.603  -3.241  -8.341 54.72  0.00           C
ATOM     28  O   LEU A   7      18.190  -3.292  -7.184 54.72  0.00           O
ATOM     29  N   LYS A   8      19.691  -2.566  -8.693 51.73  0.00           N
ATOM     30  CA  LYS A   8      20.463  -1.856  -7.680 51.73  0.00           C
ATOM     31  C   LYS A   8      19.628  -0.762  -7.044 51.73  0.00           C
ATOM     32  O   LYS A   8      19.527  -0.688  -5.819 51.73  0.00           O
ATOM     33  N   LYS A   9      18.947   0.036  -7.860 52.97  0.00           N
ATOM     34  CA  LYS A   9      18.144   1.113  -7.292 52.97  0.00           C
ATOM     35  C   LYS A   9      17.009   0.545  -6.471 52.97  0.00           C
ATOM     36  O   LYS A   9      16.708   1.086  -5.402 52.97  0.00           O
ATOM     37  N   LYS A  10      16.379  -0.547  -6.912 52.55  0.00           N
ATOM     38  CA  LYS A  10      15.262  -1.072  -6.116 52.55  0.00           C
ATOM     39  C   LYS A  10      15.660  -1.681  -4.726 52.55  0.00           C
ATOM     40  O   LYS A  10      14.931  -1.547  -3.695 52.55  0.00           O
ATOM     41  N   LEU A  11      16.798  -2.366  -4.699 38.88  0.00           N
ATOM     42  CA  LEU A  11      17.328  -2.879  -3.411 38.88  0.00           C
ATOM     43  C   LEU A  11      17.613  -1.686  -2.439 38.88  0.00           C
ATOM     44  O   LEU A  11      17.179  -1.698  -1.270 38.88  0.00           O
ATOM     45  N   GLU A  12      18.299  -0.655  -2.945 45.02  0.00           N
ATOM     46  CA  GLU A  12      18.475   0.605  -2.181 45.02  0.00           C
ATOM     47  C   GLU A  12      17.179   1.213  -1.616 45.02  0.00           C
ATOM     48  O   GLU A  12      17.048   1.390  -0.426 45.02  0.00           O
ATOM     49  N   GLU A  13      16.225   1.536  -2.461 49.97  0.00           N
ATOM     50  CA  GLU A  13      14.901   1.950  -1.974 49.97  0.00           C
ATOM     51  C   GLU A  13      14.358   1.037  -0.868 49.97  0.00           C
ATOM     52  O   GLU A  13      13.966   1.505   0.190 49.97  0.00           O
ATOM     53  N   HIS A  14      14.335  -0.267  -1.101 31.53  0.00           N
ATOM     54  CA  HIS A  14      13.794  -1.199  -0.106 31.53  0.00           C
ATOM     55  C   HIS A  14      14.560  -1.126   1.236 31.53  0.00           C
ATOM     56  O   HIS A  14      13.970  -1.122   2.322 31.53  0.00           O
```

```
ATOM     57  N   LEU A  15      15.884  -1.079   1.145 13.88  0.00           N
ATOM     58  CA  LEU A  15      16.750  -1.011   2.322 13.88  0.00           C
ATOM     59  C   LEU A  15      16.724   0.377   2.988 13.88  0.00           C
ATOM     60  O   LEU A  15      17.114   0.492   4.140 13.88  0.00           O
ATOM     61  N   SER A  16      16.283   1.421   2.277 30.48  0.00           N
ATOM     62  CA  SER A  16      16.215   2.761   2.870 30.48  0.00           C
ATOM     63  C   SER A  16      15.126   2.866   3.937 30.48  0.00           C
ATOM     64  O   SER A  16      15.184   3.766   4.746 30.48  0.00           O
ATOM     65  N   HIS A  17      14.126   1.987   3.905 47.88  0.00           N
ATOM     66  CA  HIS A  17      12.931   2.175   4.710 47.88  0.00           C
ATOM     67  C   HIS A  17      12.911   1.290   5.925 47.88  0.00           C
ATOM     68  O   HIS A  17      12.211   0.285   5.939 47.88  0.00           O
ATOM     69  N   VAL A  18      13.667   1.662   6.952 27.03  0.00           N
ATOM     70  CA  VAL A  18      13.649   0.930   8.226 27.03  0.00           C
ATOM     71  C   VAL A  18      12.455   1.416   9.073 27.03  0.00           C
ATOM     72  O   VAL A  18      12.231   2.619   9.198 27.03  0.00           O
ATOM     73  N   LYS A  19      11.711   0.480   9.654 25.84  0.00           N
ATOM     74  CA  LYS A  19      10.394   0.738  10.251 25.84  0.00           C
ATOM     75  C   LYS A  19      10.466   0.797  11.779 25.84  0.00           C
ATOM     76  O   LYS A  19      11.024  -0.102  12.394 25.84  0.00           O
ATOM     77  N   GLU A  20       9.892   1.832  12.391 52.41  0.00           N
ATOM     78  CA  GLU A  20      10.003   2.007  13.848 52.41  0.00           C
ATOM     79  C   GLU A  20       8.986   1.178  14.597 52.41  0.00           C
ATOM     80  O   GLU A  20       7.952   0.808  14.049 52.41  0.00           O
ATOM     81  N   GLU A  21       9.300   0.899  15.861 47.67  0.00           N
ATOM     82  CA  GLU A  21       8.634  -0.168  16.607 47.67  0.00           C
ATOM     83  C   GLU A  21       7.153   0.124  16.844 47.67  0.00           C
ATOM     84  O   GLU A  21       6.369  -0.811  17.040 47.67  0.00           O
ATOM     85  N   ASN A  22       6.774   1.399  16.808 55.84  0.00           N
ATOM     86  CA  ASN A  22       5.371   1.790  16.970 55.84  0.00           C
ATOM     87  C   ASN A  22       4.716   2.391  15.715 55.84  0.00           C
ATOM     88  O   ASN A  22       3.648   3.000  15.824 55.84  0.00           O
ATOM     89  N   THR A  23       5.339   2.269  14.540 52.17  0.00           N
ATOM     90  CA  THR A  23       4.636   2.674  13.297 52.17  0.00           C
ATOM     91  C   THR A  23       3.937   1.492  12.597 52.17  0.00           C
ATOM     92  O   THR A  23       4.343   0.315  12.707 52.17  0.00           O
ATOM     93  N   ILE A  24       2.863   1.841  11.907 52.05  0.00           N
ATOM     94  CA  ILE A  24       2.133   0.908  11.089 52.05  0.00           C
ATOM     95  C   ILE A  24       1.893   1.588   9.781 52.05  0.00           C
ATOM     96  O   ILE A  24       1.270   2.646   9.760 52.05  0.00           O
ATOM     97  N   TYR A  25       2.393   1.015   8.691 49.96  0.00           N
ATOM     98  CA  TYR A  25       2.072   1.558   7.393 49.96  0.00           C
ATOM     99  C   TYR A  25       0.609   1.246   7.051 49.96  0.00           C
ATOM    100  O   TYR A  25       0.029   0.273   7.510 49.96  0.00           O
ATOM    101  N   VAL A  26       0.019   2.088   6.230 44.54  0.00           N
ATOM    102  CA  VAL A  26      -1.326   1.856   5.716 44.54  0.00           C
ATOM    103  C   VAL A  26      -1.394   0.504   4.989 44.54  0.00           C
ATOM    104  O   VAL A  26      -2.320  -0.244   5.138 44.54  0.00           O
ATOM    105  N   THR A  27      -0.413   0.264   4.153 50.31  0.00           N
ATOM    106  CA  THR A  27      -0.225  -0.940   3.432 50.31  0.00           C
ATOM    107  C   THR A  27      -0.214  -2.155   4.325 50.31  0.00           C
ATOM    108  O   THR A  27      -0.816  -3.189   4.005 50.31  0.00           O
ATOM    109  N   ASP A  28       0.421  -2.066   5.476 54.19  0.00           N
ATOM    110  CA  ASP A  28       0.217  -3.125   6.447 54.19  0.00           C
ATOM    111  C   ASP A  28      -1.221  -3.366   6.899 54.19  0.00           C
ATOM    112  O   ASP A  28      -1.560  -4.494   7.172 54.19  0.00           O
ATOM    113  N   LEU A  29      -2.082  -2.355   6.958 58.99  0.00           N
ATOM    114  CA  LEU A  29      -3.418  -2.565   7.521 58.99  0.00           C
ATOM    115  C   LEU A  29      -4.304  -3.447   6.637 58.99  0.00           C
ATOM    116  O   LEU A  29      -5.335  -3.962   7.065 58.99  0.00           O
ATOM    117  N   VAL A  30      -3.930  -3.597   5.378 54.59  0.00           N
ATOM    118  CA  VAL A  30      -4.776  -4.325   4.449 54.59  0.00           C
ATOM    119  C   VAL A  30      -4.099  -5.619   4.014 54.59  0.00           C
ATOM    120  O   VAL A  30      -4.413  -6.174   2.971 54.59  0.00           O
ATOM    121  N   ARG A  31      -3.154  -6.069   4.828 99.99  0.00           N
ATOM    122  CA  ARG A  31      -2.426  -7.287   4.613 99.99  0.00           C
ATOM    123  C   ARG A  31      -2.433  -8.174   5.834 99.99  0.00           C
ATOM    124  O   ARG A  31      -2.927  -7.806   6.907 99.99  0.00           O
ATOM    125  N   CYS A  32      -1.955  -9.395   5.616 79.02  0.00           N
ATOM    126  CA  CYS A  32      -1.910 -10.426   6.637 79.02  0.00           C
ATOM    127  C   CYS A  32      -1.015  -9.951   7.770 79.02  0.00           C
ATOM    128  O   CYS A  32       0.134  -9.567   7.534 79.02  0.00           O
ATOM    129  N   PRO A  33      -1.508 -10.024   8.999 65.88  0.00           N
ATOM    130  CA  PRO A  33      -0.664  -9.610  10.083 65.88  0.00           C
ATOM    131  C   PRO A  33       0.611 -10.429  10.223 65.88  0.00           C
ATOM    132  O   PRO A  33       1.694  -9.852  10.411 65.88  0.00           O
ATOM    133  N   ARG A  34       0.512 -11.757  10.119 59.12  0.00           N
ATOM    134  CA  ARG A  34       1.689 -12.574  10.256 59.12  0.00           C
ATOM    135  C   ARG A  34       2.720 -12.198   9.218 59.12  0.00           C
ATOM    136  O   ARG A  34       3.911 -12.267   9.453 59.12  0.00           O
ATOM    137  N   ARG A  35       2.276 -11.837   8.033 93.15  0.00           N
ATOM    138  CA  ARG A  35       3.238 -11.530   6.965 93.15  0.00           C
ATOM    139  C   ARG A  35       3.971 -10.221   7.251 93.15  0.00           C
ATOM    140  O   ARG A  35       5.151 -10.042   6.901 93.15  0.00           O
ATOM    141  N   VAL A  36       3.228  -9.297   7.848 55.58  0.00           N
ATOM    142  CA  VAL A  36       3.791  -7.981   8.175 55.58  0.00           C
ATOM    143  C   VAL A  36       4.864  -8.200   9.230 55.58  0.00           C
ATOM    144  O   VAL A  36       5.972  -7.734   9.085 55.58  0.00           O
ATOM    145  N   ARG A  37       4.561  -8.972  10.259 20.52  0.00           N
ATOM    146  CA  ARG A  37       5.600  -9.259  11.227 20.52  0.00           C
ATOM    147  C   ARG A  37       6.749 -10.045  10.630 20.52  0.00           C
ATOM    148  O   ARG A  37       7.879  -9.890  11.061 20.52  0.00           O
```

```
ATOM    149  N   TYR A  38       6.481 -10.904   9.649 54.45  0.00           N
ATOM    150  CA  TYR A  38       7.570 -11.660   9.036 54.45  0.00           C
ATOM    151  C   TYR A  38       8.499 -10.724   8.363 54.45  0.00           C
ATOM    152  O   TYR A  38       9.689 -10.942   8.377 54.45  0.00           O
ATOM    153  N   GLU A  39       7.963  -9.678   7.760 73.98  0.00           N
ATOM    154  CA  GLU A  39       8.816  -8.667   7.126 73.98  0.00           C
ATOM    155  C   GLU A  39       9.769  -7.904   8.077 73.98  0.00           C
ATOM    156  O   GLU A  39      10.773  -7.394   7.597 73.98  0.00           O
ATOM    157  N   SER A  40       9.464  -7.844   9.380 37.54  0.00           N
ATOM    158  CA  SER A  40      10.398  -7.338  10.412 37.54  0.00           C
ATOM    159  C   SER A  40      11.345  -8.393  10.958 37.54  0.00           C
ATOM    160  O   SER A  40      12.540  -8.130  11.114 37.54  0.00           O
ATOM    161  N   GLU A  41      10.808  -9.578  11.263  2.00  0.00           N
ATOM    162  CA  GLU A  41      11.604 -10.637  11.865  2.00  0.00           C
ATOM    163  C   GLU A  41      12.456 -11.415  10.889  2.00  0.00           C
ATOM    164  O   GLU A  41      13.321 -12.135  11.370  2.00  0.00           O
ATOM    165  N   TYR A  42      12.238 -11.336   9.566 52.16  0.00           N
ATOM    166  CA  TYR A  42      13.052 -12.135   8.586 52.16  0.00           C
ATOM    167  C   TYR A  42      13.369 -11.290   7.378 52.16  0.00           C
ATOM    168  O   TYR A  42      13.024 -11.599   6.196 52.16  0.00           O
ATOM    169  N   LYS A  43      14.059 -10.210   7.691 61.43  0.00           N
ATOM    170  CA  LYS A  43      14.286  -9.169   6.756 61.43  0.00           C
ATOM    171  C   LYS A  43      15.175  -9.664   5.651 61.43  0.00           C
ATOM    172  O   LYS A  43      15.118  -9.127   4.534 61.43  0.00           O
ATOM    173  N   GLU A  44      16.024 -10.635   5.936 40.87  0.00           N
ATOM    174  CA  GLU A  44      16.906 -11.087   4.887 40.87  0.00           C
ATOM    175  C   GLU A  44      16.077 -11.799   3.791 40.87  0.00           C
ATOM    176  O   GLU A  44      16.309 -11.615   2.572 40.87  0.00           O
ATOM    177  N   LEU A  45      15.070 -12.551   4.234 34.19  0.00           N
ATOM    178  CA  LEU A  45      14.177 -13.244   3.311 34.19  0.00           C
ATOM    179  C   LEU A  45      13.268 -12.185   2.642 34.19  0.00           C
ATOM    180  O   LEU A  45      13.095 -12.177   1.406 34.19  0.00           O
ATOM    181  N   ALA A  46      12.710 -11.278   3.448 56.47  0.00           N
ATOM    182  CA  ALA A  46      11.893 -10.178   2.897 56.47  0.00           C
ATOM    183  C   ALA A  46      12.519  -9.534   1.649 56.47  0.00           C
ATOM    184  O   ALA A  46      11.825  -9.262   0.709 56.47  0.00           O
ATOM    185  N   ILE A  47      13.830  -9.341   1.634 44.14  0.00           N
ATOM    186  CA  ILE A  47      14.526  -8.691   0.538 44.14  0.00           C
ATOM    187  C   ILE A  47      14.299  -9.332  -0.838 44.14  0.00           C
ATOM    188  O   ILE A  47      14.451  -8.669  -1.895 44.14  0.00           O
ATOM    189  N   SER A  48      13.959 -10.620  -0.874 56.13  0.00           N
ATOM    190  CA  SER A  48      13.715 -11.277  -2.169 56.13  0.00           C
ATOM    191  C   SER A  48      12.509 -10.666  -2.882 56.13  0.00           C
ATOM    192  O   SER A  48      12.401 -10.723  -4.108 56.13  0.00           O
ATOM    193  N   GLN A  49      11.610 -10.068  -2.123 63.24  0.00           N
ATOM    194  CA  GLN A  49      10.459  -9.434  -2.743 63.24  0.00           C
ATOM    195  C   GLN A  49      10.847  -8.226  -3.623 63.24  0.00           C
ATOM    196  O   GLN A  49      10.116  -7.889  -4.482 63.24  0.00           O
ATOM    197  N   VAL A  50      12.031  -7.643  -3.424 56.27  0.00           N
ATOM    198  CA  VAL A  50      12.659  -6.700  -4.358 56.27  0.00           C
ATOM    199  C   VAL A  50      12.750  -7.176  -5.802 56.27  0.00           C
ATOM    200  O   VAL A  50      12.805  -6.373  -6.754 56.27  0.00           O
ATOM    201  N   TYR A  51      12.865  -8.468  -5.991 45.04  0.00           N
ATOM    202  CA  TYR A  51      13.231  -8.953  -7.306 45.04  0.00           C
ATOM    203  C   TYR A  51      12.000  -9.515  -8.013 45.04  0.00           C
ATOM    204  O   TYR A  51      12.116 -10.179  -9.031 45.04  0.00           O
ATOM    205  N   ALA A  52      10.823  -9.258  -7.445 44.86  0.00           N
ATOM    206  CA  ALA A  52       9.580  -9.586  -8.075 44.86  0.00           C
ATOM    207  C   ALA A  52       9.375  -8.694  -9.336 44.86  0.00           C
ATOM    208  O   ALA A  52       9.152  -7.478  -9.216 44.86  0.00           O
ATOM    209  N   PRO A  53       9.401  -9.282 -10.541 43.70  0.00           N
ATOM    210  CA  PRO A  53       9.338  -8.519 -11.804 43.70  0.00           C
ATOM    211  C   PRO A  53       8.051  -7.715 -11.969 43.70  0.00           C
ATOM    212  O   PRO A  53       8.107  -6.662 -12.589 43.70  0.00           O
ATOM    213  N   SER A  54       6.912  -8.185 -11.428 42.78  0.00           N
ATOM    214  CA  SER A  54       5.662  -7.382 -11.372 42.78  0.00           C
ATOM    215  C   SER A  54       5.807  -6.143 -10.551 42.78  0.00           C
ATOM    216  O   SER A  54       5.159  -5.116 -10.806 42.78  0.00           O
ATOM    217  N   ALA A  55       6.666  -6.210  -9.534 45.72  0.00           N
ATOM    218  CA  ALA A  55       6.871  -5.023  -8.707 45.72  0.00           C
ATOM    219  C   ALA A  55       7.756  -4.055  -9.490 45.72  0.00           C
ATOM    220  O   ALA A  55       7.598  -2.860  -9.403 45.72  0.00           O
ATOM    221  N   ILE A  56       8.662  -4.543 -10.314 37.37  0.00           N
ATOM    222  CA  ILE A  56       9.562  -3.610 -11.005 37.37  0.00           C
ATOM    223  C   ILE A  56       8.724  -2.964 -12.115 37.37  0.00           C
ATOM    224  O   ILE A  56       8.790  -1.755 -12.348 37.37  0.00           O
ATOM    225  N   LEU A  57       7.912  -3.805 -12.760 21.14  0.00           N
ATOM    226  CA  LEU A  57       6.935  -3.366 -13.747 21.14  0.00           C
ATOM    227  C   LEU A  57       6.033  -2.336 -13.087 21.14  0.00           C
ATOM    228  O   LEU A  57       5.874  -1.207 -13.590 21.14  0.00           O
ATOM    229  N   GLY A  58       5.483  -2.701 -11.946 40.76  0.00           N
ATOM    230  CA  GLY A  58       4.634  -1.806 -11.182 40.76  0.00           C
ATOM    231  C   GLY A  58       5.258  -0.473 -10.875 40.76  0.00           C
ATOM    232  O   GLY A  58       4.607   0.560 -11.036 40.76  0.00           O
ATOM    233  N   ASP A  59       6.514  -0.439 -10.443 51.13  0.00           N
ATOM    234  CA  ASP A  59       7.150   0.844 -10.105 51.13  0.00           C
ATOM    235  C   ASP A  59       7.486   1.775 -11.275 51.13  0.00           C
ATOM    236  O   ASP A  59       7.461   2.943 -11.121 51.13  0.00           O
ATOM    237  N   ILE A  60       7.874   1.212 -12.405 29.56  0.00           N
ATOM    238  CA  ILE A  60       8.072   1.923 -13.651 29.56  0.00           C
ATOM    239  C   ILE A  60       6.774   2.586 -14.133 29.56  0.00           C
ATOM    240  O   ILE A  60       6.798   3.761 -14.559 29.56  0.00           O
```

ATOM	241	N	LEU	A	61	5.641	1.870	-14.058	26.41	0.00	N
ATOM	242	CA	LEU	A	61	4.334	2.454	-14.392	26.41	0.00	C
ATOM	243	C	LEU	A	61	4.024	3.653	-13.537	26.41	0.00	C
ATOM	244	O	LEU	A	61	3.668	4.678	-14.050	26.41	0.00	O
ATOM	245	N	HIS	A	62	4.172	3.508	-12.232	53.85	0.00	N
ATOM	246	CA	HIS	A	62	3.968	4.616	-11.306	53.85	0.00	C
ATOM	247	C	HIS	A	62	4.884	5.714	-11.684	53.85	0.00	C
ATOM	248	O	HIS	A	62	4.443	6.854	-11.841	53.85	0.00	O
ATOM	249	N	LEU	A	63	6.158	5.423	-11.888	53.09	0.00	N
ATOM	250	CA	LEU	A	63	7.034	6.526	-12.325	53.09	0.00	C
ATOM	251	C	LEU	A	63	6.405	7.217	-13.537	53.09	0.00	C
ATOM	252	O	LEU	A	63	6.167	8.394	-13.497	53.09	0.00	O
ATOM	253	N	GLY	A	64	6.109	6.499	-14.603	47.77	0.00	N
ATOM	254	CA	GLY	A	64	5.719	7.158	-15.811	47.77	0.00	C
ATOM	255	C	GLY	A	64	4.400	7.890	-15.740	47.77	0.00	C
ATOM	256	O	GLY	A	64	4.174	8.755	-16.509	47.77	0.00	O
ATOM	257	N	LEU	A	65	3.499	7.454	-14.886	26.44	0.00	N
ATOM	258	CA	LEU	A	65	2.129	7.928	-14.922	26.44	0.00	C
ATOM	259	C	LEU	A	65	2.136	9.075	-13.968	26.44	0.00	C
ATOM	260	O	LEU	A	65	1.699	10.153	-14.266	26.44	0.00	O
ATOM	261	N	GLU	A	66	2.672	8.845	-12.789	54.44	0.00	N
ATOM	262	CA	GLU	A	66	3.061	9.970	-11.943	54.44	0.00	C
ATOM	263	C	GLU	A	66	3.702	11.154	-12.688	54.44	0.00	C
ATOM	264	O	GLU	A	66	3.297	12.272	-12.436	54.44	0.00	O
ATOM	265	N	SER	A	67	4.665	10.981	-13.581	47.72	0.00	N
ATOM	266	CA	SER	A	67	5.183	12.221	-14.250	47.72	0.00	C
ATOM	267	C	SER	A	67	4.100	13.012	-15.067	47.72	0.00	C
ATOM	268	O	SER	A	67	4.182	14.248	-15.203	47.72	0.00	O
ATOM	269	N	VAL	A	68	3.091	12.293	-15.600	48.52	0.00	N
ATOM	270	CA	VAL	A	68	1.939	12.934	-16.238	48.52	0.00	C
ATOM	271	C	VAL	A	68	1.158	13.773	-15.231	48.52	0.00	C
ATOM	272	O	VAL	A	68	0.645	14.865	-15.520	48.52	0.00	O
ATOM	273	N	LEU	A	69	1.040	13.216	-14.047	40.41	0.00	N
ATOM	274	CA	LEU	A	69	0.261	13.818	-13.004	40.41	0.00	C
ATOM	275	C	LEU	A	69	0.848	15.118	-12.430	40.41	0.00	C
ATOM	276	O	LEU	A	69	0.134	15.992	-11.952	40.41	0.00	O
ATOM	277	N	LYS	A	70	2.165	15.215	-12.445	41.93	0.00	N
ATOM	278	CA	LYS	A	70	2.860	16.356	-11.911	41.93	0.00	C
ATOM	279	C	LYS	A	70	2.708	17.450	-12.934	41.93	0.00	C
ATOM	280	O	LYS	A	70	2.497	18.596	-12.562	41.93	0.00	O
ATOM	281	N	GLY	A	71	2.769	17.105	-14.229	46.57	0.00	N
ATOM	282	CA	GLY	A	71	2.550	18.111	-15.305	46.57	0.00	C
ATOM	283	C	GLY	A	71	1.130	18.685	-15.376	46.57	0.00	C
ATOM	284	O	GLY	A	71	0.821	19.815	-14.923	46.57	0.00	O
ATOM	285	N	ASN	A	72	0.217	17.876	-15.876	46.68	0.00	N
ATOM	286	CA	ASN	A	72	-1.095	18.370	-16.206	46.68	0.00	C
ATOM	287	C	ASN	A	72	-2.040	18.612	-15.074	46.68	0.00	C
ATOM	288	O	ASN	A	72	-2.921	19.420	-15.211	46.68	0.00	O
ATOM	289	N	PHE	A	73	-1.880	17.933	-13.966	43.70	0.00	N
ATOM	290	CA	PHE	A	73	-2.766	18.118	-12.847	43.70	0.00	C
ATOM	291	C	PHE	A	73	-2.091	18.771	-11.639	43.70	0.00	C
ATOM	292	O	PHE	A	73	-2.674	18.861	-10.593	43.70	0.00	O
ATOM	293	N	ASN	A	74	-0.838	19.166	-11.735	53.08	0.00	N
ATOM	294	CA	ASN	A	74	-0.201	19.781	-10.582	53.08	0.00	C
ATOM	295	C	ASN	A	74	-0.355	18.949	-9.279	53.08	0.00	C
ATOM	296	O	ASN	A	74	-0.590	19.474	-8.165	53.08	0.00	O
ATOM	297	N	ALA	A	75	-0.160	17.641	-9.414	38.69	0.00	N
ATOM	298	CA	ALA	A	75	-0.115	16.792	-8.245	38.69	0.00	C
ATOM	299	C	ALA	A	75	1.282	16.777	-7.622	38.69	0.00	C
ATOM	300	O	ALA	A	75	2.270	16.992	-8.289	38.69	0.00	O
ATOM	301	N	GLU	A	76	1.336	16.530	-6.332	45.22	0.00	N
ATOM	302	CA	GLU	A	76	2.559	16.069	-5.695	45.22	0.00	C
ATOM	303	C	GLU	A	76	2.544	14.546	-5.834	45.22	0.00	C
ATOM	304	O	GLU	A	76	1.466	13.957	-5.865	45.22	0.00	O
ATOM	305	N	THR	A	77	3.710	13.915	-5.909	56.43	0.00	N
ATOM	306	CA	THR	A	77	3.794	12.484	-6.087	56.43	0.00	C
ATOM	307	C	THR	A	77	4.639	11.898	-4.978	56.43	0.00	C
ATOM	308	O	THR	A	77	5.510	12.566	-4.500	56.43	0.00	O
ATOM	309	N	GLU	A	78	4.321	10.666	-4.562	57.47	0.00	N
ATOM	310	CA	GLU	A	78	4.988	9.920	-3.517	57.47	0.00	C
ATOM	311	C	GLU	A	78	5.020	10.703	-2.232	57.47	0.00	C
ATOM	312	O	GLU	A	78	6.063	10.936	-1.623	57.47	0.00	O
ATOM	313	N	VAL	A	79	3.826	11.074	-1.816	35.16	0.00	N
ATOM	314	CA	VAL	A	79	3.635	12.010	-0.767	35.16	0.00	C
ATOM	315	C	VAL	A	79	3.474	11.303	0.544	35.16	0.00	C
ATOM	316	O	VAL	A	79	2.510	10.573	0.774	35.16	0.00	O
ATOM	317	N	GLU	A	80	4.416	11.580	1.421	42.23	0.00	N
ATOM	318	CA	GLU	A	80	4.530	10.857	2.668	42.23	0.00	C
ATOM	319	C	GLU	A	80	3.901	11.655	3.780	42.23	0.00	C
ATOM	320	O	GLU	A	80	4.090	12.840	3.853	42.23	0.00	O
ATOM	321	N	THR	A	81	3.177	11.027	4.670	32.21	0.00	N
ATOM	322	CA	THR	A	81	2.510	11.744	5.761	32.21	0.00	C
ATOM	323	C	THR	A	81	2.133	10.811	6.884	32.21	0.00	C
ATOM	324	O	THR	A	81	2.164	9.597	6.754	32.21	0.00	O
ATOM	325	N	LEU	A	82	1.667	11.373	7.976	30.57	0.00	N
ATOM	326	CA	LEU	A	82	1.644	10.601	9.202	30.57	0.00	C
ATOM	327	C	LEU	A	82	0.492	11.060	10.035	30.57	0.00	C
ATOM	328	O	LEU	A	82	0.098	12.203	9.950	30.57	0.00	O
ATOM	329	N	ARG	A	83	-0.124	10.141	10.761	43.98	0.00	N
ATOM	330	CA	ARG	A	83	-1.326	10.449	11.518	43.98	0.00	C
ATOM	331	C	ARG	A	83	-1.368	9.467	12.659	43.98	0.00	C
ATOM	332	O	ARG	A	83	-0.926	8.327	12.512	43.98	0.00	O

ATOM	333	N	GLU	A	84	-1.873	9.890	13.809	48.79	0.00	N
ATOM	334	CA	GLU	A	84	-1.727	9.022	14.978	48.79	0.00	C
ATOM	335	C	GLU	A	84	-3.042	8.425	15.425	48.79	0.00	C
ATOM	336	O	GLU	A	84	-4.110	8.996	15.183	48.79	0.00	O
ATOM	337	N	ILE	A	85	-2.918	7.275	16.095	54.30	0.00	N
ATOM	338	CA	ILE	A	85	-4.029	6.451	16.449	54.30	0.00	C
ATOM	339	C	ILE	A	85	-3.781	5.841	17.813	54.30	0.00	C
ATOM	340	O	ILE	A	85	-2.643	5.507	18.123	54.30	0.00	O
ATOM	341	N	ASN	A	86	-4.840	5.701	18.619	49.78	0.00	N
ATOM	342	CA	ASN	A	86	-4.761	5.021	19.937	49.78	0.00	C
ATOM	343	C	ASN	A	86	-5.250	3.566	19.895	49.78	0.00	C
ATOM	344	O	ASN	A	86	-6.399	3.293	19.511	49.78	0.00	O
ATOM	345	N	VAL	A	87	-4.383	2.645	20.322	52.65	0.00	N
ATOM	346	CA	VAL	A	87	-4.703	1.227	20.308	52.65	0.00	C
ATOM	347	C	VAL	A	87	-4.355	0.547	21.630	52.65	0.00	C
ATOM	348	O	VAL	A	87	-3.230	0.067	21.819	52.65	0.00	O
ATOM	349	N	GLY	A	88	-5.321	0.484	22.542	48.80	0.00	N
ATOM	350	CA	GLY	A	88	-5.066	-0.115	23.865	48.80	0.00	C
ATOM	351	C	GLY	A	88	-4.192	0.803	24.708	48.80	0.00	C
ATOM	352	O	GLY	A	88	-3.216	0.373	25.351	48.80	0.00	O
ATOM	353	N	GLY	A	89	-4.538	2.089	24.663	48.31	0.00	N
ATOM	354	CA	GLY	A	89	-3.780	3.124	25.348	48.31	0.00	C
ATOM	355	C	GLY	A	89	-2.339	3.328	24.890	48.31	0.00	C
ATOM	356	O	GLY	A	89	-1.668	4.231	25.406	48.31	0.00	O
ATOM	357	N	LYS	A	90	-1.846	2.502	23.956	50.78	0.00	N
ATOM	358	CA	LYS	A	90	-0.547	2.733	23.318	50.78	0.00	C
ATOM	359	C	LYS	A	90	-0.830	3.651	22.138	50.78	0.00	C
ATOM	360	O	LYS	A	90	-1.881	3.541	21.497	50.78	0.00	O
ATOM	361	N	VAL	A	91	0.069	4.592	21.872	52.58	0.00	N
ATOM	362	CA	VAL	A	91	-0.106	5.503	20.744	52.58	0.00	C
ATOM	363	C	VAL	A	91	0.675	4.963	19.561	52.58	0.00	C
ATOM	364	O	VAL	A	91	1.817	4.523	19.727	52.58	0.00	O
ATOM	365	N	TYR	A	92	0.052	5.005	18.377	65.64	0.00	N
ATOM	366	CA	TYR	A	92	0.676	4.480	17.190	65.64	0.00	C
ATOM	367	C	TYR	A	92	0.645	5.490	16.087	65.64	0.00	C
ATOM	368	O	TYR	A	92	-0.329	6.191	15.893	65.64	0.00	O
ATOM	369	N	LYS	A	93	1.747	5.547	15.371	72.23	0.00	N
ATOM	370	CA	LYS	A	93	1.859	6.412	14.217	72.23	0.00	C
ATOM	371	C	LYS	A	93	1.524	5.603	12.955	72.23	0.00	C
ATOM	372	O	LYS	A	93	2.270	4.714	12.581	72.23	0.00	O
ATOM	373	N	ILE	A	94	0.406	5.927	12.320	56.66	0.00	N
ATOM	374	CA	ILE	A	94	0.084	5.372	11.038	56.66	0.00	C
ATOM	375	C	ILE	A	94	0.743	6.184	9.929	56.66	0.00	C
ATOM	376	O	ILE	A	94	0.556	7.385	9.807	56.66	0.00	O
ATOM	377	N	LYS	A	95	1.497	5.507	9.097	73.79	0.00	N
ATOM	378	CA	LYS	A	95	2.305	6.166	8.106	73.79	0.00	C
ATOM	379	C	LYS	A	95	1.950	5.702	6.699	73.79	0.00	C
ATOM	380	O	LYS	A	95	1.760	4.545	6.470	73.79	0.00	O
ATOM	381	N	GLY	A	96	1.853	6.613	5.743	50.94	0.00	N
ATOM	382	CA	GLY	A	96	1.572	6.242	4.383	50.94	0.00	C
ATOM	383	C	GLY	A	96	2.142	7.179	3.378	50.94	0.00	C
ATOM	384	O	GLY	A	96	2.537	8.280	3.703	50.94	0.00	O
ATOM	385	N	ARG	A	97	2.107	6.740	2.138	36.06	0.00	N
ATOM	386	CA	ARG	A	97	2.693	7.428	1.007	36.06	0.00	C
ATOM	387	C	ARG	A	97	1.769	7.206	-0.178	36.06	0.00	C
ATOM	388	O	ARG	A	97	1.672	6.084	-0.699	36.06	0.00	O
ATOM	389	N	ALA	A	98	1.104	8.281	-0.588	44.10	0.00	N
ATOM	390	CA	ALA	A	98	0.117	8.243	-1.592	44.10	0.00	C
ATOM	391	C	ALA	A	98	0.862	8.383	-2.906	44.10	0.00	C
ATOM	392	O	ALA	A	98	1.855	9.097	-3.010	44.10	0.00	O
ATOM	393	N	ASP	A	99	0.424	7.673	-3.912	48.55	0.00	N
ATOM	394	CA	ASP	A	99	1.076	7.754	-5.196	48.55	0.00	C
ATOM	395	C	ASP	A	99	0.974	9.188	-5.720	48.55	0.00	C
ATOM	396	O	ASP	A	99	1.922	9.707	-6.350	48.55	0.00	O
ATOM	397	N	ALA	A	100	-0.166	9.846	-5.513	55.53	0.00	N
ATOM	398	CA	ALA	A	100	-0.251	11.269	-5.953	55.53	0.00	C
ATOM	399	C	ALA	A	100	-1.290	11.992	-5.184	55.53	0.00	C
ATOM	400	O	ALA	A	100	-2.232	11.366	-4.731	55.53	0.00	O
ATOM	401	N	ILE	A	101	-1.104	13.303	-4.998	45.41	0.00	N
ATOM	402	CA	ILE	A	101	-2.137	14.137	-4.384	45.41	0.00	C
ATOM	403	C	ILE	A	101	-2.390	15.420	-5.140	45.41	0.00	C
ATOM	404	O	ILE	A	101	-1.467	16.143	-5.558	45.41	0.00	O
ATOM	405	N	ILE	A	102	-3.672	15.695	-5.329	45.98	0.00	N
ATOM	406	CA	ILE	A	102	-4.083	17.004	-5.796	45.98	0.00	C
ATOM	407	C	ILE	A	102	-4.628	17.765	-4.581	45.98	0.00	C
ATOM	408	O	ILE	A	102	-5.695	17.431	-4.073	45.98	0.00	O
ATOM	409	N	ARG	A	103	-3.845	18.751	-4.103	0.00	0.00	N
ATOM	410	CA	ARG	A	103	-4.150	19.504	-2.879	0.00	0.00	C
ATOM	411	C	ARG	A	103	-5.423	20.391	-2.949	0.00	0.00	C
ATOM	412	O	ARG	A	103	-6.199	20.482	-1.960	0.00	0.00	O
ATOM	413	N	ASN	A	104	-5.626	21.051	-4.095	49.80	0.00	N
ATOM	414	CA	ASN	A	104	-6.745	21.998	-4.259	49.80	0.00	C
ATOM	415	C	ASN	A	104	-7.230	21.994	-5.679	49.80	0.00	C
ATOM	416	O	ASN	A	104	-6.693	22.692	-6.538	49.80	0.00	O
ATOM	417	N	ASP	A	105	-8.253	21.174	-5.879	49.74	0.00	N
ATOM	418	CA	ASP	A	105	-9.039	21.134	-7.084	49.74	0.00	C
ATOM	419	C	ASP	A	105	-10.471	21.605	-6.714	49.74	0.00	C
ATOM	420	O	ASP	A	105	-11.262	20.843	-6.158	49.74	0.00	O
ATOM	421	N	ASN	A	106	-10.768	22.878	-6.986	45.80	0.00	N
ATOM	422	CA	ASN	A	106	-12.040	23.506	-6.575	45.80	0.00	C
ATOM	423	C	ASN	A	106	-12.422	23.321	-5.104	45.80	0.00	C
ATOM	424	O	ASN	A	106	-13.561	23.020	-4.774	45.80	0.00	O

ATOM	425	N	GLY	A	107	-11.470	23.524	-4.215	40.33	0.00	N
ATOM	426	CA	GLY	A	107	-11.717	23.337	-2.789	40.33	0.00	C
ATOM	427	C	GLY	A	107	-11.737	21.883	-2.335	40.33	0.00	C
ATOM	428	O	GLY	A	107	-12.404	21.544	-1.352	40.33	0.00	O
ATOM	429	N	LYS	A	108	-11.008	21.019	-3.043	28.24	0.00	N
ATOM	430	CA	LYS	A	108	-10.915	19.615	-2.668	28.24	0.00	C
ATOM	431	C	LYS	A	108	-9.483	19.049	-2.884	28.24	0.00	C
ATOM	432	O	LYS	A	108	-8.779	19.403	-3.839	28.24	0.00	O
ATOM	433	N	SER	A	109	-9.039	18.236	-1.933	50.50	0.00	N
ATOM	434	CA	SER	A	109	-7.862	17.387	-2.118	50.50	0.00	C
ATOM	435	C	SER	A	109	-8.308	16.067	-2.739	50.50	0.00	C
ATOM	436	O	SER	A	109	-9.315	15.517	-2.353	50.50	0.00	O
ATOM	437	N	ILE	A	110	-7.524	15.551	-3.650	46.11	0.00	N
ATOM	438	CA	ILE	A	110	-7.790	14.265	-4.209	46.11	0.00	C
ATOM	439	C	ILE	A	110	-6.588	13.343	-3.999	46.11	0.00	C
ATOM	440	O	ILE	A	110	-5.512	13.629	-4.510	46.11	0.00	O
ATOM	441	N	VAL	A	111	-6.752	12.243	-3.266	52.77	0.00	N
ATOM	442	CA	VAL	A	111	-5.679	11.220	-3.198	52.77	0.00	C
ATOM	443	C	VAL	A	111	-5.836	10.276	-4.388	52.77	0.00	C
ATOM	444	O	VAL	A	111	-6.932	9.764	-4.636	52.77	0.00	O
ATOM	445	N	ILE	A	112	-4.775	10.018	-5.128	49.99	0.00	N
ATOM	446	CA	ILE	A	112	-4.866	9.126	-6.282	49.99	0.00	C
ATOM	447	C	ILE	A	112	-4.010	7.894	-6.056	49.99	0.00	C
ATOM	448	O	ILE	A	112	-2.842	8.019	-5.770	49.99	0.00	O
ATOM	449	N	GLU	A	113	-4.547	6.709	-6.243	56.99	0.00	N
ATOM	450	CA	GLU	A	113	-3.780	5.455	-6.060	56.99	0.00	C
ATOM	451	C	GLU	A	113	-3.823	4.710	-7.388	56.99	0.00	C
ATOM	452	O	GLU	A	113	-4.882	4.569	-8.052	56.99	0.00	O
ATOM	453	N	ILE	A	114	-2.657	4.354	-7.861	52.33	0.00	N
ATOM	454	CA	ILE	A	114	-2.531	3.758	-9.150	52.33	0.00	C
ATOM	455	C	ILE	A	114	-2.210	2.295	-8.960	52.33	0.00	C
ATOM	456	O	ILE	A	114	-1.423	1.954	-8.099	52.33	0.00	O
ATOM	457	N	LYS	A	115	-2.787	1.433	-9.786	54.13	0.00	N
ATOM	458	CA	LYS	A	115	-2.621	0.003	-9.616	54.13	0.00	C
ATOM	459	C	LYS	A	115	-2.438	-0.643	-10.947	54.13	0.00	C
ATOM	460	O	LYS	A	115	-2.964	-0.176	-11.951	54.13	0.00	O
ATOM	461	N	THR	A	116	-1.684	-1.729	-10.958	43.55	0.00	N
ATOM	462	CA	THR	A	116	-1.468	-2.466	-12.194	43.55	0.00	C
ATOM	463	C	THR	A	116	-1.602	-3.921	-11.890	43.55	0.00	C
ATOM	464	O	THR	A	116	-1.369	-4.337	-10.804	43.55	0.00	O
ATOM	465	N	SER	A	117	-2.085	-4.706	-12.802	40.11	0.00	N
ATOM	466	CA	SER	A	117	-2.109	-6.154	-12.601	40.11	0.00	C
ATOM	467	C	SER	A	117	-2.180	-6.692	-13.946	40.11	0.00	C
ATOM	468	O	SER	A	117	-2.324	-5.911	-14.909	40.11	0.00	O
ATOM	469	N	ARG	A	118	-2.032	-8.006	-14.033	45.62	0.00	N
ATOM	470	CA	ARG	A	118	-2.068	-8.640	-15.363	45.62	0.00	C
ATOM	471	C	ARG	A	118	-3.471	-8.928	-15.862	45.62	0.00	C
ATOM	472	O	ARG	A	118	-3.648	-9.162	-17.069	45.62	0.00	O
ATOM	473	N	SER	A	119	-4.457	-8.893	-14.965	45.19	0.00	N
ATOM	474	CA	SER	A	119	-5.872	-9.062	-15.362	45.19	0.00	C
ATOM	475	C	SER	A	119	-6.800	-7.967	-14.770	45.19	0.00	C
ATOM	476	O	SER	A	119	-6.533	-7.439	-13.690	45.19	0.00	O
ATOM	477	N	ASP	A	120	-7.938	-7.766	-15.424	53.65	0.00	N
ATOM	478	CA	ASP	A	120	-8.922	-6.789	-15.023	53.65	0.00	C
ATOM	479	C	ASP	A	120	-10.062	-7.464	-14.339	53.65	0.00	C
ATOM	480	O	ASP	A	120	-11.180	-6.930	-14.286	53.65	0.00	O
ATOM	481	N	LYS	A	121	-9.805	-8.622	-13.800	42.30	0.00	N
ATOM	482	CA	LYS	A	121	-10.861	-9.343	-13.147	42.30	0.00	C
ATOM	483	C	LYS	A	121	-11.305	-8.719	-11.850	42.30	0.00	C
ATOM	484	O	LYS	A	121	-10.493	-8.415	-10.994	42.30	0.00	O
ATOM	485	N	GLY	A	122	-12.595	-8.552	-11.661	49.97	0.00	N
ATOM	486	CA	GLY	A	122	-13.099	-8.150	-10.341	49.97	0.00	C
ATOM	487	C	GLY	A	122	-12.816	-6.695	-9.897	49.97	0.00	C
ATOM	488	O	GLY	A	122	-13.062	-6.363	-8.702	49.97	0.00	O
ATOM	489	N	LEU	A	123	-12.369	-5.819	-10.826	66.42	0.00	N
ATOM	490	CA	LEU	A	123	-12.047	-4.418	-10.440	66.42	0.00	C
ATOM	491	C	LEU	A	123	-13.261	-3.672	-9.972	66.42	0.00	C
ATOM	492	O	LEU	A	123	-14.252	-3.694	-10.654	66.42	0.00	O
ATOM	493	N	PRO	A	124	-13.153	-2.951	-8.838	52.81	0.00	N
ATOM	494	CA	PRO	A	124	-11.936	-2.786	-8.048	52.81	0.00	C
ATOM	495	C	PRO	A	124	-11.854	-3.776	-6.934	52.81	0.00	C
ATOM	496	O	PRO	A	124	-12.861	-4.122	-6.314	52.81	0.00	O
ATOM	497	N	LEU	A	125	-10.645	-4.184	-6.634	37.58	0.00	N
ATOM	498	CA	LEU	A	125	-10.394	-5.171	-5.542	37.58	0.00	C
ATOM	499	C	LEU	A	125	-10.497	-4.530	-4.149	37.58	0.00	C
ATOM	500	O	LEU	A	125	-10.112	-3.350	-3.908	37.58	0.00	O
ATOM	501	N	ILE	A	126	-11.048	-5.276	-3.221	55.60	0.00	N
ATOM	502	CA	ILE	A	126	-11.528	-4.647	-1.996	55.60	0.00	C
ATOM	503	C	ILE	A	126	-10.339	-4.126	-1.167	55.60	0.00	C
ATOM	504	O	ILE	A	126	-10.462	-3.123	-0.513	55.60	0.00	O
ATOM	505	N	HIS	A	127	-9.195	-4.779	-1.251	54.70	0.00	N
ATOM	506	CA	HIS	A	127	-8.055	-4.383	-0.479	54.70	0.00	C
ATOM	507	C	HIS	A	127	-7.440	-3.163	-1.103	54.70	0.00	C
ATOM	508	O	HIS	A	127	-6.834	-2.382	-0.415	54.70	0.00	O
ATOM	509	N	HIS	A	128	-7.592	-2.980	-2.400	42.39	0.00	N
ATOM	510	CA	HIS	A	128	-7.176	-1.718	-3.018	42.39	0.00	C
ATOM	511	C	HIS	A	128	-8.083	-0.558	-2.609	42.39	0.00	C
ATOM	512	O	HIS	A	128	-7.606	0.554	-2.368	42.39	0.00	O
ATOM	513	N	LYS	A	129	-9.365	-0.803	-2.526	38.83	0.00	N
ATOM	514	CA	LYS	A	129	-10.266	0.264	-2.198	38.83	0.00	C
ATOM	515	C	LYS	A	129	-10.028	0.593	-0.745	38.83	0.00	C
ATOM	516	O	LYS	A	129	-9.930	1.747	-0.408	38.83	0.00	O

```
ATOM    517  N   MSE A 130      -9.911  -0.410   0.128 53.12  0.00           N
ATOM    518  CA  MSE A 130      -9.661  -0.122   1.564 53.12  0.00           C
ATOM    519  C   MSE A 130      -8.432   0.772   1.739 53.12  0.00           C
ATOM    520  O   MSE A 130      -8.445   1.649   2.550 53.12  0.00           O
ATOM    521  N   GLN A 131      -7.394   0.520   0.958 55.83  0.00           N
ATOM    522  CA  GLN A 131      -6.133   1.211   1.097 55.83  0.00           C
ATOM    523  C   GLN A 131      -6.400   2.668   0.780 55.83  0.00           C
ATOM    524  O   GLN A 131      -5.918   3.542   1.460 55.83  0.00           O
ATOM    525  N   LEU A 132      -7.195   2.936  -0.242 51.50  0.00           N
ATOM    526  CA  LEU A 132      -7.436   4.290  -0.682 51.50  0.00           C
ATOM    527  C   LEU A 132      -8.295   4.984   0.347 51.50  0.00           C
ATOM    528  O   LEU A 132      -8.142   6.173   0.629 51.50  0.00           O
ATOM    529  N   GLN A 133      -9.173   4.244   0.963 48.48  0.00           N
ATOM    530  CA  GLN A 133     -10.034   4.835   1.951 48.48  0.00           C
ATOM    531  C   GLN A 133      -9.306   5.181   3.263 48.48  0.00           C
ATOM    532  O   GLN A 133      -9.739   6.050   4.021 48.48  0.00           O
ATOM    533  N   ILE A 134      -8.240   4.450   3.561 74.19  0.00           N
ATOM    534  CA  ILE A 134      -7.448   4.750   4.709 74.19  0.00           C
ATOM    535  C   ILE A 134      -6.635   6.008   4.375 74.19  0.00           C
ATOM    536  O   ILE A 134      -6.604   6.904   5.182 74.19  0.00           O
ATOM    537  N   TYR A 135      -5.993   6.089   3.200 50.36  0.00           N
ATOM    538  CA  TYR A 135      -5.308   7.299   2.832 50.36  0.00           C
ATOM    539  C   TYR A 135      -6.263   8.486   3.003 50.36  0.00           C
ATOM    540  O   TYR A 135      -5.876   9.536   3.592 50.36  0.00           O
ATOM    541  N   LEU A 136      -7.510   8.345   2.563 55.33  0.00           N
ATOM    542  CA  LEU A 136      -8.431   9.472   2.663 55.33  0.00           C
ATOM    543  C   LEU A 136      -8.471   9.988   4.123 55.33  0.00           C
ATOM    544  O   LEU A 136      -8.423  11.193   4.364 55.33  0.00           O
ATOM    545  N   TRP A 137      -8.562   9.085   5.079 58.33  0.00           N
ATOM    546  CA  TRP A 137      -8.471   9.465   6.480 58.33  0.00           C
ATOM    547  C   TRP A 137      -7.090  10.092   6.781 58.33  0.00           C
ATOM    548  O   TRP A 137      -6.988  11.182   7.363 58.33  0.00           O
ATOM    549  N   LEU A 138      -6.039   9.457   6.283 32.14  0.00           N
ATOM    550  CA  LEU A 138      -4.665   9.881   6.616 32.14  0.00           C
ATOM    551  C   LEU A 138      -4.353  11.332   6.246 32.14  0.00           C
ATOM    552  O   LEU A 138      -3.806  12.080   7.039 32.14  0.00           O
ATOM    553  N   PHE A 139      -4.725  11.691   5.030 45.69  0.00           N
ATOM    554  CA  PHE A 139      -4.602  13.020   4.511 45.69  0.00           C
ATOM    555  C   PHE A 139      -5.790  13.890   4.834 45.69  0.00           C
ATOM    556  O   PHE A 139      -5.772  15.074   4.508 45.69  0.00           O
ATOM    557  N   SER A 140      -6.827  13.321   5.474 48.76  0.00           N
ATOM    558  CA  SER A 140      -8.140  13.999   5.608 48.76  0.00           C
ATOM    559  C   SER A 140      -8.590  14.636   4.304 48.76  0.00           C
ATOM    560  O   SER A 140      -8.961  15.800   4.273 48.76  0.00           O
ATOM    561  N   ALA A 141      -8.519  13.878   3.227 46.70  0.00           N
ATOM    562  CA  ALA A 141      -8.945  14.332   1.917 46.70  0.00           C
ATOM    563  C   ALA A 141     -10.405  13.939   1.667 46.70  0.00           C
ATOM    564  O   ALA A 141     -10.983  13.084   2.348 46.70  0.00           O
ATOM    565  N   GLU A 142     -11.035  14.621   0.722 24.72  0.00           N
ATOM    566  CA  GLU A 142     -12.471  14.427   0.489 24.72  0.00           C
ATOM    567  C   GLU A 142     -12.738  13.477  -0.702 24.72  0.00           C
ATOM    568  O   GLU A 142     -13.747  12.791  -0.707 24.72  0.00           O
ATOM    569  N   LYS A 143     -11.805  13.444  -1.660 36.43  0.00           N
ATOM    570  CA  LYS A 143     -11.916  12.652  -2.862 36.43  0.00           C
ATOM    571  C   LYS A 143     -10.727  11.686  -3.055 36.43  0.00           C
ATOM    572  O   LYS A 143      -9.539  11.991  -2.732 36.43  0.00           O
ATOM    573  N   GLY A 144     -11.077  10.526  -3.602 50.78  0.00           N
ATOM    574  CA  GLY A 144     -10.120   9.474  -3.925 50.78  0.00           C
ATOM    575  C   GLY A 144     -10.352   8.924  -5.300 50.78  0.00           C
ATOM    576  O   GLY A 144     -11.486   8.740  -5.697 50.78  0.00           O
ATOM    577  N   ILE A 145      -9.286   8.679  -6.041 48.61  0.00           N
ATOM    578  CA  ILE A 145      -9.380   8.016  -7.325 48.61  0.00           C
ATOM    579  C   ILE A 145      -8.437   6.791  -7.270 48.61  0.00           C
ATOM    580  O   ILE A 145      -7.274   6.920  -6.924 48.61  0.00           O
ATOM    581  N   LEU A 146      -8.955   5.621  -7.649 52.71  0.00           N
ATOM    582  CA  LEU A 146      -8.233   4.396  -7.797 52.71  0.00           C
ATOM    583  C   LEU A 146      -8.251   4.098  -9.279 52.71  0.00           C
ATOM    584  O   LEU A 146      -9.304   3.843  -9.816 52.71  0.00           O
ATOM    585  N   VAL A 147      -7.117   4.065  -9.937 58.59  0.00           N
ATOM    586  CA  VAL A 147      -7.059   3.872 -11.356 58.59  0.00           C
ATOM    587  C   VAL A 147      -6.211   2.623 -11.646 58.59  0.00           C
ATOM    588  O   VAL A 147      -5.067   2.470 -11.090 58.59  0.00           O
ATOM    589  N   TYR A 148      -6.742   1.739 -12.509 50.20  0.00           N
ATOM    590  CA  TYR A 148      -6.090   0.482 -12.844 50.20  0.00           C
ATOM    591  C   TYR A 148      -5.595   0.575 -14.257 50.20  0.00           C
ATOM    592  O   TYR A 148      -6.345   0.903 -15.117 50.20  0.00           O
ATOM    593  N   ILE A 149      -4.338   0.293 -14.483 59.40  0.00           N
ATOM    594  CA  ILE A 149      -3.766   0.206 -15.807 59.40  0.00           C
ATOM    595  C   ILE A 149      -3.430  -1.275 -15.990 59.40  0.00           C
ATOM    596  O   ILE A 149      -2.545  -1.776 -15.346 59.40  0.00           O
ATOM    597  N   THR A 150      -4.180  -2.003 -16.800 50.91  0.00           N
ATOM    598  CA  THR A 150      -3.964  -3.413 -16.968 50.91  0.00           C
ATOM    599  C   THR A 150      -3.936  -3.690 -18.433 50.91  0.00           C
ATOM    600  O   THR A 150      -4.311  -2.846 -19.222 50.91  0.00           O
ATOM    601  N   PRO A 151      -3.486  -4.859 -18.836 38.79  0.00           N
ATOM    602  CA  PRO A 151      -3.299  -5.051 -20.300 38.79  0.00           C
ATOM    603  C   PRO A 151      -4.601  -5.273 -21.077 38.79  0.00           C
ATOM    604  O   PRO A 151      -4.598  -5.259 -22.317 38.79  0.00           O
ATOM    605  N   ASP A 152      -5.658  -5.505 -20.333 46.49  0.00           N
ATOM    606  CA  ASP A 152      -6.988  -5.753 -20.794 46.49  0.00           C
ATOM    607  C   ASP A 152      -7.910  -4.564 -20.707 46.49  0.00           C
ATOM    608  O   ASP A 152      -8.921  -4.561 -21.326 46.49  0.00           O
```

```
ATOM    609  N   ARG A 153      -7.550  -3.582 -19.909 48.05  0.00           N
ATOM    610  CA  ARG A 153      -8.418  -2.528 -19.517 48.05  0.00           C
ATOM    611  C   ARG A 153      -7.808  -1.437 -18.658 48.05  0.00           C
ATOM    612  O   ARG A 153      -7.169  -1.665 -17.715 48.05  0.00           O
ATOM    613  N   ILE A 154      -8.107  -0.221 -18.973 44.50  0.00           N
ATOM    614  CA  ILE A 154      -7.873   0.874 -18.098 44.50  0.00           C
ATOM    615  C   ILE A 154      -9.177   1.197 -17.432 44.50  0.00           C
ATOM    616  O   ILE A 154     -10.135   1.182 -18.066 44.50  0.00           O
ATOM    617  N   ALA A 155      -9.204   1.463 -16.150 44.74  0.00           N
ATOM    618  CA  ALA A 155     -10.490   1.563 -15.403 44.74  0.00           C
ATOM    619  C   ALA A 155     -10.307   2.411 -14.178 44.74  0.00           C
ATOM    620  O   ALA A 155      -9.405   2.165 -13.464 44.74  0.00           O
ATOM    621  N   GLU A 156     -11.159   3.389 -13.945 44.85  0.00           N
ATOM    622  CA  GLU A 156     -10.959   4.392 -12.924 44.85  0.00           C
ATOM    623  C   GLU A 156     -12.193   4.455 -12.023 44.85  0.00           C
ATOM    624  O   GLU A 156     -13.299   4.455 -12.446 44.85  0.00           O
ATOM    625  N   TYR A 157     -11.976   4.532 -10.746 45.56  0.00           N
ATOM    626  CA  TYR A 157     -13.044   4.493  -9.764 45.56  0.00           C
ATOM    627  C   TYR A 157     -12.934   5.658  -8.804 45.56  0.00           C
ATOM    628  O   TYR A 157     -11.853   6.011  -8.265 45.56  0.00           O
ATOM    629  N   GLU A 158     -14.055   6.253  -8.542 42.77  0.00           N
ATOM    630  CA  GLU A 158     -14.108   7.361  -7.646 42.77  0.00           C
ATOM    631  C   GLU A 158     -14.532   6.786  -6.302 42.77  0.00           C
ATOM    632  O   GLU A 158     -15.602   6.184  -6.178 42.77  0.00           O
ATOM    633  N   ILE A 159     -13.651   6.902  -5.311 48.46  0.00           N
ATOM    634  CA  ILE A 159     -13.878   6.419  -3.939 48.46  0.00           C
ATOM    635  C   ILE A 159     -13.755   7.649  -3.027 48.46  0.00           C
ATOM    636  O   ILE A 159     -12.671   8.212  -2.918 48.46  0.00           O
ATOM    637  N   ASN A 160     -14.849   8.038  -2.394 42.29  0.00           N
ATOM    638  CA  ASN A 160     -14.894   9.274  -1.638 42.29  0.00           C
ATOM    639  C   ASN A 160     -15.165   9.116  -0.146 42.29  0.00           C
ATOM    640  O   ASN A 160     -14.949  10.056   0.594 42.29  0.00           O
ATOM    641  N   GLU A 161     -15.536   7.926   0.306 29.62  0.00           N
ATOM    642  CA  GLU A 161     -15.720   7.704   1.724 29.62  0.00           C
ATOM    643  C   GLU A 161     -14.467   7.151   2.407 29.62  0.00           C
ATOM    644  O   GLU A 161     -13.997   6.052   2.116 29.62  0.00           O
ATOM    645  N   PRO A 162     -13.950   7.900   3.365 47.60  0.00           N
ATOM    646  CA  PRO A 162     -12.848   7.426   4.187 47.60  0.00           C
ATOM    647  C   PRO A 162     -13.323   6.439   5.227 47.60  0.00           C
ATOM    648  O   PRO A 162     -14.483   6.537   5.645 47.60  0.00           O
ATOM    649  N   LEU A 163     -12.444   5.517   5.661 43.37  0.00           N
ATOM    650  CA  LEU A 163     -12.805   4.572   6.724 43.37  0.00           C
ATOM    651  C   LEU A 163     -12.888   5.346   8.019 43.37  0.00           C
ATOM    652  O   LEU A 163     -12.040   6.223   8.266 43.37  0.00           O
ATOM    653  N   ASP A 164     -13.876   5.013   8.858 15.75  0.00           N
ATOM    654  CA  ASP A 164     -13.934   5.590  10.207 15.75  0.00           C
ATOM    655  C   ASP A 164     -12.616   5.280  10.928 15.75  0.00           C
ATOM    656  O   ASP A 164     -11.940   4.300  10.622 15.75  0.00           O
ATOM    657  N   GLU A 165     -12.301   6.090  11.926 44.28  0.00           N
ATOM    658  CA  GLU A 165     -11.127   5.880  12.758 44.28  0.00           C
ATOM    659  C   GLU A 165     -11.240   4.594  13.565 44.28  0.00           C
ATOM    660  O   GLU A 165     -10.265   3.923  13.761 44.28  0.00           O
ATOM    661  N   ALA A 166     -12.438   4.252  14.016 56.50  0.00           N
ATOM    662  CA  ALA A 166     -12.662   3.006  14.753 56.50  0.00           C
ATOM    663  C   ALA A 166     -12.336   1.750  13.921 56.50  0.00           C
ATOM    664  O   ALA A 166     -11.761   0.808  14.415 56.50  0.00           O
ATOM    665  N   THR A 167     -12.694   1.751  12.648 42.80  0.00           N
ATOM    666  CA  THR A 167     -12.382   0.638  11.768 42.80  0.00           C
ATOM    667  C   THR A 167     -10.872   0.497  11.623 42.80  0.00           C
ATOM    668  O   THR A 167     -10.346  -0.601  11.637 42.80  0.00           O
ATOM    669  N   ILE A 168     -10.171   1.619  11.528 43.48  0.00           N
ATOM    670  CA  ILE A 168      -8.732   1.633  11.358 43.48  0.00           C
ATOM    671  C   ILE A 168      -8.078   1.128  12.626 43.48  0.00           C
ATOM    672  O   ILE A 168      -7.058   0.457  12.570 43.48  0.00           O
ATOM    673  N   VAL A 169      -8.665   1.441  13.778 59.04  0.00           N
ATOM    674  CA  VAL A 169      -8.132   0.957  15.026 59.04  0.00           C
ATOM    675  C   VAL A 169      -8.314  -0.550  15.039 59.04  0.00           C
ATOM    676  O   VAL A 169      -7.394  -1.257  15.440 59.04  0.00           O
ATOM    677  N   ARG A 170      -9.472  -1.045  14.590 42.54  0.00           N
ATOM    678  CA  ARG A 170      -9.700  -2.518  14.528 42.54  0.00           C
ATOM    679  C   ARG A 170      -8.661  -3.202  13.604 42.54  0.00           C
ATOM    680  O   ARG A 170      -8.069  -4.207  13.996 42.54  0.00           O
ATOM    681  N   LEU A 171      -8.396  -2.610  12.430 50.99  0.00           N
ATOM    682  CA  LEU A 171      -7.379  -3.127  11.527 50.99  0.00           C
ATOM    683  C   LEU A 171      -6.020  -3.139  12.210 50.99  0.00           C
ATOM    684  O   LEU A 171      -5.257  -4.108  12.109 50.99  0.00           O
ATOM    685  N   ALA A 172      -5.723  -2.049  12.909 48.07  0.00           N
ATOM    686  CA  ALA A 172      -4.457  -1.898  13.604 48.07  0.00           C
ATOM    687  C   ALA A 172      -4.301  -2.947  14.696 48.07  0.00           C
ATOM    688  O   ALA A 172      -3.240  -3.541  14.801 48.07  0.00           O
ATOM    689  N   GLU A 173      -5.352  -3.187  15.479 69.67  0.00           N
ATOM    690  CA  GLU A 173      -5.292  -4.177  16.568 69.67  0.00           C
ATOM    691  C   GLU A 173      -4.915  -5.559  16.053 69.67  0.00           C
ATOM    692  O   GLU A 173      -4.131  -6.255  16.707 69.67  0.00           O
ATOM    693  N   ASP A 174      -5.506  -5.961  14.919 40.91  0.00           N
ATOM    694  CA  ASP A 174      -5.208  -7.255  14.320 40.91  0.00           C
ATOM    695  C   ASP A 174      -3.815  -7.301  13.840 40.91  0.00           C
ATOM    696  O   ASP A 174      -3.169  -8.343  13.873 40.91  0.00           O
ATOM    697  N   THR A 175      -3.325  -6.165  13.384 47.42  0.00           N
ATOM    698  CA  THR A 175      -1.966  -6.171  12.887 47.42  0.00           C
ATOM    699  C   THR A 175      -1.026  -6.460  14.019 47.42  0.00           C
ATOM    700  O   THR A 175      -0.104  -7.267  13.894 47.42  0.00           O
```

```
ATOM    701  N   ILE A 176      -1.312  -5.827  15.139 32.81  0.00           N
ATOM    702  CA  ILE A 176      -0.456  -5.907  16.306 32.81  0.00           C
ATOM    703  C   ILE A 176      -0.618  -7.264  17.013 32.81  0.00           C
ATOM    704  O   ILE A 176       0.348  -7.982  17.191 32.81  0.00           O
ATOM    705  N   MSE A 177      -1.846  -7.622  17.359 38.99  0.00           N
ATOM    706  CA  MSE A 177      -2.110  -8.862  18.066 38.99  0.00           C
ATOM    707  C   MSE A 177      -2.036 -10.148  17.184 38.99  0.00           C
ATOM    708  O   MSE A 177      -2.230 -11.232  17.705 38.99  0.00           O
ATOM    709  N   LEU A 178      -1.747 -10.045  15.885 39.02  0.00           N
ATOM    710  CA  LEU A 178      -1.765 -11.195  14.931 39.02  0.00           C
ATOM    711  C   LEU A 178      -3.082 -11.987  14.963 39.02  0.00           C
ATOM    712  O   LEU A 178      -3.059 -13.216  14.908 39.02  0.00           O
ATOM    713  N   GLN A 179      -4.195 -11.295  14.914 51.25  0.00           N
ATOM    714  CA  GLN A 179      -5.488 -11.880  15.107 51.25  0.00           C
ATOM    715  C   GLN A 179      -6.016 -12.818  14.022 51.25  0.00           C
ATOM    716  O   GLN A 179      -6.283 -13.971  14.291 51.25  0.00           O
ATOM    717  N   ASN A 180      -6.209 -12.312  12.822 45.90  0.00           N
ATOM    718  CA  ASN A 180      -6.832 -13.100  11.723 45.90  0.00           C
ATOM    719  C   ASN A 180      -5.958 -13.170  10.495 45.90  0.00           C
ATOM    720  O   ASN A 180      -5.982 -12.302   9.632 45.90  0.00           O
ATOM    721  N   SER A 181      -5.162 -14.213  10.431 62.18  0.00           N
ATOM    722  CA  SER A 181      -4.294 -14.421   9.316 62.18  0.00           C
ATOM    723  C   SER A 181      -4.685 -15.651   8.495 62.18  0.00           C
ATOM    724  O   SER A 181      -4.793 -16.745   9.008 62.18  0.00           O
ATOM    725  N   PRO A 182      -4.770 -15.497   7.199 60.53  0.00           N
ATOM    726  CA  PRO A 182      -4.417 -14.305   6.461 60.53  0.00           C
ATOM    727  C   PRO A 182      -5.557 -13.337   6.500 60.53  0.00           C
ATOM    728  O   PRO A 182      -6.623 -13.675   7.004 60.53  0.00           O
ATOM    729  N   ARG A 183      -5.357 -12.135   5.998 84.37  0.00           N
ATOM    730  CA  ARG A 183      -6.436 -11.191   6.007 84.37  0.00           C
ATOM    731  C   ARG A 183      -7.372 -11.579   4.893 84.37  0.00           C
ATOM    732  O   ARG A 183      -8.603 -11.628   5.107 84.37  0.00           O
ATOM    733  N   PHE A 184      -6.785 -11.851   3.718 52.25  0.00           N
ATOM    734  CA  PHE A 184      -7.550 -12.410   2.590 52.25  0.00           C
ATOM    735  C   PHE A 184      -7.017 -13.778   2.185 52.25  0.00           C
ATOM    736  O   PHE A 184      -5.814 -14.039   2.233 52.25  0.00           O
ATOM    737  N   ASN A 185      -7.926 -14.630   1.714 63.62  0.00           N
ATOM    738  CA  ASN A 185      -7.604 -16.007   1.378 63.62  0.00           C
ATOM    739  C   ASN A 185      -6.563 -16.229   0.291 63.62  0.00           C
ATOM    740  O   ASN A 185      -5.857 -17.232   0.316 63.62  0.00           O
ATOM    741  N   TRP A 186      -6.507 -15.349  -0.690 44.36  0.00           N
ATOM    742  CA  TRP A 186      -5.602 -15.511  -1.821 44.36  0.00           C
ATOM    743  C   TRP A 186      -4.124 -15.190  -1.442 44.36  0.00           C
ATOM    744  O   TRP A 186      -3.168 -15.441  -2.207 44.36  0.00           O
ATOM    745  N   GLU A 187      -3.932 -14.615  -0.259 82.86  0.00           N
ATOM    746  CA  GLU A 187      -2.575 -14.179   0.176 82.86  0.00           C
ATOM    747  C   GLU A 187      -1.511 -15.229   0.225 82.86  0.00           C
ATOM    748  O   GLU A 187      -0.428 -15.028  -0.334 82.86  0.00           O
ATOM    749  N   CYS A 188      -1.801 -16.352   0.884 55.08  0.00           N
ATOM    750  CA  CYS A 188      -0.760 -17.344   1.180 55.08  0.00           C
ATOM    751  C   CYS A 188      -0.146 -17.865  -0.127 55.08  0.00           C
ATOM    752  O   CYS A 188       1.067 -18.120  -0.218 55.08  0.00           O
ATOM    753  N   LYS A 189      -0.970 -17.963  -1.166 38.40  0.00           N
ATOM    754  CA  LYS A 189      -0.511 -18.522  -2.447 38.40  0.00           C
ATOM    755  C   LYS A 189       0.696 -17.731  -3.018 38.40  0.00           C
ATOM    756  O   LYS A 189       1.569 -18.272  -3.724 38.40  0.00           O
ATOM    757  N   TYR A 190       0.747 -16.439  -2.708 25.73  0.00           N
ATOM    758  CA  TYR A 190       1.763 -15.611  -3.285 25.73  0.00           C
ATOM    759  C   TYR A 190       2.819 -15.235  -2.248 25.73  0.00           C
ATOM    760  O   TYR A 190       3.823 -14.654  -2.584 25.73  0.00           O
ATOM    761  N   CYS A 191       2.587 -15.625  -0.997 41.49  0.00           N
ATOM    762  CA  CYS A 191       3.353 -15.193   0.160 41.49  0.00           C
ATOM    763  C   CYS A 191       4.692 -15.934   0.269 41.49  0.00           C
ATOM    764  O   CYS A 191       4.732 -17.173   0.348 41.49  0.00           O
ATOM    765  N   ILE A 192       5.784 -15.171   0.315 38.99  0.00           N
ATOM    766  CA  ILE A 192       7.106 -15.776   0.279 38.99  0.00           C
ATOM    767  C   ILE A 192       7.409 -16.403   1.615 38.99  0.00           C
ATOM    768  O   ILE A 192       8.394 -17.068   1.711 38.99  0.00           O
ATOM    769  N   PHE A 193       6.578 -16.191   2.632 38.05  0.00           N
ATOM    770  CA  PHE A 193       6.786 -16.794   3.943 38.05  0.00           C
ATOM    771  C   PHE A 193       5.984 -18.074   4.210 38.05  0.00           C
ATOM    772  O   PHE A 193       5.990 -18.637   5.317 38.05  0.00           O
ATOM    773  N   SER A 194       5.262 -18.513   3.198 44.71  0.00           N
ATOM    774  CA  SER A 194       4.413 -19.711   3.328 44.71  0.00           C
ATOM    775  C   SER A 194       5.249 -20.909   3.657 44.71  0.00           C
ATOM    776  O   SER A 194       4.748 -21.746   4.298 44.71  0.00           O
ATOM    777  N   VAL A 195       6.503 -20.952   3.199 51.78  0.00           N
ATOM    778  CA  VAL A 195       7.410 -21.989   3.516 51.78  0.00           C
ATOM    779  C   VAL A 195       7.733 -22.174   4.979 51.78  0.00           C
ATOM    780  O   VAL A 195       8.309 -23.201   5.323 51.78  0.00           O
ATOM    781  N   ILE A 196       7.419 -21.212   5.849 33.86  0.00           N
ATOM    782  CA  ILE A 196       7.718 -21.354   7.295 33.86  0.00           C
ATOM    783  C   ILE A 196       6.564 -20.975   8.148 33.86  0.00           C
ATOM    784  O   ILE A 196       6.683 -20.972   9.331 33.86  0.00           O
ATOM    785  N   CYS A 197       5.424 -20.671   7.561 51.61  0.00           N
ATOM    786  CA  CYS A 197       4.335 -20.157   8.329 51.61  0.00           C
ATOM    787  C   CYS A 197       3.331 -21.219   8.615 51.61  0.00           C
ATOM    788  O   CYS A 197       2.836 -21.849   7.697 51.61  0.00           O
ATOM    789  N   PRO A 198       2.960 -21.375   9.870 31.41  0.00           N
ATOM    790  CA  PRO A 198       1.970 -22.338  10.280 31.41  0.00           C
ATOM    791  C   PRO A 198       0.542 -22.046   9.971 31.41  0.00           C
ATOM    792  O   PRO A 198      -0.290 -22.938  10.156 31.41  0.00           O
```

```
ATOM    793  N   ALA A 199       0.227 -20.829   9.562 44.28  0.00           N
ATOM    794  CA  ALA A 199      -1.167 -20.431   9.353 44.28  0.00           C
ATOM    795  C   ALA A 199      -1.570 -20.467   7.877 44.28  0.00           C
ATOM    796  O   ALA A 199      -2.731 -20.267   7.563 44.28  0.00           O
ATOM    797  N   LYS A 200      -0.591 -20.655   6.991 51.19  0.00           N
ATOM    798  CA  LYS A 200      -0.778 -20.823   5.548 51.19  0.00           C
ATOM    799  C   LYS A 200      -1.946 -21.672   5.236 51.19  0.00           C
ATOM    800  O   LYS A 200      -2.051 -22.735   5.769 51.19  0.00           O
ATOM    801  N   LEU A 201      -2.799 -21.218   4.328 36.97  0.00           N
ATOM    802  CA  LEU A 201      -3.935 -22.003   3.876 36.97  0.00           C
ATOM    803  C   LEU A 201      -3.511 -23.046   2.859 36.97  0.00           C
ATOM    804  O   LEU A 201      -2.649 -22.775   2.009 36.97  0.00           O
ATOM    805  N   THR A 202      -4.127 -24.225   2.953 47.06  0.00           N
ATOM    806  CA  THR A 202      -3.796 -25.369   2.082 47.06  0.00           C
ATOM    807  C   THR A 202      -5.038 -25.920   1.370 47.06  0.00           C
ATOM    808  O   THR A 202      -6.101 -25.964   1.999 47.06  0.00           O
TER     809      THR A 202
ENDMDL
END
```
